# Mutually exclusive locales for N-linked glycans and disorder in human glycoproteins

**DOI:** 10.1038/s41598-020-61427-y

**Published:** 2020-04-08

**Authors:** Shyamili Goutham, Indu Kumari, Dharma Pally, Alvina Singh, Sujasha Ghosh, Yusuf Akhter, Ramray Bhat

**Affiliations:** 1 Department of Molecular Reproduction, Development and Genetics, Indian Institute of Sciences, Bangalore, 560012 India; 2 School of Earth and Environmental Sciences, Central University of Himachal Pradesh, District-Kangra, Shahpur, Himachal Pradesh 176206 India; 3 Department of Biotechnology, Babasaheb Bhimrao Ambedkar University, Vidya Vihar, Raebareli Road, Lucknow, Uttar Pradesh 226025 India

**Keywords:** Data mining, Protein structure predictions

## Abstract

Several post-translational protein modifications lie predominantly within regions of disorder: the biased localization has been proposed to expand the binding versatility of disordered regions. However, investigating a representative dataset of 500 human N-glycoproteins, we observed the sites of N-linked glycosylations or N-glycosites, to be predominantly present in the regions of predicted order. When compared with disordered stretches, ordered regions were not found to be enriched for asparagines, serines and threonines, residues that constitute the sequon signature for conjugation of N-glycans. We then investigated the basis of mutual exclusivity between disorder and N-glycosites on the basis of amino acid distribution: when compared with control ordered residue stretches without any N-glycosites, residue neighborhoods surrounding N-glycosites showed a depletion of bulky, hydrophobic and disorder-promoting amino acids and an enrichment for flexible and accessible residues that are frequently found in coiled structures. When compared with control disordered residue stretches without any N-glycosites, N-glycosite neighborhoods were depleted of charged, polar, hydrophobic and flexible residues and enriched for aromatic, accessible and order-promoting residues with a tendency to be part of coiled and β structures. N-glycosite neighborhoods also showed greater phylogenetic conservation among amniotes, compared with control ordered regions, which in turn were more conserved than disordered control regions. Our results lead us to propose that unique primary structural compositions and differential propensities for evolvability allowed for the mutual spatial exclusion of N-glycosite neighborhoods and disordered stretches.

## Introduction

One of the common co- and post-translational modifications of polypeptides is the conjugation of branched glycosylations to asparagines (known as N-linked glycosylations)^1^. N-linked glycosylation begins with the assembly of an oligosaccharide on dolichol pyrophosphate and the subsequent transfer of the oligosaccharide to the asparagine residues of polypeptides in the lumen of the endoplasmic reticulum; the oligosaccharide is further remodeled in the Golgi complex. Several proteins that end up in the extracellular milieu or as transmembrane proteins are N-linked glycoconjugates. The establishment of organismal morphologies has been sought to be understood through the interactions of a highly conserved set of proteins known as the developmental genetic toolkit^2^. Most toolkit proteins, which are involved in tissue-scale processes, such as cell-cell and cell-matrix adhesion, diffusion-driven signaling and cell movement, are extracellular- or membrane-bound glycoproteins^3,4^. The fundamental role of such toolkit proteins in mechanisms of organ- and organismal-development across diverse clades suggests evolutionary constraints on their structures and folds, while they may have continued to evolve to perform newer functions as organisms occupied and constructed newer niches.

A large number of eukaryotic proteins show flexible tertiary structures and are known as intrinsically disordered proteins (IDPs)^5–7^. Other proteins, while not being entirely disordered can possess variable lengths of disorder. The inherently flexible nature of disordered regions enhances the proteins’ repertoire of interacting or binding partners, and also plays an important role in the catalytic functions of the proteins^8,9^. Multiple cytoplasmic and nuclear proteins (such as those involved in signal transduction, and transcription factors) are highly disordered, although extracellular proteins have been found to also have disordered stretches^10–13^. In fact, more than 20% of extracellular proteins consist of more than 50% average disordered residues, with approximately 57% extracellular proteins containing at least one single disordered stretch of 30 continuous residues^14^. Several post-translational modifications (PTMs) have been reported to be located within disordered regions e.g., phosphorylations of serines and threonines are found within unstructured regions both in eukaryotes^15–17^ and in prokaryotes^18^. Serine/threonine phosphorylations within disordered stretches play crucial roles in the interactions between the proteins and their ligands. They can stabilize the tertiary structural organization of the disordered region in order to enhance its binding to the protein’s cognate ligand(s)^19^ besides further stabilizing the bound state of the protein-ligand complex^20^. In addition to phosphorylation, O-linked glycosylations have also been predicted to be conjugated to the disordered regions of proteins^21^ and mediate resistance to proteolysis leading to their evolutionary selection^22^.

An emerging body of literature links the contribution of N-linked glycosylation to the structure, tertiary fold and stabilization of proteins^23^. Intermediate steps in N-glycan biosynthesis act as checkpoints to ensure that only correctly folded proteins traffic along the ER-Golgi axis^24^. Thermodynamic and molecular dynamics studies show that glycans enhance the stability of the tertiary structure of proteins^22,25,26^. Keeping the above observations in mind, we asked whether the sites of N-glycan conjugation (N-glycosites) showed any bias in their locations vis-a-vis (dis)ordered regions of N-glycoproteins. Investigating a dataset of 500 representative human N-glycoproteins (out of a total of 1124 proteins with experimentally elucidated N-glycosites), wherein we mapped and predicted ordered and disordered regions, we found N-glycosites to be enriched predominantly within ordered regions relative to disordered stretches. We present biochemical (proximate) and evolutionary (distal) reasons for this enrichment and the mutual exclusion of N-glycosites and disordered regions.

## Materials and Methods

### Sequence retrieval and determination of N-glycosites

Proteins were selected from Uniprot by sequentially applying the following criteria: 1. Encoded by the human genome and 2. Proteins having at least one experimentally elucidated N-linked glycosylation. Subsequently, the disorder prediction was performed using GenesilicoMetadisorder (the best predictor of disorder based on CASP8 and CASP9), which is a meta-predictor based on the following predictors of disorder: POODLE-I, IPDA, IUPRED-I, DISPRO, POODLE-S, IUPRED-S, SPRITZ-I, PRDOS, RONN, DISOPRED2, DISEMBL AND SPRITZ-S^27^. The sequences with annotated N-glycosites and predicted disordered regions are given in Supplementary File 1. Uniprot gave the total number of human N-glycoproteins at 1124, of which the first 500 were chosen for further analysis. Amino acid enrichment was mapped using the ProtParam tool from Expasy (See Supplementary File 2)^28^. In addition, another list of proteins that have been experimentally demonstrated to have disorder was curated from the database DISPROT^29,30^ and annotated for both their N-glycosites and disordered regions (Supplementary File 3).

### Gene Ontology (GO) analysis of the proteins

For prediction of gene ontology terms, sequences of the selected proteins were scanned with the GOanna server^31^. GOanna performs a BLAST search against protein sequences that have a GO number. Gene ontological diversity was measured for the 500-protein sequence set and the complete sequence set of human proteins with experimentally elucidated N-linked glycoconjugation. The GO details of both sets are given in Supplementary File 4, wherein both sequence sets are shown to belong to an identical set of ontological categories with reasonably similar patterns of ontological representation.

### Phylogenetic analysis

The protein sequences were aligned using ClustalΩ and a phylogenetic tree was constructed using 1000 bootstrap value using MEGA5^32^. Maximum likelihood was used to construct the phylogenetic tree of the proteins as it is used for the analysis of sequences of diverse origins, while maximum parsimony method was used to construct the phylogenetic tree of the proteins which have similar functions with a comparatively higher sequence homology than the former set of sequences. The phylogenetic tree was viewed using the program FigTree (http://tree.bio.ed.ac.uk/software/figtree/). The subsets of proteins used for tree construction were constituted based on results obtained from GO analysis. Supplementary Fig. 1 represents a flow chart for the construction of phylogenetic trees.

For estimation of the conservation of disorder sites and N-linked glycosylation neighborhoods, the orthologs of each protein from three species *Pan troglodytes, Mus musculus and Gallus gallus* were obtained from Ensembl and aligned with human proteins using ClustalΩ (Supplementary File 5). The proteins that were analyzed represent a smaller subset of the complete protein list (where the separation of N-glycosites and disordered regions was established), that had 1. orthologs across all the three above-mentioned species and 2. showed a non-overlapping separation between the N-glycosite neighborhoods and disordered regions. Shannon information entropy was measured for randomly chosen 11-amino acid ordered and disordered stretches (1 each for every protein) with no N-glycosites, using Protein Variability Server (http://molbiol.edu.ru/eng/index.html) (Supplementary File 6).

### Amino acid residue frequency analysis

The comparison of frequency of occurrence of amino acids within N-glycosite neighborhoods, N-glycosite-less ordered and disordered regions was performed using Composition Profiler (http://www.cprofiler.org/)^33^. Bootstrap iterations were kept at 10,000, significance value was kept at 0.05 and Bonferroni’s multiple-comparison correction was applied.

### Protein functional site prediction

In order to compute the probability that the residue combinations constituting disordered patches or N-linked glycosylation neighborhoods likely represent clusters of function-determining residues, full length protein sequences were input into the Universal Evolutionary Trace server (http://lichtargelab.org/software/uet)^34^. The evolutionary trace of residues that varies among evolutionarily distant protein homologs is ranked higher than for residues that vary among evolutionarily proximal protein homologs^35^. The program searches for all homologous sequences within the Uniref90 database (with a minimum e-value cutoff of 1e-250) up to 500 sequences.The mean of residue ranks was computed and compared between residues from the disordered control regions, ordered control regions and residues flanking N-glycosite neighborhoods (Supplementary File 7).

### Statistics

We used both descriptive statistics and significance tests to ascertain the differences in residue characteristics between N-glycosite neighborhoods, ordered and disordered regions. For each protein, the mean value of a given characteristic was measured across a residue stretch and when comparing across protein sets, the mean, median and standard error of the mean values were calculated. In order to measure significance, the non-parametric Wilcoxon’s matched-pairs signed rank test was performed. Significance was measured through P value using GraphPad Prism software. For representation, box and whisker plots were selected in order to show the interquartile range that provides a comprehensive understanding of the difference in values between two large sets of data with wide valuedistributions.

## Results

### N-glycosites are predominantly enriched in ordered regions of N-glycoproteins

**Figure 1:**
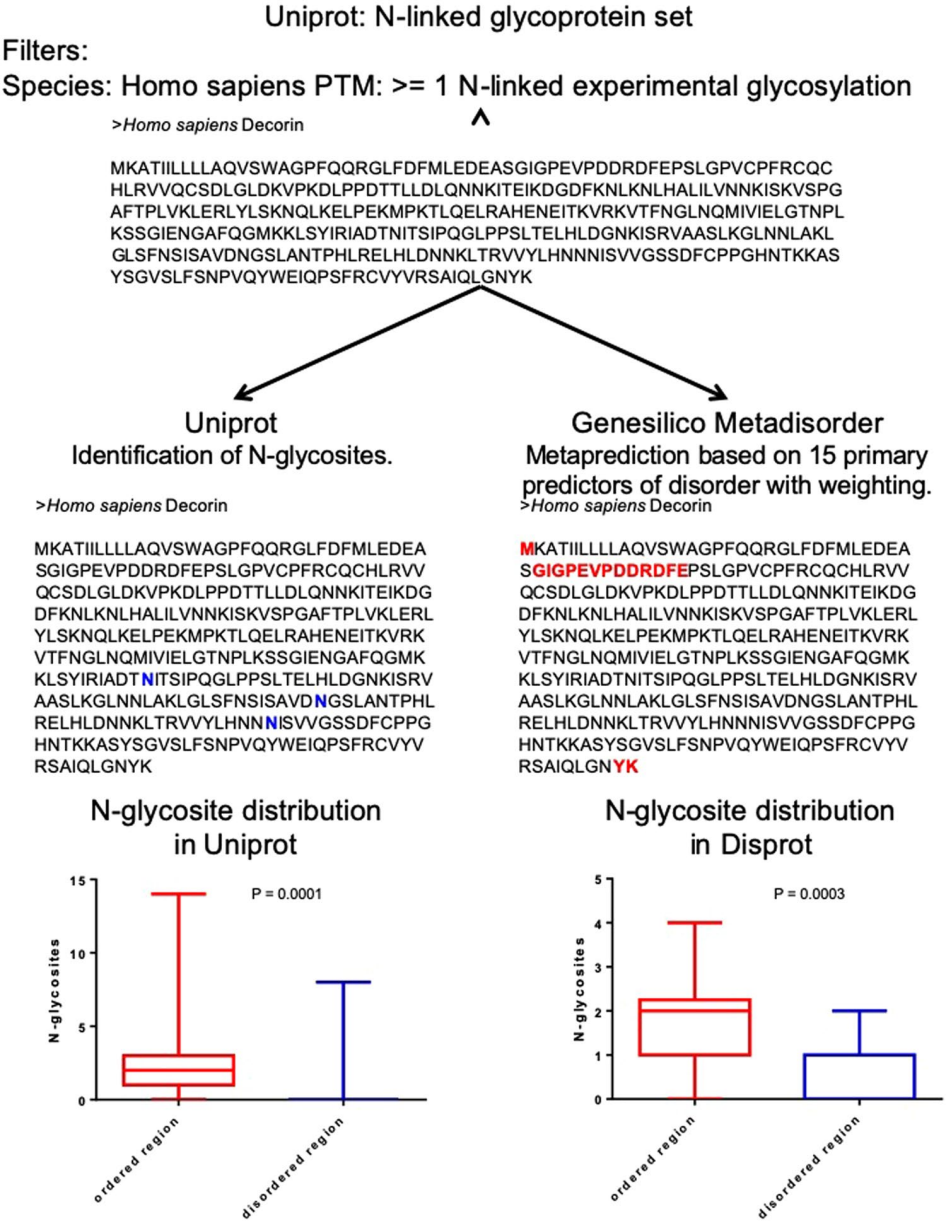
Workflow for the determination of the exclusion between N-glycosites and predicted disordered stretches A list of proteins was compiled from Uniprot based on the criteria that they were encoded by the human genome and at the same time had at least one N-linked glycosylation established through experimental elucidation. Subsequently, one of or more N-glycosites were annotated in its amino acid sequence. The sequence was also submitted to Genesilico Metadisorder in order to annotate the predicted disordered residue stretches. Bottom left: Box and whisker plot representation of the relative occurrence of experimentally established N-glycosites in ordered versus disordered regions of the 500 human N-glycoprotein set from Uniprot. Bottom right: Box and whisker plot representation of the relative occurrence of experimentally established N-glycosites in ordered versus disordered regions of the 18-protein dataset from Disprot. For both graphs, boxes represent interquartile range and whiskers extend from minimum to maximum values. Statistical significance is given by P-value measured using Wilcoxon’s matched-pairs signed rank test.

We used UniProt to collate a list of all human glycoproteins with at least one experimentally determined N-glycosite. Among 1124 such N-glycoproteins, 500 proteins representing the diverse biological function of the complete dataset were processed through in Silico Metadisorder to predict the amino acids that constituted disordered sequences. We then annotated each N-glycoprotein for (dis)order and N-glycosite location. N-linked glycoconjugated asparagines (or N-glycosites) were found to be significantly enriched within ordered regions. (Fig. 1; Supplementary File 1). Most prediction and meta-prediction algorithms tend to ‘overpredict’ disorder, i.e., the possibility of false positives is higher: therefore, the enrichment of N-glycan-conjugated asparagines within ordered stretches could be even higher than our observation. We then took a complementary approach: upon searching DISPROT, a database of proteins with experimentally annotated disordered regions^30^, we identified a set of eighteen human proteins with varying extents of disorder and also with at least one N-glycosite. In these eighteen proteins, N-glycosites were also found to be enriched significantly in the ordered regions (Fig. 1; Supplementary File 3).

In order to test whether the exclusion (or the less common inclusion) of N-glycosites from (and within) disordered stretches is a characteristic of one or more phylogenetically conserved protein families, we classified our protein set on the basis of gene ontologies. We observed that our analyzed set of N-glycoproteins perform a diverse range of predicted biological functions (Supplementary File 4). Subsets of proteins within each ontological classifier varied in their phylogenetic relatedness: for example, proteins related to immune system function were closely related (Supplementary Fig. 2 top), however, those that clustered together under the ontological heading of metabolic functions were very distantly related (Supplementary Fig. 2 bottom). Even a comparatively limited set of proteins with N-glycosites in their disordered regions were distributed across a wide set of ontological categories. This suggested that N-glycoproteins with disordered stretches represented a very diverse set of phylogenetically distant proteins.

The residue signature for N-linked glycosylation has been established to be asparagine, followed by any amino acid except proline (as it introduces a kink in the protein structure), followed in turn by a serine (Ser) or threonine (Thr) (i.e., N(X-P)S/T)^36^. Therefore, we asked whether the differential enrichment for N-glycosites could simply be reconciled by enrichment of asparagine, serine and/or threonine within ordered stretches of proteins due to their inherent biochemical difference from disordered stretches.

### N-linked glycoconjugation sequon but not its individual amino acid constituents are enriched within ordered regions

**Figure 2:**
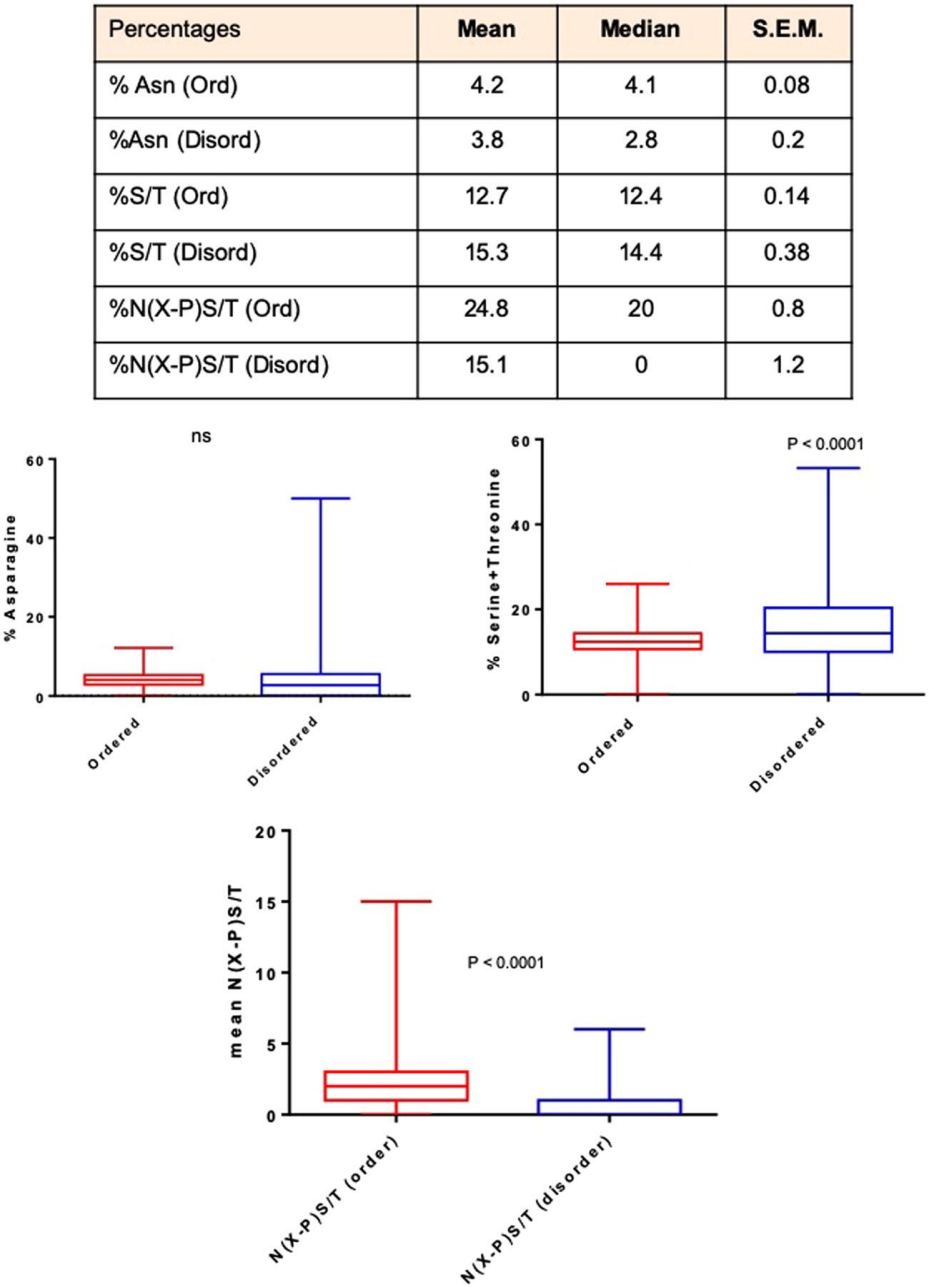
The N-linked glycosylation sequon but not the individual sequon residues show enrichment in the ordered regions (Top) Table showing the mean, median and standard error of mean in the percentage of Asparagines (Asn), Serines (Ser) and Threonines (Thr) and the N-linked glycosylation sequon (N(X-P)S/T) within the ordered and disordered regions of the 500 human N-glycoprotein set. The percentage of asparagine, serine and threonine and N(X-P)S/T for each protein is provided in Supplementary File 3. (Bottom) Box and whisker plot representation of the relative proportion of asparagine, serine and threonine, and N(X-P)S/T within the 500 human N-glycoprotein subset. For the graphs, boxes represent interquartile range and whiskers extend from minimum to maximum values. Statistical significance is given by P-value measured using Wilcoxon’s matched-pairs signed rank test.

There are several descriptors of protein disorder; one of the dominantly used descriptors is that of a consecutive stretch of polar and/or charged amino acids^37^. Since asparagine, serine and threonine are polar in nature, we estimated the abundance of these three amino acids within ordered and disordered segments of our protein dataset. The percentage of asparagine was found to be insignificantly different between disordered and ordered regions of N-glycoproteins. Serine and threonine were surprisingly found to be significantly enriched within disordered- rather than ordered- stretches (Fig. 2; Supplementary File 2). This discounted the hypothesis that the relative enrichment in N-linked glycosylation in ordered regions could be the result of a specific enrichment of asparagine, serine and/or threonine.

We then asked what fractions of asparagines participate in constituting a N(X-P)S/T sequon within ordered and disordered regions. Despite an unbiased distribution of asparagine between ordered and disordered regions, and relative enrichment of serine and threonine within the disordered region, the probability of constitution of the minimal signature required for N-glycan conjugation (N(X-P)S/T) was found to be significantly higher within ordered regions (Fig. 2).

We then probed for the relative proportion of individual amino acids that occupy the “X” of the sequon (N(X-P)S/T). Empirical and bioinformatic analysis with model and natural proteins, respectively, have suggested that the identity of “X” in the sequon may determine the extent of its glycosylation: bulky, hydrophobic or acidic amino acids may reduce the efficiency of glycosylation. Conversely, small, hydrophilic or basic amino acids being present as X may increase the propensity of the sequon to be glycosylated^38,39^. We analyzed the identity of X in the sequons that are present in predicted regions of order and disorder: we found higher proportion of hydrophobic and bulky residues as X in the sequons inhabiting the ordered regions compared with the disordered region (Supplementary File 9). The proportion of acidic and basic amino acids as X was higher in the sequons from the disordered region. These findings are consistent with the amino acid compositions of the ordered and disordered regions. Therefore, these properties of the sequon glycosylation do not explain why N-linked glycosylation predominates in ordered regions. We then asked if the residues surrounding N-linked glycosylation sequons were biochemically distinct from those constituting the disordered regions of proteins.

### N-glycosite neighborhoods have unique residue compositions based on polarity, length of side chain and aromaticity

We began by annotating five residues upstream- and downstream of the glycoconjugated asparagines as N-glycosite neighborhoods of proteins in our dataset. Additionally 11-residue stretches that did not contain N-glycosites were also identified in the disordered and ordered regions and were defined as disordered and ordered control regions respectively. We estimated the relative frequencies of amino acids between these three 11-residue subsets across our 500-protein dataset and estimated the significance in enrichment or deletion of residue properties such as aromaticity, polarity^40^, charge, hydrophobicity^41^, flexibility^42^, surface exposure^43^, normalized frequency of occurrence in β structures and coils^44^, propensities to contribute to order^45^, and bulkiness^40^.

We first compared the set of ordered control regions with disordered control regions (Supplementary Figs. 3 and 4). The former showed an enrichment of aromatic, bulky, hydrophobic, order-promoting residues with propensity to participate in β structures. It also showed a depletion for charged, polar and flexible residues that are exposed, promote disorder and participate in coil secondary structures. These observations served to confirm the veracity of our controls.

**Figure 3:**
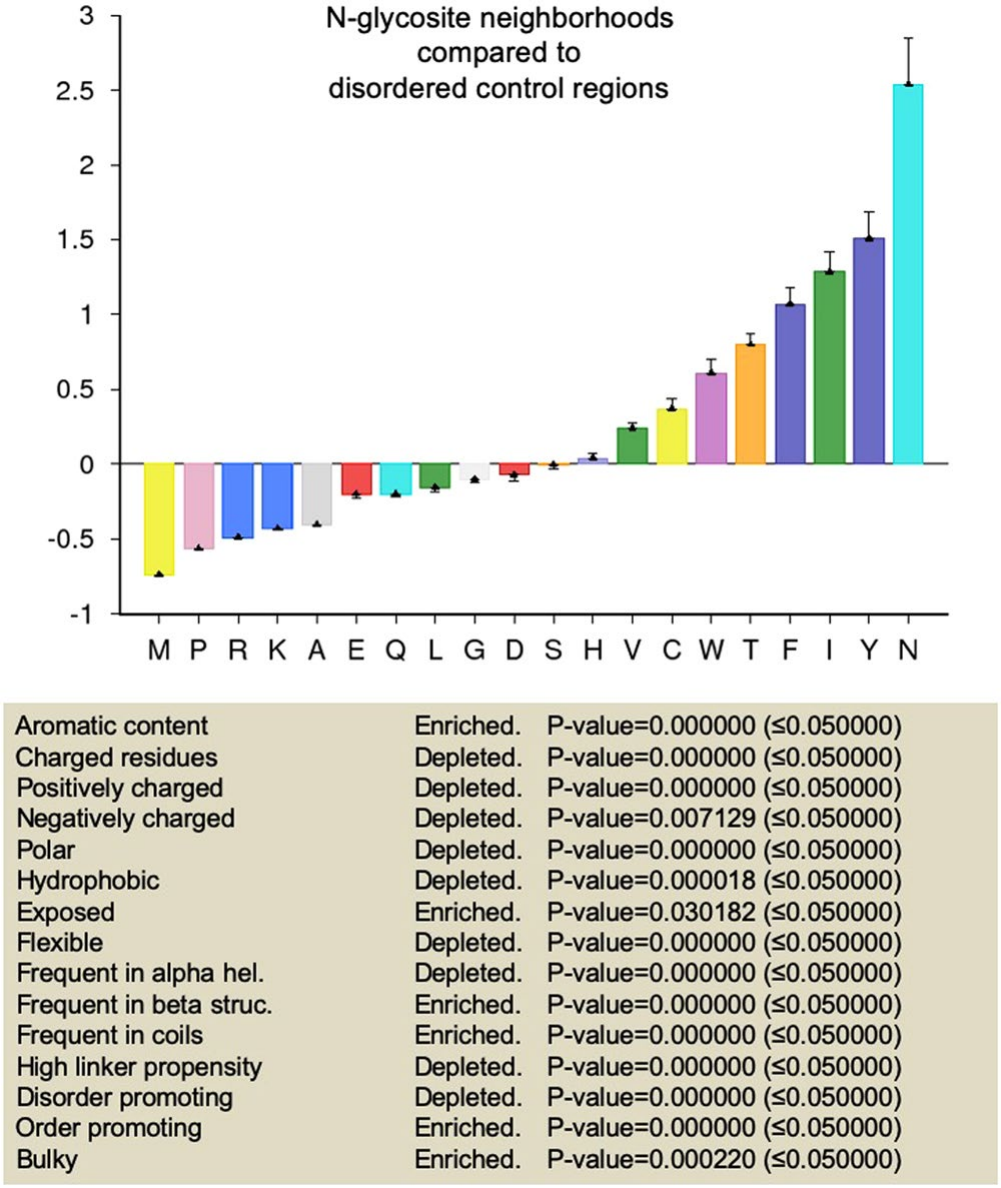
N-glycosite neighborhoods compared with disordered control regions show unique amino acid compositions. (Top) Graphical representation of fractional enrichment of amino acids in 11-residue N-glycosite-centered neighborhoods within ordered regions normalized to disordered control regions of similar length (containing no N-glycosites) (see also Supplementary Fig. 5 for tabular representation signifying enrichment and depletion of individual amino acids for the above comparison with significance). (Bottom) Tabular representation signifying enrichment and depletion of residue properties based on the above amino acid enrichment and depletion profile. Error bars represent SD. Statistical significance is given by P-value measured using two-way t-test (see^33^).

**Figure 4:**
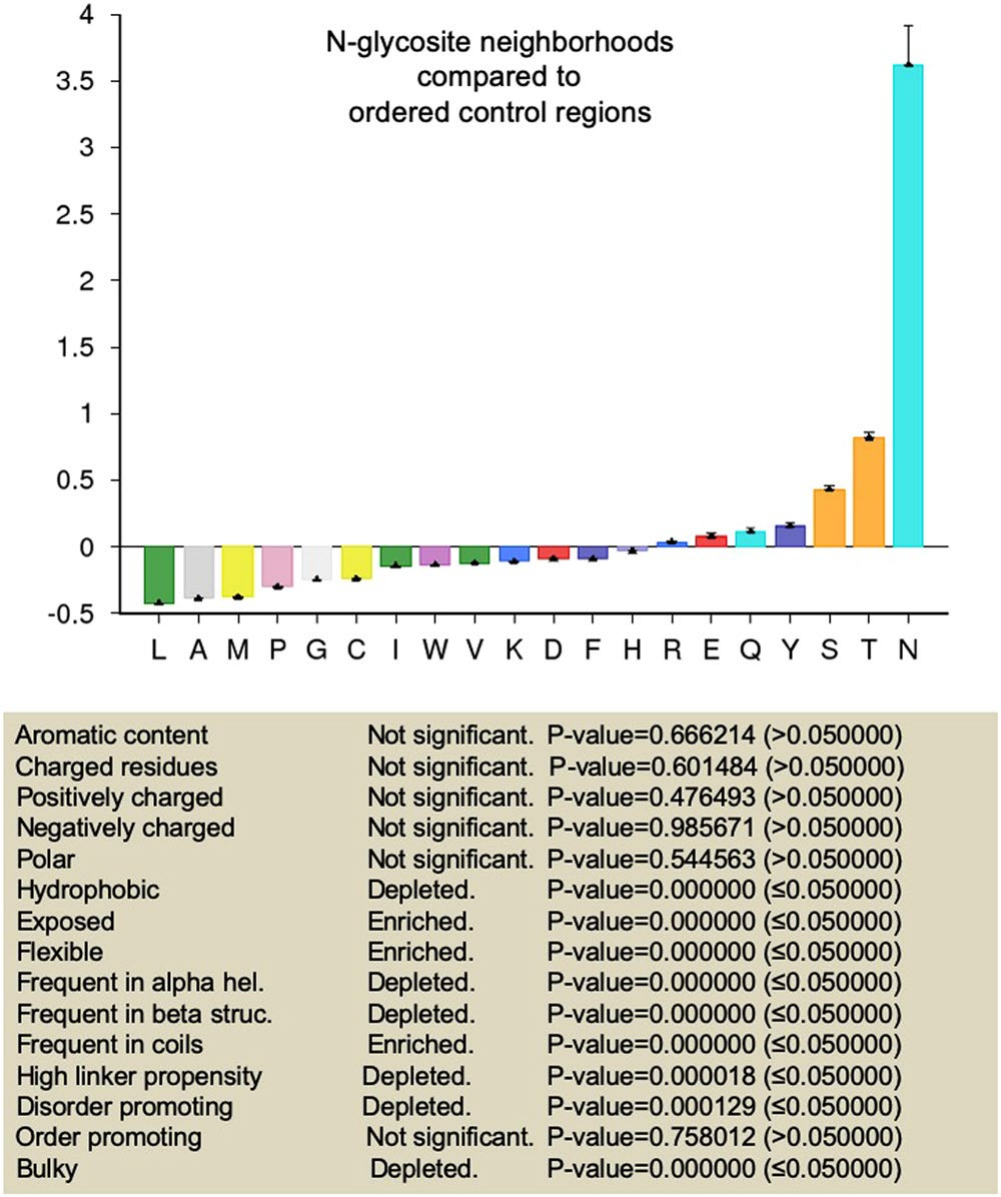
N-glycosite neighborhoods compared with ordered control regions show unique amino acid compositions. (Top) Graphical representation of fractional enrichment of amino acids in 11-residue N-glycosite-centered neighborhoods within ordered regions normalized to ordered control regions of similar length (containing no N-glycosites) (see also Supplementary Fig. 6 for tabular representation signifying enrichment and depletion of individual amino acids for the above comparison with significance). (Bottom) Tabular representation signifying enrichment and depletion of residue properties based on the above amino acid enrichment and depletion profile. Error bars represent SD. Statistical significance is given by P-value measured using two-way t-test (see^33^).

In comparison with disordered control region residues, N-glycosite neighborhoods were found to be enriched for aromatic, bulky and order-promoting amino acids that showed a propensity for access and being part of β structures and coils (Fig. 3 and Supplementary Fig. 5). N-glycosite neighborhoods also showed a depletion for charged, polar and flexible disorder-promoting residues (Fig. 3 and Supplementary Fig. 5). N-glycosites in ordered regions when compared with the less commonly found N-glycosites in disordered regions were significantly enriched also for bulky, hydrophobic and order-promoting residues and depleted of surface-exposed, flexible, disorder-promoting amino acids (Supplementary File 8). When compared with ordered control regions, N-glycosite neighborhoods showed an enrichment for flexible and exposed residues with a tendency to be part of coils (Fig. 4 and Supplementary Fig. 6). As well, N-glycosite neighborhoods were relatively depleted of hydrophobic and bulky residues as well as those that participate in β structures (Fig. 4 and Supplementary Fig. 6).

Ranking the three datasets (N-glycosite neighborhoods, ordered- and disordered- control regions) together, based on the above properties revealed two broad signatures (Supplementary Fig. 7): The first signature represented properties wherein the disordered control regions were significantly different from both the other regions (Supplementary Fig. 7 left). Aromatic and order-promoting amino acids were specifically depleted in disordered control regions. The latter also showed an enrichment for charged and polar amino acids, relative to the other two regions (between which such properties were insignificantly distinct). Properties such as bulkiness, flexibility, promotion of disorder and a tendency to be part of linker regions were also changed to the greatest extent in the disordered control regions, albeit with relative and significant differences between ordered regions and N-glycosite neighborhoods. The other signature consisted of properties for which N-glycosite neighborhoods and ordered control regions represented the ends of the comparative spectrum of differences with disordered control regions showing intermediate values (Supplementary Fig. 7 right). These were residue hydrophobicity, exposure, and the propensity to be part of coil structures.

Having ascertained the differences in primary structure between the three sequence sets, we sought to investigate their propensity for being locales where function-determining residues tend to cluster together.

### Disordered regions show higher clustering of function-determining residues; N-glycosite neighborhoods show greater evolutionary conservation within the tetrapod clade

**Figure 5:**
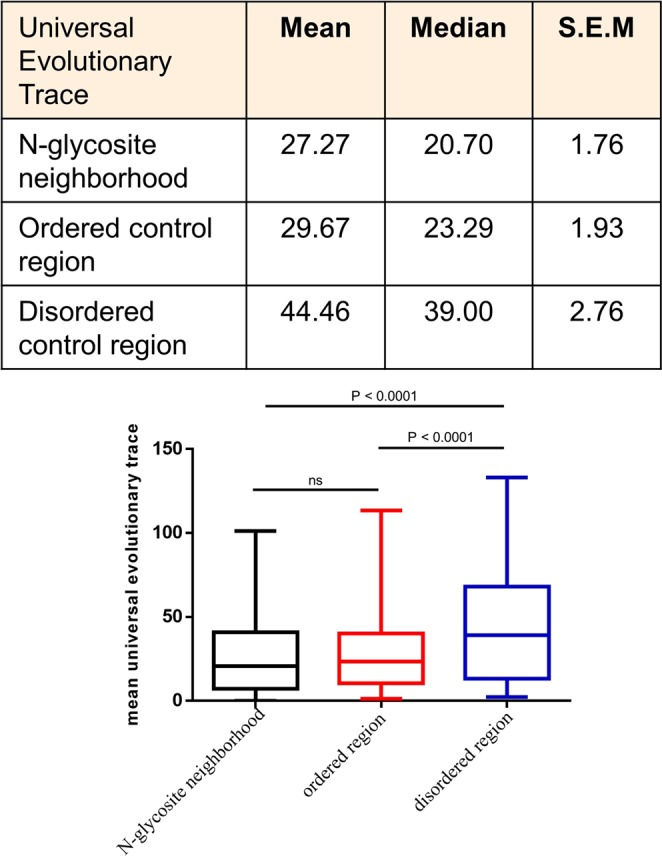
Disordered regions show greater ability to form functionally relevant residual clusters than ordered regions with and without N-glycosites (Top) Table showing mean and median universal evolutionary trace and the standard error of mean of N-glycosite neighborhoods, 11-residue ordered and disordered regions with N-glycosites. (Bottom) Box and whisker representation showing differences in mean universal evolutionary traces of N-glycosite neighborhoods, 11-residue ordered and disordered regions with N-glycosites. The mean evolutionary trace for each protein is provided in Supplementary File 5. For the graph, boxes represent interquartile range and whiskers extend from minimum to maximum values. Statistical significance is given by P-value measured using Wilcoxon’s matched-pairs signed rank test.

We used universal evolutionary trace, an algorithm that identifies and ranks each amino acid within a protein that determines its function^34^. This functional importance, is therefore not centered exclusively on conservation, but on the fact that variation in evolutionarily distant homologs ranks a residue higher than variation in evolutionarily closer homologs in being able to be part of a functional interface. Such important amino acids potentially cluster together allowing prediction of putative functional motifs for proteins that have not been crystallized. We estimated that the mean evolutionary trace ranks of residues that constituted the control order regions and those that constituted N-glycosite neighborhoods were not significantly different. On the other hand, the mean trace of the disordered control sequence set was significantly higher than both the ordered subsets (Fig. 5; Supplementary File 5). This suggested that disordered regions scored higher than similar length stretches in the ordered regions in terms of determining protein function.

**Figure 6:**
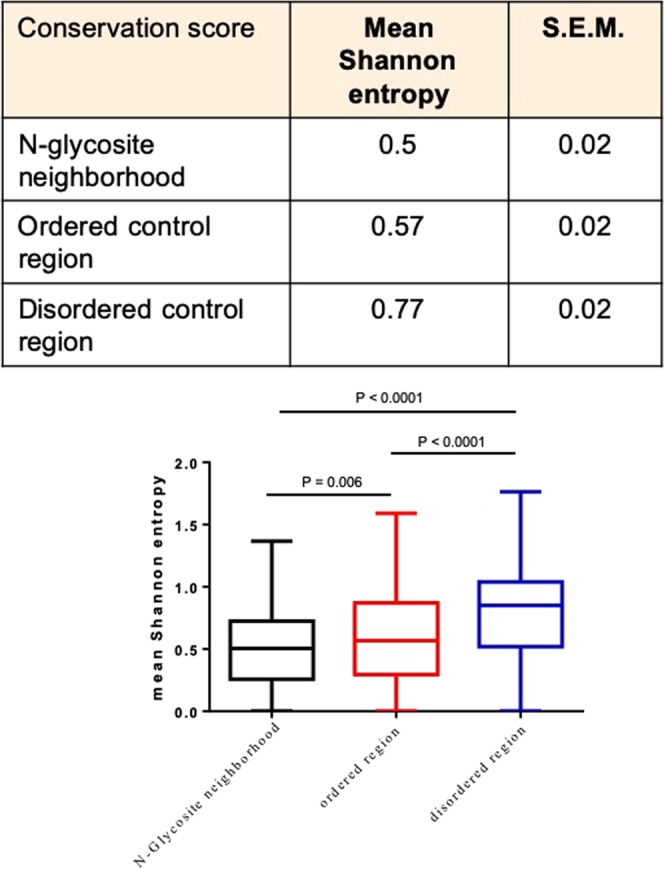
The N-glycosite neighborhoods show greater phylogenetic conservation across amniote genomes than ordered and disordered residue stretches of similar length (Top) Table shows mean Shannon information entropy and its standard error of mean of N-glycosite neighborhoods, 11-residue ordered and disordered regions with N-glycosites. (Bottom) Box and whisker representation showing differences in mean Shannon entropy of N-glycosite neighborhoods, 11-residue ordered and disordered regions with N-glycosites. Multiple sequence alignments for each protein, for which conservation was analyzed is provided in Supplementary File 4. The mean Shannon entropy for each protein is provided in Supplementary File 5. For the graph, boxes represent interquartile range and whiskers extend from minimum to maximum values. Statistical significance is given by P-value measured using Wilcoxon’s matched-pairs signed rank test.

We then asked if N-glycosite neighborhoods represented regions of higher evolutionary conservation within ordered parts of protein primary structures. We aligned the human N-glycoprotein sequences with their orthologs from chimpanzee (*Pan troglodytes*), mouse (*Mus musculus*) and chicken (*Gallus gallus*) in order to assay a possible divergence in the extent of conservation between residues surrounding the N-glycosites (in the ordered regions) and residues in the disordered regions (Supplementary File 6). Our screen sought to probe for conservation within a wider clade (i.e., amniotes), wherein despite important differences in N-linked glycoconjugation, the primary structures of proteins shows high conservation across the vertebrate clade. We quantified conservation by measuring the mean Shannon entropy of residues constituting the N-glycosite neighborhoods and disordered regions (Fig. 6; Supplementary File 7)^46–48^. Shannon entropy is a measure of the information content within a sequence. Its measure across a given residue position within multiple aligned peptide sequences provides insight into how a residue is conserved in that position across species. When 11-residue ordered and disordered regions (with no N-glycosites) were compared, disordered sequences exhibited greater entropy. In contrast, N-glycosite neighborhoods showed lower mean entropy than full-length protein sequences suggesting residues comprising N-glycosite neighborhood show higher, and those comprising disordered regions, lower, evolutionary conservation among amniote N-glycoproteins. Previous studies have shown that the association between residue conservation and residue disorder is context-specific^21,49^. Our observations indicate that N-glycosites represent high-conservation islands within ordered regions which themselves are more conserved within the amniotic clade with respect to disordered regions.

## Discussion

In this paper, we have endeavored to examine the spatial changes in the biochemical properties of amino acids that reside close to or away from the sites of N-glycan conjugation. We observe that residue neighborhoods that immediately flank the glycan conjugated sequons show a similar proportion of charged and polar residues as control ordered regions; such residues are enriched maximally within control disordered regions. On the other hand, N-glycosite neighborhoods are least enriched for hydrophobic residues, more so than even the disordered control regions. Therefore, while both N-glycosites and disordered regions are accessible to the surface, the former represents the ordered tertiary protein surface and the latter represent flexible regions.

We observed that while order-promoting residues are equivalently distributed between N-linked glycosites and control ordered regions, the former are relatively further depleted of disorder-promoting residues compared to the latter. N-glycans have been shown through mounting theoretical^26,50^ and experimental^51^ evidence to impact the folding and structure of glycoproteins (although the relationship is not always positively regulatory)^52,53^. The triose core of N-glycoproteins enhances both the kinetics and stability of tertiary glycoprotein folds^54^. In fact, N-glycosylations seems to destabilize the unfolded state more than stabilize the folded state of proteins^26^. Our results suggest that not just N-glycans but the residue neigborhoods surrounding them may help perform these functions.

Disordered regions are known to contain variable amino acid sequences and we observe that they show relatively lower phylogenetic conservation among amniotes. However, some amino acid residues which have longer side chains than others, e.g., asparagine, proline, glutamate, methionine, serine, arginine, and glutamine are significantly enriched in the disordered regions. The long side-chains of these amino acids may have some significant function in the disordered region which could be related to their ability to enhance flexibility, exposure and disorder. The abundance of pyrrolidine ring-containing proline allows it to introduce kinks or turns in the protein structure and contributes to its propensity within interdomain linkers. It is well known that proline content is generally higher within disordered stretches^22^. Therefore, N-glycosite neighborhoods are uniquely placed in terms of their biochemical properties separating them from the hydrophobic ordered core and the disordered stretches of N-linked glycoproteins.

Based on our observations of evolutionary trace and amniote phylogeny, N-glycosite neighborhoods show residue conservation but do not seem to represent hotspots for the phylogenetic clustering of amino acids that determine function. This however, does not imply that N-linked glycans or even N-glycosites do not determine protein functions. Far from it, both are crucial to fold of the protein and the function performed by it. Additionally, our results imply that disordered stretches may represent hotspots for the further accumulation of function-determining residues, which may regulate the evolvability of such N-glycoproteins.

The N-linked glycosylation of proteins also tends to act as an important quality control check point for the ability of the protein to fold itself correctly^55^. Unfolded proteins are N-glycosylated within the ER lumen and bear 3 Glc residues at the terminal end of their A-branch. N-linked glycans being bulky hydrophilic polymers can increase the thermodynamic stability of proteins, enhance their folding and allow them to evade the enzyme UGGT that would otherwise deploy unfolded proteins for proteolytic degradation^56^. However, this regulation by N-glycans of protein folding does not necessarily bias the hypothetical localization of the N-glycosites within the ordered (or disordered) regions. We would in the future, perform computational studies to examine the effect on the dynamics of disordered regions by the hypothetical presence of one or more N-glycan(s).

Our results are also consistent with an earlier approach that sought to develop algorithms to predict sites of N-linked glycosylation based on structure and pattern^57^, wherein structural properties such as secondary structure were observed to contribute further to the accuracy of prediction of whether a potential N-glycosite is conjugated with a glycan structure, than local contact order. We observed, for example, that residues that are part of N-glycosite neighborhoods show greater propensity to be part of coiled structures. Whereas local contact order may not by itself be able to distinguish successfully between N-glycan-conjugated and -unconjugated glycosites according to Chuang and coworkers^57^, it may contribute to constraining the localization of conjugated N-glycosites within the ordered stretches of the protein.

Our results are specific to N-glycoproteins, which are part of the extracellular milieu. Many of them being transmembrane- and secreted diffusible- proteins, function across multicellular tissue spatial scales and contribute to the developmental mechanisms by mediating discrete biophysical and biochemical functions^3^. The determinacy of their developmental roles is a function of their stability and N-glycosylation may have played an important role in the latter. Our study qualifies this by proposing that the localization of N-linked glycosylations allowed these proteins to also have unstructured interfaces in order that they interact with the various components in the extracellular milieu without compromising the global structured fold of the proteins. Our phylogenetic analysis suggests our N-glycoprotein set consists of proteins with diverse functions. However, it also shows that at least for some of the ontological categories, proteins, which shared some common function also were phylogenetically related. This clustering may help to choose specific representatives of clustered protein sets for further molecular dynamical studies. Such studies will help us better understand the role of disordered regions and N-glycosites in the evolution of protein function at the molecular level.

## Supplementary information

Supplementary Figure File.

Supplementary File 1.

Supplementary File 3.

Supplementary File 6.

Supplementary File 8.

Supplementary File 2.

Supplementary File 4.

Supplementary File 5.

Supplementary File 7.

Supplementary File 9.

## Data Availability

All the raw data for the analyses have been provided in the Supplemenrary Information Files.
