# Author Correction: Mutually exclusive locales for N-linked glycans and disorder in human glycoproteins

**DOI:** 10.1038/s41598-020-68282-x

**Published:** 2020-07-01

**Authors:** Shyamili Goutham, Indu Kumari, Dharma Pally, Alvina Singh, Sujasha Ghosh, Yusuf Akhter, Ramray Bhat

**Affiliations:** 1 Department of Molecular Reproduction, Development and Genetics, Indian Institute of Sciences, Bangalore, 560012 India; 2 School of Earth and Environmental Sciences, Central University of Himachal Pradesh, District-Kangra, Shahpur, Himachal Pradesh 176206 India; 3 Department of Biotechnology, Babasaheb Bhimrao Ambedkar University, Vidya Vihar, Raebareli Road, Lucknow, Uttar Pradesh 226025 India

Correction to: *Scientific Reports* 10.1038/s41598-020-61427-y, published online 08 April 2020

The Acknowledgements section in this Article contains errors.

“RB acknowledges funding from DBT (0525) and Wellcome Trust DBT India Alliance Fellowship (WELT0041). SG (Shyamili Goutham) and DP acknowledge support from IISc for fellowship. We also thank DST-FIST [SR/FST/LS11-036/2014(C)], UGC-SAP [F.4.13/2018/DRS-III (SAP-II)] and DBT-IISc Partnership Program Phase-II (BT/PR27952-INF/22/212/2018) for infrastructure and financial support. Research in YA lab is funded by Indian Council of Medical Research (Ministry of Health & Family Welfare, Government of India) and Department of Biotechnology (Ministry of Science and Technology, Government of India).”

Should read:

“This work was supported by the Wellcome Trust/DBT India Alliance Fellowship/Grant [Grant Number IA/I/17/2/503312] awarded to Ramray Bhat. He also acknowledges support from DBT [BT/PR26526/GET/119/92/2017]. SG (Shyamili Goutham) and DP acknowledge support from IISc for fellowship. We also thank DST-FIST [SR/FST/LS11-036/2014(C)], UGC-SAP [F.4.13/2018/DRS-III (SAP-II)] and DBT-IISc Partnership Program Phase-II (BT/PR27952-INF/22/212/2018) for infrastructure and financial support. Research in YA lab is funded by Indian Council of Medical Research (Ministry of Health & Family Welfare, Government of India) and Department of Biotechnology (Ministry of Science and Technology, Government of India).”

