# Supplementary material for: Mutually exclusive locales for N-linked glycans and disorder in human glycoproteins
Source: Sci Rep. 2020 Apr 8;10:6040. doi: 10.1038/s41598-020-61427-y (PMC7142085; doi:10.1038/s41598-020-61427-y)
Supplement: Supplementary file 1 — Supplementary Figure File. [file 41598_2020_61427_MOESM1_ESM.pdf]

Mutually exclusive locales for N-linked glycans and disorder in human glycoproteins

Shyamili Goutham[1], Indu Kumari[2], Dharma Pally[1], Alvina SIngh[1], Sujasha Ghosh[1], Yusuf Akhter[3] and Ramray Bhat[1]

1. Department of Molecular Reproduction, Development and Genetics, Indian Institute of Sciences, Bangalore 560012, India
2. School of Earth and Environmental Sciences, Central University of Himachal Pradesh, District-Kangra, Shahpur, Himachal Pradesh 176206, India.
3. Department of Biotechnology, Babasaheb Bhimrao Ambedkar University, Vidya Vihar, Raebareli Road, Lucknow, Uttar Pradesh 226025, India.

Running Title: N-glycans and protein disorder

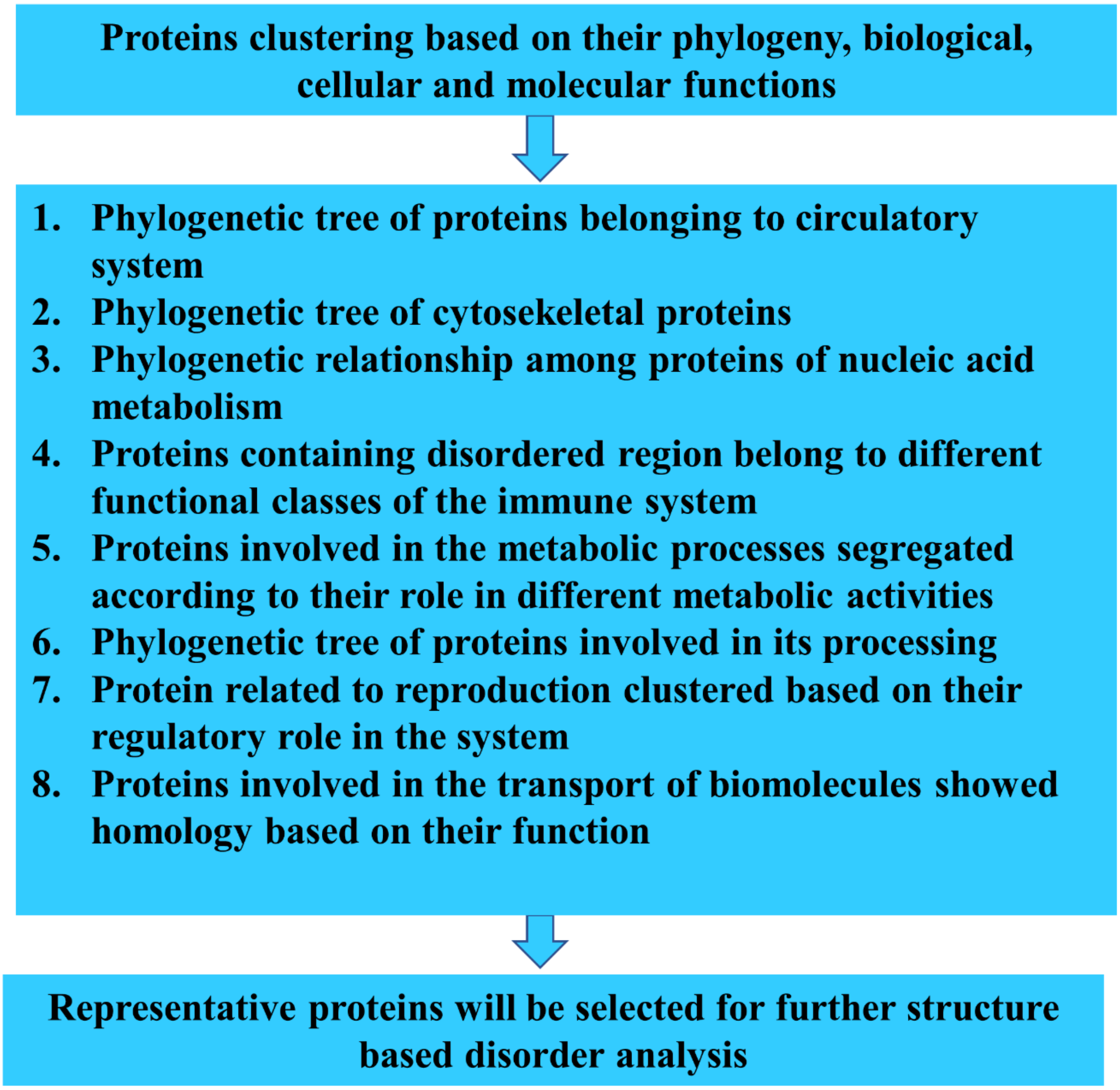

Supplementary Figure 1: Flow chart showing the work flow for the classification of proteins based on their biological functions, which was the basis for the phylogenetic analysis of the proteins.

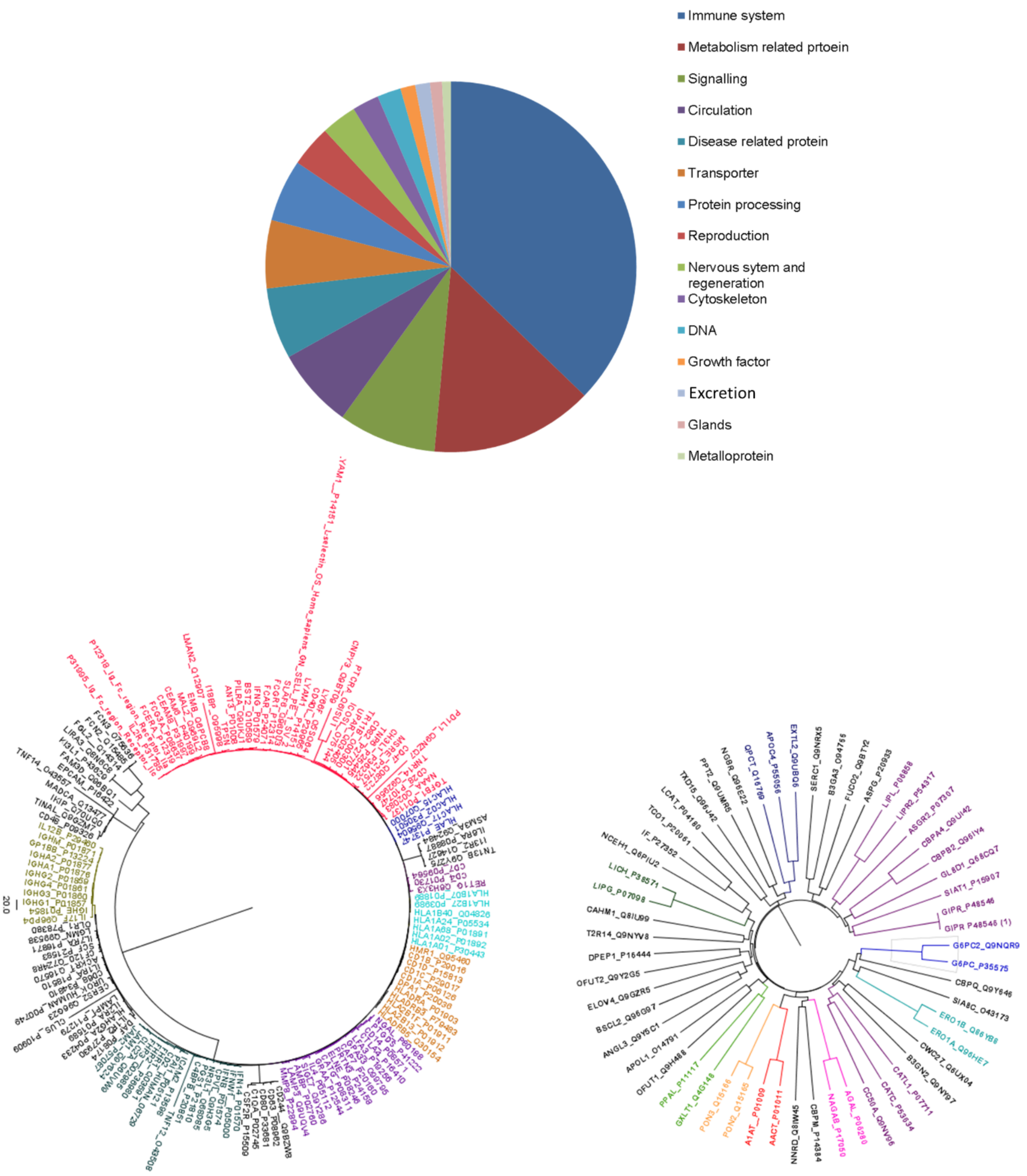

Supplementary Figure 2: Figure 2: Classification of, and evolutionary relationship between, human N-linked glycoproteins based on their biological function (Top) Pie chart represents the classification of human N-linked glycoproteins based on their biological functions. Most of the proteins selected belong to the immune system followed by proteins related to metabolic processes. A small number of proteins are found in excretion, glandular functions or are growth factors and metalloproteins. (Bottom left) N-linked glycoproteins containing disordered regions belong to different functional classes of the immune system, which have some conserved structure: It was observed that the proteins get clustered into

different groups depending on their function or their interaction with the antigen/ antigen processing step. The proteins showed in red colour encode those related to Immunoglobulins, antigen-related cell adhesion molecules, selectin protein, purple colour encode HLA class I histocompatibility antigen for alpha chain and different proteins, which involve glycoprotein interactions like CTLA, Neutrophil gelatinase-associated lipocalin (NGAL), cyan blue colour encode HLA class I histocompatibility antigen a or C class for alpha chain, wheat brown colour encode for Antigen-presenting glycoprotein CD proteins and some HLA class I histocompatibility antigen DR beta chain, black colour encode proteins related to CD like CD63, CD244 Natural killer cell receptor, CD80 T-lymphocyte activation antigen, CSF2R Granulocyte-macrophage colony-stimulating factor receptor which exhibit different functions, dark blue colour encode for different proteins ranging from interferon to proteins involved innaive B-cell development, complement factor related protein, interleukins and Lysosome-associated membrane glycoprotein, green colour encode Immunoglobulin heavy constant gamma protein while IL17 and IL2 also showed similarity to these proteins and some miscellaneous proteins formed a separate showed in black colour are Ficolin-3, Killer cell immunoglobulin-like receptor, Leukocyte immunoglobulin-like receptor, Mucosal addressin cell adhesion molecule, Epithelial cell adhesion moleculeTubulointerstitial nephritis antigen-like and Inhibitor of nuclear factor kappa-B kinase-interacting protein. Most of the proteins taken for the study belong to the immune system and formed groups according to their function. (Bottom right) Proteins involved in the metabolic processes segregate according to their role in different metabolic activities, whch have different structural and functional domains: The proteins involved in the removal of phosphate from glucose formed one group shown in blue colour. ER protein represented in cyan blue colour and proteins involved in galactose processing were shown in magenta colour. Serum paraoxonase/arylesteraseproteins formed a cluster shown in yellow colour. Antitrypsin and antichymotrysin formed cluster presented in red colour. Glucoside xylosyltransferase and Lysosomal acid phosphatase showed similarity and presented in green colour. Protein related to protein digestion, fat metabolism and gastric digestion regulation formed a cluster shown in purple colour. Apolipoprotein C-IV, Glutaminyl-peptide cyclotransferase and Exostosin-like 2 formed a cluster which is shown in dark blue colour. Rest of the proteins represented in black colour belong to different categories like Dipeptidase, Gastric intrinsic factor, Plasma alpha-L-fucosidase, Thioredoxin domain-containing protein, Lysosomal thioesterase, Calcium homeostasis modulator protein etc. did not form a separate cluster as they have different function and there is not much similarity between their sequences.

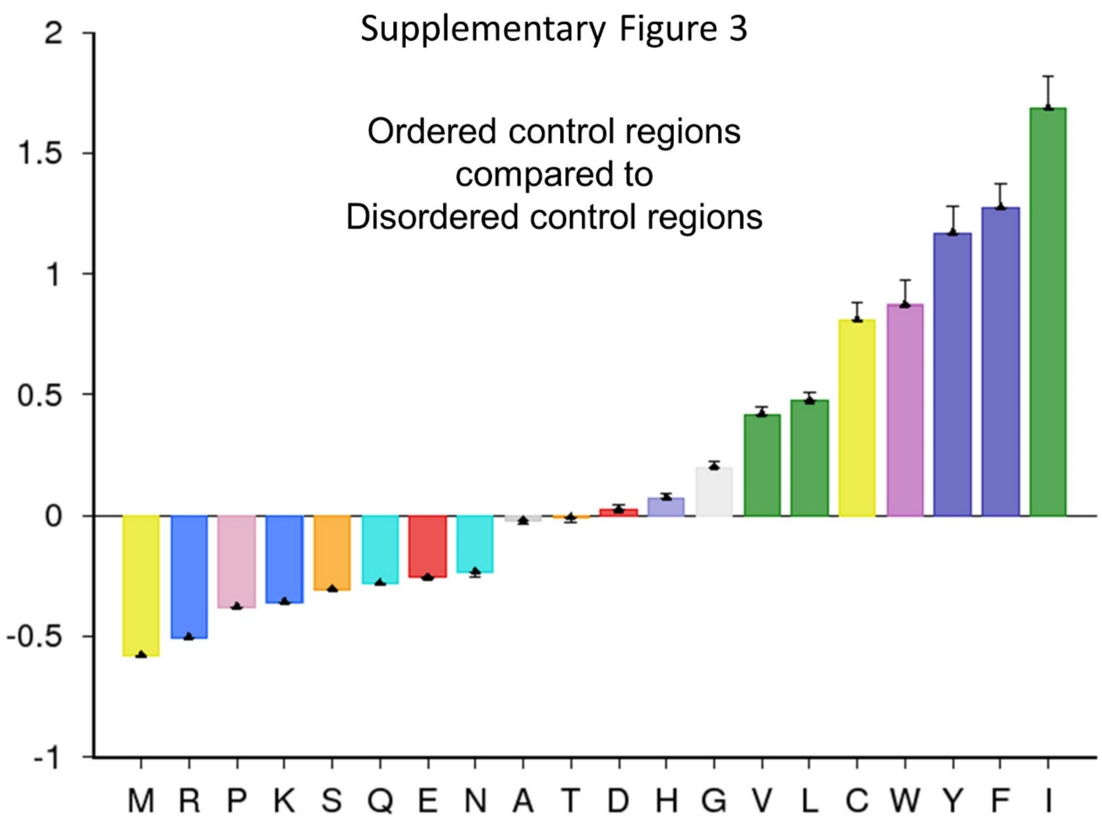

M	Depleted.	P-value=0.000000 (≤0.050000)
R	Depleted.	P-value=0.000000 (≤0.050000)
P	Depleted.	P-value=0.000000 (≤0.050000)
K	Depleted.	P-value=0.000003 (≤0.050000)
S	Depleted.	P-value=0.000000 (≤0.050000)
Q	Depleted.	P-value=0.002536 (≤0.050000)
E	Depleted.	P-value=0.000917 (≤0.050000)
N	Depleted.	P-value=0.028076 (≤0.050000)
A	Not significant.	P-value=0.770076 (>0.050000)
T	Not significant.	P-value=0.920121 (>0.050000)
D	Not significant.	P-value=0.838429 (>0.050000)
H	Not significant.	P-value=0.623179 (>0.050000)
G	Enriched.	P-value=0.026904 (≤0.050000)
V	Enriched.	P-value=0.000043 (≤0.050000)
L	Enriched.	P-value=0.000000 (≤0.050000)
C	Enriched.	P-value=0.000022 (≤0.050000)
W	Enriched.	P-value=0.000299 (≤0.050000)
Y	Enriched.	P-value=0.000000 (≤0.050000)
F	Enriched.	P-value=0.000000 (≤0.050000)
I	Enriched.	P-value=0.000000 (≤0.050000)

Supplementary Figure 3: (Top) Graphical representation of fractional enrichment of amino acids in 11-residue ordered control regions normalized to disordered control regions of similar length (both containing no N-glycosites). (Bottom) Tabular representation signifying enrichment and depletion of individual amino acids for the above comparison with significance.

Supplementary Figure 4

Ordered control regions
compared to
disordered control regions

Aromatic content	Enriched.	P-value=0.000000 (≤0.050000)
Charged residues	Depleted.	P-value=0.000000 (≤0.050000)
Positively charged	Depleted.	P-value=0.000000 (≤0.050000)
Negatively charged	Depleted.	P-value=0.014853 (≤0.050000)
Polar	Depleted.	P-value=0.000000 (≤0.050000)
Hydrophobic	Enriched.	P-value=0.000000 (≤0.050000)
Hydrophobic	Enriched.	P-value=0.000000 (≤0.050000)
Hydrophobic	Enriched.	P-value=0.000000 (≤0.050000)
Exposed	Depleted.	P-value=0.000000 (≤0.050000)
Flexible	Depleted.	P-value=0.000000 (≤0.050000)
High interface prop.	Enriched.	P-value=0.000000 (≤0.050000)
High solvation poten.	Depleted.	P-value=0.000000 (≤0.050000)
Frequent in alpha hel.	Not significant.	P-value=0.624573 (>0.050000)
Frequent in beta struc.	Enriched.	P-value=0.000000 (≤0.050000)
Frequent in coils	Depleted.	P-value=0.000000 (≤0.050000)
High linker propensity	Depleted.	P-value=0.001900 (≤0.050000)
Disorder promoting	Depleted.	P-value=0.000000 (≤0.050000)
Order promoting	Enriched.	P-value=0.000000 (≤0.050000)
Bulky	Enriched.	P-value=0.000000 (≤0.050000)
[illegible]	[illegible]	[illegible]

Supplementary Figure 4: Tabular representation signifying enrichment and depletion of residue properties based on the above amino acid enrichment and depletion profile. Error bars represent SD. Statistical significance is given by P value measured using two-way t-test (see [33]).

Supplementary Figure 5

N-glycosite neighborhoods
compared to
disordered control regions

Ala	Depleted.	P-value=0.000000 (≤0.050000)
Arg	Depleted.	P-value=0.000000 (≤0.050000)
Asn	Enriched.	P-value=0.000000 (≤0.050000)
Asp	Not signific ant.	P-value=0.463324 (>0.050000)
Cys	Enriched.	P-value=0.021742 (≤0.050000)
Gln	Depleted.	P-value=0.020156 (≤0.050000)
Glu	Depleted.	P-value=0.004992 (≤0.050000)
Gly	Not significant.	P-value=0.186428 (>0.050000)
His	Not significant.	P-value=0.772704 (>0.050000)
Ile	Enriched.	P-value=0.000000 (≤0.050000)
Leu	Depleted.	P-value=0.005205 (≤0.050000)
Lys	Depleted.	P-value=0.000000 (≤0.050000)
Met	Depleted.	P-value=0.000000 (≤0.050000)
Phe	Enriched.	P-value=0.000000 (≤0.050000)
Pro	Depleted.	P-value=0.000000 (≤0.050000)
Ser	Not significant.	P-value=0.909688 (>0.050000)
Thr	Enriched.	P-value=0.000000 (≤0.050000)
Trp	Enriched.	P-value=0.004635 (≤0.050000)
Tyr	Enriched.	P-value=0.000000 (≤0.050000)
Val	Enriched.	P-value=0.008372 (≤0.050000)

Supplementary Figure 5: Table showing statistical significance for the fractional enrichment and depletion of individual amino acids cognate to N-glycosite neighborhoods normalized with disordered control regions (See Figure 3).

Supplementary Figure 6

N-glycosite neighborhoods
compared to
ordered control regions

Ala	Depleted.	P-value=0.000000 (≤0.050000)
Arg	Not significant.	P-value=0.732934 (>0.050000)
Asn	Enriched.	P-value=0.000000 (≤0.050000)
Asp	Not significant.	P-value=0.267047 (>0.050000)
Cys	Depleted.	P-value=0.004101 (≤0.050000)
Gln	Not significant.	P-value=0.263270 (>0.050000)
Glu	Not significant.	P-value=0.344542 (>0.050000)
Gly	Depleted.	P-value=0.000010 (≤0.050000)
His	Not significant.	P-value=0.773717 (>0.050000)
Ile	Depleted.	P-value=0.028910 (≤0.050000)
Leu	Depleted.	P-value=0.000000 (≤0.050000)
Lys	Not significant.	P-value=0.175576 (>0.050000)
Met	Depleted.	P-value=0.000094 (≤0.050000)
Phe	Not significant.	P-value=0.219350 (>0.050000)
Pro	Depleted.	P-value=0.000008 (≤0.050000)
Ser	Enriched.	P-value=0.000000 (≤0.050000)
Thr	Enriched.	P-value=0.000000 (≤0.050000)
Trp	Not significant.	P-value=0.190187 (>0.050000)
Tyr	Not significant.	P-value=0.103530 (>0.050000)
Val	Depleted.	P-value=0.030419 (≤0.050000)

Supplementary Figure 6: Tables showing statistical significance for the fractional enrichment and depletion of individual amino acids cognate to N-glycosite neighborhoods normalized with ordered control regions (see Figure 4).

Supplementary Figure 7

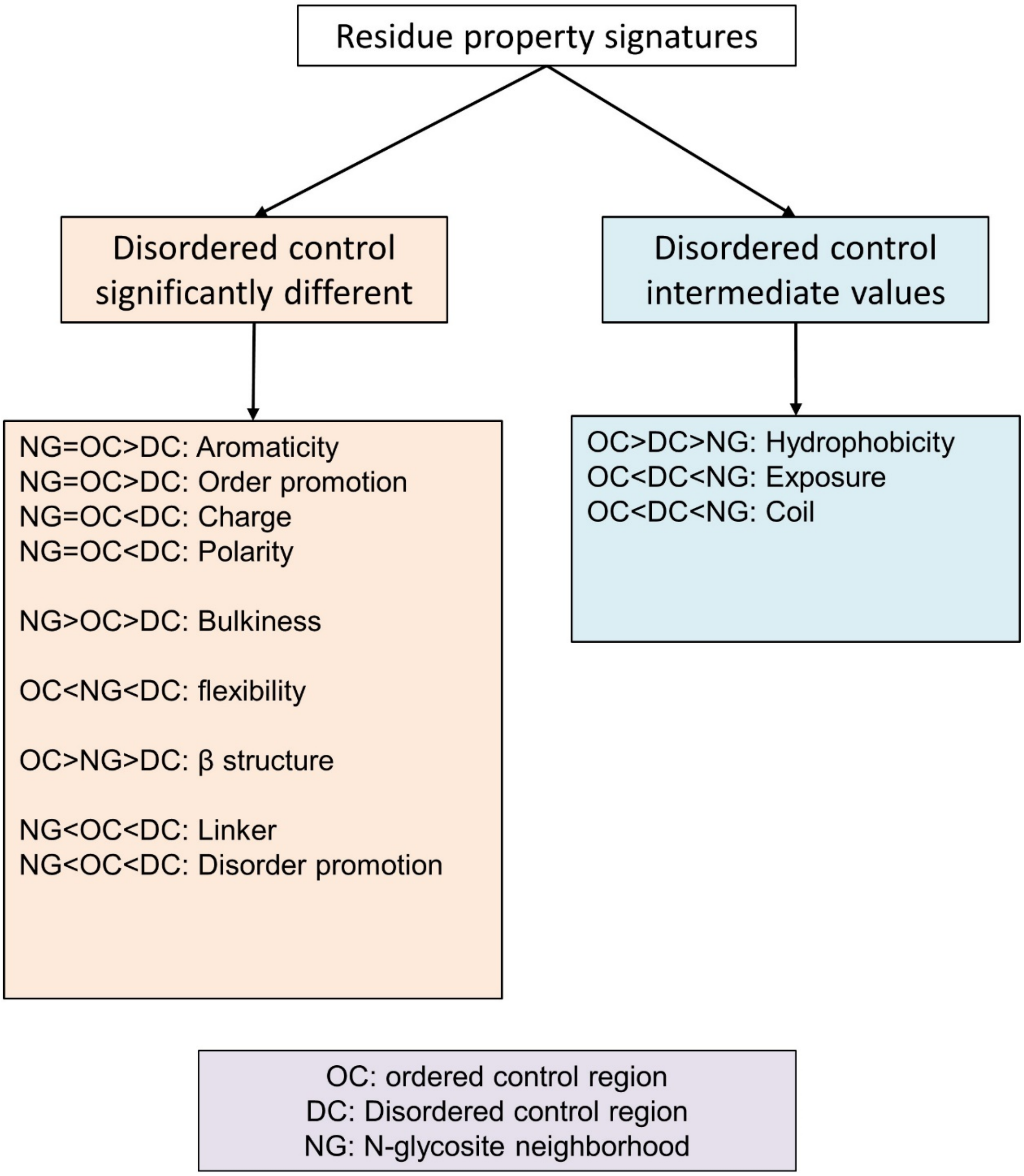

Supplementary Figure 7: Schematic showing patterns of the relationship of primary structural properties between N-glycosite neighborhoods, ordered- and disordered- control regions.

Supplementary File1: Sequences of 500 human N-glycoproteins with annotations for N-glycosites (blue highlight) and disordered residues (yellow highlight).

Supplementary File 2: List of the proportion of Asn, Ser, Thr and sequon signatures in ordered and disordered regions of human N-glycoproteins.

Supplementary File 3: Sequences of 18 human N-glycoproteins from Disprot database with annotations for N-glycosites (green font) and disordered residues (yellow highlight).

Supplementary File 4: Gene ontological categories with their proportional representation within the sequence set used for this study (n=500), the total human N-glycoprotein (with disordered regions) sequence set (n=1124) and a subset of the 500-protein set that consist of N-glycosites within disordered regions.

Supplementary File 5: Mean Universal evolutionary trace ranks for N-glycosite neighborhoods, control disordered regions and control ordered regions within human N-glycproteins.

Supplementary File 6: Multiple sequence alignments of human N-glycoproteins and their orthologs from chimpanzee, mouse and chicken with annotations for disorder (yellow highlight) and N-glycosite neighborhoods (blue highlight).

Supplementary File 7: Mean Shannon entropy scores for N-glycosite neighborhoods, control disordered regions and control ordered regions within human N-glycoproteins.

Supplementary File 8: Amino acid enrichment and depletion in N-glycosite neighborhoods with ordered regions compared with N-glycosite neighborhoods within disordered regions.

Supplementary File 9: Proportion of specific amino acids occupying ‘X’ in the NXS/T/C sequons found in ordered and disordered regions of human N-glycoproteins.
