# Supplementary material for: Mutually exclusive locales for N-linked glycans and disorder in human glycoproteins
Source: Sci Rep. 2020 Apr 8;10:6040. doi: 10.1038/s41598-020-61427-y (PMC7142085; doi:10.1038/s41598-020-61427-y)
Supplement: Supplementary file 2 — Supplementary File 1. [file 41598_2020_61427_MOESM2_ESM.docx]

1.>sp|P31358|CD52_HUMANCAMPATH-1antigenOS=HomosapiensGN=CD52PE=1SV=1

MKRFLFLLLTISLLVMVQIQTGLSGQNDTSQTSSPSASSNISGGIFLFFVANAIIHLFCFS

(2^nd^ N is in O.R)

2.>sp|P01596|KV104_HUMANIgkappachainV-IregionCAROS=HomosapiensPE=1SV=1

DIQMTQSPSTLSASVGDRVAITCRASQNISSWLAWYQQKPGKAPKVLIYKSSSLESGVPSRFSGSGSGTDFTLTISSLZPBBFATYYCQQYNTFFTFGPGTKVDIKR

3.>sp|P06888|LV109_HUMANIglambdachainV-IregionEPSOS=HomosapiensPE=1SV=1

QSVLTQPPSLSAAPGQRVSISCSGSSSNIGKNYVDWYQQLPGTAPKLLIFNNNKRPSGIPDRFSGSKSGTSATLGITGLQTGDEAIYYCGTWDNRRSVFGGGTNVTVVG

4.>sp|P01705|LV202_HUMANIglambdachainV-IIregionNEIOS=HomosapiensPE=1SV=1

QSALTQPASVSGSPGQSITISCTGTTSDVGSYNFVSWYQQNPGKAPKLMIYEGNKRPSGVSNRFSGSKSGKTASLTISGLQVEDEADYYCCSYAGNSTRVFGGGTRVTVLS

5.>sp|P01763|HV302_HUMANIgheavychainV-IIIregionWEAOS=HomosapiensPE=1SV=1

QVQLVDSGGGLVEPGGSLRLSCSASGFTFSANDMNWVRQAPGKGLEWLSFIGGSGSTIYYADSVKGRFTISRNBSKNSLYLQMSSLRAEDTAVYYCARGWLLNWGQGTLVTVSS

6.>sp|P06315|KV501_HUMANIgkappachainVregionEV15OS=HomosapiensPE=1SV=1

MGSQVHLLSFLLLWISDTRAETTLTQSPAFMSATPGDKVNISCKASQDIDDDMNWYQQKPGEAAIFIIQEATTLVPGIPPRFSGSGYGTDFTLTINNIESEDAAYYFCLQHDNFP

7.>sp|P01215|GLHA_HUMANGlycoproteinhormonesalphachainOS=HomosapiensGN=CGAPE=1SV=1

MDYYRKYAAIFLVTLSVFLHVLHSAPDVQDCPECTLQENPFFSQPGAPILQCMGCCFSRAYPTPLRSKKTMLVQKNVTSESTCCVAKSYNRVTVMGGFKVENHTACHCSTCYYHKS

8.>sp|P61769|B2MG_HUMANBeta-2-microglobulinOS=HomosapiensGN=B2MPE=1SV=1

MSRSVALAVLALLSLSGLEAIQRTPKIQVYSRHPAENGKSNFLNCYVSGFHPSDIEVDLLKNGERIEKVEHSDLSFSKDWSFYLLYYTEFTPTEKDEYACRVNHVTLSQPKIVKWDRDM

9.>sp|O00175|CCL24_HUMANC-Cmotifchemokine24OS=HomosapiensGN=CCL24PE=1SV=2

MAGLMTIVTSLLFLGVCAHHIIPTGSVVIPSPCCMFFVSKRIPENRVVSYQLSSRSTCLKAGVIFTTKKGQQFCGDPKQEWVQRYMKNLDAKQKKASPRARAVAVKGPVQRYPGNQTTC

10.>sp|P01773|HV312_HUMANIgheavychainV-IIIregionBUROS=HomosapiensPE=1SV=1

QVQLVESGGGVVQAGTSLRLSCTASAFNLSDYAMHWVRQAPGKGLZWVALISYGGSBTYYADSVRGRFTISRBISKBTLYLZMKTLRTEDTAVYYCAKLIAVAGTRBFWGQGTLVTVSL

11.>sp|Q9BUF7|CRUM3_HUMANProteincrumbshomolog3OS=HomosapiensGN=CRB3PE=1SV=3

MANPGLGLLLALGLPFLLARWGRAWGQIQTTSANENSTVLPSSTSSSSDGNLRPEAITAIIVVFSLLAALLLAVGLALLVRKLREKRQTEGTYRPSSEEQVGARVPPTPNLKLPPEERLI

12.>sp|P01815|HV202_HUMANIgheavychainV-IIregionCOROS=HomosapiensPE=1SV=1

QVTLRESGPALVKPTQTLTLTCTFSGFSLSSTGMCVGWIRQPPGKGLEWLARIDWDDDKYYNTSLETRLTISKDTSRNQVVLTMDPVDTATYYCARITVIPAPAGYMDVWGRGTPVTVSS

13.>sp|Q14508|WFDC2_HUMANWAPfour-disulfidecoredomainprotein2OS=HomosapiensGN=WFDC2PE=1SV=2

MPACRLGPLAAALLLSLLLFGFTLVSGTGAEKTGVCPELQADQNCTQECVSDSECADNLKCCSAGCATFCSLPNDKEGSCPQVNINFPQLGLCRDQCQVDSQCPGQMKCCRNGCGKVSCVTPNF

14.>sp|P55056|APOC4_HUMANApolipoproteinC-IVOS=HomosapiensGN=APOC4PE=1SV=1

MSLLRNRLQALPALCLCVLVLACIGACQPEAQEGTLSPPPKLKMSRWSLVRGRMKELLETVVNRTRDGWQWFWSPSTFRGFMQTYYDDHLRDLGPLTKAWFLESKDSLLKKTHSLCPRLVCGDKDQG

15.>sp|P13987|CD59_HUMANCD59glycoproteinOS=HomosapiensGN=CD59PE=1SV=1

MGIQGGSVLFGLLLVLAVFCHSGHSLQCYNCPNPTADCKTAVNCSSDFDACLITKAGLQVYNKCWKFEHCNFNDVTTRLRENELTYYCCKKDLCNFNEQLENGGTSLSEKTVLLLVTPFLAAAWSLHP

16.>sp|P04921|GLPC_HUMANGlycophorin-COS=HomosapiensGN=GYPCPE=1SV=1

MWSTRSPNSTAWPLSLEPDPGMASASTTMHTTTIAEPDPGMSGWPDGRMETSTPTIMDIVVIAGVIAAVAIVLVSLLFVMLRYMYRHKGTYHTNEAKGTEFAESADAALQGDPALQDAGDSSRKEYFI

17.>sp|P01225|FSHB_HUMANFollitropinsubunitbetaOS=HomosapiensGN=FSHBPE=1SV=2

MKTLQFFFLFCCWKAICCNSCELTNITIAIEKEECRFCISINTTWCAGYCYTRDLVYKDPARPKIQKTCTFKELVYETVRVPGCAHHADSLYTYPVATQCHCGKCDSDSTDCTVRGLGPSYCSFGEMKE

18.>sp|P15382|KCNE1_HUMANPotassiumvoltage-gatedchannelsubfamilyEmember1OS=HomosapiensGN=KCNE1PE=1SV=1

MILSNTTAVTPFLTKLWQETVQQGGNMSGLARRSPRSSDGKLEALYVLMVLGFFGFFTLGIMLSYIRSKKLEHSNDPFNVYIESDAWQEKDKAYVQARVLESYRSCYVVENHLAIEQPNTHLPETKPSP

(1^st^N in D.R)

19.>sp|P35542|SAA4_HUMANSerumamyloidA-4proteinOS=HomosapiensGN=SAA4PE=1SV=2

MRLFTGIVFCSLVMGVTSESWRSFFKEALQGVGDMGRAYWDIMISNHQNSNRYLYARGNYDAAQRGPGGVWAAKLISRSRVYLQGLIDCYLFGNSSTVLEDSKSNEKAEEWGRSGKDPDRFRPDGLPKKY

20.>sp|P01733|TVB1_HUMANT-cellreceptorbetachainVregionYT35OS=HomosapiensGN=TRBV12-3PE=1SV=1

MDSWTFCCVSLCILVAKHTDAGVIQSPRHEVTEMGQEVTLRCKPISGHNSLFWYRQTMMR

GLELLIYFNNNVPIDDSGMPEDRFSAKMPNASFSTLKIQPSEPRDSAVYFCASSFSTCSA

NYGYTFGSGTRLTVV

21.>sp|P01737|TVA3_HUMANT-cellreceptoralphachainVregionPY14OS=HomosapiensPE=1SV=1

MLLLLVPVLEVIFTLGGTRAQSVTQLGSHVSVSEGALVLLRCNYSSSVPPYLFWYVQYPN

QGLQLLLKYTSAATLVKGINGFEAEFKKSETSFHLTKPSAHMSDAAEYFCAVSDLEPNSS

ASKIIFGSGTRLSIR

22.>sp|Q9GZZ8|LACRT_HUMANExtracellularglycoproteinlacritinOS=HomosapiensGN=LACRTPE=1SV=1

MKFTTLLFLAAVAGALVYAEDASSDSTGADPAQEAGTSKPNEEISGPAEPASPPETTTTA

QETSAAAVQGTAKVTSSRQELNPLKSIVEKSILLTEQALAKAGKGMHGGVPGGKQFIENG

SEFAQKLLKKFSLLKPWA

23.>sp|P01222|TSHB_HUMANThyrotropinsubunitbetaOS=HomosapiensGN=TSHBPE=1SV=2

MTALFLMSMLFGLTCGQAMSFCIPTEYTMHIERRECAYCLTINTTICAGYCMTRDINGKL

FLPKYALSQDVCTYRDFIYRTVEIPGCPLHVAPYFSYPVALSCKCGKCNTDYSDCIHEAI

KTNYCTKPQKSYLVGFSV

24.>sp|P01229|LSHB_HUMANLutropinsubunitbetaOS=HomosapiensGN=LHBPE=1SV=3

MEMLQGLLLLLLLSMGGAWASREPLRPWCHPINAILAVEKEGCPVCITVNTTICAGYCPT

MMRVLQAVLPPLPQVVCTYRDVRFESIRLPGCPRGVDPVVSFPVALSCRCGPCRRSTSDC

GGPKDHPLTCDHPQLSGLLFL

25.>sp|O60676|CST8_HUMANCystatin-8OS=HomosapiensGN=CST8PE=1SV=1

MPRCRWLSLILLTIPLALVARKDPKKNETGVLRKLKPVNASNANVKQCLWFAMQEYNKES

EDKYVFLVVKTLQAQLQVTNLLEYLIDVEIARSDCRKPLSTNEICAIQENSKLKRKLSCS

FLVGALPWNGEFTVMEKKCEDA

26.>sp|P69905|HBA_HUMANHemoglobinsubunitalphaOS=HomosapiensGN=HBA1PE=1SV=2

(No N-glycosylation present)

MVLSPADKTNVKAAWGKVGAHAGEYGAEALERMFLSFPTTKTYFPHFDLSHGSAQVKGHG

KKVADALTNAVAHVDDMPNALSALSDLHAHKLRVDPVNFKLLSHCLLVTLAAHLPAEFTP

AVHASLDKFLASVSTVLTSKYR

27.>sp|P00709|LALBA_HUMANAlpha-lactalbuminOS=HomosapiensGN=LALBAPE=1SV=1

MRFFVPLFLVGILFPAILAKQFTKCELSQLLKDIDGYGGIALPELICTMFHTSGYDTQAI

VENNESTEYGLFQISNKLWCKSSQVPQSRNICDISCDKFLDDDITDDIMCAKKILDIKGI

DYWLAHKALCTEKLEQWLCEKL

28.>sp|P01848|TCA_HUMANT-cellreceptoralphachainCregionOS=HomosapiensGN=TRACPE=1SV=1

PNIQNPDPAVYQLRDSKSSDKSVCLFTDFDSQTNVSQSKDSDVYITDKTVLDMRSMDFKS

NSAVAWSNKSDFACANAFNNSIIPEDTFFPSPESSCDVKLVEKSFETDTNLNFQNLSVIG

FRILLLKVAGFNLLMTLRLWSS

29.>sp|P04141|CSF2_HUMANGranulocyte-macrophagecolony-stimulatingfactorOS=HomosapiensGN=CSF2PE=1SV=1

MWLQSLLLLGTVACSISAPARSPSPSTQPWEHVNAIQEARRLLNLSRDTAAEMNETVEVI

SEMFDLQEPTCLQTRLELYKQGLRGSLTKLKGPLTMMASHYKQHCPPTPETSCATQIITF

ESFKENLKDFLLVIPFDCWEPVQE

30.>sp|O76096|CYTF_HUMANCystatin-FOS=HomosapiensGN=CST7PE=1SV=1

MRAAGTLLAFCCLVLSTTGGPSPDTCSQDLNSRVKPGFPKTIKTNDPGVLQAARYSVEKF

NNCTNDMFLFKESRITRALVQIVKGLKYMLEVEIGRTTCKKNQHLRLDDCDFQTNHTLKQ

TLSCYSEVWVVPWLQHFEVPVLRCH

31.>sp|P12273|PIP_HUMANProlactin-inducibleproteinOS=HomosapiensGN=PIPPE=1SV=1

MRLLQLLFRASPATLLLVLCLQLGANKAQDNTRKIIIKNFDIPKSVRPNDEVTAVLAVQT

ELKECMVVKTYLISSIPLQGAFNYKYTACLCDDNPKTFYWDFYTNRTVQIAAVVDVIREL

GICPDDAAVIPIKNNRFYTIEILKVE

32.>sp|P68871|HBB_HUMANHemoglobinsubunitbetaOS=HomosapiensGN=HBBPE=1SV=2

(No N-glycosylation present)

MVHLTPEEKSAVTALWGKVNVDEVGGEALGRLLVVYPWTQRFFESFGDLSTPDAVMGNPK

VKAHGKKVLGAFSDGLAHLDNLKGTFATLSELHCDKLHVDPENFRLLGNVLVCVLAHHFG

KEFTPPVQAAYQKVVAGVANALAHKYH

33.>sp|P02766|TTHY_HUMANTransthyretinOS=HomosapiensGN=TTRPE=1SV=1

MASHRLLLLCLAGLVFVSEAGPTGTGESKCPLMVKVLDAVRGSPAINVAVHVFRKAADDT

WEPFASGKTSESGELHGLTTEEEFVEGIYKVEIDTKSYWKALGISPFHEHAEVVFTANDS

GPRRYTIAALLSPYSYSTTAVVTNPKE

34.>sp|P02724|GLPA_HUMANGlycophorin-AOS=HomosapiensGN=GYPAPE=1SV=2

MYGKIIFVLLLSEIVSISASSTTGVAMHTSTSSSVTKSYISSQTNDTHKRDTYAATPRAH

EVSEISVRTVYPPEEETGERVQLAHHFSEPEITLIIFGVMAGVIGTILLISYGIRRLIKK

SPSDVKPLPSPDTDVPLSSVEIENPETSDQ

35.>sp|P61916|NPC2_HUMANEpididymalsecretoryproteinE1OS=HomosapiensGN=NPC2PE=1SV=1

MRFLAATFLLLALSTAAQAEPVQFKDCGSVDGVIKEVNVSPCPTQPCQLSKGQSYSVNVT

FTSNIQSKSSKAVVHGILMGVPVPFPIPEPDGCKSGINCPIQKDKTYSYLNKLPVKSEYP

SIKLVVEWQLQDDKNQSLFCWEIPVQIVSHL

36.>sp|P05112|IL4_HUMANInterleukin-4OS=HomosapiensGN=IL4PE=1SV=1

MGLTSQLLPPLFFLLACAGNFVHGHKCDITLQEIIKTLNSLTEQKTLCTELTVTDIFAAS

KNTTEKETFCRAATVLRQFYSHHEKDTRCLGATAQQFHRHKQLIRFLKRLDRNLWGLAGL

NSCPVKEANQSTLENFLERLKTIMREKYSKCSS

37.>sp|Q86XR5|PRIMA_HUMANProline-richmembraneanchor1OS=HomosapiensGN=PRIMA1PE=1SV=2

MLLRDLVLRRGCCWSSLLLHCALHPLWGFVQVTHGEPQKSCSKVTDSCRHVCQCRPPPPL

PPPPPPPPPPRLLSAPAPNSTSCPTEESWWSGLVIIIAVCCASLVFLTVLVIICYKAIKR

KPLRKDENGTSVAEYPMSASQSNKGVDVNNAVV

38.>sp|P07998|RNAS1_HUMANRibonucleasepancreaticOS=HomosapiensGN=RNASE1PE=1SV=4

MALEKSLVRLLLLVLILLVLGWVQPSLGKESRAKKFQRQHMDSDSSPSSSSTYCNQMMRR

RNMTQGRCKPVNTFVHEPLVDVQNVCFQEKVTCKNGQGNCYKSNSSMHITDCRLTNGSRY

PNCAYRTSPKERHIIVACEGSPYVPVHFDASVEDST

39.>sp|P01591|IGJ_HUMANImmunoglobulinJchainOS=HomosapiensGN=JCHAINPE=1SV=4

MKNHLLFWGVLAVFIKAVHVKAQEDERIVLVDNKCKCARITSRIIRSSEDPNEDIVERNI

RIIVPLNNRENISDPTSPLRTRFVYHLSDLCKKCDPTEVELDNQIVTATQSNICDEDSAT

ETCYTYDRNKCYTAVVPLVYGGETKMVETALTPDACYPD

40.>sp|Q9Y6Y9|LY96_HUMANLymphocyteantigen96OS=HomosapiensGN=LY96PE=1SV=2

MLPFLFFSTLFSSIFTEAQKQYWVCNSSDASISYTYCDKMQYPISINVNPCIELKRSKGL

LHIFYIPRRDLKQLYFNLYITVNTMNLPKRKEVICRGSDDDYSFCRALKGETVNTTISFS

FKGIKFSKGKYKCVVEAISGSPEEMLFCLEFVILHQPNSN

41.>sp|P10153|RNAS2_HUMANNon-secretoryribonucleaseOS=HomosapiensGN=RNASE2PE=1SV=2

MVPKLFTSQICLLLLLGLLAVEGSLHVKPPQFTWAQWFETQHINMTSQQCTNAMQVINNY

QRRCKNQNTFLLTTFANVVNVCGNPNMTCPSNKTRKNCHHSGSQVPLIHCNLTTPSPQNI

SNCRYAQTPANMFYIVACDNRDQRRDPPQYPVVPVHLDRII

42.>sp|P04216|THY1_HUMANThy-1membraneglycoproteinOS=HomosapiensGN=THY1PE=1SV=2

MNLAISIALLLTVLQVSRGQKVTSLTACLVDQSLRLDCRHENTSSSPIQYEFSLTRETKK

HVLFGTVGVPEHTYRSRTNFTSKYNMKVLYLSAFTSKDEGTYTCALHHSGHSPPISSQNV

TVLRDKLVKCEGISLLAQNTSWLLLLLLSLSLLQATDFMSL

43.>sp|Q96PD4|IL17F_HUMANInterleukin-17FOS=HomosapiensGN=IL17FPE=1SV=3

MTVKTLHGPAMVKYLLLSILGLAFLSEAAARKIPKVGHTFFQKPESCPPVPGGSMKLDIG

IINENQRVSMSRNIESRSTSPWNYTVTWDPNRYPSEVVQAQCRNLGCINAQGKEDISMNS

VPIQQETLVVRRKHQGCSVSFQLEKVLVTVGCTCVTPVIHHVQ

44.>sp|P01185|NEU2_HUMANVasopressin-neurophysin2-copeptinOS=HomosapiensGN=AVPPE=1SV=2

MPDTMLPACFLGLLAFSSACYFQNCPRGGKRAMSDLELRQCLPCGPGGKGRCFGPSICCA

DELGCFVGTAEALRCQEENYLPSPCQSGQKACGSGGRCAAFGVCCNDESCVTEPECREGF

HRRARASDRSNATQLDGPAGALLLRLVQLAGAPEPFEPAQPDAY

45.>sp|P01233|CGHB_HUMANChoriogonadotropinsubunitbetaOS=HomosapiensGN=CGBPE=1SV=1

MEMFQGLLLLLLLSMGGTWASKEPLRPRCRPINATLAVEKEGCPVCITVNTTICAGYCPT

MTRVLQGVLPALPQVVCNYRDVRFESIRLPGCPRGVNPVVSYAVALSCQCALCRRSTTDC

GGPKDHPLTCDDPRFQDSSSSKAPPPSLPSPSRLPGPSDTPILPQ

46.>sp|P01579|IFNG_HUMANInterferongammaOS=HomosapiensGN=IFNGPE=1SV=1

MKYTSYILAFQLCIVLGSLGCYCQDPYVKEAENLKKYFNAGHSDVADNGTLFLGILKNWK

EESDRKIMQSQIVSFYFKLFKNFKDDQSIQKSVETIKEDMNVKFFNSNKKKRDDFEKLTN

YSVTDLNVQRKAIHELIQVMAELSPAAKTGKRKRSQMLFRGRRASQ

47.>sp|Q13361|MFAP5_HUMANMicrofibrillar-associatedprotein5OS=HomosapiensGN=MFAP5PE=1SV=1

MSLLGPKVLLFLAAFIITSDWIPLGVNSQRGDDVTQATPETFTEDPNLVNDPATDETVLA

VLADIAPSTDDLASLSEKNTTAECWDEKFTCTRLYSVHRPVKQCIHQLCFTSLRRMYIVN

KEICSRLVCKEHEAMKDELCRQMAGLPPRRLRRSNYFRLPPCENVDLQRPNGL

48.>sp|Q6UVW9|CLC2A_HUMANC-typelectindomainfamily2memberAOS=HomosapiensGN=CLEC2APE=1SV=2

MINPELRDGRADGFIHRIVPKLIQNWKIGLMCFLSIIITTVCIIMIATWSKHAKPVACSG

DWLGVRDKCFYFSDDTRNWTASKIFCSLQKAELAQIDTQEDMEFLKRYAGTDMHWIGLSR

KQGDSWKWTNGTTFNGWFEIIGNGSFAFLSADGVHSSRGFIDIKWICSKPKYFL

49.>sp|Q969L2|MAL2_HUMANProteinMAL2OS=HomosapiensGN=MAL2PE=1SV=1

MSAGGASVPPPPNPAVSFPPPRVTLPAGPDILRTYSGAFVCLEILFGGLVWILVASSNVP

LPLLQGWVMFVSVTAFFFSLLFLGMFLSGMVAQIDANWNFLDFAYHFTVFVFYFGAFLLE

AAATSLHDLHCNTTITGQPLLSDNQYNINVAASIFAFMTTACYGCSLGLALRRWRP

50.>sp|P18510|IL1RA_HUMANInterleukin-1receptorantagonistproteinOS=HomosapiensGN=IL1RNPE=1SV=1

MEICRGLRSHLITLLLFLFHSETICRPSGRKSSKMQAFRIWDVNQKTFYLRNNQLVAGYL

QGPNVNLEEKIDVVPIEPHALFLGIHGGKMCLSCVKSGDETRLQLEAVNITDLSENRKQD

KRFAFIRSDSGPTTSFESAACPGWFLCTAMEADQPVSLTNMPDEGVMVTKFYFQEDE

51.>sp|Q9GZX6|IL22_HUMANInterleukin-22OS=HomosapiensGN=IL22PE=1SV=1

MAALQKSVSSFLMGTLATSCLLLLALLVQGGAAAPISSHCRLDKSNFQQPYITNRTFMLA

KEASLADNNTDVRLIGEKLFHGVSMSERCYLMKQVLNFTLEEVLFPQSDRFQPYMQEVVP

FLARLSNRLSTCHIEGDDLHIQRNVQKLKDTVKKLGESGEIKAIGELDLLFMSLRNACI

52.>sp|Q10589|BST2_HUMANBonemarrowstromalantigen2OS=HomosapiensGN=BST2PE=1SV=1

MASTSYDYCRVPMEDGDKRCKLLLGIGILVLLIIVILGVPLIIFTIKANSEACRDGLRAV

MECRNVTHLLQQELTEAQKGFQDVEAQAATCNHTVMALMASLDAEKAQGQKKVEELEGEI

TTLNHKLQDASAEVERLRRENQVLSVRIADKKYYPSSQDSSSAAAPQLLIVLLGLSALLQ

53.>sp|P09466|PAEP_HUMANGlycodelinOS=HomosapiensGN=PAEPPE=1SV=2

MLCLLLTLGVALVCGVPAMDIPQTKQDLELPKLAGTWHSMAMATNNISLMATLKAPLRVH

ITSLLPTPEDNLEIVLHRWENNSCVEKKVLGEKTENPKKFKINYTVANEATLLDTDYDNF

LFLCLQDTTTPIQSMMCQYLARVLVEDDEIMQGFIRAFRPLPRHLWYLLDLKQMEEPCRF

54.>sp|P61009|SPCS3_HUMANSignalpeptidasecomplexsubunit3OS=HomosapiensGN=SPCS3PE=1SV=1

MNTVLSRANSLFAFSLSVMAALTFGCFITTAFKDRSVPVRLHVSRIMLKNVEDFTGPRER

SDLGFITFDITADLENIFDWNVKQLFLYLSAEYSTKNNALNQVVLWDKIVLRGDNPKLLL

KDMKTKYFFFDDGNGLKGNRNVTLTLSWNVVPNAGILPLVTGSGHVSVPFPDTYEITKSY

55.>sp|P43308|SSRB_HUMANTranslocon-associatedproteinsubunitbetaOS=HomosapiensGN=SSR2PE=1SV=1

MRLLSFVVLALFAVTQAEEGARLLASKSLLNRYAVEGRDLTLQYNIYNVGSSAALDVELS

DDSFPPEDFGIVSGMLNVKWDRIAPASNVSHTVVLRPLKAGYFNFTSATITYLAQEDGPV

VIGSTSAPGQGGILAQREFDRRFSPHFLDWAAFGVMTLPSIGIPLLLWYSSKRKYDTPKT

KKN

56.>sp|P23510|TNFL4_HUMANTumornecrosisfactorligandsuperfamilymember4OS=HomosapiensGN=TNFSF4PE=1SV=1

MERVQPLEENVGNAARPRFERNKLLLVASVIQGLGLLLCFTYICLHFSALQVSHRYPRIQ

SIKVQFTEYKKEKGFILTSQKEDEIMKVQNNSVIINCDGFYLISLKGYFSQEVNISLHYQ

KDEEPLFQLKKVRSVNSLMVASLTYKDKVYLNVTTDNTSLDDFHVNGGELILIHQNPGEF

CVL

57.>sp|P01574|IFNB_HUMANInterferonbetaOS=HomosapiensGN=IFNB1PE=1SV=1

MTNKCLLQIALLLCFSTTALSMSYNLLGFLQRSSNFQCQKLLWQLNGRLEYCLKDRMNFD

IPEEIKQLQQFQKEDAALTIYEMLQNIFAIFRQDSSSTGWNETIVENLLANVYHQINHLK

TVLEEKLEKEDFTRGKLMSSLHLKRYYGRILHYLKAKEYSHCAWTIVRVEILRNFYFINR

LTGYLRN

58.>sp|O95445|APOM_HUMANApolipoproteinMOS=HomosapiensGN=APOMPE=1SV=2

MFHQIWAALLYFYGIILNSIYQCPEHSQLTTLGVDGKEFPEVHLGQWYFIAGAAPTKEEL

ATFDPVDNIVFNMAAGSAPMQLHLRATIRMKDGLCVPRKWIYHLTEGSTDLRTEGRPDMK

TELFSSSCPGGIMLNETGQGYQRFLLYNRSPHPPEKCVEEFKSLTSCLDSKAFLLTPRNQ

EACELSNN

59.>sp|Q9BSG0|PADC1_HUMANProtease-associateddomain-containingprotein1OS=HomosapiensGN=PRADC1PE=1SV=1

MVPGAAGWCCLVLWLPACVAAHGFRIHDYLYFQVLSPGDIRYIFTATPAKDFGGIFHTRY

EQIHLVPAEPPEACGELSNGFFIQDQIALVERGGCSFLSKTRVVQEHGGRAVIISDNAVD

NDSFYVEMIQDSTQRTADIPALFLLGRDGYMIRRSLEQHGLPWAIISIPVNVTSIPTFEL

LQPPWTFW

60.>sp|P05090|APOD_HUMANApolipoproteinDOS=HomosapiensGN=APODPE=1SV=1

MVMLLLLLSALAGLFGAAEGQAFHLGKCPNPPVQENFDVNKYLGRWYEIEKIPTTFENGR

CIQANYSLMENGKIKVLNQELRADGTVNQIEGEATPVNLTEPAKLEVKFSWFMPSAPYWI

LATDYENYALVYSCTCIIQLFHVDFAWILARNPNLPPETVDSLKNILTSNNIDVKKMTVT

DQVNCPKLS

61.>sp|P01570|IFN14_HUMANInterferonalpha-14OS=HomosapiensGN=IFNA14PE=1SV=3

MALPFALMMALVVLSCKSSCSLGCNLSQTHSLNNRRTLMLMAQMRRISPFSCLKDRHDFE

FPQEEFDGNQFQKAQAISVLHEMMQQTFNLFSTKNSSAAWDETLLEKFYIELFQQMNDLE

ACVIQEVGVEETPLMNEDSILAVKKYFQRITLYLMEKKYSPCAWEVVRAEIMRSLSFSTN

LQKRLRRKD

62.>sp|O15431|COPT1_HUMANHighaffinitycopperuptakeprotein1OS=HomosapiensGN=SLC31A1PE=1SV=1

MDHSHHMGMSYMDSNSTMQPSHHHPTTSASHSHGGGDSSMMMMPMTFYFGFKNVELLFSG

LVINTAGEMAGAFVAVFLLAMFYEGLKIARESLLRKSQVSIRYNSMPVPGPNGTILMETH

KTVGQQMLSFPHLLQTVLHIIQVVISYFLMLIFMTYNGYLCIAVAAGAGTGYFLFSWKKA

VVVDITEHCH

63.>sp|P41222|PTGDS_HUMANProstaglandin-H2D-isomeraseOS=HomosapiensGN=PTGDSPE=1SV=1

MATHHTLWMGLALLGVLGDLQAAPEAQVSVQPNFQQDKFLGRWFSAGLASNSSWLREKKA

ALSMCKSVVAPATDGGLNLTSTFLRKNQCETRTMLLQPAGSLGSYSYRSPHWGSTYSVSV

VETDYDQYALLYSQGSKGPGEDFRMATLYSRTQTPRAELKEKFTAFCKAQGFTEDTIVFL

PQTDKCMTEQ

64.>sp|Q7Z4R8|CF120_HUMANUPF0669proteinC6orf120OS=HomosapiensGN=C6orf120PE=1SV=1

MAAPRGRAAPWTTALLLLLASQVLSPGSCADEEEVPEEWVLLHVVQGQIGAGNYSYLRLN

HEGKIVLRMRSLKGDADLYVSASSLHPSFDDYELQSATCGPDAVSIPAHFRRPVGIGVYG

HPSHLESEFEMKVYYDGTVEQHPFGEAAYPADGADAGQKHAGAPEDASQEEESVLWTILI

SILKLVLEILF

65.>sp|P01588|EPO_HUMAN ErythropoietinOS=HomosapiensGN=EPOPE=1SV=1

MGVHECPAWLWLLLSLLSLPLGLPVLGAPPRLICDSRVLERYLLEAKEAENITTGCAEHC

SLNENITVPDTKVNFYAWKRMEVGQQAVEVWQGLALLSEAVLRGQALLVNSSQPWEPLQL

HVDKAVSGLRSLTTLLRALGAQKEAISPPDAASAAPLRTITADTFRKLFRVYSNFLRGKL

KLYTGEACRTGDR

66.>sp|P17900|SAP3_HUMANGangliosideGM2activatorOS=HomosapiensGN=GM2APE=1SV=4

MQSLMQAPLLIALGLLLAAPAQAHLKKPSQLSSFSWDNCDEGKDPAVIRSLTLEPDPIIV

PGNVTLSVMGSTSVPLSSPLKVDLVLEKEVAGLWIKIPCTDYIGSCTFEHFCDVLDMLIP

TGEPCPEPLRTYGLPCHCPFKEGTYSLPKSEFVVPDLELPSWLTTGNYRIESVLSSSGKR

LGCIKIAASLKGI

67.>sp|O95998|I18BP_HUMANInterleukin-18-bindingproteinOS=HomosapiensGN=IL18BPPE=1SV=2

MTMRHNWTPDLSPLWVLLLCAHVVTLLVRATPVSQTTTAATASVRSTKDPCPSQPPVFPA

AKQCPALEVTWPEVEVPLNGTLSLSCVACSRFPNFSILYWLGNGSFIEHLPGRLWEGSTS

RERGSTGTQLCKALVLEQLTPALHSTNFSCVLVDPEQVVQRHVVLAQLWAGLRATLPPTQ

EALPSSHSSPQQQG

68.>sp|P05000|IFNW1_HUMANInterferonomega-1OS=HomosapiensGN=IFNW1PE=1SV=2

MALLFPLLAALVMTSYSPVGSLGCDLPQNHGLLSRNTLVLLHQMRRISPFLCLKDRRDFR

FPQEMVKGSQLQKAHVMSVLHEMLQQIFSLFHTERSSAAWNMTLLDQLHTGLHQQLQHLE

TCLLQVVGEGESAGAISSPALTLRRYFQGIRVYLKEKKYSDCAWEVVRMEIMKSLFLSTN

MQERLRSKDRDLGSS

69.>sp|Q9Y3P8|SIT1_HUMANSignalingthreshold-regulatingtransmembraneadapter1OS=HomosapiensGN=SIT1PE=1SV=1

MNQADPRLRAVCLWTLTSAAMSRGDNCTDLLALGIPSITQAWGLWVLLGAVTLLFLISLA

AHLSQWTRGRSRSHPGQGRSGESVEEVPLYGNLHYLQTGRLSQDPEPDQQDPTLGGPARA

AEEVMCYTSLQLRPPQGRIPGPGTPVKYSEVVLDSEPKSQASGPEPELYASVCAQTRRAR

ASFPDQAYANSQPAAS

70.>sp|P80188|NGAL_HUMANNeutrophilgelatinase-associatedlipocalinOS=HomosapiensGN=LCN2PE=1SV=2

MPLGLLWLGLALLGALHAQAQDSTSDLIPAPPLSKVPLQQNFQDNQFQGKWYVVGLAGNA

ILREDKDPQKMYATIYELKEDKSYNVTSVLFRKKKCDYWIRTFVPGCQPGEFTLGNIKSY

PGLTSYLVRVVSTNYNQHAMVFFKKVSQNREYFKITLYGRTKELTSELKENFIRFSKSLG

LPENHIVFPVPIDQCIDG

71.>sp|P02763|A1AG1_HUMANAlpha-1-acidglycoprotein1OS=HomosapiensGN=ORM1PE=1SV=1

MALSWVLTVLSLLPLLEAQIPLCANLVPVPITNATLDQITGKWFYIASAFRNEEYNKSVQ

EIQATFFYFTPNKTEDTIFLREYQTRQDQCIYNTTYLNVQRENGTISRYVGGQEHFAHLL

ILRDTKTYMLAFDVNDEKNWGLSVYADKPETTKEQLGEFYEALDCLRIPKSDVVYTDWKK

DKCEPLEKQHEKERKQEEGES

72.>sp|P19652|A1AG2_HUMANAlpha-1-acidglycoprotein2OS=HomosapiensGN=ORM2PE=1SV=2

MALSWVLTVLSLLPLLEAQIPLCANLVPVPITNATLDRITGKWFYIASAFRNEEYNKSVQ

EIQATFFYFTPNKTEDTIFLREYQTRQNQCFYNSSYLNVQRENGTVSRYEGGREHVAHLL

FLRDTKTLMFGSYLDDEKNWGLSFYADKPETTKEQLGEFYEALDCLCIPRSDVMYTDWKK

DKCEPLEKQHEKERKQEEGES

73.>sp|P15018|LIF_HUMANLeukemiainhibitoryfactorOS=HomosapiensGN=LIFPE=1SV=1

MKVLAAGVVPLLLVLHWKHGAGSPLPITPVNATCAIRHPCHNNLMNQIRSQLAQLNGSAN

ALFILYYTAQGEPFPNNLDKLCGPNVTDFPPFHANGTEKAKLVELYRIVVYLGTSLGNIT

RDQKILNPSALSLHSKLNATADILRGLLSNVLCRLCSKYHVGHVDVTYGPDTSGKDVFQK

KKLGCQLLGKYKQIIAVLAQAF

74.>sp|P48230|T4S4_HUMANTransmembrane4L6familymember4OS=HomosapiensGN=TM4SF4PE=1SV=1

MCTGGCARCLGGTLIPLAFFGFLANILLFFPGGKVIDDNDHLSQEIWFFGGILGSGVLMI

FPALVFLGLKNNDCCGCCGNEGCGKRFAMFTSTIFAVVGFLGAGYSFIISAISINKGPKC

LMANSTWGYPFHDGDYLNDEALWNKCREPLNVVPWNLTLFSILLVVGGIQMVLCAIQVVN

GLLGTLCGDCQCCGCCGGDGPV

75.>sp|Q01151|CD83_HUMANCD83antigenOS=HomosapiensGN=CD83PE=1SV=1

MSRGLQLLLLSCAYSLAPATPEVKVACSEDVDLPCTAPWDPQVPYTVSWVKLLEGGEERM

ETPQEDHLRGQHYHQKGQNGSFDAPNERPYSLKIRNTTSCNSGTYRCTLQDPDGQRNLSG

KVILRVTGCPAQRKEETFKKYRAEIVLLLALVIFYLTLIIFTCKFARLQSIFPDFSKAGM

ERAFLPVTSPNKHLGLVTPHKTELV

(2^nd^ N in D.R)

76.>sp|P20827|EFNA1_HUMANEphrin-A1OS=HomosapiensGN=EFNA1PE=1SV=2

MEFLWAPLLGLCCSLAAADRHTVFWNSSNPKFRNEDYTIHVQLNDYVDIICPHYEDHSVA

DAAMEQYILYLVEHEEYQLCQPQSKDQVRWQCNRPSAKHGPEKLSEKFQRFTPFTLGKEF

KEGHSYYYISKPIHQHEDRCLRLKVTVSGKITHSPQAHDNPQEKRLAADDPEVRVLHSIG

HSAAPRLFPLAWTVLLLPLLLLQTP

77.>sp|Q96G30|MRAP2_HUMANMelanocortin-2receptoraccessoryprotein2OS=HomosapiensGN=MRAP2PE=1SV=2

MSAQRLISNRTSQQSASNSDYTWEYEYYEIGPVSFEGLKAHKYSIVIGFWVGLAVFVIFM

FFVLTLLTKTGAPHQDNAESSEKRFRMNSFVSDFGRPLEPDKVFSRQGNEESRSLFHCYI

NEVERLDRAKACHQTTALDSDVQLQEAIRSSGQPEEELNRLMKFDIPNFVNTDQNYFGED

DLLISEPPIVLETKPLSQTSHKDLD

78.>sp|P01374|TNFB_HUMANLymphotoxin-alphaOS=HomosapiensGN=LTAPE=1SV=2

MTPPERLFLPRVCGTTLHLLLLGLLLVLLPGAQGLPGVGLTPSAAQTARQHPKMHLAHST

LKPAAHLIGDPSKQNSLLWRANTDRAFLQDGFSLSNNSLLVPTSGIYFVYSQVVFSGKAY

SPKATSSPLYLAHEVQLFSSQYPFHVPLLSSQKMVYPGLQEPWLHSMYHGAAFQLTQGDQ

LSTHTDGIPHLVLSPSTVFFGAFAL

79.>sp|P13224|GP1BB_HUMANPlateletglycoproteinIbbetachainOS=HomosapiensGN=GP1BBPE=1SV=1

MGSGPRGALSLLLLLLAPPSRPAAGCPAPCSCAGTLVDCGRRGLTWASLPTAFPVDTTEL

VLTGNNLTALPPGLLDALPALRTAHLGANPWRCDCRLVPLRAWLAGRPERAPYRDLRCVA

PPALRGRLLPYLAEDELRAACAPGPLCWGALAAQLALLGLGLLHALLLVLLLCRLRRLRA

RARARAAARLSLTDPLVAERAGTDES

80.>sp|P01033|TIMP1_HUMANMetalloproteinaseinhibitor1OS=HomosapiensGN=TIMP1PE=1SV=1

MAPFEPLASGILLLLWLIAPSRACTCVPPHPQTAFCNSDLVIRAKFVGTPEVNQTTLYQR

YEIKMTKMYKGFQALGDAADIRFVYTPAMESVCGYFHRSHNRSEEFLIAGKLQDGLLHIT

TCSFVAPWNSLSLAQRRGFTKTYTVGCEECTVFPCLSIPCKLQSGTHCLWTDQLLQGSEK

GFQSRHLACLPREPGLCTWQSLRSQIA

81.>sp|P31371|FGF9_HUMANFibroblastgrowthfactor9OS=HomosapiensGN=FGF9PE=1SV=3

MAPLGEVGNYFGVQDAVPFGNVPVLPVDSPVLLSDHLGQSEAGGLPRGPAVTDLDHLKGI

LRRRQLYCRTGFHLEIFPNGTIQGTRKDHSRFGILEFISIAVGLVSIRGVDSGLYLGMNE

KGELYGSEKLTQECVFREQFEENWYNTYSSNLYKHVDTGRRYYVALNKDGTPREGTRTKR

HQKFTHFLPRPVDPDKVPELYKDILSQS

82.>sp|Q86W47|KCMB4_HUMANCalcium-activatedpotassiumchannelsubunitbeta-4OS=HomosapiensGN=KCNMB4PE=1SV=2

MAKLRVAYEYTEAEDKSIRLGLFLIISGVVSLFIFGFCWLSPALQDLQATEANCTVLSVQ

QIGEVFECTFTCGADCRGTSQYPCVQVYVNNSESNSRALLHSDEHQLLTNPKCSYIPPCK

RENQKNLESVMNWQQYWKDEIGSQPFTCYFNQHQRPDDVLLHRTHDEIVLLHCFLWPLVT

FVVGVLIVVLTICAKSLAVKAEAMKKRKFS

83.>sp|Q8TAL6|FIBIN_HUMANFinbudinitiationfactorhomologOS=HomosapiensGN=FIBINPE=1SV=1

MVFLKFFCMSFFCHLCQGYFDGPLYPEMSNGTLHHYFVPDGDYEENDDPEKCQLLFRVSD

HRRCSQGEGSQVGSLLSLTLREEFTVLGRQVEDAGRVLEGISKSISYDLDGEESYGKYLR

RESHQIGDAYSNSDKSLTELESKFKQGQEQDSRQESRLNEDFLGMLVHTRSLLKETLDIS

VGLRDKYELLALTIRSHGTRLGRLKNDYLKV

84.>sp|P39905|GDNF_HUMANGlialcellline-derivedneurotrophicfactorOS=HomosapiensGN=GDNFPE=1SV=1

MKLWDVVAVCLVLLHTASAFPLPAGKRPPEAPAEDRSLGRRRAPFALSSDSNMPEDYPDQ

FDDVMDFIQATIKRLKRSPDKQMAVLPRRERNRQAAAANPENSRGKGRRGQRGKNRGCVL

TAIHLNVTDLGLGYETKEELIFRYCSGSCDAAETTYDKILKNLSRNRRLVSDKVGQACCR

PIAFDDDLSFLDDNLVYHILRKHSAKRCGCI

85.>sp|P05231|IL6_HUMANInterleukin-6OS=HomosapiensGN=IL6PE=1SV=1

MNSFSTSAFGPVAFSLGLLLVLPAAFPAPVPPGEDSKDVAAPHRQPLTSSERIDKQIRYI

LDGISALRKETCNKSNMCESSKEALAENNLNLPKMAEKDGCFQSGFNEETCLVKIITGLL

EFEVYLEYLQNRFESSEEQARAVQMSTKVLIQFLQKKAKNLDAITTPDPTTNASLLTKLQ

AQNQWLQDMTTHLILRSFKEFLQSSLRALRQM

86.>sp|O60487|MPZL2_HUMANMyelinproteinzero-likeprotein2OS=HomosapiensGN=MPZL2PE=1SV=1

MYGKSSTRAVLLLLGIQLTALWPIAAVEIYTSRVLEAVNGTDARLKCTFSSFAPVGDALT

VTWNFRPLDGGPEQFVFYYHIDPFQPMSGRFKDRVSWDGNPERYDASILLWKLQFDDNGT

YTCQVKNPPDVDGVIGEIRLSVVHTVRFSEIHFLALAIGSACALMIIIVIVVVLFQHYRK

KRWAERAHKVVEIKSKEEERLNQEKKVSVYLEDTD

87.>sp|P10747|CD28_HUMANT-cell-specificsurfaceglycoproteinCD28OS=HomosapiensGN=CD28PE=1SV=1

MLRLLLALNLFPSIQVTGNKILVKQSPMLVAYDNAVNLSCKYSYNLFSREFRASLHKGLD

SAVEVCVVYGNYSQQLQVYSKTGFNCDGKLGNESVTFYLQNLYVNQTDIYFCKIEVMYPP

PYLDNEKSNGTIIHVKGKHLCPSPLFPGPSKPFWVLVVVGGVLACYSLLVTVAFIIFWVR

SKRSRLLHSDYMNMTPRRPGPTRKHYQPYAPPRDFAAYRS

88.>sp|O75629|CREG1_HUMANProteinCREG1OS=HomosapiensGN=CREG1PE=1SV=1

MAGLSRGSARALLAALLASTLLALLVSPARGRGGRDHGDWDEASRLPPLPPREDAARVAR

FVTHVSDWGALATISTLEAVRGRPFADVLSLSDGPPGAGSGVPYFYLSPLQLSVSNLQEN

PYATLTMTLAQTNFCKKHGFDPQSPLCVHIMLSGTVTKVNETEMDIAKHSLFIRHPEMKT

WPSSHNWFFAKLNITNIWVLDYFGGPKIVTPEEYYNVTVQ

89.>sp|P13727|PRG2_HUMANBonemarrowproteoglycanOS=HomosapiensGN=PRG2PE=1SV=2

MKLPLLLALLFGAVSALHLRSETSTFETPLGAKTLPEDEETPEQEMEETPCRELEEEEEW

GSGSEDASKKDGAVESISVPDMVDKNLTCPEEEDTVKVVGIPGCQTCRYLLVRSLQTFSQ

AWFTCRRCYRGNLVSIHNFNINYRIQCSVSALNQGQVWIGGRITGSGRCRRFQWVDGSRW

NFAYWAAHQPWSRGGHCVALCTRGGHWRRAHCLRRLPFICSY

90.>sp|P16410|CTLA4_HUMANCytotoxicT-lymphocyteprotein4OS=HomosapiensGN=CTLA4PE=1SV=3

MACLGFQRHKAQLNLATRTWPCTLLFFLLFIPVFCKAMHVAQPAVVLASSRGIASFVCEY

ASPGKATEVRVTVLRQADSQVTEVCAATYMMGNELTFLDDSICTGTSSGNQVNLTIQGLR

AMDTGLYICKVELMYPPPYYLGIGNGTQIYVIDPEPCPDSDFLLWILAAVSSGLFFYSFL

LTAVSLSKMLKKRSPLTTGVYVKMPPTEPECEKQFQPYFIPIN

91.>sp|P02743|SAMP_HUMANSerumamyloidP-componentOS=HomosapiensGN=APCSPE=1SV=2

MNKPLLWISVLTSLLEAFAHTDLSGKVFVFPRESVTDHVNLITPLEKPLQNFTLCFRAYS

DLSRAYSLFSYNTQGRDNELLVYKERVGEYSLYIGRHKVTSKVIEKFPAPVHICVSWESS

SGIAEFWINGTPLVKKGLRQGYFVEAQPKIVLGQEQDSYGGKFDRSQSFVGEIGDLYMWD

SVLPPENILSAYQGTPLPANILDWQALNYEIRGYVIIKPLVWV

92.>sp|Q9UM22|EPDR1_HUMANMammalianependymin-relatedprotein1OS=HomosapiensGN=EPDR1PE=1SV=2

MPGRAPLRTVPGALGAWLLGGLWAWTLCGLCSLGAVGAPRPCQAPQQWEGRQVMYQQSSG

RNSRALLSYDGLNQRVRVLDERKALIPCKRLFEYILLYKDGVMFQIDQATKQCSKMTLTQ

PWDPLDIPQNSTFEDQYSIGGPQEQITVQEWSDRKSARSYETWIGIYTVKDCYPVQETFT

INYSVILSTRFFDIQLGIKDPSVFTPPSTCQMAQLEKMSEDCSW

93.>sp|Q96BQ1|FAM3D_HUMANProteinFAM3DOS=HomosapiensGN=FAM3DPE=1SV=1

MRVSGVLRLLALIFAIVTTWMFIRSYMSFSMKTIRLPRWLAASPTKEIQVKKYKCGLIKP

CPANYFAFKICSGAANVVGPTMCFEDRMIMSPVKNNVGRGLNIALVNGTTGAVLGQKAFD

MYSGDVMHLVKFLKEIPGGALVLVASYDDPGTKMNDESRKLFSDLGSSYAKQLGFRDSWV

FIGAKDLRGKSPFEQFLKNSPDTNKYEGWPELLEMEGCMPPKPF

94.>sp|P01236|PRL_HUMANProlactinOS=HomosapiensGN=PRLPE=1SV=1

MNIKGSPWKGSLLLLLVSNLLLCQSVAPLPICPGGAARCQVTLRDLFDRAVVLSHYIHNL

SSEMFSEFDKRYTHGRGFITKAINSCHTSSLATPEDKEQAQQMNQKDFLSLIVSILRSWN

EPLYHLVTEVRGMQEAPEAILSKAVEIEEQTKRLLEGMELIVSQVHPETKENEIYPVWSG

LPSLQMADEESRLSAYYNLLHCLRRDSHKIDNYLKLLKCRIIHNNNC

95.>sp|Q7Z7H5|TMED4_HUMANTransmembraneemp24domain-containingprotein4OS=HomosapiensGN=TMED4PE=1SV=1

MAGVGAGPLRAMGRQALLLLALCATGAQGLYFHIGETEKRCFIEEIPDETMVIGNYRTQM

WDKQKEVFLPSTPGLGMHVEVKDPDGKVVLSRQYGSEGRFTFTSHTPGDHQICLHSNSTR

MALFAGGKLRVHLDIQVGEHANNYPEIAAKDKLTELQLRARQLLDQVEQIQKEQDYQRYR

EERFRLTSESTNQRVLWWSIAQTVILILTGIWQMRHLKSFFEAKKLV

96.>sp|P52803|EFNA5_HUMANEphrin-A5OS=HomosapiensGN=EFNA5PE=1SV=1

MLHVEMLTLVFLVLWMCVFSQDPGSKAVADRYAVYWNSSNPRFQRGDYHIDVCINDYLDV

FCPHYEDSVPEDKTERYVLYMVNFDGYSACDHTSKGFKRWECNRPHSPNGPLKFSEKFQL

FTPFSLGFEFRPGREYFYISSAIPDNGRRSCLKLKVFVRPTNSCMKTIGVHDRVFDVNDK

VENSLEPADDTVHESAEPSRGENAAQTPRIPSRLLAILLFLLAMLLTL

97.>sp|Q5VTT2|CI135_HUMANUncharacterizedproteinC9orf135OS=HomosapiensGN=C9orf135PE=1SV=1

MDSLDRSCQDWCDRKQHWLEIGPPDLVERKGSLTLRSHHKKYSKPVLVYSWHRDREAFPK

GYDIEGPEKVKKLCNSTYRRLGTDESPIWTSETHEKLSQMCLNTEWVEMKSKALLNEETV

SSGIIERVTGLPATGFGAVFPRHPPDWSKMCALTTYSEDYVPPYDYQPHAYPCQDDYSIV

HRKCRSQFTDLNGSKRFGINTWHDESGIYANSDVKQKLYPLTSGPIVPI

98.>sp|P15692|VEGFA_HUMANVascularendothelialgrowthfactorAOS=HomosapiensGN=VEGFAPE=1SV=2

MNFLLSWVHWSLALLLYLHHAKWSQAAPMAEGGGQNHHEVVKFMDVYQRSYCHPIETLVD

IFQEYPDEIEYIFKPSCVPLMRCGGCCNDEGLECVPTEESNITMQIMRIKPHQGQHIGEM

SFLQHNKCECRPKKDRARQEKKSVRGKGKGQKRKRKKSRYKSWSVYVGARCCLMPWSLPG

PHPCGPCSERRKHLFVQDPQTCKCSCKNTDSRCKARQLELNERTCRCDKPRR

99.>sp|Q9BVK6|TMED9_HUMANTransmembraneemp24domain-containingprotein9OS=HomosapiensGN=TMED9PE=1SV=2

MAVELGVLLVRPRPGTGLGRVMRTLLLVLWLATRGSALYFHIGETEKKCFIEEIPDETMV

IGNYRTQLYDKQREEYQPATPGLGMFVEVKDPEDKVILARQYGSEGRFTFTSHTPGEHQI

CLHSNSTKFSLFAGGMLRVHLDIQVGEHANDYAEIAAKDKLSELQLRVRQLVEQVEQIQK

EQNYQRWREERFRQTSESTNQRVLWWSILQTLILVAIGVWQMRHLKSFFEAKKLV

100.>sp|P19075|TSN8_HUMANTetraspanin-8OS=HomosapiensGN=TSPAN8PE=1SV=1

MAGVSACIKYSMFTFNFLFWLCGILILALAIWVRVSNDSQAIFGSEDVGSSSYVAVDILI

AVGAIIMILGFLGCCGAIKESRCMLLLFFIGLLLILLLQVATGILGAVFKSKSDRIVNET

LYENTKLLSATGESEKQFQEAIIVFQEEFKCCGLVNGAADWGNNFQHYPELCACLDKQRP

CQSYNGKQVYKETCISFIKDFLAKNLIIVIGISFGLAVIEILGLVFSMVLYCQIGNK

101.>sp|P08962|CD63_HUMANCD63antigenOS=HomosapiensGN=CD63PE=1SV=2

MAVEGGMKCVKFLLYVLLLAFCACAVGLIAVGVGAQLVLSQTIIQGATPGSLLPVVIIAV

GVFLFLVAFVGCCGACKENYCLMITFAIFLSLIMLVEVAAAIAGYVFRDKVMSEFNNNFR

QQMENYPKNNHTASILDRMQADFKCCGAANYTDWEKIPSMSKNRVPDSCCINVTVGCGIN

FNEKAIHKEGCVEKIGGWLRKNVLVVAAAALGIAFVEVLGIVFACCLVKSIRSGYEVM

102.>sp|Q9UNN8|EPCR_HUMANEndothelialproteinCreceptorOS=HomosapiensGN=PROCRPE=1SV=1

MLTTLLPILLLSGWAFCSQDASDGLQRLHMLQISYFRDPYHVWYQGNASLGGHLTHVLEG

PDTNTTIIQLQPLQEPESWARTQSGLQSYLLQFHGLVRLVHQERTLAFPLTIRCFLGCEL

PPEGSRAHVFFEVAVNGSSFVSFRPERALWQADTQVTSGVVTFTLQQLNAYNRTRYELRE

FLEDTCVQYVQKHISAENTKGSQTSRSYTSLVLGVLVGSFIIAGVAVGIFLCTGGRRC

103.>sp|O14817|TSN4_HUMANTetraspanin-4OS=HomosapiensGN=TSPAN4PE=1SV=1

MARACLQAVKYLMFAFNLLFWLGGCGVLGVGIWLAATQGSFATLSSSFPSLSAANLLIIT

GAFVMAIGFVGCLGAIKENKCLLLTFFLLLLLVFLLEATIAILFFAYTDKIDRYAQQDLK

KGLHLYGTQGNVGLTNAWSIIQTDFRCCGVSNYTDWFEVYNATRVPDSCCLEFSESCGLH

APGTWWKAPCYETVKVWLQENLLAVGIFGLCTALVQILGLTFAMTMYCQVVKADTYCA

104.>sp|P09564|CD7_HUMANT-cellantigenCD7OS=HomosapiensGN=CD7PE=1SV=1

MAGPPRLLLLPLLLALARGLPGALAAQEVQQSPHCTTVPVGASVNITCSTSGGLRGIYLR

QLGPQPQDIIYYEDGVVPTTDRRFRGRIDFSGSQDNLTITMHRLQLSDTGTYTCQAITEV

NVYGSGTLVLVTEEQSQGWHRCSDAPPRASALPAPPTGSALPDPQTASALPDPPAASALP

AALAVISFLLGLGLGVACVLARTQIKKLCSWRDKNSAACVVYEDMSHSRCNTLSSPNQYQ

105.>sp|P08294|SODE_HUMANExtracellularsuperoxidedismutase[Cu-Zn]OS=HomosapiensGN=SOD3PE=1SV=2

MLALLCSCLLLAAGASDAWTGEDSAEPNSDSAEWIRDMYAKVTEIWQEVMQRRDDDGALH

AACQVQPSATLDAAQPRVTGVVLFRQLAPRAKLDAFFALEGFPTEPNSSSRAIHVHQFGD

LSQGCESTGPHYNPLAVPHPQHPGDFGNFAVRDGSLWRYRAGLAASLAGPHSIVGRAVVV

HAGEDDLGRGGNQASVENGNAGRRLACCVVGVCGPGLWERQAREHSERKKRRRESECKAA

106.>sp|O43557|TNF14_HUMANTumornecrosisfactorligandsuperfamilymember14OS=HomosapiensGN=TNFSF14PE=1SV=2

MEESVVRPSVFVVDGQTDIPFTRLGRSHRRQSCSVARVGLGLLLLLMGAGLAVQGWFLLQ

LHWRLGEMVTRLPDGPAGSWEQLIQERRSHEVNPAAHLTGANSSLTGSGGPLLWETQLGL

AFLRGLSYHDGALVVTKAGYYYIYSKVQLGGVGCPLGLASTITHGLYKRTPRYPEELELL

VSQQSPCGRATSSSRVWWDSSFLGGVVHLEAGEKVVVRVLDERLVRLRDGTRSYFGAFMV

107.>sp|O60635|TSN1_HUMANTetraspanin-1OS=HomosapiensGN=TSPAN1PE=1SV=2

MQCFSFIKTMMILFNLLIFLCGAALLAVGIWVSIDGASFLKIFGPLSSSAMQFVNVGYFL

IAAGVVVFALGFLGCYGAKTESKCALVTFFFILLLIFIAEVAAAVVALVYTTMAEHFLTL

LVVPAIKKDYGSQEDFTQVWNTTMKGLKCCGFTNYTDFEDSPYFKENSAFPPFCCNDNVT

NTANETCTKQKAHDQKVEGCFNQLLYDIRTNAVTVGGVAAGIGGLELAAMIVSMYLYCNL

Q

108.>sp|P09326|CD48_HUMANCD48antigenOS=HomosapiensGN=CD48PE=1SV=2

MCSRGWDSCLALELLLLPLSLLVTSIQGHLVHMTVVSGSNVTLNISESLPENYKQLTWFY

TFDQKIVEWDSRKSKYFESKFKGRVRLDPQSGALYISKVQKEDNSTYIMRVLKKTGNEQE

WKIKLQVLDPVPKPVIKIEKIEDMDDNCYLKLSCVIPGESVNYTWYGDKRPFPKELQNSV

LETTLMPHNYSRCYTCQVSNSVSSKNGTVCLSPPCTLARSFGVEWIASWLVVTVPTILGL

LLT

109.>sp|P02745|C1QA_HUMANComplementC1qsubcomponentsubunitAOS=HomosapiensGN=C1QAPE=1SV=2

MEGPRGWLVLCVLAISLASMVTEDLCRAPDGKKGEAGRPGRRGRPGLKGEQGEPGAPGIR

TGIQGLKGDQGEPGPSGNPGKVGYPGPSGPLGARGIPGIKGTKGSPGNIKDQPRPAFSAI

RRNPPMGGNVVIFDTVITNQEEPYQNHSGRFVCTVPGYYYFTFQVLSQWEICLSIVSSSR

GQVRRSLGFCDTTNKGLFQVVSGGMVLQLQQGDQVWVEKDPKKGHIYQGSEADSVFSGFL

IFPSA

110.>sp|P23560|BDNF_HUMANBrain-derivedneurotrophicfactorOS=HomosapiensGN=BDNFPE=1SV=1

MTILFLTMVISYFGCMKAAPMKEANIRGQGGLAYPGVRTHGTLESVNGPKAGSRGLTSLA

DTFEHVIEELLDEDQKVRPNEENNKDADLYTSRVMLSSQVPLEPPLLFLLEEYKNYLDAA

NMSMRVRRHSDPARRGELSVCDSISEWVTAADKKTAVDMSGGTVTVLEKVPVSKGQLKQY

FYETKCNPMGYTKEGCRGIDKRHWNSQCRTTQSYVRALTMDSKKRIGWRFIRIDTSCVCT

LTIKRGR

111.>sp|P23946|CMA1_HUMANChymaseOS=HomosapiensGN=CMA1PE=1SV=1

MLLLPLPLLLFLLCSRAEAGEIIGGTECKPHSRPYMAYLEIVTSNGPSKFCGGFLIRRNF

VLTAAHCAGRSITVTLGAHNITEEEDTWQKLEVIKQFRHPKYNTSTLHHDIMLLKLKEKA

SLTLAVGTLPFPSQFNFVPPGRMCRVAGWGRTGVLKPGSDTLQEVKLRLMDPQACSHFRD

FDHNLQLCVGNPRKTKSAFKGDSGGPLLCAGVAQGIVSYGRSDAKPPAVFTRISHYRPWI

NQILQAN

112.>sp|P10144|GRAB_HUMANGranzymeBOS=HomosapiensGN=GZMBPE=1SV=2

MQPILLLLAFLLLPRADAGEIIGGHEAKPHSRPYMAYLMIWDQKSLKRCGGFLIRDDFVL

TAAHCWGSSINVTLGAHNIKEQEPTQQFIPVKRPIPHPAYNPKNFSNDIMLLQLERKAKR

TRAVQPLRLPSNKAQVKPGQTCSVAGWGQTAPLGKHSHTLQEVKMTVQEDRKCESDLRHY

YDSTIELCVGDPEIKKTSFKGDSGGPLVCNKVAQGIVSYGRNNGMPPRACTKVSSFVHWI

KKTMKRY

113.>sp|Q9UKR0|KLK12_HUMANKallikrein-12OS=HomosapiensGN=KLK12PE=1SV=1

MGLSIFLLLCVLGLSQAATPKIFNGTECGRNSQPWQVGLFEGTSLRCGGVLIDHRWVLTA

AHCSGSRYWVRLGEHSLSQLDWTEQIRHSGFSVTHPGYLGASTSHEHDLRLLRLRLPVRV

TSSVQPLPLPNDCATAGTECHVSGWGITNHPRNPFPDLLQCLNLSIVSHATCHGVYPGRI

TSNMVCAGGVPGQDACQGDSGGPLVCGGVLQGLVSWGSVGPCGQDGIPGVYTYICKYVDW

IRMIMRNN

114.>sp|Q96DR5|BPIA2_HUMANBPIfold-containingfamilyAmember2OS=HomosapiensGN=BPIFA2PE=1SV=2

MLQLWKLVLLCGVLTGTSESLLDNLGNDLSNVVDKLEPVLHEGLETVDNTLKGILEKLKV

DLGVLQKSSAWQLAKQKAQEAEKLLNNVISKLLPTNTDIFGLKISNSLILDVKAEPIDDG

KGLNLSFPVTANVTVAGPIIGQIINLKASLDLLTAVTIETDPQTHQPVAVLGECASDPTS

ISLSLLDKHSQIINKFVNSVINTLKSTVSSLLQKEICPLIRIFIHSLDVNVIQQVVDNPQ

HKTQLQTLI

115.>sp|0|CRIS1_HUMANCysteine-richsecretoryprotein1OS=HomosapiensGN=CRISP1PE=1SV=1

MEIKHLLFLVAAACLLPMLSMKKKSARDQFNKLVTDLPNVQEEIVNIHNALRRRVVPPAS

NMLKMSWSEEAAQNARIFSKYCDMTESNPLERRLPNTFCGENMHMTSYPVSWSSVIGVWY

SESTSFKHGEWTTTDDDITTDHYTQIVWATSYLIGCAIASCRQQGSPRYLYVCHYCHEGN

DPETKNEPYKTGVPCEACPSNCEDKLCTNPCIYYDEYFDCDIQVHYLGCNHSTTILFCKA

TCLCDTEIK

116.>sp|O43508|TNF12_HUMANTumornecrosisfactorligandsuperfamilymember12OS=HomosapiensGN=TNFSF12PE=1SV=1

MAARRSQRRRGRRGEPGTALLVPLALGLGLALACLGLLLAVVSLGSRASLSAQEPAQEEL

VAEEDQDPSELNPQTEESQDPAPFLNRLVRPRRSAPKGRKTRARRAIAAHYEVHPRPGQD

GAQAGVDGTVSGWEEARINSSSPLRYNRQIGEFIVTRAGLYYLYCQVHFDEGKAVYLKLD

LLVDGVLALRCLEEFSATAASSLGPQLRLCQVSGLLALRPGSSLRIRTLPWAHLKAAPFL

TYFGLFQVH

117.>sp|P19256|LFA3_HUMANLymphocytefunction-associatedantigen3OS=HomosapiensGN=CD58PE=1SV=1

MVAGSDAGRALGVLSVVCLLHCFGFISCFSQQIYGVVYGNVTFHVPSNVPLKEVLWKKQK

DKVAELENSEFRAFSSFKNRVYLDTVSGSLTIYNLTSSDEDEYEMESPNITDTMKFFLYV

LESLPSPTLTCALTNGSIEVQCMIPEHYNSHRGLIMYSWDCPMEQCKRNSTSIYFKMEND

LPQKIQCTLSNPLFNTTSSIILTTCIPSSGHSRHRYALIPIPLAVITTCIVLYMNGILKC

DRKPDRTNSN

118.>sp|P20160|CAP7_HUMANAzurocidinOS=HomosapiensGN=AZU1PE=1SV=3

MTRLTVLALLAGLLASSRAGSSPLLDIVGGRKARPRQFPFLASIQNQGRHFCGGALIHAR

FVMTAASCFQSQNPGVSTVVLGAYDLRRRERQSRQTFSISSMSENGYDPQQNLNDLMLLQ

LDREANLTSSVTILPLPLQNATVEAGTRCQVAGWGSQRSGGRLSRFPRFVNVTVTPEDQC

RPNNVCTGVLTRRGGICNGDGGTPLVCEGLAHGVASFSLGPCGRGPDFFTRVALFRDWID

GVLNNPGPGPA

119.>sp|P15514|AREG_HUMANAmphiregulinOS=HomosapiensGN=AREGPE=1SV=2

MRAPLLPPAPVVLSLLILGSGHYAAGLDLNDTYSGKREPFSGDHSADGFEVTSRSEMSSG

SEISPVSEMPSSSEPSSGADYDYSEEYDNEPQIPGYIVDDSVRVEQVVKPPQNKTESENT

SDKPKRKKKGGKNGKNRRNRKKKNPCNAEFQNFCIHGECKYIEHLEAVTCKCQQEYFGER

CGEKSMKTHSMIDSSLSKIALAAIAAFMSAVILTAVAVITVQLRRQYVRKYEGEAEERKK

LRQENGNVHAIA

120.>sp|P20851|C4BPB_HUMANC4b-bindingproteinbetachainOS=HomosapiensGN=C4BPBPE=1SV=1

MFFWCACCLMVAWRVSASDAEHCPELPPVDNSIFVAKEVEGQILGTYVCIKGYHLVGKKT

LFCNASKEWDNTTTECRLGHCPDPVLVNGEFSSSGPVNVSDKITFMCNDHYILKGSNRSQ

CLEDHTWAPPFPICKSRDCDPPGNPVHGYFEGNNFTLGSTISYYCEDRYYLVGVQEQQCV

DGEWSSALPVCKLIQEAPKPECEKALLAFQESKNLCEAMENFMQQLKESGMTMEELKYSL

ELKKAELKAKLL

121.>sp|O43291|SPIT2_HUMANKunitz-typeproteaseinhibitor2OS=HomosapiensGN=SPINT2PE=1SV=2

MAQLCGLRRSRAFLALLGSLLLSGVLAADRERSIHDFCLVSKVVGRCRASMPRWWYNVTD

GSCQLFVYGGCDGNSNNYLTKEECLKKCATVTENATGDLATSRNAADSSVPSAPRRQDSE

DHSSDMFNYEEYCTANAVTGPCRASFPRWYFDVERNSCNNFIYGGCRGNKNSYRSEEACM

LRCFRQQENPPLPLGSKVVVLAGLFVMVLILFLGASMVYLIRVARRNQERALRTVWSSGD

DKEQLVKNTYVL

122.>sp|Q9NY35|CLDN1_HUMANClaudindomain-containingprotein1OS=HomosapiensGN=CLDND1PE=1SV=1

MDNRFATAFVIACVLSLISTIYMAASIGTDFWYEYRSPVQENSSDLNKSIWDEFISDEAD

EKTYNDALFRYNGTVGLWRRCITIPKNMHWYSPPERTESFDVVTKCVSFTLTEQFMEKFV

DPGNHNSGIDLLRTYLWRCQFLLPFVSLGLMCFGALIGLCACICRSLYPTIATGILHLLA

GLCTLGSVSCYVAGIELLHQKLELPDNVSGEFGWSFCLACVSAPLQFMASALFIWAAHTN

RKEYTLMKAYRVA

123.>sp|P04156|PRIO_HUMANMajorprionproteinOS=HomosapiensGN=PRNPPE=1SV=1

MANLGCWMLVLFVATWSDLGLCKKRPKPGGWNTGGSRYPGQGSPGGNRYPPQGGGGWGQP

HGGGWGQPHGGGWGQPHGGGWGQPHGGGWGQGGGTHSQWNKPSKPKTNMKHMAGAAAAGA

VVGGLGGYMLGSAMSRPIIHFGSDYEDRYYRENMHRYPNQVYYRPMDEYSNQNNFVHDCV

NITIKQHTVTTTTKGENFTETDVKMMERVVEQMCITQYERESQAYYQRGSSMVLFSSPPV

ILLISFLIFLIVG

124.>sp|P01903|DRA_HUMANHLAclassIIhistocompatibilityantigen,DRalphachainOS=HomosapiensGN=HLA-DRAPE=1SV=1

MAISGVPVLGFFIIAVLMSAQESWAIKEEHVIIQAEFYLNPDQSGEFMFDFDGDEIFHVD

MAKKETVWRLEEFGRFASFEAQGALANIAVDKANLEIMTKRSNYTPITNVPPEVTVLTNS

PVELREPNVLICFIDKFTPPVVNVTWLRNGKPVTTGVSETVFLPREDHLFRKFHYLPFLP

STEDVYDCRVEHWGLDEPLLKHWEFDAPSPLPETTENVVCALGLTVGLVGIIIGTIFIIK

GVRKSNAAERRGPL

125.>sp|P08637|FCG3A_HUMANLowaffinityimmunoglobulingammaFcregionreceptorIII-AOS=HomosapiensGN=FCGR3APE=1SV=2

MWQLLLPTALLLLVSAGMRTEDLPKAVVFLEPQWYRVLEKDSVTLKCQGAYSPEDNSTQW

FHNESLISSQASSYFIDAATVDDSGEYRCQTNLSTLSDPVQLEVHIGWLLLQAPRWVFKE

EDPIHLRCHSWKNTALHKVTYLQNGKGRKYFHHNSDFYIPKATLKDSGSYFCRGLFGSKN

VSSETVNITITQGLAVSTISSFFPPGYQVSFCLVMVLLFAVDTGLYFSVKTNIRSSTRDW

KDHKFKWRKDPQDK

126.>sp|P08311|CATG_HUMANCathepsinGOS=HomosapiensGN=CTSGPE=1SV=2

MQPLLLLLAFLLPTGAEAGEIIGGRESRPHSRPYMAYLQIQSPAGQSRCGGFLVREDFVL

TAAHCWGSNINVTLGAHNIQRRENTQQHITARRAIRHPQYNQRTIQNDIMLLQLSRRVRR

NRNVNPVALPRAQEGLRPGTLCTVAGWGRVSMRRGTDTLREVQLRVQRDRQCLRIFGSYD

PRRQICVGDRRERKAAFKGDSGGPLLCNNVAHGIVSYGKSSGVPPEVFTRVSSFLPWIRT

TMRSFKLLDQMETPL

127.>sp|P14207|FOLR2_HUMANFolatereceptorbetaOS=HomosapiensGN=FOLR2PE=1SV=4

MVWKWMPLLLLLVCVATMCSAQDRTDLLNVCMDAKHHKTKPGPEDKLHDQCSPWKKNACC

TASTSQELHKDTSRLYNFNWDHCGKMEPACKRHFIQDTCLYECSPNLGPWIQQVNQSWRK

ERFLDVPLCKEDCQRWWEDCHTSHTCKSNWHRGWDWTSGVNKCPAGALCRTFESYFPTPA

ALCEGLWSHSYKVSNYSRGSGRCIQMWFDSAQGNPNEEVARFYAAAMHVNAGEMLHGTGG

LLLSLALMLQLWLLG

128.>sp|P55083|MFAP4_HUMANMicrofibril-associatedglycoprotein4OS=HomosapiensGN=MFAP4PE=1SV=2

MKALLALPLLLLLSTPPCAPQVSGIRGDALERFCLQQPLDCDDIYAQGYQSDGVYLIYPS

GPSVPVPVFCDMTTEGGKWTVFQKRFNGSVSFFRGWNDYKLGFGRADGEYWLGLQNMHLL

TLKQKYELRVDLEDFENNTAYAKYADFSISPNAVSAEEDGYTLFVAGFEDGGAGDSLSYH

SGQKFSTFDRDQDLFVQNCAALSSGAFWFRSCHFANLNGFYLGGSHLSYANGINWAQWKG

FYYSLKRTEMKIRRA

129.>sp|P24158|PRTN3_HUMANMyeloblastinOS=HomosapiensGN=PRTN3PE=1SV=3

MAHRPPSPALASVLLALLLSGAARAAEIVGGHEAQPHSRPYMASLQMRGNPGSHFCGGTL

IHPSFVLTAAHCLRDIPQRLVNVVLGAHNVRTQEPTQQHFSVAQVFLNNYDAENKLNDVL

LIQLSSPANLSASVATVQLPQQDQPVPHGTQCLAMGWGRVGAHDPPAQVLQELNVTVVTF

FCRPHNICTFVPRRKAGICFGDSGGPLICDGIIQGIDSFVIWGCATRLFPDFFTRVALYV

DWIRSTLRRVEAKGRP

130.>sp|O00584|RNT2_HUMANRibonucleaseT2OS=HomosapiensGN=RNASET2PE=1SV=2

MRPAALRGALLGCLCLALLCLGGADKRLRDNHEWKKLIMVQHWPETVCEKIQNDCRDPPD

YWTIHGLWPDKSEGCNRSWPFNLEEIKDLLPEMRAYWPDVIHSFPNRSRFWKHEWEKHGT

CAAQVDALNSQKKYFGRSLELYRELDLNSVLLKLGIKPSINYYQVADFKDALARVYGVIP

KIQCLPPSQDEEVQTIGQIELCLTKQDQQLQNCTEPGEQPSPKQEVWLANGAAESRGLRV

CEDGPVFYPPPKKTKH(LastNoutsideD.R)

131.>sp|P12319|FCERA_HUMANHighaffinityimmunoglobulinepsilonreceptorsubunitalphaOS=HomosapiensGN=FCER1APE=1SV=1

MAPAMESPTLLCVALLFFAPDGVLAVPQKPKVSLNPPWNRIFKGENVTLTCNGNNFFEVS

STKWFHNGSLSEETNSSLNIVNAKFEDSGEYKCQHQQVNESEPVYLEVFSDWLLLQASAE

VVMEGQPLFLRCHGWRNWDVYKVIYYKDGEALKYWYENHNISITNATVEDSGTYYCTGKV

WQLDYESEPLNITVIKAPREKYWLQFFIPLLVVILFAVDTGLFISTQQQVTFLLKIKRTR

KGFRLLNPHPKPNPKNN

132.>sp|P15328|FOLR1_HUMANFolatereceptoralphaOS=HomosapiensGN=FOLR1PE=1SV=3

MAQRMTTQLLLLLVWVAVVGEAQTRIAWARTELLNVCMNAKHHKEKPGPEDKLHEQCRPW

RKNACCSTNTSQEAHKDVSYLYRFNWNHCGEMAPACKRHFIQDTCLYECSPNLGPWIQQV

DQSWRKERVLNVPLCKEDCEQWWEDCRTSYTCKSNWHKGWNWTSGFNKCAVGAACQPFHF

YFPTPTVLCNEIWTHSYKVSNYSRGSGRCIQMWFDPAQGNPNEEVARFYAAAMSGAGPWA

AWPFLLSLALMLLWLLS

133.>sp|P07738|PMGE_HUMANBisphosphoglyceratemutaseOS=HomosapiensGN=BPGMPE=1SV=2

MSKYKLIMLRHGEGAWNKENRFCSWVDQKLNSEGMEEARNCGKQLKALNFEFDLVFTSVL

NRSIHTAWLILEELGQEWVPVESSWRLNERHYGALIGLNREQMALNHGEEQVRLWRRSYN

VTPPPIEESHPYYQEIYNDRRYKVCDVPLDQLPRSESLKDVLERLLPYWNERIAPEVLRG

KTILISAHGNSSRALLKHLEGISDEDIINITLPTGVPILLELDENLRAVGPHQFLGDQEA

IQAAIKKVEDQGKVKQAKK

134.>sp|Q16563|SYPL1_HUMANSynaptophysin-likeprotein1OS=HomosapiensGN=SYPL1PE=1SV=1

MAPNIYLVRQRISRLGQRMSGFQINLNPLKEPLGFIKVLEWIASIFAFATCGGFKGQTEI

QVNCPPAVTENKTVTATFGYPFRLNEASFQPPPGVNICDVNWKDYVLIGDYSSSAQFYVT

FAVFVFLYCIAALLLYVGYTSLYLDSRKLPMIDFVVTLVATFLWLVSTSAWAKALTDIKI

ATGHNIIDELPPCKKKAVLCYFGSVTSMGSLNVSVIFGFLNMILWGGNAWFVYKETSLHS

PSNTSAPHSQGGIPPPTGI

135.>sp|P20036|DPA1_HUMANHLAclassIIhistocompatibilityantigen,DPalpha1chainOS=HomosapiensGN=HLA-DPA1PE=1SV=1

MRPEDRMFHIRAVILRALSLAFLLSLRGAGAIKADHVSTYAAFVQTHRPTGEFMFEFDED

EMFYVDLDKKETVWHLEEFGQAFSFEAQGGLANIAILNNNLNTLIQRSNHTQATNDPPEV

TVFPKEPVELGQPNTLICHIDKFFPPVLNVTWLCNGELVTEGVAESLFLPRTDYSFHKFH

YLTFVPSAEDFYDCRVEHWGLDQPLLKHWEAQEPIQMPETTETVLCALGLVLGLVGIIVG

TVLIIKSLRSGHDPRAQGTL

136.>sp|P29965|CD40L_HUMANCD40ligandOS=HomosapiensGN=CD40LGPE=1SV=1

MIETYNQTSPRSAATGLPISMKIFMYLLTVFLITQMIGSALFAVYLHRRLDKIEDERNLH

EDFVFMKTIQRCNTGERSLSLLNCEEIKSQFEGFVKDIMLNKEETKKENSFEMQKGDQNP

QIAAHVISEASSKTTSVLQWAEKGYYTMSNNLVTLENGKQLTVKRQGLYYIYAQVTFCSN

REASSQAPFIASLCLKSPGRFERILLRAANTHSSAKPCGQQSIHLGGVFELQPGASVFVN

VTDPSQVSHGTGFTSFGLLKL

137.>sp|P07288|KLK3_HUMANProstate-specificantigenOS=HomosapiensGN=KLK3PE=1SV=2

MWVPVVFLTLSVTWIGAAPLILSRIVGGWECEKHSQPWQVLVASRGRAVCGGVLVHPQWV

LTAAHCIRNKSVILLGRHSLFHPEDTGQVFQVSHSFPHPLYDMSLLKNRFLRPGDDSSHD

LMLLRLSEPAELTDAVKVMDLPTQEPALGTTCYASGWGSIEPEEFLTPKKLQCVDLHVIS

NDVCAQVHPQKVTKFMLCAGRWTGGKSTCSGDSGGPLVCNGVLQGITSWGSEPCALPERP

SLYTKVVHYRKWIKDTIVANP

138.>sp|Q5UCC4|EMC10_HUMANERmembraneproteincomplexsubunit10OS=HomosapiensGN=EMC10PE=1SV=1

MAAASAGATRLLLLLLMAVAAPSRARGSGCRAGTGARGAGAEGREGEACGTVGLLLEHSF

EIDDSANFRKRGSLLWNQQDGTLSLSQRQLSEEERGRLRDVAALNGLYRVRIPRRPGALD

GLEAGGYVSSFVPACSLVESHLSDQLTLHVDVAGNVVGVSVVTHPGGCRGHEVEDVDLEL

FNTSVQLQPPTTAPGPETAAFIERLEMEQAQKAKNPQEQKSFFAKYWMYIIPVVLFLMMS

GAPDTGGQGGGGGGGGGGGSGR

139.>sp|P12544|GRAA_HUMANGranzymeAOS=HomosapiensGN=GZMAPE=1SV=2

MRNSYRFLASSLSVVVSLLLIPEDVCEKIIGGNEVTPHSRPYMVLLSLDRKTICAGALIA

KDWVLTAAHCNLNKRSQVILGAHSITREEPTKQIMLVKKEFPYPCYDPATREGDLKLLQL

MEKAKINKYVTILHLPKKGDDVKPGTMCQVAGWGRTHNSASWSDTLREVNITIIDRKVCN

DRNHYNFNPVIGMNMVCAGSLRGGRDSCNGDSGSPLLCEGVFRGVTSFGLENKCGDPRGP

GVYILLSKKHLNWIIMTIKGAV

140.>sp|P06870|KLK1_HUMANKallikrein-1OS=HomosapiensGN=KLK1PE=1SV=2

MWFLVLCLALSLGGTGAAPPIQSRIVGGWECEQHSQPWQAALYHFSTFQCGGILVHRQWV

LTAAHCISDNYQLWLGRHNLFDDENTAQFVHVSESFPHPGFNMSLLENHTRQADEDYSHD

LMLLRLTEPADTITDAVKVVELPTEEPEVGSTCLASGWGSIEPENFSFPDDLQCVDLKIL

PNDECKKAHVQKVTDFMLCVGHLEGGKDTCVGDSGGPLMCDGVLQGVTSWGYVPCGTPNK

PSVAVRVLSYVKWIEDTIAENS(ThirdNisPartiallyglycosylated)

141.>sp|P28068|DMB_HUMANHLAclassIIhistocompatibilityantigen,DMbetachainOS=HomosapiensGN=HLA-DMBPE=1SV=1

MITFLPLLLGLSLGCTGAGGFVAHVESTCLLDDAGTPKDFTYCISFNKDLLTCWDPEENK

MAPCEFGVLNSLANVLSQHLNQKDTLMQRLRNGLQNCATHTQPFWGSLTNRTRPPSVQVA

KTTPFNTREPVMLACYVWGFYPAEVTITWRKNGKLVMPHSSAHKTAQPNGDWTYQTLSHL

ALTPSYGDTYTCVVEHIGAPEPILRDWTPGLSPMQTLKVSVSAVTLGLGLIIFSLGVISW

RRAGHSSYTPLPGSNYSEGWHIS(NinD.R)

142.>sp|O95633|FSTL3_HUMANFollistatin-relatedprotein3OS=HomosapiensGN=FSTL3PE=1SV=1

MRPGAPGPLWPLPWGALAWAVGFVSSMGSGNPAPGGVCWLQQGQEATCSLVLQTDVTRAE

CCASGNIDTAWSNLTHPGNKINLLGFLGLVHCLPCKDSCDGVECGPGKACRMLGGRPRCE

CAPDCSGLPARLQVCGSDGATYRDECELRAARCRGHPDLSVMYRGRCRKSCEHVVCPRPQ

SCVVDQTGSAHCVVCRAAPCPVPSSPGQELCGNNNVTYISSCHMRQATCFLGRSIGVRHA

GSCAGTPEEPPGGESAEEEENFV

143.>sp|Q96FE7|P3IP1_HUMANPhosphoinositide-3-kinase-interactingprotein1OS=HomosapiensGN=PIK3IP1PE=1SV=2

MLLAWVQAFLVSNMLLAEAYGSGGCFWDNGHLYREDQTSPAPGLRCLNWLDAQSGLASAP

VSGAGNHSYCRNPDEDPRGPWCYVSGEAGVPEKRPCEDLRCPETTSQALPAFTTEIQEAS

EGPGADEVQVFAPANALPARSEAAAVQPVIGISQRVRMNSKEKKDLGTLGYVLGITMMVI

IIAIGAGIILGYSYKRGKDLKEQHDQKVCEREMQRITLPLSAFTNPTCEIVDEKTVVVHT

SQTPVDPQEGTTPLMGQAGTPGA

144.>sp|Q5QGZ9|CL12A_HUMANC-typelectindomainfamily12memberAOS=HomosapiensGN=CLEC12APE=1SV=3

MSEEVTYADLQFQNSSEMEKIPEIGKFGEKAPPAPSHVWRPAALFLTLLCLLLLIGLGVL

ASMFHVTLKIEMKKMNKLQNISEELQRNISLQLMSNMNISNKIRNLSTTLQTIATKLCRE

LYSKEQEHKCKPCPRRWIWHKDSCYFLSDDVQTWQESKMACAAQNASLLKINNKNALEFI

KSQSRSYDYWLGLSPEEDSTRGMRVDNIINSSAWVIRNAPDLNNMYCGYINRLYVQYYHC

TYKKRMICEKMANPVQLGSTYFREA

145.>sp|P01912|2B13_HUMANHLAclassIIhistocompatibilityantigen,DRB1-3chainOS=HomosapiensGN=HLA-DRB1PE=1SV=2

MVCLRLPGGSCMAVLTVTLMVLSSPLALAGDTRPRFLEYSTSECHFFNGTERVRYLDRYF

HNQEENVRFDSDVGEFRAVTELGRPDAEYWNSQKDLLEQKRGRVDNYCRHNYGVVESFTV

QRRVHPKVTVYPSKTQPLQHHNLLVCSVSGFYPGSIEVRWFRNGQEEKTGVVSTGLIHNG

DWTFQTLVMLETVPRSGEVYTCQVEHPSVTSPLTVEWRARSESAQSKMLSGVGGFVLGLL

FLGAGLFIYFRNQKGHSGLQPRGFLS

146.>sp|P01911|2B1F_HUMANHLAclassIIhistocompatibilityantigen,DRB1-15betachainOS=HomosapiensGN=HLA-DRB1PE=1SV=2

MVCLKLPGGSCMTALTVTLMVLSSPLALSGDTRPRFLWQPKRECHFFNGTERVRFLDRYF

YNQEESVRFDSDVGEFRAVTELGRPDAEYWNSQKDILEQARAAVDTYCRHNYGVVESFTV

QRRVQPKVTVYPSKTQPLQHHNLLVCSVSGFYPGSIEVRWFLNGQEEKAGMVSTGLIQNG

DWTFQTLVMLETVPRSGEVYTCQVEHPSVTSPLTVEWRARSESAQSKMLSGVGGFVLGLL

FLGAGLFIYFRNQKGHSGLQPTGFLS

147.>sp|O94907|DKK1_HUMANDickkopf-relatedprotein1OS=HomosapiensGN=DKK1PE=1SV=1

MMALGAAGATRVFVAMVAAALGGHPLLGVSATLNSVLNSNAIKNLPPPLGGAAGHPGSAV

SAAPGILYPGGNKYQTIDNYQPYPCAEDEECGTDEYCASPTRGGDAGVQICLACRKRRKR

CMRHAMCCPGNYCKNGICVSSDQNHFRGEIEETITESFGNDHSTLDGYSRRTTLSSKMYH

TKGQEGSVCLRSSDCASGLCCARHFWSKICKPVLKEGQVCTKHRRKGSHGLEIFQRCYCG

EGLSCRIQKDHHQASNSSRLHTCQRH

148.>sp|P79483|DRB3_HUMANHLAclassIIhistocompatibilityantigen,DRbeta3chainOS=HomosapiensGN=HLA-DRB3PE=1SV=1

MVCLKLPGGSSLAALTVTLMVLSSRLAFAGDTRPRFLELRKSECHFFNGTERVRYLDRYF

HNQEEFLRFDSDVGEYRAVTELGRPVAESWNSQKDLLEQKRGRVDNYCRHNYGVGESFTV

QRRVHPQVTVYPAKTQPLQHHNLLVCSVSGFYPGSIEVRWFRNGQEEKAGVVSTGLIQNG

DWTFQTLVMLETVPRSGEVYTCQVEHPSVTSALTVEWRARSESAQSKMLSGVGGFVLGLL

FLGAGLFIYFRNQKGHSGLQPTGFLS

149.>sp|Q30154|DRB5_HUMANHLAclassIIhistocompatibilityantigen,DRbeta5chainOS=HomosapiensGN=HLA-DRB5PE=1SV=1

MVCLKLPGGSYMAKLTVTLMVLSSPLALAGDTRPRFLQQDKYECHFFNGTERVRFLHRDI

YNQEEDLRFDSDVGEYRAVTELGRPDAEYWNSQKDFLEDRRAAVDTYCRHNYGVGESFTV

QRRVEPKVTVYPARTQTLQHHNLLVCSVNGFYPGSIEVRWFRNSQEEKAGVVSTGLIQNG

DWTFQTLVMLETVPRSGEVYTCQVEHPSVTSPLTVEWRAQSESAQSKMLSGVGGFVLGLL

FLGAGLFIYFKNQKGHSGLHPTGLVS

150.>sp|Q9Y287|ITM2B_HUMANIntegralmembraneprotein2BOS=HomosapiensGN=ITM2BPE=1SV=1

MVKVTFNSALAQKEAKKDEPKSGEEALIIPPDAVAVDCKDPDDVVPVGQRRAWCWCMCFG

LAFMLAGVILGGAYLYKYFALQPDDVYYCGIKYIKDDVILNEPSADAPAALYQTIEENIK

IFEEEEVEFISVPVPEFADSDPANIVHDFNKKLTAYLDLNLDKCYVIPLNTSIVMPPRNL

LELLINIKAGTYLPQSYLIHEHMVITDRIENIDHLGFFIYRLCHDKETYKLQRRETIKGI

QKREASNCFAIRHFENKFAVETLICS

151.>sp|P02647|APOA1_HUMANApolipoproteinA-IOS=HomosapiensGN=APOA1PE=1SV=1

MKAAVLTLAVLFLTGSQARHFWQQDEPPQSPWDRVKDLATVYVDVLKDSGRDYVSQFEGS

ALGKQLNLKLLDNWDSVTSTFSKLREQLGPVTQEFWDNLEKETEGLRQEMSKDLEEVKAK

VQPYLDDFQKKWQEEMELYRQKVEPLRAELQEGARQKLHELQEKLSPLGEEMRDRARAHV

DALRTHLAPYSDELRQRLAARLEALKENGGARLAEYHAKATEHLSTLSEKAKPALEDLRQ

GLLPVLESFKVSFLSALEEYTKKLNTQ

152.>sp|P27701|CD82_HUMANCD82antigenOS=HomosapiensGN=CD82PE=1SV=1

MGSACIKVTKYFLFLFNLIFFILGAVILGFGVWILADKSSFISVLQTSSSSLRMGAYVFI

GVGAVTMLMGFLGCIGAVNEVRCLLGLYFAFLLLILIAQVTAGALFYFNMGKLKQEMGGI

VTELIRDYNSSREDSLQDAWDYVQAQVKCCGWVSFYNWTDNAELMNRPEVTYPCSCEVKG

EEDNSLSVRKGFCEAPGNRTQSGNHPEDWPVYQEGCMEKVQAWLQENLGIILGVGVGVAI

IELLGMVLSICLCRHVHSEDYSKVPKY

153.>sp|P01189|COLI_HUMANPro-opiomelanocortinOS=HomosapiensGN=POMCPE=1SV=2

MPRSCCSRSGALLLALLLQASMEVRGWCLESSQCQDLTTESNLLECIRACKPDLSAETPM

FPGNGDEQPLTENPRKYVMGHFRWDRFGRRNSSSSGSSGAGQKREDVSAGEDCGPLPEGG

PEPRSDGAKPGPREGKRSYSMEHFRWGKPVGKKRRPVKVYPNGAEDESAEAFPLEFKREL

TGQRLREGDGPDGPADDGAGAQADLEHSLLVAAEKKDEGPYRMEHFRWGSPPKDKRYGGF

MTSEKSQTPLVTLFKNAIIKNAYKKGE

154.>sp|P08246|ELNE_HUMANNeutrophilelastaseOS=HomosapiensGN=ELANEPE=1SV=1

MTLGRRLACLFLACVLPALLLGGTALASEIVGGRRARPHAWPFMVSLQLRGGHFCGATLI

APNFVMSAAHCVANVNVRAVRVVLGAHNLSRREPTRQVFAVQRIFENGYDPVNLLNDIVI

LQLNGSATINANVQVAQLPAQGRRLGNGVQCLAMGWGLLGRNRGIASVLQELNVTVVTSL

CRRSNVCTLVRGRQAGVCFGDSGSPLVCNGLIHGIASFVRGGCASGLYPDAFAPVAQFVN

WIDSIIQRSEDNPCPHPRDPDPASRTH

155.>sp|P29972|AQP1_HUMANAquaporin-1OS=HomosapiensGN=AQP1PE=1SV=3

MASEFKKKLFWRAVVAEFLATTLFVFISIGSALGFKYPVGNNQTAVQDNVKVSLAFGLSI

ATLAQSVGHISGAHLNPAVTLGLLLSCQISIFRALMYIIAQCVGAIVATAILSGITSSLT

GNSLGRNDLADGVNSGQGLGIEIIGTLQLVLCVLATTDRRRRDLGGSAPLAIGLSVALGH

LLAIDYTGCGINPARSFGSAVITHNFSNHWIFWVGPFIGGALAVLIYDFILAPRSSDLTD

RVKVWTSGQVEEYDLDADDINSRVEMKPK

156.>sp|O95297|MPZL1_HUMANMyelinproteinzero-likeprotein1OS=HomosapiensGN=MPZL1PE=1SV=1

MAASAGAGAVIAAPDSRRWLWSVLAAALGLLTAGVSALEVYTPKEIFVANGTQGKLTCKF

KSTSTTGGLTSVSWSFQPEGADTTVSFFHYSQGQVYLGNYPPFKDRISWAGDLDKKDASI

NIENMQFIHNGTYICDVKNPPDIVVQPGHIRLYVVEKENLPVFPVWVVVGIVTAVVLGLT

LLISMILAVLYRRKNSKRDYTGCSTSESLSPVKQAPRKSPSDTEGLVKSLPSGSHQGPVI

YAQLDHSGGHHSDKINKSESVVYADIRKN

157.>sp|P08861|CEL3B_HUMANChymotrypsin-likeelastasefamilymember3BOS=HomosapiensGN=CELA3BPE=1SV=3

MMLRLLSSLLLVAVASGYGPPSSRPSSRVVNGEDAVPYSWPWQVSLQYEKSGSFYHTCGG

SLIAPDWVVTAGHCISSSRTYQVVLGEYDRAVKEGPEQVIPINSGDLFVHPLWNRSCVAC

GNDIALIKLSRSAQLGDAVQLASLPPAGDILPNETPCYITGWGRLYTNGPLPDKLQEALL

PVVDYEHCSRWNWWGSSVKKTMVCAGGDIRSGCNGDSGGPLNCPTEDGGWQVHGVTSFVS

AFGCNTRRKPTVFTRVSAFIDWIEETIASH

158.>sp|P36980|FHR2_HUMANComplementfactorH-relatedprotein2OS=HomosapiensGN=CFHR2PE=1SV=1

MWLLVSVILISRISSVGGEAMFCDFPKINHGILYDEEKYKPFSQVPTGEVFYYSCEYNFV

SPSKSFWTRITCAEEGWSPTPKCLRLCFFPFVENGHSESSGQTHLEGDTVQIICNTGYRL

QNNENNISCVERGWSTPPKCRSTISAEKCGPPPPIDNGDITSFLLSVYAPGSSVEYQCQN

LYQLEGNNQITCRNGQWSEPPKCLDPCVISQEIMEKYNIKLKWTNQQKLYSRTGDIVEFV

CKSGYHPTKSHSFRAMCQNGKLVYPSCEEK

159.>sp|P01589|IL2RA_HUMANInterleukin-2receptorsubunitalphaOS=HomosapiensGN=IL2RAPE=1SV=1

MDSYLLMWGLLTFIMVPGCQAELCDDDPPEIPHATFKAMAYKEGTMLNCECKRGFRRIKS

GSLYMLCTGNSSHSSWDNQCQCTSSATRNTTKQVTPQPEEQKERKTTEMQSPMQPVDQAS

LPGHCREPPPWENEATERIYHFVVGQMVYYQCVQGYRALHRGPAESVCKMTHGKTRWTQP

QLICTGEMETSQFPGEEKPQASPEGRPESETSCLVTTTDFQIQTEMAATMETSIFTTEYQ

VAVAGCVFLLISVLLLSGLTWQRRQRKSRRTI

160.>sp|P78380|OLR1_HUMANOxidizedlow-densitylipoproteinreceptor1OS=HomosapiensGN=OLR1PE=1SV=1

MTFDDLKIQTVKDQPDEKSNGKKAKGLQFLYSPWWCLAAATLGVLCLGLVVTIMVLGMQL

SQVSDLLTQEQANLTHQKKKLEGQISARQQAEEASQESENELKEMIETLARKLNEKSKEQ

MELHHQNLNLQETLKRVANCSAPCPQDWIWHGENCYLFSSGSFNWEKSQEKCLSLDAKLL

KINSTADLDFIQQAISYSSFPFWMGLSRRNPSYPWLWEDGSPLMPHLFRVRGAVSQTYPS

GTCAYIQRGAVYAENCILAAFSICQKKANLRAQ

161.>sp|P21583|SCF_HUMANKitligandOS=HomosapiensGN=KITLGPE=1SV=1

MKKTQTWILTCIYLQLLLFNPLVKTEGICRNRVTNNVKDVTKLVANLPKDYMITLKYVPG

MDVLPSHCWISEMVVQLSDSLTDLLDKFSNISEGLSNYSIIDKLVNIVDDLVECVKENSS

KDLKKSFKSPEPRLFTPEEFFRIFNRSIDAFKDFVVASETSDCVVSSTLSPEKDSRVSVT

KPFMLPPVAASSLRNDSSSSNRKAKNPPGDSSLHWAAMALPALFSLIIGFAFGALYWKKR

QPSLTRAVENIQINEEDNEISMLQEKEREFQEV(2^nd^NinD.R)

162.>sp|Q9NUM4|T106B_HUMANTransmembraneprotein106BOS=HomosapiensGN=TMEM106BPE=1SV=2

MGKSLSHLPLHSSKEDAYDGVTSENMRNGLVNSEVHNEDGRNGDVSQFPYVEFTGRDSVT

CPTCQGTGRIPRGQENQLVALIPYSDQRLRPRRTKLYVMASVFVCLLLSGLAVFFLFPRS

IDVKYIGVKSAYVSYDVQKRTIYLNITNTLNITNNNYYSVEVENITAQVQFSKTVIGKAR

LNNITIIGPLDMKQIDYTVPTVIAEEMSYMYDFCTLISIKVHNIVLMMQVTVTTTYFGHS

EQISQERYQYVDCGRNTTYQLGQSEYLNVLQPQQ

163.>sp|P13598|ICAM2_HUMANIntercellularadhesionmolecule2OS=HomosapiensGN=ICAM2PE=1SV=2

MSSFGYRTLTVALFTLICCPGSDEKVFEVHVRPKKLAVEPKGSLEVNCSTTCNQPEVGGL

ETSLDKILLDEQAQWKHYLVSNISHDTVLQCHFTCSGKQESMNSNVSVYQPPRQVILTLQ

PTLVAVGKSFTIECRVPTVEPLDSLTLFLFRGNETLHYETFGKAAPAPQEATATFNSTAD

REDGHRNFSCLAVLDLMSRGGNIFHKHSAPKMLEIYEPVSDSQMVIIVTVVSVLLSLFVT

SVLLCFIFGQHLRQQRMGTYGVRAAWRRLPQAFRP

164.>sp|Q15661|TRYB1_HUMANTryptasealpha/beta-1OS=HomosapiensGN=TPSAB1PE=1SV=1

MLNLLLLALPVLASRAYAAPAPGQALQRVGIVGGQEAPRSKWPWQVSLRVHGPYWMHFCG

GSLIHPQWVLTAAHCVGPDVKDLAALRVQLREQHLYYQDQLLPVSRIIVHPQFYTAQIGA

DIALLELEEPVNVSSHVHTVTLPPASETFPPGMPCWVTGWGDVDNDERLPPPFPLKQVKV

PIMENHICDAKYHLGAYTGDDVRIVRDDMLCAGNTRRDSCQGDSGGPLVCKVNGTWLQAG

VVSWGEGCAQPNRPGIYTRVTYYLDWIHHYVPKKP

165.>sp|P20645|MPRD_HUMANCation-dependentmannose-6-phosphatereceptorOS=HomosapiensGN=M6PRPE=1SV=1

MFPFYSCWRTGLLLLLLAVAVRESWQTEEKTCDLVGEKGKESEKELALVKRLKPLFNKSF

ESTVGQGSDTYIYIFRVCREAGNHTSGAGLVQINKSNGKETVVGRLNETHIFNGSNWIML

IYKGGDEYDNHCGKEQRRAVVMISCNRHTLADNFNPVSEERGKVQDCFYLFEMDSSLACS

PEISHLSVGSILLVTFASLVAVYVVGGFLYQRLVVGAKGMEQFPHLAFWQDLGNLVADGC

DFVCRSKPRNVPAAYRGVGDDQLGEESEERDDHLLPM

166.>sp|Q9BT09|CNPY3_HUMANProteincanopyhomolog3OS=HomosapiensGN=CNPY3PE=1SV=1

MDSMPEPASRCLLLLPLLLLLLLLLPAPELGPSQAGAEENDWVRLPSKCEVCKYVAVELK

SAFEETGKTKEVIGTGYGILDQKASGVKYTKSDLRLIEVTETICKRLLDYSLHKERTGSN

RFAKGMSETFETLHNLVHKGVKVVMDIPYELWNETSAEVADLKKQCDVLVEEFEEVIEDW

YRNHQEEDLTEFLCANHVLKGKDTSCLAEQWSGKKGDTAALGGKKSKKKSSRAKAAGGRS

SSSKQRKELGGLEGDPSPEEDEGIQKASPLTHSPPDEL

167.>sp|P54709|AT1B3_HUMANSodium/potassium-transportingATPasesubunitbeta-3OS=HomosapiensGN=ATP1B3PE=1SV=1

MTKNEKKSLNQSLAEWKLFIYNPTTGEFLGRTAKSWGLILLFYLVFYGFLAALFSFTMWV

MLQTLNDEVPKYRDQIPSPGLMVFPKPVTALEYTFSRSDPTSYAGYIEDLKKFLKPYTLE

EQKNLTVCPDGALFEQKGPVYVACQFPISLLQACSGMNDPDFGYSQGNPCILVKMNRIIG

LKPEGVPRIDCVSKNEDIPNVAVYPHNGMIDLKYFPYYGKKLHVGYLQPLVAVQVSFAPN

NTGKEVTVECKIDGSANLKSQDDRDKFLGRVMFKITARA

168.>sp|Q6ISU1|PTCRA_HUMANPreT-cellantigenreceptoralphaOS=HomosapiensGN=PTCRAPE=1SV=1

MAGTWLLLLLALGCPALPTGVGGTPFPSLAPPIMLLVDGKQQMVVVCLVLDVAPPGLDSP

IWFSAGNGSALDAFTYGPSPATDGTWTNLAHLSLPSEELASWEPLVCHTGPGAEGHSRST

QPMHLSGEASTARTCPQEPLRGTPGGALWLGVLRLLLFKLLLFDLLLTCSCLCDPAGPLP

SPATTTRLRALGSHRLHPATETGGREATSSPRPQPRDRRWGDTPPGRKPGSPVWGEGSYL

SSYPTCPAQAWCSRSALRAPSSSLGAFFAGDLPPPLQAGAA

169.>sp|P24855|DNAS1_HUMANDeoxyribonuclease-1OS=HomosapiensGN=DNASE1PE=1SV=1

MRGMKLLGALLALAALLQGAVSLKIAAFNIQTFGETKMSNATLVSYIVQILSRYDIALVQ

EVRDSHLTAVGKLLDNLNQDAPDTYHYVVSEPLGRNSYKERYLFVYRPDQVSAVDSYYYD

DGCEPCGNDTFNREPAIVRFFSRFTEVREFAIVPLHAAPGDAVAEIDALYDVYLDVQEKW

GLEDVMLMGDFNAGCSYVRPSQWSSIRLWTSPTFQWLIPDSADTTATPTHCAYDRIVVAG

MLLRGAVVPDSALPFNFQAAYGLSDQLAQAISDHYPVEVMLK

170.>sp|Q92956|TNR14_HUMANTumornecrosisfactorreceptorsuperfamilymember14OS=HomosapiensGN=TNFRSF14PE=1SV=3

MEPPGDWGPPPWRSTPKTDVLRLVLYLTFLGAPCYAPALPSCKEDEYPVGSECCPKCSPG

YRVKEACGELTGTVCEPCPPGTYIAHLNGLSKCLQCQMCDPAMGLRASRNCSRTENAVCG

CSPGHFCIVQDGDHCAACRAYATSSPGQRVQKGGTESQDTLCQNCPPGTFSPNGTLEECQ

HQTKCSWLVTKAGAGTSSSHWVWWFLSGSLVIVIVCSTVGLIICVKRRKPRGDVVKVIVS

VQRKRQEAEGEATVIEALQAPPDVTTVAVEETIPSFTGRSPNH

171.>sp|Q9Y275|TN13B_HUMANTumornecrosisfactorligandsuperfamilymember13BOS=HomosapiensGN=TNFSF13BPE=1SV=1

MDDSTEREQSRLTSCLKKREEMKLKECVSILPRKESPSVRSSKDGKLLAATLLLALLSCC

LTVVSFYQVAALQGDLASLRAELQGHHAEKLPAGAGAPKAGLEEAPAVTAGLKIFEPPAP

GEGNSSQNSRNKRAVQGPEETVTQDCLQLIADSETPTIQKGSYTFVPWLLSFKRGSALEE

KENKILVKETGYFFIYGQVLYTDKTYAMGHLIQRKKVHVFGDELSLVTLFRCIQNMPETL

PNNSCYSAGIAKLEEGDELQLAIPRENAQISLDGDVTFFGALKLL

172.>sp|P43307|SSRA_HUMANTranslocon-associatedproteinsubunitalphaOS=HomosapiensGN=SSR1PE=1SV=3

MRLLPRLLLLLLLVFPATVLFRGGPRGLLAVAQDLTEDEETVEDSIIEDEDDEAEVEEDE

PTDLVEDKEEEDVSGEPEASPSADTTILFVKGEDFPANNIVKFLVGFTNKGTEDFIVESL

DASFRYPQDYQFYIQNFTALPLNTVVPPQRQATFEYSFIPAEPMGGRPFGLVINLNYKDL

NGNVFQDAVFNQTVTVIEREDGLDGETIFMYMFLAGLGLLVIVGLHQLLESRKRKRPIQK

VEMGTSSQNDVDMSWIPQETLNQINKASPRRLPRKRAQKRSVGSDE

173.>sp|P24071|FCAR_HUMANImmunoglobulinalphaFcreceptorOS=HomosapiensGN=FCARPE=1SV=1

MDPKQTTLLCLVLCLGQRIQAQEGDFPMPFISAKSSPVIPLDGSVKIQCQAIREAYLTQL

MIIKNSTYREIGRRLKFWNETDPEFVIDHMDANKAGRYQCQYRIGHYRFRYSDTLELVVT

GLYGKPFLSADRGLVLMPGENISLTCSSAHIPFDRFSLAKEGELSLPQHQSGEHPANFSL

GPVDLNVSGIYRCYGWYNRSPYLWSFPSNALELVVTDSIHQDYTTQNLIRMAVAGLVLVA

LLAILVENWHSHTALNKEASADVAEPSWSQQMCQPGLTFARTPSVCK

174.>sp|Q6GTX8|LAIR1_HUMANLeukocyte-associatedimmunoglobulin-likereceptor1OS=HomosapiensGN=LAIR1PE=1SV=1

MSPHPTALLGLVLCLAQTIHTQEEDLPRPSISAEPGTVIPLGSHVTFVCRGPVGVQTFRL

ERESRSTYNDTEDVSQASPSESEARFRIDSVSEGNAGPYRCIYYKPPKWSEQSDYLELLV

KETSGGPDSPDTEPGSSAGPTQRPSDNSHNEHAPASQGLKAEHLYILIGVSVVFLFCLLL

LVLFCLHRQNQIKQGPPRSKDEEQKPQQRPDLAVDVLERTADKATVNGLPEKDRETDTSA

LAAGSSQEVTYAQLDHWALTQRTARAVSPQSTKPMAESITYAAVARH

175.>sp|P33681|CD80_HUMANT-lymphocyteactivationantigenCD80OS=HomosapiensGN=CD80PE=1SV=1

MGHTRRQGTSPSKCPYLNFFQLLVLAGLSHFCSGVIHVTKEVKEVATLSCGHNVSVEELA

QTRIYWQKEKKMVLTMMSGDMNIWPEYKNRTIFDITNNLSIVILALRPSDEGTYECVVLK

YEKDAFKREHLAEVTLSVKADFPTPSISDFEIPTSNIRRIICSTSGGFPEPHLSWLENGE

ELNAINTTVSQDPETELYAVSSKLDFNMTTNHSFMCLIKYGHLRVNQTFNWNTTKQEHFP

DNLLPSWAITLISVNGIFVICCLTYCFAPRCRERRRNERLRRESVRPV

176.>sp|P14415|AT1B2_HUMANSodium/potassium-transportingATPasesubunitbeta-2OS=HomosapiensGN=ATP1B2PE=1SV=3

MVIQKEKKSCGQVVEEWKEFVWNPRTHQFMGRTGTSWAFILLFYLVFYGFLTAMFTLTMW

VMLQTVSDHTPKYQDRLATPGLMIRPKTENLDVIVNVSDTESWDQHVQKLNKFLEPYNDS

IQAQKNDVCRPGRYYEQPDNGVLNYPKRACQFNRTQLGNCSGIGDSTHYGYSTGQPCVFI

KMNRVINFYAGANQSMNVTCAGKRDEDAENLGNFVMFPANGNIDLMYFPYYGKKFHVNYT

QPLVAVKFLNVTPNVEVNVECRINAANIATDDERDKFAGRVAFKLRINKT

177.>sp|Q969X5|ERGI1_HUMANEndoplasmicreticulum-Golgiintermediatecompartmentprotein1OS=HomosapiensGN=ERGIC1PE=1SV=1

MPFDFRRFDIYRKVPKDLTQPTYTGAIISICCCLFILFLFLSELTGFITTEVVNELYVDD

PDKDSGGKIDVSLNISLPNLHCELVGLDIQDEMGRHEVGHIDNSMKIPLNNGAGCRFEGQ

FSINKVPGNFHVSTHSATAQPQNPDMTHVIHKLSFGDTLQVQNIHGAFNALGGADRLTSN

PLASHDYILKIVPTVYEDKSGKQRYSYQYTVANKEYVAYSHTGRIIPAIWFRYDLSPITV

KYTERRQPLYRFITTICAIIGGTFTVAGILDSCIFTASEAWKKIQLGKMH

178.>sp|Q9NZQ7|PD1L1_HUMANProgrammedcelldeath1ligand1OS=HomosapiensGN=CD274PE=1SV=1

MRIFAVFIFMTYWHLLNAFTVTVPKDLYVVEYGSNMTIECKFPVEKQLDLAALIVYWEME

DKNIIQFVHGEEDLKVQHSSYRQRARLLKDQLSLGNAALQITDVKLQDAGVYRCMISYGG

ADYKRITVKVNAPYNKINQRILVVDPVTSEHELTCQAEGYPKAEVIWTSSDHQVLSGKTT

TTNSKREEKLFNVTSTLRINTTTNEIFYCTFRRLDPEENHTAELVIPELPLAHPPNERTH

LVILGAILLCLGVALTFIFRLRKGRMMDVKKCGIQDTNSKKQSDTHLEET

179.>sp|P07306|ASGR1_HUMANAsialoglycoproteinreceptor1OS=HomosapiensGN=ASGR1PE=1SV=2

MTKEYQDLQHLDNEESDHHQLRKGPPPPQPLLQRLCSGPRLLLLSLGLSLLLLVVVCVIG

SQNSQLQEELRGLRETFSNFTASTEAQVKGLSTQGGNVGRKMKSLESQLEKQQKDLSEDH

SSLLLHVKQFVSDLRSLSCQMAALQGNGSERTCCPVNWVEHERSCYWFSRSGKAWADADN

YCRLEDAHLVVVTSWEEQKFVQHHIGPVNTWMGLHDQNGPWKWVDGTDYETGFKNWRPEQ

PDDWYGHGLGGGEDCAHFTDDGRWNDDVCQRPYRWVCETELDKASQEPPLL

180.>sp|A6NH11|GLTD2_HUMANGlycolipidtransferproteindomain-containingprotein2OS=HomosapiensGN=GLTPD2PE=1SV=2

MGVAARPPALRHWFSHSIPLAIFALLLLYLSVRSLGARSGCGPRAQPCVPGETAPFQVRQ

ESGTLEAPERKQPPCLGPRGMLGRMMRRFHASLKPEGDVGLSPYLAGWRALVEFLTPLGS

VFAFATREAFTKVTDLEARVHGPDAEHYWSLVAMAAWERRAGLLEQPGAAPRDPTRSSGS

RTLLLLHRALRWSQLCLHRVATGALGGPDAGVQCSDAYRAALGPHHPWLVRQTARLAFLA

FPGRRRLLELACPGATEAEARAALVRAAGTLEDVYNRTQSLLAERGLLQLA

181.>sp|P17936|IBP3_HUMANInsulin-likegrowthfactor-bindingprotein3OS=HomosapiensGN=IGFBP3PE=1SV=2

MQRARPTLWAAALTLLVLLRGPPVARAGASSAGLGPVVRCEPCDARALAQCAPPPAVCAE

LVREPGCGCCLTCALSEGQPCGIYTERCGSGLRCQPSPDEARPLQALLDGRGLCVNASAV

SRLRAYLLPAPPAPGNASESEEDRSAGSVESPSVSSTHRVSDPKFHPLHSKIIIIKKGHA

KDSQRYKVDYESQSTDTQNFSSESKRETEYGPCRREMEDTLNHLKFLNVLSPRGVHIPNC

DKKGFYKKKQCRPSKGRKRGFCWCVDKYGQPLPGYTTKGKEDVHCYSMQSK

182.>sp|P28845|DHI1_HUMANCorticosteroid11-beta-dehydrogenaseisozyme1OS=HomosapiensGN=HSD11B1PE=1SV=3

MAFMKKYLLPILGLFMAYYYYSANEEFRPEMLQGKKVIVTGASKGIGREMAYHLAKMGAH

VVVTARSKETLQKVVSHCLELGAASAHYIAGTMEDMTFAEQFVAQAGKLMGGLDMLILNH

ITNTSLNLFHDDIHHVRKSMEVNFLSYVVLTVAALPMLKQSNGSIVVVSSLAGKVAYPMV

AAYSASKFALDGFFSSIRKEYSVSRVNVSITLCVLGLIDTETAMKAVSGIVHMQAAPKEE

CALEIIKGGALRQEEVYYDSSLWTTLLIRNPCRKILEFLYSTSYNMDRFINK

183.>sp|Q9Y337|KLK5_HUMANKallikrein-5OS=HomosapiensGN=KLK5PE=1SV=2

MATARPPWMWVLCALITALLLGVTEHVLANNDVSCDHPSNTVPSGSNQDLGAGAGEDARS

DDSSSRIINGSDCDMHTQPWQAALLLRPNQLYCGAVLVHPQWLLTAAHCRKKVFRVRLGH

YSLSPVYESGQQMFQGVKSIPHPGYSHPGHSNNLMLIKLNRRIRPTKDVRPINVSSHCPS

AGTKCLVSGWGTTKSPQVHFPKVLQCLNISVLSQKRCEDAYPRQIDDTMFCAGDKAGRDS

CQGDSGGPVVCNGSLQGLVSWGDYPCARPNRPGVYTNLCKFTKWIQETIQANS

184.>sp|Q96E22|NGBR_HUMANDehydrodolichyldiphosphatesyntasecomplexsubunitNUS1OS=HomosapiensGN=NUS1PE=1SV=1

MTGLYELVWRVLHALLCLHRTLTSWLRVRFGTWNWIWRRCCRAASAAVLAPLGFTLRKPP

AVGRNRRHHRHPRGGSCLAAAHHRMRWRADGRSLEKLPVHMGLVITEVEQEPSFSDIASL

VVWCMAVGISYISVYDHQGIFKRNNSRLMDEILKQQQELLGLDCSKYSPEFANSNDKDDQ

VLNCHLAVKVLSPEDGKADIVRAAQDFCQLVAQKQKRPTDLDVDTLASLLSSNGCPDPDL

VLKFGPVDSTLGFLPWHIRLTEIVSLPSHLNISYEDFFSALRQYAACEQRLGK

185.>sp|P04233|HG2A_HUMANHLAclassIIhistocompatibilityantigengammachainOS=HomosapiensGN=CD74PE=1SV=3

MHRRRSRSCREDQKPVMDDQRDLISNNEQLPMLGRRPGAPESKCSRGALYTGFSILVTLL

LAGQATTAYFLYQQQGRLDKLTVTSQNLQLENLRMKLPKPPKPVSKMRMATPLLMQALPM

GALPQGPMQNATKYGNMTEDHVMHLLQNADPLKVYPPLKGSFPENLRHLKNTMETIDWKV

FESWMHHWLLFEMSRHSLEQKPTDAPPKVLTKCQEEVSHIPAVHPGSFRPKCDENGNYLP

LQCYGSIGYCWCVFPNGTEVPNTRSRGHHNCSESLELEDPSSGLGVTKQDLGPVPM

186.>sp|Q5SQ64|LY66F_HUMANLymphocyteantigen6complexlocusproteinG6fOS=HomosapiensGN=LY6G6FPE=1SV=2

MAVLFLLLFLCGTPQAADNMQAIYVALGEAVELPCPSPPTLHGDEHLSWFCSPAAGSFTT

LVAQVQVGRPAPDPGKPGRESRLRLLGNYSLWLEGSKEEDAGRYWCAVLGQHHNYQNWRV

YDVLVLKGSQLSARAADGSPCNVLLCSVVPSRRMDSVTWQEGKGPVRGRVQSFWGSEAAL

LLVCPGEGLSEPRSRRPRIIRCLMTHNKGVSFSLAASIDASPALCAPSTGWDMPWILMLL

LTMGQGVVILALSIVLWRQRVRGAPGRDASIPQFKPEIQVYENIHLARLGPPAHKPR

187.>sp|P57087|JAM2_HUMANJunctionaladhesionmoleculeBOS=HomosapiensGN=JAM2PE=1SV=1

MARRSRHRLLLLLLRYLVVALGYHKAYGFSAPKDQQVVTAVEYQEAILACKTPKKTVSSR

LEWKKLGRSVSFVYYQQTLQGDFKNRAEMIDFNIRIKNVTRSDAGKYRCEVSAPSEQGQN

LEEDTVTLEVLVAPAVPSCEVPSSALSGTVVELRCQDKEGNPAPEYTWFKDGIRLLENPR

LGSQSTNSSYTMNTKTGTLQFNTVSKLDTGEYSCEARNSVGYRRCPGKRMQVDDLNISGI

IAAVVVVALVISVCGLGVCYAQRKGYFSKETSFQKSNSSSKATTMSENDFKHTKSFII

188.>sp|P20774|MIME_HUMANMimecanOS=HomosapiensGN=OGNPE=1SV=1

MKTLQSTLLLLLLVPLIKPAPPTQQDSRIIYDYGTDNFEESIFSQDYEDKYLDGKNIKEK

ETVIIPNEKSLQLQKDEAITPLPPKKENDEMPTCLLCVCLSGSVYCEEVDIDAVPPLPKE

SAYLYARFNKIKKLTAKDFADIPNLRRLDFTGNLIEDIEDGTFSKLSLLEELSLAENQLL

KLPVLPPKLTLFNAKYNKIKSRGIKANAFKKLNNLTFLYLDHNALESVPLNLPESLRVIH

LQFNNIASITDDTFCKANDTSYIRDRIEEIRLEGNPIVLGKHPNSFICLKRLPIGSYF

189.>sp|P25311|ZA2G_HUMANZinc-alpha-2-glycoproteinOS=HomosapiensGN=AZGP1PE=1SV=2

MVRMVPVLLSLLLLLGPAVPQENQDGRYSLTYIYTGLSKHVEDVPAFQALGSLNDLQFFR

YNSKDRKSQPMGLWRQVEGMEDWKQDSQLQKAREDIFMETLKDIVEYYNDSNGSHVLQGR

FGCEIENNRSSGAFWKYYYDGKDYIEFNKEIPAWVPFDPAAQITKQKWEAEPVYVQRAKA

YLEEECPATLRKYLKYSKNILDRQDPPSVVVTSHQAPGEKKKLKCLAYDFYPGKIDVHWT

RAGEVQEPELRGDVLHNGNGTYQSWVVVAVPPQDTAPYSCHVQHSSLAQPLVVPWEAS

190.>sp|O75636|FCN3_HUMANFicolin-3OS=HomosapiensGN=FCN3PE=1SV=2

MDLLWILPSLWLLLLGGPACLKTQEHPSCPGPRELEASKVVLLPSCPGAPGSPGEKGAPG

PQGPPGPPGKMGPKGEPGDPVNLLRCQEGPRNCRELLSQGATLSGWYHLCLPEGRALPVF

CDMDTEGGGWLVFQRRQDGSVDFFRSWSSYRAGFGNQESEFWLGNENLHQLTLQGNWELR

VELEDFNGNRTFAHYATFRLLGEVDHYQLALGKFSEGTAGDSLSLHSGRPFTTYDADHDS

SNSNCAVIVHGAWWYASCYRSNLNGRYAVSEAAAHKYGIDWASGRGVGHPYRRVRMMLR

191.>sp|Q9Y624|JAM1_HUMANJunctionaladhesionmoleculeAOS=HomosapiensGN=F11RPE=1SV=1

MGTKAQVERKLLCLFILAILLCSLALGSVTVHSSEPEVRIPENNPVKLSCAYSGFSSPRV

EWKFDQGDTTRLVCYNNKITASYEDRVTFLPTGITFKSVTREDTGTYTCMVSEEGGNSYG

EVKVKLIVLVPPSKPTVNIPSSATIGNRAVLTCSEQDGSPPSEYTWFKDGIVMPTNPKST

RAFSNSSYVLNPTTGELVFDPLSASDTGEYSCEARNGYGTPMTSNAVRMEAVERNVGVIV

AAVLVTLILLGILVFGIWFAYSRGHFDRTKKGTSSKKVIYSQPSARSEGEFKQTSSFLV

192.>sp|P28907|CD38_HUMANADP-ribosylcyclase/cyclicADP-ribosehydrolase1OS=HomosapiensGN=CD38PE=1SV=2

MANCEFSPVSGDKPCCRLSRRAQLCLGVSILVLILVVVLAVVVPRWRQQWSGPGTTKRFP

ETVLARCVKYTEIHPEMRHVDCQSVWDAFKGAFISKHPCNITEEDYQPLMKLGTQTVPCN

KILLWSRIKDLAHQFTQVQRDMFTLEDTLLGYLADDLTWCGEFNTSKINYQSCPDWRKDC

SNNPVSVFWKTVSRRFAEAACDVVHVMLNGSRSKIFDKNSTFGSVEVHNLQPEKVQTLEA

WVIHGGREDSRDLCQDPTIKELESIISKRNIQFSCKNIYRPDKFLQCVKNPEDSSCTSEI

193.>sp|Q8NBJ7|SUMF2_HUMANSulfatase-modifyingfactor2OS=HomosapiensGN=SUMF2PE=1SV=2

MARHGLPLLPLLSLLVGAWLKLGNGQATSMVQLQGGRFLMGTNSPDSRDGDGPVREATVK

PFAIDIFPVTNKDFRDFVREKKYRTEAEMFGWSFVFEDFVSDELRNKATQPMKSVLWWLP

VEKAFWRQPAGPGSGIRERLEHPVLHVSWNDARAYCAWRGKRLPTEEEWEFAARGGLKGQ

VYPWGNWFQPNRTNLWQGKFPKGDKAEDGFHGVSPVNAFPAQNNYGLYDLLGNVWEWTAS

PYQAAEQDMRVLRGASWIDTADGSANHRARVTTRMGNTPDSASDNLGFRCAADAGRPPGE

L

194.>sp|Q63HQ0|AP1AR_HUMANAP-1complex-associatedregulatoryproteinOS=HomosapiensGN=AP1ARPE=1SV=1

MGNCCWTQCFGLLRKEAGRLQRVGGGGGSKYFRTCSRGEHLTIEFENLVESDEGESPGSS

HRPLTEEEIVDLRERHYDSIAEKQKDLDKKIQKELALQEEKLRLEEEALYAAQREAARAA

KQRKLLEQERQRIVQQYHPSNNGEYQSSGPEDDFESCLRNMKSQYEVFRSSRLSSDATVL

TPNTESSCDLMTKTKSTSGNDDSTSLDLEWEDEEGMNRMLPMRERSKTEEDILRAALKYS

NKKTGSNPTSASDDSNGLEWENDFVSAEMDDNGNSEYSGFVNPVLELSDSGIRHSDTDQQ

TR

195.>sp|P49184|DNSL1_HUMANDeoxyribonuclease-1-like1OS=HomosapiensGN=DNASE1L1PE=1SV=1

MHYPTALLFLILANGAQAFRICAFNAQRLTLAKVAREQVMDTLVRILARCDIMVLQEVVD

SSGSAIPLLLRELNRFDGSGPYSTLSSPQLGRSTYMETYVYFYRSHKTQVLSSYVYNDED

DVFAREPFVAQFSLPSNVLPSLVLVPLHTTPKAVEKELNALYDVFLEVSQHWQSKDVILL

GDFNADCASLTKKRLDKLELRTEPGFHWVIADGEDTTVRASTHCTYDRVVLHGERCRSLL

HTAAAFDFPTSFQLTEEEALNISDHYPVEVELKLSQAHSVQPLSLTVLLLLSLLSPQLCP

AA

196.>sp|O75144|ICOSL_HUMANICOSligandOS=HomosapiensGN=ICOSLGPE=1SV=2

MRLGSPGLLFLLFSSLRADTQEKEVRAMVGSDVELSCACPEGSRFDLNDVYVYWQTSESK

TVVTYHIPQNSSLENVDSRYRNRALMSPAGMLRGDFSLRLFNVTPQDEQKFHCLVLSQSL

GFQEVLSVEVTLHVAANFSVPVVSAPHSPSQDELTFTCTSINGYPRPNVYWINKTDNSLL

DQALQNDTVFLNMRGLYDVVSVLRIARTPSVNIGCCIENVLLQQNLTVGSQTGNDIGERD

KITENPVSTGEKNAATWSILAVLCLLVVVAVAIGWVCRDRCLQHSYAGAWAVSPETELTG

HV

197.>sp|Q9UMR5|PPT2_HUMANLysosomalthioesterasePPT2OS=HomosapiensGN=PPT2PE=1SV=4

MLGLCGQRLPAAWVLLLLPFLPLLLLAAPAPHRASYKPVIVVHGLFDSSYSFRHLLEYIN

ETHPGTVVTVLDLFDGRESLRPLWEQVQGFREAVVPIMAKAPQGVHLICYSQGGLVCRAL

LSVMDDHNVDSFISLSSPQMGQYGDTDYLKWLFPTSMRSNLYRICYSPWGQEFSICNYWH

DPHHDDLYLNASSFLALINGERDHPNATVWRKNFLRVGHLVLIGGPDDGVITPWQSSFFG

FYDANETVLEMEEQLVYLRDSFGLKTLLARGAIVRCPMAGISHTAWHSNRTLYETCIEPW

LS

198.>sp|P05026|AT1B1_HUMANSodium/potassium-transportingATPasesubunitbeta-1OS=HomosapiensGN=ATP1B1PE=1SV=1

MARGKAKEEGSWKKFIWNSEKKEFLGRTGGSWFKILLFYVIFYGCLAGIFIGTIQVMLLT

ISEFKPTYQDRVAPPGLTQIPQIQKTEISFRPNDPKSYEAYVLNIVRFLEKYKDSAQRDD

MIFEDCGDVPSEPKERGDFNHERGERKVCRFKLEWLGNCSGLNDETYGYKEGKPCIIIKL

NRVLGFKPKPPKNESLETYPVMKYNPNVLPVQCTGKRDEDKDKVGNVEYFGLGNSPGFPL

QYYPYYGKLLQPKYLQPLLAVQFTNLTMDTEIRIECKAYGENIGYSEKDRFQGRFDVKIE

VKS(2^nd^NinD.R)

199.>sp|Q9UBR2|CATZ_HUMANCathepsinZOS=HomosapiensGN=CTSZPE=1SV=1

MARRGPGWRPLLLLVLLAGAAQGGLYFRRGQTCYRPLRGDGLAPLGRSTYPRPHEYLSPA

DLPKSWDWRNVDGVNYASITRNQHIPQYCGSCWAHASTSAMADRINIKRKGAWPSTLLSV

QNVIDCGNAGSCEGGNDLSVWDYAHQHGIPDETCNNYQAKDQECDKFNQCGTCNEFKECH

AIRNYTLWRVGDYGSLSGREKMMAEIYANGPISCGIMATERLANYTGGIYAEYQDTTYIN

HVVSVAGWGISDGTEYWIVRNSWGEPWGERGWLRIVTSTYKDGKGARYNLAIEEHCTFGD

PIV

200.>sp|Q9UKJ1|PILRA_HUMANPairedimmunoglobulin-liketype2receptoralphaOS=HomosapiensGN=PILRAPE=1SV=3

MGRPLLLPLLPLLLPPAFLQPSGSTGSGPSYLYGVTQPKHLSASMGGSVEIPFSFYYPWE

LATAPDVRISWRRGHFHRQSFYSTRPPSIHKDYVNRLFLNWTEGQKSGFLRISNLQKQDQ

SVYFCRVELDTRSSGRQQWQSIEGTKLSITQAVTTTTQRPSSMTTTWRLSSTTTTTGLRV

TQGKRRSDSWHISLETAVGVAVAVTVLGIMILGLICLLRWRRRKGQQRTKATTPAREPFQ

NTEEPYENIRNEGQNTDPKLNPKDDGIVYASLALSSSTSPRAPPSHRPLKSPQNETLYSV

LKA

201.>sp|P09486|SPRC_HUMANSPARCOS=HomosapiensGN=SPARCPE=1SV=1

MRAWIFFLLCLAGRALAAPQQEALPDETEVVEETVAEVTEVSVGANPVQVEVGEFDDGAE

ETEEEVVAENPCQNHHCKHGKVCELDENNTPMCVCQDPTSCPAPIGEFEKVCSNDNKTFD

SSCHFFATKCTLEGTKKGHKLHLDYIGPCKYIPPCLDSELTEFPLRMRDWLKNVLVTLYE

RDEDNNLLTEKQKLRVKKIHENEKRLEAGDHPVELLARDFEKNYNMYIFPVHWQFGQLDQ

HPIDGYLSHTELAPLRAPLIPMEHCTTRFFETCDLDNDKYIALDEWAGCFGIKQKDIDKD

LVI

202.>sp|P10646|TFPI1_HUMANTissuefactorpathwayinhibitorOS=HomosapiensGN=TFPIPE=1SV=1

MIYTMKKVHALWASVCLLLNLAPAPLNADSEEDEEHTIITDTELPPLKLMHSFCAFKADD

GPCKAIMKRFFFNIFTRQCEEFIYGGCEGNQNRFESLEECKKMCTRDNANRIIKTTLQQE

KPDFCFLEEDPGICRGYITRYFYNNQTKQCERFKYGGCLGNMNNFETLEECKNICEDGPN

GFQVDNYGTQLNAVNNSLTPQSTKVPSLFEFHGPSWCLTPADRGLCRANENRFYYNSVIG

KCRPFKYSGCGGNENNFTSKQECLRACKKGFIQRISKGGLIKTKRKRKKQRVKIAYEEIF

VKNM

203.>sp|P50897|PPT1_HUMANPalmitoyl-proteinthioesterase1OS=HomosapiensGN=PPT1PE=1SV=1

MASPGCLWLLAVALLPWTCASRALQHLDPPAPLPLVIWHGMGDSCCNPLSMGAIKKMVEK

KIPGIYVLSLEIGKTLMEDVENSFFLNVNSQVTTVCQALAKDPKLQQGYNAMGFSQGGQF

LRAVAQRCPSPPMINLISVGGQHQGVFGLPRCPGESSHICDFIRKTLNAGAYSKVVQERL

VQAEYWHDPIKEDVYRNHSIFLADINQERGINESYKKNLMALKKFVMVKFLNDSIVDPVD

SEWFGFYRSGQAKETIPLQETSLYTQDRLGLKEMDNAGQLVFLATEGDHLQLSEEWFYAH

IIPFLG

204.>sp|Q12841|FSTL1_HUMANFollistatin-relatedprotein1OS=HomosapiensGN=FSTL1PE=1SV=1

MWKRWLALALALVAVAWVRAEEELRSKSKICANVFCGAGRECAVTEKGEPTCLCIEQCKP

HKRPVCGSNGKTYLNHCELHRDACLTGSKIQVDYDGHCKEKKSVSPSASPVVCYQSNRDE

LRRRIIQWLEAEIIPDGWFSKGSNYSEILDKYFKNFDNGDSRLDSSEFLKFVEQNETAIN

ITTYPDQENNKLLRGLCVDALIELSDENADWKLSFQEFLKCLNPSFNPPEKKCALEDETY

ADGAETEVDCNRCVCACGNWVCTAMTCDGKNQKGAQTQTEEEMTRYVQELQKHQETAEKT

KRVSTKEI

(3^rd^NisinD.R)

205.>sp|Q04118|PRB3_HUMANBasicsalivaryproline-richprotein3OS=HomosapiensGN=PRB3PE=1SV=2

MLLILLSVALLALSSAQSLNEDVSQEESPSVISGKPEGRRPQGGNQPQRTPPPPGKPEGR

PPQGGNQSQGPPPRPGKPEGPPPQGGNQSQGPPPRPGKPEGQPPQGGNQSQGPPPRPGKP

EGPPPQGGNQSQGPPPRPGKPEGPPPQGGNQSQGPPPHPGKPEGPPPQGGNQSQGPPPRP

GKPEGPPPQGGNQSQGPPPRPGKPEGPPPQGGNQSQGPPPRPGKPEGSPSQGGNKPQGPP

PHPGKPQGPPPQEGNKPQRPPPPGRPQGPPPPGGNPQQPLPPPAGKPQGPPPPPQGGRPH

RPPQGQPPQ

(The entire protein has disordered region)

206.>sp|Q9BX67|JAM3_HUMANJunctionaladhesionmoleculeCOS=HomosapiensGN=JAM3PE=1SV=1

MALRRPPRLRLCARLPDFFLLLLFRGCLIGAVNLKSSNRTPVVQEFESVELSCIITDSQT

SDPRIEWKKIQDEQTTYVFFDNKIQGDLAGRAEILGKTSLKIWNVTRRDSALYRCEVVAR

NDRKEIDEIVIELTVQVKPVTPVCRVPKAVPVGKMATLHCQESEGHPRPHYSWYRNDVPL

PTDSRANPRFRNSSFHLNSETGTLVFTAVHKDDSGQYYCIASNDAGSARCEEQEMEVYDL

NIGGIIGGVLVVLAVLALITLGICCAYRRGYFINNKQDGESYKNPGKPDGVNYIRTDEEG

DFRHKSSFVI

207.>sp|Q6UWI2|PARM1_HUMANProstateandrogen-regulatedmucin-likeprotein1OS=HomosapiensGN=PARM1PE=1SV=1

MVYKTLFALCILTAGWRVQSLPTSAPLSVSLPTNIVPPTTIWTSSPQNTDADTASPSNGT

HNNSVLPVTASAPTSLLPKNISIESREEEITSPGSNWEGTNTDPSPSGFSSTSGGVHLTT

TLEEHSSGTPEAGVAATLSQSAAEPPTLISPQAPASSPSSLSTSPPEVFSASVTTNHSST

VTSTQPTGAPTAPESPTEESSSDHTPTSHATAEPVPQEKTPPTTVSGKVMCELIDMETTT

TFPRVIMQEVEHALSSGSIAAITVTVIAVVLLVFGVAAYLKIRHSSYGRLLDDHDYGSWG

NYNNPLYDDS

208.>sp|P10163|PRB4_HUMANBasicsalivaryproline-richprotein4OS=HomosapiensGN=PRB4PE=1SV=4

MLLILLSVALLALSSAESSSEDVSQEESLFLISGKPEGRRPQGGNQPQRPPPPPGKPQGP

PPQGGNQSQGPPPPPGKPEGRPPQGGNQSQGPPPHPGKPERPPPQGGNQSQGPPPHPGKP

ESRPPQGGHQSQGPPPTPGKPEGPPPQGGNQSQGTPPPPGKPEGRPPQGGNQSQGPPPHP

GKPERPPPQGGNQSHRPPPPPGKPERPPPQGGNQSQGPPPHPGKPEGPPPQEGNKSRSAR

SPPGKPQGPPQQEGNKPQGPPPPGKPQGPPPAGGNPQQPQAPPAGKPQGPPPPPQGGRPP

RPAQGQQPPQ

(The entire protein has disordered region)

209.>sp|P07307|ASGR2_HUMANAsialoglycoproteinreceptor2OS=HomosapiensGN=ASGR2PE=1SV=2

MAKDFQDIQQLSSEENDHPFHQGEGPGTRRLNPRRGNPFLKGPPPAQPLAQRLCSMVCFS

LLALSFNILLLVVICVTGSQSEGHGGAQLQAELRSLKEAFSNFSSSTLTEVQAISTHGGS

VGDKITSLGAKLEKQQQDLKADHDALLFHLKHFPVDLRFVACQMELLHSNGSQRTCCPVN

WVEHQGSCYWFSHSGKAWAEAEKYCQLENAHLVVINSWEEQKFIVQHTNPFNTWIGLTDS

DGSWKWVDGTDYRHNYKNWAVTQPDNWHGHELGGSEDCVEVQPDGRWNDDFCLQVYRWVC

EKRRNATGEVA

(3^rd^NinO.R)

210.>sp|Q15485|FCN2_HUMANFicolin-2OS=HomosapiensGN=FCN2PE=1SV=2

MELDRAVGVLGAATLLLSFLGMAWALQAADTCPEVKMVGLEGSDKLTILRGCPGLPGAPG

PKGEAGTNGKRGERGPPGPPGKAGPPGPNGAPGEPQPCLTGPRTCKDLLDRGHFLSGWHT

IYLPDCRPLTVLCDMDTDGGGWTVFQRRVDGSVDFYRDWATYKQGFGSRLGEFWLGNDNI

HALTAQGTSELRVDLVDFEDNYQFAKYRSFKVADEAEKYNLVLGAFVEGSAGDSLTFHNN

QSFSTKDQDNDLNTGNCAVMFQGAWWYKNCHVSNLNGRYLRGTHGSFANGINWKSGKGYN

YSYKVSEMKVRPA

211.>sp|Q9GZR5|ELOV4_HUMANElongationofverylongchainfattyacidsprotein4OS=HomosapiensGN=ELOVL4PE=1SV=1

MGLLDSEPGSVLNVVSTALNDTVEFYRWTWSIADKRVENWPLMQSPWPTLSISTLYLLFV

WLGPKWMKDREPFQMRLVLIIYNFGMVLLNLFIFRELFMGSYNAGYSYICQSVDYSNNVH

EVRIAAALWWYFVSKGVEYLDTVFFILRKKNNQVSFLHVYHHCTMFTLWWIGIKWVAGGQ

AFFGAQLNSFIHVIMYSYYGLTAFGPWIQKYLWWKRYLTMLQLIQFHVTIGHTALSLYTD

CPFPKWMHWALIAYAISFIFLFLNFYIRTYKEPKKPKAGKTAMNGISANGVSKSEKQLMI

ENGKKQKNGKAKGD

212.>sp|P16422|EPCAM_HUMANEpithelialcelladhesionmoleculeOS=HomosapiensGN=EPCAMPE=1SV=2

MAPPQVLAFGLLLAAATATFAAAQEECVCENYKLAVNCFVNNNRQCQCTSVGAQNTVICS

KLAAKCLVMKAEMNGSKLGRRAKPEGALQNNDGLYDPDCDESGLFKAKQCNGTSMCWCVN

TAGVRRTDKDTEITCSERVRTYWIIIELKHKAREKPYDSKSLRTALQKEITTRYQLDPKF

ITSILYENNVITIDLVQNSSQKTQNDVDIADVAYYFEKDVKGESLFHSKKMDLTVNGEQL

DLDPGQTLIYYVDEKAPEFSMQGLKAGVIAVIVVVVIAVVAGIVVLVISRKKRMAKYEKA

EIKEMGEMHRELNA

(1^st^NinD.R)(3^rd^NinO.R)

213.>sp|P10451|OSTP_HUMANOsteopontinOS=HomosapiensGN=SPP1PE=1SV=1

MRIAVICFCLLGITCAIPVKQADSGSSEEKQLYNKYPDAVATWLNPDPSQKQNLLAPQNA

VSSEETNDFKQETLPSKSNESHDHMDDMDDEDDDDHVDSQDSIDSNDSDDVDDTDDSHQS

DESHHSDESDELVTDFPTDLPATEVFTPVVPTVDTYDGRGDSVVYGLRSKSKKFRRPDIQ

YPDATDEDITSHMESEELNGAYKAIPVAQDLNAPSDWDSRGKDSYETSQLDDQSAETHSH

KQSRLYKRKANDESNEHSDVIDSQELSKVSREFHSHEFHSHEDMLVVDPKSKEEDKHLKF

RISHELDSASSEVN

214.>sp|Q8N474|SFRP1_HUMANSecretedfrizzled-relatedprotein1OS=HomosapiensGN=SFRP1PE=1SV=1

MGIGRSEGGRRGAALGVLLALGAALLAVGSASEYDYVSFQSDIGPYQSGRFYTKPPQCVD

IPADLRLCHNVGYKKMVLPNLLEHETMAEVKQQASSWVPLLNKNCHAGTQVFLCSLFAPV

CLDRPIYPCRWLCEAVRDSCEPVMQFFGFYWPEMLKCDKFPEGDVCIAMTPPNATEASKP

QGTTVCPPCDNELKSEAIIEHLCASEFALRMKIKEVKKENGDKKIVPKKKKPLKLGPIKK

KDLKKLVLYLKNGADCPCHQLDNLSHHFLIMGRKVKSQYLLTAIHKWDKKNKEFKNFMKK

MKNHECPTFQSVFK

215.>sp|O43852|CALU_HUMANCalumeninOS=HomosapiensGN=CALUPE=1SV=2

MDLRQFLMCLSLCTAFALSKPTEKKDRVHHEPQLSDKVHNDAQSFDYDHDAFLGAEEAKT

FDQLTPEESKERLGKIVSKIDGDKDGFVTVDELKDWIKFAQKRWIYEDVERQWKGHDLNE

DGLVSWEEYKNATYGYVLDDPDPDDGFNYKQMMVRDERRFKMADKDGDLIATKEEFTAFL

HPEEYDYMKDIVVQETMEDIDKNADGFIDLEEYIGDMYSHDGNTDEPEWVKTEREQFVEF

RDKNRDGKMDKEETKDWILPSDYDHAEAEARHLVYESDQNKDGKLTKEEIVDKYDLFVGS

QATDFGEALVRHDEF

216.>sp|P02649|APOE_HUMANApolipoproteinEOS=HomosapiensGN=APOEPE=1SV=1

MKVLWAALLVTFLAGCQAKVEQAVETEPEPELRQQTEWQSGQRWELALGRFWDYLRWVQT

LSEQVQEELLSSQVTQELRALMDETMKELKAYKSELEEQLTPVAEETRARLSKELQAAQA

RLGADMEDVCGRLVQYRGEVQAMLGQSTEELRVRLASHLRKLRKRLLRDADDLQKRLAVY

QAGAREGAERGLSAIRERLGPLVEQGRVRAATVGSLAGQPLQERAQAWGERLRARMEEMG

SRTRDRLDEVKEQVAEVRAKLEEQAQQIRLQAEAFQARLKSWFEPLVEDMQRQWAGLVEK

VQAAVGTSAAPVPSDNH

(glycation)

217.>sp|Q9GZN4|BSSP4_HUMANBrain-specificserineprotease4OS=HomosapiensGN=PRSS22PE=1SV=1

MVVSGAPPALGGGCLGTFTSLLLLASTAILNAARIPVPPACGKPQQLNRVVGGEDSTDSE

WPWIVSIQKNGTHHCAGSLLTSRWVITAAHCFKDNLNKPYLFSVLLGAWQLGNPGSRSQK

VGVAWVEPHPVYSWKEGACADIALVRLERSIQFSERVLPICLPDASIHLPPNTHCWISGW

GSIQDGVPLPHPQTLQKLKVPIIDSEVCSHLYWRGAGQGPITEDMLCAGYLEGERDACLG

DSGGPLMCQVDGAWLLAGIISWGEGCAERNRPGVYISLSAHRSWVEKIVQGVQLRGRAQG

GGALRAPSQGSGAAARS

218.>sp|P12318|FCG2A_HUMANLowaffinityimmunoglobulingammaFcregionreceptorII-aOS=HomosapiensGN=FCGR2APE=1SV=4

MTMETQMSQNVCPRNLWLLQPLTVLLLLASADSQAAAPPKAVLKLEPPWINVLQEDSVTL

TCQGARSPESDSIQWFHNGNLIPTHTQPSYRFKANNNDSGEYTCQTGQTSLSDPVHLTVL

SEWLVLQTPHLEFQEGETIMLRCHSWKDKPLVKVTFFQNGKSQKFSHLDPTFSIPQANHS

HSGDYHCTGNIGYTLFSSKPVTITVQVPSMGSSSPMGIIVAVVIATAVAAIVAAVVALIY

CRKKRISANSTDPVKAAQFEPPGRQMIAIRKRQLEETNNDYETADGGYMTLNPRAPTDDD

KNIYLTLPPNDHVNSNN

219.>sp|Q9NYV8|T2R14_HUMANTastereceptortype2member14OS=HomosapiensGN=TAS2R14PE=1SV=1

MGGVIKSIFTFVLIVEFIIGNLGNSFIALVNCIDWVKGRKISSVDRILTALAISRISLVW

LIFGSWCVSVFFPALFATEKMFRMLTNIWTVINHFSVWLATGLGTFYFLKIANFSNSIFL

YLKWRVKKVVLVLLLVTSVFLFLNIALINIHINASINGYRRNKTCSSDSSNFTRFSSLIV

LTSTVFIFIPFTLSLAMFLLLIFSMWKHRKKMQHTVKISGDASTKAHRGVKSVITFFLLY

AIFSLSFFISVWTSERLEENLIILSQVMGMAYPSCHSCVLILGNKKLRQASLSVLLWLRY

MFKDGEPSGHKEFRESS

(2^ND^NinO.R)

220.>sp|Q10588|BST1_HUMANADP-ribosylcyclase/cyclicADP-ribosehydrolase2OS=HomosapiensGN=BST1PE=1SV=2

MAAQGCAASRLLQLLLQLLLLLLLLAAGGARARWRGEGTSAHLRDIFLGRCAEYRALLSP

EQRNKNCTAIWEAFKVALDKDPCSVLPSDYDLFINLSRHSIPRDKSLFWENSHLLVNSFA

DNTRRFMPLSDVLYGRVADFLSWCRQKNDSGLDYQSCPTSEDCENNPVDSFWKRASIQYS

KDSSGVIHVMLNGSEPTGAYPIKGFFADYEIPNLQKEKITRIEIWVMHEIGGPNVESCGE

GSMKVLEKRLKDMGFQYSCINDYRPVKLLQCVDHSTHPDCALKSAAAATQRKAPSLYTEQ

RAGLIIPLFLVLASRTQL

221.>sp|Q92820|GGH_HUMANGamma-glutamylhydrolaseOS=HomosapiensGN=GGHPE=1SV=2

MASPGCLLCVLGLLLCGAASLELSRPHGDTAKKPIIGILMQKCRNKVMKNYGRYYIAASY

VKYLESAGARVVPVRLDLTEKDYEILFKSINGILFPGGSVDLRRSDYAKVAKIFYNLSIQ

SFDDGDYFPVWGTCLGFEELSLLISGECLLTATDTVDVAMPLNFTGGQLHSRMFQNFPTE

LLLSLAVEPLTANFHKWSLSVKNFTMNEKLKKFFNVLTTNTDGKIEFISTMEGYKYPVYG

VQWHPEKAPYEWKNLDGISHAPNAVKTAFYLAEFFVNEARKNNHHFKSESEEEKALIYQF

SPIYTGNISSFQQCYIFD

222.>sp|Q16585|SGCB_HUMANBeta-sarcoglycanOS=HomosapiensGN=SGCBPE=1SV=1

MAAAAAAAAEQQSSNGPVKKSMREKAVERRSVNKEHNSNFKAGYIPIDEDRLHKTGLRGR

KGNLAICVIILLFILAVINLIITLVIWAVIRIGPNGCDSMEFHESGLLRFKQVSDMGVIH

PLYKSTVGGRRNENLVITGNNQPIVFQQGTTKLSVENNKTSITSDIGMQFFDPRTQNILF

STDYETHEFHLPSGVKSLNVQKASTERITSNATSDLNIKVDGRAIVRGNEGVFIMGKTIE

FHMGGNMELKAENSIILNGSVMVSTTRLPSSSSGDQLGSGDWVRYKLCMCADGTLFKVQV

TSQNMGCQISDNPCGNTH

223.>sp|Q99795|GPA33_HUMANCellsurfaceA33antigenOS=HomosapiensGN=GPA33PE=1SV=1

MVGKMWPVLWTLCAVRVTVDAISVETPQDVLRASQGKSVTLPCTYHTSTSSREGLIQWDK

LLLTHTERVVIWPFSNKNYIHGELYKNRVSISNNAEQSDASITIDQLTMADNGTYECSVS

LMSDLEGNTKSRVRLLVLVPPSKPECGIEGETIIGNNIQLTCQSKEGSPTPQYSWKRYNI

LNQEQPLAQPASGQPVSLKNISTDTSGYYICTSSNEEGTQFCNITVAVRSPSMNVALYVG

IAVGVVAALIIIGIIIYCCCCRGKDDNTEDKEDARPNREAYEEPPEQLRELSREREEEDD

YRQEEQRSTGRESPDHLDQ

224.>sp|Q9Y5Y7|LYVE1_HUMANLymphaticvesselendothelialhyaluronicacidreceptor1OS=HomosapiensGN=LYVE1PE=1SV=2

MARCFSLVLLLTSIWTTRLLVQGSLRAEELSIQVSCRIMGITLVSKKANQQLNFTEAKEA

CRLLGLSLAGKDQVETALKASFETCSYGWVGDGFVVISRISPNPKCGKNGVGVLIWKVPV

SRQFAAYCYNSSDTWTNSCIPEIITTKDPIFNTQTATQTTEFIVSDSTYSVASPYSTIPA

PTTTPPAPASTSIPRRKKLICVTEVFMETSTMSTETEPFVENKAAFKNEAAGFGGVPTAL

LVLALLFFGAAAGLGFCYVKRYVKAFPFTNKNQQKEMIETKVVKEEKANDSNPNEESKKT

DKNPEESKSPSKTTVRCLEAEV

225.>sp|Q08722|CD47_HUMANLeukocytesurfaceantigenCD47OS=HomosapiensGN=CD47PE=1SV=1

MWPLVAALLLGSACCGSAQLLFNKTKSVEFTFCNDTVVIPCFVTNMEAQNTTEVYVKWKF

KGRDIYTFDGALNKSTVPTDFSSAKIEVSQLLKGDASLKMDKSDAVSHTGNYTCEVTELT

REGETIIELKYRVVSWFSPNENILIVIFPIFAILLFWGQFGIKTLKYRSGGMDEKTIALL

VAGLVITVIVIVGAILFVPGEYSLKNATGLGLIVTSTGILILLHYYVFSTAIGLTSFVIA

ILVIQVIAYILAVVGLSLCIAACIPMHGPLLISGLSILALAQLLGLVYMKFVASNQKTIQ

PPRKAVEEPLNAFKESKGMMNDE

(LastNisD.R)

226.>sp|P31995|FCG2C_HUMANLowaffinityimmunoglobulingammaFcregionreceptorII-cOS=HomosapiensGN=FCGR2CPE=1SV=1

MGILSFLPVLATESDWADCKSPQPWGHMLLWTAVLFLAPVAGTPAAPPKAVLKLEPQWIN

VLQEDSVTLTCRGTHSPESDSIQWFHNGNLIPTHTQPSYRFKANNNDSGEYTCQTGQTSL

SDPVHLTVLSEWLVLQTPHLEFQEGETIVLRCHSWKDKPLVKVTFFQNGKSKKFSRSDPN

FSIPQANHSHSGDYHCTGNIGYTLYSSKPVTITVQAPSSSPMGIIVAVVTGIAVAAIVAA

VVALIYCRKKRISANSTDPVKAAQFEPPGRQMIAIRKRQPEETNNDYETADGGYMTLNPR

APTDDDKNIYLTLPPNDHVNSNN

227.>sp|P13686|PPA5_HUMANTartrate-resistantacidphosphatasetype5OS=HomosapiensGN=ACP5PE=1SV=3

MDMWTALLILQALLLPSLADGATPALRFVAVGDWGGVPNAPFHTAREMANAKEIARTVQI

LGADFILSLGDNFYFTGVQDINDKRFQETFEDVFSDRSLRKVPWYVLAGNHDHLGNVSAQ

IAYSKISKRWNFPSPFYRLHFKIPQTNVSVAIFMLDTVTLCGNSDDFLSQQPERPRDVKL

ARTQLSWLKKQLAAAREDYVLVAGHYPVWSIAEHGPTHCLVKQLRPLLATYGVTAYLCGH

DHNLQYLQDENGVGYVLSGAGNFMDPSKRHQRKVPNGYLRFHYGTEDSLGGFAYVEISSK

EMTVTYIEASGKSLFKTRLPRRARP

228.>sp|P01859|IGHG2_HUMANIggamma-2chainCregionOS=HomosapiensGN=IGHG2PE=1SV=2

ASTKGPSVFPLAPCSRSTSESTAALGCLVKDYFPEPVTVSWNSGALTSGVHTFPAVLQSS

GLYSLSSVVTVPSSNFGTQTYTCNVDHKPSNTKVDKTVERKCCVECPPCPAPPVAGPSVF

LFPPKPKDTLMISRTPEVTCVVVDVSHEDPEVQFNWYVDGVEVHNAKTKPREEQFNSTFR

VVSVLTVVHQDWLNGKEYKCKVSNKGLPAPIEKTISKTKGQPREPQVYTLPPSREEMTKN

QVSLTCLVKGFYPSDISVEWESNGQPENNYKTTPPMLDSDGSFFLYSKLTVDKSRWQQGN

VFSCSVMHEALHNHYTQKSLSLSPGK

229.>sp|P06126|CD1A_HUMANT-cellsurfaceglycoproteinCD1aOS=HomosapiensGN=CD1APE=1SV=4

MLFLLLPLLAVLPGDGNADGLKEPLSFHVTWIASFYNHSWKQNLVSGWLSDLQTHTWDSN

SSTIVFLCPWSRGNFSNEEWKELETLFRIRTIRSFEGIRRYAHELQFEYPFEIQVTGGCE

LHSGKVSGSFLQLAYQGSDFVSFQNNSWLPYPVAGNMAKHFCKVLNQNQHENDITHNLLS

DTCPRFILGLLDAGKAHLQRQVKPEAWLSHGPSPGPGHLQLVCHVSGFYPKPVWVMWMRG

EQEQQGTQRGDILPSADGTWYLRATLEVAAGEAADLSCRVKHSSLEGQDIVLYWEHHSSV

GFIILAVIVPLLLLIGLALWFRKRCFC

230.>sp|Q6PCB8|EMB_HUMANEmbiginOS=HomosapiensGN=EMBPE=1SV=1

MRALPGLLEARARTPRLLLLQCLLAAARPSSADGSAPDSPFTSPPLREEIMANNFSLESH

NISLTEHSSMPVEKNITLERPSNVNLTCQFTTSGDLNAVNVTWKKDGEQLENNYLVSATG

STLYTQYRFTIINSKQMGSYSCFFREEKEQRGTFNFKVPELHGKNKPLISYVGDSTVLTC

KCQNCFPLNWTWYSSNGSVKVPVGVQMNKYVINGTYANETKLKITQLLEEDGESYWCRAL

FQLGESEEHIELVVLSYLVPLKPFLVIVAEVILLVATILLCEKYTQKKKKHSDEGKEFEQ

IEQLKSDDSNGIENNVPRHRKNESLGQ

231.>sp|P01861|IGHG4_HUMANIggamma-4chainCregionOS=HomosapiensGN=IGHG4PE=1SV=1

ASTKGPSVFPLAPCSRSTSESTAALGCLVKDYFPEPVTVSWNSGALTSGVHTFPAVLQSS

GLYSLSSVVTVPSSSLGTKTYTCNVDHKPSNTKVDKRVESKYGPPCPSCPAPEFLGGPSV

FLFPPKPKDTLMISRTPEVTCVVVDVSQEDPEVQFNWYVDGVEVHNAKTKPREEQFNSTY

RVVSVLTVLHQDWLNGKEYKCKVSNKGLPSSIEKTISKAKGQPREPQVYTLPPSQEEMTK

NQVSLTCLVKGFYPSDIAVEWESNGQPENNYKTTPPVLDSDGSFFLYSRLTVDKSRWQEG

NVFSCSVMHEALHNHYTQKSLSLSLGK

232.>sp|O75493|CAH11_HUMANCarbonicanhydrase-relatedprotein11OS=HomosapiensGN=CA11PE=1SV=2

MGAAARLSAPRALVLWAALGAAAHIGPAPDPEDWWSYKDNLQGNFVPGPPFWGLVNAAWS

LCAVGKRQSPVDVELKRVLYDPFLPPLRLSTGGEKLRGTLYNTGRHVSFLPAPRPVVNVS

GGPLLYSHRLSELRLLFGARDGAGSEHQINHQGFSAEVQLIHFNQELYGNFSAASRGPNG

LAILSLFVNVASTSNPFLSRLLNRDTITRISYKNDAYFLQDLSLELLFPESFGFITYQGS

LSTPPCSETVTWILIDRALNITSLQMHSLRLLSQNPPSQIFQSLSGNSRPLQPLAHRALR

GNRDPRHPERRCRGPNYRLHVDGVPHGR

233.>sp|P29460|IL12B_HUMANInterleukin-12subunitbetaOS=HomosapiensGN=IL12BPE=1SV=1

MCHQQLVISWFSLVFLASPLVAIWELKKDVYVVELDWYPDAPGEMVVLTCDTPEEDGITW

TLDQSSEVLGSGKTLTIQVKEFGDAGQYTCHKGGEVLSHSLLLLHKKEDGIWSTDILKDQ

KEPKNKTFLRCEAKNYSGRFTCWWLTTISTDLTFSVKSSRGSSDPQGVTCGAATLSAERV

RGDNKEYEYSVECQEDSACPAAEESLPIEVMVDAVHKLKYENYTSSFFIRDIIKPDPPKN

LQLKPLKNSRQVEVSWEYPDTWSTPHSYFSLTFCVQVQGKSKREKKDRVFTDKTSATVIC

RKNASISVRAQDRYYSSSWSEWASVPCS

234.>sp|Q96D15|RCN3_HUMANReticulocalbin-3OS=HomosapiensGN=RCN3PE=1SV=1

MMWRPSVLLLLLLLRHGAQGKPSPDAGPHGQGRVHQAAPLSDAPHDDAHGNFQYDHEAFL

GREVAKEFDQLTPEESQARLGRIVDRMDRAGDGDGWVSLAELRAWIAHTQQRHIRDSVSA

AWDTYDTDRDGRVGWEELRNATYGHYAPGEEFHDVEDAETYKKMLARDERRFRVADQDGD

SMATREELTAFLHPEEFPHMRDIVIAETLEDLDRNKDGYVQVEEYIADLYSAEPGEEEPA

WVQTERQQFRDFRDLNKDGHLDGSEVGHWVLPPAQDQPLVEANHLLHESDTDKDGRLSKA

EILGNWNMFVGSQATNYGEDLTRHHDEL

235.>sp|Q9UBQ6|EXTL2_HUMANExostosin-like2OS=HomosapiensGN=EXTL2PE=1SV=1

MRCCHICKLPGRVMGIRVLRLSLVVILVLLLVAGALTALLPSVKEDKMLMLRREIKSQGK

STMDSFTLIMQTYNRTDLLLKLLNHYQAVPNLHKVIVVWNNIGEKAPDELWNSLGPHPIP

VIFKQQTANRMRNRLQVFPELETNAVLMVDDDTLISTPDLVFAFSVWQQFPDQIVGFVPR

KHVSTSSGIYSYGSFEMQAPGSGNGDQYSMVLIGASFFNSKYLELFQRQPAAVHALIDDT

QNCDDIAMNFIIAKHIGKTSGIFVKPVNMDNLEKETNSGYSGMWHRAEHALQRSYCINKL

VNIYDSMPLRYSNIMISQFGFPYANYKRKI

236.>sp|Q03591|FHR1_HUMANComplementfactorH-relatedprotein1OS=HomosapiensGN=CFHR1PE=1SV=2

MWLLVSVILISRISSVGGEATFCDFPKINHGILYDEEKYKPFSQVPTGEVFYYSCEYNFV

SPSKSFWTRITCTEEGWSPTPKCLRLCFFPFVENGHSESSGQTHLEGDTVQIICNTGYRL

QNNENNISCVERGWSTPPKCRSTDTSCVNPPTVQNAHILSRQMSKYPSGERVRYECRSPY

EMFGDEEVMCLNGNWTEPPQCKDSTGKCGPPPPIDNGDITSFPLSVYAPASSVEYQCQNL

YQLEGNKRITCRNGQWSEPPKCLHPCVISREIMENYNIALRWTAKQKLYLRTGESAEFVC

KRGYRLSSRSHTLRTTCWDGKLEYPTCAKR

237.>sp|Q02985|FHR3_HUMANComplementfactorH-relatedprotein3OS=HomosapiensGN=CFHR3PE=1SV=2

MLLLINVILTLWVSCANGQVKPCDFPDIKHGGLFHENMRRPYFPVAVGKYYSYYCDEHFE

TPSGSYWDYIHCTQNGWSPAVPCLRKCYFPYLENGYNQNYGRKFVQGNSTEVACHPGYGL

PKAQTTVTCTEKGWSPTPRCIRVRTCSKSDIEIENGFISESSSIYILNKEIQYKCKPGYA

TADGNSSGSITCLQNGWSAQPICINSSEKCGPPPPISNGDTTSFLLKVYVPQSRVEYQCQ

PYYELQGSNYVTCSNGEWSEPPRCIHPCIITEENMNKNNIKLKGRSDRKYYAKTGDTIEF

MCKLGYNANTSILSFQAVCREGIVEYPRCE

238.>sp|P01857|IGHG1_HUMANIggamma-1chainCregionOS=HomosapiensGN=IGHG1PE=1SV=1

ASTKGPSVFPLAPSSKSTSGGTAALGCLVKDYFPEPVTVSWNSGALTSGVHTFPAVLQSS

GLYSLSSVVTVPSSSLGTQTYICNVNHKPSNTKVDKKVEPKSCDKTHTCPPCPAPELLGG

PSVFLFPPKPKDTLMISRTPEVTCVVVDVSHEDPEVKFNWYVDGVEVHNAKTKPREEQYN

STYRVVSVLTVLHQDWLNGKEYKCKVSNKALPAPIEKTISKAKGQPREPQVYTLPPSRDE

LTKNQVSLTCLVKGFYPSDIAVEWESNGQPENNYKTTPPVLDSDGSFFLYSKLTVDKSRW

QQGNVFSCSVMHEALHNHYTQKSLSLSPGK

239.>sp|Q6P5S2|LEG1H_HUMANProteinLEG1homologOS=HomosapiensGN=LEG1PE=1SV=2

MAFLPSWVCVLVGSFSASLAGTSNLSETEPPLWKESPGQLSDYRVENSMYIINPWVYLER

MGMYKIILNQTARYFAKFAPDNEQNILWGLPLQYGWQYRTGRLADPTRRTNCGYESGDHM

CISVDSWWADLNYFLSSLPFLAAVDSGVMGISSDQVRLLPPPKNERKFCYDVSSCRSSFP

ETMNKWNTFYQYLQSPFSKFDDLLKYLWAAHTSTLADNIKSFEDRYDYYSKAEAHFERSW

VLAVDHLAAVLFPTTLIRSYKFQKGMPPRILLNTDVAPFISDFTAFQNVVLVLLNMLDNV

DKSIGYLCTEKSNVYRDHSESSSRSYGNNS

240.>sp|Q15293|RCN1_HUMANReticulocalbin-1OS=HomosapiensGN=RCN1PE=1SV=1

MARGGRGRRLGLALGLLLALVLAPRVLRAKPTVRKERVVRPDSELGERPPEDNQSFQYDH

EAFLGKEDSKTFDQLTPDESKERLGKIVDRIDNDGDGFVTTEELKTWIKRVQKRYIFDNV

AKVWKDYDRDKDDKISWEEYKQATYGYYLGNPAEFHDSSDHHTFKKMLPRDERRFKAADL

NGDLTATREEFTAFLHPEEFEHMKEIVVLETLEDIDKNGDGFVDQDEYIADMFSHEENGP

EPDWVLSEREQFNEFRDLNKDGKLDKDEIRHWILPQDYDHAQAEARHLVYESDKNKDEKL

TKEEILENWNMFVGSQATNYGEDLTKNHDEL

241.>sp|Q9Y274|SIA10_HUMANType2lactosaminealpha-2,3-sialyltransferaseOS=HomosapiensGN=ST3GAL6PE=1SV=1

MRGYLVAIFLSAVFLYYVLHCILWGTNVYWVAPVEMKRRNKIQPCLSKPAFASLLRFHQF

HPFLCAADFRKIASLYGSDKFDLPYGMRTSAEYFRLALSKLQSCDLFDEFDNIPCKKCVV

VGNGGVLKNKTLGEKIDSYDVIIRMNNGPVLGHEEEVGRRTTFRLFYPESVFSDPIHNDP

NTTVILTAFKPHDLRWLLELLMGDKINTNGFWKKPALNLIYKPYQIRILDPFIIRTAAYE

LLHFPKVFPKNQKPKHPTTGIIAITLAFYICHEVHLAGFKYNFSDLKSPLHYYGNATMSL

MNKNAYHNVTAEQLFLKDIIEKNLVINLTQD

242.>sp|Q9UBM4|OPT_HUMANOpticinOS=HomosapiensGN=OPTCPE=1SV=1

MRLLAFLSLLALVLQETGTASLPRKERKRREEQMPREGDSFEVLPLRNDVLNPDNYGEVI

DLSNYEELTDYGDQLPEVKVTSLAPATSISPAKSTTAPGTPSSNPTMTRPTTAGLLLSSQ

PNHGLPTCLVCVCLGSSVYCDDIDLEDIPPLPRRTAYLYARFNRISRIRAEDFKGLTKLK

RIDLSNNLISSIDNDAFRLLHALQDLILPENQLEALPVLPSGIEFLDVRLNRLQSSGIQP

AAFRAMEKLQFLYLSDNLLDSIPGPLPLSLRSVHLQNNLIETMQRDVFCDPEEHKHTRRQ

LEDIRLDGNPINLSLFPSAYFCLPRLPIGRFT

243.>sp|Q96DU3|SLAF6_HUMANSLAMfamilymember6OS=HomosapiensGN=SLAMF6PE=1SV=3

MLWLFQSLLFVFCFGPGNVVSQSSLTPLMVNGILGESVTLPLEFPAGEKVNFITWLFNET

SLAFIVPHETKSPEIHVTNPKQGKRLNFTQSYSLQLSNLKMEDTGSYRAQISTKTSAKLS

SYTLRILRQLRNIQVTNHSQLFQNMTCELHLTCSVEDADDNVSFRWEALGNTLSSQPNLT

VSWDPRISSEQDYTCIAENAVSNLSFSVSAQKLCEDVKIQYTDTKMILFMVSGICIVFGF

IILLLLVLRKRRDSLSLSTQRTQGPAESARNLEYVSVSPTNNTVYASVTHSNRETEIWTP

RENDTITIYSTINHSKESKPTFSRATALDNVV

244.>sp|O14656|TOR1A_HUMANTorsin-1AOS=HomosapiensGN=TOR1APE=1SV=1

MKLGRAVLGLLLLAPSVVQAVEPISLGLALAGVLTGYIYPRLYCLFAECCGQKRSLSREA

LQKDLDDNLFGQHLAKKIILNAVFGFINNPKPKKPLTLSLHGWTGTGKNFVSKIIAENIY

EGGLNSDYVHLFVATLHFPHASNITLYKDQLQLWIRGNVSACARSIFIFDEMDKMHAGLI

DAIKPFLDYYDLVDGVSYQKAMFIFLSNAGAERITDVALDFWRSGKQREDIKLKDIEHAL

SVSVFNNKNSGFWHSSLIDRNLIDYFVPFLPLEYKHLKMCIRVEMQSRGYEIDEDIVSRV

AEEMTFFPKEERVFSDKGCKTVFTKLDYYYDD

245.>sp|P07711|CATL1_HUMANCathepsinL1OS=HomosapiensGN=CTSLPE=1SV=2

MNPTLILAAFCLGIASATLTFDHSLEAQWTKWKAMHNRLYGMNEEGWRRAVWEKNMKMIE

LHNQEYREGKHSFTMAMNAFGDMTSEEFRQVMNGFQNRKPRKGKVFQEPLFYEAPRSVDW

REKGYVTPVKNQGQCGSCWAFSATGALEGQMFRKTGRLISLSEQNLVDCSGPQGNEGCNG

GLMDYAFQYVQDNGGLDSEESYPYEATEESCKYNPKYSVANDTGFVDIPKQEKALMKAVA

TVGPISVAIDAGHESFLFYKEGIYFEPDCSSEDMDHGVLVVGYGFESTESDNNKYWLVKN

SWGEEWGMGGYVKMAKDRRNHCGIASAASYPTV

246.>sp|P29016|CD1B_HUMANT-cellsurfaceglycoproteinCD1bOS=HomosapiensGN=CD1BPE=1SV=1

MLLLPFQLLAVLFPGGNSEHAFQGPTSFHVIQTSSFTNSTWAQTQGSGWLDDLQIHGWDS

DSGTAIFLKPWSKGNFSDKEVAELEEIFRVYIFGFAREVQDFAGDFQMKYPFEIQGIAGC

ELHSGGAIVSFLRGALGGLDFLSVKNASCVPSPEGGSRAQKFCALIIQYQGIMETVRILL

YETCPRYLLGVLNAGKADLQRQVKPEAWLSSGPSPGPGRLQLVCHVSGFYPKPVWVMWMR

GEQEQQGTQLGDILPNANWTWYLRATLDVADGEAAGLSCRVKHSSLEGQDIILYWRNPTS

IGSIVLAIIVPSLLLLLCLALWYMRRRSYQNIP

247.>sp|P29017|CD1C_HUMANT-cellsurfaceglycoproteinCD1cOS=HomosapiensGN=CD1CPE=1SV=2

MLFLQFLLLALLLPGGDNADASQEHVSFHVIQIFSFVNQSWARGQGSGWLDELQTHGWDS

ESGTIIFLHNWSKGNFSNEELSDLELLFRFYLFGLTREIQDHASQDYSKYPFEVQVKAGC

ELHSGKSPEGFFQVAFNGLDLLSFQNTTWVPSPGCGSLAQSVCHLLNHQYEGVTETVYNL

IRSTCPRFLLGLLDAGKMYVHRQVRPEAWLSSRPSLGSGQLLLVCHASGFYPKPVWVTWM

RNEQEQLGTKHGDILPNADGTWYLQVILEVASEEPAGLSCRVRHSSLGGQDIILYWGHHF

SMNWIALVVIVPLVILIVLVLWFKKHCSYQDIL

248.>sp|P52799|EFNB2_HUMANEphrin-B2OS=HomosapiensGN=EFNB2PE=1SV=1

MAVRRDSVWKYCWGVLMVLCRTAISKSIVLEPIYWNSSNSKFLPGQGLVLYPQIGDKLDI

ICPKVDSKTVGQYEYYKVYMVDKDQADRCTIKKENTPLLNCAKPDQDIKFTIKFQEFSPN

LWGLEFQKNKDYYIISTSNGSLEGLDNQEGGVCQTRAMKILMKVGQDASSAGSTRNKDPT

RRPELEAGTNGRSSTTSPFVKPNPGSSTDGNSAGHSGNNILGSEVALFAGIASGCIIFIV

IIITLVVLLLKYRRRHRKHSPQHTTTLSLSTLATPKRSGNNNGSEPSDIIIPLRTADSVF

CPHYEKVSGDYGHPVYIVQEMPPQSPANIYYKV

249.>sp|O60911|CATL2_HUMANCathepsinL2OS=HomosapiensGN=CTSVPE=1SV=2

MNLSLVLAAFCLGIASAVPKFDQNLDTKWYQWKATHRRLYGANEEGWRRAVWEKNMKMIE

LHNGEYSQGKHGFTMAMNAFGDMTNEEFRQMMGCFRNQKFRKGKVFREPLFLDLPKSVDW

RKKGYVTPVKNQKQCGSCWAFSATGALEGQMFRKTGKLVSLSEQNLVDCSRPQGNQGCNG

GFMARAFQYVKENGGLDSEESYPYVAVDEICKYRPENSVANDTGFTVVAPGKEKALMKAV

ATVGPISVAMDAGHSSFQFYKSGIYFEPDCSSKNLDHGVLVVGYGFEGANSNNSKYWLVK

NSWGPEWGSNGYVKIAKDKNNHCGIATAASYPNV

250.>sp|Q2I0M4|LRC26_HUMANLeucine-richrepeat-containingprotein26OS=HomosapiensGN=LRRC26PE=1SV=2

MRGPSWSRPRPLLLLLLLLSPWPVWAQVSATASPSGSLGAPDCPEVCTCVPGGLASCSAL

SLPAVPPGLSLRLRALLLDHNRVRALPPGAFAGAGALQRLDLRENGLHSVHVRAFWGLGA

LQLLDLSANQLEALAPGTFAPLRALRNLSLAGNRLARLEPAALGALPLLRSLSLQDNELA

ALAPGLLGRLPALDALHLRGNPWGCGCALRPLCAWLRRHPLPASEAETVLCVWPGRLTLS

PLTAFSDAAFSHCAQPLALRDLAVVYTLGPASFLVSLASCLALGSGLTACRARRRRLRTA

ALRPPRPPDPNPDPDPHGCASPADPGSPAAAAQA

251.>sp|Q6H3X3|RET1G_HUMANRetinoicacidearlytranscript1GproteinOS=HomosapiensGN=RAET1GPE=1SV=1

MAAAASPAFLLRLPLLLLLSSWCRTGLADPHSLCYDITVIPKFRPGPRWCAVQGQVDEKT

FLHYDCGSKTVTPVSPLGKKLNVTTAWKAQNPVLREVVDILTEQLLDIQLENYIPKEPLT

LQARMSCEQKAEGHGSGSWQLSFDGQIFLLFDSENRMWTTVHPGARKMKEKWENDKDMTM

SFHYISMGDCTGWLEDFLMGMDSTLEPSAGAPPTMSSGTAQPRATATTLILCCLLIMCLL

ICSRHSLTQSHGHHPQSLQPPPHPPLLHPTWLLRRVLWSDSYQIAKRPLSGGHVTRVTLP

IIGDDSHSLPCPLALYTINNGAARYSEPLQVSIS

252.>sp|O94766|B3GA3_HUMANGalactosylgalactosylxylosylprotein3-beta-glucuronosyltransferase3OS=HomosapiensGN=B3GAT3PE=1SV=2

MKLKLKNVFLAYFLVSIAGLLYALVQLGQPCDCLPPLRAAAEQLRQKDLRISQLQAELRR

PPPAPAQPPEPEALPTIYVVTPTYARLVQKAELVRLSQTLSLVPRLHWLLVEDAEGPTPL

VSGLLAASGLLFTHLVVLTPKAQRLREGEPGWVHPRGVEQRNKALDWLRGRGGAVGGEKD

PPPPGTQGVVYFADDDNTYSRELFEEMRWTRGVSVWPVGLVGGLRFEGPQVQDGRVVGFH

TAWEPSRPFPVDMAGFAVALPLLLDKPNAQFDSTAPRGHLESSLLSHLVDPKDLEPRAAN

CTRVLVWHTRTEKPKMKQEEQLQRQGRGSDPAIEV

253.>sp|P09668|CATH_HUMANPro-cathepsinHOS=HomosapiensGN=CTSHPE=1SV=4

MWATLPLLCAGAWLLGVPVCGAAELCVNSLEKFHFKSWMSKHRKTYSTEEYHHRLQTFAS

NWRKINAHNNGNHTFKMALNQFSDMSFAEIKHKYLWSEPQNCSATKSNYLRGTGPYPPSV

DWRKKGNFVSPVKNQGACGSCWTFSTTGALESAIAIATGKMLSLAEQQLVDCAQDFNNHG

CQGGLPSQAFEYILYNKGIMGEDTYPYQGKDGYCKFQPGKAIGFVKDVANITIYDEEAMV

EAVALYNPVSFAFEVTQDFMMYRTGIYSSTSCHKTPDKVNHAVLAVGYGEKNGIPYWIVK

NSWGPQWGMNGYFLIERGKNMCGLAACASYPIPLV

254.>sp|P15813|CD1D_HUMANAntigen-presentingglycoproteinCD1dOS=HomosapiensGN=CD1DPE=1SV=1

MGCLLFLLLWALLQAWGSAEVPQRLFPLRCLQISSFANSSWTRTDGLAWLGELQTHSWSN

DSDTVRSLKPWSQGTFSDQQWETLQHIFRVYRSSFTRDVKEFAKMLRLSYPLELQVSAGC

EVHPGNASNNFFHVAFQGKDILSFQGTSWEPTQEAPLWVNLAIQVLNQDKWTRETVQWLL

NGTCPQFVSGLLESGKSELKKQVKPKAWLSRGPSPGPGRLLLVCHVSGFYPKPVWVKWMR

GEQEQQGTQPGDILPNADETWYLRATLDVVAGEAAGLSCRVKHSSLEGQDIVLYWGGSYT

SMGLIALAVLACLLFLLIVGFTSRFKRQTSYQGVL

255.>sp|P25445|TNR6_HUMANTumornecrosisfactorreceptorsuperfamilymember6OS=HomosapiensGN=FASPE=1SV=1

MLGIWTLLPLVLTSVARLSSKSVNAQVTDINSKGLELRKTVTTVETQNLEGLHHDGQFCH

KPCPPGERKARDCTVNGDEPDCVPCQEGKEYTDKAHFSSKCRRCRLCDEGHGLEVEINCT

RTQNTKCRCKPNFFCNSTVCEHCDPCTKCEHGIIKECTLTSNTKCKEEGSRSNLGWLCLL

LLPIPLIVWVKRKEVQKTCRKHRKENQGSHESPTLNPETVAINLSDVDLSKYITTIAGVM

TLSQVKGFVRKNGVNEAKIDEIKNDNVQDTAEQKVQLLRNWHQLHGKKEAYDTLIKDLKK

ANLCTLAEKIQTIILKDITSDSENSNFRNEIQSLV

256.>sp|Q03405|UPAR_HUMANUrokinaseplasminogenactivatorsurfacereceptorOS=HomosapiensGN=PLAURPE=1SV=1

MGHPPLLPLLLLLHTCVPASWGLRCMQCKTNGDCRVEECALGQDLCRTTIVRLWEEGEEL

ELVEKSCTHSEKTNRTLSYRTGLKITSLTEVVCGLDLCNQGNSGRAVTYSRSRYLECISC

GSSDMSCERGRHQSLQCRSPEEQCLDVVTHWIQEGEEGRPKDDRHLRGCGYLPGCPGSNG

FHNNDTFHFLKCCNTTKCNEGPILELENLPQNGRQCYSCKGNSTHGCSSEETFLIDCRGP

MNQCLVATGTHEPKNQSYMVRGCATASMCQHAHLGDAFSMNHIDVSCCTKSGCNHPDLDV

QYRSGAAPQPGPAHLSLTITLLMTARLWGGTLLWT

(1^st^NinO.R)

257.>sp|Q16570|ACKR1_HUMANAtypicalchemokinereceptor1OS=HomosapiensGN=ACKR1PE=1SV=3

MGNCLHRAELSPSTENSSQLDFEDVWNSSYGVNDSFPDGDYGANLEAAAPCHSCNLLDDS

ALPFFILTSVLGILASSTVLFMLFRPLFRWQLCPGWPVLAQLAVGSALFSIVVPVLAPGL

GSTRSSALCSLGYCVWYGSAFAQALLLGCHASLGHRLGAGQVPGLTLGLTVGIWGVAALL

TLPVTLASGASGGLCTLIYSTELKALQATHTVACLAIFVLLPLGLFGAKGLKKALGMGPG

PWMNILWAWFIFWWPHGVVLGLDFLVRSKLLLLSTCLAQQALDLLLNLAEALAILHCVAT

PLLLALFCHQATRTLLPSLPLPEGWSSHLDTLGSKS

258.>sp|O00180|KCNK1_HUMANPotassiumchannelsubfamilyKmember1OS=HomosapiensGN=KCNK1PE=1SV=1

MLQSLAGSSCVRLVERHRSAWCFGFLVLGYLLYLVFGAVVFSSVELPYEDLLRQELRKLK

RRFLEEHECLSEQQLEQFLGRVLEASNYGVSVLSNASGNWNWDFTSALFFASTVLSTTGY

GHTVPLSDGGKAFCIIYSVIGIPFTLLFLTAVVQRITVHVTRRPVLYFHIRWGFSKQVVA

IVHAVLLGFVTVSCFFFIPAAVFSVLEDDWNFLESFYFCFISLSTIGLGDYVPGEGYNQK

FRELYKIGITCYLLLGLIAMLVVLETFCELHELKKFRKMFYVKKDKDEDQVHIIEHDQLS

FSSITDQAAGMKEDQKQNEPFVATQSSACVDGPANH

259.>sp|Q9ULX7|CAH14_HUMANCarbonicanhydrase14OS=HomosapiensGN=CA14PE=1SV=1

MLFSALLLEVIWILAADGGQHWTYEGPHGQDHWPASYPECGNNAQSPIDIQTDSVTFDPD

LPALQPHGYDQPGTEPLDLHNNGHTVQLSLPSTLYLGGLPRKYVAAQLHLHWGQKGSPGG

SEHQINSEATFAELHIVHYDSDSYDSLSEAAERPQGLAVLGILIEVGETKNIAYEHILSH

LHEVRHKDQKTSVPPFNLRELLPKQLGQYFRYNGSLTTPPCYQSVLWTVFYRRSQISMEQ

LEKLQGTLFSTEEEPSKLLVQNYRALQPLNQRMVFASFIQAGSSYTTGEMLSLGVGILVG

CLCLLLAVYFIARKIRKKRLENRKSVVFTSAQATTEA

260.>sp|P51884|LUM_HUMANLumicanOS=HomosapiensGN=LUMPE=1SV=2

MSLSAFTLFLALIGGTSGQYYDYDFPLSIYGQSSPNCAPECNCPESYPSAMYCDELKLKS

VPMVPPGIKYLYLRNNQIDHIDEKAFENVTDLQWLILDHNLLENSKIKGRVFSKLKQLKK

LHINHNNLTESVGPLPKSLEDLQLTHNKITKLGSFEGLVNLTFIHLQHNRLKEDAVSAAF

KGLKSLEYLDLSFNQIARLPSGLPVSLLTLYLDNNKISNIPDEYFKRFNALQYLRLSHNE

LADSGIPGNSFNVSSLVELDLSYNKLKNIPTVNENLENYYLEVNQLEKFDIKSFCKILGP

LSYSKIKHLRLDGNRISETSLPPDMYECLRVANEVTLN

261.>sp|Q13449|LSAMP_HUMANLimbicsystem-associatedmembraneproteinOS=HomosapiensGN=LSAMPPE=1SV=2

MVRRVQPDRKQLPLVLLRLLCLLPTGLPVRSVDFNRGTDNITVRQGDTAILRCVVEDKNS

KVAWLNRSGIIFAGHDKWSLDPRVELEKRHSLEYSLRIQKVDVYDEGSYTCSVQTQHEPK

TSQVYLIVQVPPKISNISSDVTVNEGSNVTLVCMANGRPEPVITWRHLTPTGREFEGEEE

YLEILGITREQSGKYECKAANEVSSADVKQVKVTVNYPPTITESKSNEATTGRQASLKCE

ASAVPAPDFEWYRDDTRINSANGLEIKSTEGQSSLTVTNVTEEHYGNYTCVAANKLGVTN

ASLVLFRPGSVRGINGSISLAVPLWLLAASLLCLLSKC

(AllNinO.R.)

262.>sp|P07858|CATB_HUMANCathepsinBOS=HomosapiensGN=CTSBPE=1SV=3

MWQLWASLCCLLVLANARSRPSFHPLSDELVNYVNKRNTTWQAGHNFYNVDMSYLKRLCG

TFLGGPKPPQRVMFTEDLKLPASFDAREQWPQCPTIKEIRDQGSCGSCWAFGAVEAISDR

ICIHTNAHVSVEVSAEDLLTCCGSMCGDGCNGGYPAEAWNFWTRKGLVSGGLYESHVGCR

PYSIPPCEHHVNGSRPPCTGEGDTPKCSKICEPGYSPTYKQDKHYGYNSYSVSNSEKDIM

AEIYKNGPVEGAFSVYSDFLLYKSGVYQHVTGEMMGGHAIRILGWGVENGTPYWLVANSW

NTDWGDNGFFKILRGQDHCGIESEVVAGIPRTDQYWEKI

263.>sp|O94905|ERLN2_HUMANErlin-2OS=HomosapiensGN=ERLIN2PE=1SV=1

MAQLGAVVAVASSFFCASLFSAVHKIEEGHIGVYYRGGALLTSTSGPGFHLMLPFITSYK

SVQTTLQTDEVKNVPCGTSGGVMIYFDRIEVVNFLVPNAVYDIVKNYTADYDKALIFNKI

HHELNQFCSVHTLQEVYIELFDQIDENLKLALQQDLTSMAPGLVIQAVRVTKPNIPEAIR

RNYELMESEKTKLLIAAQKQKVVEKEAETERKKALIEAEKVAQVAEITYGQKVMEKETEK

KISEIEDAAFLAREKAKADAECYTAMKIAEANKLKLTPEYLQLMKYKAIASNSKIYFGKD

IPNMFMDSAGSVSKQFEGLADKLSFGLEDEPLETATKEN

264.>sp|Q9HCN6|GPVI_HUMANPlateletglycoproteinVIOS=HomosapiensGN=GP6PE=1SV=4

MSPSPTALFCLGLCLGRVPAQSGPLPKPSLQALPSSLVPLEKPVTLRCQGPPGVDLYRLE

KLSSSRYQDQAVLFIPAMKRSLAGRYRCSYQNGSLWSLPSDQLELVATGVFAKPSLSAQP

GPAVSSGGDVTLQCQTRYGFDQFALYKEGDPAPYKNPERWYRASFPIITVTAAHSGTYRC

YSFSSRDPYLWSAPSDPLELVVTGTSVTPSRLPTEPPSPVAEFSEATAELTVSFTNEVFT

TETSRSITASPKESDSPAGPARQYYTKGNLVRICLGAVILIILAGFLAEDWHSRRKRLRH

RGRAVQRPLPPLPPLPLTRKSNGGQDGGRQDVHSRGLCS

265.>sp|P01877|IGHA2_HUMANIgalpha-2chainCregionOS=HomosapiensGN=IGHA2PE=1SV=3

ASPTSPKVFPLSLDSTPQDGNVVVACLVQGFFPQEPLSVTWSESGQNVTARNFPPSQDAS

GDLYTTSSQLTLPATQCPDGKSVTCHVKHYTNPSQDVTVPCPVPPPPPCCHPRLSLHRPA

LEDLLLGSEANLTCTLTGLRDASGATFTWTPSSGKSAVQGPPERDLCGCYSVSSVLPGCA

QPWNHGETFTCTAAHPELKTPLTANITKSGNTFRPEVHLLPPPSEELALNELVTLTCLAR

GFSPKDVLVRWLQGSQELPREKYLTWASRQEPSQGTTTFAVTSILRVAAEDWKKGDTFSC

MVGHEALPLAFTQKTIDRMAGKPTHVNVSVVMAEVDGTCY

266.>sp|Q95460|HMR1_HUMANMajorhistocompatibilitycomplexclassI-relatedgeneproteinOS=HomosapiensGN=MR1PE=1SV=1

MGELMAFLLPLIIVLMVKHSDSRTHSLRYFRLGVSDPIHGVPEFISVGYVDSHPITTYDS

VTRQKEPRAPWMAENLAPDHWERYTQLLRGWQQMFKVELKRLQRHYNHSGSHTYQRMIGC

ELLEDGSTTGFLQYAYDGQDFLIFNKDTLSWLAVDNVAHTIKQAWEANQHELLYQKNWLE

EECIAWLKRFLEYGKDTLQRTEPPLVRVNRKETFPGVTALFCKAHGFYPPEIYMTWMKNG

EEIVQEIDYGDILPSGDGTYQAWASIELDPQSSNLYSCHVEHCGVHMVLQVPQESETIPL

VMKAVSGSIVLVIVLAGVGVLVWRRRPREQNGAIYLPTPDR

267.>sp|P21731|TA2R_HUMANThromboxaneA2receptorOS=HomosapiensGN=TBXA2RPE=1SV=3

MWPNGSSLGPCFRPTNITLEERRLIASPWFAASFCVVGLASNLLALSVLAGARQGGSHTR

SSFLTFLCGLVLTDFLGLLVTGTIVVSQHAALFEWHAVDPGCRLCRFMGVVMIFFGLSPL

LLGAAMASERYLGITRPFSRPAVASQRRAWATVGLVWAAALALGLLPLLGVGRYTVQYPG

SWCFLTLGAESGDVAFGLLFSMLGGLSVGLSFLLNTVSVATLCHVYHGQEAAQQRPRDSE

VEMMAQLLGIMVVASVCWLPLLVFIAQTVLRNPPAMSPAGQLSRTTEKELLIYLRVATWN

QILDPWVYILFRRAVLRRLQPRLSTRPRSLSLQPQLTQRSGLQ

268.>sp|P40199|CEAM6_HUMANCarcinoembryonicantigen-relatedcelladhesionmolecule6OS=HomosapiensGN=CEACAM6PE=1SV=3

MGPPSAPPCRLHVPWKEVLLTASLLTFWNPPTTAKLTIESTPFNVAEGKEVLLLAHNLPQ

NRIGYSWYKGERVDGNSLIVGYVIGTQQATPGPAYSGRETIYPNASLLIQNVTQNDTGFY

TLQVIKSDLVNEEATGQFHVYPELPKPSISSNNSNPVEDKDAVAFTCEPEVQNTTYLWWV

NGQSLPVSPRLQLSNGNMTLTLLSVKRNDAGSYECEIQNPASANRSDPVTLNVLYGPDGP

TISPSKANYRPGENLNLSCHAASNPPAQYSWFINGTFQQSTQELFIPNITVNNSGSYMCQ

AHNSATGLNRTTVTMITVSGSAPVLSAVATVGITIGVLARVALI

269.>sp|P02749|APOH_HUMANBeta-2-glycoprotein1OS=HomosapiensGN=APOHPE=1SV=3

MISPVLILFSSFLCHVAIAGRTCPKPDDLPFSTVVPLKTFYEPGEEITYSCKPGYVSRGG

MRKFICPLTGLWPINTLKCTPRVCPFAGILENGAVRYTTFEYPNTISFSCNTGFYLNGAD

SAKCTEEGKWSPELPVCAPIICPPPSIPTFATLRVYKPSAGNNSLYRDTAVFECLPQHAM

FGNDTITCTTHGNWTKLPECREVKCPFPSRPDNGFVNYPAKPTLYYKDKATFGCHDGYSL

DGPEEIECTKLGNWSAMPSCKASCKVPVKKATVVYQGERVKIQEKFKNGMLHGDKVSFFC

KNKEKKCSYTEDAQCIDGTIEVPKCFKEHSSLAFWKTDASDVKPC

270.>sp|P20933|ASPG_HUMANN(4)-(beta-N-acetylglucosaminyl)-L-asparaginaseOS=HomosapiensGN=AGAPE=1SV=2

MARKSNLPVLLVPFLLCQALVRCSSPLPLVVNTWPFKNATEAAWRALASGGSALDAVESG

CAMCEREQCDGSVGFGGSPDELGETTLDAMIMDGTTMDVGAVGDLRRIKNAIGVARKVLE

HTTHTLLVGESATTFAQSMGFINEDLSTTASQALHSDWLARNCQPNYWRNVIPDPSKYCG

PYKPPGILKQDIPIHKETEDDRGHDTIGMVVIHKTGHIAAGTSTNGIKFKIHGRVGDSPI

PGAGAYADDTAGAAAATGNGDILMRFLPSYQAVEYMRRGEDPTIACQKVISRIQKHFPEF

FGAVICANVTGSYGAACNKLSTFTQFSFMVYNSEKNQPTEEKVDCI

271.>sp|Q8IU99|CAHM1_HUMANCalciumhomeostasismodulatorprotein1OS=HomosapiensGN=CALHM1PE=1SV=2

MMDKFRMIFQFLQSNQESFMNGICGIMALASAQMYSAFDFNCPCLPGYNAAYSAGILLAP

PLVLFLLGLVMNNNVSMLAEEWKRPLGRRAKDPAVLRYMFCSMAQRALIAPVVWVAVTLL

DGKCFLCAFCTAVPVSALGNGSLAPGLPAPELARLLARVPCPEIYDGDWLLAREVAVRYL

RCISQALGWSFVLLTTLLAFVVRSVRPCFTQAAFLKSKYWSHYIDIERKLFDETCTEHAK

AFAKVCIQQFFEAMNHDLELGHTHGTLATAPASAAAPTTPDGAEEEREKLRGITDQGTMN

RLLTSWHKCKPPLRLGQEEPPLMGNGWAGGGPRPPRKEVATYFSKV

272.>sp|O75477|ERLN1_HUMANErlin-1OS=HomosapiensGN=ERLIN1PE=1SV=1

MTQARVLVAAVVGLVAVLLYASIHKIEEGHLAVYYRGGALLTSPSGPGYHIMLPFITTFR

SVQTTLQTDEVKNVPCGTSGGVMIYIDRIEVVNMLAPYAVFDIVRNYTADYDKTLIFNKI

HHELNQFCSAHTLQEVYIELFDQIDENLKQALQKDLNLMAPGLTIQAVRVTKPKIPEAIR

RNFELMEAEKTKLLIAAQKQKVVEKEAETERKKAVIEAEKIAQVAKIRFQQKVMEKETEK

RISEIEDAAFLAREKAKADAEYYAAHKYATSNKHKLTPEYLELKKYQAIASNSKIYFGSN

IPNMFVDSSCALKYSDIRTGRESSLPSKEALEPSGENVIQNKESTG

273.>sp|Q6ZSG1|RN165_HUMANRINGfingerprotein165OS=HomosapiensGN=RNF165PE=1SV=1

MVLVHVGYLVLPVFGSVRNRGAPFQRSQHPHATSCRHFHLGPPQPQQLAPDFPLAHPVQS

QPGLSAHMAPAHQHSGALHQSLTPLPTLQFQDVTGPSFLPQALHQQYLLQQQLLEAQHRR

LVSHPRRSQERVSVHPHRLHPSFDFGQLQTPQPRYLAEGTDWDLSVDAGLSPAQFQVRPI

PQHYQHYLATPRMHHFPRNSSSTQMVVHEIRNYPYPQLHFLALQGLNPSRHTSAVRESYE

ELLQLEDRLGNVTRGAVQNTIERFTFPHKYKKRRPQDGKGKKDEGEESDTDEKCTICLSM

LEDGEDVRRLPCMHLFHQLCVDQWLAMSKKCPICRVDIETQLGADS

274.>sp|P02750|A2GL_HUMANLeucine-richalpha-2-glycoproteinOS=HomosapiensGN=LRG1PE=1SV=2

MSSWSRQRPKSPGGIQPHVSRTLFLLLLLAASAWGVTLSPKDCQVFRSDHGSSISCQPPA

EIPGYLPADTVHLAVEFFNLTHLPANLLQGASKLQELHLSSNGLESLSPEFLRPVPQLRV

LDLTRNALTGLPPGLFQASATLDTLVLKENQLEVLEVSWLHGLKALGHLDLSGNRLRKLP

PGLLANFTLLRTLDLGENQLETLPPDLLRGPLQLERLHLEGNKLQVLGKDLLLPQPDLRY

LFLNGNKLARVAAGAFQGLRQLDMLDLSNNSLASVPEGLWASLGQPNWDMRDGFDISGNP

WICDQNLSDLYRWLQAQKDKMFSQNDTRCAGPEAVKGQTLLAVAKSQ

275.>sp|Q8IW45|NNRD_HUMANATP-dependent(S)-NAD(P)H-hydratedehydrataseOS=HomosapiensGN=CARKDPE=1SV=1

MVTRAGAGTAVAGAVVVALLSAALALYGPPLDAVLERAFSLRKAHSIKDMENTLQLVRNI

IPPLSSTKHKGQDGRIGVVGGCQEYTGAPYFAAISALKVGADLSHVFCASAAAPVIKAYS

PELIVHPVLDSPNAVHEVEKWLPRLHALVVGPGLGRDDALLRNVQGILEVSKARDIPVVI

DADGLWLVAQQPALIHGYRKAVLTPNHVEFSRLYDAVLRGPMDSDDSHGSVLRLSQALGN

VTVVQKGERDILSNGQQVLVCSQEGSSRRCGGQGDLLSGSLGVLVHWALLAGPQKTNGSS

PLLVAAFGACSLTRQCNHQAFQKHGRSTTTSDMIAEVGAAFSKLFET

276.>sp|Q9H0V9|LMA2L_HUMANVIP36-likeproteinOS=HomosapiensGN=LMAN2LPE=1SV=1

MAATLGPLGSWQQWRRCLSARDGSRMLLLLLLLGSGQGPQQVGAGQTFEYLKREHSLSKP

YQGVGTGSSSLWNLMGNAMVMTQYIRLTPDMQSKQGALWNRVPCFLRDWELQVHFKIHGQ

GKKNLHGDGLAIWYTKDRMQPGPVFGNMDKFVGLGVFVDTYPNEEKQQERVFPYISAMVN

NGSLSYDHERDGRPTELGGCTAIVRNLHYDTFLVIRYVKRHLTIMMDIDGKHEWRDCIEV

PGVRLPRGYYFGTSSITGDLSDNHDVISLKLFELTVERTPEEEKLHRDVFLPSVDNMKLP

EMTAPLPPLSGLALFLIVFFSLVFSVFAIVIGIILYNKWQEQSRKRFY

277.>sp|Q12908|NTCP2_HUMANIlealsodium/bileacidcotransporterOS=HomosapiensGN=SLC10A2PE=1SV=2

MNDPNSCVDNATVCSGASCVVPESNFNNILSVVLSTVLTILLALVMFSMGCNVEIKKFLG

HIKRPWGICVGFLCQFGIMPLTGFILSVAFDILPLQAVVVLIIGCCPGGTASNILAYWVD

GDMDLSVSMTTCSTLLALGMMPLCLLIYTKMWVDSGSIVIPYDNIGTSLVSLVVPVSIGM

FVNHKWPQKAKIILKIGSIAGAILIVLIAVVGGILYQSAWIIAPKLWIIGTIFPVAGYSL

GFLLARIAGLPWYRCRTVAFETGMQNTQLCSTIVQLSFTPEELNVVFTFPLIYSIFQLAF

AAIFLGFYVAYKKCHGKNKAEIPESKENGTEPESSFYKANGGFQPDEK

(NinO.R.)

278.>sp|P31997|CEAM8_HUMANCarcinoembryonicantigen-relatedcelladhesionmolecule8OS=HomosapiensGN=CEACAM8PE=1SV=2

MGPISAPSCRWRIPWQGLLLTASLFTFWNPPTTAQLTIEAVPSNAAEGKEVLLLVHNLPQ

DPRGYNWYKGETVDANRRIIGYVISNQQITPGPAYSNRETIYPNASLLMRNVTRNDTGSY

TLQVIKLNLMSEEVTGQFSVHPETPKPSISSNNSNPVEDKDAVAFTCEPETQNTTYLWWV

NGQSLPVSPRLQLSNGNRTLTLLSVTRNDVGPYECEIQNPASANFSDPVTLNVLYGPDAP

TISPSDTYYHAGVNLNLSCHAASNPPSQYSWSVNGTFQQYTQKLFIPNITTKNSGSYACH

TTNSATGRNRTTVRMITVSDALVQGSSPGLSARATVSIMIGVLARVALI

279.>sp|Q70UQ0|IKIP_HUMANInhibitorofnuclearfactorkappa-Bkinase-interactingproteinOS=HomosapiensGN=IKBIPPE=1SV=1

MSEVKSRKKSGPKGAPAAEPGKRSEGGKTPVARSSGGGGWADPRTCLSLLSLGTCLGLAW

FVFQQSEKFAKVENQYQLLKLETNEFQQLQSKISLISEKWQKSEAIMEQLKSFQIIAHLK

RLQEEINEVKTWSNRITEKQDILNNSLTTLSQDITKVDQSTTSMAKDVGLKITSVKTDIR

RISGLVTDVISLTDSVQELENKIEKVEKNTVKNIGDLLSSSIDRTATLRKTASENSQRIN

SVKKTLTELKSDFDKHTDRFLSLEGDRAKVLKTVTFANDLKPKVYNLKKDFSRLEPLVND

LTLRIGRLVTDLLQREKEIAFLSEKISNLTIVQAEIKDIKDEIAHISDMN

280.>sp|P58166|INHBE_HUMANInhibinbetaEchainOS=HomosapiensGN=INHBEPE=1SV=1

MRLPDVQLWLVLLWALVRAQGTGSVCPSCGGSKLAPQAERALVLELAKQQILDGLHLTSR

PRITHPPPQAALTRALRRLQPGSVAPGNGEEVISFATVTDSTSAYSSLLTFHLSTPRSHH

LYHARLWLHVLPTLPGTLCLRIFRWGPRRRRQGSRTLLAEHHITNLGWHTLTLPSSGLRG

EKSGVLKLQLDCRPLEGNSTVTGQPRRLLDTAGHQQPFLELKIRANEPGAGRARRRTPTC

EPATPLCCRRDHYVDFQELGWRDWILQPEGYQLNYCSGQCPPHLAGSPGIAASFHSAVFS

LLKANNPWPASTSCCVPTARRPLSLLYLDHNGNVVKTDVPDMVVEACGCS

281.>sp|Q9H813|TM206_HUMANTransmembraneprotein206OS=HomosapiensGN=TMEM206PE=1SV=1

MIRQERSTSYQELSEELVQVVENSELADEQDKETVRVQGPGILPGLDSESASSSIRFSKA

CLKNVFSVLLIFIYLLLMAVAVFLVYRTITDFREKLKHPVMSVSYKEVDRYDAPGIALYP

GQAQLLSCKHHYEVIPPLTSPGQPGDMNCTTQRINYTDPFSNQTVKSALIVQGPREVKKR

ELVFLQFRLNKSSEDFSAIDYLLFSSFQEFLQSPNRVGFMQACESAYSSWKFSGGFRTWV

KMSLVKTKEEDGREAVEFRQETSVVNYIDQRPAAKKSAQLFFVVFEWKDPFIQKVQDIVT

ANPWNTIALLCGAFLALFKAAEFAKLSIKWMIKIRKRYLKRRGQATSHIS

282.>sp|Q9GZM5|YIPF3_HUMANProteinYIPF3OS=HomosapiensGN=YIPF3PE=1SV=1

MATTAAPAGGARNGAGPEWGGFEENIQGGGSAVIDMENMDDTSGSSFEDMGELHQRLREE

EVDADAADAAAAEEEDGEFLGMKGFKGQLSRQVADQMWQAGKRQASRAFSLYANIDILRP

YFDVEPAQVRSRLLESMIPIKMVNFPQKIAGELYGPLMLVFTLVAILLHGMKTSDTIIRE

GTLMGTAIGTCFGYWLGVSSFIYFLAYLCNAQITMLQMLALLGYGLFGHCIVLFITYNIH

LHALFYLFWLLVGGLSTLRMVAVLVSRTVGPTQRLLLCGTLAALHMLFLLYLHFAYHKVV

EGILDTLEGPNIPPIQRVPRDIPAMLPAARLPTTVLNATAKAVAVTLQSH

283.>sp|P06729|CD2_HUMANT-cellsurfaceantigenCD2OS=HomosapiensGN=CD2PE=1SV=2

MSFPCKFVASFLLIFNVSSKGAVSKEITNALETWGALGQDINLDIPSFQMSDDIDDIKWE

KTSDKKKIAQFRKEKETFKEKDTYKLFKNGTLKIKHLKTDDQDIYKVSIYDTKGKNVLEK

IFDLKIQERVSKPKISWTCINTTLTCEVMNGTDPELNLYQDGKHLKLSQRVITHKWTTSL

SAKFKCTAGNKVSKESSVEPVSCPEKGLDIYLIIGICGGGSLLMVFVALLVFYITKRKKQ

RSRRNDEELETRAHRVATEERGRKPHQIPASTPQNPATSQHPPPPPGHRSQAPSHRPPPP

GHRVQHQPQKRPPAPSGTQVHQQKGPPLPRPRVQPKPPHGAAENSLSPSSN

284.>sp|P02760|AMBP_HUMANProteinAMBPOS=HomosapiensGN=AMBPPE=1SV=1

MRSLGALLLLLSACLAVSAGPVPTPPDNIQVQENFNISRIYGKWYNLAIGSTCPWLKKIM

DRMTVSTLVLGEGATEAEISMTSTRWRKGVCEETSGAYEKTDTDGKFLYHKSKWNITMES

YVVHTNYDEYAIFLTKKFSRHHGPTITAKLYGRAPQLRETLLQDFRVVAQGVGIPEDSIF

TMADRGECVPGEQEPEPILIPRVRRAVLPQEEEGSGGGQLVTEVTKKEDSCQLGYSAGPC

MGMTSRYFYNGTSMACETFQYGGCMGNGNNFVTEKECLQTCRTVAACNLPIVRGPCRAFI

QLWAFDAVKGKCVLFPYGGCQGNGNKFYSEKECREYCGVPGDGDEELLRFSN

285.>sp|P61073|CXCR4_HUMANC-X-Cchemokinereceptortype4OS=HomosapiensGN=CXCR4PE=1SV=1

MEGISIYTSDNYTEEMGSGDYDSMKEPCFREENANFNKIFLPTIYSIIFLTGIVGNGLVI

LVMGYQKKLRSMTDKYRLHLSVADLLFVITLPFWAVDAVANWYFGNFLCKAVHVIYTVNL

YSSVLILAFISLDRYLAIVHATNSQRPRKLLAEKVVYVGVWIPALLLTIPDFIFANVSEA

DDRYICDRFYPNDLWVVVFQFQHIMVGLILPGIVILSCYCIIISKLSHSKGHQKRKALKT

TVILILAFFACWLPYYIGISIDSFILLEIIKQGCEFENTVHKWISITEALAFFHCCLNPI

LYAFLGAKFKTSAQHALTSVSRGSSLKILSKGKRGGHSSVSTESESSSFHSS

286.>sp|O60938|KERA_HUMANKeratocanOS=HomosapiensGN=KERAPE=1SV=1

MAGTICFIMWVLFITDTVWSRSVRQVYEVHDSDDWTIHDFECPMECFCPPSFPTALYCEN

RGLKEIPAIPSRIWYLYLQNNLIETIPEKPFENATQLRWINLNKNKITNYGIEKGALSQL

KKLLFLFLEDNELEEVPSPLPRSLEQLQLARNKVSRIPQGTFSNLENLTLLDLQNNKLVD

NAFQRDTFKGLKNLMQLNMAKNALRNMPPRLPANTMQLFLDNNSIEGIPENYFNVIPKVA

FLRLNHNKLSDEGLPSRGFDVSSILDLQLSHNQLTKVPRISAHLQHLHLDHNKIKSVNVS

VICPSPSMLPAERDSFSYGPHLRYLRLDGNEIKPPIPMALMTCFRLLQAVII

287.>sp|P56704|WNT3A_HUMANProteinWnt-3aOS=HomosapiensGN=WNT3APE=1SV=2

MAPLGYFLLLCSLKQALGSYPIWWSLAVGPQYSSLGSQPILCASIPGLVPKQLRFCRNYV

EIMPSVAEGIKIGIQECQHQFRGRRWNCTTVHDSLAIFGPVLDKATRESAFVHAIASAGV

AFAVTRSCAEGTAAICGCSSRHQGSPGKGWKWGGCSEDIEFGGMVSREFADARENRPDAR

SAMNRHNNEAGRQAIASHMHLKCKCHGLSGSCEVKTCWWSQPDFRAIGDFLKDKYDSASE

MVVEKHRESRGWVETLRPRYTYFKVPTERDLVYYEASPNFCEPNPETGSFGTRDRTCNVS

SHGIDGCDLLCCGRGHNARAERRREKCRCVFHWCCYVSCQECTRVYDVHTCK

288.>sp|P01876|IGHA1_HUMANIgalpha-1chainCregionOS=HomosapiensGN=IGHA1PE=1SV=2

ASPTSPKVFPLSLCSTQPDGNVVIACLVQGFFPQEPLSVTWSESGQGVTARNFPPSQDAS

GDLYTTSSQLTLPATQCLAGKSVTCHVKHYTNPSQDVTVPCPVPSTPPTPSPSTPPTPSP

SCCHPRLSLHRPALEDLLLGSEANLTCTLTGLRDASGVTFTWTPSSGKSAVQGPPERDLC

GCYSVSSVLPGCAEPWNHGKTFTCTAAYPESKTPLTATLSKSGNTFRPEVHLLPPPSEEL

ALNELVTLTCLARGFSPKDVLVRWLQGSQELPREKYLTWASRQEPSQGTTTFAVTSILRV

AAEDWKKGDTFSCMVGHEALPLAFTQKTIDRLAGKPTHVNVSVVMAEVDGTCY

289.>sp|P40225|TPO_HUMANThrombopoietinOS=HomosapiensGN=THPOPE=1SV=1

MELTELLLVVMLLLTARLTLSSPAPPACDLRVLSKLLRDSHVLHSRLSQCPEVHPLPTPV

LLPAVDFSLGEWKTQMEETKAQDILGAVTLLLEGVMAARGQLGPTCLSSLLGQLSGQVRL

LLGALQSLLGTQLPPQGRTTAHKDPNAIFLSFQHLLRGKVRFLMLVGGSTLCVRRAPPTT

AVPSRTSLVLTLNELPNRTSGLLETNFTASARTTGSGLLKWQQGFRAKIPGLLNQTSRSL

DQIPGYLNRIHELLNGTRGLFPGPSRRTLGAPDISSGTSDTGSLPPNLQPGYSPSPTHPP

TGQYTLFPLPPTLPTPVVQLHPLLPDPSAPTPTPTSPLLNTSYTHSQNLSQEG

290.>sp|P34810|CD68_HUMANMacrosialinOS=HomosapiensGN=CD68PE=1SV=2

MRLAVLFSGALLGLLAAQGTGNDCPHKKSATLLPSFTVTPTVTESTGTTSHRTTKSHKTT

THRTTTTGTTSHGPTTATHNPTTTSHGNVTVHPTSNSTATSQGPSTATHSPATTSHGNAT

VHPTSNSTATSPGFTSSAHPEPPPPSPSPSPTSKETIGDYTWTNGSQPCVHLQAQIQIRV

MYTTQGGGEAWGISVLNPNKTKVQGSCEGAHPHLLLSFPYGHLSFGFMQDLQQKVVYLSY

MAVEYNVSFPHAAQWTFSAQNASLRDLQAPLGQSFSCSNSSIILSPAVHLDLLSLRLQAA

QLPHTGVFGQSFSCPSDRSILLPLIIGLILLGLLALVLIAFCIIRRRPSAYQAL

291.>sp|Q15165|PON2_HUMANSerumparaoxonase/arylesterase2OS=HomosapiensGN=PON2PE=1SV=3

MGRLVAVGLLGIALALLGERLLALRNRLKASREVESVDLPHCHLIKGIEAGSEDIDILPN

GLAFFSVGLKFPGLHSFAPDKPGGILMMDLKEEKPRARELRISRGFDLASFNPHGISTFI

DNDDTVYLFVVNHPEFKNTVEIFKFEEAENSLLHLKTVKHELLPSVNDITAVGPAHFYAT

NDHYFSDPFLKYLETYLNLHWANVVYYSPNEVKVVAEGFDSANGINISPDDKYIYVADIL

AHEIHVLEKHTNMNLTQLKVLELDTLVDNLSIDPSSGDIWVGCHPNGQKLFVYDPNNPPS

SEVLRIQNILCEKPTVTTVYANNGSVLQGSSVASVYDGKLLIGTLYHRALYCEL

292.>sp|Q15166|PON3_HUMANSerumparaoxonase/lactonase3OS=HomosapiensGN=PON3PE=1SV=3

MGKLVALVLLGVGLSLVGEMFLAFRERVNASREVEPVEPENCHLIEELESGSEDIDILPS

GLAFISSGLKYPGMPNFAPDEPGKIFLMDLNEQNPRAQALEISGGFDKELFNPHGISIFI

DKDNTVYLYVVNHPHMKSTVEIFKFEEQQRSLVYLKTIKHELLKSVNDIVVLGPEQFYAT

RDHYFTNSLLSFFEMILDLRWTYVLFYSPREVKVVAKGFCSANGITVSADQKYVYVADVA

AKNIHIMEKHDNWDLTQLKVIQLGTLVDNLTVDPATGDILAGCHPNPMKLLNYNPEDPPG

SEVLRIQNVLSEKPRVSTVYANNGSVLQGTSVASVYHGKILIGTVFHKTLYCEL(NinO.R.)

293.>sp|O43915|VEGFD_HUMANVascularendothelialgrowthfactorDOS=HomosapiensGN=FIGFPE=1SV=1

MYREWVVVNVFMMLYVQLVQGSSNEHGPVKRSSQSTLERSEQQIRAASSLEELLRITHSE

DWKLWRCRLRLKSFTSMDSRSASHRSTRFAATFYDIETLKVIDEEWQRTQCSPRETCVEV

ASELGKSTNTFFKPPCVNVFRCGGCCNEESLICMNTSTSYISKQLFEISVPLTSVPELVP

VKVANHTGCKCLPTAPRHPYSIIRRSIQIPEEDRCSHSKKLCPIDMLWDSNKCKCVLQEE

NPLAGTEDHSHLQEPALCGPHMMFDEDRCECVCKTPCPKDLIQHPKNCSCFECKESLETC

CQKHKLFHPDTCSCEDRCPFHTRPCASGKTACAKHCRFPKEKRAAQGPHSRKNP

294.>sp|Q9NQR9|G6PC2_HUMANGlucose-6-phosphatase2OS=HomosapiensGN=G6PC2PE=1SV=1

MDFLHRNGVLIIQHLQKDYRAYYTFLNFMSNVGDPRNIFFIYFPLCFQFNQTVGTKMIWV

AVIGDWLNLIFKWILFGHRPYWWVQETQIYPNHSSPCLEQFPTTCETGPGSPSGHAMGAS

CVWYVMVTAALSHTVCGMDKFSITLHRLTWSFLWSVFWLIQISVCISRVFIATHFPHQVI

LGVIGGMLVAEAFEHTPGIQTASLGTYLKTNLFLFLFAVGFYLLLRVLNIDLLWSVPIAK

KWCANPDWIHIDTTPFAGLVRNLGVLFGLGFAINSEMFLLSCRGGNNYTLSFRLLCALTS

LTILQLYHFLQIPTHEEHLFYVLSFCKSASIPLTVVAFIPYSVHMLMKQSGKKSQ

295.>sp|P27169|PON1_HUMANSerumparaoxonase/arylesterase1OS=HomosapiensGN=PON1PE=1SV=3

MAKLIALTLLGMGLALFRNHQSSYQTRLNALREVQPVELPNCNLVKGIETGSEDLEILPN

GLAFISSGLKYPGIKSFNPNSPGKILLMDLNEEDPTVLELGITGSKFDVSSFNPHGISTF

TDEDNAMYLLVVNHPDAKSTVELFKFQEEEKSLLHLKTIRHKLLPNLNDIVAVGPEHFYG

TNDHYFLDPYLQSWEMYLGLAWSYVVYYSPSEVRVVAEGFDFANGINISPDGKYVYIAEL

LAHKIHVYEKHANWTLTPLKSLDFNTLVDNISVDPETGDLWVGCHPNGMKIFFYDSENPP

ASEVLRIQNILTEEPKVTQVYAENGTVLQGSTVASVYKGKLLIGTVFHKALYCEL

296.>sp|Q12907|LMAN2_HUMANVesicularintegral-membraneproteinVIP36OS=HomosapiensGN=LMAN2PE=1SV=1

MAAEGWIWRWGWGRRCLGRPGLLGPGPGPTTPLFLLLLLGSVTADITDGNSEHLKREHSL

IKPYQGVGSSSMPLWDFQGSTMLTSQYVRLTPDERSKEGSIWNHQPCFLKDWEMHVHFKV

HGTGKKNLHGDGIALWYTRDRLVPGPVFGSKDNFHGLAIFLDTYPNDETTERVFPYISVM

VNNGSLSYDHSKDGRWTELAGCTADFRNRDHDTFLAVRYSRGRLTVMTDLEDKNEWKNCI

DITGVRLPTGYYFGASAGTGDLSDNHDIISMKLFQLMVEHTPDEESIDWTKIEPSVNFLK

SPKDNVDDPTGNFRSGPLTGWRVFLLLLCALLGIVVCAVVGAVVFQKRQERNKRFY

297.>sp|P30533|AMRP_HUMANAlpha-2-macroglobulinreceptor-associatedproteinOS=HomosapiensGN=LRPAP1PE=1SV=1

MAPRRVRSFLRGLPALLLLLLFLGPWPAASHGGKYSREKNQPKPSPKRESGEEFRMEKLN

QLWEKAQRLHLPPVRLAELHADLKIQERDELAWKKLKLDGLDEDGEKEARLIRNLNVILA

KYGLDGKKDARQVTSNSLSGTQEDGLDDPRLEKLWHKAKTSGKFSGEELDKLWREFLHHK

EKVHEYNVLLETLSRTEEIHENVISPSDLSDIKGSVLHSRHTELKEKLRSINQGLDRLRR

VSHQGYSTEAEFEEPRVIDLWDLAQSANLTDKELEAFREELKHFEAKIEKHNHYQKQLEI

AHEKLRHAESVGDGERVSRSREKHALLEGRTKELGYTVKKHLQDLSGRISRARHNEL

298.>sp|P35575|G6PC_HUMANGlucose-6-phosphataseOS=HomosapiensGN=G6PCPE=1SV=2

MEEGMNVLHDFGIQSTHYLQVNYQDSQDWFILVSVIADLRNAFYVLFPIWFHLQEAVGIK

LLWVAVIGDWLNLVFKWILFGQRPYWWVLDTDYYSNTSVPLIKQFPVTCETGPGSPSGHA

MGTAGVYYVMVTSTLSIFQGKIKPTYRFRCLNVILWLGFWAVQLNVCLSRIYLAAHFPHQ

VVAGVLSGIAVAETFSHIHSIYNASLKKYFLITFFLFSFAIGFYLLLKGLGVDLLWTLEK

AQRWCEQPEWVHIDTTPFASLLKNLGTLFGLGLALNSSMYRESCKGKLSKWLPFRLSSIV

ASLVLLHVFDSLKPPSQVELVFYVLSFCKSAVVPLASVSVIPYCLAQVLGQPHKKSL

299.>sp|Q9UBS4|DJB11_HUMANDnaJhomologsubfamilyBmember11OS=HomosapiensGN=DNAJB11PE=1SV=1

MAPQNLSTFCLLLLYLIGAVIAGRDFYKILGVPRSASIKDIKKAYRKLALQLHPDRNPDD

PQAQEKFQDLGAAYEVLSDSEKRKQYDTYGEEGLKDGHQSSHGDIFSHFFGDFGFMFGGT

PRQQDRNIPRGSDIIVDLEVTLEEVYAGNFVEVVRNKPVARQAPGKRKCNCRQEMRTTQL

GPGRFQMTQEVVCDECPNVKLVNEERTLEVEIEPGVRDGMEYPFIGEGEPHVDGEPGDLR

FRIKVVKHPIFERRGDDLYTNVTISLVESLVGFEMDITHLDGHKVHISRDKITRPGAKLW

KKGEGLPNFDNNNIKGSLIITFDVDFPKEQLTEEAREGIKQLLKQGSVQKVYNGLQGY

300.>sp|P13747|HLAE_HUMANHLAclassIhistocompatibilityantigen,alphachainEOS=HomosapiensGN=HLA-EPE=1SV=3

MVDGTLLLLLSEALALTQTWAGSHSLKYFHTSVSRPGRGEPRFISVGYVDDTQFVRFDND

AASPRMVPRAPWMEQEGSEYWDRETRSARDTAQIFRVNLRTLRGYYNQSEAGSHTLQWMH

GCELGPDRRFLRGYEQFAYDGKDYLTLNEDLRSWTAVDTAAQISEQKSNDASEAEHQRAY

LEDTCVEWLHKYLEKGKETLLHLEPPKTHVTHHPISDHEATLRCWALGFYPAEITLTWQQ

DGEGHTQDTELVETRPAGDGTFQKWAAVVVPSGEEQRYTCHVQHEGLPEPVTLRWKPASQ

PTIPIVGIIAGLVLLGSVVSGAVVAAVIWRKKSSGGKGGSYSKAEWSDSAQGSESHSL

301.>sp|P30556|AGTR1_HUMANType-1angiotensinIIreceptorOS=HomosapiensGN=AGTR1PE=1SV=1

MILNSSTEDGIKRIQDDCPKAGRHNYIFVMIPTLYSIIFVVGIFGNSLVVIVIYFYMKLK

TVASVFLLNLALADLCFLLTLPLWAVYTAMEYRWPFGNYLCKIASASVSFNLYASVFLLT

CLSIDRYLAIVHPMKSRLRRTMLVAKVTCIIIWLLAGLASLPAIIHRNVFFIENTNITVC

AFHYESQNSTLPIGLGLTKNILGFLFPFLIILTSYTLIWKALKKAYEIQKNKPRNDDIFK

IIMAIVLFFFFSWIPHQIFTFLDVLIQLGIIRDCRIADIVDTAMPITICIAYFNNCLNPL

FYGFLGKKFKRYFLQLLKYIPPKAKSHSNLSTKMSTLSYRPSDNVSSSTKKPAPCFEVE

302.>sp|Q02083|NAAA_HUMANN-acylethanolamine-hydrolyzingacidamidaseOS=HomosapiensGN=NAAAPE=1SV=3

MRTADREARPGLPSLLLLLLAGAGLSAASPPAAPRFNVSLDSVPELRWLPVLRHYDLDLV

RAAMAQVIGDRVPKWVHVLIGKVVLELERFLPQPFTGEIRGMCDFMNLSLADCLLVNLAY

ESSVFCTSIVAQDSRGHIYHGRNLDYPFGNVLRKLTVDVQFLKNGQIAFTGTTFIGYVGL

WTGQSPHKFTVSGDERDKGWWWENAIAALFRRHIPVSWLIRATLSESENFEAAVGKLAKT

PLIADVYYIVGGTSPREGVVITRNRDGPADIWPLDPLNGAWFRVETNYDHWKPAPKEDDR

RTSAIKALNATGQANLSLEALFQILSVVPVYNNFTIYTTVMSAGSPDKYMTRIRNPSRK

303.>sp|Q86VZ1|P2RY8_HUMANP2Ypurinoceptor8OS=HomosapiensGN=P2RY8PE=1SV=1

MQVPNSTGPDNATLQMLRNPAIAVALPVVYSLVAAVSIPGNLFSLWVLCRRMGPRSPSVI

FMINLSVTDLMLASVLPFQIYYHCNRHHWVFGVLLCNVVTVAFYANMYSSILTMTCISVE

RFLGVLYPLSSKRWRRRRYAVAACAGTWLLLLTALSPLARTDLTYPVHALGIITCFDVLK

WTMLPSVAMWAVFLFTIFILLFLIPFVITVACYTATILKLLRTEEAHGREQRRRAVGLAA

VVLLAFVTCFAPNNFVLLAHIVSRLFYGKSYYHVYKLTLCLSCLNNCLDPFVYYFASREF

QLRLREYLGCRRVPRDTLDTRRESLFSARTTSVRSEAGAHPEGMEGATRPGLQRQESVF

(2^nd^NinO.R.)

304.>sp|P07585|PGS2_HUMANDecorinOS=HomosapiensGN=DCNPE=1SV=1

MKATIILLLLAQVSWAGPFQQRGLFDFMLEDEASGIGPEVPDDRDFEPSLGPVCPFRCQC

HLRVVQCSDLGLDKVPKDLPPDTTLLDLQNNKITEIKDGDFKNLKNLHALILVNNKISKV

SPGAFTPLVKLERLYLSKNQLKELPEKMPKTLQELRAHENEITKVRKVTFNGLNQMIVIE

LGTNPLKSSGIENGAFQGMKKLSYIRIADTNITSIPQGLPPSLTELHLDGNKISRVDAAS

LKGLNNLAKLGLSFNSISAVDNGSLANTPHLRELHLDNNKLTRVPGGLAEHKYIQVVYLH

NNNISVVGSSDFCPPGHNTKKASYSGVSLFSNPVQYWEIQPSTFRCVYVRSAIQLGNYK

305.>sp|Q92187|SIA8D_HUMANCMP-N-acetylneuraminate-poly-alpha-2,8-sialyltransferaseOS=HomosapiensGN=ST8SIA4PE=1SV=1

MRSIRKRWTICTISLLLIFYKTKEIARTEEHQETQLIGDGELSLSRSLVNSSDKIIRKAG

SSIFQHNVEGWKINSSLVLEIRKNILRFLDAERDVSVVKSSFKPGDVIHYVLDRRRTLNI

SHDLHSLLPEVSPMKNRRFKTCAVVGNSGILLDSECGKEIDSHNFVIRCNLAPVVEFAAD

VGTKSDFITMNPSVVQRAFGGFRNESDREKFVHRLSMLNDSVLWIPAFMVKGGEKHVEWV

NALILKNKLKVRTAYPSLRLIHAVRGYWLTNKVPIKRPSTGLLMYTLATRFCDEIHLYGF

WPFPKDLNGKAVKYHYYDDLKYRYFSNASPHRMPLEFKTLNVLHNRGALKLTTGKCVKQ

306.>sp|O00115|DNS2A_HUMANDeoxyribonuclease-2-alphaOS=HomosapiensGN=DNASE2PE=1SV=2

MIPLLLAALLCVPAGALTCYGDSGQPVDWFVVYKLPALRGSGEAAQRGLQYKYLDESSGG

WRDGRALINSPEGAVGRSLQPLYRSNTSQLAFLLYNDQPPQPSKAQDSSMRGHTKGVLLL

DHDGGFWLVHSVPNFPPPASSAAYSWPHSACTYGQTLLCVSFPFAQFSKMGKQLTYTYPW

VYNYQLEGIFAQEFPDLENVVKGHHVSQEPWNSSITLTSQAGAVFQSFAKFSKFGDDLYS

GWLAAALGTNLQVQFWHKTVGILPSNCSDIWQVLNVNQIAFPGPAGPSFNSTEDHSKWCV

SPKGPWTCVGDMNRNQGEEQRGGGTLCAQLPALWKAFQPLVKNYQPCNGMARKPSRAYKI

307.>sp|Q96J42|TXD15_HUMANThioredoxindomain-containingprotein15OS=HomosapiensGN=TXNDC15PE=1SV=1

MVPAAGRRPPRVMRLLGWWQVLLWVLGLPVRGVEVAEESGRLWSEEQPAHPLQVGAVYLG

EEELLHDPMGQDRAAEEANAVLGLDTQGDHMVMLSVIPGEAEDKVSSEPSGVTCGAGGAE

DSRCNVRESLFSLDGAGAHFPDREEEYYTEPEVAESDAAPTEDSNNTESLKSPKVNCEER

NITGLENFTLKILNMSQDLMDFLNPNGSDCTLVLFYTPWCRFSASLAPHFNSLPRAFPAL

HFLALDASQHSSLSTRFGTVAVPNILLFQGAKPMARFNHTDRTLETLKIFIFNQTGIEAK

KNVVVTQADQIGPLPSTLIKSVDWLLVFSLFFLISFIMYATIRTESIRWLIPGQEQEHVE

308.>sp|Q9NV96|CC50A_HUMANCellcyclecontrolprotein50AOS=HomosapiensGN=TMEM30APE=1SV=1

MAMNYNAKDEVDGGPPCAPGGTAKTRRPDNTAFKQQRLPAWQPILTAGTVLPIFFIIGLI

FIPIGIGIFVTSNNIREIEIDYTGTEPSSPCNKCLSPDVTPCFCTINFTLEKSFEGNVFM

YYGLSNFYQNHRRYVKSRDDSQLNGDSSALLNPSKECEPYRRNEDKPIAPCGAIANSMFN

DTLELFLIGNDSYPIPIALKKKGIAWWTDKNVKFRNPPGGDNLEERFKGTTKPVNWLKPV

YMLDSDPDNNGFINEDFIVWMRTAALPTFRKLYRLIERKSDLHPTLPAGRYSLNVTYNYP

VHYFDGRKRMILSTISWMGGKNPFLGIAYIAVGSISFLLGVVLLVINHKYRNSSNTADIT

I

309.>sp|Q16769|QPCT_HUMANGlutaminyl-peptidecyclotransferaseOS=HomosapiensGN=QPCTPE=1SV=1

MAGGRHRRVVGTLHLLLLVAALPWASRGVSPSASAWPEEKNYHQPAILNSSALRQIAEGT

SISEMWQNDLQPLLIERYPGSPGSYAARQHIMQRIQRLQADWVLEIDTFLSQTPYGYRSF

SNIISTLNPTAKRHLVLACHYDSKYFSHWNNRVFVGATDSAVPCAMMLELARALDKKLLS

LKTVSDSKPDLSLQLIFFDGEEAFLHWSPQDSLYGSRHLAAKMASTPHPPGARGTSQLHG

MDLLVLLDLIGAPNPTFPNFFPNSARWFERLQAIEHELHELGLLKDHSLEGRYFQNYSYG

GVIQDDHIPFLRRGVPVLHLIPSPFPEVWHTMDDNEENLDESTIDNLNKILQVFVLEYLH

L

310.>sp|P03989|1B27_HUMANHLAclassIhistocompatibilityantigen,B-27alphachainOS=HomosapiensGN=HLA-BPE=1SV=2

MRVTAPRTLLLLLWGAVALTETWAGSHSMRYFHTSVSRPGRGEPRFITVGYVDDTLFVRF

DSDAASPREEPRAPWIEQEGPEYWDRETQICKAKAQTDREDLRTLLRYYNQSEAGSHTLQ

NMYGCDVGPDGRLLRGYHQDAYDGKDYIALNEDLSSWTAADTAAQITQRKWEAARVAEQL

RAYLEGECVEWLRRYLENGKETLQRADPPKTHVTHHPISDHEATLRCWALGFYPAEITLT

WQRDGEDQTQDTELVETRPAGDRTFQKWAAVVVPSGEEQRYTCHVQHEGLPKPLTLRWEP

SSQSTVPIVGIVAGLAVLAVVVIGAVVAAVMCRRKSSGGKGGSYSQAACSDSAQGSDVSL

TA

311.>sp|P01889|1B07_HUMANHLAclassIhistocompatibilityantigen,B-7alphachainOS=HomosapiensGN=HLA-BPE=1SV=3

MLVMAPRTVLLLLSAALALTETWAGSHSMRYFYTSVSRPGRGEPRFISVGYVDDTQFVRF

DSDAASPREEPRAPWIEQEGPEYWDRNTQIYKAQAQTDRESLRNLRGYYNQSEAGSHTLQ

SMYGCDVGPDGRLLRGHDQYAYDGKDYIALNEDLRSWTAADTAAQITQRKWEAAREAEQR

RAYLEGECVEWLRRYLENGKDKLERADPPKTHVTHHPISDHEATLRCWALGFYPAEITLT

WQRDGEDQTQDTELVETRPAGDRTFQKWAAVVVPSGEEQRYTCHVQHEGLPKPLTLRWEP

SSQSTVPIVGIVAGLAVLAVVVIGAVVAAVMCRRKSSGGKGGSYSQAACSDSAQGSDVSL

TA

312.>sp|Q04826|1B40_HUMANHLAclassIhistocompatibilityantigen,B-40alphachainOS=HomosapiensGN=HLA-BPE=1SV=1

MRVTAPRTLLLLLWGAVALTETWAGSHSMRYFHTSVSRPGRGEPRFITVGYVDDTLFVRF

DSDATSPRKEPRAPWIEQEGPEYWDRETQISKTNTQTYRESLRNLRGYYNQSEAGSHTLQ

SMYGCDVGPDGRLLRGHNQYAYDGKDYIALNEDLRSWTAADTAAQITQRKWEAARVAEQL

RAYLEGECVEWLRRYLENGKETLQRADPPKTHVTHHPISDHEATLRCWALGFYPAEITLT

WQRDGEDQTQDTELVETRPAGDRTFQKWAAVVVPSGEEQRYTCHVQHEGLPKPLTLRWEP

SSQSTVPIVGIVAGLAVLAVVVIGAVVAAVMCRRKSSGGKGGSYSQAACSDSAQGSDVSL

TA

313.>sp|P30443|1A01_HUMANHLAclassIhistocompatibilityantigen,A-1alphachainOS=HomosapiensGN=HLA-APE=1SV=1

MAVMAPRTLLLLLSGALALTQTWAGSHSMRYFFTSVSRPGRGEPRFIAVGYVDDTQFVRF

DSDAASQKMEPRAPWIEQEGPEYWDQETRNMKAHSQTDRANLGTLRGYYNQSEDGSHTIQ

IMYGCDVGPDGRFLRGYRQDAYDGKDYIALNEDLRSWTAADMAAQITKRKWEAVHAAEQR

RVYLEGRCVDGLRRYLENGKETLQRTDPPKTHMTHHPISDHEATLRCWALGFYPAEITLT

WQRDGEDQTQDTELVETRPAGDGTFQKWAAVVVPSGEEQRYTCHVQHEGLPKPLTLRWEL

SSQPTIPIVGIIAGLVLLGAVITGAVVAAVMWRRKSSDRKGGSYTQAASSDSAQGSDVSL

TACKV

314.>sp|P01892|1A02_HUMANHLAclassIhistocompatibilityantigen,A-2alphachainOS=HomosapiensGN=HLA-APE=1SV=1

MAVMAPRTLVLLLSGALALTQTWAGSHSMRYFFTSVSRPGRGEPRFIAVGYVDDTQFVRF

DSDAASQRMEPRAPWIEQEGPEYWDGETRKVKAHSQTHRVDLGTLRGYYNQSEAGSHTVQ

RMYGCDVGSDWRFLRGYHQYAYDGKDYIALKEDLRSWTAADMAAQTTKHKWEAAHVAEQL

RAYLEGTCVEWLRRYLENGKETLQRTDAPKTHMTHHAVSDHEATLRCWALSFYPAEITLT

WQRDGEDQTQDTELVETRPAGDGTFQKWAAVVVPSGQEQRYTCHVQHEGLPKPLTLRWEP

SSQPTIPIVGIIAGLVLFGAVITGAVVAAVMWRRKSSDRKGGSYSQAASSDSAQGSDVSL

TACKV

315.>sp|P01891|1A68_HUMANHLAclassIhistocompatibilityantigen,A-68alphachainOS=HomosapiensGN=HLA-APE=1SV=4

MAVMAPRTLVLLLSGALALTQTWAGSHSMRYFYTSVSRPGRGEPRFIAVGYVDDTQFVRF

DSDAASQRMEPRAPWIEQEGPEYWDRNTRNVKAQSQTDRVDLGTLRGYYNQSEAGSHTIQ

MMYGCDVGSDGRFLRGYRQDAYDGKDYIALKEDLRSWTAADMAAQTTKHKWEAAHVAEQW

RAYLEGTCVEWLRRYLENGKETLQRTDAPKTHMTHHAVSDHEATLRCWALSFYPAEITLT

WQRDGEDQTQDTELVETRPAGDGTFQKWVAVVVPSGQEQRYTCHVQHEGLPKPLTLRWEP

SSQPTIPIVGIIAGLVLFGAVITGAVVAAVMWRRKSSDRKGGSYSQAASSDSAQGSDVSL

TACKV

316.>sp|P05534|1A24_HUMANHLAclassIhistocompatibilityantigen,A-24alphachainOS=HomosapiensGN=HLA-APE=1SV=2

MAVMAPRTLVLLLSGALALTQTWAGSHSMRYFSTSVSRPGRGEPRFIAVGYVDDTQFVRF

DSDAASQRMEPRAPWIEQEGPEYWDEETGKVKAHSQTDRENLRIALRYYNQSEAGSHTLQ

MMFGCDVGSDGRFLRGYHQYAYDGKDYIALKEDLRSWTAADMAAQITKRKWEAAHVAEQQ

RAYLEGTCVDGLRRYLENGKETLQRTDPPKTHMTHHPISDHEATLRCWALGFYPAEITLT

WQRDGEDQTQDTELVETRPAGDGTFQKWAAVVVPSGEEQRYTCHVQHEGLPKPLTLRWEP

SSQPTVPIVGIIAGLVLLGAVITGAVVAAVMWRRNSSDRKGGSYSQAASSDSAQGSDVSL

TACKV

317.>sp|P30501|1C02_HUMANHLAclassIhistocompatibilityantigen,Cw-2alphachainOS=HomosapiensGN=HLA-CPE=1SV=1

MRVMEPRTLILLLSGALALTETWACSHSMRYFYTAVSRPSRGEPHFIAVGYVDDTQFVRF

DSDAASPRGEPRGRWVEQEGPEYWDRETQKYNRQAQTDRVNLRKLRGYYNQSEAGSHTLQ

RMYGCDLGPDGRLLRGYDQSAYDGKDYIALNEDLRSWTAADTAAQITQRKWEAAREAEEW

RAYLEGECVEWLRRYLENGKEKLQRAEHPKTHVTHHPVSDHEATLRCWALGFYPTEITLT

WQRDGEDQTQDTELVETRPAGDGTFQKWAAVVVPSGEEQRYTCHVQHEGLPEPLTLRWEP

SSQPTIPIVGIVAGLAVLAVLAVLGAVVAVVMCRRKSSGGKGGSCSQAASSNSAQGSDES

LIASKA

318.>sp|Q07000|1C15_HUMANHLAclassIhistocompatibilityantigen,Cw-15alphachainOS=HomosapiensGN=HLA-CPE=1SV=1

MRVMAPRTLLLLLSGALALTETWACSHSMRYFYTAVSRPGRGEPHFIAVGYVDDTQFVRF

DSDAASPRGEPRAPWVEQEGPEYWDRETQNYKRQAQTDRVNLRKLRGYYNQSEAGSHIIQ

RMYGCDLGPDGRLLRGHDQLAYDGKDYIALNEDLRSWTAADTAAQITQRKWEAAREAEQL

RAYLEGTCVEWLRRYLENGKETLQRAEHPKTHVTHHPVSDHEATLRCWALGFYPAEITLT

WQRDGEDQTQDTELVETRPAGDGTFQKWAAVVVPSGEEQRYTCHVQHEGLPEPLTLRWEP

SSQPTIPIVGIVAGLAVLAVLAVLGAVMAVVMCRRKSSGGKGGSCSQAASSNSAQGSDES

LIACKA

319.>sp|P05111|INHA_HUMANInhibinalphachainOS=HomosapiensGN=INHAPE=1SV=1

MVLHLLLFLLLTPQGGHSCQGLELARELVLAKVRALFLDALGPPAVTREGGDPGVRRLPR

RHALGGFTHRGSEPEEEEDVSQAILFPATDASCEDKSAARGLAQEAEEGLFRYMFRPSQH

TRSRQVTSAQLWFHTGLDRQGTAASNSSEPLLGLLALSPGGPVAVPMSLGHAPPHWAVLH

LATSALSLLTHPVLVLLLRCPLCTCSARPEATPFLVAHTRTRPPSGGERARRSTPLMSWP

WSPSALRLLQRPPEEPAAHANCHRVALNISFQELGWERWIVYPPSFIFHYCHGGCGLHIP

PNLSLPVPGAPPTPAQPYSLLPGAQPCCAALPGTMRPLHVRTTSDGGYSFKYETVPNLLT

QHCACI(3^rd^Npartial)

320.>sp|P02765|FETUA_HUMANAlpha-2-HS-glycoproteinOS=HomosapiensGN=AHSGPE=1SV=1

MKSLVLLLCLAQLWGCHSAPHGPGLIYRQPNCDDPETEEAALVAIDYINQNLPWGYKHTL

NQIDEVKVWPQQPSGELFEIEIDTLETTCHVLDPTPVARCSVRQLKEHAVEGDCDFQLLK

LDGKFSVVYAKCDSSPDSAEDVRKVCQDCPLLAPLNDTRVVHAAKAALAAFNAQNNGSNF

QLEEISRAQLVPLPPSTYVEFTVSGTDCVAKEATEAAKCNLLAEKQYGFCKATLSEKLGG

AEVAVTCTVFQTQPVTSQPQPEGANEAVPTPVVDPDAPPSPPLGAPGLPPAGSPPDSHVL

LAAPPGHQLHRAHYDLRHTFMGVVSLGSPSGEVSHPRKTRTVVQPSVGAAAGPVVPPCPG

RIRHFKV

321.>sp|P21810|PGS1_HUMANBiglycanOS=HomosapiensGN=BGNPE=1SV=2

MWPLWRLVSLLALSQALPFEQRGFWDFTLDDGPFMMNDEEASGADTSGVLDPDSVTPTYS

AMCPFGCHCHLRVVQCSDLGLKSVPKEISPDTTLLDLQNNDISELRKDDFKGLQHLYALV

LVNNKISKIHEKAFSPLRKLQKLYISKNHLVEIPPNLPSSLVELRIHDNRIRKVPKGVFS

GLRNMNCIEMGGNPLENSGFEPGAFDGLKLNYLRISEAKLTGIPKDLPETLNELHLDHNK

IQAIELEDLLRYSKLYRLGLGHNQIRMIENGSLSFLPTLRELHLDNNKLARVPSGLPDLK

LLQVVYLHSNNITKVGVNDFCPMGFGVKRAYYNGISLFNNPVPYWEVQPATFRCVTDRLA

IQFGNYKK

322.>sp|Q6ZUK4|TMM26_HUMANTransmembraneprotein26OS=HomosapiensGN=TMEM26PE=1SV=1

MEGLVFLNALATRLLFLLHSLVGVWRVTEVKKEPRYWLLALLNLLLFLETALTLKFKRGR

GYKWFSPAIFLYLISIVPSLWLLELHHETQYCSIQAEGTSQNTSRKEDFNQTLTSNEQTS

RADDLIETAKVFVNNLSTVCEKVWTLGLHQTFLLMLIIGRWLLPIGGGITRDQLSQLLLM

FVGTAADILEFTSETLEEQNVRNSPALVYAILVIWTWSMLQFPLDLAVQNVVCPVSVTER

GFPSLFFCQYSADLWNIGISVFIQDGPFLVVRLILMTYFKVINQMLVFFAAKNFLVVVLQ

LYRLVVLALAVRASLRSQSEGLKGEHGCRAQTSESGPSQRDWQNESKEGLAIPLRGSPVT

SDDSHHTP

323.>sp|P31785|IL2RG_HUMANCytokinereceptorcommonsubunitgammaOS=HomosapiensGN=IL2RGPE=1SV=1

MLKPSLPFTSLLFLQLPLLGVGLNTTILTPNGNEDTTADFFLTTMPTDSLSVSTLPLPEV

QCFVFNVEYMNCTWNSSSEPQPTNLTLHYWYKNSDNDKVQKCSHYLFSEEITSGCQLQKK

EIHLYQTFVVQLQDPREPRRQATQMLKLQNLVIPWAPENLTLHKLSESQLELNWNNRFLN

HCLEHLVQYRTDWDHSWTEQSVDYRHKFSLPSVDGQKRYTFRVRSRFNPLCGSAQHWSEW

SHPIHWGSNTSKENPFLFALEAVVISVGSMGLIISLLCVYFWLERTMPRIPTLKNLEDLV

TEYHGNFSAWSGVSKGLAESLQPDYSERLCLVSEIPPKGGALGEGPGASPCNQHSPYWAP

PCYTLKPET

324.>sp|Q9BZW8|CD244_HUMANNaturalkillercellreceptor2B4OS=HomosapiensGN=CD244PE=1SV=2

MLGQVVTLILLLLLKVYQGKGCQGSADHVVSISGVPLQLQPNSIQTKVDSIAWKKLLPSQ

NGFHHILKWENGSLPSNTSNDRFSFIVKNLSLLIKAAQQQDSGLYCLEVTSISGKVQTAT

FQVFVFESLLPDKVEKPRLQGQGKILDRGRCQVALSCLVSRDGNVSYAWYRGSKLIQTAG

NLTYLDEEVDINGTHTYTCNVSNPVSWESHTLNLTQDCQNAHQEFRFWPFLVIIVILSAL

FLGTLACFCVWRRKRKEKQSETSPKEFLTIYEDVKDLKTRRNHEQEQTFPGGGSTIYSMI

QSQSSAPTSQEPAYTLYSLIQPSRKSGSRKRNHSPSFNSTIYEVIGKSQPKAQNPARLSR

KELENFDVYS

325.>sp|Q99677|LPAR4_HUMANLysophosphatidicacidreceptor4OS=HomosapiensGN=LPAR4PE=1SV=1

MGDRRFIDFQFQDSNSSLRPRLGNATANNTCIVDDSFKYNLNGAVYSVVFILGLITNSVS

LFVFCFRMKMRSETAIFITNLAVSDLLFVCTLPFKIFYNFNRHWPFGDTLCKISGTAFLT

NIYGSMLFLTCISVDRFLAIVYPFRSRTIRTRRNSAIVCAGVWILVLSGGISASLFSTTN

VNNATTTCFEGFSKRVWKTYLSKITIFIEVVGFIIPLILNVSCSSVVLRTLRKPATLSQI

GTNKKKVLKMITVHMAVFVVCFVPYNSVLFLYALVRSQAITNCFLERFAKIMYPITLCLA

TLNCCFDPFIYYFTLESFQKSFYINAHIRMESLFKTETPLTTKPSLPAIQEEVSDQTTNN

GGELMLESTF

326.>sp|Q68CQ7|GL8D1_HUMANGlycosyltransferase8domain-containingprotein1OS=HomosapiensGN=GLT8D1PE=1SV=2

MSFRKVNIIILVLAVALFLLVLHHNFLSLSSLLRNEVTDSGIVGPQPIDFVPNALRHAVD

GRQEEIPVVIAASEDRLGGAIAAINSIQHNTRSNVIFYIVTLNNTADHLRSWLNSDSLKS

IRYKIVNFDPKLLEGKVKEDPDQGESMKPLTFARFYLPILVPSAKKAIYMDDDVIVQGDI

LALYNTALKPGHAAAFSEDCDSASTKVVIRGAGNQYNYIGYLDYKKERIRKLSMKASTCS

FNPGVFVANLTEWKRQNITNQLEKWMKLNVEEGLYSRTLAGSITTPPLLIVFYQQHSTID

PMWNVRHLGSSAGKRYSPQFVKAAKLLHWNGHLKPWGRTASYTDVWEKWYIPDPTGKFNL

IRRYTEISNIK

327.>sp|Q95604|1C17_HUMANHLAclassIhistocompatibilityantigen,Cw-17alphachainOS=HomosapiensGN=HLA-CPE=1SV=1

MRVMAPQALLLLLSGALALIETWAGSHSMRYFYTAVSRPGRGEPRFIAVGYVDDTQFVRF

DSDAASPRGEPRAPWVEQEGPEYWDRETQKYKRQAQADRVNLRKLRGYYNQSEAGSHTIQ

RMYGCDLGPDGRLLRGYNQFAYDGKDYIALNEDLRSWTAADTAAQISQRKLEAAREAEQL

RAYLEGECVEWLRGYLENGKETLQRAERPKTHVTHHPVSDHEATLRCWALGFYPAEITLT

WQRDGEDQTQDTELVETRPAGDGTFQKWAAVVVPSGQEQRYTCHVQHEGLQEPCTLRWKP

SSQPTIPNLGIVSGPAVLAVLAVLAVLAVLGAVVAAVIHRRKSSGGKGGSCSQAASSNSA

QGSDESLIACKA

328.>sp|P14151|LYAM1_HUMANL-selectinOS=HomosapiensGN=SELLPE=1SV=2

MIFPWKCQSTQRDLWNIFKLWGWTMLCCDFLAHHGTDCWTYHYSEKPMNWQRARRFCRDN

YTDLVAIQNKAEIEYLEKTLPFSRSYYWIGIRKIGGIWTWVGTNKSLTEEAENWGDGEPN

NKKNKEDCVEIYIKRNKDAGKWNDDACHKLKAALCYTASCQPWSCSGHGECVEIINNYTC

NCDVGYYGPQCQFVIQCEPLEAPELGTMDCTHPLGNFSFSSQCAFSCSEGTNLTGIEETT

CGPFGNWSSPEPTCQVIQCEPLSAPDLGIMNCSHPLASFSFTSACTFICSEGTELIGKKK

TICESSGIWSNPSPICQKLDKSFSMIKEGDYNPLFIPVAVMVTAFSGLAFIIWLARRLKK

GKKSKRSMNDPY

329.>sp|P12314|FCGR1_HUMANHighaffinityimmunoglobulingammaFcreceptorIOS=HomosapiensGN=FCGR1APE=1SV=2

MWFLTTLLLWVPVDGQVDTTKAVITLQPPWVSVFQEETVTLHCEVLHLPGSSSTQWFLNG

TATQTSTPSYRITSASVNDSGEYRCQRGLSGRSDPIQLEIHRGWLLLQVSSRVFTEGEPL

ALRCHAWKDKLVYNVLYYRNGKAFKFFHWNSNLTILKTNISHNGTYHCSGMGKHRYTSAG

ISVTVKELFPAPVLNASVTSPLLEGNLVTLSCETKLLLQRPGLQLYFSFYMGSKTLRGRN

TSSEYQILTARREDSGLYWCEAATEDGNVLKRSPELELQVLGLQLPTPVWFHVLFYLAVG

IMFLVNTVLWVTIRKELKRKKKWDLEISLDSGHEKKVISSLQEDRHLEEELKCQEQKEEQ

LQEGVHRKEPQGAT

330.>sp|Q8NBK3|SUMF1_HUMANSulfatase-modifyingfactor1OS=HomosapiensGN=SUMF1PE=1SV=3

MAAPALGLVCGRCPELGLVLLLLLLSLLCGAAGSQEAGTGAGAGSLAGSCGCGTPQRPGA

HGSSAAAHRYSREANAPGPVPGERQLAHSKMVPIPAGVFTMGTDDPQIKQDGEAPARRVT

IDAFYMDAYEVSNTEFEKFVNSTGYLTEAEKFGDSFVFEGMLSEQVKTNIQQAVAAAPWW

LPVKGANWRHPEGPDSTILHRPDHPVLHVSWNDAVAYCTWAGKRLPTEAEWEYSCRGGLH

NRLFPWGNKLQPKGQHYANIWQGEFPVTNTGEDGFQGTAPVDAFPPNGYGLYNIVGNAWE

WTSDWWTVHHSVEETLNPKGPPSGKDRVKKGGSYMCHRSYCYRYRCAARSQNTPDSSASN

LGFRCAADRLPTMD

331.>sp|Q9UIK5|TEFF2_HUMANTomoregulin-2OS=HomosapiensGN=TMEFF2PE=1SV=1

MVLWESPRQCSSWTLCEGFCWLLLLPVMLLIVARPVKLAAFPTSLSDCQTPTGWNCSGYD

DRENDLFLCDTNTCKFDGECLRIGDTVTCVCQFKCNNDYVPVCGSNGESYQNECYLRQAA

CKQQSEILVVSEGSCATDAGSGSGDGVHEGSGETSQKETSTCDICQFGAECDEDAEDVWC

VCNIDCSQTNFNPLCASDGKSYDNACQIKEASCQKQEKIEVMSLGRCQDNTTTTTKSEDG

HYARTDYAENANKLEESAREHHIPCPEHYNGFCMHGKCEHSINMQEPSCRCDAGYTGQHC

EKKDYSVLYVVPGPVRFQYVLIAAVIGTIQIAVICVVVLCITRKCPRSNRIHRQKQNTGH

YSSDNTTRASTRLI

332.>sp|P08571|CD14_HUMANMonocytedifferentiationantigenCD14OS=HomosapiensGN=CD14PE=1SV=2

MERASCLLLLLLPLVHVSATTPEPCELDDEDFRCVCNFSEPQPDWSEAFQCVSAVEVEIH

AGGLNLEPFLKRVDADADPRQYADTVKALRVRRLTVGAAQVPAQLLVGALRVLAYSRLKE

LTLEDLKITGTMPPLPLEATGLALSSLRLRNVSWATGRSWLAELQQWLKPGLKVLSIAQA

HSPAFSCEQVRAFPALTSLDLSDNPGLGERGLMAALCPHKFPAIQNLALRNTGMETPTGV

CAALAAAGVQPHSLDLSHNSLRATVNPSAPRCMWSSALNSLNLSFAGLEQVPKGLPAKLR

VLDLSCNRLNRAPQPDELPEVDNLTLDGNPFLVPGTALPHEGSMNSGVVPACARSTLSVG

VSGTLVLLQGARGFA

333.>sp|Q8TCT9|HM13_HUMANMinorhistocompatibilityantigenH13OS=HomosapiensGN=HM13PE=1SV=1

MDSALSDPHNGSAEAGGPTNSTTRPPSTPEGIALAYGSLLLMALLPIFFGALRSVRCARG

KNASDMPETITSRDAARFPIIASCTLLGLYLFFKIFSQEYINLLLSMYFFVLGILALSHT

ISPFMNKFFPASFPNRQYQLLFTQGSGENKEEIINYEFDTKDLVCLGLSSIVGVWYLLRK

HWIANNLFGLAFSLNGVELLHLNNVSTGCILLGGLFIYDVFWVFGTNVMVTVAKSFEAPI

KLVFPQDLLEKGLEANNFAMLGLGDVVIPGIFIALLLRFDISLKKNTHTYFYTSFAAYIF

GLGLTIFIMHIFKHAQPALLYLVPACIGFPVLVALAKGEVTEMFSYEESNPKDPAAVTES

KEGTEASASKGLEKKEK

334.>sp|P01860|IGHG3_HUMANIggamma-3chainCregionOS=HomosapiensGN=IGHG3PE=1SV=2

ASTKGPSVFPLAPCSRSTSGGTAALGCLVKDYFPEPVTVSWNSGALTSGVHTFPAVLQSS

GLYSLSSVVTVPSSSLGTQTYTCNVNHKPSNTKVDKRVELKTPLGDTTHTCPRCPEPKSC

DTPPPCPRCPEPKSCDTPPPCPRCPEPKSCDTPPPCPRCPAPELLGGPSVFLFPPKPKDT

LMISRTPEVTCVVVDVSHEDPEVQFKWYVDGVEVHNAKTKPREEQYNSTFRVVSVLTVLH

QDWLNGKEYKCKVSNKALPAPIEKTISKTKGQPREPQVYTLPPSREEMTKNQVSLTCLVK

GFYPSDIAVEWESSGQPENNYNTTPPMLDSDGSFFLYSKLTVDKSRWQQGNIFSCSVMHE

ALHNRFTQKSLSLSPGK

335.>sp|Q9BXN1|ASPN_HUMANAsporinOS=HomosapiensGN=ASPNPE=1SV=2

MKEYVLLLFLALCSAKPFFSPSHIALKNMMLKDMEDTDDDDDDDDDDDDDDEDNSLFPTR

EPRSHFFPFDLFPMCPFGCQCYSRVVHCSDLGLTSVPTNIPFDTRMLDLQNNKIKEIKEN

DFKGLTSLYGLILNNNKLTKIHPKAFLTTKKLRRLYLSHNQLSEIPLNLPKSLAELRIHE

NKVKKIQKDTFKGMNALHVLEMSANPLDNNGIEPGAFEGVTVFHIRIAEAKLTSVPKGLP

PTLLELHLDYNKISTVELEDFKRYKELQRLGLGNNKITDIENGSLANIPRVREIHLENNK

LKKIPSGLPELKYLQIIFLHSNSIARVGVNDFCPTVPKMKKSLYSAISLFNNPVKYWEMQ

PATFRCVLSRMSVQLGNFGM

336.>sp|Q96G23|CERS2_HUMANCeramidesynthase2OS=HomosapiensGN=CERS2PE=1SV=1

MLQTLYDYFWWERLWLPVNLTWADLEDRDGRVYAKASDLYITLPLALLFLIVRYFFELYV

ATPLAALLNIKEKTRLRAPPNATLEHFYLTSGKQPKQVEVELLSRQSGLSGRQVERWFRR

RRNQDRPSLLKKFREASWRFTFYLIAFIAGMAVIVDKPWFYDMKKVWEGYPIQSTIPSQY

WYYMIELSFYWSLLFSIASDVKRKDFKEQIIHHVATIILISFSWFANYIRAGTLIMALHD

SSDYLLESAKMFNYAGWKNTCNNIFIVFAIVFIITRLVILPFWILHCTLVYPLELYPAFF

GYYFFNSMMGVLQLLHIFWAYLILRMAHKFITGKLVEDERSDREETESSEGEEAAAGGGA

KSRPLANGHPILNNNHRKND

337.>sp|Q14627|I13R2_HUMANInterleukin-13receptorsubunitalpha-2OS=HomosapiensGN=IL13RA2PE=1SV=1

MAFVCLAIGCLYTFLISTTFGCTSSSDTEIKVNPPQDFEIVDPGYLGYLYLQWQPPLSLD

HFKECTVEYELKYRNIGSETWKTIITKNLHYKDGFDLNKGIEAKIHTLLPWQCTNGSEVQ

SSWAETTYWISPQGIPETKVQDMDCVYYNWQYLLCSWKPGIGVLLDTNYNLFYWYEGLDH

ALQCVDYIKADGQNIGCRFPYLEASDYKDFYICVNGSSENKPIRSSYFTFQLQNIVKPLP

PVYLTFTRESSCEIKLKWSIPLGPIPARCFDYEIEIREDDTTLVTATVENETYTLKTTNE

TRQLCFVVRSKVNIYCSDDGIWSEWSDKQCWEGEDLSKKTLLRFWLPFGFILILVIFVTG

LLLRKPNTYPKMIPEFFCDT

338.>sp|P41145|OPRK_HUMANKappa-typeopioidreceptorOS=HomosapiensGN=OPRK1PE=1SV=2

MDSPIQIFRGEPGPTCAPSACLPPNSSAWFPGWAEPDSNGSAGSEDAQLEPAHISPAIPV

IITAVYSVVFVVGLVGNSLVMFVIIRYTKMKTATNIYIFNLALADALVTTTMPFQSTVYL

MNSWPFGDVLCKIVISIDYYNMFTSIFTLTMMSVDRYIAVCHPVKALDFRTPLKAKIINI

CIWLLSSSVGISAIVLGGTKVREDVDVIECSLQFPDDDYSWWDLFMKICVFIFAFVIPVL

IIIVCYTLMILRLKSVRLLSGSREKDRNLRRITRLVLVVVAVFVVCWTPIHIFILVEALG

STSHSTAALSSYYFCIALGYTNSSLNPILYAFLDENFKRCFRDFCFPLKMRMERQSTSRV

RNTVQDPAYLRDIDGMNKPV

339.>sp|O43173|SIA8C_HUMANSia-alpha-2,3-Gal-beta-1,4-GlcNAc-R:alpha2,8-sialyltransferaseOS=HomosapiensGN=ST8SIA3PE=1SV=3

MRNCKMARVASVLGLVMLSVALLILSLISYVSLKKENIFTTPKYASPGAPRMYMFHAGFR

SQFALKFLDPSFVPITNSLTQELQEKPSKWKFNRTAFLHQRQEILQHVDVIKNFSLTKNS

VRIGQLMHYDYSSHKYVFSISNNFRSLLPDVSPIMNKHYNICAVVGNSGILTGSQCGQEI

DKSDFVFRCNFAPTEAFQRDVGRKTNLTTFNPSILEKYYNNLLTIQDRNNFFLSLKKLDG

AILWIPAFFFHTSATVTRTLVDFFVEHRGQLKVQLAWPGNIMQHVNRYWKNKHLSPKRLS

TGILMYTLASAICEEIHLYGFWPFGFDPNTREDLPYHYYDKKGTKFTTKWQESHQLPAEF

QLLYRMHGEGLTKLTLSHCA

340.>sp|P08174|DAF_HUMANComplementdecay-acceleratingfactorOS=HomosapiensGN=CD55PE=1SV=4

MTVARPSVPAALPLLGELPRLLLLVLLCLPAVWGDCGLPPDVPNAQPALEGRTSFPEDTV

ITYKCEESFVKIPGEKDSVICLKGSQWSDIEEFCNRSCEVPTRLNSASLKQPYITQNYFP

VGTVVEYECRPGYRREPSLSPKLTCLQNLKWSTAVEFCKKKSCPNPGEIRNGQIDVPGGI

LFGATISFSCNTGYKLFGSTSSFCLISGSSVQWSDPLPECREIYCPAPPQIDNGIIQGER

DHYGYRQSVTYACNKGFTMIGEHSIYCTVNNDEGEWSGPPPECRGKSLTSKVPPTVQKPT

TVNVPTTEVSPTSQKTTTKTTTPNAQATRSTPVSRTTKHFHETTPNKGSGTTSGTTRLLS

GHTCFTLTGLLGTLVTMGLLT

341.>sp|O43567|RNF13_HUMANE3ubiquitin-proteinligaseRNF13OS=HomosapiensGN=RNF13PE=1SV=1

MLLSIGMLMLSATQVYTILTVQLFAFLNLLPVEADILAYNFENASQTFDDLPARFGYRLP

AEGLKGFLINSKPENACEPIVPPPVKDNSSGTFIVLIRRLDCNFDIKVLNAQRAGYKAAI

VHNVDSDDLISMGSNDIEVLKKIDIPSVFIGESSANSLKDEFTYEKGGHLILVPEFSLPL

EYYLIPFLIIVGICLILIVIFMITKFVQDRHRARRNRLRKDQLKKLPVHKFKKGDEYDVC

AICLDEYEDGDKLRILPCSHAYHCKCVDPWLTKTKKTCPVCKQKVVPSQGDSDSDTDSSQ

EENEVTEHTPLLRPLASVSAQSFGALSESRSHQNMTESSDYEEDDNEDTDSSDAENEINE

HDVVVQLQPNGERDYNIANTV

342.>sp|P49908|SEPP1_HUMANSelenoproteinPOS=HomosapiensGN=SEPP1PE=1SV=3

MWRSLGLALALCLLPSGGTESQDQSSLCKQPPAWSIRDQDPMLNSNGSVTVVALLQASUY

LCILQASKLEDLRVKLKKEGYSNISYIVVNHQGISSRLKYTHLKNKVSEHIPVYQQEENQ

TDVWTLLNGSKDDFLIYDRCGRLVYHLGLPFSFLTFPYVEEAIKIAYCEKKCGNCSLTTL

KDEDFCKRVSLATVDKTVETPSPHYHHEHHHNHGHQHLGSSELSENQQPGAPNAPTHPAP

PGLHHHHKHKGQHRQGHPENRDMPASEDLQDLQKKLCRKRCINQLLCKLPTDSELAPRSU

CCHCRHLIFEKTGSAITUQCKENLPSLCSUQGLRAEENITESCQURLPPAAUQISQQLIP

TEASASURUKNQAKKUEUPSN

343.>sp|Q9UGM5|FETUB_HUMANFetuin-BOS=HomosapiensGN=FETUBPE=1SV=2

MGLLLPLALCILVLCCGAMSPPQLALNPSALLSRGCNDSDVLAVAGFALRDINKDRKDGY

VLRLNRVNDAQEYRRGGLGSLFYLTLDVLETDCHVLRKKAWQDCGMRIFFESVYGQCKAI

FYMNNPSRVLYLAAYNCTLRPVSKKKIYMTCPDCPSSIPTDSSNHQVLEAATESLAKYNN

ENTSKQYSLFKVTRASSQWVVGPSYFVEYLIKESPCTKSQASSCSLQSSDSVPVGLCKGS

LTRTHWEKFVSVTCDFFESQAPATGSENSAVNQKPTNLPKVEESQQKNTPPTDSPSKAGP

RGSVQYLPDLDDKNSQEKGPQEAFPVHLDLTTNPQGETLDISFLFLEPMEEKLVVLPFPK

EKARTAECPGPAQNASPLVLPP

344.>sp|Q13477|MADCA_HUMANMucosaladdressincelladhesionmolecule1OS=HomosapiensGN=MADCAM1PE=1SV=2

MDFGLALLLAGLLGLLLGQSLQVKPLQVEPPEPVVAVALGASRQLTCRLACADRGASVQW

RGLDTSLGAVQSDTGRSVLTVRNASLSAAGTRVCVGSCGGRTFQHTVQLLVYAFPDQLTV

SPAALVPGDPEVACTAHKVTPVDPNALSFSLLVGGQELEGAQALGPEVQEEEEEPQGDED

VLFRVTERWRLPPLGTPVPPALYCQATMRLPGLELSHRQAIPVLHSPTSPEPPDTTSPES

PDTTSPESPDTTSQEPPDTTSPEPPDKTSPEPAPQQGSTHTPRSPGSTRTRRPEISQAGP

TQGEVIPTGSSKPAGDQLPAALWTSSAVLGLLLLALPTYHLWKRCRHLAEDDTHPPASLR

LLPQVSAWAGLRGTGQVGISPS

345.>sp|P51888|PRELP_HUMANProlarginOS=HomosapiensGN=PRELPPE=1SV=1

MRSPLCWLLPLLILASVAQGQPTRRPRPGTGPGRRPRPRPRPTPSFPQPDEPAEPTDLPP

PLPPGPPSIFPDCPRECYCPPDFPSALYCDSRNLRKVPVIPPRIHYLYLQNNFITELPVE

SFQNATGLRWINLDNNRIRKIDQRVLEKLPGLVFLYMEKNQLEEVPSALPRNLEQLRLSQ

NHISRIPPGVFSKLENLLLLDLQHNRLSDGVFKPDTFHGLKNLMQLNLAHNILRKMPPRV

PTAIHQLYLDSNKIETIPNGYFKSFPNLAFIRLNYNKLTDRGLPKNSFNISNLLVLHLSH

NRISSVPAINNRLEHLYLNNNSIEKINGTQICPNDLVAFHDFSSDLENVPHLRYLRLDGN

YLKPPIPLDLMMCFRLLQSVVI

346.>sp|P21453|S1PR1_HUMANSphingosine1-phosphatereceptor1OS=HomosapiensGN=S1PR1PE=1SV=2

MGPTSVPLVKAHRSSVSDYVNYDIIVRHYNYTGKLNISADKENSIKLTSVVFILICCFII

LENIFVLLTIWKTKKFHRPMYYFIGNLALSDLLAGVAYTANLLLSGATTYKLTPAQWFLR

EGSMFVALSASVFSLLAIAIERYITMLKMKLHNGSNNFRLFLLISACWVISLILGGLPIM

GWNCISALSSCSTVLPLYHKHYILFCTTVFTLLLLSIVILYCRIYSLVRTRSRRLTFRKN

ISKASRSSEKSLALLKTVIIVLSVFIACWAPLFILLLLDVGCKVKTCDILFRAEYFLVLA

VLNSGTNPIIYTLTNKEMRRAFIRIMSCCKCPSGDSAGKFKRPIIAGMEFSRSKSDNSSH

PQKDEGDNPETIMSSGNVNSSS

347.>sp|P36222|CH3L1_HUMANChitinase-3-likeprotein1OS=HomosapiensGN=CHI3L1PE=1SV=2

MGVKASQTGFVVLVLLQCCSAYKLVCYYTSWSQYREGDGSCFPDALDRFLCTHIIYSFAN

ISNDHIDTWEWNDVTLYGMLNTLKNRNPNLKTLLSVGGWNFGSQRFSKIASNTQSRRTFI

KSVPPFLRTHGFDGLDLAWLYPGRRDKQHFTTLIKEMKAEFIKEAQPGKKQLLLSAALSA

GKVTIDSSYDIAKISQHLDFISIMTYDFHGAWRGTTGHHSPLFRGQEDASPDRFSNTDYA

VGYMLRLGAPASKLVMGIPTFGRSFTLASSETGVGAPISGPGIPGRFTKEAGTLAYYEIC

DFLRGATVHRILGQQVPYATKGNQWVGYDDQESVKSKVQYLKDRQLAGAMVWALDLDDFQ

GSFCGQDLRFPLTNAIKDALAAT

348.>sp|P80370|DLK1_HUMANProteindeltahomolog1OS=HomosapiensGN=DLK1PE=1SV=3

MTATEALLRVLLLLLAFGHSTYGAECFPACNPQNGFCEDDNVCRCQPGWQGPLCDQCVTS

PGCLHGLCGEPGQCICTDGWDGELCDRDVRACSSAPCANNRTCVSLDDGLYECSCAPGYS

GKDCQKKDGPCVINGSPCQHGGTCVDDEGRASHASCLCPPGFSGNFCEIVANSCTPNPCE

NDGVCTDIGGDFRCRCPAGFIDKTCSRPVTNCASSPCQNGGTCLQHTQVSYECLCKPEFT

GLTCVKKRALSPQQVTRLPSGYGLAYRLTPGVHELPVQQPEHRILKVSMKELNKKTPLLT

EGQAICFTILGVLTSLVVLGTVGIVFLNKCETWVSNLRYNHMLRKKKNLLLQYNSGEDLA

VNIIFPEKIDMTTFSKEAGDEEI

349.>sp|Q9Y282|ERGI3_HUMANEndoplasmicreticulum-Golgiintermediatecompartmentprotein3OS=HomosapiensGN=ERGIC3PE=1SV=1

MEALGKLKQFDAYPKTLEDFRVKTCGGATVTIVSGLLMLLLFLSELQYYLTTEVHPELYV

DKSRGDKLKINIDVLFPHMPCAYLSIDAMDVAGEQQLDVEHNLFKQRLDKDGIPVSSEAE

RHELGKVEVTVFDPDSLDPDRCESCYGAEAEDIKCCNTCEDVREAYRRRGWAFKNPDTIE

QCRREGFSQKMQEQKNEGCQVYGFLEVNKVAGNFHFAPGKSFQQSHVHVHDLQSFGLDNI

NMTHYIQHLSFGEDYPGIVNPLDHTNVTAPQASMMFQYFVKVVPTVYMKVDGEVLRTNQF

SVTRHEKVANGLLGDQGLPGVFVLYELSPMMVKLTEKHRSFTHFLTGVCAIIGGMFTVAG

LIDSLIYHSARAIQKKIDLGKTT

350.>sp|Q6ZMG9|CERS6_HUMANCeramidesynthase6OS=HomosapiensGN=CERS6PE=1SV=1

MAGILAWFWNERFWLPHNVTWADLKNTEEATFPQAEDLYLAFPLAFCIFMVRLIFERFVA

KPCAIALNIQANGPQIAPPNAILEKVFTAITKHPDEKRLEGLSKQLDWDVRSIQRWFRQR

RNQEKPSTLTRFCESMWRFSFYLYVFTYGVRFLKKTPWLWNTRHCWYNYPYQPLTTDLHY

YYILELSFYWSLMFSQFTDIKRKDFGIMFLHHLVSIFLITFSYVNNMARVGTLVLCLHDS

ADALLEAAKMANYAKFQKMCDLLFVMFAVVFITTRLGIFPLWVLNTTLFESWEIVGPYPS

WWVFNLLLLLVQGLNCFWSYLIVKIACKAVSRGKVSKDDRSDIESSSDEEDSEPPGKNPH

TATTTNGTSGTNGYLLTGSCSMDD

351.>sp|P01880|IGHD_HUMANIgdeltachainCregionOS=HomosapiensGN=IGHDPE=1SV=2

APTKAPDVFPIISGCRHPKDNSPVVLACLITGYHPTSVTVTWYMGTQSQPQRTFPEIQRR

DSYYMTSSQLSTPLQQWRQGEYKCVVQHTASKSKKEIFRWPESPKAQASSVPTAQPQAEG

SLAKATTAPATTRNTGRGGEEKKKEKEKEEQEERETKTPECPSHTQPLGVYLLTPAVQDL

WLRDKATFTCFVVGSDLKDAHLTWEVAGKVPTGGVEEGLLERHSNGSQSQHSRLTLPRSL

WNAGTSVTCTLNHPSLPPQRLMALREPAAQAPVKLSLNLLASSDPPEAASWLLCEVSGFS

PPNILLMWLEDQREVNTSGFAPARPPPQPGSTTFWAWSVLRVPAPPSPQPATYTCVVSHE

DSRTLLNASRSLEVSYVTDHGPMK

352.>sp|Q7Z418|KCNKI_HUMANPotassiumchannelsubfamilyKmember18OS=HomosapiensGN=KCNK18PE=1SV=1

MEVSGHPQARRCCPEALGKLFPGLCFLCFLVTYALVGAVVFSAIEDGQVLVAADDGEFEK

FLEELCRILNCSETVVEDRKQDLQGHLQKVKPQWFNRTTHWSFLSSLFFCCTVFSTVGYG

YIYPVTRLGKYLCMLYALFGIPLMFLVLTDTGDILATILSTSYNRFRKFPFFTRPLLSKW

CPKSLFKKKPDPKPADEAVPQIIISAEELPGPKLGTCPSRPSCSMELFERSHALEKQNTL

QLPPQAMERSNSCPELVLGRLSYSIISNLDEVGQQVERLDIPLPIIALIVFAYISCAAAI

LPFWETQLDFENAFYFCFVTLTTIGFGDTVLEHPNFFLFFSIYIIVGMEIVFIAFKLVQN

RLIDIYKNVMLFFAKGKFYHLVKK

353.>sp|P35613|BASI_HUMANBasiginOS=HomosapiensGN=BSGPE=1SV=2

MAAALFVLLGFALLGTHGASGAAGFVQAPLSQQRWVGGSVELHCEAVGSPVPEIQWWFEG

QGPNDTCSQLWDGARLDRVHIHATYHQHAASTISIDTLVEEDTGTYECRASNDPDRNHLT

RAPRVKWVRAQAVVLVLEPGTVFTTVEDLGSKILLTCSLNDSATEVTGHRWLKGGVVLKE

DALPGQKTEFKVDSDDQWGEYSCVFLPEPMGTANIQLHGPPRVKAVKSSEHINEGETAML

VCKSESVPPVTDWAWYKITDSEDKALMNGSESRFFVSSSQGRSELHIENLNMEADPGQYR

CNGTSSKGSDQAIITLRVRSHLAALWPFLGIVAEVLVLVTIIFIYEKRRKPEDVLDDDDA

GSAPLKSSGQHQNDKGKNVRQRNSS

354.>sp|Q01459|DIAC_HUMANDi-N-acetylchitobiaseOS=HomosapiensGN=CTBSPE=1SV=1

MSRPQLRRWRLVSSPPSGVPGLALLALLALLALRLAAGTDCPCPEPELCRPIRHHPDFEV

FVFDVGQKTWKSYDWSQITTVATFGKYDSELMCYAHSKGARVVLKGDVSLKDIIDPAFRA

SWIAQKLNLAKTQYMDGINIDIEQEVNCLSPEYDALTALVKETTDSFHREIEGSQVTFDV

AWSPKNIDRRCYNYTGIADACDFLFVMSYDEQSQIWSECIAAANAPYNQTLTGYNDYIKM

SINPKKLVMGVPWYGYDYTCLNLSEDHVCTIAKVPFRGAPCSDAAGRQVPYKTIMKQINS

SISGNLWDKDQRAPYYNYKDPAGHFHQVWYDNPQSISLKATYIQNYRLRGIGMWNANCLD

YSGDAVAKQQTEEMWEVLKPKLLQR

355.>sp|P15309|PPAP_HUMANProstaticacidphosphataseOS=HomosapiensGN=ACPPPE=1SV=3

MRAAPLLLARAASLSLGFLFLLFFWLDRSVLAKELKFVTLVFRHGDRSPIDTFPTDPIKE

SSWPQGFGQLTQLGMEQHYELGEYIRKRYRKFLNESYKHEQVYIRSTDVDRTLMSAMTNL

AALFPPEGVSIWNPILLWQPIPVHTVPLSEDQLLYLPFRNCPRFQELESETLKSEEFQKR

LHPYKDFIATLGKLSGLHGQDLFGIWSKVYDPLYCESVHNFTLPSWATEDTMTKLRELSE

LSLLSLYGIHKQKEKSRLQGGVLVNEILNHMKRATQIPSYKKLIMYSAHDTTVSGLQMAL

DVYNGLLPPYASCHLTELYFEKGEYFVEMYYRNETQHEPYPLMLPGCSPSCPLERFAELV

GPVIPQDWSTECMTTNSHQGTEDSTD

356.>sp|Q08431|MFGM_HUMANLactadherinOS=HomosapiensGN=MFGE8PE=1SV=2

MPRPRLLAALCGALLCAPSLLVALDICSKNPCHNGGLCEEISQEVRGDVFPSYTCTCLKG

YAGNHCETKCVEPLGMENGNIANSQIAASSVRVTFLGLQHWVPELARLNRAGMVNAWTPS

SNDDNPWIQVNLLRRMWVTGVVTQGASRLASHEYLKAFKVAYSLNGHEFDFIHDVNKKHK

EFVGNWNKNAVHVNLFETPVEAQYVRLYPTSCHTACTLRFELLGCELNGCANPLGLKNNS

IPDKQITASSSYKTWGLHLFSWNPSYARLDKQGNFNAWVAGSYGNDQWLQVDLGSSKEVT

GIITQGARNFGSVQFVASYKVAYSNDSANWTEYQDPRTGSSKIFPGNWDNHSHKKNLFET

PILARYVRILPVAWHNRIALRLELLGC

357.>sp|Q9P1W8|SIRPG_HUMANSignal-regulatoryproteingammaOS=HomosapiensGN=SIRPGPE=1SV=3

MPVPASWPHPPGPFLLLTLLLGLTEVAGEEELQMIQPEKLLLVTVGKTATLHCTVTSLLP

VGPVLWFRGVGPGRELIYNQKEGHFPRVTTVSDLTKRNNMDFSIRISSITPADVGTYYCV

KFRKGSPENVEFKSGPGTEMALGAKPSAPVVLGPAARTTPEHTVSFTCESHGFSPRDITL

KWFKNGNELSDFQTNVDPTGQSVAYSIRSTARVVLDPWDVRSQVICEVAHVTLQGDPLRG

TANLSEAIRVPPTLEVTQQPMRVGNQVNVTCQVRKFYPQSLQLTWSENGNVCQRETASTL

TENKDGTYNWTSWFLVNISDQRDDVVLTCQVKHDGQLAVSKRLALEVTVHQKDQSSDATP

GPASSLTALLLIAVLLGPIYVPWKQKT

(2^nd^NinO.R)

358.>sp|Q9H488|OFUT1_HUMANGDP-fucoseproteinO-fucosyltransferase1OS=HomosapiensGN=POFUT1PE=1SV=1

MGAAAWARPLSVSFLLLLLPLPGMPAGSWDPAGYLLYCPCMGRFGNQADHFLGSLAFAKL

LNRTLAVPPWIEYQHHKPPFTNLHVSYQKYFKLEPLQAYHRVISLEDFMEKLAPTHWPPE

KRVAYCFEVAAQRSPDKKTCPMKEGNPFGPFWDQFHVSFNKSELFTGISFSASYREQWSQ

RFSPKEHPVLALPGAPAQFPVLEEHRPLQKYMVWSDEMVKTGEAQIHAHLVRPYVGIHLR

IGSDWKNACAMLKDGTAGSHFMASPQCVGYSRSTAAPLTMTMCLPDLKEIQRAVKLWVRS

LDAQSVYVATDSESYVPELQQLFKGKVKVVSLKPEVAQVDLYILGQADHFIGNCVSSFTA

FVKRERDLQGRPSSFFGMDRPPKLRDEF

359.>sp|Q99571|P2RX4_HUMANP2Xpurinoceptor4OS=HomosapiensGN=P2RX4PE=1SV=2

MAGCCAALAAFLFEYDTPRIVLIRSRKVGLMNRAVQLLILAYVIGWVFVWEKGYQETDSV

VSSVTTKVKGVAVTNTSKLGFRIWDVADYVIPAQEENSLFVMTNVILTMNQTQGLCPEIP

DATTVCKSDASCTAGSAGTHSNGVSTGRCVAFNGSVKTCEVAAWCPVEDDTHVPQPAFLK

AAENFTLLVKNNIWYPKFNFSKRNILPNITTTYLKSCIYDAKTDPFCPIFRLGKIVENAG

HSFQDMAVEGGIMGIQVNWDCNLDRAASLCLPRYSFRRLDTRDVEHNVSPGYNFRFAKYY

RDLAGNEQRTLIKAYGIRFDIIVFGKAGKFDIIPTMINIGSGLALLGMATVLCDIIVLYC

MKKRLYYREKKYKYVEDYEQGLASELDQ

360.>sp|Q96AP7|ESAM_HUMANEndothelialcell-selectiveadhesionmoleculeOS=HomosapiensGN=ESAMPE=1SV=1

MISLPGPLVTNLLRFLFLGLSALAPPSRAQLQLHLPANRLQAVEGGEVVLPAWYTLHGEV

SSSQPWEVPFVMWFFKQKEKEDQVLSYINGVTTSKPGVSLVYSMPSRNLSLRLEGLQEKD

SGPYSCSVNVQDKQGKSRGHSIKTLELNVLVPPAPPSCRLQGVPHVGANVTLSCQSPRSK

PAVQYQWDRQLPSFQTFFAPALDVIRGSLSLTNLSSSMAGVYVCKAHNEVGTAQCNVTLE

VSTGPGAAVVAGAVVGTLVGLGLLAGLVLLYHRRGKALEEPANDIKEDAIAPRTLPWPKS

SDTISKNGTLSSVTSARALRPPHGPPRPGALTPTPSLSSQALPSPRLPTTDGAHPQPISP

IPGGVSSSGLSRMGAVPVMVPAQSQAGSLV

361.>sp|P01137|TGFB1_HUMANTransforminggrowthfactorbeta-1OS=HomosapiensGN=TGFB1PE=1SV=2

MPPSGLRLLLLLLPLLWLLVLTPGRPAAGLSTCKTIDMELVKRKRIEAIRGQILSKLRLA

SPPSQGEVPPGPLPEAVLALYNSTRDRVAGESAEPEPEPEADYYAKEVTRVLMVETHNEI

YDKFKQSTHSIYMFFNTSELREAVPEPVLLSRAELRLLRLKLKVEQHVELYQKYSNNSWR

YLSNRLLAPSDSPEWLSFDVTGVVRQWLSRGGEIEGFRLSAHCSCDSRDNTLQVDINGFT

TGRRGDLATIHGMNRPFLLLMATPLERAQHLQSSRHRRALDTNYCFSSTEKNCCVRQLYI

DFRKDLGWKWIHEPKGYHANFCLGPCPYIWSLDTQYSKVLALYNQHNPGASAAPCCVPQA

LEPLPIVYYVGRKPKVEQLSNMIVRSCKCS

362.>sp|P48048|KCNJ1_HUMANATP-sensitiveinwardrectifierpotassiumchannel1OS=HomosapiensGN=KCNJ1PE=1SV=1

MNASSRNVFDTLIRVLTESMFKHLRKWVVTRFFGHSRQRARLVSKDGRCNIEFGNVEAQS

RFIFFVDIWTTVLDLKWRYKMTIFITAFLGSWFFFGLLWYAVAYIHKDLPEFHPSANHTP

CVENINGLTSAFLFSLETQVTIGYGFRCVTEQCATAIFLLIFQSILGVIINSFMCGAILA

KISRPKKRAKTITFSKNAVISKRGGKLCLLIRVANLRKSLLIGSHIYGKLLKTTVTPEGE

TIILDQININFVVDAGNENLFFISPLTIYHVIDHNSPFFHMAAETLLQQDFELVVFLDGT

VESTSATCQVRTSYVPEEVLWGYRFAPIVSKTKEGKYRVDFHNFSKTVEVETPHCAMCLY

NEKDVRARMKRGYDNPNFILSEVNETDDTKM

363.>sp|P04220|MUCB_HUMANIgmuheavychaindiseaseproteinOS=HomosapiensPE=1SV=1

DSPLEQSGHEVGILKETEAEDRIIKEEEARLSGRDMQVTSQPVIAELPPKVSVFVPPRDG

FFGNPRKSKLICQATGFSPRQIEVSWLREGKQVGSGVTTDEVEAEAKESGPTTYKVTSTL

TIKESDWLGQSMFTCRVDHRGLTFQQNASSMCGPDQDTAIRVFAIPPSFASIFLTKSTKL

TCLVTDLTTYDSVTISWTRQDGEAVKTHTNISESHPNATFSAVGEASICEDDWDSGERFT

CTVTHTDLPSPLKQTISRPKGVALHRPDVYLLPPAREQLNLRESATITCLVTGFSPADVF

VQWMQRGQPLSPEKYVTSAPMPEPQAPGRYFAHSILTVSEEEWNTGETYTCVVAHEALPN

RVTERTVDKSTGKPTLYNVSLVMSDTAGTCY

364.>sp|Q93097|WNT2B_HUMANProteinWnt-2bOS=HomosapiensGN=WNT2BPE=1SV=2

MLRPGGAEEAAQLPLRRASAPVPVPSPAAPDGSRASARLGLACLLLLLLLTLPARVDTSW

WYIGALGARVICDNIPGLVSRQRQLCQRYPDIMRSVGEGAREWIRECQHQFRHHRWNCTT

LDRDHTVFGRVMLRSSREAAFVYAISSAGVVHAITRACSQGELSVCSCDPYTRGRHHDQR

GDFDWGGCSDNIHYGVRFAKAFVDAKEKRLKDARALMNLHNNRCGRTAVRRFLKLECKCH

GVSGSCTLRTCWRALSDFRRTGDYLRRRYDGAVQVMATQDGANFTAARQGYRRATRTDLV

YFDNSPDYCVLDKAAGSLGTAGRVCSKTSKGTDGCEIMCCGRGYDTTRVTRVTQCECKFH

WCCAVRCKECRNTVDVHTCKAPKKAEWLDQT

365.>sp|P15529|MCP_HUMANMembranecofactorproteinOS=HomosapiensGN=CD46PE=1SV=3

MEPPGRRECPFPSWRFPGLLLAAMVLLLYSFSDACEEPPTFEAMELIGKPKPYYEIGERV

DYKCKKGYFYIPPLATHTICDRNHTWLPVSDDACYRETCPYIRDPLNGQAVPANGTYEFG

YQMHFICNEGYYLIGEEILYCELKGSVAIWSGKPPICEKVLCTPPPKIKNGKHTFSEVEV

FEYLDAVTYSCDPAPGPDPFSLIGESTIYCGDNSVWSRAAPECKVVKCRFPVVENGKQIS

GFGKKFYYKATVMFECDKGFYLDGSDTIVCDSNSTWDPPVPKCLKVLPPSSTKPPALSHS

VSTSSTTKSPASSASGPRPTYKPPVSNYPGYPKPEEGILDSLDVWVIAVIVIAIVVGVAV

ICVVPYRYLQRRKKKGTYLTDETHREVKFTSL

366.>sp|Q13510|ASAH1_HUMANAcidceramidaseOS=HomosapiensGN=ASAH1PE=1SV=5

MPGRSCVALVLLAAAVSCAVAQHAPPWTEDCRKSTYPPSGPTYRGAVPWYTINLDLPPYK

RWHELMLDKAPVLKVIVNSLKNMINTFVPSGKIMQVVDEKLPGLLGNFPGPFEEEMKGIA

AVTDIPLGEIISFNIFYELFTICTSIVAEDKKGHLIHGRNMDFGVFLGWNINNDTWVITE

QLKPLTVNLDFQRNNKTVFKASSFAGYVGMLTGFKPGLFSLTLNERFSINGGYLGILEWI

LGKKDVMWIGFLTRTVLENSTSYEEAKNLLTKTKILAPAYFILGGNQSGEGCVITRDRKE

SLDVYELDAKQGRWYVVQTNYDRWKHPFFLDDRRTPAKMCLNRTSQENISFETMYDVLST

KPVLNKLTVYTTLIDVTKGQFETYLRDCPDPCIGW

367.>sp|P12643|BMP2_HUMANBonemorphogeneticprotein2OS=HomosapiensGN=BMP2PE=1SV=1

MVAGTRCLLALLLPQVLLGGAAGLVPELGRRKFAAASSGRPSSQPSDEVLSEFELRLLSM

FGLKQRPTPSRDAVVPPYMLDLYRRHSGQPGSPAPDHRLERAASRANTVRSFHHEESLEE

LPETSGKTTRRFFFNLSSIPTEEFITSAELQVFREQMQDALGNNSSFHHRINIYEIIKPA

TANSKFPVTRLLDTRLVNQNASRWESFDVTPAVMRWTAQGHANHGFVVEVAHLEEKQGVS

KRHVRISRSLHQDEHSWSQIRPLLVTFGHDGKGHPLHKREKRQAKHKQRKRLKSSCKRHP

LYVDFSDVGWNDWIVAPPGYHAFYCHGECPFPLADHLNSTNHAIVQTLVNSVNSKIPKAC

CVPTELSAISMLYLDENEKVVLKNYQDMVVEGCGCR

368.>sp|Q9NY97|B3GN2_HUMANN-acetyllactosaminidebeta-1,3-N-acetylglucosaminyltransferase2OS=HomosapiensGN=B3GNT2PE=1SV=2

MSVGRRRIKLLGILMMANVFIYFIMEVSKSSSQEKNGKGEVIIPKEKFWKISTPPEAYWN

REQEKLNRQYNPILSMLTNQTGEAGRLSNISHLNYCEPDLRVTSVVTGFNNLPDRFKDFL

LYLRCRNYSLLIDQPDKCAKKPFLLLAIKSLTPHFARRQAIRESWGQESNAGNQTVVRVF

LLGQTPPEDNHPDLSDMLKFESEKHQDILMWNYRDTFFNLSLKEVLFLRWVSTSCPDTEF

VFKGDDDVFVNTHHILNYLNSLSKTKAKDLFIGDVIHNAGPHRDKKLKYYIPEVVYSGLY

PPYAGGGGFLYSGHLALRLYHITDQVHLYPIDDVYTGMCLQKLGLVPEKHKGFRTFDIEE

KNKNNICSYVDLMLVHSRKPQEMIDIWSQLQSAH

369.>sp|P55085|PAR2_HUMANProteinase-activatedreceptor2OS=HomosapiensGN=F2RL1PE=1SV=1

MRSPSAAWLLGAAILLAASLSCSGTIQGTNRSSKGRSLIGKVDGTSHVTGKGVTVETVFS

VDEFSASVLTGKLTTVFLPIVYTIVFVVGLPSNGMALWVFLFRTKKKHPAVIYMANLALA

DLLSVIWFPLKIAYHIHGNNWIYGEALCNVLIGFFYGNMYCSILFMTCLSVQRYWVIVNP

MGHSRKKANIAIGISLAIWLLILLVTIPLYVVKQTIFIPALNITTCHDVLPEQLLVGDMF

NYFLSLAIGVFLFPAFLTASAYVLMIRMLRSSAMDENSEKKRKRAIKLIVTVLAMYLICF

TPSNLLLVVHYFLIKSQGQSHVYALYIVALCLSTLNSCIDPFVYYFVSHDFRDHAKNALL

CRSVRTVKQMQVSLTSKKHSRKSSSYSSSSTTVKTSY

370.>sp|O14791|APOL1_HUMANApolipoproteinL1OS=HomosapiensGN=APOL1PE=1SV=5

MEGAALLRVSVLCIWMSALFLGVGVRAEEAGARVQQNVPSGTDTGDPQSKPLGDWAAGTM

DPESSIFIEDAIKYFKEKVSTQNLLLLLTDNEAWNGFVAAAELPRNEADELRKALDNLAR

QMIMKDKNWHDKGQQYRNWFLKEFPRLKSELEDNIRRLRALADGVQKVHKGTTIANVVSG

SLSISSGILTLVGMGLAPFTEGGSLVLLEPGMELGITAALTGITSSTMDYGKKWWTQAQA

HDLVIKSLDKLKEVREFLGENISNFLSLAGNTYQLTRGIGKDIRALRRARANLQSVPHAS

ASRPRVTEPISAESGEQVERVNEPSILEMSRGVKLTDVAPVSFFLVLDVVYLVYESKHLH

EGAKSETAEELKKVAQELEEKLNILNNNYKILQADQEL

371.>sp|Q96G97|BSCL2_HUMANSeipinOS=HomosapiensGN=BSCL2PE=1SV=3

MVNDPPVPALLWAQEVGQVLAGRARRLLLQFGVLFCTILLLLWVSVFLYGSFYYSYMPTV

SHLSPVHFYYRTDCDSSTTSLCSFPVANVSLTKGGRDRVLMYGQPYRVTLELELPESPVN

QDLGMFLVTISCYTRGGRIISTSSRSVMLHYRSDLLQMLDTLVFSSLLLFGFAEQKQLLE

VELYADYRENSYVPTTGAIIEIHSKRIQLYGAYLRIHAHFTGLRYLLYNFPMTCAFIGVA

SNFTFLSVIVLFSYMQWVWGGIWPRHRFSLQVNIRKRDNSRKEVQRRISAHQPGPEGQEE

STPQSDVTEDGESPEDPSGTEGQLSEEEKPDQQPLSGEEELEPEASDGSGSWEDAALLTE

ANLPAPAPASASAPVLETLGSSEPAGGALRQRPTCSSS

372.>sp|Q8N126|CADM3_HUMANCelladhesionmolecule3OS=HomosapiensGN=CADM3PE=1SV=1

MGAPAASLLLLLLLFACCWAPGGANLSQDDSQPWTSDETVVAGGTVVLKCQVKDHEDSSL

QWSNPAQQTLYFGEKRALRDNRIQLVTSTPHELSISISNVALADEGEYTCSIFTMPVRTA

KSLVTVLGIPQKPIITGYKSSLREKDTATLNCQSSGSKPAARLTWRKGDQELHGEPTRIQ

EDPNGKTFTVSSSVTFQVTREDDGASIVCSVNHESLKGADRSTSQRIEVLYTPTAMIRPD

PPHPREGQKLLLHCEGRGNPVPQQYLWEKEGSVPPLKMTQESALIFPFLNKSDSGTYGCT

ATSNMGSYKAYYTLNVNDPSPVPSSSSTYHAIIGGIVAFIVFLLLIMLIFLGHYLIRHKG

TYLTHEAKGSDDAPDADTAIINAEGGQSGGDDKKEYFI

373.>sp|P27930|IL1R2_HUMANInterleukin-1receptortype2OS=HomosapiensGN=IL1R2PE=1SV=1

MLRLYVLVMGVSAFTLQPAAHTGAARSCRFRGRHYKREFRLEGEPVALRCPQVPYWLWAS

VSPRINLTWHKNDSARTVPGEEETRMWAQDGALWLLPALQEDSGTYVCTTRNASYCDKMS

IELRVFENTDAFLPFISYPQILTLSTSGVLVCPDLSEFTRDKTDVKIQWYKDSLLLDKDN

EKFLSVRGTTHLLVHDVALEDAGYYRCVLTFAHEGQQYNITRSIELRIKKKKEETIPVII

SPLKTISASLGSRLTIPCKVFLGTGTPLTTMLWWTANDTHIESAYPGGRVTEGPRQEYSE

NNENYIEVPLIFDPVTREDLHMDFKCVVHNTLSFQTLRTTVKEASSTFSWGIVLAPLSLA

FLVLGGIWMHRRCKHRTGKADGLTVLWPHHQDFQSYPK

374.>sp|P07098|LIPG_HUMANGastrictriacylglycerollipaseOS=HomosapiensGN=LIPFPE=1SV=1

MWLLLTMASLISVLGTTHGLFGKLHPGSPEVTMNISQMITYWGYPNEEYEVVTEDGYILE

VNRIPYGKKNSGNTGQRPVVFLQHGLLASATNWISNLPNNSLAFILADAGYDVWLGNSRG

NTWARRNLYYSPDSVEFWAFSFDEMAKYDLPATIDFIVKKTGQKQLHYVGHSQGTTIGFI

AFSTNPSLAKRIKTFYALAPVATVKYTKSLINKLRFVPQSLFKFIFGDKIFYPHNFFDQF

LATEVCSREMLNLLCSNALFIICGFDSKNFNTSRLDVYLSHNPAGTSVQNMFHWTQAVKS

GKFQAYDWGSPVQNRMHYDQSQPPYYNVTAMNVPIAVWNGGKDLLADPQDVGLLLPKLPN

LIYHKEIPFYNHLDFIWAMDAPQEVYNDIVSMISEDKK

375.>sp|Q9Y639|NPTN_HUMANNeuroplastinOS=HomosapiensGN=NPTNPE=1SV=2

MSGSSLPSALALSLLLVSGSLLPGPGAAQNAGFVKSPMSETKLTGDAFELYCDVVGSPTP

EIQWWYAEVNRAESFRQLWDGARKRRVTVNTAYGSNGVSVLRITRLTLEDSGTYECRASN

DPKRNDLRQNPSITWIRAQATISVLQKPRIVTSEEVIIRDSPVLPVTLQCNLTSSSHTLT

YSYWTKNGVELSATRKNASNMEYRINKPRAEDSGEYHCVYHFVSAPKANATIEVKAAPDI

TGHKRSENKNEGQDATMYCKSVGYPHPDWIWRKKENGMPMDIVNTSGRFFIINKENYTEL

NIVNLQITEDPGEYECNATNAIGSASVVTVLRVRSHLAPLWPFLGILAEIIILVVIIVVY

EKRKRPDEVPDDDEPAGPMKTNSTNNHKDKNLRQRNTN

376.>sp|O00241|SIRB1_HUMANSignal-regulatoryproteinbeta-1OS=HomosapiensGN=SIRPB1PE=1SV=5

MPVPASWPHLPSPFLLMTLLLGRLTGVAGEDELQVIQPEKSVSVAAGESATLRCAMTSLI

PVGPIMWFRGAGAGRELIYNQKEGHFPRVTTVSELTKRNNLDFSISISNITPADAGTYYC

VKFRKGSPDDVEFKSGAGTELSVRAKPSAPVVSGPAVRATPEHTVSFTCESHGFSPRDIT

LKWFKNGNELSDFQTNVDPAGDSVSYSIHSTARVVLTRGDVHSQVICEIAHITLQGDPLR

GTANLSEAIRVPPTLEVTQQPMRAENQANVTCQVSNFYPRGLQLTWLENGNVSRTETAST

LIENKDGTYNWMSWLLVNTCAHRDDVVLTCQVEHDGQQAVSKSYALEISAHQKEHGSDIT

HEAALAPTAPLLVALLLGPKLLLVVGVSAIYICWKQKA

(SecondNinO.R.)

377.>sp|P22760|AAAD_HUMANArylacetamidedeacetylaseOS=HomosapiensGN=AADACPE=1SV=5

MGRKSLYLLIVGILIAYYIYTPLPDNVEEPWRMMWINAHLKTIQNLATFVELLGLHHFMD

SFKVVGSFDEVPPTSDENVTVTETKFNNILVRVYVPKRKSEALRRGLFYIHGGGWCVGSA

ALSGYDLLSRWTADRLDAVVVSTNYRLAPKYHFPIQFEDVYNALRWFLRKKVLAKYGVNP

ERIGISGDSAGGNLAAAVTQQLLDDPDVKIKLKIQSLIYPALQPLDVDLPSYQENSNFLF

LSKSLMVRFWSEYFTTDRSLEKAMLSRQHVPVESSHLFKFVNWSSLLPERFIKGHVYNNP

NYGSSELAKKYPGFLDVRAAPLLADDNKLRGLPLTYVITCQYDLLRDDGLMYVTRLRNTG

VQVTHNHVEDGFHGAFSFLGLKISHRLINQYIEWLKENL

378.>sp|P38571|LICH_HUMANLysosomalacidlipase/cholesterylesterhydrolaseOS=HomosapiensGN=LIPAPE=1SV=2

MKMRFLGLVVCLVLWTLHSEGSGGKLTAVDPETNMNVSEIISYWGFPSEEYLVETEDGYI

LCLNRIPHGRKNHSDKGPKPVVFLQHGLLADSSNWVTNLANSSLGFILADAGFDVWMGNS

RGNTWSRKHKTLSVSQDEFWAFSYDEMAKYDLPASINFILNKTGQEQVYYVGHSQGTTIG

FIAFSQIPELAKRIKMFFALGPVASVAFCTSPMAKLGRLPDHLIKDLFGDKEFLPQSAFL

KWLGTHVCTHVILKELCGNLCFLLCGFNERNLNMSRVDVYTTHSPAGTSVQNMLHWSQAV

KFQKFQAFDWGSSAKNYFHYNQSYPPTYNVKDMLVPTAVWSGGHDWLADVYDVNILLTQI

TNLVFHESIPEWEHLDFIWGLDAPWRLYNKIINLMRKYQ

379.>sp|P15509|CSF2R_HUMANGranulocyte-macrophagecolony-stimulatingfactorreceptorsubunitalphaOS=HomosapiensGN=CSF2RAPE=1SV=1

MLLLVTSLLLCELPHPAFLLIPEKSDLRTVAPASSLNVRFDSRTMNLSWDCQENTTFSKC

FLTDKKNRVVEPRLSNNECSCTFREICLHEGVTFEVHVNTSQRGFQQKLLYPNSGREGTA

AQNFSCFIYNADLMNCTWARGPTAPRDVQYFLYIRNSKRRREIRCPYYIQDSGTHVGCHL

DNLSGLTSRNYFLVNGTSREIGIQFFDSLLDTKKIERFNPPSNVTVRCNTTHCLVRWKQP

RTYQKLSYLDFQYQLDVHRKNTQPGTENLLINVSGDLENRYNFPSSEPRAKHSVKIRAAD

VRILNWSSWSEAIEFGSDDGNLGSVYIYVLLIVGTLVCGIVLGFLFKRFLRIQRLFPPVP

QIKDKLNDNHEVEDEIIWEEFTPEEGKGYREEVLTVKEIT

380.>sp|O60609|GFRA3_HUMANGDNFfamilyreceptoralpha-3OS=HomosapiensGN=GFRA3PE=1SV=2

MVRPLNPRPLPPVVLMLLLLLPPSPLPLAAGDPLPTESRLMNSCLQARRKCQADPTCSAA

YHHLDSCTSSISTPLPSEEPSVPADCLEAAQQLRNSSLIGCMCHRRMKNQVACLDIYWTV

HRARSLGNYELDVSPYEDTVTSKPWKMNLSKLNMLKPDSDLCLKFAMLCTLNDKCDRLRK

AYGEACSGPHCQRHVCLRQLLTFFEKAAEPHAQGLLLCPCAPNDRGCGERRRNTIAPNCA

LPPVAPNCLELRRLCFSDPLCRSRLVDFQTHCHPMDILGTCATEQSRCLRAYLGLIGTAM

TPNFVSNVNTSVALSCTCRGSGNLQEECEMLEGFFSHNPCLTEAIAAKMRFHSQLFSQDW

PHPTFAVMAHQNENPAVRPQPWVPSLFSCTLPLILLLSLW

381.>sp|P16150|LEUK_HUMANLeukosialinOS=HomosapiensGN=SPNPE=1SV=1

MATLLLLLGVLVVSPDALGSTTAVQTPTSGEPLVSTSEPLSSKMYTTSITSDPKADSTGD

QTSALPPSTSINEGSPLWTSIGASTGSPLPEPTTYQEVSIKMSSVPQETPHATSHPAVPI

TANSLGSHTVTGGTITTNSPETSSRTSGAPVTTAASSLETSRGTSGPPLTMATVSLETSK

GTSGPPVTMATDSLETSTGTTGPPVTMTTGSLEPSSGASGPQVSSVKLSTMMSPTTSTNA

STVPFRNPDENSRGMLPVAVLVALLAVIVLVALLLLWRRRQKRRTGALVLSRGGKRNGVV

DAWAGPAQVPEEGAVTVTVGGSGGDKGSGFPDGEGSSRRPTLTTFFGRRKSRQGSLAMEE

LKSGSGPSLKGEEEPLVASEDGAVDAPAPDEPEGGDGAAP

382.>sp|P22891|PROZ_HUMANVitaminK-dependentproteinZOS=HomosapiensGN=PROZPE=1SV=2

MAGCVPLLQGLVLVLALHRVEPSVFLPASKANDVLVRWKRAGSYLLEELFEGNLEKECYE

EICVYEEAREVFENEVVTDEFWRRYKGGSPCISQPCLHNGSCQDSIWGYTCTCSPGYEGS

NCELAKNECHPERTDGCQHFCLPGQESYTCSCAQGYRLGEDHKQCVPHDQCACGVLTSEK

RAPDLQDLPWQVKLTNSEGKDFCGGVIIRENFVLTTAKCSLLHRNITVKTYFNRTSQDPL

MIKITHVHVHMRYDADAGENDLSLLELEWPIQCPGAGLPVCTPEKDFAEHLLIPRTRGLL

SGWARNGTDLGNSLTTRPVTLVEGEECGQVLNVTVTTRTYCERSSVAAMHWMDGSVVTRE

HRGSWFLTGVLGSQPVGGQAHMVLVTKVSRYSLWFKQIMN

383.>sp|P14091|CATE_HUMANCathepsinEOS=HomosapiensGN=CTSEPE=1SV=2

MKTLLLLLLVLLELGEAQGSLHRVPLRRHPSLKKKLRARSQLSEFWKSHNLDMIQFTESC

SMDQSAKEPLINYLDMEYFGTISIGSPPQNFTVIFDTGSSNLWVPSVYCTSPACKTHSRF

QPSQSSTYSQPGQSFSIQYGTGSLSGIIGADQVSAFATQVEGLTVVGQQFGESVTEPGQT

FVDAEFDGILGLGYPSLAVGGVTPVFDNMMAQNLVDLPMFSVYMSSNPEGGAGSELIFGG

YDHSHFSGSLNWVPVTKQAYWQIALDNIQVGGTVMFCSEGCQAIVDTGTSLITGPSDKIK

QLQNAIGAAPVDGEYAVECANLNVMPDVTFTINGVPYTLSPTAYTLLDFVDGMQFCSSGF

QGLDIHPPAGPLWILGDVFIRQFYSVFDRGNNRVGLAPAVP

384.>sp|Q8NBJ4|GOLM1_HUMANGolgimembraneprotein1OS=HomosapiensGN=GOLM1PE=1SV=1

MMGLGNGRRSMKSPPLVLAALVACIIVLGFNYWIASSRSVDLQTRIMELEGRVRRAAAER

GAVELKKNEFQGELEKQREQLDKIQSSHNFQLESVNKLYQDEKAVLVNNITTGERLIRVL

QDQLKTLQRNYGRLQQDVLQFQKNQTNLERKFSYDLSQCINQMKEVKEQCEERIEEVTKK

GNEAVASRDLSENNDQRQQLQALSEPQPRLQAAGLPHTEVPQGKGNVLGNSKSQTPAPSS

EVVLDSKRQVEKEETNEIQVVNEEPQRDRLPQEPGREQVVEDRPVGGRGFGGAGELGQTP

QVQAALSVSQENPEMEGPERDQLVIPDGQEEEQEAAGEGRNQQKLRGEDDYNMDENEAES

ETDKQAALAGNDRNIDVFNVEDQKRDTINLLDQREKRNHTL

385.>sp|O00300|TR11B_HUMANTumornecrosisfactorreceptorsuperfamilymember11BOS=HomosapiensGN=TNFRSF11BPE=1SV=3

MNNLLCCALVFLDISIKWTTQETFPPKYLHYDEETSHQLLCDKCPPGTYLKQHCTAKWKT

VCAPCPDHYYTDSWHTSDECLYCSPVCKELQYVKQECNRTHNRVCECKEGRYLEIEFCLK

HRSCPPGFGVVQAGTPERNTVCKRCPDGFFSNETSSKAPCRKHTNCSVFGLLLTQKGNAT

HDNICSGNSESTQKCGIDVTLCEEAFFRFAVPTKFTPNWLSVLVDNLPGTKVNAESVERI

KRQHSSQEQTFQLLKLWKHQNKDQDIVKKIIQDIDLCENSVQRHIGHANLTFEQLRSLME

SLPGKKVGAEDIEKTIKACKPSDQILKLLSLWRIKNGDQDTLKGLMHALKHSKTYHFPKT

VTQSLKKTIRFLHSFTMYKLYQKLFLEMIGNQVQSVKISCL

386.>sp|P05121|PAI1_HUMANPlasminogenactivatorinhibitor1OS=HomosapiensGN=SERPINE1PE=1SV=1

MQMSPALTCLVLGLALVFGEGSAVHHPPSYVAHLASDFGVRVFQQVAQASKDRNVVFSPY

GVASVLAMLQLTTGGETQQQIQAAMGFKIDDKGMAPALRHLYKELMGPWNKDEISTTDAI

FVQRDLKLVQGFMPHFFRLFRSTVKQVDFSEVERARFIINDWVKTHTKGMISNLLGKGAV

DQLTRLVLVNALYFNGQWKTPFPDSSTHRRLFHKSDGSTVSVPMMAQTNKFNYTEFTTPD

GHYYDILELPYHGDTLSMFIAAPYEKEVPLSALTNILSAQLISHWKGNMTRLPRLLVLPK

FSLETEVDLRKPLENLGMTDMFRQFQADFTSLSDQEPLHVAQALQKVKIEVNESGTVASS

STAVIVSARMAPEEIIMDRPFLFVVRHNPTGTVLFMGQVMEP

387.>sp|P04278|SHBG_HUMANSexhormone-bindingglobulinOS=HomosapiensGN=SHBGPE=1SV=2

MESRGPLATSRLLLLLLLLLLRHTRQGWALRPVLPTQSAHDPPAVHLSNGPGQEPIAVMT

FDLTKITKTSSSFEVRTWDPEGVIFYGDTNPKDDWFMLGLRDGRPEIQLHNHWAQLTVGA

GPRLDDGRWHQVEVKMEGDSVLLEVDGEEVLRLRQVSGPLTSKRHPIMRIALGGLLFPAS

NLRLPLVPALDGCLRRDSWLDKQAEISASAPTSLRSCDVESNPGIFLPPGTQAEFNLRDI

PQPHAEPWAFSLDLGLKQAAGSGHLLALGTPENPSWLSLHLQDQKVVLSSGSGPGLDLPL

VLGLPLQLKLSMSRVVLSQGSKMKALALPPLGLAPLLNLWAKPQGRLFLGALPGEDSSTS

FCLNGLWAQGQRLDVDQALNRSHEIWTHSCPQSPGNGTDASH

(1^st^NinO.R)

388.>sp|P08185|CBG_HUMANCorticosteroid-bindingglobulinOS=HomosapiensGN=SERPINA6PE=1SV=1

MPLLLYTCLLWLPTSGLWTVQAMDPNAAYVNMSNHHRGLASANVDFAFSLYKHLVALSPK

KNIFISPVSISMALAMLSLGTCGHTRAQLLQGLGFNLTERSETEIHQGFQHLHQLFAKSD

TSLEMTMGNALFLDGSLELLESFSADIKHYYESEVLAMNFQDWATASRQINSYVKNKTQG

KIVDLFSGLDSPAILVLVNYIFFKGTWTQPFDLASTREENFYVDETTVVKVPMMLQSSTI

SYLHDSELPCQLVQMNYVGNGTVFFILPDKGKMNTVIAALSRDTINRWSAGLTSSQVDLY

IPKVTISGVYDLGDVLEEMGIADLFTNQANFSRITQDAQLKSSKVVHKAVLQLNEEGVDT

AGSTGVTLNLTSKPIILRFNQPFIIMIFDHFTWSSLFLARVMNPV

389.>sp|P00738|HPT_HUMANHaptoglobinOS=HomosapiensGN=HPPE=1SV=1

MSALGAVIALLLWGQLFAVDSGNDVTDIADDGCPKPPEIAHGYVEHSVRYQCKNYYKLRT

EGDGVYTLNDKKQWINKAVGDKLPECEADDGCPKPPEIAHGYVEHSVRYQCKNYYKLRTE

GDGVYTLNNEKQWINKAVGDKLPECEAVCGKPKNPANPVQRILGGHLDAKGSFPWQAKMV

SHHNLTTGATLINEQWLLTTAKNLFLNHSENATAKDIAPTLTLYVGKKQLVEIEKVVLHP

NYSQVDIGLIKLKQKVSVNERVMPICLPSKDYAEVGRVGYVSGWGRNANFKFTDHLKYVM

LPVADQDQCIRHYEGSTVPEKKTPKSPVGVQPILNEHTFCAGMSKYQEDTCYGDAGSAFA

VHDLEEDTWYATGILSFDKSCAVAEYGVYVKVTSIQDWVQKTIAEN

390.>sp|P05154|IPSP_HUMANPlasmaserineproteaseinhibitorOS=HomosapiensGN=SERPINA5PE=1SV=3

MQLFLLLCLVLLSPQGASLHRHHPREMKKRVEDLHVGATVAPSSRRDFTFDLYRALASAA

PSQSIFFSPVSISMSLAMLSLGAGSSTKMQILEGLGLNLQKSSEKELHRGFQQLLQELNQ

PRDGFQLSLGNALFTDLVVDLQDTFVSAMKTLYLADTFPTNFRDSAGAMKQINDYVAKQT

KGKIVDLLKNLDSNAVVIMVNYIFFKAKWETSFNHKGTQEQDFYVTSETVVRVPMMSRED

QYHYLLDRNLSCRVVGVPYQGNATALFILPSEGKMQQVENGLSEKTLRKWLKMFKKRQLE

LYLPKFSIEGSYQLEKVLPSLGISNVFTSHADLSGISNHSNIQVSEMVHKAVVEVDESGT

RAAAATGTIFTFRSARLNSQRLVFNRPFLMFIVDNNILFLGKVNRP

391.>sp|P00797|RENI_HUMANReninOS=HomosapiensGN=RENPE=1SV=1

MDGWRRMPRWGLLLLLWGSCTFGLPTDTTTFKRIFLKRMPSIRESLKERGVDMARLGPEW

SQPMKRLTLGNTTSSVILTNYMDTQYYGEIGIGTPPQTFKVVFDTGSSNVWVPSSKCSRL

YTACVYHKLFDASDSSSYKHNGTELTLRYSTGTVSGFLSQDIITVGGITVTQMFGEVTEM

PALPFMLAEFDGVVGMGFIEQAIGRVTPIFDNIISQGVLKEDVFSFYYNRDSENSQSLGG

QIVLGGSDPQHYEGNFHYINLIKTGVWQIQMKGVSVGSSTLLCEDGCLALVDTGASYISG

STSSIEKLMEALGAKKRLFDYVVKCNEGPTLPDISFHLGGKEYTLTSADYVFQESYSSKK

LCTLAIHAMDIPPPTGPTWALGATFIRKFYTEFDRRNNRIGFALAR

(1^st^NinD.R)

392.>sp|P15907|SIAT1_HUMANBeta-galactosidealpha-2,6-sialyltransferase1OS=HomosapiensGN=ST6GAL1PE=1SV=1

MIHTNLKKKFSCCVLVFLLFAVICVWKEKKKGSYYDSFKLQTKEFQVLKSLGKLAMGSDS

QSVSSSSTQDPHRGRQTLGSLRGLAKAKPEASFQVWNKDSSSKNLIPRLQKIWKNYLSMN

KYKVSYKGPGPGIKFSAEALRCHLRDHVNVSMVEVTDFPFNTSEWEGYLPKESIRTKAGP

WGRCAVVSSAGSLKSSQLGREIDDHDAVLRFNGAPTANFQQDVGTKTTIRLMNSQLVTTE

KRFLKDSLYNEGILIVWDPSVYHSDIPKWYQNPDYNFFNNYKTYRKLHPNQPFYILKPQM

PWELWDILQEISPEEIQPNPPSSGMLGIIIMMTLCDQVDIYEFLPSKRKTDVCYYYQKFF

DSACTMGAYHPLLYEKNLVKHLNQGTDEDIYLLGKATLPGFRTIHC

393.>sp|Q6PIU2|NCEH1_HUMANNeutralcholesterolesterhydrolase1OS=HomosapiensGN=NCEH1PE=1SV=3

MRSSCVLLTALVALAAYYVYIPLPGSVSDPWKLMLLDATFRGAQQVSNLIHYLGLSHHLL

ALNFIIVSFGKKSAWSSAQVKVTDTDFDGVEVRVFEGPPKPEEPLKRSVVYIHGGGWALA

SAKIRYYDELCTAMAEELNAVIVSIEYRLVPKVYFPEQIHDVVRATKYFLKPEVLQKYMV

DPGRICISGDSAGGNLAAALGQQFTQDASLKNKLKLQALIYPVLQALDFNTPSYQQNVNT

PILPRYVMVKYWVDYFKGNYDFVQAMIVNNHTSLDVEEAAAVRARLNWTSLLPASFTKNY

KPVVQTTGNARIVQELPQLLDARSAPLIADQAVLQLLPKTYILTCEHDVLRDDGIMYAKR

LESAGVEVTLDHFEDGFHGCMIFTSWPTNFSVGIRTRNSYIKWLDQNL

394.>sp|P13473|LAMP2_HUMANLysosome-associatedmembraneglycoprotein2OS=HomosapiensGN=LAMP2PE=1SV=2

MVCFRLFPVPGSGLVLVCLVLGAVRSYALELNLTDSENATCLYAKWQMNFTVRYETTNKT

YKTVTISDHGTVTYNGSICGDDQNGPKIAVQFGPGFSWIANFTKAASTYSIDSVSFSYNT

GDNTTFPDAEDKGILTVDELLAIRIPLNDLFRCNSLSTLEKNDVVQHYWDVLVQAFVQNG

TVSTNEFLCDKDKTSTVAPTIHTTVPSPTTTPTPKEKPEAGTYSVNNGNDTCLLATMGLQ

LNITQDKVASVININPNTTHSTGSCRSHTALLRLNSSTIKYLDFVFAVKNENRFYLKEVN

ISMYLVNGSVFSIANNNLSYWDAPLGSSYMCNKEQTVSVSGAFQINTFDLRVQPFNVTQG

KYSTAQDCSADDDNFLVPIAVGAALAGVLILVLLAYFIGLKHHHAGYEQF

(11^th^NinD.R)

395.>sp|P16444|DPEP1_HUMANDipeptidase1OS=HomosapiensGN=DPEP1PE=1SV=3

MWSGWWLWPLVAVCTADFFRDEAERIMRDSPVIDGHNDLPWQLLDMFNNRLQDERANLTT

LAGTHTNIPKLRAGFVGGQFWSVYTPCDTQNKDAVRRTLEQMDVVHRMCRMYPETFLYVT

SSAGIRQAFREGKVASLIGVEGGHSIDSSLGVLRALYQLGMRYLTLTHSCNTPWADNWLV

DTGDSEPQSQGLSPFGQRVVKELNRLGVLIDLAHVSVATMKATLQLSRAPVIFSHSSAYS

VCASRRNVPDDVLRLVKQTDSLVMVNFYNNYISCTNKANLSQVADHLDHIKEVAGARAVG

FGGDFDGVPRVPEGLEDVSKYPDLIAELLRRNWTEAEVKGALADNLLRVFEAVEQASNLT

QAPEEEPIPLDQLGGSCRTHYGYSSGASSLHRHWGLLLASLAPLVLCLSLL

396.>sp|Q14623|IHH_HUMANIndianhedgehogproteinOS=HomosapiensGN=IHHPE=1SV=4

MSPARLRPRLHFCLVLLLLLVVPAAWGCGPGRVVGSRRRPPRKLVPLAYKQFSPNVPEKT

LGASGRYEGKIARSSERFKELTPNYNPDIIFKDEENTGADRLMTQRCKDRLNSLAISVMN

QWPGVKLRVTEGWDEDGHHSEESLHYEGRAVDITTSDRDRNKYGLLARLAVEAGFDWVYY

ESKAHVHCSVKSEHSAAAKTGGCFPAGAQVRLESGARVALSAVRPGDRVLAMGEDGSPTF

SDVLIFLDREPHRLRAFQVIETQDPPRRLALTPAHLLFTADNHTEPAARFRATFASHVQP

GQYVLVAGVPGLQPARVAAVSTHVALGAYAPLTKHGTLVVEDVVASCFAAVADHHLAQLA

FWPLRLFHSLAWGSWTPGEGVHWYPQLLYRLGRLLLEEGSFHPLGMSGAGS

397.>sp|P17050|NAGAB_HUMANAlpha-N-acetylgalactosaminidaseOS=HomosapiensGN=NAGAPE=1SV=2

MLLKTVLLLGHVAQVLMLDNGLLQTPPMGWLAWERFRCNINCDEDPKNCISEQLFMEMAD

RMAQDGWRDMGYTYLNIDDCWIGGRDASGRLMPDPKRFPHGIPFLADYVHSLGLKLGIYA

DMGNFTCMGYPGTTLDKVVQDAQTFAEWKVDMLKLDGCFSTPEERAQGYPKMAAALNATG

RPIAFSCSWPAYEGGLPPRVNYSLLADICNLWRNYDDIQDSWWSVLSILNWFVEHQDILQ

PVAGPGHWNDPDMLLIGNFGLSLEQSRAQMALWTVLAAPLLMSTDLRTISAQNMDILQNP

LMIKINQDPLGIQGRRIHKEKSLIEVYMRPLSNKASALVFFSCRTDMPYRYHSSLGQLNF

TGSVIYEAQDVYSGDIISGLRDETNFTVIINPSGVVMWYLYPIKNLEMSQQ

398.>sp|P07339|CATD_HUMANCathepsinDOS=HomosapiensGN=CTSDPE=1SV=1

MQPSSLLPLALCLLAAPASALVRIPLHKFTSIRRTMSEVGGSVEDLIAKGPVSKYSQAVP

AVTEGPIPEVLKNYMDAQYYGEIGIGTPPQCFTVVFDTGSSNLWVPSIHCKLLDIACWIH

HKYNSDKSSTYVKNGTSFDIHYGSGSLSGYLSQDTVSVPCQSASSASALGGVKVERQVFG

EATKQPGITFIAAKFDGILGMAYPRISVNNVLPVFDNLMQQKLVDQNIFSFYLSRDPDAQ

PGGELMLGGTDSKYYKGSLSYLNVTRKAYWQVHLDQVEVASGLTLCKEGCEAIVDTGTSL

MVGPVDEVRELQKAIGAVPLIQGEYMIPCEKVSTLPAITLKLGGKGYKLSPEDYTLKVSQ

AGKTLCLSGFMGMDIPPPSGPLWILGDVFIGRYYTVFDRDNNRVGFAEAARL

399.>sp|Q8NCC3|PAG15_HUMANGroupXVphospholipaseA2OS=HomosapiensGN=PLA2G15PE=1SV=2

MGLHLRPYRVGLLPDGLLFLLLLLMLLADPALPAGRHPPVVLVPGDLGNQLEAKLDKPTV

VHYLCSKKTESYFTIWLNLELLLPVIIDCWIDNIRLVYNKTSRATQFPDGVDVRVPGFGK

TFSLEFLDPSKSSVGSYFHTMVESLVGWGYTRGEDVRGAPYDWRRAPNENGPYFLALREM

IEEMYQLYGGPVVLVAHSMGNMYTLYFLQRQPQAWKDKYIRAFVSLGAPWGGVAKTLRVL

ASGDNNRIPVIGPLKIREQQRSAVSTSWLLPYNYTWSPEKVFVQTPTINYTLRDYRKFFQ

DIGFEDGWLMRQDTEGLVEATMPPGVQLHCLYGTGVPTPDSFYYESFPDRDPKICFGDGD

GTVNLKSALQCQAWQSRQEHQVLLQELPGSEHIEMLANATTLAYLKRVLLGP

400.>sp|Q99519|NEUR1_HUMANSialidase-1OS=HomosapiensGN=NEU1PE=1SV=1

MTGERPSTALPDRRWGPRILGFWGGCRVWVFAAIFLLLSLAASWSKAENDFGLVQPLVTM

EQLLWVSGRQIGSVDTFRIPLITATPRGTLLAFAEARKMSSSDEGAKFIALRRSMDQGST

WSPTAFIVNDGDVPDGLNLGAVVSDVETGVVFLFYSLCAHKAGCQVASTMLVWSKDDGVS

WSTPRNLSLDIGTEVFAPGPGSGIQKQREPRKGRLIVCGHGTLERDGVFCLLSDDHGASW

RYGSGVSGIPYGQPKQENDFNPDECQPYELPDGSVVINARNQNNYHCHCRIVLRSYDACD

TLRPRDVTFDPELVDPVVAAGAVVTSSGIVFFSNPAHPEFRVNLTLRWSFSNGTSWRKET

VQLWPGPSGYSSLATLEGSMDGEEQAPQLYVLYEKGRNHYTESISVAKISVYGTL

401.>sp|P05543|THBG_HUMANThyroxine-bindingglobulinOS=HomosapiensGN=SERPINA7PE=1SV=2

MSPFLYLVLLVLGLHATIHCASPEGKVTACHSSQPNATLYKMSSINADFAFNLYRRFTVE

TPDKNIFFSPVSISAALVMLSFGACCSTQTEIVETLGFNLTDTPMVEIQHGFQHLICSLN

FPKKELELQIGNALFIGKHLKPLAKFLNDVKTLYETEVFSTDFSNISAAKQEINSHVEMQ

TKGKVVGLIQDLKPNTIMVLVNYIHFKAQWANPFDPSKTEDSSSFLIDKTTTVQVPMMHQ

MEQYYHLVDMELNCTVLQMDYSKNALALFVLPKEGQMESVEAAMSSKTLKKWNRLLQKGW

VDLFVPKFSISATYDLGATLLKMGIQHAYSENADFSGLTEDNGLKLSNAAHKAVLHIGEK

GTEAAAVPEVELSDQPENTFLHPIIQIDRSFMLLILERSTRSILFLGKVVNPTEA

(1^st^N in D.R.)

402.>sp|Q9HDC9|APMAP_HUMANAdipocyteplasmamembrane-associatedproteinOS=HomosapiensGN=APMAPPE=1SV=2

MSEADGLRQRRPLRPQVVTDDDGQAPEAKDGSSFSGRVFRVTFLMLAVSLTVPLLGAMML

LESPIDPQPLSFKEPPLLLGVLHPNTKLRQAERLFENQLVGPESIAHIGDVMFTGTADGR

VVKLENGEIETIARFGSGPCKTRDDEPVCGRPLGIRAGPNGTLFVADAYKGLFEVNPWKR

EVKLLLSSETPIEGKNMSFVNDLTVTQDGRKIYFTDSSSKWQRRDYLLLVMEGTDDGRLL

EYDTVTREVKVLLDQLRFPNGVQLSPAEDFVLVAETTMARIRRVYVSGLMKGGADLFVEN

MPGFPDNIRPSSSGGYWVGMSTIRPNPGFSMLDFLSERPWIKRMIFKLFSQETVMKFVPR

YSLVLELSDSGAFRRSLHDPDGLVATYISEVHEHDGHLYLGSFRSPFLCRLSLQAV

403.>sp|Q9UQV4|LAMP3_HUMANLysosome-associatedmembraneglycoprotein3OS=HomosapiensGN=LAMP3PE=1SV=3

MPRQLSAAAALFASLAVILHDGSQMRAKAFPETRDYSQPTAAATVQDIKKPVQQPAKQAP

HQTLAARFMDGHITFQTAATVKIPTTTPATTKNTATTSPITYTLVTTQATPNNSHTAPPV

TEVTVGPSLAPYSLPPTITPPAHTTGTSSSTVSHTTGNTTQPSNQTTLPATLSIALHKST

TGQKPVQPTHAPGTTAAAHNTTRTAAPASTVPGPTLAPQPSSVKTGIYQVLNGSRLCIKA

EMGIQLIVQDKESVFSPRRYFNIDPNATQASGNCGTRKSNLLLNFQGGFVNLTFTKDEES

YYISEVGAYLTVSDPETIYQGIKHAVVMFQTAVGHSFKCVSEQSLQLSAHLQVKTTDVQL

QAFDFEDDHFGNVDECSSDYTIVLPVIGAIVVGLCLMGMGVYKIRLRCQSSGYQRI

(2^nd^lastNinD.R)

404.>sp|P02812|PRB2_HUMANBasicsalivaryproline-richprotein2OS=HomosapiensGN=PRB2PE=1SV=3

MLLILLSVALLALSSAQNLNEDVSQEESPSLIAGNPQGAPPQGGNKPQGPPSPPGKPQGP

PPQGGNQPQGPPPPPGKPQGPPPQGGNKPQGPPPPGKPQGPPPQGDKSRSPRSPPGKPQG

PPPQGGNQPQGPPPPPGKPQGPPPQGGNKPQGPPPPGKPQGPPPQGDNKSRSSRSPPGKP

QGPPPQGGNQPQGPPPPPGKPQGPPPQGGNKPQGPPPPGKPQGPPPQGDNKSQSARSPPG

KPQGPPPQGGNQPQGPPPPPGKPQGPPPQGGNKSQGPPPPGKPQGPPPQGGSKSRSSRSP

PGKPQGPPPQGGNQPQGPPPPPGKPQGPPPQGGNKPQGPPPPGKPQGPPPQGGSKSRSAR

SPPGKPQGPPQQEGNNPQGPPPPAGGNPQQPQAPPAGQPQGPPRPPQGGRPSRPPQ

Disordered region not available.

405.>sp|P27797|CALR_HUMANCalreticulinOS=HomosapiensGN=CALRPE=1SV=1

MLLSVPLLLGLLGLAVAEPAVYFKEQFLDGDGWTSRWIESKHKSDFGKFVLSSGKFYGDE

EKDKGLQTSQDARFYALSASFEPFSNKGQTLVVQFTVKHEQNIDCGGGYVKLFPNSLDQT

DMHGDSEYNIMFGPDICGPGTKKVHVIFNYKGKNVLINKDIRCKDDEFTHLYTLIVRPDN

TYEVKIDNSQVESGSLEDDWDFLPPKKIKDPDASKPEDWDERAKIDDPTDSKPEDWDKPE

HIPDPDAKKPEDWDEEMDGEWEPPVIQNPEYKGEWKPRQIDNPDYKGTWIHPEIDNPEYS

PDPSIYAYDNFGVLGLDLWQVKSGTIFDNFLITNDEAYAEEFGNETWGVTKAAEKQMKDK

QDEEQRLKEEEEDKKRKEEEEAEDKEDDEDKDEDEEDEEDKEEDEEEDVPGQAKDEL

(NinO.R)

406.>sp|P05981|HEPS_HUMANSerineproteasehepsinOS=HomosapiensGN=HPNPE=1SV=1

MAQKEGGRTVPCCSRPKVAALTAGTLLLLTAIGAASWAIVAVLLRSDQEPLYPVQVSSAD

ARLMVFDKTEGTWRLLCSSRSNARVAGLSCEEMGFLRALTHSELDVRTAGANGTSGFFCV

DEGRLPHTQRLLEVISVCDCPRGRFLAAICQDCGRRKLPVDRIVGGRDTSLGRWPWQVSL

RYDGAHLCGGSLLSGDWVLTAAHCFPERNRVLSRWRVFAGAVAQASPHGLQLGVQAVVYH

GGYLPFRDPNSEENSNDIALVHLSSPLPLTEYIQPVCLPAAGQALVDGKICTVTGWGNTQ

YYGQQAGVLQEARVPIISNDVCNGADFYGNQIKPKMFCAGYPEGGIDACQGDSGGPFVCE

DSISRTPRWRLCGIVSWGTGCALAQKPGVYTKVSDFREWIFQAIKTHSEASGMVTQL

407.>sp|P27352|IF_HUMANGastricintrinsicfactorOS=HomosapiensGN=GIFPE=1SV=2

MAWFALYLLSLLWATAGTSTQTQSSCSVPSAQEPLVNGIQVLMENSVTSSAYPNPSILIA

MNLAGAYNLKAQKLLTYQLMSSDNNDLTIGQLGLTIMALTSSCRDPGDKVSILQRQMENW

APSSPNAEASAFYGPSLAILALCQKNSEATLPIAVRFAKTLLANSSPFNVDTGAMATLAL

TCMYNKIPVGSEEGYRSLFGQVLKDIVEKISMKIKDNGIIGDIYSTGLAMQALSVTPEPS

KKEWNCKKTTDMILNEIKQGKFHNPMSIAQILPSLKGKTYLDVPQVTCSPDHEVQPTLPS

NPGPGPTSASNITVIYTINNQLRGVELLFNETINVSVKSGSVLLVVLEEAQRKNPMFKFE

TTMTSWGLVVSSINNIAENVNHKTYWQFLSGVTPLNEGVADYIPFNHEHITANFTQY

408.>sp|P11279|LAMP1_HUMANLysosome-associatedmembraneglycoprotein1OS=HomosapiensGN=LAMP1PE=1SV=3

MAAPGSARRPLLLLLLLLLLGLMHCASAAMFMVKNGNGTACIMANFSAAFSVNYDTKSGP

KNMTFDLPSDATVVLNRSSCGKENTSDPSLVIAFGRGHTLTLNFTRNATRYSVQLMSFVY

NLSDTHLFPNASSKEIKTVESITDIRADIDKKYRCVSGTQVHMNNVTVTLHDATIQAYLS

NSSFSRGETRCEQDRPSPTTAPPAPPSPSPSPVPKSPSVDKYNVSGTNGTCLLASMGLQL

NLTYERKDNTTVTRLLNINPNKTSASGSCGAHLVTLELHSEGTTVLLFQFGMNASSSRFF

LQGIQLNTILPDARDPAFKAANGSLRALQATVGNSYKCNAEEHVRVTKAFSVNIFKVWVQ

AFKVEGGQFGSVEECLLDENSMLIPIAVGGALAGLVLIVLIAYLVGRKRSHAGYQTI

(4^th^,5^th^and11^th^NinD.R)

409.>sp|P15151|PVR_HUMANPoliovirusreceptorOS=HomosapiensGN=PVRPE=1SV=2

MARAMAAAWPLLLVALLVLSWPPPGTGDVVVQAPTQVPGFLGDSVTLPCYLQVPNMEVTH

VSQLTWARHGESGSMAVFHQTQGPSYSESKRLEFVAARLGAELRNASLRMFGLRVEDEGN

YTCLFVTFPQGSRSVDIWLRVLAKPQNTAEVQKVQLTGEPVPMARCVSTGGRPPAQITWH

SDLGGMPNTSQVPGFLSGTVTVTSLWILVPSSQVDGKNVTCKVEHESFEKPQLLTVNLTV

YYPPEVSISGYDNNWYLGQNEATLTCDARSNPEPTGYNWSTTMGPLPPFAVAQGAQLLIR

PVDKPINTTLICNVTNALGARQAELTVQVKEGPPSEHSGISRNAIIFLVLGILVFLILLG

IGIYFYWSKCSREVLWHCHLCPSSTEHASASANGHVSYSAVSRENSSSQDPQTEGTR

410.>sp|P01009|A1AT_HUMANAlpha-1-antitrypsinOS=HomosapiensGN=SERPINA1PE=1SV=3

MPSSVSWGILLLAGLCCLVPVSLAEDPQGDAAQKTDTSHHDQDHPTFNKITPNLAEFAFS

LYRQLAHQSNSTNIFFSPVSIATAFAMLSLGTKADTHDEILEGLNFNLTEIPEAQIHEGF

QELLRTLNQPDSQLQLTTGNGLFLSEGLKLVDKFLEDVKKLYHSEAFTVNFGDTEEAKKQ

INDYVEKGTQGKIVDLVKELDRDTVFALVNYIFFKGKWERPFEVKDTEEEDFHVDQVTTV

KVPMMKRLGMFNIQHCKKLSSWVLLMKYLGNATAIFFLPDEGKLQHLENELTHDIITKFL

ENEDRRSASLHLPKLSITGTYDLKSVLGQLGITKVFSNGADLSGVTEEAPLKLSKAVHKA

VLTIDEKGTEAAGAMFLEAIPMSIPPEVKFNKPFVFLMIEQNTKSPLFMGKVVNPTQK

411.>sp|P36955|PEDF_HUMANPigmentepithelium-derivedfactorOS=HomosapiensGN=SERPINF1PE=1SV=4

MQALVLLLCIGALLGHSSCQNPASPPEEGSPDPDSTGALVEEEDPFFKVPVNKLAAAVSN

FGYDLYRVRSSTSPTTNVLLSPLSVATALSALSLGAEQRTESIIHRALYYDLISSPDIHG

TYKELLDTVTAPQKNLKSASRIVFEKKLRIKSSFVAPLEKSYGTRPRVLTGNPRLDLQEI

NNWVQAQMKGKLARSTKEIPDEISILLLGVAHFKGQWVTKFDSRKTSLEDFYLDEERTVR

VPMMSDPKAVLRYGLDSDLSCKIAQLPLTGSMSIIFFLPLKVTQNLTLIEESLTSEFIHD

IDRELKTVQAVLTVPKLKLSYEGEVTKSLQEMKLQSLFDSPDFSKITGKPIKLTQVEHRA

GFEWNEDGAGTTPSPGLQPAHLTFPLDYHLNQPFIFVLRDTDTGALLFIGKILDPRGP

412.>sp|Q86XS8|GOLI_HUMANE3ubiquitin-proteinligaseRNF130OS=HomosapiensGN=RNF130PE=1SV=1

MSCAGRAGPARLAALALLTCSLWPARADNASQEYYTALINVTVQEPGRGAPLTFRIDRGR

YGLDSPKAEVRGQVLAPLPLHGVADHLGCDPQTRFFVPPNIKQWIALLQRGNCTFKEKIS

RAAFHNAVAVVIYNNKSKEEPVTMTHPGTGDIIAVMITELRGKDILSYLEKNISVQMTIA

VGTRMPPKNFSRGSLVFVSISFIVLMIISSAWLIFYFIQKIRYTNARDRNQRRLGDAAKK

AISKLTTRTVKKGDKETDPDFDHCAVCIESYKQNDVVRILPCKHVFHKSCVDPWLSEHCT

CPMCKLNILKALGIVPNLPCTDNVAFDMERLTRTQAVNRRSALGDLAGDNSLGLEPLRTS

GISPLPQDGELTPRTGEINIAVTKEWFIIASFGLLSALTLCYMIIRATASLNANEVEWF

413.>sp|P49767|VEGFC_HUMANVascularendothelialgrowthfactorCOS=HomosapiensGN=VEGFCPE=1SV=1

MHLLGFFSVACSLLAAALLPGPREAPAAAAAFESGLDLSDAEPDAGEATAYASKDLEEQL

RSVSSVDELMTVLYPEYWKMYKCQLRKGGWQHNREQANLNSRTEETIKFAAAHYNTEILK

SIDNEWRKTQCMPREVCIDVGKEFGVATNTFFKPPCVSVYRCGGCCNSEGLQCMNTSTSY

LSKTLFEITVPLSQGPKPVTISFANHTSCRCMSKLDVYRQVHSIIRRSLPATLPQCQAAN

KTCPTNYMWNNHICRCLAQEDFMFSSDAGDDSTDGFHDICGPNKELDEETCQCVCRAGLR

PASCGPHKELDRNSCQCVCKNKLFPSQCGANREFDENTCQCVCKRTCPRNQPLNPGKCAC

ECTESPQKCLLKGKKFHHQTCSCYRRPCTNRQKACEPGFSYSEEVCRCVPSYWKRPQMS

414.>sp|Q13641|TPBG_HUMANTrophoblastglycoproteinOS=HomosapiensGN=TPBGPE=1SV=1

MPGGCSRGPAAGDGRLRLARLALVLLGWVSSSSPTSSASSFSSSAPFLASAVSAQPPLPD

QCPALCECSEAARTVKCVNRNLTEVPTDLPAYVRNLFLTGNQLAVLPAGAFARRPPLAEL

AALNLSGSRLDEVRAGAFEHLPSLRQLDLSHNPLADLSPFAFSGSNASVSAPSPLVELIL

NHIVPPEDERQNRSFEGMVVAALLAGRALQGLRRLELASNHFLYLPRDVLAQLPSLRHLD

LSNNSLVSLTYVSFRNLTHLESLHLEDNALKVLHNGTLAELQGLPHIRVFLDNNPWVCDC

HMADMVTWLKETEVVQGKDRLTCAYPEKMRNRVLLELNSADLDCDPILPPSLQTSYVFLG

IVLALIGAIFLLVLYLNRKGIKKWMHNIRDACRDHMEGYHYRYEINADPRLTNLSSNSDV

415.>sp|Q9UI42|CBPA4_HUMANCarboxypeptidaseA4OS=HomosapiensGN=CPA4PE=1SV=2

MRWILFIGALIGSSICGQEKFFGDQVLRINVRNGDEISKLSQLVNSNNLKLNFWKSPSSF

NRPVDVLVPSVSLQAFKSFLRSQGLEYAVTIEDLQALLDNEDDEMQHNEGQERSSNNFNY

GAYHSLEAIYHEMDNIAADFPDLARRVKIGHSFENRPMYVLKFSTGKGVRRPAVWLNAGI

HSREWISQATAIWTARKIVSDYQRDPAITSILEKMDIFLLPVANPDGYVYTQTQNRLWRK

TRSRNPGSSCIGADPNRNWNASFAGKGASDNPCSEVYHGPHANSEVEVKSVVDFIQKHGN

FKGFIDLHSYSQLLMYPYGYSVKKAPDAEELDKVARLAAKALASVSGTEYQVGPTCTTVY

PASGSSIDWAYDNGIKFAFTFELRDTGTYGFLLPANQIIPTAEETWLGLKTIMEHVRDNL

Y

416.>sp|P01011|AACT_HUMANAlpha-1-antichymotrypsinOS=HomosapiensGN=SERPINA3PE=1SV=2

MERMLPLLALGLLAAGFCPAVLCHPNSPLDEENLTQENQDRGTHVDLGLASANVDFAFSL

YKQLVLKAPDKNVIFSPLSISTALAFLSLGAHNTTLTEILKGLKFNLTETSEAEIHQSFQ

HLLRTLNQSSDELQLSMGNAMFVKEQLSLLDRFTEDAKRLYGSEAFATDFQDSAAAKKLI

NDYVKNGTRGKITDLIKDLDSQTMMVLVNYIFFKAKWEMPFDPQDTHQSRFYLSKKKWVM

VPMMSLHHLTIPYFRDEELSCTVVELKYTGNASALFILPDQDKMEEVEAMLLPETLKRWR

DSLEFREIGELYLPKFSISRDYNLNDILLQLGIEEAFTSKADLSGITGARNLAVSQVVHK

AVLDVFEEGTEASAATAVKITLLSALVETRTIVRFNRPFLMIIVPTDTQNIFFMSKVTNP

KQA

417.>sp|Q96IY4|CBPB2_HUMANCarboxypeptidaseB2OS=HomosapiensGN=CPB2PE=1SV=2

MKLCSLAVLVPIVLFCEQHVFAFQSGQVLAALPRTSRQVQVLQNLTTTYEIVLWQPVTAD

LIVKKKQVHFFVNASDVDNVKAHLNVSGIPCSVLLADVEDLIQQQISNDTVSPRASASYY

EQYHSLNEIYSWIEFITERHPDMLTKIHIGSSFEKYPLYVLKVSGKEQAAKNAIWIDCGI

HAREWISPAFCLWFIGHITQFYGIIGQYTNLLRLVDFYVMPVVNVDGYDYSWKKNRMWRK

NRSFYANNHCIGTDLNRNFASKHWCEEGASSSSCSETYCGLYPESEPEVKAVASFLRRNI

NQIKAYISMHSYSQHIVFPYSYTRSKSKDHEELSLVASEAVRAIEKISKNTRYTHGHGSE

TLYLAPGGGDDWIYDLGIKYSFTIELRDTGTYGFLLPERYIKPTCREAFAAVSKIAWHVI

RNV

418.>sp|P11117|PPAL_HUMANLysosomalacidphosphataseOS=HomosapiensGN=ACP2PE=1SV=3

MAGKRSGWSRAALLQLLLGVNLVVMPPTRARSLRFVTLLYRHGDRSPVKTYPKDPYQEEE

WPQGFGQLTKEGMLQHWELGQALRQRYHGFLNTSYHRQEVYVRSTDFDRTLMSAEANLAG

LFPPNGMQRFNPNISWQPIPVHTVPITEDRLLKFPLGPCPRYEQLQNETRQTPEYQNESS

RNAQFLDMVANETGLTDLTLETVWNVYDTLFCEQTHGLRLPPWASPQTMQRLSRLKDFSF

RFLFGIYQQAEKARLQGGVLLAQIRKNLTLMATTSQLPKLLVYSAHDTTLVALQMALDVY

NGEQAPYASCHIFELYQEDSGNFSVEMYFRNESDKAPWPLSLPGCPHRCPLQDFLRLTEP

VVPKDWQQECQLASGPADTEVIVALAVCGSILFLLIVLLLTVLFRMQAQPPGYRHVADGE

DHA

419.>sp|P21754|ZP3_HUMANZonapellucidasperm-bindingprotein3OS=HomosapiensGN=ZP3PE=1SV=2

MELSYRLFICLLLWGSTELCYPQPLWLLQGGASHPETSVQPVLVECQEATLMVMVSKDLF

GTGKLIRAADLTLGPEACEPLVSMDTEDVVRFEVGLHECGNSMQVTDDALVYSTFLLHDP

RPVGNLSIVRTNRAEIPIECRYPRQGNVSSQAILPTWLPFRTTVFSEEKLTFSLRLMEEN

WNAEKRSPTFHLGDAAHLQAEIHTGSHVPLRLFVDHCVATPTPDQNASPYHTIVDFHGCL

VDGLTDASSAFKVPRPGPDTLQFTVDVFHFANDSRNMIYITCHLKVTLAEQDPDELNKAC

SFSKPSNSWFPVEGSADICQCCNKGDCGTPSHSRRQPHVMSQWSRSASRNRRHVTEEADV

TVGPLIFLDRRGDHEVEQWALPSDTSVVLLGVGLAVVVSLTLTAVILVLTRRCRTASHPV

SASE

420.>sp|Q96RD7|PANX1_HUMANPannexin-1OS=HomosapiensGN=PANX1PE=1SV=4

MAIAQLATEYVFSDFLLKEPTEPKFKGLRLELAVDKMVTCIAVGLPLLLISLAFAQEISI

GTQISCFSPSSFSWRQAAFVDSYCWAAVQQKNSLQSESGNLPLWLHKFFPYILLLFAILL

YLPPLFWRFAAAPHICSDLKFIMEELDKVYNRAIKAAKSARDLDMRDGACSVPGVTENLG

QSLWEVSESHFKYPIVEQYLKTKKNSNNLIIKYISCRLLTLIIILLACIYLGYYFSLSSL

SDEFVCSIKSGILRNDSTVPDQFQCKLIAVGIFQLLSVINLVVYVLLAPVVVYTLFVPFR

QKTDVLKVYEILPTFDVLHFKSEGYNDLSLYNLFLEENISEVKSYKCLKVLENIKSSGQG

IDPMLLLTNLGMIKMDVVDGKTPMSAEMREEQGNQTAELQGMNIDSETKANNGEKNARQR

LLDSSC

421.>sp|P29622|KAIN_HUMANKallistatinOS=HomosapiensGN=SERPINA4PE=1SV=3

MHLIDYLLLLLVGLLALSHGQLHVEHDGESCSNSSHQQILETGEGSPSLKIAPANADFAF

RFYYLIASETPGKNIFFSPLSISAAYAMLSLGACSHSRSQILEGLGFNLTELSESDVHRG

FQHLLHTLNLPGHGLETRVGSALFLSHNLKFLAKFLNDTMAVYEAKLFHTNFYDTVGTIQ

LINDHVKKETRGKIVDLVSELKKDVLMVLVNYIYFKALWEKPFISSRTTPKDFYVDENTT

VRVPMMLQDQEHHWYLHDRYLPCSVLRMDYKGDATVFFILPNQGKMREIEEVLTPEMLMR

WNNLLRKRNFYKKLELHLPKFSISGSYVLDQILPRLGFTDLFSKWADLSGITKQQKLEAS

KSFHKATLDVDEAGTEAAAATSFAIKFFSAQTNRHILRFNRPFLVVIFSTSTQSVLFLGK

VVDPTKP

422.>sp|O75356|ENTP5_HUMANEctonucleosidetriphosphatediphosphohydrolase5OS=HomosapiensGN=ENTPD5PE=1SV=1

MATSWGTVFFMLVVSCVCSAVSHRNQQTWFEGIFLSSMCPINVSASTLYGIMFDAGSTGT

RIHVYTFVQKMPGQLPILEGEVFDSVKPGLSAFVDQPKQGAETVQGLLEVAKDSIPRSHW

KKTPVVLKATAGLRLLPEHKAKALLFEVKEIFRKSPFLVPKGSVSIMDGSDEGILAWVTV

NFLTGQLHGHRQETVGTLDLGGASTQITFLPQFEKTLEQTPRGYLTSFEMFNSTYKLYTH

SYLGFGLKAARLATLGALETEGTDGHTFRSACLPRWLEAEWIFGGVKYQYGGNQEGEVGF

EPCYAEVLRVVRGKLHQPEEVQRGSFYAFSYYYDRAVDTDMIDYEKGGILKVEDFERKAR

EVCDNLENFTSGSPFLCMDLSYITALLKDGFGFADSTVLQLTKKVNNIETGWALGATFHL

LQSLGISH

423.>sp|P01854|IGHE_HUMANIgepsilonchainCregionOS=HomosapiensGN=IGHEPE=1SV=1

ASTQSPSVFPLTRCCKNIPSNATSVTLGCLATGYFPEPVMVTWDTGSLNGTTMTLPATTL

TLSGHYATISLLTVSGAWAKQMFTCRVAHTPSSTDWVDNKTFSVCSRDFTPPTVKILQSS

CDGGGHFPPTIQLLCLVSGYTPGTINITWLEDGQVMDVDLSTASTTQEGELASTQSELTL

SQKHWLSDRTYTCQVTYQGHTFEDSTKKCADSNPRGVSAYLSRPSPFDLFIRKSPTITCL

VVDLAPSKGTVNLTWSRASGKPVNHSTRKEEKQRNGTLTVTSTLPVGTRDWIEGETYQCR

VTHPHLPRALMRSTTKTSGPRAAPEVYAFATPEWPGSRDKRTLACLIQNFMPEDISVQWL

HNEVQLPDARHSTTQPRKTKGSGFFVFSRLEVTRAEWEQKDEFICRAVHEAASPSQTVQR

AVSVNPGK

(1^st^and5^th^NinD.R)

424.>sp|Q8TEB7|RN128_HUMANE3ubiquitin-proteinligaseRNF128OS=HomosapiensGN=RNF128PE=1SV=1

MGPPPGAGVSCRGGCGFSRLLAWCFLLALSPQAPGSRGAEAVWTAYLNVSWRVPHTGVNR

TVWELSEEGVYGQDSPLEPVAGVLVPPDGPGALNACNPHTNFTVPTVWGSTVQVSWLALI

QRGGGCTFADKIHLAYERGASGAVIFNFPGTRNEVIPMSHPGAVDIVAIMIGNLKGTKIL

QSIQRGIQVTMVIEVGKKHGPWVNHYSIFFVSVSFFIITAATVGYFIFYSARRLRNARAQ

SRKQRQLKADAKKAIGRLQLRTLKQGDKEIGPDGDSCAVCIELYKPNDLVRILTCNHIFH

KTCVDPWLLEHRTCPMCKCDILKALGIEVDVEDGSVSLQVPVSNEISNSASSHEEDNRSE

TASSGYASVQGTDEPPLEEHVQSTNESLQLVNHEANSVAVDVIPHVDNPTFEEDETPNQE

TAVREIKS

425.>sp|P06280|AGAL_HUMANAlpha-galactosidaseAOS=HomosapiensGN=GLAPE=1SV=1

MQLRNPELHLGCALALRFLALVSWDIPGARALDNGLARTPTMGWLHWERFMCNLDCQEEP

DSCISEKLFMEMAELMVSEGWKDAGYEYLCIDDCWMAPQRDSEGRLQADPQRFPHGIRQL

ANYVHSKGLKLGIYADVGNKTCAGFPGSFGYYDIDAQTFADWGVDLLKFDGCYCDSLENL

ADGYKHMSLALNRTGRSIVYSCEWPLYMWPFQKPNYTEIRQYCNHWRNFADIDDSWKSIK

SILDWTSFNQERIVDVAGPGGWNDPDMLVIGNFGLSWNQQVTQMALWAIMAAPLFMSNDL

RHISPQAKALLQDKDVIAINQDPLGKQGYQLRQGDNFEVWERPLSGLAWAVAMINRQEIG

GPRSYTIAVASLGKGVACNPACFITQLLPVKRKLGFYEWTSRLRSHINPTGTVLLQLENT

MQMSLKDLL

426.>sp|Q6WN34|CRDL2_HUMANChordin-likeprotein2OS=HomosapiensGN=CHRDL2PE=1SV=1

MVPEVRVLSSLLGLALLWFPLDSHARARPDMFCLFHGKRYSPGESWHPYLEPQGLMYCLR

CTCSEGAHVSCYRLHCPPVHCPQPVTEPQQCCPKCVEPHTPSGLRAPPKSCQHNGTMYQH

GEIFSAHELFPSRLPNQCVLCSCTEGQIYCGLTTCPEPGCPAPLPLPDSCCQACKDEASE

QSDEEDSVQSLHGVRHPQDPCSSDAGRKRGPGTPAPTGLSAPLSFIPRHFRPKGAGSTTV

KIVLKEKHKKACVHGGKTYSHGEVWHPAFRAFGPLPCILCTCEDGRQDCQRVTCPTEYPC

RHPEKVAGKCCKICPEDKADPGHSEISSTRCPKAPGRVLVHTSVSPSPDNLRRFALEHEA

SDLVEIYLWKLVKGIFHLTQIKKVRKQDFQKEAQHFRLLAGPHEGHWNVFLAQTLELKVT

ASPDKVTKT

(NinD.R)

427.>sp|Q9Y2G5|OFUT2_HUMANGDP-fucoseproteinO-fucosyltransferase2OS=HomosapiensGN=POFUT2PE=1SV=3

MATLSFVFLLLGAVSWPPASASGQEFWPGQSAADILSGAASRRRYLLYDVNPPEGFNLRR

DVYIRIASLLKTLLKTEEWVLVLPPWGRLYHWQSPDIHQVRIPWSEFFDLPSLNKNIPVI

EYEQFIAESGGPFIDQVYVLQSYAEGWKEGTWEEKVDERPCIDQLLYSQDKHEYYRGWFW

GYEETRGLNVSCLSVQGSASIVAPLLLRNTSARSVMLDRAENLLHDHYGGKEYWDTRRSM

VFARHLREVGDEFRSRHLNSTDDADRIPFQEDWMKMKVKLGSALGGPYLGVHLRRKDFIW

GHRQDVPSLEGAVRKIRSLMKTHRLDKVFVATDAVRKEYEELKKLLPEMVRFEPTWEELE

LYKDGGVAIIDQWICAHARFFIGTSVSTFSFRIHEEREILGLDPKTTYNRFCGDQEKACE

QPTHWKITY

428.>sp|Q8WWF5|ZNRF4_HUMANE3ubiquitin-proteinligaseZNRF4OS=HomosapiensGN=ZNRF4PE=1SV=3

MPLCRPEHLMPRASRVPVAASLPLSHAVIPTQLPSRPGHRPPGRPRRCPKASCLPPPVGP

SSTQTAKRVTMGWPRPGRALVAVKALLVLSLLQVPAQAVVRAVLEDNSSSVDFADLPALF

GVPLAPEGIRGYLMEVKPANACHPIEAPRLGNRSLGAIVLIRRYDCTFDLKVLNAQRAGF

EAAIVHNVHSDDLVSMTHVYEDLRGQIAIPSVFVSEAASQDLRVILGCNKSAHALLLPDD

PPCHDLGCHPVLTVSWVLGCTLALVVSAFFVLNHLWLWAQACCSHRRPVKTSTCQKAQVR

TFTWHNDLCAICLDEYEEGDQLKILPCSHTYHCKCIDPWFSQAPRRSCPVCKQSVAATED

SFDSTTYSFRDEDPSLPGHRPPIWAIQVQLRSRRLELLGRASPHCHCSTTSLEAEYTTVS

SAPPEAPGQ

429.>sp|P00749|UROK_HUMANUrokinase-typeplasminogenactivatorOS=HomosapiensGN=PLAUPE=1SV=2

MRALLARLLLCVLVVSDSKGSNELHQVPSNCDCLNGGTCVSNKYFSNIHWCNCPKKFGGQ

HCEIDKSKTCYEGNGHFYRGKASTDTMGRPCLPWNSATVLQQTYHAHRSDALQLGLGKHN

YCRNPDNRRRPWCYVQVGLKPLVQECMVHDCADGKKPSSPPEELKFQCGQKTLRPRFKII

GGEFTTIENQPWFAAIYRRHRGGSVTYVCGGSLISPCWVISATHCFIDYPKKEDYIVYLG

RSRLNSNTQGEMKFEVENLILHKDYSADTLAHHNDIALLKIRSKEGRCAQPSRTIQTICL

PSMYNDPQFGTSCEITGFGKENSTDYLYPEQLKMTVVKLISHRECQQPHYYGSEVTTKML

CAADPQWKTDSCQGDSGGPLVCSLQGRMTLTGIVSWGRGCALKDKPGVYTRVSHFLPWIR

SHTKEENGLAL

430.>sp|Q99538|LGMN_HUMANLegumainOS=HomosapiensGN=LGMNPE=1SV=1

MVWKVAVFLSVALGIGAVPIDDPEDGGKHWVVIVAGSNGWYNYRHQADACHAYQIIHRNG

IPDEQIVVMMYDDIAYSEDNPTPGIVINRPNGTDVYQGVPKDYTGEDVTPQNFLAVLRGD

AEAVKGIGSGKVLKSGPQDHVFIYFTDHGSTGILVFPNEDLHVKDLNETIHYMYKHKMYR

KMVFYIEACESGSMMNHLPDNINVYATTAANPRESSYACYYDEKRSTYLGDWYSVNWMED

SDVEDLTKETLHKQYHLVKSHTNTSHVMQYGNKTISTMKVMQFQGMKRKASSPVPLPPVT

HLDLTPSPDVPLTIMKRKLMNTNDLEESRQLTEEIQRHLDARHLIEKSVRKIVSLLAASE

AEVEQLLSERAPLTGHSCYPEALLHFRTHCFNWHSPTYEYALRHLYVLVNLCEKPYPLHR

IKLSMDHVCLGHY

431.>sp|P20061|TCO1_HUMANTranscobalamin-1OS=HomosapiensGN=TCN1PE=1SV=2

MRQSHQLPLVGLLLFSFIPSQLCEICEVSEENYIRLKPLLNTMIQSNYNRGTSAVNVVLS

LKLVGIQIQTLMQKMIQQIKYNVKSRLSDVSSGELALIILALGVCRNAEENLIYDYHLID

KLENKFQAEIENMEAHNGTPLTNYYQLSLDVLALCLFNGNYSTAEVVNHFTPENKNYYFG

SQFSVDTGAMAVLALTCVKKSLINGQIKADEGSLKNISIYTKSLVEKILSEKKENGLIGN

TFSTGEAMQALFVSSDYYNENDWNCQQTLNTVLTEISQGAFSNPNAAAQVLPALMGKTFL

DINKDSSCVSASGNFNISADEPITVTPPDSQSYISVNYSVRINETYFTNVTVLNGSVFLS

VMEKAQKMNDTIFGFTMEERSWGPYITCIQGLCANNNDRTYWELLSGGEPLSQGAGSYVV

RNGENLEVRWSKY

(3^rd^NinO.R)

432.>sp|Q12794|HYAL1_HUMANHyaluronidase-1OS=HomosapiensGN=HYAL1PE=1SV=2

MAAHLLPICALFLTLLDMAQGFRGPLLPNRPFTTVWNANTQWCLERHGVDVDVSVFDVVA

NPGQTFRGPDMTIFYSSQLGTYPYYTPTGEPVFGGLPQNASLIAHLARTFQDILAAIPAP

DFSGLAVIDWEAWRPRWAFNWDTKDIYRQRSRALVQAQHPDWPAPQVEAVAQDQFQGAAR

AWMAGTLQLGRALRPRGLWGFYGFPDCYNYDFLSPNYTGQCPSGIRAQNDQLGWLWGQSR

ALYPSIYMPAVLEGTGKSQMYVQHRVAEAFRVAVAAGDPNLPVLPYVQIFYDTTNHFLPL

DELEHSLGESAAQGAAGVVLWVSWENTRTKESCQAIKEYMDTTLGPFILNVTSGALLCSQ

ALCSGHGRCVRRTSHPKALLLLNPASFSIQLTPGGGPLSLRGALSLEDQAQMAVEFKCRC

YPGWQAPWCERKSMW

433.>sp|O43556|SGCE_HUMANEpsilon-sarcoglycanOS=HomosapiensGN=SGCEPE=1SV=6

MQLPRWWELGDPCAWTGQGRGTRRMSPATTGTFLLTVYSIFSKVHSDRNVYPSAGVLFVH

VLEREYFKGEFPPYPKPGEISNDPITFNTNLMGYPDRPGWLRYIQRTPYSDGVLYGSPTA

ENVGKPTIIEITAYNRRTFETARHNLIINIMSAEDFPLPYQAEFFIKNMNVEEMLASEVL

GDFLGAVKNVWQPERLNAINITSALDRGGRVPLPINDLKEGVYVMVGADVPFSSCLREVE

NPQNQLRCSQEMEPVITCDKKFRTQFYIDWCKISLVDKTKQVSTYQEVIRGEGILPDGGE

YKPPSDSLKSRDYYTDFLITLAVPSAVALVLFLILAYIMCCRREGVEKRNMQTPDIQLVH

HSAIQKSTKELRDMSKNREIAWPLSTLPVFHPVTGEIIPPLHTDNYDSTNMPLMQTQQNL

PHQTQIPQQQTTGKWYP

434.>sp|Q13286|CLN3_HUMANBatteninOS=HomosapiensGN=CLN3PE=1SV=1

MGGCAGSRRRFSDSEGEETVPEPRLPLLDHQGAHWKNAVGFWLLGLCNNFSYVVMLSAAH

DILSHKRTSGNQSHVDPGPTPIPHNSSSRFDCNSVSTAAVLLADILPTLVIKLLAPLGLH

LLPYSPRVLVSGICAAGSFVLVAFSHSVGTSLCGVVFASISSGLGEVTFLSLTAFYPRAV

ISWWSSGTGGAGLLGALSYLGLTQAGLSPQQTLLSMLGIPALLLASYFLLLTSPEAQDPG

GEEEAESAARQPLIRTEAPESKPGSSSSLSLRERWTVFKGLLWYIVPLVVVYFAEYFINQ

GLFELLFFWNTSLSHAQQYRWYQMLYQAGVFASRSSLRCCRIRFTWALALLQCLNLVFLL

ADVWFGFLPSIYLVFLIILYEGLLGGAAYVNTFHNIALETSDEHREFAMAATCISDTLGI

SLSGLLALPLHDFLCQLS

435.>sp|Q14314|FGL2_HUMANFibroleukinOS=HomosapiensGN=FGL2PE=1SV=1

MKLANWYWLSSAVLATYGFLVVANNETEEIKDERAKDVCPVRLESRGKCEEAGECPYQVS

LPPLTIQLPKQFSRIEEVFKEVQNLKEIVNSLKKSCQDCKLQADDNGDPGRNGLLLPSTG

APGEVGDNRVRELESEVNKLSSELKNAKEEINVLHGRLEKLNLVNMNNIENYVDSKVANL

TFVVNSLDGKCSKCPSQEQIQSRPVQHLIYKDCSDYYAIGKRSSETYRVTPDPKNSSFEV

YCDMETMGGGWTVLQARLDGSTNFTRTWQDYKAGFGNLRREFWLGNDKIHLLTKSKEMIL

RIDLEDFNGVELYALYDQFYVANEFLKYRLHVGNYNGTAGDALRFNKHYNHDLKFFTTPD

KDNDRYPSGNCGLYYSSGWWFDACLSANLNGKYYHQKYRGVRNGIFWGTWPGVSEAHPGG

YKSSFKEAKMMIRPKHFKP

(1^st^NinD.R)

436.>sp|Q8N6C8|LIRA3_HUMANLeukocyteimmunoglobulin-likereceptorsubfamilyAmember3OS=HomosapiensGN=LILRA3PE=1SV=3

MTPILTVLICLGLSLDPRTHVQAGPLPKPTLWAEPGSVITQGSPVTLRCQGSLETQEYHL

YREKKTALWITRIPQELVKKGQFPILSITWEHAGRYCCIYGSHTAGLSESSDPLELVVTG

AYSKPTLSALPSPVVTSGGNVTIQCDSQVAFDGFILCKEGEDEHPQCLNSHSHARGSSRA

IFSVGPVSPSRRWSYRCYGYDSRAPYVWSLPSDLLGLLVPGVSKKPSLSVQPGPVVAPGE

KLTFQCGSDAGYDRFVLYKEWGRDFLQRPGRQPQAGLSQANFTLGPVSRSYGGQYTCSGA

YNLSSEWSAPSDPLDILITGQIRARPFLSVRPGPTVASGENVTLLCQSQGGMHTFLLTKE

GAADSPLRLKSKRQSHKYQAEFPMSPVTSAHAGTYRCYGSLSSNPYLLTHPSDPLELVVS

GAAETLSPPQNKSDSKAGE

437.>sp|Q4G148|GXLT1_HUMANGlucosidexylosyltransferase1OS=HomosapiensGN=GXYLT1PE=1SV=2

MRRYLRVVVLCVACGFCSLLYAFSQLAVSLEEGTGGGGGKPQAAVASWLAGGGRGAVRGA

GVAGPAAHPGVSDRCKDFSLCYWNPYWMLPSDVCGMNCFWEAAFRYSLKIQPVEKMHLAV

VACGERLEETMTMLKSAIIFSIKPLQFHIFAEDQLHHSFKGRLDNWSFLQTFNYTLYPIT

FPSENAAEWKKLFKPCASQRLFLPLILKEVDSLLYVDTDILFLRPVDDIWSLLKKFNSTQ

IAAMAPEHEEPRIGWYNRFARHPYYGKTGVNSGVMLMNMTRMRRKYFKNDMTTVRLQWGD

ILMPLLKKYKLNITWGDQDLLNIVFFHNPESLFVFPCQWNYRPDHCIYGSNCQEAEEGGI

FILHGNRGVYHDDKQPAFRAVYEALRNCSFEDDNIRSLLKPLELELQKTVHTYCGKIYKI

FIKQLAKSVRDRYARSPKEK

438.>sp|P04180|LCAT_HUMANPhosphatidylcholine-sterolacyltransferaseOS=HomosapiensGN=LCATPE=1SV=1

MGPPGSPWQWVTLLLGLLLPPAAPFWLLNVLFPPHTTPKAELSNHTRPVILVPGCLGNQL

EAKLDKPDVVNWMCYRKTEDFFTIWLDLNMFLPLGVDCWIDNTRVVYNRSSGLVSNAPGV

QIRVPGFGKTYSVEYLDSSKLAGYLHTLVQNLVNNGYVRDETVRAAPYDWRLEPGQQEEY

YRKLAGLVEEMHAAYGKPVFLIGHSLGCLHLLYFLLRQPQAWKDRFIDGFISLGAPWGGS

IKPMLVLASGDNQGIPIMSSIKLKEEQRITTTSPWMFPSRMAWPEDHVFISTPSFNYTGR

DFQRFFADLHFEEGWYMWLQSRDLLAGLPAPGVEVYCLYGVGLPTPRTYIYDHGFPYTDP

VGVLYEDGDDTVATRSTELCGLWQGRQPQPVHLLPLHGIQHLNMVFSNLTLEHINAILLG

AYRQGPPASPTASPEPPPPE

439.>sp|O95264|5HT3B_HUMAN5-hydroxytryptaminereceptor3BOS=HomosapiensGN=HTR3BPE=1SV=1

MLSSVMAPLWACILVAAGILATDTHHPQDSALYHLSKQLLQKYHKEVRPVYNWTKATTVY

LDLFVHAILDVDAENQILKTSVWYQEVWNDEFLSWNSSMFDEIREISLPLSAIWAPDIII

NEFVDIERYPDLPYVYVNSSGTIENYKPIQVVSACSLETYAFPFDVQNCSLTFKSILHTV

EDVDLAFLRSPEDIQHDKKAFLNDSEWELLSVSSTYSILQSSAGGFAQIQFNVVMRRHPL

VYVVSLLIPSIFLMLVDLGSFYLPPNCRARIVFKTSVLVGYTVFRVNMSNQVPRSVGSTP

LIGHFFTICMAFLVLSLAKSIVLVKFLHDEQRGGQEQPFLCLRGDTDADRPRVEPRAQRA

VVTESSLYGEHLAQPGTLKEVWSQLQSISNYLQTQDQTDQQEAEWLVLLSRFDRLLFQSY

LFMLGIYTITLCSLWALWGGV

440.>sp|Q13093|PAFA_HUMANPlatelet-activatingfactoracetylhydrolaseOS=HomosapiensGN=PLA2G7PE=1SV=1

MVPPKLHVLFCLCGCLAVVYPFDWQYINPVAHMKSSAWVNKIQVLMAAASFGQTKIPRGN

GPYSVGCTDLMFDHTNKGTFLRLYYPSQDNDRLDTLWIPNKEYFWGLSKFLGTHWLMGNI

LRLLFGSMTTPANWNSPLRPGEKYPLVVFSHGLGAFRTLYSAIGIDLASHGFIVAAVEHR

DRSASATYYFKDQSAAEIGDKSWLYLRTLKQEEETHIRNEQVRQRAKECSQALSLILDID

HGKPVKNALDLKFDMEQLKDSIDREKIAVIGHSFGGATVIQTLSEDQRFRCGIALDAWMF

PLGDEVYSRIPQPLFFINSEYFQYPANIIKMKKCYSPDKERKMITIRGSVHQNFADFTFA

TGKIIGHMLKLKGDIDSNVAIDLSNKASLAFLQKHLGLHKDFDQWDCLIEGDDENLIPGT

NINTTNQHIMLQNSSGIEKYN

441.>sp|Q9BY67|CADM1_HUMANCelladhesionmolecule1OS=HomosapiensGN=CADM1PE=1SV=2

MASVVLPSGSQCAAAAAAAAPPGLRLRLLLLLFSAAALIPTGDGQNLFTKDVTVIEGEVA

TISCQVNKSDDSVIQLLNPNRQTIYFRDFRPLKDSRFQLLNFSSSELKVSLTNVSISDEG

RYFCQLYTDPPQESYTTITVLVPPRNLMIDIQKDTAVEGEEIEVNCTAMASKPATTIRWF

KGNTELKGKSEVEEWSDMYTVTSQLMLKVHKEDDGVPVICQVEHPAVTGNLQTQRYLEVQ

YKPQVHIQMTYPLQGLTREGDALELTCEAIGKPQPVMVTWVRVDDEMPQHAVLSGPNLFI

NNLNKTDNGTYRCEASNIVGKAHSDYMLYVYDPPTTIPPPTTTTTTTTTTTTTILTIITD

SRAGEEGSIRAVDHAVIGGVVAVVVFAMLCLLIILGRYFARHKGTYFTHEAKGADDAADA

DTAIINAEGGQNNSEEKKEYFI

442.>sp|P14384|CBPM_HUMANCarboxypeptidaseMOS=HomosapiensGN=CPMPE=1SV=2

MDFPCLWLGLLLPLVAALDFNYHRQEGMEAFLKTVAQNYSSVTHLHSIGKSVKGRNLWVL

VVGRFPKEHRIGIPEFKYVANMHGDETVGRELLLHLIDYLVTSDGKDPEITNLINSTRIH

IMPSMNPDGFEAVKKPDCYYSIGRENYNQYDLNRNFPDAFEYNNVSRQPETVAVMKWLKT

ETFVLSANLHGGALVASYPFDNGVQATGALYSRSLTPDDDVFQYLAHTYASRNPNMKKGD

ECKNKMNFPNGVTNGYSWYPLQGGMQDYNYIWAQCFEITLELSCCKYPREEKLPSFWNNN

KASLIEYIKQVHLGVKGQVFDQNGNPLPNVIVEVQDRKHICPYRTNKYGEYYLLLLPGSY

IINVTVPGHDPHITKVIIPEKSQNFSALKKDILLPFQGQLDSIPVSNPSCPMIPLYRNLP

DHSAATKPSLFLFLVSLLHIFFK

(LastNinD.R)

443.>sp|P43629|KI3L1_HUMANKillercellimmunoglobulin-likereceptor3DL1OS=HomosapiensGN=KIR3DL1PE=1SV=1

MSLMVVSMACVGLFLVQRAGPHMGGQDKPFLSAWPSAVVPRGGHVTLRCHYRHRFNNFML

YKEDRIHIPIFHGRIFQESFNMSPVTTAHAGNYTCRGSHPHSPTGWSAPSNPVVIMVTGN

HRKPSLLAHPGPLVKSGERVILQCWSDIMFEHFFLHKEGISKDPSRLVGQIHDGVSKANF

SIGPMMLALAGTYRCYGSVTHTPYQLSAPSDPLDIVVTGPYEKPSLSAQPGPKVQAGESV

TLSCSSRSSYDMYHLSREGGAHERRLPAVRKVNRTFQADFPLGPATHGGTYRCFGSFRHS

PYEWSDPSDPLLVSVTGNPSSSWPSPTEPSSKSGNPRHLHILIGTSVVIILFILLLFFLL

HLWCSNKKNAAVMDQEPAGNRTANSEDSDEQDPEEVTYAQLDHCVFTQRKITRPSQRPKT

PPTDTILYTELPNAKPRSKVVSCP

444.>sp|Q9UK55|ZPI_HUMANProteinZ-dependentproteaseinhibitorOS=HomosapiensGN=SERPINA10PE=1SV=1

MKVVPSLLLSVLLAQVWLVPGLAPSPQSPETPAPQNQTSRVVQAPKEEEEDEQEASEEKA

SEEEKAWLMASRQQLAKETSNFGFSLLRKISMRHDGNMVFSPFGMSLAMTGLMLGATGPT

ETQIKRGLHLQALKPTKPGLLPSLFKGLRETLSRNLELGLTQGSFAFIHKDFDVKETFFN

LSKRYFDTECVPMNFRNASQAKRLMNHYINKETRGKIPKLFDEINPETKLILVDYILFKG

KWLTPFDPVFTEVDTFHLDKYKTIKVPMMYGAGKFASTFDKNFRCHVLKLPYQGNATMLV

VLMEKMGDHLALEDYLTTDLVETWLRNMKTRNMEVFFPKFKLDQKYEMHELLRQMGIRRI

FSPFADLSELSATGRNLQVSRVLQRTVIEVDERGTEAVAGILSEITAYSMPPVIKVDRPF

HFMIYEETSGMLLFLGRVVNPTLL

445.>sp|O15533|TPSN_HUMANTapasinOS=HomosapiensGN=TAPBPPE=1SV=1

MKSLSLLLAVALGLATAVSAGPAVIECWFVEDASGKGLAKRPGALLLRQGPGEPPPRPDL

DPELYLSVHDPAGALQAAFRRYPRGAPAPHCEMSRFVPLPASAKWASGLTPAQNCPRALD

GAWLMVSISSPVLSLSSLLRPQPEPQQEPVLITMATVVLTVLTHTPAPRVRLGQDALLDL

SFAYMPPTSEAASSLAPGPPPFGLEWRRQHLGKGHLLLAATPGLNGQMPAAQEGAVAFAA

WDDDEPWGPWTGNGTFWLPRVQPFQEGTYLATIHLPYLQGQVTLELAVYKPPKVSLMPAT

LARAAPGEAPPELLCLVSHFYPSGGLEVEWELRGGPGGRSQKAEGQRWLSALRHHSDGSV

SLSGHLQPPPVTTEQHGARYACRIHHPSLPASGRSAEVTLEVAGLSGPSLEDSVGLFLSA

FLLLGLFKALGWAAVYLSTCKDSKKKAE

446.>sp|P10909|CLUS_HUMANClusterinOS=HomosapiensGN=CLUPE=1SV=1

MMKTLLLFVGLLLTWESGQVLGDQTVSDNELQEMSNQGSKYVNKEIQNAVNGVKQIKTLI

EKTNEERKTLLSNLEEAKKKKEDALNETRESETKLKELPGVCNETMMALWEECKPCLKQT

CMKFYARVCRSGSGLVGRQLEEFLNQSSPFYFWMNGDRIDSLLENDRQQTHMLDVMQDHF

SRASSIIDELFQDRFFTREPQDTYHYLPFSLPHRRPHFFFPKSRIVRSLMPFSPYEPLNF

HAMFQPFLEMIHEAQQAMDIHFHSPAFQHPPTEFIREGDDDRTVCREIRHNSTGCLRMKD

QCDKCREILSVDCSTNNPSQAKLRRELDESLQVAERLTRKYNELLKSYQWKMLNTSSLLE

QLNEQFNWVSRLANLTQGEDQYYLRVTTVASHTSDSDVPSGVTEVVVKLFDSDPITVTVP

VEVSRKNPKFMETVAEKALQEYRKKHREE

447.>sp|P21757|MSRE_HUMANMacrophagescavengerreceptortypesIandIIOS=HomosapiensGN=MSR1PE=1SV=1

MEQWDHFHNQQEDTDSCSESVKFDARSMTALLPPNPKNSPSLQEKLKSFKAALIALYLLV

FAVLIPLIGIVAAQLLKWETKNCSVSSTNANDITQSLTGKGNDSEEEMRFQEVFMEHMSN

MEKRIQHILDMEANLMDTEHFQNFSMTTDQRFNDILLQLSTLFSSVQGHGNAIDEISKSL

ISLNTTLLDLQLNIENLNGKIQENTFKQQEEISKLEERVYNVSAEIMAMKEEQVHLEQEI

KGEVKVLNNITNDLRLKDWEHSQTLRNITLIQGPPGPPGEKGDRGPTGESGPRGFPGPIG

PPGLKGDRGAIGFPGSRGLPGYAGRPGNSGPKGQKGEKGSGNTLTPFTKVRLVGGSGPHE

GRVEILHSGQWGTICDDRWEVRVGQVVCRSLGYPGVQAVHKAAHFGQGTGPIWLNEVFCF

GRESSIEECKIRQWGTRACSHSEDAGVTCTL

448.>sp|P01871|IGHM_HUMANIgmuchainCregionOS=HomosapiensGN=IGHMPE=1SV=3

GSASAPTLFPLVSCENSPSDTSSVAVGCLAQDFLPDSITLSWKYKNNSDISSTRGFPSVL

RGGKYAATSQVLLPSKDVMQGTDEHVVCKVQHPNGNKEKNVPLPVIAELPPKVSVFVPPR

DGFFGNPRKSKLICQATGFSPRQIQVSWLREGKQVGSGVTTDQVQAEAKESGPTTYKVTS

TLTIKESDWLGQSMFTCRVDHRGLTFQQNASSMCVPDQDTAIRVFAIPPSFASIFLTKST

KLTCLVTDLTTYDSVTISWTRQNGEAVKTHTNISESHPNATFSAVGEASICEDDWNSGER

FTCTVTHTDLPSPLKQTISRPKGVALHRPDVYLLPPAREQLNLRESATITCLVTGFSPAD

VFVQWMQRGQPLSPEKYVTSAPMPEPQAPGRYFAHSILTVSEEEWNTGETYTCVAHEALP

NRVTERTVDKSTGKPTLYNVSLVMSDTAGTCY

449.>sp|Q9HB40|RISC_HUMANRetinoid-inducibleserinecarboxypeptidaseOS=HomosapiensGN=SCPEP1PE=1SV=1

MELALRRSPVPRWLLLLPLLLGLNAGAVIDWPTEEGKEVWDYVTVRKDAYMFWWLYYATN

SCKNFSELPLVMWLQGGPGGSSTGFGNFEEIGPLDSDLKPRKTTWLQAASLLFVDNPVGT

GFSYVNGSGAYAKDLAMVASDMMVLLKTFFSCHKEFQTVPFYIFSESYGGKMAAGIGLEL

YKAIQRGTIKCNFAGVALGDSWISPVDSVLSWGPYLYSMSLLEDKGLAEVSKVAEQVLNA

VNKGLYREATELWGKAEMIIEQNTDGVNFYNILTKSTPTSTMESSLEFTQSHLVCLCQRH

VRHLQRDALSQLMNGPIRKKLKIIPEDQSWGGQATNVFVNMEEDFMKPVISIVDELLEAG

INVTVYNGQLDLIVDTMGQEAWVRKLKWPELPKFSQLKWKALYSDPKSLETSAFVKSYKN

LAFYWILKAGHMVPSDQGDMALKMMRLVTQQE

450.>sp|Q92484|ASM3A_HUMANAcidsphingomyelinase-likephosphodiesterase3aOS=HomosapiensGN=SMPDL3APE=1SV=2

MALVRALVCCLLTAWHCRSGLGLPVAPAGGRNPPPAIGQFWHVTDLHLDPTYHITDDHTK

VCASSKGANASNPGPFGDVLCDSPYQLILSAFDFIKNSGQEASFMIWTGDSPPHVPVPEL

STDTVINVITNMTTTIQSLFPNLQVFPALGNHDYWPQDQLPVVTSKVYNAVANLWKPWLD

EEAISTLRKGGFYSQKVTTNPNLRIISLNTNLYYGPNIMTLNKTDPANQFEWLESTLNNS

QQNKEKVYIIAHVPVGYLPSSQNITAMREYYNEKLIDIFQKYSDVIAGQFYGHTHRDSIM

VLSDKKGSPVNSLFVAPAVTPVKSVLEKQTNNPGIRLFQYDPRDYKLLDMLQYYLNLTEA

NLKGESIWKLEYILTQTYDIEDLQPESLYGLAKQFTILDSKQFIKYYNYFFVSYDSSVTC

DKTCKAFQICAIMNLDNISYADCLKQLYIKHNY

451.>sp|Q9Y6X5|ENPP4_HUMANBis(5'-adenosyl)-triphosphataseENPP4OS=HomosapiensGN=ENPP4PE=1SV=3

MKLLVILLFSGLITGFRSDSSSSLPPKLLLVSFDGFRADYLKNYEFPHLQNFIKEGVLVE

HVKNVFITKTFPNHYSIVTGLYEESHGIVANSMYDAVTKKHFSDSNDKDPFWWNEAVPIW

VTNQLQENRSSAAAMWPGTDVPIHDTISSYFMNYNSSVSFEERLNNITMWLNNSNPPVTF

ATLYWEEPDASGHKYGPEDKENMSRVLKKIDDLIGDLVQRLKMLGLWENLNVIITSDHGM

TQCSQDRLINLDSCIDHSYYTLIDLSPVAAILPKINRTEVYNKLKNCSPHMNVYLKEDIP

NRFYYQHNDRIQPIILVADEGWTIVLNESSQKLGDHGYDNSLPSMHPFLAAHGPAFHKGY

KHSTINIVDIYPMMCHILGLKPHPNNGTFGHTKCLLVDQWCINLPEAIAIVIGSLLVLTM

LTCLIIIMQNRLSVPRPFSRLQLQEDDDDPLIG

452.>sp|P02679|FIBG_HUMANFibrinogengammachainOS=HomosapiensGN=FGGPE=1SV=3

MSWSLHPRNLILYFYALLFLSSTCVAYVATRDNCCILDERFGSYCPTTCGIADFLSTYQT

KVDKDLQSLEDILHQVENKTSEVKQLIKAIQLTYNPDESSKPNMIDAATLKSRKMLEEIM

KYEASILTHDSSIRYLQEIYNSNNQKIVNLKEKVAQLEAQCQEPCKDTVQIHDITGKDCQ

DIANKGAKQSGLYFIKPLKANQQFLVYCEIDGSGNGWTVFQKRLDGSVDFKKNWIQYKEG

FGHLSPTGTTEFWLGNEKIHLISTQSAIPYALRVELEDWNGRTSTADYAMFKVGPEADKY

RLTYAYFAGGDAGDAFDGFDFGDDPSDKFFTSHNGMQFSTWDNDNDKFEGNCAEQDGSGW

WMNKCHAGHLNGVYYQGGTYSKASTPNGYDNGIIWATWKTRWYSMKKTTMKIIPFNRLTI

GEGQQHHLGGAKQVRPEHPAETEYDSLYPEDDL

453.>sp|Q9NRX5|SERC1_HUMANSerineincorporator1OS=HomosapiensGN=SERINC1PE=1SV=1

MGSVLGLCSMASWIPCLCGSAPCLLCRCCPSGNNSTVTRLIYALFLLVGVCVACVMLIPG

MEEQLNKIPGFCENEKGVVPCNILVGYKAVYRLCFGLAMFYLLLSLLMIKVKSSSDPRAA

VHNGFWFFKFAAAIAIIIGAFFIPEGTFTTVWFYVGMAGAFCFILIQLVLLIDFAHSWNE

SWVEKMEEGNSRCWYAALLSATALNYLLSLVAIVLFFVYYTHPASCSENKAFISVNMLLC

VGASVMSILPKIQESQPRSGLLQSSVITVYTMYLTWSAMTNEPETNCNPSLLSIIGYNTT

STVPKEGQSVQWWHAQGIIGLILFLLCVFYSSIRTSNNSQVNKLTLTSDESTLIEDGGAR

SDGSLEDGDDVHRAVDNERDGVTYSYSFFHFMLFLASLYIMMTLTNWYRYEPSREMKSQW

TAVWVKISSSWIGIVLYVWTLVAPLVLTNRDFD

454.>sp|Q68D85|NR3L1_HUMANNaturalcytotoxicitytriggeringreceptor3ligand1OS=HomosapiensGN=NCR3LG1PE=1SV=1

MTWRAAASTCAALLILLWALTTEGDLKVEMMAGGTQITPLNDNVTIFCNIFYSQPLNITS

MGITWFWKSLTFDKEVKVFEFFGDHQEAFRPGAIVSPWRLKSGDASLRLPGIQLEEAGEY

RCEVVVTPLKAQGTVQLEVVASPASRLLLDQVGMKENEDKYMCESSGFYPEAINITWEKQ

TQKFPHPIEISEDVITGPTIKNMDGTFNVTSCLKLNSSQEDPGTVYQCVVRHASLHTPLR

SNFTLTAARHSLSETEKTDNFSIHWWPISFIGVGLVLLIVLIPWKKICNKSSSAYTPLKC

ILKHWNSFDTQTLKKEHLIFFCTRAWPSYQLQDGEAWPPEGSVNINTIQQLDVFCRQEGK

WSEVPYVQAFFALRDNPDLCQCCRIDPALLTVTSGKSIDDNSTKSEKQTPREHSDAVPDA

PILPVSPIWEPPPATTSTTPVLSSQPPTLLLPLQ

(NinO.R)

455.>sp|Q96JJ7|TMX3_HUMANProteindisulfide-isomeraseTMX3OS=HomosapiensGN=TMX3PE=1SV=2

MAAWKSWTALRLCATVVVLDMVVCKGFVEDLDESFKENRNDDIWLVDFYAPWCGHCKKLE

PIWNEVGLEMKSIGSPVKVGKMDATSYSSIASEFGVRGYPTIKLLKGDLAYNYRGPRTKD

DIIEFAHRVSGALIRPLPSQQMFEHMQKRHRVFFVYVGGESPLKEKYIDAASELIVYTYF

FSASEEVVPEYVTLKEMPAVLVFKDETYFVYDEYEDGDLSSWINRERFQNYLAMDGFLLY

ELGDTGKLVALAVIDEKNTSVEHTRLKSIIQEVARDYRDLFHRDFQFGHMDGNDYINTLL

MDELTVPTVVVLNTSNQQYFLLDRQIKNVEDMVQFINNILDGTVEAQGGDSILQRLKRIV

FDAKSTIVSIFKSSPLMGCFLFGLPLGVISIMCYGIYTADTDGGYIEERYEVSKSENENQ

EQIEESKEQQEPSSGGSVVPTVQEPKDVLEKKKD

456.>sp|Q14542|S29A2_HUMANEquilibrativenucleosidetransporter2OS=HomosapiensGN=SLC29A2PE=1SV=3

MARGDAPRDSYHLVGISFFILGLGTLLPWNFFITAIPYFQARLAGAGNSTARILSTNHTG

PEDAFNFNNWVTLLSQLPLLLFTLLNSFLYQCVPETVRILGSLLAILLLFALTAALVKVD

MSPGPFFSITMASVCFINSFSAVLQGSLFGQLGTMPSTYSTLFLSGQGLAGIFAALAMLL

SMASGVDAETSALGYFITPCVGILMSIVCYLSLPHLKFARYYLANKSSQAQAQELETKAE

LLQSDENGIPSSPQKVALTLDLDLEKEPESEPDEPQKPGKPSVFTVFQKIWLTALCLVLV

FTVTLSVFPAITAMVTSSTSPGKWSQFFNPICCFLLFNIMDWLGRSLTSYFLWPDEDSRL

LPLLVCLRFLFVPLFMLCHVPQRSRLPILFPQDAYFITFMLLFAVSNGYLVSLTMCLAPR

QVLPHEREVAGALMTFFLALGLSCGASLSFLFKALL

(LastNinO.R)

457.>sp|Q99808|S29A1_HUMANEquilibrativenucleosidetransporter1OS=HomosapiensGN=SLC29A1PE=1SV=3

MTTSHQPQDRYKAVWLIFFMLGLGTLLPWNFFMTATQYFTNRLDMSQNVSLVTAELSKDA

QASAAPAAPLPERNSLSAIFNNVMTLCAMLPLLLFTYLNSFLHQRIPQSVRILGSLVAIL

LVFLITAILVKVQLDALPFFVITMIKIVLINSFGAILQGSLFGLAGLLPASYTAPIMSGQ

GLAGFFASVAMICAIASGSELSESAFGYFITACAVIILTIICYLGLPRLEFYRYYQQLKL

EGPGEQETKLDLISKGEEPRAGKEESGVSVSNSQPTNESHSIKAILKNISVLAFSVCFIF

TITIGMFPAVTVEVKSSIAGSSTWERYFIPVSCFLTFNIFDWLGRSLTAVFMWPGKDSRW

LPSLVLARLVFVPLLLLCNIKPRRYLTVVFEHDAWFIFFMAAFAFSNGYLASLCMCFGPK

KVKPAEAETAGAIMAFFLCLGLALGAVFSFLFRAIV

458.>sp|Q8N4F0|BPIB2_HUMANBPIfold-containingfamilyBmember2OS=HomosapiensGN=BPIFB2PE=1SV=2

MAWASRLGLLLALLLPVVGASTPGTVVRLNKAALSYVSEIGKAPLQRALQVTVPHFLDWS

GEALQPTRIRILNVHVPRLHLKFIAGFGVRLLAAANFTFKVFRAPEPLELTLPVELLADT

RVTQSSIRTPVVSISACSLFSGHANEFDGSNSTSHALLVLVQKHIKAVLSNKLCLSISNL

VQGVNVHLGTLIGLNPVGPESQIRYSMVSVPTVTSDYISLEVNAVLFLLGKPIILPTDAT

PFVLPRHVGTEGSMATVGLSQQLFDSALLLLQKAGALNLDITGQLRSDDNLLNTSALGRL

IPEVARQFPEPMPVVLKVRLGATPVAMLHTNNATLRLQPFVEVLATASNSAFQSLFSLDV

VVNLRLQLSVSKVKLQGTTSVLGDVQLTVASSNVGFIDTDQVRTLMGTVFEKPLLDHLNA

LLAMGIALPGVNLHYVAPEIFVYEGYVVISSGLFYQS

459.>sp|P01730|CD4_HUMANT-cellsurfaceglycoproteinCD4OS=HomosapiensGN=CD4PE=1SV=1

MNRGVPFRHLLLVLQLALLPAATQGKKVVLGKKGDTVELTCTASQKKSIQFHWKNSNQIK

ILGNQGSFLTKGPSKLNDRADSRRSLWDQGNFPLIIKNLKIEDSDTYICEVEDQKEEVQL

LVFGLTANSDTHLLQGQSLTLTLESPPGSSPSVQCRSPRGKNIQGGKTLSVSQLELQDSG

TWTCTVLQNQKKVEFKIDIVVLAFQKASSIVYKKEGEQVEFSFPLAFTVEKLTGSGELWW

QAERASSSKSWITFDLKNKEVSVKRVTQDPKLQMGKKLPLHLTLPQALPQYAGSGNLTLA

LEAKTGKLHQEVNLVVMRATQLQKNLTCEVWGPTSPKLMLSLKLENKEAKVSKREKAVWV

LNPEAGMWQCLLSDSGQVLLESNIKVLPTWSTPVQPMALIVLGGVAGLLLFIGLGIFFCV

RCRHRRRQAERMSQIKRLLSEKKTCQCPHRFQKTCSPI

460.>sp|Q6UWV6|ENPP7_HUMANEctonucleotidepyrophosphatase/phosphodiesterasefamilymember7OS=HomosapiensGN=ENPP7PE=1SV=3

MRGLAVLLTVALATLLAPGAGAPVQSQGSQNKLLLVSFDGFRWNYDQDVDTPNLDAMARD

GVKARYMTPAFVTMTSPCHFTLVTGKYIENHGVVHNMYYNTTSKVKLPYHATLGIQRWWD

NGSVPIWITAQRQGLRAGSFFYPGGNVTYQGVAVTRSRKEGIAHNYKNETEWRANIDTVM

AWFTEEDLDLVTLYFGEPDSTGHRYGPESPERREMVRQVDRTVGYLRESIARNHLTDRLN

LIITSDHGMTTVDKRAGDLVEFHKFPNFTFRDIEFELLDYGPNGMLLPKEGRLEKVYDAL

KDAHPKLHVYKKEAFPEAFHYANNPRVTPLLMYSDLGYVIHGRINVQFNNGEHGFDNKDM

DMKTIFRAVGPSFRAGLEVEPFESVHVYELMCRLLGIVPEANDGHLATLLPMLHTESALP

PDGRPTLLPKGRSALPPSSRPLLVMGLLGTVILLSEVA

461.>sp|Q16790|CAH9_HUMANCarbonicanhydrase9OS=HomosapiensGN=CA9PE=1SV=2

MAPLCPSPWLPLLIPAPAPGLTVQLLLSLLLLVPVHPQRLPRMQEDSPLGGGSSGEDDPL

GEEDLPSEEDSPREEDPPGEEDLPGEEDLPGEEDLPEVKPKSEEEGSLKLEDLPTVEAPG

DPQEPQNNAHRDKEGDDQSHWRYGGDPPWPRVSPACAGRFQSPVDIRPQLAAFCPALRPL

ELLGFQLPPLPELRLRNNGHSVQLTLPPGLEMALGPGREYRALQLHLHWGAAGRPGSEHT

VEGHRFPAEIHVVHLSTAFARVDEALGRPGGLAVLAAFLEEGPEENSAYEQLLSRLEEIA

EEGSETQVPGLDISALLPSDFSRYFQYEGSLTTPPCAQGVIWTVFNQTVMLSAKQLHTLS

DTLWGPGDSRLQLNFRATQPLNGRVIEASFPAGVDSSPRAAEPVQLNSCLAAGDILALVF

GLLFAVTSVAFLVQMRRQHRRGTKGGVSYRPAEVAETGA

462.>sp|P16871|IL7RA_HUMANInterleukin-7receptorsubunitalphaOS=HomosapiensGN=IL7RPE=1SV=2

MTILGTTFGMVFSLLQVVSGESGYAQNGDLEDAELDDYSFSCYSQLEVNGSQHSLTCAFE

DPDVNTTNLEFEICGALVEVKCLNFRKLQEIYFIETKKFLLIGKSNICVKVGEKSLTCKK

IDLTTIVKPEAPFDLSVIYREGANDFVVTFNTSHLQKKYVKVLMHDVAYRQEKDENKWTH

VNLSSTKLTLLQRKLQPAAMYEIKVRSIPDHYFKGFWSEWSPSYYFRTPEINNSSGEMDP

ILLTISILSFFSVALLVILACVLWKKRIKPIVWPSLPDHKKTLEHLCKKPRKNLNVSFNP

ESFLDCQIHRVDDIQARDEVEGFLQDTFPQQLEESEKQRLGGDVQSPNCPSEDVVITPES

FGRDSSLTCLAGNVSACDAPILSSSRSLDCRESGKNGPHVYQDLLLSLGTTNSTLPPPFS

LQSGILTLNPVAQGQPILTSLGSNQEEAYVTMSSFYQNQ

463.>sp|Q96NT5|PCFT_HUMANProton-coupledfolatetransporterOS=HomosapiensGN=SLC46A1PE=1SV=1

MEGSASPPEKPRARPAAAVLCRGPVEPLVFLANFALVLQGPLTTQYLWHRFSADLGYNGT

RQRGGCSNRSADPTMQEVETLTSHWTLYMNVGGFLVGLFSSTLLGAWSDSVGRRPLLVLA

SLGLLLQALVSVFVVQLQLHVGYFVLGRILCALLGDFGGLLAASFASVADVSSSRSRTFR

MALLEASIGVAGMLASLLGGHWLRAQGYANPFWLALALLIAMTLYAAFCFGETLKEPKST

RLFTFRHHRSIVQLYVAPAPEKSRKHLALYSLAIFVVITVHFGAQDILTLYELSTPLCWD

SKLIGYGSAAQHLPYLTSLLALKLLQYCLADAWVAEIGLAFNILGMVVFAFATITPLMFT

GYGLLFLSLVITPVIRAKLSKLVRETEQGALFSAVACVNSLAMLTASGIFNSLYPATLNF

MKGFPFLLGAGLLLIPAVLIGMLEKADPHLEFQQFPQSP

464.>sp|Q687X5|STEA4_HUMANMetalloreductaseSTEAP4OS=HomosapiensGN=STEAP4PE=1SV=1

MEKTCIDALPLTMNSSEKQETVCIFGTGDFGRSLGLKMLQCGYSVVFGSRNPQKTTLLPS

GAEVLSYSEAAKKSGIIIIAIHREHYDFLTELTEVLNGKILVDISNNLKINQYPESNAEY

LAHLVPGAHVVKAFNTISAWALQSGALDASRQVFVCGNDSKAKQRVMDIVRNLGLTPMDQ

GSLMAAKEIEKYPLQLFPMWRFPFYLSAVLCVFLFFYCVIRDVIYPYVYEKKDNTFRMAI

SIPNRIFPITALTLLALVYLPGVIAAILQLYRGTKYRRFPDWLDHWMLCRKQLGLVALGF

AFLHVLYTLVIPIRYYVRWRLGNLTVTQAILKKENPFSTSSAWLSDSYVALGILGFFLFV

LLGITSLPSVSNAVNWREFRFVQSKLGYLTLILCTAHTLVYGGKRFLSPSNLRWYLPAAY

VLGLIIPCTVLVIKFVLIMPCVDNTLTRIRQGWERNSKH

465.>sp|Q9Y5C1|ANGL3_HUMANAngiopoietin-relatedprotein3OS=HomosapiensGN=ANGPTL3PE=1SV=1

MFTIKLLLFIVPLVISSRIDQDNSSFDSLSPEPKSRFAMLDDVKILANGLLQLGHGLKDF

VHKTKGQINDIFQKLNIFDQSFYDLSLQTSEIKEEEKELRRTTYKLQVKNEEVKNMSLEL

NSKLESLLEEKILLQQKVKYLEEQLTNLIQNQPETPEHPEVTSLKTFVEKQDNSIKDLLQ

TVEDQYKQLNQQHSQIKEIENQLRRTSIQEPTEISLSSKPRAPRTTPFLQLNEIRNVKHD

GIPAECTTIYNRGEHTSGMYAIRPSNSQVFHVYCDVISGSPWTLIQHRIDGSQNFNETWE

NYKYGFGRLDGEFWLGLEKIYSIVKQSNYVLRIELEDWKDNKHYIEYSFYLGNHETNYTL

HLVAITGNVPNAIPENKDLVFSTWDHKAKGHFNCPEGYSGGWWWHDECGENNLNGKYNKP

RAKSKPERRRGLSWKSQNGRLYSIKSTKMLIHPTDSESFE

466.>sp|Q68CJ9|CR3L3_HUMANCyclicAMP-responsiveelement-bindingprotein3-likeprotein3OS=HomosapiensGN=CREB3L3PE=1SV=2

MNTDLAAGKMASAACSMDPIDSFELLDLLFDRQDGILRHVELGEGWGHVKDQQVLPNPDS

DDFLSSILGSGDSLPSSPLWSPEGSDSGISEDLPSDPQDTPPRSGPATSPAGCHPAQPGK

GPCLSYHPGNSCSTTTPGPVIQVPEASVTIDLEMWSPGGRICAEKPADPVDLSPRCNLTV

KDLLLSGSSGDLQQHHLGASYLLRPGAGHCQELVLTEDEKKLLAKEGITLPTQLPLTKYE

ERVLKKIRRKIRNKQSAQESRKKKKEYIDGLETRMSACTAQNQELQRKVLHLEKQNLSLL

EQLKKLQAIVVQSTSKSAQTGTCVAVLLLSFALIILPSISPFGPNKTESPGDFAPVRVFS

RTLHNDAASRVAADAVPGSEAPGPRPEADTTREESPGSPGADWGFQDTANLTNSTEELDN

ATLVLRNATEGLGQVALLDWVAPGPSTGSGRAGLEAAGDEL

(LastNinO.R)

467.>sp|P04070|PROC_HUMANVitaminK-dependentproteinCOS=HomosapiensGN=PROCPE=1SV=1

MWQLTSLLLFVATWGISGTPAPLDSVFSSSERAHQVLRIRKRANSFLEELRHSSLERECI

EEICDFEEAKEIFQNVDDTLAFWSKHVDGDQCLVLPLEHPCASLCCGHGTCIDGIGSFSC

DCRSGWEGRFCQREVSFLNCSLDNGGCTHYCLEEVGWRRCSCAPGYKLGDDLLQCHPAVK

FPCGRPWKRMEKKRSHLKRDTEDQEDQVDPRLIDGKMTRRGDSPWQVVLLDSKKKLACGA

VLIHPSWVLTAAHCMDESKKLLVRLGEYDLRRWEKWELDLDIKEVFVHPNYSKSTTDNDI

ALLHLAQPATLSQTIVPICLPDSGLAERELNQAGQETLVTGWGYHSSREKEAKRNRTFVL

NFIKIPVVPHNECSEVMSNMVSENMLCAGILGDRQDACEGDSGGPMVASFHGTWFLVGLV

SWGEGCGLLHNYGVYTKVSRYLDWIHGHIRDKEAPQKSWAP

468.>sp|Q9H173|SIL1_HUMANNucleotideexchangefactorSIL1OS=HomosapiensGN=SIL1PE=1SV=1

MAPQSLPSSRMAPLGMLLGLLMAACFTFCLSHQNLKEFALTNPEKSSTKETERKETKAEE

ELDAEVLEVFHPTHEWQALQPGQAVPAGSHVRLNLQTGEREAKLQYEDKFRNNLKGKRLD

INTNTYTSQDLKSALAKFKEGAEMESSKEDKARQAEVKRLFRPIEELKKDFDELNVVIET

DMQIMVRLINKFNSSSSSLEEKIAALFDLEYYVHQMDNAQDLLSFGGLQVVINGLNSTEP

LVKEYAAFVLGAAFSSNPKVQVEAIEGGALQKLLVILATEQPLTAKKKVLFALCSLLRHF

PYAQRQFLKLGGLQVLRTLVQEKGTEVLAVRVVTLLYDLVTEKMFAEEEAELTQEMSPEK

LQQYRQVHLLPGLWEQGWCEITAHLLALPEHDAREKVLQTLGVLLTTCRDRYRQDPQLGR

TLASLQAEYQVLASLELQDGEDEGYFQELLGSVNSLLKELR

469.>sp|P02790|HEMO_HUMANHemopexinOS=HomosapiensGN=HPXPE=1SV=2

MARVLGAPVALGLWSLCWSLAIATPLPPTSAHGNVAEGETKPDPDVTERCSDGWSFDATT

LDDNGTMLFFKGEFVWKSHKWDRELISERWKNFPSPVDAAFRQGHNSVFLIKGDKVWVYP

PEKKEKGYPKLLQDEFPGIPSPLDAAVECHRGECQAEGVLFFQGDREWFWDLATGTMKER

SWPAVGNCSSALRWLGRYYCFQGNQFLRFDPVRGEVPPRYPRDVRDYFMPCPGRGHGHRN

GTGHGNSTHHGPEYMRCSPHLVLSALTSDNHGATYAFSGTHYWRLDTSRDGWHSWPIAHQ

WPQGPSAVDAAFSWEEKLYLVQGTQVYVFLTKGGYTLVSGYPKRLEKEVGTPHGIILDSV

DAAFICPGSSRLHIMAGRRLWWLDLKSGAQATWTELPWPHEKVDGALCMEKSLGPNSCSA

NGPGLYLIHGPNLYCYSDVEKLNAAKALPQPQNVTSLLGCTH

470.>sp|Q15465|SHH_HUMANSonichedgehogproteinOS=HomosapiensGN=SHHPE=1SV=1

MLLLARCLLLVLVSSLLVCSGLACGPGRGFGKRRHPKKLTPLAYKQFIPNVAEKTLGASG

RYEGKISRNSERFKELTPNYNPDIIFKDEENTGADRLMTQRCKDKLNALAISVMNQWPGV

KLRVTEGWDEDGHHSEESLHYEGRAVDITTSDRDRSKYGMLARLAVEAGFDWVYYESKAH

IHCSVKAENSVAAKSGGCFPGSATVHLEQGGTKLVKDLSPGDRVLAADDQGRLLYSDFLT

FLDRDDGAKKVFYVIETREPRERLLLTAAHLLFVAPHNDSATGEPEASSGSGPPSGGALG

PRALFASRVRPGQRVYVVAERDGDRRLLPAAVHSVTLSEEAAGAYAPLTAQGTILINRVL

ASCYAVIEEHSWAHRAFAPFRLAHALLAALAPARTDRGGDSGGGDRGGGGGRVALTAPGA

ADAPGAGATAGIHWYSQLLYQIGTWLLDSEALHPLGMAVKSS

Note:N glycosylation in the D.R.

471.>sp|P53634|CATC_HUMANDipeptidylpeptidase1OS=HomosapiensGN=CTSCPE=1SV=2

MGAGPSLLLAALLLLLSGDGAVRCDTPANCTYLDLLGTWVFQVGSSGSQRDVNCSVMGPQ

EKKVVVYLQKLDTAYDDLGNSGHFTIIYNQGFEIVLNDYKWFAFFKYKEEGSKVTTYCNE

TMTGWVHDVLGRNWACFTGKKVGTASENVYVNIAHLKNSQEKYSNRLYKYDHNFVKAINA

IQKSWTATTYMEYETLTLGDMIRRSGGHSRKIPRPKPAPLTAEIQQKILHLPTSWDWRNV

HGINFVSPVRNQASCGSCYSFASMGMLEARIRILTNNSQTPILSPQEVVSCSQYAQGCEG

GFPYLIAGKYAQDFGLVEEACFPYTGTDSPCKMKEDCFRYYSSEYHYVGGFYGGCNEALM

KLELVHHGPMAVAFEVYDDFLHYKKGIYHHTGLRDPFNPFELTNHAVLLVGYGTDSASGM

DYWIVKNSWGTGWGENGYFRIRRGTDECAIESIAVAATPIPKL

472.>sp|P43220|GLP1R_HUMANGlucagon-likepeptide1receptorOS=HomosapiensGN=GLP1RPE=1SV=2

MAGAPGPLRLALLLLGMVGRAGPRPQGATVSLWETVQKWREYRRQCQRSLTEDPPPATDL

FCNRTFDEYACWPDGEPGSFVNVSCPWYLPWASSVPQGHVYRFCTAEGLWLQKDNSSLPW

RDLSECEESKRGERSSPEEQLLFLYIIYTVGYALSFSALVIASAILLGFRHLHCTRNYIH

LNLFASFILRALSVFIKDAALKWMYSTAAQQHQWDGLLSYQDSLSCRLVFLLMQYCVAAN

YYWLLVEGVYLYTLLAFSVLSEQWIFRLYVSIGWGVPLLFVVPWGIVKYLYEDEGCWTRN

SNMNYWLIIRLPILFAIGVNFLIFVRVICIVVSKLKANLMCKTDIKCRLAKSTLTLIPLL

GTHEVIFAFVMDEHARGTLRFIKLFTELSFTSFQGLMVAILYCFVNNEVQLEFRKSWERW

RLEHLHIQRDSSMKPLKCPTSSLSSGATAGSSMYTATCQASCS

473.>sp|P01008|ANT3_HUMANAntithrombin-IIIOS=HomosapiensGN=SERPINC1PE=1SV=1

MYSNVIGTVTSGKRKVYLLSLLLIGFWDCVTCHGSPVDICTAKPRDIPMNPMCIYRSPEK

KATEDEGSEQKIPEATNRRVWELSKANSRFATTFYQHLADSKNDNDNIFLSPLSISTAFA

MTKLGACNDTLQQLMEVFKFDTISEKTSDQIHFFFAKLNCRLYRKANKSSKLVSANRLFG

DKSLTFNETYQDISELVYGAKLQPLDFKENAEQSRAAINKWVSNKTEGRITDVIPSEAIN

ELTVLVLVNTIYFKGLWKSKFSPENTRKELFYKADGESCSASMMYQEGKFRYRRVAEGTQ

VLELPFKGDDITMVLILPKPEKSLAKVEKELTPEVLQEWLDELEEMMLVVHMPRFRIEDG

FSLKEQLQDMGLVDLFSPEKSKLPGIVAEGRDDLYVSDAFHKAFLEVNEEGSEAAASTAV

VIAGRSLNPNRVTFKANRPFLVFIREVPLNTIIFMGRVANPCVK

474.>sp|O75311|GLRA3_HUMANGlycinereceptorsubunitalpha-3OS=HomosapiensGN=GLRA3PE=1SV=2

MAHVRHFRTLVSGFYFWEAALLLSLVATKETDSARSRSAPMSPSDFLDKLMGRTSGYDAR

IRPNFKGPPVNVTCNIFINSFGSIAETTMDYRVNIFLRQKWNDPRLAYSEYPDDSLDLDP

SMLDSIWKPDLFFANEKGANFHEVTTDNKLLRIFKNGNVLYSIRLTLTLSCPMDLKNFPM

DVQTCIMQLESFGYTMNDLIFEWQDEAPVQVAEGLTLPQFLLKEEKDLRYCTKHYNTGKF

TCIEVRFHLERQMGYYLIQMYIPSLLIVILSWVSFWINMDAAPARVALGITTVLTMTTQS

SGSRASLPKVSYVKAIDIWMAVCLLFVFSALLEYAAVNFVSRQHKELLRFRRKRKNKTEA

FALEKFYRFSDMDDEVRESRFSFTAYGMGPCLQAKDGMTPKGPNHPVQVMPKSPDEMRKV

FIDRAKKIDTISRACFPLAFLIFNIFYWVIYKILRHEDIHQQQD

475.>sp|P16233|LIPP_HUMANPancreatictriacylglycerollipaseOS=HomosapiensGN=PNLIPPE=1SV=1

MLPLWTLSLLLGAVAGKEVCYERLGCFSDDSPWSGITERPLHILPWSPKDVNTRFLLYTN

ENPNNFQEVAADSSSISGSNFKTNRKTRFIIHGFIDKGEENWLANVCKNLFKVESVNCIC

VDWKGGSRTGYTQASQNIRIVGAEVAYFVEFLQSAFGYSPSNVHVIGHSLGAHAAGEAGR

RTNGTIGRITGLDPAEPCFQGTPELVRLDPSDAKFVDVIHTDGAPIVPNLGFGMSQVVGH

LDFFPNGGVEMPGCKKNILSQIVDIDGIWEGTRDFAACNHLRSYKYYTDSIVNPDGFAGF

PCASYNVFTANKCFPCPSGGCPQMGHYADRYPGKTNDVGQKFYLDTGDASNFARWRYKVS

VTLSGKKVTGHILVSLFGNKGNSKQYEIFKGTLKPDSTHSNEFDSDVDVGDLQMVKFIWY

NNVINPTLPRVGASKIIVETNVGKQFNFCSPETVREEVLLTLTPC

476.>sp|Q13231|CHIT1_HUMANChitotriosidase-1OS=HomosapiensGN=CHIT1PE=1SV=1

MVRSVAWAGFMVLLMIPWGSAAKLVCYFTNWAQYRQGEARFLPKDLDPSLCTHLIYAFAG

MTNHQLSTTEWNDETLYQEFNGLKKMNPKLKTLLAIGGWNFGTQKFTDMVATANNRQTFV

NSAIRFLRKYSFDGLDLDWEYPGSQGSPAVDKERFTTLVQDLANAFQQEAQTSGKERLLL

SAAVPAGQTYVDAGYEVDKIAQNLDFVNLMAYDFHGSWEKVTGHNSPLYKRQEESGAAAS

LNVDAAVQQWLQKGTPASKLILGMPTYGRSFTLASSSDTRVGAPATGSGTPGPFTKEGGM

LAYYEVCSWKGATKQRIQDQKVPYIFRDNQWVGFDDVESFKTKVSYLKQKGLGGAMVWAL

DLDDFAGFSCNQGRYPLIQTLRQELSLPYLPSGTPELEVPKPGQPSEPEHGPSPGQDTFC

QGKADGLYPNPRERSSFYSCAAGRLFQQSCPTGLVFSNSCKCCTWN

submittedas477.

477.>sp|P08709|FA7_HUMANCoagulationfactorVIIOS=HomosapiensGN=F7PE=1SV=1

MVSQALRLLCLLLGLQGCLAAGGVAKASGGETRDMPWKPGPHRVFVTQEEAHGVLHRRRR

ANAFLEELRPGSLERECKEEQCSFEEAREIFKDAERTKLFWISYSDGDQCASSPCQNGGS

CKDQLQSYICFCLPAFEGRNCETHKDDQLICVNENGGCEQYCSDHTGTKRSCRCHEGYSL

LADGVSCTPTVEYPCGKIPILEKRNASKPQGRIVGGKVCPKGECPWQVLLLVNGAQLCGG

TLINTIWVVSAAHCFDKIKNWRNLIAVLGEHDLSEHDGDEQSRRVAQVIIPSTYVPGTTN

HDIALLRLHQPVVLTDHVVPLCLPERTFSERTLAFVRFSLVSGWGQLLDRGATALELMVL

NVPRLMTQDCLQQSRKVGDSPNITEYMFCAGYSDGSKDSCKGDSGGPHATHYRGTWYLTG

IVSWGQGCATVGHFGVYTRVSQYIEWLQKLMRSEPRPGVLLRAPFP

478.>sp|P04066|FUCO_HUMANTissuealpha-L-fucosidaseOS=HomosapiensGN=FUCA1PE=1SV=4

MRAPGMRSRPAGPALLLLLLFLGAAESVRRAQPPRRYTPDWPSLDSRPLPAWFDEAKFGV

FIHWGVFSVPAWGSEWFWWHWQGEGRPQYQRFMRDNYPPGFSYADFGPQFTARFFHPEEW

ADLFQAAGAKYVVLTTKHHEGFTNWPSPVSWNWNSKDVGPHRDLVGELGTALRKRNIRYG

LYHSLLEWFHPLYLLDKKNGFKTQHFVSAKTMPELYDLVNSYKPDLIWSDGEWECPDTYW

NSTNFLSWLYNDSPVKDEVVVNDRWGQNCSCHHGGYYNCEDKFKPQSLPDHKWEMCTSID

KFSWGYRRDMALSDVTEESEIISELVQTVSLGGNYLLNIGPTKDGLIVPIFQERLLAVGK

WLSINGEAIYASKPWRVQWEKNTTSVWYTSKGSAVYAIFLHWPENGVLNLESPITTSTTK

ITMLGIQGDLKWSTDPDKGLFISLPQLPPSAVPAEFAWTIKLTGVK

479.>sp|P48546|GIPR_HUMANGastricinhibitorypolypeptidereceptorOS=HomosapiensGN=GIPRPE=1SV=1

MTTSPILQLLLRLSLCGLLLQRAETGSKGQTAGELYQRWERYRRECQETLAAAEPPSGLA

CNGSFDMYVCWDYAAPNATARASCPWYLPWHHHVAAGFVLRQCGSDGQWGLWRDHTQCEN

PEKNEAFLDQRLILERLQVMYTVGYSLSLATLLLALLILSLFRRLHCTRNYIHINLFTSF

MLRAAAILSRDRLLPRPGPYLGDQALALWNQALAACRTAQIVTQYCVGANYTWLLVEGVY

LHSLLVLVGGSEEGHFRYYLLLGWGAPALFVIPWVIVRYLYENTQCWERNEVKAIWWIIR

TPILMTILINFLIFIRILGILLSKLRTRQMRCRDYRLRLARSTLTLVPLLGVHEVVFAPV

TEEQARGALRFAKLGFEIFLSSFQGFLVSVLYCFINKEVQSEIRRGWHHCRLRRSLGEEQ

RQLPERAFRALPSGSGPGEVPTSRGLSSGTLPGPGNEASRELESYC

480.>sp|Q86YB8|ERO1B_HUMANERO1-likeproteinbetaOS=HomosapiensGN=ERO1BPE=1SV=2

MSQGVRRAGAGQGVAAAVQLLVTLSFLRSVVEAQVTGVLDDCLCDIDSIDNFNTYKIFPK

IKKLQERDYFRYYKVNLKRPCPFWAEDGHCSIKDCHVEPCPESKIPVGIKAGHSNKYLKM

ANNTKELEDCEQANKLGAINSTLSNQSKEAFIDWARYDDSRDHFCELDDERSPAAQYVDL

LLNPERYTGYKGTSAWRVWNSIYEENCFKPRSVYRPLNPLAPSRGEDDGESFYTWLEGLC

LEKRVFYKLISGLHASINLHLCANYLLEETWGKPSWGPNIKEFKHRFDPVETKGEGPRRL

KNLYFLYLIELRALSKVAPYFERSIVDLYTGNAEEDADTKTLLLNIFQDTKSFPMHFDEK

SMFAGDKKGAKSLKEEFRLHFKNISRIMDCVGCDKCRLWGKLQTQGLGTALKILFSEKEI

QKLPENSPSKGFQLTRQEIVALLNAFGRLSTSIRDLQNFKVLLQHSR

481.>sp|Q9BTY2|FUCO2_HUMANPlasmaalpha-L-fucosidaseOS=HomosapiensGN=FUCA2PE=1SV=2

MRPQELPRLAFPLLLLLLLLLPPPPCPAHSATRFDPTWESLDARQLPAWFDQAKFGIFIH

WGVFSVPSFGSEWFWWYWQKEKIPKYVEFMKDNYPPSFKYEDFGPLFTAKFFNANQWADI

FQASGAKYIVLTSKHHEGFTLWGSEYSWNWNAIDEGPKRDIVKELEVAIRNRTDLRFGLY

YSLFEWFHPLFLEDESSSFHKRQFPVSKTLPELYELVNNYQPEVLWSDGDGGAPDQYWNS

TGFLAWLYNESPVRGTVVTNDRWGAGSICKHGGFYTCSDRYNPGHLLPHKWENCMTIDKL

SWGYRREAGISDYLTIEELVKQLVETVSCGGNLLMNIGPTLDGTISVVFEERLRQMGSWL

KVNGEAIYETHTWRSQNDTVTPDVWYTSKPKEKLVYAIFLKWPTSGQLFLGHPKAILGAT

EVKLLGHGQPLNWISLEQNGIMVELPQLTIHQMPCKWGWALALTNVI

482.>sp|P22894|MMP8_HUMANNeutrophilcollagenaseOS=HomosapiensGN=MMP8PE=1SV=1

MFSLKTLPFLLLLHVQISKAFPVSSKEKNTKTVQDYLEKFYQLPSNQYQSTRKNGTNVIV

EKLKEMQRFFGLNVTGKPNEETLDMMKKPRCGVPDSGGFMLTPGNPKWERTNLTYRIRNY

TPQLSEAEVERAIKDAFELWSVASPLIFTRISQGEADINIAFYQRDHGDNSPFDGPNGIL

AHAFQPGQGIGGDAHFDAEETWTNTSANYNLFLVAAHEFGHSLGLAHSSDPGALMYPNYA

FRETSNYSLPQDDIDGIQAIYGLSSNPIQPTGPSTPKPCDPSLTFDAITTLRGEILFFKD

RYFWRRHPQLQRVEMNFISLFWPSLPTGIQAAYEDFDRDLIFLFKGNQYWALSGYDILQG

YPKDISNYGFPSSVQAIDAAVFYRSKTYFFVNDQFWRYDNQRQFMEPGYPKSISGAFPGI

ESKVDAVFQQEHFFHVFSGPRYYAFDLIAQRVTRVARGNKWLNCRYG

483.>sp|Q9Y286|SIGL7_HUMANSialicacid-bindingIg-likelectin7OS=HomosapiensGN=SIGLEC7PE=1SV=1

MLLLLLLPLLWGRERVEGQKSNRKDYSLTMQSSVTVQEGMCVHVRCSFSYPVDSQTDSDP

VHGYWFRAGNDISWKAPVATNNPAWAVQEETRDRFHLLGDPQTKNCTLSIRDARMSDAGR

YFFRMEKGNIKWNYKYDQLSVNVTALTHRPNILIPGTLESGCFQNLTCSVPWACEQGTPP

MISWMGTSVSPLHPSTTRSSVLTLIPQPQHHGTSLTCQVTLPGAGVTTNRTIQLNVSYPP

QNLTVTVFQGEGTASTALGNSSSLSVLEGQSLRLVCAVDSNPPARLSWTWRSLTLYPSQP

SNPLVLELQVHLGDEGEFTCRAQNSLGSQHVSLNLSLQQEYTGKMRPVSGVLLGAVGGAG

ATALVFLSFCVIFIVVRSCRKKSARPAADVGDIGMKDANTIRGSASQGNLTESWADDNPR

HHGLAAHSSGEEREIQYAPLSFHKGEPQDLSGQEATNNEYSEIKIPK

Note:all N’s in the O.R

484.>sp|Q9GZM7|TINAL_HUMANTubulointerstitialnephritisantigen-likeOS=HomosapiensGN=TINAGL1PE=1SV=1

MWRCPLGLLLLLPLAGHLALGAQQGRGRRELAPGLHLRGIRDAGGRYCQEQDLCCRGRAD

DCALPYLGAICYCDLFCNRTVSDCCPDFWDFCLGVPPPFPPIQGCMHGGRIYPVLGTYWD

NCNRCTCQENRQWQCDQEPCLVDPDMIKAINQGNYGWQAGNHSAFWGMTLDEGIRYRLGT

IRPSSSVMNMHEIYTVLNPGEVLPTAFEASEKWPNLIHEPLDQGNCAGSWAFSTAAVASD

RVSIHSLGHMTPVLSPQNLLSCDTHQQQGCRGGRLDGAWWFLRRRGVVSDHCYPFSGRER

DEAGPAPPCMMHSRAMGRGKRQATAHCPNSYVNNNDIYQVTPVYRLGSNDKEIMKELMEN

GPVQALMEVHEDFFLYKGGIYSHTPVSLGRPERYRRHGTHSVKITGWGEETLPDGRTLKY

WTAANSWGPAWGERGHFRIVRGVNECDIESFVLGVWGRVGMEDMGHH

485.>sp|Q96HE7|ERO1A_HUMANERO1-likeproteinalphaOS=HomosapiensGN=ERO1APE=1SV=2

MGRGWGFLFGLLGAVWLLSSGHGEEQPPETAAQRCFCQVSGYLDDCTCDVETIDRFNNYR

LFPRLQKLLESDYFRYYKVNLKRPCPFWNDISQCGRRDCAVKPCQSDEVPDGIKSASYKY

SEEANNLIEECEQAERLGAVDESLSEETQKAVLQWTKHDDSSDNFCEADDIQSPEAEYVD

LLLNPERYTGYKGPDAWKIWNVIYEENCFKPQTIKRPLNPLASGQGTSEENTFYSWLEGL

CVEKRAFYRLISGLHASINVHLSARYLLQETWLEKKWGHNITEFQQRFDGILTEGEGPRR

LKNLYFLYLIELRALSKVLPFFERPDFQLFTGNKIQDEENKMLLLEILHEIKSFPLHFDE

NSFFAGDKKEAHKLKEDFRLHFRNISRIMDCVGCFKCRLWGKLQTQGLGTALKILFSEKL

IANMPESGPSYEFHLTRQEIVSLFNAFGRISTSVKELENFRNLLQNIH

486.>sp|P08887|IL6RA_HUMANInterleukin-6receptorsubunitalphaOS=HomosapiensGN=IL6RPE=1SV=1

MLAVGCALLAALLAAPGAALAPRRCPAQEVARGVLTSLPGDSVTLTCPGVEPEDNATVHW

VLRKPAAGSHPSRWAGMGRRLLLRSVQLHDSGNYSCYRAGRPAGTVHLLVDVPPEEPQLS

CFRKSPLSNVVCEWGPRSTPSLTTKAVLLVRKFQNSPAEDFQEPCQYSQESQKFSCQLAV

PEGDSSFYIVSMCVASSVGSKFSKTQTFQGCGILQPDPPANITVTAVARNPRWLSVTWQD

PHSWNSSFYRLRFELRYRAERSKTFTTWMVKDLQHHCVIHDAWSGLRHVVQLRAQEEFGQ

GEWSEWSPEAMGTPWTESRSPPAENEVSTPMQALTTNKDDDNILFRDSANATSLPVQDSS

SVPLPTFLVAGGSLAFGTLLCIAIVLRFKKTWKLRALKEGKTSMHPPYSLGQLVPERPRP

TPVLVPLISPPVSPSSLGSDNTSSHNRPDARDPRSPYDISNTDYFFPR

487.>sp|P54317|LIPR2_HUMANPancreaticlipase-relatedprotein2OS=HomosapiensGN=PNLIPRP2PE=1SV=1

MLPPWTLGLLLLATVRGKEVCYGQLGCFSDEKPWAGTLQRPVKLLPWSPEDIDTRFLLYT

NENPNNFQLITGTEPDTIEASNFQLDRKTRFIIHGFLDKAEDSWPSDMCKKMFEVEKVNC

ICVDWRHGSRAMYTQAVQNIRVVGAETAFLIQALSTQLGYSLEDVHVIGHSLGAHTAAEA

GRRLGGRVGRITGLDPAGPCFQDEPEEVRLDPSDAVFVDVIHTDSSPIVPSLGFGMSQKV

GHLDFFPNGGKEMPGCKKNVLSTITDIDGIWEGIGGFVSCNHLRSFEYYSSSVLNPDGFL

GYPCASYDEFQESKCFPCPAEGCPKMGHYADQFKGKTSAVEQTFFLNTGESGNFTSWRYK

VSVTLSGKEKVNGYIRIALYGSNENSKQYEIFKGSLKPDASHTCAIDVDFNVGKIQKVKF

LWNKRGINLSEPKLGASQITVQSGEDGTEYNFCSSDTVEENVLQSLYPC

488.>sp|P03956|MMP1_HUMANInterstitialcollagenaseOS=HomosapiensGN=MMP1PE=1SV=3

MHSFPPLLLLLFWGVVSHSFPATLETQEQDVDLVQKYLEKYYNLKNDGRQVEKRRNSGPV

VEKLKQMQEFFGLKVTGKPDAETLKVMKQPRCGVPDVAQFVLTEGNPRWEQTHLTYRIEN

YTPDLPRADVDHAIEKAFQLWSNVTPLTFTKVSEGQADIMISFVRGDHRDNSPFDGPGGN

LAHAFQPGPGIGGDAHFDEDERWTNNFREYNLHRVAAHELGHSLGLSHSTDIGALMYPSY

TFSGDVQLAQDDIDGIQAIYGRSQNPVQPIGPQTPKACDSKLTFDAITTIRGEVMFFKDR

FYMRTNPFYPEVELNFISVFWPQLPNGLEAAYEFADRDEVRFFKGNKYWAVQGQNVLHGY

PKDIYSSFGFPRTVKHIDAALSEENTGKTYFFVANKYWRYDEYKRSMDPGYPKMIAHDFP

GIGHKVDAVFMKDGFFYFFHGTRQYKFDPKTKRILTLQKANSWFNCRKN

489.>sp|P27918|PROP_HUMANProperdinOS=HomosapiensGN=CFPPE=1SV=2

MITEGAQAPRLLLPPLLLLLTLPATGSDPVLCFTQYEESSGKCKGLLGGGVSVEDCCLNT

AFAYQKRSGGLCQPCRSPRWSLWSTWAPCSVTCSEGSQLRYRRCVGWNGQCSGKVAPGTL

EWQLQACEDQQCCPEMGGWSGWGPWEPCSVTCSKGTRTRRRACNHPAPKCGGHCPGQAQE

SEACDTQQVCPTHGAWATWGPWTPCSASCHGGPHEPKETRSRKCSAPEPSQKPPGKPCPG

LAYEQRRCTGLPPCPVAGGWGPWGPVSPCPVTCGLGQTMEQRTCNHPVPQHGGPFCAGDA

TRTHICNTAVPCPVDGEWDSWGEWSPCIRRNMKSISCQEIPGQQSRGRTCRGRKFDGHRC

AGQQQDIRHCYSIQHCPLKGSWSEWSTWGLCMPPCGPNPTRARQRLCTPLLPKYPPTVSM

VEGQGEKNVTFWGRPLPRCEELQGQKLVVEEKRPCLHVPACKDPEEEEL

490.>sp|O15204|ADEC1_HUMANADAMDEC1OS=HomosapiensGN=ADAMDEC1PE=1SV=2

MLRGISQLPAVATMSWVLLPVLWLIVQTQAIAIKQTPELTLHEIVCPKKLHILHKREIKN

NQTEKHGKEERYEPEVQYQMILNGEEIILSLQKTKHLLGPDYTETLYSPRGEEITTKPEN

MEHCYYKGNILNEKNSVASISTCDGLRGYFTHHHQRYQIKPLKSTDEKEHAVFTSNQEEQ

DPANHTCGVKSTDGKQGPIRISRSLKSPEKEDFLRAQKYIDLYLVLDNAFYKNYNENLTL

IRSFVFDVMNLLNVIYNTIDVQVALVGMEIWSDGDKIKVVPSASTTFDNFLRWHSSNLGK

KIHDHAQLLSGISFNNRRVGLAASNSLCSPSSVAVIEAKKKNNVALVGVMSHELGHVLGM

PDVPFNTKCPSGSCVMNQYLSSKFPKDFSTSCRAHFERYLLSQKPKCLLQAPIPTNIMTT

PVCGNHLLEVGEDCDCGSPKECTNLCCEALTCKLKPGTDCGGDAPNHTTE

491.>sp|Q8NI99|ANGL6_HUMANAngiopoietin-relatedprotein6OS=HomosapiensGN=ANGPTL6PE=1SV=1

MGKPWLRALQLLLLLGASWARAGAPRCTYTFVLPPQKFTGAVCWSGPASTRATPEAANAS

ELAALRMRVGRHEELLRELQRLAAADGAVAGEVRALRKESRGLSARLGQLRAQLQHEAGP

GAGPGADLGAEPAAALALLGERVLNASAEAQRAAARFHQLDVKFRELAQLVTQQSSLIAR

LERLCPGGAGGQQQVLPPPPLVPVVPVRLVGSTSDTSRMLDPAPEPQRDQTQRQQEPMAS

PMPAGHPAVPTKPVGPWQDCAEARQAGHEQSGVYELRVGRHVVSVWCEQQLEGGGWTVIQ

RRQDGSVNFFTTWQHYKAGFGRPDGEYWLGLEPVYQLTSRGDHELLVLLEDWGGRGARAH

YDGFSLEPESDHYRLRLGQYHGDAGDSLSWHNDKPFSTVDRDRDSYSGNCALYQRGGWWY

HACAHSNLNGVWHHGGHYRSRYQDGVYWAEFRGGAYSLRKAAMLIRPLKL

492.>sp|Q15904|VAS1_HUMANV-typeprotonATPasesubunitS1OS=HomosapiensGN=ATP6AP1PE=1SV=2

MMAAMATARVRMGPRCAQALWRMPWLPVFLSLAAAAAAAAAEQQVPLVLWSSDRDLWAPA

ADTHEGHITSDLQLSTYLDPALELGPRNVLLFLQDKLSIEDFTAYGGVFGNKQDSAFSNL

ENALDLAPSSLVLPAVDWYAVSTLTTYLQEKLGASPLHVDLATLRELKLNASLPALLLIR

LPYTASSGLMAPREVLTGNDEVIGQVLSTLKSEDVPYTAALTAVRPSRVARDVAVVAGGL

GRQLLQKQPVSPVIHPPVSYNDTAPRILFWAQNFSVAYKDQWEDLTPLTFGVQELNLTGS

FWNDSFARLSLTYERLFGTTVTFKFILANRLYPVSARHWFTMERLEVHSNGSVAYFNASQ

VTGPSIYSFHCEYVSSLSKKGSLLVARTQPSPWQMMLQDFQIQAFNVMGEQFSYASDCAS

FFSPGIWMGLLTSLFMLFIFTYGLHMILSLKTMDRFDDHKGPTISLTQIV

493.>sp|P45452|MMP13_HUMANCollagenase3OS=HomosapiensGN=MMP13PE=1SV=1

MHPGVLAAFLFLSWTHCRALPLPSGGDEDDLSEEDLQFAERYLRSYYHPTNLAGILKENA

ASSMTERLREMQSFFGLEVTGKLDDNTLDVMKKPRCGVPDVGEYNVFPRTLKWSKMNLTY

RIVNYTPDMTHSEVEKAFKKAFKVWSDVTPLNFTRLHDGIADIMISFGIKEHGDFYPFDG

PSGLLAHAFPPGPNYGGDAHFDDDETWTSSSKGYNLFLVAAHEFGHSLGLDHSKDPGALM

FPIYTYTGKSHFMLPDDDVQGIQSLYGPGDEDPNPKHPKTPDKCDPSLSLDAITSLRGET

MIFKDRFFWRLHPQQVDAELFLTKSFWPELPNRIDAAYEHPSHDLIFIFRGRKFWALNGY

DILEGYPKKISELGLPKEVKKISAAVHFEDTGKTLLFSGNQVWRYDDTNHIMDKDYPRLI

EEDFPGIGDKVDAVYEKNGYIYFFNGPIQFEYSIWSNRIVRVMPANSILWC

494.>sp|Q9Y646|CBPQ_HUMANCarboxypeptidaseQOS=HomosapiensGN=CPQPE=1SV=1

MKFLIFAFFGGVHLLSLCSGKAICKNGISKRTFEEIKEEIASCGDVAKAIINLAVYGKAQ

NRSYERLALLVDTVGPRLSGSKNLEKAIQIMYQNLQQDGLEKVHLEPVRIPHWERGEESA

VMLEPRIHKIAILGLGSSIGTPPEGITAEVLVVTSFDELQRRASEARGKIVVYNQPYINY

SRTVQYRTQGAVEAAKVGALASLIRSVASFSIYSPHTGIQEYQDGVPKIPTACITVEDAE

MMSRMASHGIKIVIQLKMGAKTYPDTDSFNTVAEITGSKYPEQVVLVSGHLDSWDVGQGA

MDDGGGAFISWEALSLIKDLGLRPKRTLRLVLWTAEEQGGVGAFQYYQLHKVNISNYSLV

MESDAGTFLPTGLQFTGSEKARAIMEEVMSLLQPLNITQVLSHGEGTDINFWIQAGVPGA

SLLDDLYKYFFFHHSHGDTMTVMDPKQMNVAAAVWAVVSYVVADMEEMLPRS

495.>sp|P16671|CD36_HUMANPlateletglycoprotein4OS=HomosapiensGN=CD36PE=1SV=2

MGCDRNCGLIAGAVIGAVLAVFGGILMPVGDLLIQKTIKKQVVLEEGTIAFKNWVKTGTE

VYRQFWIFDVQNPQEVMMNSSNIQVKQRGPYTYRVRFLAKENVTQDAEDNTVSFLQPNGA

IFEPSLSVGTEADNFTVLNLAVAAASHIYQNQFVQMILNSLINKSKSSMFQVRTLRELLW

GYRDPFLSLVPYPVTTTVGLFYPYNNTADGVYKVFNGKDNISKVAIIDTYKGKRNLSYWE

SHCDMINGTDAASFPPFVEKSQVLQFFSSDICRSIYAVFESDVNLKGIPVYRFVLPSKAF

ASPVENPDNYCFCTEKIISKNCTSYGVLDISKCKEGRPVYISLPHFLYASPDVSEPIDGL

NPNEEEHRTYLDIEPITGFTLQFAKRLQVNLLVKPSEKIQVLKNLKRNYIVPILWLNETG

TIGDEKANMFRSQVTGKINLLGLIEMILLSVGVVMFVAFMISYCACRSKTIK

(1^st^NinD.R)

496.>sp|Q6UX04|CWC27_HUMANPeptidyl-prolylcis-transisomeraseCWC27homologOS=HomosapiensGN=CWC27PE=1SV=1

MSNIYIQEPPTNGKVLLKTTAGDIDIELWSKEAPKACRNFIQLCLEAYYDNTIFHRVVPG

FIVQGGDPTGTGSGGESIYGAPFKDEFHSRLRFNRRGLVAMANAGSHDNGSQFFFTLGRA

DELNNKHTIFGKVTGDTVYNMLRLSEVDIDDDERPHNPHKIKSCEVLFNPFDDIIPREIK

RLKKEKPEEEVKKLKPKGTKNFSLLSFGEEAEEEEEEVNRVSQSMKGKSKSSHDLLKDDP

HLSSVPVVESEKGDAPDLVDDGEDESAEHDEYIDGDEKNLMRERIAKKLKKDTSANVKSA

GEGEVEKKSVSRSEELRKEARQLKRELLAAKQKKVENAAKQAEKRSEEEEAPPDGAVAEY

RREKQKYEALRKQQSKKGTSREDQTLALLNQFKSKLTQAIAETPENDIPETEVEDDEGWM

SHVLQFEDKSRKVKDASMQDSDTFEIYDPRNPVNKRRREESKKLMREKKERR

497.>sp|P28472|GBRB3_HUMANGamma-aminobutyricacidreceptorsubunitbeta-3OS=HomosapiensGN=GABRB3PE=1SV=1

MWGLAGGRLFGIFSAPVLVAVVCCAQSVNDPGNMSFVKETVDKLLKGYDIRLRPDFGGPP

VCVGMNIDIASIDMVSEVNMDYTLTMYFQQYWRDKRLAYSGIPLNLTLDNRVADQLWVPD

TYFLNDKKSFVHGVTVKNRMIRLHPDGTVLYGLRITTTAACMMDLRRYPLDEQNCTLEIE

SYGYTTDDIEFYWRGGDKAVTGVERIELPQFSIVEHRLVSRNVVFATGAYPRLSLSFRLK

RNIGYFILQTYMPSILITILSWVSFWINYDASAARVALGITTVLTMTTINTHLRETLPKI

PYVKAIDMYLMGCFVFVFLALLEYAFVNYIFFGRGPQRQKKLAEKTAKAKNDRSKSESNR

VDAHGNILLTSLEVHNEMNEVSGGIGDTRNSAISFDNSGIQYRKQSMPREGHGRFLGDRS

LPHKKTHLRRRSSQLKIKIPDLTDVNAIDRWSRIVFPFTFSLFNLVYWLYYVN

(1^st^NinD.R)

498.>sp|Q9BZR6|RTN4R_HUMANReticulon-4receptorOS=HomosapiensGN=RTN4RPE=1SV=1

MKRASAGGSRLLAWVLWLQAWQVAAPCPGACVCYNEPKVTTSCPQQGLQAVPVGIPAASQ

RIFLHGNRISHVPAASFRACRNLTILWLHSNVLARIDAAAFTGLALLEQLDLSDNAQLRS

VDPATFHGLGRLHTLHLDRCGLQELGPGLFRGLAALQYLYLQDNALQALPDDTFRDLGNL

THLFLHGNRISSVPERAFRGLHSLDRLLLHQNRVAHVHPHAFRDLGRLMTLYLFANNLSA

LPTEALAPLRALQYLRLNDNPWVCDCRARPLWAWLQKFRGSSSEVPCSLPQRLAGRDLKR

LAANDLQGCAVATGPYHPIWTGRATDEEPLGLPKCCQPDAADKASVLEPGRPASAGNALK

GRVPPGDSPPGNGSGPRHINDSPFGTLPGSAEPPLTAVRPEGSEPPGFPTSGPRRRPGCS

RKNRTRSHCRLGQAGSGGGGTGDSEGSGALPSLTCSLTPLGLALVLWTVLGPC

499.>sp|P06858|LIPL_HUMANLipoproteinlipaseOS=HomosapiensGN=LPLPE=1SV=1

MESKALLVLTLAVWLQSLTASRGGVAAADQRRDFIDIESKFALRTPEDTAEDTCHLIPGV

AESVATCHFNHSSKTFMVIHGWTVTGMYESWVPKLVAALYKREPDSNVIVVDWLSRAQEH

YPVSAGYTKLVGQDVARFINWMEEEFNYPLDNVHLLGYSLGAHAAGIAGSLTNKKVNRIT

GLDPAGPNFEYAEAPSRLSPDDADFVDVLHTFTRGSPGRSIGIQKPVGHVDIYPNGGTFQ

PGCNIGEAIRVIAERGLGDVDQLVKCSHERSIHLFIDSLLNEENPSKAYRCSSKEAFEKG

LCLSCRKNRCNNLGYEINKVRAKRSSKMYLKTRSQMPYKVFHYQVKIHFSGTESETHTNQ

AFEISLYGTVAESENIPFTLPEVSTNKTYSFLIYTEVDIGELLMLKLKWKSDSYFSWSDW

WSSPGFAIQKIRVKAGETQKKVIFCSREKVSHLQKGKAPAVFVKCHDKSLNKKSG

500.>sp|Q9H3G5|CPVL_HUMANProbableserinecarboxypeptidaseCPVLOS=HomosapiensGN=CPVLPE=1SV=2

MVGAMWKVIVSLVLLMPGPCDGLFRSLYRSVSMPPKGDSGQPLFLTPYIEAGKIQKGREL

SLVGPFPGLNMKSYAGFLTVNKTYNSNLFFWFFPAQIQPEDAPVVLWLQGGPGGSSMFGL

FVEHGPYVVTSNMTLRDRDFPWTTTLSMLYIDNPVGTGFSFTDDTHGYAVNEDDVARDLY

SALIQFFQIFPEYKNNDFYVTGESYAGKYVPAIAHLIHSLNPVREVKINLNGIAIGDGYS

DPESIIGGYAEFLYQIGLLDEKQKKYFQKQCHECIEHIRKQNWFEAFEILDKLLDGDLTS

DPSYFQNVTGCSNYYNFLRCTEPEDQLYYVKFLSLPEVRQAIHVGNQTFNDGTIVEKYLR

EDTVQSVKPWLTEIMNNYKVLIYNGQLDIIVAAALTERSLMGMDWKGSQEYKKAEKKVWK

IFKSDSEVAGYIRQAGDFHQVIIRGGGHILPYDQPLRAFDMINRFIYGKGWDPYVG
