# Supplementary material for: Mutually exclusive locales for N-linked glycans and disorder in human glycoproteins
Source: Sci Rep. 2020 Apr 8;10:6040. doi: 10.1038/s41598-020-61427-y (PMC7142085; doi:10.1038/s41598-020-61427-y)
Supplement: Supplementary file 3 [file 41598_2020_61427_MOESM3_ESM.docx]

P13569

MQRSPLEKASVVSKLFFSWTRPILRKGYRQRLELSDIYQIPSVDSADNLSEKLEREWDRE

LASKKNPKLINALRRCFFWRFMFYGIFLYLGEVTKAVQPLLLGRIIASYDPDNKEERSIA

IYLGIGLCLLFIVRTLLLHPAIFGLHHIGMQMRIAMFSLIYKKTLKLSSRVLDKISIGQL

VSLLSNNLNKFDEGLALAHFVWIAPLQVALLMGLIWELLQASAFCGLGFLIVLALFQAGL

GRMMMKYRDQRAGKISERLVITSEMIENIQSVKAYCWEEAMEKMIENLRQTELKLTRKAA

YVRYFNSSAFFFSGFFVVFLSVLPYALIKGIILRKIFTTISFCIVLRMAVTRQFPWAVQT

WYDSLGAINKIQDFLQKQEYKTLEYNLTTTEVVMENVTAFWEEGFGELFEKAKQNNNNRK

TSNGDDSLFFSNFSLLGTPVLKDINFKIERGQLLAVAGSTGAGKTSLLMVIMGELEPSEG

KIKHSGRISFCSQFSWIMPGTIKENIIFGVSYDEYRYRSVIKACQLEEDISKFAEKDNIV

LGEGGITLSGGQRARISLARAVYKDADLYLLDSPFGYLDVLTEKEIFESCVCKLMANKTR

ILVTSKMEHLKKADKILILHEGSSYFYGTFSELQNLQPDFSSKLMGCDSFDQFSAERRNS

ILTETLHRFSLEGDAPVSWTETKKQSFKQTGEFGEKRKNSILNPINSIRKFSIVQKTPLQ

MNGIEEDSDEPLERRLSLVPDSEQGEAILPRISVISTGPTLQARRRQSVLNLMTHSVNQG

QNIHRKTTASTRKVSLAPQANLTELDIYSRRLSQETGLEISEEINEEDLKECFFDDMESI

PAVTTWNTYLRYITVHKSLIFVLIWCLVIFLAEVAASLVVLWLLGNTPLQDKG**N**STHSR**N**

NSYAVIITSTSSYYVFYIYVGVADTLLAMGFFRGLPLVHTLITVSKILHHKMLHSVLQAP

MSTLNTLKAGGILNRFSKDIAILDDLLPLTIFDFIQLLLIVIGAIAVVAVLQPYIFVATV

PVIVAFIMLRAYFLQTSQQLKQLESEGRSPIFTHLVTSLKGLWTLRAFGRQPYFETLFHK

ALNLHTANWFLYLSTLRWFQMRIEMIFVIFFIAVTFISILTTGEGEGRVGIILTLAMNIM

STLQWAVNSSIDVDSLMRSVSRVFKFIDMPTEGKPTKSTKPYKNGQLSKVMIIENSHVKK

DDIWPSGGQMTVKDLTAKYTEGGNAILENISFSISPGQRVGLLGRTGSGKSTLLSAFLRL

LNTEGEIQIDGVSWDSITLQQWRKAFGVIPQKVFIFSGTFRKNLDPYEQWSDQEIWKVAD

EVGLRSVIEQFPGKLDFVLVDGGCVLSHGHKQLMCLARSVLSKAKILLLDEPSAHLDPVT

YQIIRRTLKQAFADCTVILCEHRIEAMLECQQFLVIEENKVRQYDSIQKLLNERSLFRQA

ISPSDRVKLFPHRNSSKCKSKPQIAALKEETEEEVQDTRL

P15309

MRAAPLLLARAASLSLGFLFLLFFWLDRSVLAKELKFVTLVFRHGDRSPIDTFPTDPIKE

SSWPQGFGQLTQLGMEQHYELGEYIRKRYRKFL**N**ESYKHEQVYIRSTDVDRTLMSAMTNL

AALFPPEGVSIWNPILLWQPIPVHTVPLSEDQLLYLPFRNCPRFQELESETLKSEEFQKR

LHPYKDFIATLGKLSGLHGQDLFGIWSKVYDPLYCESVH**N**FTLPSWATEDTMTKLRELSE

LSLLSLYGIHKQKEKSRLQGGVLVNEILNHMKRATQIPSYKKLIMYSAHDTTVSGLQMAL

DVYNGLLPPYASCHLTELYFEKGEYFVEMYYR**N**ETQHEPYPLMLPGCSPSCPLERFAELV

GPVIPQDWSTECMTTNSHQGTEDSTD

P32004

MVVALRYVWPLLLCSPCLLIQIPEEYEGHHVMEPPVITEQSPRRLVVFPTDDISLKCEAS

GKPEVQFRWTRDGVHFKPKEELGVTVYQSPHSGSFTITGNNSNFAQRFQGIYRCFASNKL

GTAMSHEIRLMAEGAPKWPKETVKPVEVEEGESVVLPCNPPPSAEPLRIYWMNSKILHIK

QDERVTMGQNGNLYFANVLTSDNHSDYICHAHFPGTRTIIQKEPIDLRVKATNSMIDRKP

RLLFPTNSSSHLVALQGQPLVLECIAEGFPTPTIKWLRPSGPMPADRVTYQNHNKTLQLL

KVGEEDDGEYRCLAENSLGSARHAYYVTVEAAPYWLHKPQSHLYGPGETARLDCQVQGRP

QPEVTWRINGIPVEELAKDQKYRIQRGALILSNVQPSDTMVTQCEARNRHGLLLANAYIY

VVQLPAKILTADNQTYMAVQGSTAYLLCKAFGAPVPSVQWLDEDGTTVLQDERFFPYANG

TLGIRDLQANDTGRYFCLAANDQNNVTIMANLKVKDATQITQGPRSTIEKKGSRVTFTCQ

ASFDPSLQPSITWRGDGRDLQELGDSDKYFIEDGRLVIHSLDYSDQGNYSCVASTELDVV

ESRAQLLVVGSPGPVPRLVLSDLHLLTQSQVRVSWSPAEDHNAPIEKYDIEFEDKEMAPE

KWYSLGKVPG**N**QTSTTLKLSPYVHYTFRVTAINKYGPGEPSPVSETVVTPEAAPEKNPVD

VKGEGNETTNMVITWKPLRWMDWNAPQVQYRVQWRPQGTRGPWQEQIVSDPFLVVSNTST

FVPYEIKVQAVNSQGKGPEPQVTIGYSGEDYPQAIPELEGIEILNSSAVLVKWRPVDLAQ

VKGHLRGYNVTYWREGSQRKHSKRHIHKDHVVVPANTTSVILSGLRPYSSYHLEVQAFNG

RGSGPASEFTFSTPEGVPGHPEALHLECQSNTSLLLRWQPPLSHNGVLTGYVLSYHPLDE

GGKGQLSFNLRDPELRTHNLTDLSPHLRYRFQLQATTKEGPGEAIVREGGTMALSGISDF

GNISATAGENYSVVSWVPKEGQCNFRFHILFKALGEEKGGASLSPQYVSYNQSSYTQWDL

QPDTDYEIHLFKERMFRHQMAVKTNGTGRVRLPPAGFATEGWFIGFVSAIILLLLVLLIL

CFIKRSKGGKYSVKDKEDTQVDSEARPMKDETFGEYRSLESDNEEKAFGSSQPSLNGDIK

PLGSDDSLADYGGSVDVQFNEDGSFIGQYSGKKEKEAAGGNDSSGATSPINPAVALE

P07602

MYALFLLASLLGAALAGPVLGLKECTRGSAVWCQNVKTASDCGAVKHCLQTVWNKPTVKS

LPCDICKDVVTAAGDMLKD**N**ATEEEILVYLEKTCDWLPKP**N**MSASCKEIVDSYLPVILDI

IKGEMSRPGEVCSALNLCESLQKHLAELNHQKQLESNKIPELDMTEVVAPFMANIPLLLY

PQDGPRSKPQPKDNGDVCQDCIQMVTDIQTAVRT**N**STFVQALVEHVKEECDRLGPGMADI

CKNYISQYSEIAIQMMMHMQPKEICALVGFCDEVKEMPMQTLVPAKVASKNVIPALELVE

PIKKHEVPAKSDVYCEVCEFLVKEVTKLIDN**N**KTEKEILDAFDKMCSKLPKSLSEECQEV

VDTYGSSILSILLEEVSPELVCSMLHLCSGTRLPALTVHVTQPKDGGFCEVCKKLVGYLD

RNLEK**N**STKQEILAALEKGCSFLPDPYQKQCDQFVAEYEPVLIEILVEVMDPSFVCLKIG

ACPSAHKPLLGTEKCIWGPSYWCQNTETAAQCNAVEHCKRHVWN

P15382

MILS**N**TTAVTPFLTKLWQETVQQGG**N**MSGLARRSPRSSDGKLEALYVLMVLGFFGFFTLG

IMLSYIRSKKLEHSNDPFNVYIESDAWQEKDKAYVQARVLESYRSCYVVENHLAIEQPNT

HLPETKPSP

P21583

MKKTQTWILTCIYLQLLLFNPLVKTEGICRNRVTNNVKDVTKLVANLPKDYMITLKYVPG

MDVLPSHCWISEMVVQLSDSLTDLLDKFS**N**ISEGLSNYSIIDKLVNIVDDLVECVKE**N**SS

KDLKKSFKSPEPRLFTPEEFFRIF**N**RSIDAFKDFVVASETSDCVVSSTLSPEKDSRVSVT

KPFMLPPVAASSLRNDSSSSNRKAKNPPGDSSLHWAAMALPALFSLIIGFAFGALYWKKR

QPSLTRAVENIQINEEDNEISMLQEKEREFQEV

Q9Y6C2

MAPRTLWSCYLCCLLTAAAGAASYPPRGFSLYTGSSGALSPGGPQAQIAPRPASRHRNWC

AYVVTRTVSCVLEDGVETYVKYQPCAWGQPQCPQSIMYRRFLRPRYRVAYKTVTDMEWRC

CQGYGGDDCAESPAPALGPASSTPRPLARPARPNLSGSSAGSPLSGLGGEGPGESEKVQQ

LEEQVQSLTKELQGLRGVLQGLSGRLAEDVQRAVETAFNGRQQPADAAARPGVHETLNEI

QHQLQLLDTRVSTHDQELGHLNNHHGGSSSSGGSRAPAPASAPPGPSEELLRQLEQRLQE

SCSVCLAGLDGFRRQQQEDRERLRAMEKLLASVEERQRHLAGLAVGRRPPQECCSPELGR

RLAELERRLDVVAGSVTVLSGRRGTELGGAAGQGGHPPGYTSLASRLSRLEDRF**N**STLGP

SEEQEESWPGAPGGLSHWLPAARGRLEQLGGLLA**N**VSGELGGRLDLLEEQVAGAMQACGQ

LCSGAPGEQDSQVSEILSALERRVLDSEGQLRLVGSGLHTVEAAGEARQATLEGLQEVVG

RLQDRVDAQDETAAEFTLRLNLTAARLGQLEGLLQAHGDEGCGACGGVQEELGRLRDGVE

RCSCPLLPPRGPGAGPGVGGPSRGPLDGFSVFGGSSGSALQALQGELSEVILSFSSLNDS

LNELQTTVEGQGADLADLGATKDRIISEINRLQQEATEHATESEERFRGLEEGQAQAGQC

PSLEGRLGRLEGVCERLDTVAGGLQGLREGLSRHVAGLWAGLRET**N**TTSQMQAALLEKLV

GGQAGLGRRLGAL**N**SSLQLLEDRLHQLSLKDLTGPAGEAGPPGPPGLQGPPGPAGPPGSP

GKDGQEGPIGPPGPQGEQGVEGAPAAPVPQVAFSAALSLPRSEPGTVPFDRVLLNDGGYY

DPETGVFTAPLAGRYLLSAVLTGHRHEKVEAVLSRSNQGVARVDSGGYEPEGLENKPVAE

SQPSPGTLGVFSLILPLQAGDTVCVDLVMGQLAHSEEPLTIFSGALLYGDPELEHA

P04156

MANLGCWMLVLFVATWSDLGLCKKRPKPGGWNTGGSRYPGQGSPGGNRYPPQGGGGWGQP

HGGGWGQPHGGGWGQPHGGGWGQPHGGGWGQGGGTHSQWNKPSKPKTNMKHMAGAAAAGA

VVGGLGGYMLGSAMSRPIIHFGSDYEDRYYRENMHRYPNQVYYRPMDEYSNQNNFVHDCV

**N**ITIKQHTVTTTTKGE**N**FTETDVKMMERVVEQMCITQYERESQAYYQRGSSMVLFSSPPV

ILLISFLIFLIVG

P09693

MEQGKGLAVLILAIILLQGTLAQSIKGNHLVKVYDYQEDGSVLLTCDAEAK**N**ITWFKDGK

MIGFLTEDKKKWNLGSNAKDPRGMYQCKGSQ**N**KSKPLQVYYRMCQNCIELNAATISGFLF

AEIVSIFVLAVGVYFIAGQDGVRQSRASDKQTLLPNDQLYQPLKDREDDQYSHLQGNQLR

RN

P02788

MKLVFLVLLFLGALGLCLAGRRRSVQWCAVSQPEATKCFQWQRNMRKVRGPPVSCIKRDS

PIQCIQAIAENRADAVTLDGGFIYEAGLAPYKLRPVAAEVYGTERQPRTHYYAVAVVKKG

GSFQLNELQGLKSCHTGLRRTAGWNVPIGTLRPFL**N**WTGPPEPIEAAVARFFSASCVPGA

DKGQFPNLCRLCAGTGENKCAFSSQEPYFSYSGAFKCLRDGAGDVAFIRESTVFEDLSDE

AERDEYELLCPDNTRKPVDKFKDCHLARVPSHAVVARSVNGKEDAIWNLLRQAQEKFGKD

KSPKFQLFGSPSGQKDLLFKDSAIGFSRVPPRIDSGLYLGSGYFTAIQNLRKSEEEVAAR

RARVVWCAVGEQELRKCNQWSGLSEGSVTCSSASTTEDCIALVLKGEADAMSLDGGYVYT

AGKCGLVPVLAENYKSQQSSDPDPNCVDRPVEGYLAVAVVRRSDTSLTWNSVKGKKSCHT

AVDRTAGWNIPMGLLF**N**QTGSCKFDEYFSQSCAPGSDPRSNLCALCIGDEQGENKCVPNS

NERYYGYTGAFRCLAENAGDVAFVKDVTVLQNTDGNNNEAWAKDLKLADFALLCLDGKRK

PVTEARSCHLAMAPNHAVVSRMDKVERLKQVLLHQQAKFGR**N**GSDCPDKFCLFQSETKNL

LFNDNTECLARLHGKTTYEKYLGPQYVAGITNLKKCSTSPLLEACEFLRK

P00736

MWLLYLLVPALFCRAGGSIPIPQKLFGEVTSPLFPKPYPNNFETTTVITVPTGYRVKLVF

QQFDLEPSEGCFYDYVKISADKKSLGRFCGQLGSPLGNPPGKKEFMSQGNKMLLTFHTDF

SNEE**N**GTIMFYKGFLAYYQAVDLDECASRSKSGEEDPQPQCQHLCHNYVGGYFCSCRPGY

ELQEDTHSCQAECSSELYTEASGYISSLEYPRSYPPDLRC**N**YSIRVERGLTLHLKFLEPF

DIDDHQQVHCPYDQLQIYANGKNIGEFCGKQRPPDLDTSSNAVDLLFFTDESGDSRGWKL

RYTTEIIKCPQPKTLDEFTIIQNLQPQYQFRDYFIATCKQGYQLIEGNQVLHSFTAVCQD

DGTWHRAMPRCKIKDCGQPRNLPNGDFRYTTTMGVNTYKARIQYYCHEPYYKMQTRAGSR

ESEQGVYTCTAQGIWKNEQKGEKIPRCLPVCGKPVNPVEQRQRIIGGQKAKMGNFPWQVF

TNIHGRGGGALLGDRWILTAAHTLYPKEHEAQS**N**ASLDVFLGHTNVEELMKLGNHPIRRV

SVHPDYRQDESYNFEGDIALLELENSVTLGPNLLPICLPDNDTFYDLGLMGYVSGFGVME

EKIAHDLRFVRLPVANPQACENWLRGKNRMDVFSQNMFCAGHPSLKQDACQGDSGGVFAV

RDPNTDRWVATGIVSWGIGCSRGYGFYTKVLNYVDWIKKEMEEED

P0DN86

MEMFQGLLLLLLLSMGGTWASKEPLRPRCRPI**N**ATLAVEKEGCPVCITV**N**TTICAGYCPT

MTRVLQGVLPALPQVVCNYRDVRFESIRLPGCPRGVNPVVSYAVALSCQCALCRRSTTDC

GGPKDHPLTCDDPRFQDSSSSKAPPPSLPSPSRLPGPSDTPILPQ

P10163

MLLILLSVALLALSSAESSSEDVSQEESLFLISGKPEGRRPQGGNQPQRPPPPPGKPQGP

PPQGG**N**QSQGPPPPPGKPEGRPPQGGNQSQGPPPHPGKPERPPPQGGNQSQGPPPHPGKP

ESRPPQGGHQSQGPPPTPGKPEGPPPQGGNQSQGTPPPPGKPEGRPPQGGNQSQGPPPHP

GKPERPPPQGGNQSHRPPPPPGKPERPPPQGGNQSQGPPPHPGKPEGPPPQEGNKSRSAR

SPPGKPQGPPQQEGNKPQGPPPPGKPQGPPPAGGNPQQPQAPPAGKPQGPPPPPQGGRPP

RPAQGQQPPQ

P01730

MNRGVPFRHLLLVLQLALLPAATQGKKVVLGKKGDTVELTCTASQKKSIQFHWKNSNQIK

ILGNQGSFLTKGPSKLNDRADSRRSLWDQGNFPLIIKNLKIEDSDTYICEVEDQKEEVQL

LVFGLTANSDTHLLQGQSLTLTLESPPGSSPSVQCRSPRGKNIQGGKTLSVSQLELQDSG

TWTCTVLQNQKKVEFKIDIVVLAFQKASSIVYKKEGEQVEFSFPLAFTVEKLTGSGELWW

QAERASSSKSWITFDLKNKEVSVKRVTQDPKLQMGKKLPLHLTLPQALPQYAGSG**N**LTLA

LEAKTGKLHQEVNLVVMRATQLQK**N**LTCEVWGPTSPKLMLSLKLENKEAKVSKREKAVWV

LNPEAGMWQCLLSDSGQVLLESNIKVLPTWSTPVQPMALIVLGGVAGLLLFIGLGIFFCV

RCRHRRRQAERMSQIKRLLSEKKTCQCPHRFQKTCSPI

P00747

MEHKEVVLLLLLFLKSGQGEPLDDYVNTQGASLFSVTKKQLGAGSIEECAAKCEEDEEFT

CRAFQYHSKEQQCVIMAENRKSSIIIRMRDVVLFEKKVYLSECKTGNGKNYRGTMSKTKN

GITCQKWSSTSPHRPRFSPATHPSEGLEENYCRNPDNDPQGPWCYTTDPEKRYDYCDILE

CEEECMHCSGENYDGKISKTMSGLECQAWDSQSPHAHGYIPSKFPNKNLKKNYCRNPDRE

LRPWCFTTDPNKRWELCDIPRCTTPPPSSGPTYQCLKGTGENYRGNVAVTVSGHTCQHWS

AQTPHTH**N**RTPENFPCKNLDENYCRNPDGKRAPWCHTTNSQVRWEYCKIPSCDSSPVSTE

QLAPTAPPELTPVVQDCYHGDGQSYRGTSSTTTTGKKCQSWSSMTPHRHQKTPENYPNAG

LTMNYCRNPDADKGPWCFTTDPSVRWEYCNLKKCSGTEASVVAPPPVVLLPDVETPSEED

CMFGNGKGYRGKRATTVTGTPCQDWAAQEPHRHSIFTPETNPRAGLEKNYCRNPDGDVGG

PWCYTTNPRKLYDYCDVPQCAAPSFDCGKPQVEPKKCPGRVVGGCVAHPHSWPWQVSLRT

RFGMHFCGGTLISPEWVLTAAHCLEKSPRPSSYKVILGAHQEVNLEPHVQEIEVSRLFLE

PTRKDIALLKLSSPAVITDKVIPACLPSPNYVVADRTECFITGWGETQGTFGAGLLKEAQ

LPVIENKVCNRYEFLNGRVQSTELCAGHLAGGTDSCQGDSGGPLVCFEKDKYILQGVTSW

GLGCARPNKPGVYVRVSRFVTWIEGVMRNN

P00533

MRPSGTAGAALLALLAALCPASRALEEKKVCQGTSNKLTQLGTFEDHFLSLQRMF**N**NCEV

VLGNLEITYVQR**N**YDLSFLKTIQEVAGYVLIALNTVERIPLENLQIIRGNMYYENSYALA

VLSNYDANKTGLKELPMRNLQEILHGAVRFSNNPALCNVESIQWRDIVSSDFLSNMSMDF

QNHLGSCQKCDPSCPNGSCWGAGEENCQKLTKIICAQQCSGRCRGKSPSDCCHNQCAAGC

TGPRESDCLVCRKFRDEATCKDTCPPLMLYNPTTYQMDVNPEGKYSFGATCVKKCPRNYV

VTDHGSCVRACGADSYEMEEDGVRKCKKCEGPCRKVCNGIGIGEFKDSLSINATNIKHFK

NCTSISGDLHILPVAFRGDSFTHTPPLDPQELDILKTVKEITGFLLIQAWPENRTDLHAF

ENLEIIRGRTKQHGQFSLAVVSLNITSLGLRSLKEISDGDVIISGNKNLCYANTINWKKL

FGTSGQKTKIISNRGENSCKATGQVCHALCSPEGCWGPEPRDCVSCRNVSRGRECVDKCN

LLEGEPREFVENSECIQCHPECLPQAMNITCTGRGPDNCIQCAHYIDGPHCVKTCPAGVM

GENNTLVWKYADAGHVCHLCHPNCTYGCTGPGLEGCPTNGPKIPSIATGMVGALLLLLVV

ALGIGLFMRRRHIVRKRTLRRLLQERELVEPLTPSGEAPNQALLRILKETEFKKIKVLGS

GAFGTVYKGLWIPEGEKVKIPVAIKELREATSPKANKEILDEAYVMASVDNPHVCRLLGI

CLTSTVQLITQLMPFGCLLDYVREHKDNIGSQYLLNWCVQIAKGMNYLEDRRLVHRDLAA

RNVLVKTPQHVKITDFGLAKLLGAEEKEYHAEGGKVPIKWMALESILHRIYTHQSDVWSY

GVTVWELMTFGSKPYDGIPASEISSILEKGERLPQPPICTIDVYMIMVKCWMIDADSRPK

FRELIIEFSKMARDPQRYLVIQGDERMHLPSPTDSNFYRALMDEEDMDDVVDADEYLIPQ

QGFFSSPSTSRTPLLSSLSATSNNSTVACIDRNGLQSCPIKEDSFLQRYSSDPTGALTED

SIDDTFLPVPEYINQSVPKRPAGSVQNPVYHNQPLNPAPSRDPHYQDPHSTAVGNPEYLN

TVQPTCVNSTFDSPAHWAQKGSHQISLDNPDYQQDFFPKEAKPNGIFKGSTAENAEYLRV

APQSSEFIGA

P24071

MDPKQTTLLCLVLCLGQRIQAQEGDFPMPFISAKSSPVIPLDGSVKIQCQAIREAYLTQL

MIIKNSTYREIGRRLKFWNETDPEFVIDHMDANKAGRYQCQYRIGHYRFRYSDTLELVVT

GLYGKPFLSADRGLVLMPGE**N**ISLTCSSAHIPFDRFSLAKEGELSLPQHQSGEHPA**N**FSL

GPVDLNVSGIYRCYGWYNRSPYLWSFPSNALELVVTDSIHQDYTTQNLIRMAVAGLVLVA

LLAILVENWHSHTALNKEASADVAEPSWSQQMCQPGLTFARTPSVCK

P27797

MLLSVPLLLGLLGLAVAEPAVYFKEQFLDGDGWTSRWIESKHKSDFGKFVLSSGKFYGDE

EKDKGLQTSQDARFYALSASFEPFSNKGQTLVVQFTVKHEQNIDCGGGYVKLFPNSLDQT

DMHGDSEYNIMFGPDICGPGTKKVHVIFNYKGKNVLINKDIRCKDDEFTHLYTLIVRPDN

TYEVKIDNSQVESGSLEDDWDFLPPKKIKDPDASKPEDWDERAKIDDPTDSKPEDWDKPE

HIPDPDAKKPEDWDEEMDGEWEPPVIQNPEYKGEWKPRQIDNPDYKGTWIHPEIDNPEYS

PDPSIYAYDNFGVLGLDLWQVKSGTIFDNFLITNDEAYAEEFG**N**ETWGVTKAAEKQMKDK

QDEEQRLKEEEEDKKRKEEEEAEDKEDDEDKDEDEEDEEDKEEDEEEDVPGQAKDEL

32 6 31 84%
