# Supplementary material for: Mutually exclusive locales for N-linked glycans and disorder in human glycoproteins
Source: Sci Rep. 2020 Apr 8;10:6040. doi: 10.1038/s41598-020-61427-y (PMC7142085; doi:10.1038/s41598-020-61427-y)
Supplement: Supplementary file 4 — Supplementary File 6. [file 41598_2020_61427_MOESM4_ESM.docx]

**KEY:**

YELLOW = Disordered Region

**N** = Glycosylation

BLUE = Glycosylated Region (5 residues on both sides)

1. **Apolipoprotein C-IV**

gal MPAVSKGDGMRGLAVFISDIRNCKSKEAEIKRINKELANIRSKFKGDKALDGYSKKKYVC

mus ------------------------------------------------------------

hum ------------------------------------------------------------

pan ------------------------------------------------------------

gal KLLFIFLLGHDIDFGHMEAVNLLSSNRYTEKQIGYLFISVLVNSNSELIRLINNAIKNDL

mus ------------------------------------------------------------

hum ------------------------------------------------------------

pan ------------------------------------------------------------

gal ASRNPTFMGLALHCIANVCSREMAEAFAGEIPKILVAGDTMDSVKQSAALCLLRLYRTSP

mus ----------------------------------------------------MSLLRCRP

hum ----------------------------------------------------MSLLRNRL

pan ----------------------------------------------------MSLLRNRL

: * *

gal DLVPMGDWTSRVVHLLNDQHLGVVTAATSLITTLAQKNPEEFKTSVSLAVSRLSRIVTSA

mus RDL---------------------------------------------------------

hum QAL---------------------------------------------------------

pan QAL---------------------------------------------------------

:

gal STDLQDYTYYFVPAPWLSVKLL-RLLQC---------YPPPD------PAVRGRLTECLE

mus ------------PSVSLSVLFLVSFVA---SMSTESLSPTPGPESSRWSLVRARVLEMVE

hum ------------PALCLCVLVLACIGACQPEAQEGTLSPPPKLKMSRWSLVRGRMKELLE

pan ------------PALCLCVLVLACIGACQPEAQEGTPSTPPKLKMSRWSLVRGRMKELLE

*: *.* .* : * **.*: * :*

gal TILNKAQEPPKSKKVQHSNAKNAVLFEAISLIIHHDSEPNLLVRACNQLGQFLQHRETNL

mus PLVTRTR-----------------------------------------------------

hum TVV**N**RTR-----------------------------------------------------

pan PVVNRTR-----------------------------------------------------

::.:::

gal RYLALESMCTLASSEFSHEAVKTHIETVINALKTERDVSVRQRAVDLLYAMCDRSNAQQI

mus ------------------------------------------------------------

hum ------------------------------------------------------------

pan ------------------------------------------------------------

gal VAEMLNYLETADYSIREEIVLKVAILAEKYAVDYTWYVDTILNLIRIAGDYVSEEVWYRV

mus ------------------------------------------------------------

hum ------------------------------------------------------------

pan ------------------------------------------------------------

gal IQIVINRDDVQGYAAKTVFEALQAPACHENLVKVGGYILGEFGNLIAGDPRSSPLIQFNL

mus ------------------------------------------------------------

hum ------------------------------------------------------------

pan ------------------------------------------------------------

gal LHSKFHLCSVPTRALLLSTYIKFVNLFPEIKTTIQDVLRSDSQLKNADVELQQRAVEYLR

mus ------------------------------------------------------------

hum ------------------------------------------------------------

pan ------------------------------------------------------------

gal LSTIASTDILATVLEEMPPFPERESSILAKLKKKKGPGTVTDLEEIKKERSSDMNGSAEP

mus ------------------------------------------------------------

hum ------------------------------------------------------------

pan ------------------------------------------------------------

gal ASVNASAVSTPSPSADLLGLGAAPLTNSAPPPSSSGSLLVDVFSDSASAVAPLAPGSDDN

mus ----------------------------------------------------------DR

hum ----------------------------------------------------------DG

pan ----------------------------------------------------------DG

*

gal FARFVCKNN-----GVLFENQLLQIGLKSEFRQNLGRMFIFYGNKTSTQFLNFTPTVICS

mus WQWFWGPGAVQGFMQTYYEDHLKDLGPRTQA------WLQSSRDHLLNKTHSLCPRLLCK

hum WQWFWSPSTFRGFMQTYYDDHLRDLGPLTKA------WFLESKDSLLKKTHSLCPRLVCG

pan WQWFWSPSTFWGFMQTYYDDHLRDLGPRTKA------WLLESKDSLLKKTHSLCPRLVCG

: * . . ::::* ::* :: : : .: .: * ::*

gal DDLQPSLNLQTKPVDPTVDGGAQVQQVVNIECVSDFMEAPILNIQFRYGGTFQNLSVKLP

mus DRTQG-------------------------------------------------------

hum DKDQG-------------------------------------------------------

pan DKDQG-------------------------------------------------------

* *

gal ITLNKFFQPTEMSSQDFFQRWKQLSNPKQEVQNIFKAKHPMDAEITKAKIIGFGSALLEE

mus ------------------------------------------------------------

hum ------------------------------------------------------------

pan ------------------------------------------------------------

gal VDPNPANFVGAGIIHTKTTQIGCLLRLEPNLQAQMYRLTLRTSKEAVSQRLCELLSEQF

mus -----------------------------------------------------------

hum -----------------------------------------------------------

pan -----------------------------------------------------------

1. **Lutropin subunit beta**

gal MGGAQVLVLMTLLGTPPATTGNPPVAVDPPLAVVGPPMGLGGGGRPPCRPINVTVAVEKD

mus MEMLQGLLLWLLLS-------------------MGGARASREPLRPLCRPINATLAAEKE

hum MEMLQGLLLLLLLS-------------------MGGAWASREPLRPWCHPINAILAVEKE

pan MEMLQGLLLLLLLS-------------------MGGAWASREPLRPWCHPINATLAVEKE

* * *:* **. :* . ** *:***. :*.**:

gal GCPQCMAVTTTACGGYCRTREPVYRSPLGPPPQSACTYGALRYERWALWGCPIGSDPRVL

mus ACPVCITVNTTICAGYCPTMMRVLQAVLPPVPQVVRNYREVRFESIRLPGCPPGVDPVVS

hum GCPVCITV**N**TTICAGYCPTMMRVLQAVLPPLPQVVCTYRDVRFESIRLPGCPRGVDPVVS

pan GCPVCITVNTTICAGYCPTMMRVLQAVLPPLPQVVCTYRDVRFESIRLPGCPRGVDPVVS

.** *::*.** *.*** * * :: * * ** . .* :*:* * *** * ** *

gal LPVALSCRCARCPMATSDCTVQGLGPAFCGAPGGFGGE----------------------

mus VPVALSCRCALCRRSTSDCGGPKDHPLTCDDPHLQASSSSKDPPPSPPSPSGLLEPADNP

hum FPVALSCRCGPCRRSTSDCGGPKDHPLTCDHPQLSGLLFL--------------------

pan FPVALSCRCGPCRRSTSDCGGPKDHPLTCDHPQLSGLLFL--------------------

.********. * :**** * *. * .

gal ----

mus FLPQ

hum ----

pan ----

1. **Cystatin-8**

gal MAAAACSAVCGAMAGARG-CVVLLAAALMLVGAVLGSEDRSRLLGAPVPVDENDEGLQRA

mus ------------MAKPLWLSLILFIIPVALAVGVDQSKNEVKAQNYFGSINISNANVKQC

hum ------------MPRCRWLSLILLTIPLALVARKDPKK**N**ETGVLRKLKPV**N**ASNANVKQC

pan ------------MPRCRWLSLILLTIPLALVARKDPKKNETGVLRKLKPVNASNANVKQC

* .::*: : *. .::. :: .: .:::.

gal LQFAMAEYNRASNDKYSSRVVRVISAKRQLVSGIKYILQVEIGRTTCPKSSGDLQSCEFH

mus VWFAMKEYNKESEDKYVFLVDKILHAKLQITDRMEYQIDVQISRSNCKKPLNNTENCIPQ

hum LWFAMQEYNKESEDKYVFLVVKTLQAQLQVTNLLEYLIDVEIARSDCRKPLSTNEICAIQ

pan LWFAMQEYNKESEDKYVFLVVKTLQAQLQVTNLLEYLIDVEIARSDCRKPLSTNEICAIQ

: *** ***: *:*** * : : *: *:.. ::* ::*:*.*: * * . : * :

gal DEPEMAKYTTCTFVVYSIPWLNQIKLLESKCQ--

mus KKPELEKKMSCSFLVGALPWNGEFNLLSKECKDV

hum ENSKLKRKLSCSFLVGALPWNGEFTVMEKKCEDA

pan ENSKLKKKLSCSFLVGALPWNGEFTVMEKKCEDA

.: :: : :*:*:* ::** .::.::..:*:

1. **Transthyretin**

gal MAFHSTLLVFLAGLVFLSEAAPLVSHGSVDSKCPLMVKVLDAVRGSPAANVAVKVFKKAA

hum MASHRLLLLCLAGLVFVSEAGPT---GTGESKCPLMVKVLDAVRGSPAINVAVHVFRKAA

pan MASHRLLLLCLAGLVFVSEAGPT---GTGESKCPLMVKVLDAVRGSPAINVAVHVFKKAA

mus MASLRLFLLCLAGLVFVSEAGPA---GAGESKCPLMVKVLDAVRGSPAVDVAVKVFKKTS

** :*: ******:***.* *: :****************** :***:**:*::

gal DGTWQDFATGKTTEFGEIHELTTEEQFVEGVYRVEFDTSSYWKGLGLSPFHEYADVVFTA

hum DDTWEPFASGKTSESGELHGLTTEEEFVEGIYKVEIDTKSYWKALGISPFHEHAEVVFTA

pan DETWEPFASGKTSESGELHGLTTEEEFVEGIYKVEIDTKSYWKALGISPFHEHAEVVFTA

mus EGSWEPFASGKTAESGELHGLTTDEKFVEGVYRVELDTKSYWKTLGISPFHEFADVVFTA

: :*: **:***:* **:* ***:*:****:*:**:**.**** **:*****.*:*****

gal NDSGHRHYTIAALLSPFSYSTTAVVSDPQE

hum **N**DSGPRRYTIAALLSPYSYSTTAVVTNPKE

pan NDSGPRRYTIAALLSPYSYSTTAVVTIPKE

mus NDSGHRHYTIAALLSPYSYSTTAVVSNPQN

**** *:*********:********: *::

1. **Epididymal Secretory protein E1**

gal -MVPPPLVLLLALAAAALAEPLRFVDCGSKDGSIQEVNVSPCPTQPCQLVKGTSYSINVT

hum MRFLAATFLLLALSTAAQAEPVQFKDCGSVDGVIKEVNVSPCPTQPCQLSKGQSYSV**N**VT

pan MRFLAATFLLLALSTAAQAEPVQFKDCGSVDGVIKEVNVSPCPTQPCQLSKGQSYSVNVT

mus MRFLAATILLLALVAASQAEPLHFKDCGSKVGVIKEVNVSPCPTDPCQLHKGQSYSVNIT

. .***** :*: ***::* **** * *:*********:**** ** ***:*:*

gal FSSKIESQGSKAKVYGEMLHVDIPFPIPEPDGCKSGIQCPIQKGHSYSYLNKLPVKSEYP

hum FTSNIQSKSSKAVVHGILMGVPVPFPIPEPDGCKSGINCPIQKDKTYSYLNKLPVKSEYP

pan FTSNIQSKSSKAVVHGILMGVPVPFPIPEPDGCKSGINCPIQKDKTYSYLNKLPVKSEYP

mus FTSGTQSQNSTALVHGILEGIRVPFPIPEPDGCKSGINCPIQKDKVYSYLNKLPVKNEYP

*:* :*:.*.* *:* : : :**************:*****.: **********.***

gal SIKLIVKWELVDDQDQMLFCWKIPVQITS--

hum SIKLVVEWQLQDDK**N**QSLFCWEIPVQIVSHL

pan SIKLVVEWQLQDDKNQSLFCWEIPVQIVSHL

mus SIKLVVEWKLEDDKKNNLFCWEIPVQITS--

****:*:*:* **:.: ****:*****.*

1. **Interleukin-4**

gal MSSSLPTLLALLVLLAGPGAVPMLCLQLSVPLMESIRIVNDIQGE-VSCVKMNVTDIFAD

hum MGLTSQLLPPLFFLLACAGNFVH-GHKCDITLQEIIKTLNSLTEQKTLCTELTVTDIFAA

pan MGLTSQLLPPLFFLLACAGNFVH-GHKCDITLQEIIKTLNSLTEQKTLCTELTVTDIFAA

mus MGLNPQLVVILLFFLECTRSHIH-GCDK-NHLREIIGILNEVTGEGTPCXEMDVPNVLTA

*. . : *:.:* . * * * :*.: : . * :: * ::::

gal NKTNNKTELLCKASTIVWESQH-------CHKNLQGLFLNMRQLL--------NASSTSL

hum SK**N**TTEKETFCRAATVLRQFYSHHEKDTRCLGATAQQFHRHKQLIRFLKRLDRNLWGLAG

pan SKNTTEKETFCRAATVLRQFYSHHEKDTRCLGATAQQFHRHKQLIRFLKRLDRNLWGLAG

mus TKNTTESELVCRASKVLRIFYLKHGKT-PCLKKNSSV-------LMELQRLFRAFRCLDS

.*...:.* .*:*:.:: * :

gal KAPCPTAAGNTTSMEKFLADLRTFFHQLAKNK--

hum LNSCPVKEANQSTLENFLERLKTIMREKYSKCSS

pan LNSCPVKEANQSTLENFLERLKTIMREKYSKCSS

mus SISCTMNESKSXSLKDFLESLKSIMQMDYS----

* .: :::.** *::::: .

1. **Pancreatic ribonuclease**

gal ----------------------------------------------MTMSPCPLLLVFVL

mus MCKEAEVGESSVETEVGAAALLSICPFFLFLSFQAFPSSQATMGLEKSL---ILFPLFVL

hum ------------------------------------------MALEKSLVRLLLLVLILL

pan ------------------------------------------MALEKSLVLLPLLVLILL

:: *: :::*

gal GLVVIPPTLAQNEGYEKFLRQHYDAKPK-GRDDRYCESMMKERKLT-SPCKDVNTFIHGT

mus LLGWVQPSLGRESAAQKFQRQHMDPDGSSINSPTYCNQMMKRRDMTNGSCKPVNTFVHEP

hum VLGWVQPSLGKESRAKKFQRQHMDSDSSPSSSSTYCNQMMRRR**N**MTQGRCKPVNTFVHEP

pan VLGWVQPSLGKESRAKKFQRQHVDSDSSPSSSSTYCNQMMRRRNMTQGRCKPVNTFVHEP

* : *:*.::. :** *** * . . . **:.**:.*.:* . ** ****:*

gal KKNIRAICGKKGSPY---GENFRISNSPFQITTCTHSGASPRPPCGYRAFKDFRYIVIAC

mus LADVQAVCSQENVTCKNRKSNCYKSSSALHITDCHLKGNSKYPNCDYKTTQYQKHIIVAC

hum LVDVQNVCFQEKVTCKNGQGNCYKS**N**SSMHITDCRLT**N**GSRYPNCAYRTSPKERHIIVAC

pan LVDVQNVCFQEKVTCKNGQGNCYKSNSSMHITDCRLTNGSRYPNCAYRTSPKERHIIVAC

::: :* :: * *.* ::** * .. * * * *:: ::*::**

gal EDG--WPVHFDESFISP-

mus EGNPYVPVHFDATV----

hum EGSPYVPVHFDASVEDST

pan EGSPYVPVHFDASVEDST

*.. ***** :.

1. **Interleukin-17F**

gal MAF--ASCAAVFRSLLLVLVLALTVWSSPHGKVVRPRPRKDGGSEKLSEDCLNQKDPSFP

mus ----------MVKS-LLLLMLGLAILREVA---ARKNPKAGVPALQKAGNCP----PLED

hum MTVKTLHGPAMVKY-LLLSILGLAFLSEAA---ARKIPKVGHTFFQKPESCP----PVPG

pan MTVKTLHGPAMVKY-LLLLILGLAFLSEAA---ARKIPKVGHTFFQKPESCP----PVPG

:.: **: :*.*:. . .* *: . : .* *

gal TMVKVDIRIGSSDPASRMIHDIRNRSLAPWNYRLDEDPNRFPQVIADAECRLLGCLNSLG

mus NTVRVDIRIFNQNQGISVPREFQNRSSSPWDYNITRDPHRFPSEIAEAQCRHSGCINAQG

hum GSMKLDIGIINENQRVSMSRNIESRSTSPW**N**YTVTWDPNRYPSEVVQAQCRNLGCINAQG

pan DSMKLDIGIINENQRVSMSRNIESRSTSPWNYTVTRDPNRYPSEVVQAQCRNLGCINAQG

:::** * ..: : :::..** :**:* : **:*:*. :.:*:** **:*: *

gal QEDRSLNSVPITQEILVLRREQRGCQPTYHLEKKLITVGCTCAAPVIQHQS

mus QEDSTMNSVAIQQEILVLRREPQGCSNSFRLEKMLLKVGCTCVKPIVHQAA

hum KEDISMNSVPIQQETLVVRRKHQGCSVSFQLEKVLVTVGCTCVTPVIHHVQ

pan KEDISMNSVPIQQETLVVRRKHQGCSVSFQLEKVLVTVGCTCVTPVIHHVQ

:** ::*** * ** **:**: :**. :::*** *:.*****. *::::

1. **Choriogonadotropin subunit beta**

gal MGGAQVLVLMTLLGTPPATTGNPPVAVDPPLAVVGPPMGLGGGGRPPCRPINVTVAVEKD

mus MEMLQGLLLWLLL-------------------SMGGARASREPLRPLCRPINATLAAEKE

hum MEMFQGLLLLLLL-------------------SMGGTWASKEPLRPRCRPI**N**ATLAVEKE

pan MEMFQGLMLLLLL-------------------SMGGTWATKEPLRPRCRPINATLAVEKE

* * *:* ** :* . ** *****.*:*.**:

gal GCPQCMAVTTTACGGYCRTREPVYRSPLGPPPQSACTYGALRYERWALWGCPIGSDPRVL

mus ACPVCITVNTTICAGYCPTMMRVLQAVLPPVPQVVRNYREVRFESIRLPGCPPGVDPVVS

hum GCPVCITV**N**TTICAGYCPTMTRVLQGVLPALPQVVCNYRDVRFESIRLPGCPRGVNPVVS

pan GCPVCITVNTTICAGYCPTMTRVLQGVLPALPQVVCNYRDVRFESIRLPGCPRGVNPVVS

.** *::*.** *.*** * * :. * ** . .* :*:* * *** * :* *

gal LPVALSCRCARCPMATSDCTVQGLGPAFCGAPGGFGGE----------------------

mus VPVALSCRCALCRRSTSDCGGPKDHPLTCDDPHLQASSSSKDPPPSPPSPSGLLEPADNP

hum YAVALSCQCALCRRSTTDCGGPKDHPLTCDDPRFQDSSSSKAPPPSLPSPSRLPGPSDTP

pan YAVALSCQCALCRRSTTDCGGPKDHPLTCDDPRFQASSSSKAPPPSLPSPSRLPGPSDTP

*****:** * :*:** * *. * ..

gal ----

mus FLPQ

hum ILPQ

pan ILPQ

1. **Interferon-gamma**

gal MTCQTY----NLFVLSVIMIYYGHTASSLNLVQLQDDIDKLKADFNSSHSDVADGGPIIV

hum MKYTSYILAFQLCIVLGSLGCYC-------QDPYVKEAENLKKYFNAGHSDVAD**N**GTLFL

pan MKYTSYILAFQLCIVLGSLGCYC-------QDPYVKEAENLKKYFNAGHSDVADNGTLFL

mus MNATHCILALQLF-LMAVSGCYC-------HGTVIESLESLNNYFNSSGIDVE-EKSLFL

*. :* : * .. :.*: **:. ** :::

gal EKLKNWTERNEKRIILSQIVSMYLEMLENTDKSKPHIKHISEELYTL-KNNLPDGVKKVK

hum GILKNWKEESDRKIMQSQIVSFYFKLFKNFKDDQSIQKSVETIKEDMNVKFFNSNKKKRD

pan GILKNWKEESDRKIMQSQIVSFYFKLFKNFKDDQSIQKSVETIKEDMNVKFFNSNKKKRD

mus DIWRNWQKDGDMKILQSQIISFYLRLFEVLKDNQAISNNISVIESHLITTFFSNSKAKKD

:** : .: :*: ***:*:*:.::: ...: : :. : . : .. * .

gal DIMDLAKLPMSDLRIQRKAANELFSILQKLVDPPSFK-RKRSQSQRRCNC---

hum DFEKLT**N**YSVTDLNVQRKAIHELIQVMAELSPAAKTGKRKRSQMLFRGRRASQ

pan DFEKLTNYSVTDLNVQRKAIHELIQVMAELSPAVKTGKRKRSQMLFRGRRASQ

mus AFMSIAKFEVNNPQVQRQAFNELIRVVHQLSPESSLRKRKRSRC---------

: .::: :.: .:**:* :**: :: :* . ****:

1. **Microfibrillar associated protein 5**

gal MKTSATFMALYLLAFSLSPAGWFPWVVNGQELESVTGDVHTSL-----LISTPV------

mus MLFLGQKA-LLLVLAVSIPSDWLPLGVSGQRGDDV----PETFTDDPNLVNDPSTDDTAL

hum MSLLGPKV-LLFLAAFIITSDWIPLGVNSQRGDDVTQATPETFTEDPNLVNDPATDETVL

pan MSLLGPKV-LLFLAAFIITSDWIPLGVNSQRGDDVTQTTPETFTEDPNLVNDPATDETVL

* . * :: :.*:* *..*. :.* :: *:. *

gal ------------QSDTETTTASSDCREEQFPCTRLYSVHKPVKQCISYLCVTSVRRMYVI

mus ---ADITPSTDDLA--DDKNATAECRDEKFACTRLYSVHRPVRQCVHQSCFTSLRRMYII

hum AVLADIAPSTDDLASLSEK**N**TTAECWDEKFTCTRLYSVHRPVKQCIHQLCFTSLRRMYIV

pan AVLADIAPSTDDLASLSEKNTTAECWDEKFTCTRLYSVHRPVKQCIHQLCFTSSRRMYIV

: . ..::::* :*:* ********:**:**: *.** ****::

gal NKEVCSRIVCKENEVMQDEICRQLAGLPSRRLRRSGQLSHLPCKQILEQQHRRPDAL

mus NNEICSRLVCKEHEAMKDELCRQMAGLPPRRLRRSNYFRLPPCENMNLQ---RPDGL

hum NKEICSRLVCKEHEAMKDELCRQMAGLPPRRLRRSNYFRLPPCENVDLQ---RPNGL

pan NKEICSRLVCKEHEAMKDELCRQMAGLPPRRLRRSNYFRLPPCENVDLQ---RPNGL

*:*:***:****:*.*:**:***:**** ******. : **::: * **:.*

1. **protein MAL2**

hum MSAGGASVPPPPNPAVSFPP-------PRVTLPAGPDILRTYSGAFVCLEILFGGLVWIL

pan -----M---INPE---LRDGRADG-----FIHRIVPKLIQNWKIGLICFLS----II---

gal -----------MEEVREYPH----VLGTEWSRREGPSRGACVTFQLTMAAV----FTVLL

mus MNAQCL---KKPEEGESSPGTGDKILQRNSLRAISPESSAKLY---CCCGV----IMVLT

: *. :

hum VASSN-----------------VPLPLLQGWVMFVSVTAFFFSLLFLGMFLSGMVAQIDA

pan ITTVCIIM--I---ATWS-KHAKPVACSGDWLGVRD-KCFYFS-------------DDTR

gal ITAVAFAV------QAFQPHPQPCAQCPFDWIGFRG-KCYYFS-------------EDES

mus VAVVALSVALPATKTEQILINKTYAACPKNWIGVGN-KCFYFS-------------EYTS

:: .*: . . ..::** :

hum NWNFLDFAYHFT---VFVFY---FGAFLLEAAATSLHDLHC**N**TTITGQPLLSDN-----Q

pan NWTASKIFCSLQKAELAQIDTQEDMEFLKRYAGTDMHWIGLSRKQ-GDSWKWTNGTTFND

gal NWTSSQNNCSALGASLAVFDSAEDLSFTMRHKGSSPHWVGLSREGKEHAWEWVNRSPLSH

mus NWTFAQTFCMAQEAQLARFDNEKELNFLKRHMN-SSHWIGLHRDSSEHPWRWTDNTEYNN

**. . : : * . . * : . : .

hum YNINVAASIFAFMTTACYGCSLGLALRRWRP----------------

pan WFEIIGNGSFAFLSADGVHSSRGFIDIKWICSKPKYFL---------

gal LFQVQGDGLCAYLGDAGLSSSHCSARRNWVCTKPALQKPRKNFCIST

mus TFLIQGDGECGFLSDNGISSSRDYIPRKWICSRSSNYMLQC------

. . .:: .* .*

1. **IL-1 receptor antagonist**

gal ---------------MGEAAGSVPCRAP-ALQTKVFKYRIWDMNQQSLYLRDDQLVAGHL

mus ------------------MASEAACRPSGKRPCKMQAFRIWDTNQKTFYLRNNQLIAGYL

hum MEICRGLRSHLITLLLFLFHSETICRPSGRKSSKMQAFRIWDVNQKTFYLRNNQLVAGYL

pan MEICRGLRSHLITLLLFLFHSETVCRPSGRKSSKMQAFRIWDVNQKTFYLRNNQLVAGYL

... ** *: :**** **:::***::**:**:*

gal QGANAALEEKVFWVPNRFFKHELQPVIMGIRNGTRCLACPAAP-QPTLQLQDADITELPR

mus QGPNIKLEEKIDMVPI-----DLHSVFLGIHGGKLCLSCAKSGDDIKLQLEEVNITDLSK

hum QGPNVNLEEKIDVVPI-----EPHALFLGIHGGKMCLSCVKSGDETRLQLEAV**N**ITDLSE

pan QGPNVNLEEKIDVVPI-----EPHALFLGIHGGKMCLSCVKSGDETRLQLEAVNITDLSE

** * ****: ** : : :::**:.*. **:* : : ***: .:**:* .

gal SGAASAPFTFFRTYKDGLWRFESAANPGWFLCTSARAHQPLGLSRRPDAAHV-LDFYFQL

mus NKEEDKRFTFIRSEKGPTTSFESAACPGWFLCTTLEADRPVSLTNTPEEPLIVTKFYFQE

hum NRKQDKRFAFIRSDSGPTTSFESAACPGWFLCTAMEADQPVSLTNMPDEGVMVTKFYFQE

pan NRKQDKRFTFIRSDSGPTTSFESAACPGWFLCTAMEADQPVSLTNMPDEGVMVTKFYFQE

. . *:*:*: .. ***** *******: .*.:*:.*:. *: : .****

gal C-

mus DQ

hum DE

pan DE

1. **Bone marrow stromal antigen 2**

gal MCSAVQGAPGSGRARDLMAEPEPPPEPLPDEAGAAPAPHPGAPCSPIDPAPGLGAVPAPG

mus ------------------------------------------------------------

hum ------------------------------------------------------------

pan ------------------------------------------------------------

gal ACGELQEGVGDAALNLTMPGTLLGKETARAEAPLGVDGAETPLDSEADEQILLDVDAGGT

mus ------------------------------------------------------------

hum ------------------------------------------------------------

pan ------------------------------------------------------------

gal GILLDVDGAGIPLDVEDEVGIPLDVDAGGTGALLDADGAGLPLDVEAEGLGILLDMDAAV

mus ------------------------------------------------------------

hum ------------------------------------------------------------

pan ------------------------------------------------------------

gal AGLLQDMDASGIPLHVDGAEIPLDAVRAGIPLAMDTDGAGIPLGAAGVGVPQDMDAEGAG

mus ------------------------------------------------------------

hum ------------------------------------------------------------

pan ------------------------------------------------------------

gal TPQAPEGTERPCLSLEELGEYFQECIEAVEQLEKERDALIEELTQLREPALQDIRHAHQE

mus ----------------MAPSFYHYLPVPMDEMGGK------------------------Q

hum ----------------MASTSYDYCRVPME-----------------------------D

pan ----------------MASTLYDYCRVPMDDIWKK------------------------D

:. :: :

gal IQAACRLLAKVELERDNLRDEIRQIKQKLFKVTKECVACQYQLESRRHDLSQ-HAAYRDE

mus GWGSHRQWLGAAILV-VLFGVTLVILTIYFAVTANSVACRDGLRAQAECRNTTHLLQRQL

hum GDKRCKLLLGIGILV-LLIIVILGVPLIIFTIKANSEACRDGLRAVMECR**N**VTHLLQQEL

pan GDKRCKLLLGIGILM-LLIIVILGVPLIIFTIKANSEACRDGLRAVMECRNVTHLLQQEL

: : * : * :. :. **: *.: . . * ::

gal LESQAGRLTGELSRLRESCEKEKEALRQRLEAPPCRQDPQYLQESRRLSAEFESLVTRSR

mus TRTQDS------------------LLQAETQANSCNLTVVTLQE---------SLEKKVS

hum TEAQKG------------------FQDVEAQAATC**N**HTVMALMA---------SLDAEKA

pan TEAQKG------------------FQDVEAQAATCNHTVMALMA---------SLDAEKA

.:* . . :* *. * ** .

gal RGLEEHYEPQLLRLLERREAGTRALQELQGEVQGMKEALRPLQGEVSRLRLQNRSLEEQI

mus QALE----------------QQARIKELENEVTKLNQELENLRIQKETSS----T--VQV

hum QG-------------------QKKVEELEGEITTLNHKLQDASAEVERLRRENQVLSVRI

pan QG-------------------QKKVEELEEEITTLNHKLQDASAEVERLRRENQVLSVRI

:. ::**: *: ::. *. : . ::

gal VLVKQKRDEEVGQYREQVEELEDRLKELKNSVQLQQRKNQELEELRSSLHHEL-SIYKGC

mus ---------NSGS---------------------------------SMVVSSLLVLKVSL

hum ADKKYYPSSQDSS---------------------------------SAAAPQLLIVLLGL

pan ADKKYYSSSQDSS---------------------------------SAAAPQLLIVLLGL

: .. * .* : .

gal LEIYGHLCKSEEKPDQEC

mus FLLF--------------

hum SALLQ-------------

pan SALLQ-------------

:

1. **Glycodelin**

hum MLCLLLTLGVALVCGVPAMDIPQTKQDLELPKLAGTWHSMAMATN**N**ISLMATLKAPLRVH

pan MQCLLLTLGVALVCGVPAMDIPQTKQDLELPK----------------------APLRVH

mus MKSLL--LTILLLGLVAVLK-AQEAPPDDLEDFSGIWYTQAMVSDRNHTD-GKR-PMKVF

gal MRTSALSLALALLCLL--HT-EAAATVPDSSEVAGKWYIVALASNTDSFL-REKGKMKMV

* * : *: : : . :::

hum ITSLLPTPEDNLEIVLHRWE**N**NSCVEK-KVLGEKTENPKKFKINYTVANEATLLDTDYDN

pan ITSLWPTPEDNLEIVLHRWENNSCVEK-KVLGEKTENPKKFKINYTVANEATLLDTDYDN

mus PMTVIALEGGSLEAQLTFWDNGHCHMK-KILMHKTDEPHKYT-AFKGKKTIYIQETSVKG

gal MARISFLGEDELEVSYAAPSPKGCRKWETTFKKTSDDGELYY-SEEAEKTVEVLDTDYKS

: ..** . * . : ..::: . : : : :*. ..

hum FLFLCLQDTTTPIQSMMCQYLARVLVEDDEIMQGFIRAFRPLPRHLWYLLD---LKQMEE

pan FLFLCLKDTTTPIQSMMCQYLARVLVEDDEIMQRFIRAFRPLPRHLWYLLD---LKQMEE

mus YYILYCEGQRHGRSHRKGKLIG----TNSEKNPEAMEEFKKFAMSKGFREENIIVPEQLD

gal YAVIFATRVKDGRTLHMMRLYS----RSREVSPTAMAIFRKLARERNYTDEMVAVLPSQE

: .: : . . * : *: : : : : :

hum PCRF---

pan LCRF---

mus QCVSGSN

gal ECSVDEV

*

1. **Signal peptidase complex subunit 3**

hum MNTVLSRANSLFAFSLSVMAALTFGCFITTAFKDRSVPVRLHVSRIMLKNVEDFTGPRER

pan MNTVLSRANSLFAFSLSVMAALTFGCFITTAFKDRSVPVRLHVSRIMLKNVEDFTGPRER

mus MNTVLSRANSLFAFSLSVMAALTFGCFITTAFKDRSVPVRLHVSRIMLKNVEDFTGPRER

gal MNTVLSRANSLFAFSLSVMAALTFGCFITTAFKERSVPVSIAVSRVTLRNVEDFTGPRER

*********************************:***** : ***: *:***********

hum SDLGFITFDITADLENIFDWNVKQLFLYLSAEYSTKNNALNQVVLWDKIVLRGDNPKLLL

pan SDLGFITFDITADLENIFDWNVKQLFLYLSAEYSTKNNALNQVVLWDKIVLRGDNPKLLL

mus SDLGFITFDITADLENIFDWNVKQLFLYLSAEYSTKNNALNQVVLWDKIVLRGDNPKLLL

gal SDLAFVTFDITADLQSIFDWNVKQLFLYLSAEYSTKNNALNQVVLWDKIILRGDNPRLFL

***.*:********:.*********************************:******:*:*

hum KDMKTKYFFFDDGNGLKGNR**N**VTLTLSWNVVPNAGILPLVTGSGHVSVPFPDTYEITKSY

pan KDMKTKYFFFDDGNGLKGNRNVTLTLSWNVVPNAGILPLVTGSGHVSVPFPDTYEITKSY

mus KDMKTKYFFFDDGNGLKGNRNVTLTLSWNVVPNAGILPLVTGSGHVSVPFPDTYEITKSY

gal KDMKSKYFFFDDGNGLKGNRNVTLTLSWNVVPNAGLLPLVTGSGHMSVPFPDTYETTKSY

****:******************************:*********:********* ****

1. **Translocon-associated protein subunit beta**

gal -------------------MKLVILAAFALFYVARCEEGARLLASKSLLNRYAVEGKDLT

hum -------------------MRLLSFVVLALFAVTQAEEGARLLASKSLLNRYAVEGRDLT

pan MLTLSSCLCGSGKAFGMPTMRLLSFVVLALFAVTQAEEGARLLASKSLLNRYAVEGRDLT

mus -------------------MRLLAVVVLALLAVSQAEEGARLLASKSLLNRYAVEGRDLT

*:*: ...:**: *::.********************:***

gal LQYNIYNVGSSAALDVELSDDSFPPEDFGIVSGMLNVKWDRIAPASNVSHTVVLRPLKAG

hum LQYNIYNVGSSAALDVELSDDSFPPEDFGIVSGMLNVKWDRIAPAS**N**VSHTVVLRPLKAG

pan LQYNIYNVGSSAALDVELSDDSFPPEDFGIVSGMLNVKWDRIAPASNVSHTVVLRPLKAG

mus LQYNIYNVGSSAALDVELSDDSFPPEDFGIVSGMLNVKWDRIAPASNVSHTVVLRPLKAG

************************************************************

gal YFNFTSATITYLAQEGGQVVVGFTSAPGQGGILAQREFDRRFSPHFLDWAAFGVMTLPSI

hum YF**N**FTSATITYLAQEDGPVVIGSTSAPGQGGILAQREFDRRFSPHFLDWAAFGVMTLPSI

pan YFNFTSATITYLAQEDGPVVIGSTSAPGQGGILAQREFDRRFSPHFLDWAAFGVMTLPSI

mus YFNFTSATITYLAQEDGPVVIGSTSAPGQGGILAQREFDRRFSPHFLDWAAFGVMTLPSI

***************.* **:* *************************************

gal GIPLLLWYSSKRKYDTPKTKKN

hum GIPLLLWYSSKRKYDTPKTKKN

pan GIPLLLWYSSKRKYDTPKTKKN

mus GMPLLLWYSSKRKYDTPKPKKN

*:**************** ***

1. **Interferon-beta**

gal MYAFGFIQIGFILLCTITISSLTCNHLPLQQRRVIESSLQLLDKMGRRFPQQCLREKMSF

mus MNNRWILHAAFLLCFSTTALSINYKQLQLQERTNIRKCQELLEQLNGKI-NLTYR--ADF

hum MTNKCLLQIALLLCFSTTALSMSYNLLGFLQRSSNFQCQKLLWQLNGRL-EYCLKDRMNF

pan MTNKCLLQIALLLCFSTTALSMSYNLLGFLQRSSNIQCQKLLWQLNGRL-EYCLKDRMNF

* ::: .::* : * *:. : * : :* .. :** ::. :: : : .*

gal RFPEQVL--KPRQKETVKVAIEEILQHIFYIFSKNLTLAAWDGAALEQFQNGLYQQIEKL

mus KIPMEMT--EKMQKSYTAFAIQEMLQNVFLVFRNNFSSTGWNETIVVRLLDELHQQTVFL

hum DIPEEIKQLQQFQKEDAALTIYEMLQNIFAIFRQDSSSTGW**N**ETIVENLLANVYHQINHL

pan DIPEEIKQLQQFQKEDAALTIYEMLQNIFAIFRQDSSSTGWNETIVKNLLANVYHQINHL

:* :: : **. . .:* *:**::* :* :: : :.*: : : .: :::* *

gal EACIIKKQT--QYFWSKEVNRLKLKKYFQKIDSFLKEKQHNLCSWEISRAEMRRCLQLID

mus KTVLEEKQEE-RLTWEMSSTALHLKSYYWRVQRYLKLMKYNSYAWMVVRAEIFRNFLIIR

hum KTVLEEKLEKEDFTRGKLMSSLHLKRYYGRILHYLKAKEYSHCAWTIVRVEILRNFYFIN

pan KTVLEEKPEKEDFTRGKFMSSLHLKRYYGRILHYLKAKEYSHCAWTIVRVEILRNFYFIN

:: : :* . *:** *: :: :** ::. :* : *.*: * : :*

gal KVIRKLYK

mus RLTRNFQN

hum RLTGYLRN

pan RLAGYLRN

:: : :

1. **Apolipoprotein D**

gal MLGTAAQLSVLLSLLGLGNAQMFHMGPCPDPPVQQDFDINKYLGKWYEIEKLPSNFEKGS

mus MVTMLMFLATLAGLFTTAKGQNFHLGKCPSPPVQENFDVKKYLGRWYEIEKIPASFEKGN

hum MVMLLLLLSALAGLFGAAEGQAFHLGKCPNPPVQENFDVNKYLGRWYEIEKIPTTFENGR

pan MVMLLLLFSALAGLFGAAEGQAFHLGKCPKPPVQENFDVNKYLGRWYEIEKIPTTFENGR

*: ::.* .*: .:.* **:* **.****::**::****:******:*:.**:*

gal CVQANYSLKENGKFKVINKEMLSSGKINAIEGEIMHTDVKEPAKLGVRFNWFMPSAPYWV

mus CIQANYSLMENGNIEVLNKELSPDGTMNQVKGEAKQSNVSEPAKLEVQFFPLMPPAPYWI

hum CIQA**N**YSLMENGKIKVLNQELRADGTVNQIEGEATPV**N**LTEPAKLEVKFSWFMPSAPYWI

pan CIQANYSLMENGKIKVLNQELRADGTVNQIEGEATPVNLTEPAKLEVKFSWFMPSAPYWI

*:****** ***:::*:*:*: .*.:* ::** ::.***** *:* :** ****:

gal ISTDYENYSLVYSCTNILWLFHFDYAWIMSRSPDMHPDTVEHLKSMLRTYKIDTDKMMPT

mus LATDYENYALVYSCTTFFWLFHVDFFWILGRNPYLPPETITYLKDILTSNGIDIEKMTTT

hum LATDYENYALVYSCTCIIQLFHVDFAWILARNPNLPPETVDSLKNILTSNNIDVKKMTVT

pan LATDYENYALVYSCTSIIQLFHVDFAWILARNPNLPPETVDSLKNILTSNNIDVKKMTVT

::******:****** :: ***.*: **:.*.* : *:*: **.:* : ** .** *

gal DQLNCPAEM

mus DQANCPDFL

hum DQVNCPKLS

pan DQVNCPKLS

** ***

1. **Interferon-alpha 14**

gal -MYAFGFIQIGFILLCTITISSLTCNHLPLQQRRVIESSLQLLDKMGRRFPQQCLREKMS

mus MARPCAFLMV-LVVLSYWSACSLGCDL-PQTHNLRNKRALTLLEQMRRLSPLSCLKDRKD

hum MALPFALMMA-LVVLSCKSSCSLGCNL-SQTHSLNNRRTLMLMAQMRRISPFSCLKDRHD

pan MALPFALMMA-LVVLSCKSSCSLGCDL-SQTHSLNNRRTLMLMTQMRRISPFSCLKDRHD

.:: :::*. : .** *: : . :* *: :* * * .**::: .

gal FRFPEQVLKPRQKE--TVKVAIEEILQHIFYIFSKNLTLAAWDGAALEQFQNGLYQQIEK

mus FGFPQEKVDAQQIKKAQAIPFVHELTQQILTLFTSNDSSAAWNATLLDSFCNDLHQQLND

hum FEFPQEEFDGNQFQKAQAISVLHEMMQQTFNLFSTK**N**SSAAWDETLLEKFYIELFQQMND

pan FEFPQEEFDGNQFQKAQAISVLHEMIQQTFNLFSTKNSSAAWDETLLEKFYIELFQQMND

* **:: .. .* : . :.*: *: : :*:.: : ***: : *:.* *.**::.

gal LEACIIKKQT--QYFWSKEVNRLKLKKYFQKIDSFLKEKQHNLCSWEISRAEMRRCLQLI

mus LKACLMQQVGVQEFPLTQEDSLLAVRKYFHSITVYLREKKHSPCAWEVVRAEVQRTLSSS

hum LEACVIQEVGVEETPLMNEDSILAVKKYFQRITLYLMEKKYSPCAWEVVRAEIMRSLSFS

pan LEACVIQEVGVEETPLMNEDSILAVKKYFQRITLYLMEKKYSPCAWEVVRAEIMRSLSFS

*:**:::: : :* . * ::***: * :* **::. *:**: ***: * *.

gal DKVIRKLYKVDILSSLPVMDAS

mus ANLLARLSKEE-----------

hum TNLQKRLRRKD-----------

pan TNLQKRLRRKD-----------

:: :* : :

1. **Prostaglandin H2 D-isomerase**

gal MQATLLSILGLALLGAL-----HAQNSIPVQADFQQDKLAGRWYSIGLASNSNWFKDKKH

mus MAALRMLWMGLVLLGLLGFPQTPAQGHDTVQPNFQQDKFLGRWYSAGLASNSSWFREKKA

hum MATHHTLWMGLALLGVLGDLQAAPEAQVSVQPNFQQDKFLGRWFSAGLAS**N**SSWLREKKA

pan MATHHTLWMGLALLGVLGDLQAAPEAQVSVQPNFQQDKFLGRWFSAGLASNSSWLREKKA

* : :**.*** * : ** :*****: ***:* ******.*:::**

gal LLKMCTTDIAVTADGNMEVTSTYPKGEQCEKRNSLYIRTEQPGRFSYTNPRWGSNHDIRV

mus VLYMCKTVVAPSTEGGLNLTSTFLRKNQCETKIMVLQPAGAPGHYTYSSPHSGSIHSVSV

hum ALSMCKSVVAPATDGGL**N**LTSTFLRKNQCETRTMLLQPAGSLGSYSYRSPHWGSTYSVSV

pan ALSMCKSVVAPAADGGLNLTSTFLRKNQCETRTMLLQPAGSLGSYSYRSPHWGSTYSVSV

* **.: :* :::*.:::***: : :***.: : : * ::* .*: ** :.: *

gal VETNYDEYALVATQISKSTGS-SNMVLLYSRTKEVAPQRLERFMQFSQEQGLKDEEILIL

mus VEANYDEYALLFSRGTKGPGQDFRMATLYSRTQTLKDELKEKFTTFSKAQGLTEEDIVFL

hum VETDYDQYALLYSQGSKGPGEDFRMATLYSRTQTPRAELKEKFTAFCKAQGFTEDTIVFL

pan VETDYDQYALLYSQGSKGPGEDFRMATLYSRTQTPRAELKEKFTAFCKAQGFTEDTIVFL

**::**:***: :: :*. *. .*. *****: : *:* *.: **:.:: *::*

gal PQTDKCMADAA

mus PQPDKCIQE--

hum PQTDKCMTEQ-

pan PQTDKCMMEQ-

** ***: :

1. **UPF0669 protein C6orf120**

gal ------------------------------------------------------------

mus ------------------------------------------------------------

hum ------------------------------------------------------------

pan MYKARPPAGSGGESEGATTALPLPLPLVGAALGGGVNPPPPPRGLTRVGRDSPSDRPGAW

gal -------------------------------------------MAARWRRILIVFVAAQV

mus -------------------------------------------MATPWRRALLMILASQV

hum -------------------------------------MAAPRGRAAPWTTALLLLLASQV

pan TRRGPACGRRGQAPVLSASGVPSSTDPLAGPGAEPPAMAAPRGRAAPWTTALLLLLASQV

*: * *::::*:**

gal LCLVNTFEEEDVPEEWILLHVVQGQIGAGNYSYLRLNHEGKIVLQMQSLKGDADLYVSDM

mus VTLVKCLEDDDVPEEWLLLHVVQGQIGAGNYSYLRLNHEGKIILRMQSLRGDADLYVSDS

hum LSPGSCADEEEVPEEWVLLHVVQGQIGAG**N**YSYLRLNHEGKIVLRMRSLKGDADLYVSAS

pan LSPGSCADEEEVPEEWVLLHVVQGQIGAGNYSYLRLSHEGKIVLRMRSLKGDADLYVSAS

: . ::::*****:*******************.*****:*:*:**:********

gal TLHPSFDEYELQSVTCGQDIVHVPAHFRRPVGIGIYGHPSHQESEFEMKVYYDRTVVQYP

mus TPHPSFDDYELQSVTCGQDVVSIPAHFQRPVGIGIYGHPSHHESDFEMRVYYDRTVDQYP

hum SLHPSFDDYELQSATCGPDAVSIPAHFRRPVGIGVYGHPSHLESEFEMKVYYDGTVEQHP

pan SLHPSFDDYELQSATCGPDAVSIPAHFRRPVGIGVYGHPSHLESEFEMKVYYDGTVEQHP

: *****:*****.*** * * :****:******:****** **:***:**** ** *:*

gal FGEASYNPEEMEANQKYSHSTEDESQDEESVFWTVLIGILKLILEILF

mus FGEAAYFTDPTGASQQQAYAPEEAAQEEESVLWTILISILKLVLEILF

hum FGEAAYPADGADAGQKHAGAPEDASQEEESVLWTILISILKLVLEILF

pan FGEAAYPADGAEAGQKHARAPEDASQEEESVLWTILISILKLVLEILF

****:* : *.*: : : *: :*:****:**:**.****:*****

1. **Erythropoeitin**

gal MDVNGAGLCAVL----LLLLLLRGGAGGRPDGPPSLCDPRVMERFIREARDAERGMVGCG

hum ---MGVHECPAWLWLLLSLLSLPLGLPVLGAPPRLICDSRVLERYLLEAKEAE**N**ITTGCA

pan ---MGVHECPAWLWLLLSLLSLPLGLPVLGAPPRLICDSRVLERYLLEAKEAENITTGCA

mus ---MGVPERPTL-LLLLSLLLIPLGLPVLCAPPRLICDSRVLERYILEAKEAENVTMGCA

*. . * ** : * * :** **:**:: **::**. **.

gal RRCDLPEAVAVPDPGVSFSEWQRMDVGARVRAVLGGHAVLVAAVLRARELLSDPQLRP--

hum EHCSLNE**N**ITVPDTKVNFYAWKRMEVGQQAVEVWQGLALLSEAVLRGQALLV**N**SSQPWEP

pan EHCSLNENITVPDTKVNFYAWKRMEVRQQAVEVWQGLALLSEAVLRGQALLVNSSQPWEP

mus EGPRLSENITVPDTKVNFYAWKRMEVEEQAIEVWQGLSLLSEAILQAQALLANSSQPPET

. * * ::*** *.* *:**:* :. * * ::* *:*:.: ** : .

gal ---TLDLIYGAARSLAHLLRGVVSPPTPTPTRTPHSPTPTPFSPTPSSPTPFSPPSSPHS

hum LQLHVDKAVSGLRSLTTLLRALGAQK-----------------------EAIS---PPDA

pan LQLHVDKAVSGLRSLTTLLRALGAQK-----------------------EAIS---PPDA

mus LQLHIDKAISGLRSLTSLLRVLGAQK-----------------------ELMS---PPDT

:* .. ***: *** : : :* *.:

gal APPPPPPPPQVRTLSRLLGVHSAFLRGKVRLLLIDVCTPVSPPRHWR

hum ASAAPLRTITADTFRKLFRVYSNFLRGKLKLYTGEACRTGDR-----

pan ASAAPLRTITADTFRKLFRVYSNFLRGKLKLYTGEACRTGDR-----

mus TPPAPLRTLTVDTFCKLFRVYANFLRGKLKLYTGEVCRRGDR-----

: * . *: :*: *:: *****::* :.* .

1. **Ganglioside activator GM2**

gal ---------MLLAGLFLALCAPSLCPAVLGAARPQLLVERSGPRMLREVGGFSWEDCGNG

mus ---MHRLPLLLLLGLLLAG---SV------------APARLVPKRLSQLGGFSWDNCDEG

hum MQSLMQAPLLIALGLLLAA------------------PAQAHLKKPSQLSSFSWDNCDEG

pan MQSLMQAPLLIALGLLLAA------------------PAQAHLKKPSQLSSFSWDNCDEG

:: **:** : : ::..***::*.:*

gal RDPVVLQSLSVAPDPISIPGNLRISAAVSSSKAMTSPLKAVLVVEKALGDLWIQLPCIDQ

mus KDPAVIKSLTTQPDPIVVPGDVVVSLEGKTSVPLTAPQKVELTVEKEVAGFWVKIPCVEQ

hum KDPAVIRSLTLEPDPIIVPG**N**VTLSVMGSTSVPLSSPLKVDLVLEKEVAGLWIKIPCTDY

pan KDPAVIRSLTLEPDPIIVPGNVTLSVVGSTSVPLSSPLKVDLVLEKEVAGLWIKIPCTDY

:**.*::**: **** :**:: :* .:* :::* *. *.:** :..:*:::** :

gal LGSCTYDDVCNILDNLIPPGTPCPEPLLTYGIPCHCPFKAGSYSLPASDFALPDVELPSW

mus LGSCSYENICDLIDEYIPPGESCPEPLHTYGLPCHCPFKEGTYSLPTSNFTVPDLELPSW

hum IGSCTFEHFCDVLDMLIPTGEPCPEPLRTYGLPCHCPFKEGTYSLPKSEFVVPDLELPSW

pan IGSCTFEHFCDVLDMLIPTGEPCPEPLRTYGLPCHCPFKEGTYSLPKSEFVVPDLELPSW

:***:::..*:::* ** * ***** ***:******* *:**** *:*.:**:*****

gal MTNGNYRVRAVVSNKGQELACVKLGFSLQSQ

mus LSTGNYRIQSILSSGGKRLGCIKIAASLKGR

hum LTTGNYRIESVLSSSGKRLGCIKIAASLKGI

pan LTTGNYRIESVLSSGGKRLGCIKIAASLKGI

::.****:.:::*. *:.*.*:*:. **:.

1. **Interleukin-18 binding protein**

gal -----------------------------------------------MAPCPRSDAMA--

mus MTMRHCWTAGPSSWWVLLLYVHVILARATSAP----QTTATVLTGSSKDPCSSWSPAVP-

hum MTMRHNWTPDLSPLWVLLLCAHVVTLLVRATPVSQTTTAATASVRSTKDPCPSQPPVFPA

pan MTMRHNWTPDLSPLWVLLLCAHVVTLLVRATPVSQTTKAATASVRSTKDPCPSQPPVFPA

**

gal -LQPPRITRLSTPAQTPQMGSNVSVSCEAESALPELTLLYWLGNGSFVEQLQPNVREGAV

mus TKQYPAL-DVIWPEKEVPLNGTLTLSCTACSRFPYFSILYWLGNGSFIEHLPGRLKEGHT

hum AKQCPAL-EVTWPEVEVPL**N**GTLSLSCVACSRFP**N**FSILYWLG**N**GSFIEHLPGRLWEGST

pan AKQCPAL-EVTWPEVEVPLNGTLSLSCVACSRFPNFSILYWLGNGSFIEHLPGQLWEGST

* * : : * :...:::** * * :* :::*********:*:* .: ** .

gal REETWGSLATLRRDLHFTPFSFQDLSTNFTCVALSPSGVDIRKLQ----WVPFGADPSNE

mus SREHRNTSTWLHRALVLEELSPTLRSTNFSCLFVDPGQVAQYHIILAQLWDGLKTAPPPS

hum SRERGSTGTQLCKALVLEQLTPALHST**N**FSCVLVDPEQVVQRHVVLAQLWAGLRATLPPT

pan SRERGSTGTQLCKALVLEQLTPALHSTNFSCVLVDPEQVVQRHVVLAQLWAGLRATLPPT

.* .: : * : * : :: ****:*: :.* * :: * : :

gal --------------GGGMG

mus QETLSSHSPVSRSAGPGVA

hum QEALPSSHSSPQQQG----

pan QEALPSSHSSPQQQG----

*

1. **Interferon-omega-1**

gal -MYAFGFIQIGFILLCTITISSLTCNHLPLQQRRVIESSLQLLDKMGRRFPQQCLREKMS

hum MALLFPLLA-ALVMTSYSPVGSLGCD-LPQNHGLLSRNTLVLLHQMRRISPFLCLKDRRD

pan MALLFPLLA-ALVMTSYSPVGSLGCD-LPQNHGLLSRNTLVLLHQMRRISPFLCLKDRRD

mus MARPFAFLM-VLVVISYWSTCSLGCD-LPQTHNLRNKKILTLLAQMRRLSPLSCLKDRKD

* :: ::: . ** *: ** : .. * ** :* * * **::: .

gal FRFPEQVLKPRQKE--TVKVAIEEILQHIFYIFSKNLTLAAWDGAALEQFQNGLYQQIEK

hum FRFPQEMVKGSQLQKAHVMSVLHEMLQQIFSLFHTERSSAAW**N**MTLLDQLHTGLHQQLQH

pan FRFPQEMVKGSQLQKAQVMSVLHEMLQQIFSLFHTERSSAAWNMTLLDQLHTGLHQQLQH

mus FGFPQEKVDAQQIQEAQAIPVLSELTQQILTLFTSKDSSAAWNATLLDSFCTGLHQLLND

* **:: :. * : . .: *: *:*: :* .: : ***: : *:.: .**:* ::.

gal LEACIIKKQTQ--YFWSKEVNRLKLKKYFQKIDSFLKEKQHNLCSWEISRAEMRRCLQLI

hum LETCLLQVVGEGESAGAISSPALTLRRYFQGIRVYLKEKKYSDCAWEVVRMEIMKSLFLS

pan LETCLLQVMGEGESAGAISSPALTLRRYFQGIRVYLKEKKYSDCAWEVVRMEIMKSLFLS

mus LQGCLMQLVGMKELPLTQEDSQLAMKKYFHRITVYLREKKHSPCAWEVVRAEVWRALSSS

*: *::: : . * :::**: * :*:**::. *:**: * *: :.*

gal DKVIRKLYKVDILSSLPVMDAS

hum TNMQERLRSKDRDLGSS-----

pan TNMQERLRSKDRDLGSSRNDSH

mus VNLLARLSEEKE----------

:: :* . .

1. **Neutrophil gelatinase** **-associated lipocalin**

gal MQATLLSILGLALLGALHAQN--------------SIPVQADFQQDKLAGRWYSIGLASN

mus -MALSVMCLGLALLGVLQSQAQDSTQNLIPAPSLLTVPLQPDFRSDQFRGRWYVVGLAGN

hum -MPLGLLWLGLALLGALHAQAQDSTSDLIPAPPLSKVPLQQNFQDNQFQGKWYVVGLAGN

pan -MPLGLLWLGLALLGALHAQAQDSTSDLIPAPPLSKVPLQQNFQDNQFQGKWYVVGLAGN

: *******.*::* .:*:* :*:.::: *:** :***.*

gal SNWFKDKKHLLKMCTTDIAVTADGNMEVTSTYPK--GEQCEKRNSLYIRTEQPGRFSYTN

mus AVQKKTEG-SFTMYSTIYELQENNSYNVTSILVRDQDQGCRYWIRTFVPSSRAGQFTLGN

hum AILREDKD-PQKMYATIYELKEDKSY**N**VTSVLFRK--KKCDYWIRTFVPGCQPGEFTLGN

pan AILREDKD-PQKMYATIYELKEDKSYNVTSVLFRK--KKCDYWIRTFVPGRQPGEFTLGN

: : : .* :* : : . :*** : : * :: : *.*: *

gal PRWG---SNHDIRVVETNYDEYALVATQISKSTGSSNMVLLYSRTKEVAPQRLERFMQFS

mus MHRYPQVQSYNVQVATTDYNQFAMVFFRKTSENKQYFKITLYGRTKELSPELKERFTRFA

hum IKSYPGLTSYLVRVVSTNYNQHAMVFFKKVSQNREYFKITLYGRTKELTSELKENFIRFS

pan IKSYPGLTSYLVRVVSTNYNQHAMVFFKKVSQNREYFKITLYGRTKELTSELQENFIRFS

: .: ::*. *:*::.*:* : ... . : **.****:: : *.* :*:

gal QEQGLKDEEILILPQTDKCMADAA

mus KSLGLKDDNIIFSVPTDQCIDN--

hum KSLGLPENHIVFPVPIDQCIDG--

pan KSLGLPENHIVFPVPIDQCIDG--

:. ** ::.*:: *:*: .

1. **Alpha-1-acid glycoprotein 1**

gal MLAFLVPVLILAMGLVGAHGTESPTCAPL-VPADMDNATVDRLLGHWVYIMGASQYPPHM

mus ---MALHTVLIILSLLPMLEAQNPEHANFTIGEPITNETLSWLSDKWFFMGAAFRKLEYR

hum ---MALSWVLTVLSLLPLLEAQIPLCANL-VPVPIT**N**ATLDQITGKWFYIASAFRNEEY**N**

pan ---MALSWVLTVLSLLPLLEAQIPLCANL-VPVPITNATLDRISGKWFYIASAFRNEEYN

: : :: :.*: :: * * : : : * *:. : .:*.:: .* : :

gal AEMRELKYATFTLFPGSHEDEFNVTEIMRLNETCVVKNSSKIHVFRHNSTLTHEDGQVVS

mus QAIQTMQSEFFYLTTNLINDTIELRESQTIGDQCVY-NSTHLGFQRENGTFSKYEGGVET

hum KSVQEIQATFFYFTP**N**KTEDTIFLREYQTRQDQCIY-**N**TTYLNVQRE**N**GTISRYVGGQEH

pan KSVQEIQATFFYFTPNKTEDTIFLREYQTRQNQCFY-NSSYLNVQRENGTVSRYEGGREH

:: :: * : . :* : : * : *. *:: : . *.*.*.:: *

gal MAELIHSDK-DLFIL--KHFKDNHVGLSLSARTAEVTKEQLEEFEAQLRCHGFKLEEAF-

mus FAHLIVLRKHGAFMLAFDLKDEKKRGLSLYAKRPDITPELQEVFQKAVTHVGMDESEIIF

hum FAHLLILRDTKTYMLAFDVNDEKNWGLSVYADKPETTKEQLGEFYEALDCLRIPKSDVVY

pan VAHLLFLRDTKTLMFGSYLDDEKNWGLSVYADKPEATKEQLGEFYEALDCLCIPRSEVMY

.*.*: . :: .::: ***: * : * * * : : .: .

gal ITSPKDACPAAGEETG-EGSAATAEPQLG--

mus VDWKKDRCGQQEKKQLELGKETKKDPEEGQA

hum TDWKKDKCEPLEKQHEKER-----KQEEGES

pan TDWKKDKCEPLEKQHEKER-----KQEEGES

** * :: . : *

1. **Alpha acid-glycoprotein 2**

gal MLAFLVPVLILAMGLVGAHGTESPTCAPL-VPADMDNATVDRLLGHWVYIMGASQYPPHM

mus ---MALHTVLIILSLLPMLEAQNPEHANFTIGEPITNETLSWLSDKWFFMGAAFRKLEYR

hum ---MALSWVLTVLSLLPLLEAQIPLCANL-VPVPIT**N**ATLDRITGKWFYIASAFRNEEY**N**

pan ---MALSWVLTVLSLLPLLEAQIPLCANL-VPVPITNATLDRISGKWFYIASAFRNEEYN

: : :: :.*: :: * * : : : * *:. : .:*.:: .* : :

gal AEMRELKYATFTLFPGSHEDEFNVTEIMRLNETCVVKNSSKIHVFRHNSTLTHEDGQVVS

mus QAIQTMQSEFFYLTTNLINDTIELRESQTIGDQCVY-NSTHLGFQRENGTFSKYEGGVET

hum KSVQEIQATFFYFTP**N**KTEDTIFLREYQTRQNQCFY-**N**SSYLNVQRE**N**GTVSRYEGGREH

pan KSVQEIQATFFYFTPNKTEDTIFLREYQTRQNQCFY-NSSYLNVQRENGTVSRYEGGREH

:: :: * : . :* : : * : *. **: : . *.*.*.:: :*

gal MAELIHSDKD-LFILK--HFKDNHVGLSLSARTAEVTKEQLEEFEAQLRCHGFKLEEAF-

mus FAHLIVLRKHGAFMLAFDLKDEKKRGLSLYAKRPDITPELQEVFQKAVTHVGMDESEIIF

hum VAHLLFLRDTKTLMFGSYLDDEKNWGLSFYADKPETTKEQLGEFYEALDCLCIPRSDVMY

pan VAHLLFLRDTKTLMFGSYLDDEKNWGLSVYADKPEATKEQLGEFYEALDCLCIPRSEVMY

.*.*: . ::: .::: ***. * : * * * : : .: :

gal ITSPKDACPAAGEET-GEGSAATAEPQLG--

mus VDWKKDRCGQQEKKQLELGKETKKDPEEGQA

hum TDWKKDKCEPLEKQHEKERK-----QEEGES

pan TDWKKDKCEPLEKQHEKERK-----QEEGES

** * :: . : *

1. **Ephrin-A1**

gal MPRWEAAALLAAIVGVCVWSDDPGKVISDRYAVYWNRSNPRFHRGDYTVEVSINDYLDIY

hum -----MEFLWAPLLGLCCS-----LAAADRHTVFW**N**SSNPKFRNEDYTIHVQLNDYVDII

pan -----MEFLWAPLLGLCCS-----LAAADRHTVFWNSSNPKFRNEDYTIHVQLNDYVDII

mus -----MEFLWAPLLGLCCS-----LAAADRHIVFWNSSNPKFREEDYTVHVQLNDYLDII

* * ::*:* . :**: *:** ***:*:. ***:.*.:***:**

gal CPHYEEPL-PAERMERYVLYMVNYEGHASCDHRQKGFKRWECNRPDSPSGPLKFSEKFQL

hum CPHYEDHSVADAAMEQYILYLVEHEEYQLCQPQSKDQVRWQCNRPSAKHGPEKLSEKFQR

pan CPHYEDHSVADAAMEQYILYLVEHEEYQLCQPQSKDQVRWQCNRPSAKHGPEKLSEKFQR

mus CPHYEDDSVADAAMERYTLYMVEHQEYVACQPQSKDQVRWNCNRPSAKHGPEKLSEKFQR

*****: **:* **:*::: : *: :.*. **:****.: ** *:*****

gal FTPFSLGFEFRPGHEYYYISASPLNVVDRPCLKLKVYVRPTNDSLY-----------ESP

hum FTPFTLGKEFKEGHSYYYISK-PIHQHEDRCLRLKVTVSGKITHSPQAHDNPQEKRLAAD

pan FTPFTLGKEFKEGHSYYYISK-PIHQHEDRCLRLKVTVSGKITHSPQAHDNPQEKRLAAD

mus FTPFILGKEFKEGHSYYYISK-PIYHQESQCLKLKVTVNGKITHNPQAHVNPQEKRLQAD

**** ** **: **.***** *: : **:*** * . :

gal EPIFTSNNSCCSLAVPRAVLVAAPVFWTLLGS--------

hum DPEVRVLHSIGHSAAPR----LFPLAWTVLLLPLLLLQTP

pan DPEVRVLHSIGHSAAPR----LFPLAWTVLLLPLLLLQTP

mus DPEVQVLHSIGYSAAPR----LFPLVWAVLLLPLLLLQSQ

:* . :* *.** *: *::*

1. **Platelet glycoprotein Ib beta chain**

gal MN-----SGILFLSLLGFLPSVIPTCPLPCKCATNIIDCMSKGLTVTRLPAAFRPSAEII

mus MGSRPRGALSLLLLLLALLSRPASGCPAPCSCAGTLVDCGRRGLTWASLPAAFPPDTTEL

hum MGSGPRGALSLLLLLLAPPSRPAAGCPAPCSCAGTLVDCGRRGLTWASLPTAFPVDTTEL

pan MGSGPRGALSLLLLLLAPPSRPAAGCPAPCSCAGTLVDCGRRGLTWASLPTAFPVDTTEL

*. : *:* **. ** **.** .::** :*** : **:** .: :

gal NLSYNRLTSIPSGLFDNLNSLQAVHLQGNPWECDCDILYLRSWLQWQQNRTFYRDVRCAS

mus VLTGNNLTALPPGLLDALPALRAAHLGANPWRCDCRLLPLRAWLAGRPERAPYRDLRCVA

hum VLTGN**N**LTALPPGLLDALPALRTAHLGANPWRCDCRLVPLRAWLAGRPERAPYRDLRCVA

pan VLTGNNLTALPPGLLDALPALRTAHLGANPWRCDCRLVPLRAWLAGRPERAPYRDLRCVA

*: *.**::* **:* * :*::.** .***.*** :: **:** : :*: ***:**.:

gal PAHLQGRVVAYLTEDEIISTCQYW-YCSLALFSQLVLIILLFLQAILVIFIIIYLQRFRR

mus PPALRGRLLPYVAEDELRAACAPGLLCWGALVAQLALLVLGLLHALLLALL---LGRLRR

hum PPALRGRLLPYLAEDELRAACAPGPLCWGALAAQLALLGLGLLHALLLVLL---LCRLRR

pan PPALRGRLLPYLAEDELRAACAPGPLCWGALAAQLALLGLGLLHALLLVLL---LCRLRR

* *:**:: *::***: ::* * ** :**.*: * :*:*:*: :: * *:**

gal MAAEARSTTRDLYQRADTWSLQEQ-----

mus LRARARARSIQEFSLTAPLVAESARGGAS

hum LRARARARAAARLSLTDPLVAERAGTDES

pan LRARARARAAARLSLTDPLVAERAGTDEF

: *.**: : . : :

1. **Metalloproteinase inhibitor-1 (TIMP)**

gal --MPGAALPSLLAWLAVLLLGRARPADACSCSPIHPQQAFCNADVVIRAKRVSAKEVDSG

hum -MAPFEPL----ASGILLLLWLIAPSRACTCVPPHPQTAFCNSDLVIRAKFVGTPEV**N**QT

pan -MAPFEPL----ASGILLLLWLIAPSRACTCVPPHPQTAFCNSDLVIRAKFVGTPEVNQT

mus MMAPFASL----ASGILLLLSLIASSKACSCAPPHPQTAFCNSDLVIRAKFMGSPRINET

* * * :*** : **:* * *** ****:*:***** :.: .::.

gal NDIYGNPIKRIQYEVKQIKMFKGPDQDIEFIYTAPSTEVCGQPLDTGGKKEYLIAGKSEG

hum TLYQRYEIKM-TKMYKGF-QALGDAADIRFVYTPAMESVCGYFHRSH**N**RSEEFLIAGKLQ

pan TLYQRYEIKM-TKMYKGF-QALGDAADIRFVYTPAMESVCGYFHRSHNRSEEFLIAGKLQ

mus TLYQRYKIKMMTKMLKGF-KAVGNAADIRYAYTPVMESLCGYAHKSQNRSEEFLITGRLR

. ** * : * **.: ** .:** : .:.* ::

gal DGKMHITLCDLVATWDSVSPTQKKSLN-QRYQMGCE-CKISRCLSIPCFVSSSDECLWTD

hum DGLLHITTCSFVAPWNSLSLAQRRGFT-KTYTVGCEECTVFPCLSIPCKLQSGTHCLWTD

pan DGLLHITTCSFVAPWNSLSLAQRRGFT-KTYTVGCEECTVFPCLSIPCKLQSGTHCLWTD

mus NGKFHINACSFLVPWRTLSPAQQRVFSKKNYSAGCGVCTVFPCLSIPCKLESDTHCLWTD

:* :**. *.::. * ::* :*:: :. : * ** *.: ****** :.*. .*****

gal WAMEKIVGGRQAKHYACIKRSDGSCAWYRGMAPPKQEFLDIEDP

hum QLLQGSEKGFQSRHLACLPREPGLCTWQSLRSQIA---------

pan QLLQGSEKGFQSRHLACLPREPGLCTWQSLRSQIA---------

mus QVLVGSE-DYQSRHFACLPRNLGLCTWRSLGAR-----------

: . *::* **: *. * *:* :

1. **Fibroblast growth factor 9**

gal MAPLGEVGNYFGVQDAVPFGNVPALPADSPVLLSDHLGQAEAGGLPRGPAVTDLDHLKGI

hum MAPLGEVGNYFGVQDAVPFGNVPVLPVDSPVLLSDHLGQSEAGGLPRGPAVTDLDHLKGI

pan MAPLGEVGNYFGVQDAVPFGNVPVLPVDSPVLLSDHLGQSEAGGLPRGPAVTDLDHLKGI

mus MAPLGEVGSYFGVQDAVPFGNVPVLPVDSPVLLSDHLGQSEAGGLPRGPAVTDLDHLKGI

********.**************.**.************:********************

gal LRRRQLYCRTGFHLEIFPNGTIQGTRQDHSRFGILEFISIAVGLVSIRGVDSGLYLGMNE

hum LRRRQLYCRTGFHLEIFP**N**GTIQGTRKDHSRFGILEFISIAVGLVSIRGVDSGLYLGMNE

pan LRRRQLYCRTGFHLEIFPNGTIQGTRKDHSRFGILEFISIAVGLVSIRGVDSGLYLGMNE

mus LRRRQLYCRTGFHLEIFPNGTIQGTRKDHSRFGILEFISIAVGLVSIRGVDSGLYLGMNE

**************************:*********************************

gal KGELYGSEKLTQECVFREQFEENWYNTYSSNLYKHVDTGRRYYVALNKDGTPREGTRTKR

hum KGELYGSEKLTQECVFREQFEENWYNTYSSNLYKHVDTGRRYYVALNKDGTPREGTRTKR

pan KGELYGSEKLTQECVFREQFEENWYNTYSSNLYKHVDTGRRYYVALNKDGTPREGTRTKR

mus KGELYGSEKLTQECVFREQFEENWYNTYSSNLYKHVDTGRRYYVALNKDGTPREGTRTKR

************************************************************

gal HQKFTHFLPRPVDPEKVPELYKDILSQS

hum HQKFTHFLPRPVDPDKVPELYKDILSQS

pan HQKFTHFLPRPVDPDKVPELYKDILSQS

mus HQKFTHFLPRPVDPDKVPELYKDILSQS

**************:*************

1. **Calcium-activated Potassium channel subunit beta 4**

gal MARGRAAYEYTEAEDKSMRLGFLLIAAGLLSLLGLGCFWLRPALQERGGGGAANCTVLAV

hum MAKLRVAYEYTEAEDKSIRLGLFLIISGVVSLFIFGFCWLSPALQDLQA-TEA**N**CTVLSV

pan MAKLRVAYEYTEAEDKSIRLGLFLIISGVVSLFIFGFCWLSPALQDLQA-TEANCTVLSV

mus MAKLRVSYEYTEAEDKSIRLGLFLIVSGILSLFIFGFCWLSPALQDLQA-TAANCTVLSV

**: *.:**********:***::** :*::**: :* ** ****: . ******:*

gal RQLGERFACTFSCGAACRGTARYPCLQVLVRTSRSAAPALLHEDERQLRTNPKCSYIPPC

hum QQIGEVFECTFTCGADCRGTSQYPCVQVYV**N**NSESNSRALLHSDEHQLLTNPKCSYIPPC

pan QQIGEVFECTFTCGADCRGTSQYPCVQVYVNNSESNSRALLHSDEHQLLTNPKCSYIPPC

mus QQIGEVFECTFTCGTDCRGTSQYPCVQVYVNNSESNSRALLHSDQHQLLTNPKCSYIPPC

:*:** * ***:**: ****::***:** *..*.* : ****.*::** ***********

gal ARDDQENSENVTYKQKYWKEKVGSQPFTCYFNQHLRPDDVMLKRTHDETVLLHCFLWPLV

hum KRENQKNLESVMNWQQYWKDEIGSQPFTCYFNQHQRPDDVLLHRTHDEIVLLHCFLWPLV

pan KRENQKNLESVMNWQQYWKDEIGSQPFTCYFNQHQRPDDVLLHRTHDEIVLLHCFLWPLV

mus KRENQKNSESVMNWQQYWKDEIGSQPFTCYFNQHQRPEDVLLQRTHDEIALLHCFLWPVV

*::*:* *.* *:***:::************ **:**:*:***** .********:*

gal TFLLGVLIVALTACARSLAARAEAIQRKKHS

hum TFVVGVLIVVLTICAKSLAVKAEAMKKRKFS

pan TFVVGVLIVVLTICAKSLAVKAEAMKKRKFS

mus AFVVGVLIVVLTICAKSLAVKAEAMKKRKFS

:*::*****.** **:***.:***::::*.*

1. **Myelin protein zero-like protein subunit 2**

gal MP-GPTWLGAVLVLGVQLRALWPVAAIEVYTSKEVYAVNGTSLRLKCTFSSSSPISPLLS

mus MYGKSPALVLPLLLSLQLTALCPTEAVEIYTSGALEAVNGTDVRLKCTFSSFAPVGDALT

hum MYGKSSTRAVLLLLGIQLTALWPIAAVEIYTSRVLEAV**N**GTDARLKCTFSSFAPVGDALT

pan MYGKSSTRAVLLLLGIQLTALWPIAAVEIYTSRVLEAVNGTDARLKCTFSSFAPVGDALT

* *:*.:** ** * *:*:*** : *****. ******** :*:. *:

gal VTWNFQPEDLSSHEPVFYYLKEPYTPSAGRFKGRITWDGHIERHDVSIVIWDLQPTDNGT

mus VTWNFRPRDGGREQFVFYYHMDPFRPMSGRFKDRVVWDGNPERYDVSILLWKLQFDDNGT

hum VTWNFRPLDGGPEQFVFYYHIDPFQPMSGRFKDRVSWDGNPERYDASILLWKLQFDD**N**GT

pan VTWNFRPLDGGPEQFVFYYHIDPFQPMSGRFKDRVSWDGNPERYDASILLWKLQFDDNGT

*****:* * . .: **** :*: * :****.*: ***: **:*.**::*.** ****

gal FTCQVKNPRDIDGTIGEVRLRVVQKVNFSEIHFLAIAIGSACGLMIIVVTLVIICRHRRK

mus YTCQVKNPPDVDGLVGTIRLSVVHTVPFSEIYFLAVAIGSACALMIIVVIVVVLFQHFRK

hum YTCQVKNPPDVDGVIGEIRLSVVHTVRFSEIHFLALAIGSACALMIIIVIVVVLFQHYRK

pan YTCQVKNPPDVDGVIGEIRLSVVHTVRFSEIHFLALAIGSACALMIIIVIVVVLFQHYRK

:******* *:** :* :** **:.* ****:***:******.****:* :*:: :* **

gal KQQEKMIE-VADTELREKEKLKNIGEKEITPLED--

mus KRWADRADKAEGTKSKEEEKL-NQGNKVSVFVEDTD

hum KRWAERAHKVVEIKSKEEERL-NQEKKVSVYLEDTD

pan KRWAERAHKVVEIKSKEEERL-NQEKKVPVYLEDTD

*: . . . : :*:*:* * :* . :**

1. **T-cell specific glycoprotein CD28**

gal -MLGILVVLCLIPAADVTENKILVAQRPLLIVANRTATLVCNYTYNGTGKEFRASLHKGT

hum -MLRLLLALNLFPSIQVTGNKILVKQSPMLVAYDNAV**N**LSCKYSYNLFSREFRASLHKGL

pan -MLRLLLALNLFPSIQVTGNKILVKQSPMLVAYDNAVNLSCKYSYNLFSREFRASLHKGL

mus MTLRLLFLALNFFSVQVTENKILVKQSPLLVVDSNEVSLSCRYSYNLLAKEFRASLYKGV

* :*. : : :** ***** * *:*:. .. ..* *.*:** .:******:**

gal DSAVEVCFISWNMTK-INSNSNKEFNCRGIHDKDKVIFNLWNMSASQTDIYFCKIEAMYP

hum DSAVEVCVVYG**N**YSQQLQVYSKTGFNCDGKLG**N**ESVTFYLQNLYV**N**QTDIYFCKIEVMYP

pan DSAVEVCVVYGNYSQQLQVYSKTGFNCDGKLGNESVTFYLQNLYVNQTDIYFCKIEVMYP

mus NSDVEVCVGNGNFTYQPQFRSNAEFNCDGDFDNETVTFRLWNLHVNHTDIYFCKIEFMYP

:* ****. * : : *: *** * .::.* * * *: ..:********* ***

gal PPYVYNEKSNGTVIHVRETPIQT--QEPESATSYWVMVAVTGLLGFYSMLITAVFIIYRQ

hum PPYLDNEKSNGTIIHVKGKHLCPSPLFPGPSKPFWVLVVVGGVLACYSLLVTVAFIIFWV

pan PPYLDNEKSNGTIIHVKGKHLCPSPLFPGPSKPFWVLVVVGGVLACYSLLVTVAFIIFWV

mus PPYLDNERS**N**GTIIHIKEKHLCHTQS---SPKLFWALVVVAGVLFCYGLLVTVALCVIWT

***: **:****:**:: . : . :*.:*.* *:* *.:*:*..: :

gal KSKRNRYRQSDYMNMTPRHPPHQKNKGYPSYAPTRDYTAYRSWQP

hum RSKRSRLLHSDYMNMTPRRPGP-TRKHYQPYAPPRDFAAYRS---

pan RSKRSRLLHSDYMNMTPRRPGP-TRKHYQPYAPPRDFAAYRS---

mus NSRRNRLLQSDYMNMTPRRPGL-TRKPYQPYAPARDFAAYRP---

.*:*.* :*********:* ..* * *** **::***

1. **Cytotoxic T-cell lymphocyte protein 4**

gal ---------------MLSAWVTV--SFLCAATATAEVMEVTQPAIVLANRQGVASLVCNY

hum MACLGFQRHKAQLNLATRTWPCTLLFFLLFIPVFCKAMHVAQPAVVLASSRGIASFVCEY

pan MACLGFQRHKAQLNLATRTWPCTLLFFLLFIPVFCKAMHVAQPAVVLASSRGIASFVCEY

mus MACLGLRRYKAQLQLPSRTWPFVALLTLLFIPVFSEAIQVTQPSVVLASSHGVASFPCEY

:* . * . .:.:.*:**::***. :*:**: *:*

gal KHIGNAKEIRVTLLKQTGDKFTEICASTYTTEFKMFSVEEVIQCHVSPSRNNVTLTLTGL

hum ASPGKATEVRVTVLRQADSQVTEVCAATYMMGNELTFL-DDSICTGTSSGNQV**N**LTIQGL

pan ASPGKATEVRVTVLRQADSQVTEVCAATYMMGNELTFL-DDSICTGTSSGNQVNLTIQGL

mus SPSHNTDEVRVTVLRQTNDQMTEVCATTFTEKNTVGFL-DYPFCSGTFNESRVNLTIQGL

:: *:***:*:*:..:.**:**:*: : : : * : . ..*.**: **

gal QANDTGLYVCKMERMYPPPYFMNKGNGTQLYVIDPEPCPDTATYLWVLGATASGFFLYSI

hum RAMDTGLYICKVELMYPPPYYLGIG**N**GTQIYVIDPEPCPDSDFLLWILAAVSSGLFFYSF

pan RAMDTGLYICKVELMYPPPYYLGIGNGTQIYVIDPEPCPDSDFLLWILAAVSSGLFFYSF

mus RAVDTGLYLCKVELMYPPPYFVGMGNGTQIYVIDPEPCPDSDFLLWILVAVSLGLFFYSF

:* *****:**:* ******::. *****:**********: **:* *.: *:*:**:

gal IISAIVVGKAIQRRQRLTTGVYVKMPSEKL--EKKVIPFHITVN

hum LLTAVSLSKMLKKRSPLTTGVYVKMPPTEPECEKQFQPYFIPIN

pan LLTAVSLSKMLKKRSPLTTGVYVKMPPTEPECEKQFQPYFIPIN

mus LVTAVSLSKMLKKRSPLTTGVYVKMPPTEPECEKQFQPYFIPIN

:::*: :.* :::*. ********** : **:. *:.* :*

1. **Mammalian ependymin-related protein 1**

gal ------------------------------------------------------------

pan MPPRMLVAPLPLHFLLAAVSSENRSGRRQWQQAVAAGSGPGRNSSRAIHWSLPRALVPAT

hum ------------------------------------------------------------

mus ------------------------------------------------------------

gal ------------------------------------------------------------

pan GTRASGSQKRQRPPKGQEAPWSRPHSRHSAERAGGSHSDPGRLSAPLGLGLPSGRGRLTA

hum ------------------------------------------------------------

mus ------------------------------------------------------------

gal --------------MALATGGLL-LLLGGLLVRG-GAGAEPCRAPGQWEGRSVRYDHGTG

pan MPGRAPLRTVPGALGAWLLGGLWAWTLCGLCGLGAVGAPRPCQAPQQWEGRQVMYQQSSG

hum MPGRAPLRTVPGALGAWLLGGLWAWTLCGLCSLGAVGAPRPCQAPQQWEGRQVMYQQSSG

mus MPGRAPLRTVPGALGAWLLSGLWAWTLCGLCSLGAVGAPRPCQAPQQWEGRQVMYQQSSG

* .** * ** * .. .**:** *****.* *::.:*

gal RNTRAAVSYDGLNQRLRILEERKALIPCKKFFEYILLYKDAVMFQIEQVTKLCSKIALTE

pan RNSRALLSYDGLNQRVRVLDERKALIPCKRLFEYILLYKDGVMFQIDQATKHCSKMTLTQ

hum RNSRALLSYDGLNQRVRVLDERKALIPCKRLFEYILLYKDGVMFQIDQATKQCSKMTLTQ

mus RNSRALLSYDGLNQRVRVLDERKALIPCKRLFEYILLYKDGVMFQIDQATKQCSKMTLTQ

**:** :********:*:*:*********::*********.*****:*.** ***::**:

gal PWDPYDIPTNSTFEDQYYIGGPGDQIMVQEWSDRKPARKLESWVGVYTVKDCYPVQETYT

pan PWDPLDIPQNSTFEDQYSIGGPQEQITVQEWSDRKSARSYETWIGIYTVKDCYPVQETFT

hum PWDPLDIPQ**N**STFEDQYSIGGPQEQITVQEWSDRKSARSYETWIGIYTVKDCYPVQETFT

mus PRDPLDIPQNSTFEDQYSIGGPQEQITVQEWSDRKSARSYETWIGIYTVKDCYPVQETFT

* ** *** ******** **** :** ******** **. *:*:*:************:*

gal KNYSVTTSTRFFDLKLGIADPSVFTPPSTCQTAQPRKMKDEC--

pan VNYSVILSTRFFDIQLGIKDPSVFTPPSTCQMAQLEKMSEDCSW

hum I**N**YSVILSTRFFDIQLGIKDPSVFTPPSTCQMAQLEKMSEDCSW

mus INYSVILSTRFFDIQLGIKDPSVFTPPSTCQMAQLEKMSEDCSW

**** ******::*** ************ ** .**.::*

1. **Protein FAM3D**

gal -----------------------------MRMTGVIRLLVLLATLLGSWFIVQTYFERSG

hum -----------------------------MRVSGVLRLLALIFAIVTTWMFIRSYMSFSM

pan MTGRAAMMDVLPSRTIGGSLPQATLHLDRMRVSGVLRLLALIFAIVTTWMFIRSYMSFSM

mus -----------------------------MRVAGLIRVVVFIFTIVTMWVFLRSYTSFSR

**::*::*::.:: ::: *.::::* . *

gal RAISLRSWLGATSEPMSTPPQPRHKCANKRDCPADHFAFRIISGAANVVGPSICFEDRIL

hum KTIRLPRWLAASPT--KEIQVKKYKCGLIKPCPANYFAFKICSGAANVVGPTMCFEDRMI

pan KTIRLPRWLAASPT--KEIQVKKYKCGLIKPCPANYFAFKICSGAANVVGPTMCFEDRMI

mus KTIRLPRWLGITP---KDIQTPKSKCGLSKICPNNFFAFKISSGAANVVGPSMCFEDEII

::* * **. : . : **. : ** :.***:* *********::****.::

gal MSTVKNNIGRGLNIALVNGKSGQLLKVGSFDMYSGDVTQLETFLQEIKDGTIMLVATYDD

hum MSPVKNNVGRGLNIALV**N**GTTGAVLGQKAFDMYSGDVMHLVKFLKEIPGGALVLVASYDD

pan MSPVKNNVGRGLNIALVNGTTGAVLGQKAFDMYSGDVMHLVKFLKEIPGGALVLVASYDD

mus MSPVRNNVGRGLNVALVNGSTGQVMKKDSFDMYSGDPQLLLNFLTEIPDSTLVLVASYDD

** *:**:*****:*****.:* :: :******* * .** ** ..:::***:***

gal AATKMNNKVRALFSALGSRYANQLGFRDNWVFLGAKGLKGKSPFEEHIKNDQKTNKYEGW

hum PGTKMNDESRKLFSDLGSSYAKQLGFRDSWVFIGAKDLRGKSPFEQFLKNSPDTNKYEGW

pan PGTKMNDESRKLFSDLGSSYAKQLGFRDSWVFIGAKDLRGKSPFEQFLKNSPDTNKYEGW

mus PGTKMNDKIKTLFSNLGSSYAKQLGFRDSWVFVGAKDLKSKSPYEQFLKNNPETNKYDGW

.****:: : *** *** **:******.***:***.*:.***:*:.:**. .****:**

gal PELLEMEGCAPRKQD

hum PELLEMEGCMPPKPF

pan PELLEMEGCMPPKPF

mus PELLELEGCVPRKVM

*****:*** * *

1. **Transmembrane emp24 domain containing protein 4**

mus MAGVGVGPLQGMVRFGLLVLTVCAACARGLYFHIGETEKRCFIEEIPDETMVIGNYRTQM

hum MAGVGAGPLRAMGRQALLLLALCATGAQGLYFHIGETEKRCFIEEIPDETMVIGNYRTQM

pan MAGVGARPLRAMGRQALLLLALCATGAQGLYFHIGETEKRCFIEEIPDETMVIGNYRTQM

gal ----------------------MAVGARGLYFHIGDREGRCFIEEIPDETMVIGNYRTQL

*. *:*******: * ********************:

mus WDKQKEVFLPSTPGLGMHVEVKDPDGK-VVLSRQYGSEGRFTFTSHTPGDHQICLHSNST

hum WDKQKEVFLPSTPGLGMHVEVKDPDGK-VVLSRQYGSEGRFTFTSHTPGDHQICLHS**N**ST

pan WDKQKEVFLPSTPGLGMHVEVKDPDGK-VVLSRQYGSEGRFTFTSHTPGDHQICLHSNST

gal WDKQSESFLPSTPGWGMHVEVKDPMAKXVVLSRQYGSEGRFTFTSHTPGEHQICLHSNST

****.* ******* ********* .* *********************:**********

mus RMALFAGGKLRVHLDIQVGEHANNYPEIAAKDKLTELQLRARQLLDQVEQIQKEQDYQRY

hum RMALFAGGKLRVHLDIQVGEHANNYPEIAAKDKLTELQLRARQLLDQVEQIQKEQDYQRY

pan RMALFAGGKLRVHLDIQVGEHANNYPEIAAKDKLTELQLRARQLLDQVEQIQKEQDYQRY

gal RMALFAGGKLRVHLDIQVGEHTNNYPEIAAKDKLTELQLRARQLLDQVEQIQKEQNYQRY

*********************:*********************************:****

mus REERFRLTSESTNQRVLWWSIAQTVILILTGIWQMRHLKSFFEAKKLV

hum REERFRLTSESTNQRVLWWSIAQTVILILTGIWQMRHLKSFFEAKKLV

pan REERFRLTSESTNQRVLWWSIAQTVILILTGIWQMRHLKSFFEAKKLV

gal REERFRMTSESTNQRVLWWSIAQTVILILTGIWQMRHLKSFFEAKKLV

******:*****************************************

1. **Ephrin A5**

gal MPHVEMLLLAVAALWVCVRGQEPGRKAVADRYAVYWNSTNPRFQQGDYHIDVCINDYLDV

hum MLHVEMLTLVFLVLWMCVFSQDPGSKAVADRYAVYW**N**SSNPRFQRGDYHIDVCINDYLDV

pan MLHVEMLTLVFLVLWMCVFSQDPGSKAVADRYAVYWNSSNPRFQRGDYHIDVCINDYLDV

mus MLHVEMLTLLFLVLWMCVFSQDPGSKVVADRYAVYWNSSNPRFQRGDYHIDVCINDYLDV

* ***** * . .**:** .*:** *.***********:*****:***************

gal FCPHYEDSVPEDKTERYVLYMVNFDGYSSCDHISKGFKRWECNRPHSPNGPLKFSEKFQL

hum FCPHYEDSVPEDKTERYVLYMVNFDGYSACDHTSKGFKRWECNRPHSPNGPLKFSEKFQL

pan FCPHYEDSVPEDKTERYVLYMVNFDGYSACDHTSKGFKRWECNRPHSPNGPLKFSEKFQL

mus FCPHYEDSVPEDKTERYVLYMVNFDGYSACDHTSKGFKRWECNRPHSPNGPLKFSEKFQL

****************************:*** ***************************

gal FTPFSLGFEFRPGREYFYISSAIPDNGRRSCLKLKVFVRPANSCMKTIGVHDRVFDVNDK

hum FTPFSLGFEFRPGREYFYISSAIPDNGRRSCLKLKVFVRPTNSCMKTIGVHDRVFDVNDK

pan FTPFSLGFEFRPGREYFYISSAIPDNGRRSCLKLKVFVRPTNSCMKTIGVHDRVFDVNDK

mus FTPFSLGFEFRPGREYFYISSAIPDNGRRSCLKLKVFVRPTNSCMKTIGVHDRVFDVNDK

****************************************:*******************

gal VENSLEPADDTVRESAEPSRGENAAQTPRIPIRLLATLLFLLAMLLIL

hum VENSLEPADDTVHESAEPSRGENAAQTPRIPSRLLAILLFLLAMLLTL

pan VENSLEPADDTVHESAEPSRGENAAQTPRIPSRLLAILLFLLAMLLTL

mus VENSLEPADDTVHESAEPSRGENAAQTPRIPSRLLAILLFLLAMLLTL

************:****************** **** ********* *

1. **Transmembrane emp24 domain containing protein 9**

gal -------------------------------MAVGARGLYFHIGDREGRCFIEEIPDETM

mus MAAVRGVRVVGSSPGLLLGRGMRAFL-LLLWLAARGSALYFHIGETEKKCFIEEIPDETM

hum MAVELGVLLVRPRPGTGLGRVMRTLL-LVLWLATRGSALYFHIGETEKKCFIEEIPDETM

pan MAVELGVLVVRPRPGTGLGRVMRTLLLLLLWLATRGSALYFHIGETEKKCFIEEIPDETM

:*. . .******: * :***********

gal VIGNYRTQLWDKQSESFLPSTPGWGMHVEVKDPMAKXVVLSRQYGSEGRFTFTSHTPGEH

mus VIGNYRTQLYDKQREEYQPATPGLGMFVEVKDPEDK-VILARQYGSEGRFTFTSHTPGEH

hum VIGNYRTQLYDKQREEYQPATPGLGMFVEVKDPEDK-VILARQYGSEGRFTFTSHTPGEH

pan VIGNYRTQLYDKQREEYQPATPGLGMFVEVKDPEDK-VILARQYGSEGRFTFTSHTPGEH

*********:*** *.: *:*** **.****** * *:*:*******************

gal QICLHSNSTRMALFAGGKLRVHLDIQVGEHTNNYPEIAAKDKLTELQLRARQLLDQVEQI

mus QICLHSNSTKFSLFAGGMLRVHLDIQVGEHANDYAEIAAKDKLSELQLRVRQLVEQVEQI

hum QICLHS**N**STKFSLFAGGMLRVHLDIQVGEHANDYAEIAAKDKLSELQLRVRQLVEQVEQI

pan QICLHSNSTKFSLFAGGMLRVHLDIQVGEHANDYAEIAAKDKLSELQLRVRQLVEQVEQI

*********:::***** ************:*:* ********:*****.***::*****

gal QKEQNYQRYREERFRMTSESTNQRVLWWSIAQTVILILTGIWQMRHLKSFFEAKKLV

mus QKEQNYQRWREERFRQTSESTNQRVLWWSILQTLILVAIGVWQMRHLKSFFEAKKLV

hum QKEQNYQRWREERFRQTSESTNQRVLWWSILQTLILVAIGVWQMRHLKSFFEAKKLV

pan QKEQNYQRWREERFRQTSESTNQRVLWWSILQTLILVAIGVWQMRHLKSFFEAKKLV

********:****** ************** **:**: *:****************

1. **Tetraspanin-8**

gal MAGVSRCLKYSMFIFNFLFWVCGCIILGVSIWIRVNKDAQEALNIN--NSNMFVGADVLI

hum MAGVSACIKYSMFTFNFLFWLCGILILALAIWVRVSNDSQAIFGSEDVGSSSYVAVDILI

pan MAGVSACIKYSMFTFNFLFWLCGILILALAIWVRVSNDSQAIFGSEDVGSSSYVAVDILI

mus MAGVSSCLKYSMFFFNFLFWVCGTLILGLAIWVRVSKDGKEIITSGDSSTNPFIAVNILI

***** *:***** ******:** :**.::**:**.:*.: : .:. ::..::**

gal AVGSIIMILGFLGCCGAIKESRCMLLLFFIGLLLILILQVTGGILGAVYKPQLEEGFNLV

hum AVGAIIMILGFLGCCGAIKESRCMLLLFFIGLLLILLLQVATGILGAVFKSKSDRIV**N**ET

pan AVGAIIMILGFLGCCGAIKESRCMLLLFFIGLLLILLLQVATGILGAVFKSKSDRIVNET

mus AVGSIIMVLGFLGCCGAVKESRCMLLLFFIGLLLILILQVAAGILGAAFKPEYNRILNET

***:***:*********:******************:***: *****.:* : :. .* .

gal ITEAANLLKENTENAKQVQESLQKFQLQGHCCGIVDGITDWGNNYNLVIDGKAVCECERK

hum LYENTKLLSATGESEKQFQEAIIVFQEEFKCCGLVNGAADWGNNFQH---YPELCACLDK

pan LYENTKLLSATGENEKQFQEAIIMFQEEFKCCGLVNGAADWGNNFQH---YPELCACLDK

mus LYENAKLLSDNTDEAKDFQKAMIVFQSEFKCCGLENGAADWGNNFVE---AKESCQCTG-

: * ::**. . :. *:.*::: ** : :***: :* :*****: * *

gal YQEENLCTYFQGRYVFKEPCKTVILNFLQTNMDIIMGIAFGMAVIEVLGLVFSMCLYCQI

hum ---QRPCQSYNGKQVYKETCISFIKDFLAKNLIIVIGISFGLAVIEILGLVFSMVLYCQI

pan ---QRSCQSYNGKEVYKETCISFIKDFLAKNFIIVIGIAFGLAVIEILGLVFSMVLYCQI

mus ----TDCATYQGSSVYPKTCLSLIKDLFEKNIIIVIGIAFGLAVIEILGLVFSMVLYCQI

* ::* *: : * :.* ::: .*: *::**:**:****:******* *****

gal QRK

hum GNK

pan GNK

mus GSK

*

1. CD63 ANTIGEN

gal MAVEGGMKCVKFLVFIFNFIFWVCGVALVAIGIYAHVALNKALVAGSSSAASSPVAIMVV

mus MAVEGGMKCVKFLLYVLLLAFCACAVGLIAIGVAVQVVLKQAITHETTAGSLLPVVIIAV

hum MAVEGGMKCVKFLLYVLLLAFCACAVGLIAVGVGAQLVLSQTIIQGATPGSLLPVVIIAV

pan MAVEGGMKCVKFLLYVLLLAFCACAVGLIAVGVGAQLVLSQTIIQGATPGSLLPVVIIAV

*************:::: : * .*.*.*:*:*: .::.*.::: :: .: **.*:.*

gal GIIIFFVSFFGCCGAWKESYCMVTTFAVLLSIIFLVEIAAAIAGYVFKDKVRSVLEEGLW

mus GAFLFLVAFVGCCGACKENYCLMITFAIFLSLIMLVEVAVAIAGYVFRDQVKSEFNKSFQ

hum GVFLFLVAFVGCCGACKENYCLMITFAIFLSLIMLVEVAAAIAGYVFRDKVMSEFNNNFR

pan GVFLFLVAFVGCCGACKENYCLMITFAIFLSLIMLVEVAAAIAGYVFRDKVMSEFNNNFR

* ::*:*:*.***** **.**:: ***::**:*:***:*.*******:*:* * :::.:

gal DTMRKYGEDVPLTEAVDKLQEDFHCCGANNYTDWATLERFRANNTVPRSCCRVNTTTCNI

mus QQMQNYLKDNKTATILDKLQKENNCCGASNYTDWENIPGMA-KDRVPDSCCINITVGCGN

hum QQMENYPKN**N**HTASILDRMQADFKCCGAA**N**YTDWEKIPSMS-KNRVPDSCCI**N**VTVGCGI

pan QQMENYPKNNHTASILDRMQADFKCCGAANYTDWEKIPSMS-KNRVPDSCCVNVTVGCGI

: *.:* :: : :*::* : :**** ***** .: : :: ** *** *. *.

gal NPTPSTIYEEGCQKGIEVWMKKNILIVAAVALGIAFFEILGIIFTCCLMKGIRSGYEVM

mus DFKESTIHTQGCVETIAIWLRKNILLVAAAALGIAFVEVLGIIFSCCLVKSIRSGYEVM

hum NFNEKAIHKEGCVEKIGGWLRKNVLVVAAAALGIAFVEVLGIVFACCLVKSIRSGYEVM

pan NFNEKAIHKEGCVEKIGGWLRKNVLVVAAAALGIAFVEVLGIVFACCLVKSIRSGYEVM

: . .:*: :** : * *::**:*:***.******.*:***:*:***:*.********

1. ENDOTHELIAL PROTEIN C RECEPTOR

gal ----MLRL--LLLCAALGC-GAGGDAPLTFTMLQWTRVS-NGNYAFWGNATLGGRLSHLL

mus MLTKFLPLLLLLLPGCALC---NSDGSQSLHMLQISYFQDNHHVRHQGNASLGKLLTHTL

hum MLTTLLPIL--LLSGWAFCSQDASDGLQRLHMLQISYFRDPYHVWYQGNASLGGHLTHVL

pan MLTTLLPIL--LLSGWAFCSQDASDGLQRLHMLQISYFRDPYHVWYQGNASLGGHLTHVL

:* : ** . * .*. : *** : . : . ***:** *:* *

gal EDRN----VTQVLPLEPPAGWARQQDMVANYLSYFSGIVQVFSKER--PLNYTQNLHCRL

mus EGPSQNVTILQLQPWQDPESWERTESGLQIYLTQFESLVKLVYRERKENVFFPLTVSCSL

hum EGPDT**N**TTIIQLQPLQEPESWARTQSGLQSYLLQFHGLVRLVHQER--TLAFPLTIRCFL

pan EGPDTNTTIIQLQPLQEPESWARTQSGLQSYLLQFHGLVRLVHQER--TLAFPLTIRCFL

*. . : *: * : * .* * :. : ** * .:*::. :** : : .: * *

gal GCCLFPNG----TTCSFYEVSLNGTAFLTFHVPNATWKLRWPRKDPVATFAQQELMKYSE

mus GCELPEEEEEGSEPHVFFDVAVNGSAFVSFRPKTAVWVSGSQEPSKAANFTLKQLNAYNR

hum GCELPP---EGSRAHVFFEVAV**N**GSSFVSFRPERALWQADTQVTSGVVTFTLQQLNAY**N**R

pan GCELPP---EGSRPHVFFEVAVNGTSFVSFRPERALWQADTQVTSGVVTFTLQQLNAYNR

** * *::*::**::*::*: * * . ...*: ::* *..

gal TTHHLQHFLNTTCVDILWAQ---SPQTGKHRGRSHAPLVLGLILGVSAVVGMAVGIFLCT

mus TRYELQEFLQDTCVEFLENHITTQNMKGSQTGRSYTSLVLGILMGCFIIAGVAVGIFMCT

hum TRYELREFLEDTCVQYVQKHISAENTKGSQTSRSYTSLVLGVLVGSFIIAGVAVGIFLCT

pan TRYELREFLEDTCVQYVQKHISAENTKGSQTSRSYTSLVLGILVGSFIIAGVAVGIFLCT

* :.*:.**: ***: : : . .*.: .**:: ****:::* :.*:*****:**

gal GGSC-

mus SGRRC

hum GGRRC

pan GGRRC

.*

1. TETRASPANIN 4

gal ---------------------------------MAHNCLQCIKYLMFVFNLLFWLGGCGI

mus MNRPSNLFFPGYLAQTRRLRARLLVATELKRCSMARGCLQGVKYLMFAFNLLFWLGGCGV

hum ---------------------------------MARACLQAVKYLMFAFNLLFWLGGCGV

pan ---------------------------------MARACLQAVKYLMFAFNLLFWLGGCGV

**: *** :*****.***********:

gal LGVGIWLAVTQGNFATLSSSFPSLSAANLLIVTGTFVMIIGFVGCIGAIKENKCLLLSFF

mus LGVGIWLAATQGNFATLSSSFPSLSAANLLIVTGTFVMAIGFVGCIGALKENKCLLLTFF

hum LGVGIWLAATQGSFATLSSSFPSLSAANLLIITGAFVMAIGFVGCLGAIKENKCLLLTFF

pan LGVGIWLAATQGSFATLSSSFPSLSAANLLIITGAFVMAIGFVGCLGAIKENKCLLLTFF

********.***.******************:**:*** ******:**:********:**

gal IMLLIVFLLELTVVILFFVYTDKIDKYAQRDLKKGLHLYGTDGNIGLTNAWSIIQTDFRC

mus VLLLLVFLLEATIAVLFFAYSDKIDSYAQQDLKKGLHLYGTQGNVGLTNAWSIIQTDFRC

hum LLLLLVFLLEATIAILFFAYTDKIDRYAQQDLKKGLHLYGTQGNVGLTNAWSIIQTDFRC

pan LLLLLVFLLEATIAILFFAYTDKIDRYAQRDLKKGLHLYGTQGNVGLTNAWSIIQTDFRC

::**:***** *:.:***.*:**** ***:***********:**:***************

gal CGVSNYTDWFEVYNTTRVPDSCCLEFSENCGLHSPGTWWKDSCYEAVKIWLQENLLAVGI

mus CGVSNYTDWFEVYNATRVPDSCCLEFSDSCGLHEPGTWWKSPCYETVKAWLQENLLAVGI

hum CGVS**N**YTDWFEVY**N**ATRVPDSCCLEFSESCGLHAPGTWWKAPCYETVKVWLQENLLAVGI

pan CGVSNYTDWFEVYNATRVPDSCCLEFSESCGLHAPGTWWKAPCYETVKVWLQENLLAVGI

**************:************:.**** ****** ***:** ***********

gal FGLCTVLVQILGLTFAMTMYCQVVKADTYCA

mus FGLCTALVQILGLTFAMTMYCQVVKADTYCA

hum FGLCTALVQILGLTFAMTMYCQVVKADTYCA

pan FGLCTALVQILGLTFAMTMYCQVVKADTYCA

*****.*************************

1. T-cell antigen CD7

gal MQWTSPLPFVYLLFLLLPFFRGQNGEENKITDWSTDIISVWEGDTINLTCSKNSSENEVG

mus ------MTQQAVLALLLTLAGILPGP----------------------------------

hum ---MAGPPRLLLLPLLLALARGLPGA----------------------------------

pan ---MAGPPRLLLLPLLLALARGLPGA----------------------------------

:* *** : *

gal TQLRIRAPPTNIIYFSTKYAPYIDPAFANRTKYLNEGTNLTITVHNVQKSDSNIYLCIHY

mus ------------------------------------------------------------

hum ------------------------------------------------------------

pan ------------------------------------------------------------

gal SVVNERHFKEFGKAVILVVKAKSSGVIEQSPLSVHAQQGESINITCVLKSSNGDEEIMLL

mus ---------------------LDAQDVHQSPRLTIASEGDSVNITCST--RGHLEGILMK

hum ---------------------LAAQEVQQSPHCTTVPVGASV**N**ITCST--SGGLRGIYLR

pan ---------------------LAAQEVQQSPHCTTVPVGASVNITCST--SGGLHGIYLR

: :.*** . . * *:**** . . * :

gal RLHRQLEKVLYVLSQNTTTISPTFANRLEYSKQEKKLVITLHNLQENDTDIYVCAAVLKN

mus KIWPQAYNVIYFEDRQEPTVDRTFSGRINFSGSQKNLTITISSLQLADTGDYTCEAVRKV

hum QLGPQPQDIIYYEDGVVPTTDRRFRGRIDFSGSQD**N**LTITMHRLQLSDTGTYTCQAITEV

pan QLGPQPQDIIYYEDGVVPTTDRRFRGRIDFSGSQDNLTITMHRLQLSDTGTYTCQAITEV

:: * .::* . * . * .*:::* .:.:*.**: ** **. *.* *: :

gal SSFSVSESGTMVLVKGEKQTACSIS-----------------------------------

mus SAR---GLFTTVVVKEKSSQEAYRSQEP--------------------------------

hum NVY---GSGTLVLVTEEQSQGWHRCSDAPPRASA--------LPAPPTGSALPDPQTASA

pan NVY---GSGTLVLVTEEQSQGWHRCSDAPPRAFALPDPTRSALPVPPTASALPDPPAASA

. * *:*. :.. .

gal --------SLAIYIP-IIVVALLLSALICFILNRINKKKFVQKR----KIDGVYEDMSFS

mus ---LQTSFSFPAAIAVGFFFTGLLLGVVCSMLRKIQIKKLCASGI-KESPCVVYEDMSYS

hum LPDPPAASALPAALAVISFLLGLGLGVACVLA-RTQIKKLCSWRDKNSAACVVYEDMSHS

pan LPDPPTASALPAALAVISFLLGLGLGVACVLA-RTQIKKLCSWRDKSSAACVVYEDMSHS

:: : .. * .: * : : : **: ******.*

gal SRSNTLVRN---

mus NRKTPCIPNQYQ

hum RCNTLSSPNQYQ

pan RCNTLSSPNQYQ

.. *

1. Extracellular SUPEROXIDE DISMUTASE

gal MLLLLSLVTGLALSASDVVT--E---------TG--------ADPSSALLHDIQRKVNDL

hum MLAL--LCSCLLLAAGAS--------DAWTGEDSAEP-----NSDSAEWIRDMYAKVTEI

pan MLAL--LCSCLLLAAGAS--------DAWTGEDSAEP-----NSDSAEWIRDMYAKVTEI

mus MLAF--LFYGLLLAACGSVTMSDPGESSFDLADRLDPVEKIDRLDLVEKIGDTHAKVLEI

** : * * *:* : * ** ::

gal WQSLLYPVMADNETDGLTYATCEVKPSSKIDADKPQVTGQVLFRQHYSQGKLEAIFHLDG

hum WQEVMQR---RDD-DGALHAACQVQPSATLDAAQPRVTGVVLFRQLAPRAKLDAFFALEG

pan WQEVMQR---RDD-DGALHAACQVQPSATLDAAQPRVTGVVLFRQLAPRAKLDAFFALEG

mus WMELGRR---REVDAAEMHAICRVQPSATLPPDQPQITGLVLFRQLGPGSRLEAYFSLEG

* .: : . :* *.*:**:.: :*::** ***** .:*:* * *:*

gal FPLDNNQSGRAIHIHELGDLSDGCNSAGGHYNPFGVNHPRHPGDFGNFSPKDGKIRKYKP

hum FPTEP**N**SSSRAIHVHQFGDLSQGCESTGPHYNPLAVPHPQHPGDFGNFAVRDGSLWRYRA

pan FPTEPNSSSRAIHVHQFGDLSQGCESTGPHYNPLAVPHPQHPGDFGNFAVRDGSLWRYRA

mus FPAEQNASNRAIHVHEFGDLSQGCDSTGPHYNPMEVPHPQHPGDFGNFVVRNGQLWRHRV

** : * *.****:*::****:**:*:* ****: * **:******** ::*.: :::

gal NLFATMFGPYSILGRSVVIHEQEDDMGKGNNKASLENGNAGKRLACCVIGICNKNLWEEK

hum GLAASLAGPHSIVGRAVVVHAGEDDLGRGGNQASVENGNAGRRLACCVVGVCGPGLWERQ

pan GLAASLAGPHSIVGRAVVVHAGEDDLGRGGNQASVENGNAGRRLACCVVGVCGPGLWERQ

mus GLTASLAGPHSILGRSVVVHAGEDDLGKGGNQASLQNGNAGRRLACCVVGTSSSAAWESQ

.* *:: **:**:**:**:* ***:*:*.*:**::*****:******:* .. ** :

gal QSEAMDRKKRWRRTRLS--

hum AREHSERKKRRRESECKAA

pan AREHSERKKRRRESECKAA

mus TK---ERKKRRRESECKTT

:**** *.:. .

1. Tumor necrosis factor ligand superfamily member 14

gal MQQNLNYMYPQIFWVDGCADASTSCHPASPVAPFPPPVPDRRKKPKSSKERSSVSFLVIF

hum --MEESVVRPSVFVVDGQTDI-----------------PFTRLGRSH--RRQSCSVARVG

pan --MEESVVRPSVFVVDGQTDI-----------------PFTRLGRSR--RRQSCSVARVG

mus ---MESVVQPSVFVVDGQTDI-----------------PFRRLEQNH--RRRRCGTVQVS

. : *.:* *** :* * * . .* . :

gal LLILLALTGAGLSMFQIFH--LEKELAELRESVNADRIPPALEKLIGQESKPVKKEARKA

hum LGLLLLLMGAGLAVQGWFLLQLHWRLGEMVTRLP-DGPAGSWEQLIQERR---SHEVNPA

pan LGLLLLLMGAGLAVQGWFLLQLHWRLGEMVTRLP-DGHAGSWEQLIQERR---SHEVNPA

mus LALV-LLLGAGLATQGWFLLRLHQRLGDIVAHLP-DGGKGSWEKLIQDQR---SHQANPA

* :: * ****: * *. .*.:: : * : *:** :. .::.. *

gal AHLTGNP---TQRNLPLEWEPVSGHAFTSGIQYRDQGLVINETGLYFVYSNVLFRGRHCD

hum AHLTGA**N**SSLTGSGGPLLWETQLGLAFLRGLSYHDGALVVTKAGYYYIYSKVQLGGVGCP

pan AHLTGANSSLTGSGGPLLWETQLGLAFLRGLSYHDGALVATKAGYYYIYSKVQLGGVGCP

mus AHLTGANASLIGIGGPLLWETRLGLAFLRGLTYHDGALVTMEPGYYYVYSKVQLSGVGCP

***** . ** ** * ** *: *:* .** : * *::**:* : * *

gal NE-----VLTHVVYKRNPASPGSHVLMEDKRIN---YCTKEKMWARKSYLGALFKLRERD

hum LGLAS--TITHGLYKRTPRYPEELELLVSQQSPCGRATSSSRVWWDSSFLGGVVHLEAGE

pan LGLAS--TITHGLYKRTPRYPEELELLVSQQSPCGRATSSSRVWWDSSFLGGVVHLEAGE

mus QGLANGLPITHGLYKRTSRYPKELELLVSRRSPCGRAN-SSRVWWDSSFLGGVVHLEAGE

:** :***. * . *: .:: ..::* .*:**.:.:*. :

gal SLYVNVSRIDLVNFE-ESKTFFGLFKL

hum KVVVRVLDERLVRLRDGTRSYFGAFMV

pan EVVVRVLDERLVRLRDGTRSYFGAFMV

mus EVVVRVPGNRLVRPRDGTRSYFGAFMV

.: *.* **. . ::::** * :

1. CD 48 antigen

gal MQQHG-GALHALEMLLCLVLGAADSGQGTGECRNRTVLTGSDLHLLLEEQLPPDWSAVDW

mus MCFIRQGWCLVLEL-LLLPLGT---GFQGHSIPDINATTGSNVTLKIHKDPLGPYKRITW

hum MCSRGWDSCLALEL-LLLPLSLLVTSIQGHL-VHMTVVSGS**N**VTL**N**ISESLPENYKQLTW

pan MCSRGWDSCLALEL-LLLPLSLLVTSIQGHL-VHMTVVSGSNVTLNISESLPENYKQLTW

* . .**: * * *. . . .. :**:: * : :. :. : *

gal KVTLEAQPRQRILTVRKDR-VDRANSTLSQRATFHWEPLSLQIRAVTQADSGIYYAEIVK

mus LHTKNQK----ILEYNYNSTKTIFESEFKGRVYLEENNGALHISNVRKEDKGTYYMRVLR

hum FYTFDQK----IVEWDSRK-SKYFESKFKGRVRLDPQSGALYISKVQKED**N**STYIMRVLK

pan FYTFDQK----IVEWDSRK-SKYFESKFKGRVRLDPQSGALYISKVQKEDNSIYIMRVLK

* : : *: :* :. *. :. : :* * * : *.. * .:::

gal STGSVSNKCFHVSVWEPVGSPRLETLVLQEEQGRCHLQLSCTVPGATAVSYSWSRDSDPL

mus ETEN--ELKITLEVFDPVPKPSIEINKTEASTDSCHLRLSCEVKDQ-HVDYTWYESSGPF

hum KTGNEQEWKIKLQVLDPVPKPVIKIEKIEDMDDNCYLKLSCVIPGE-SV**N**YTWYGDKRPF

pan KTGNEQEWKIKLQVLDPVPKPVIKIEKIEDMDDNCYLKLSCVIPGK-SVNYTWYGDKRPF

.* . : : :.* :** .* :: : . *:*:*** : . *.*:* .. *:

gal GNQSVLLV-----SEYVQPVLYVCNVSNPASWSTASIDMATACTQKGLFGA---VPWWAV

mus PKKSPGYVLDLIVTPQNKSTFYTCQVSNPVSSKNDTVYFTLPCDLARSSGVCWTATWLVV

hum PKELQ**N**SVLETTLMPH**N**YSRCYTCQVSNSVSSKNGTVCLSPPCTLARSFGVEWIASWLVV

pan PKELQNSVLETTLMPHNYSRCYTCQVSNSVSSKNGTVCLSPPCTLARSFGVEWIASWLVV

:: * *.*:*** .* .. :: :: * *. . * .*

gal TVLLVLAVCIAGSISFWCWRRRGKDYPAEHTEPPLTVYEEVGRVRTGQEPNRNSEAHAVG

mus TTLIIHRILLT-------------------------------------------------

hum TVPTILGLLLT-------------------------------------------------

pan TVPTILGLLLT-------------------------------------------------

*. : : ::

gal NTIYAMVHPKEQKPKSPQNPESCTIYSTVQHRMKQPPPLSPDPRSALQSPSFRRKKLDPV

mus ------------------------------------------------------------

hum ------------------------------------------------------------

pan ------------------------------------------------------------

gal LVSTAYMEVTAPLRRGYTPSQRSSPSPMDHHNS

mus ---------------------------------

hum ---------------------------------

pan ---------------------------------

1. COMPLEMENT C1Q SUBCOMPONENT Subunit A

gal -MQLSLWLVTSSLAAVLGMEQLEDGVCRAPNGKDGFPGIPGLDGRPGQKGDVGEPGRSAP

mus METSQGWLVACVLTMTL-VWTVAEDVCRAPNGKDGAPGNPGRPGRPGLKGERGEPGAAGI

hum MEGPRGWLVLCVLAISL-ASMVTEDLCRAPDGKKGEAGRPGRRGRPGLKGEQGEPGAPGI

pan MEGPRGWLVVCVLAISL-ASMVTEDLCRAPDGKKGEAGRPGRPGRPGLKGEQGEPGAPGI

*** . *: * : :.:****:**.* * ** **** **: **** .

gal RTGIRGPKGDKGESGLPGIPGNRGYHGPPGLPGMPGMPGLKGAKGNAGSFQQQQHPAFSA

mus RTGIRGFKGDPGESGPPGKPGNVGLPGPSGPLGDSGPQGLKGVKGNPGNIRDQPRPAFSA

hum RTGIQGLKGDQGEPGPSGNPGKVGYPGPSGPLGARGIPGIKGTKGSPGNIKDQPRPAFSA

pan RTGIQGLKGDQGEPGPSGNPGKVGYPGPSGPLGARGIPGIKGTKGSPGNIKDQPRPAFSA

****:* *** ** * * **: * ** * * * *:**.**. *.:::* :*****

gal SRTMSLFRGTTVVFNNIITNEENSYSPQTGEFTCSIPGIYYFAYQVVSNGDLCLSITKN-

mus IRQNPMTLGNVVIFDKVLTNQESPYQNHTGRFICAVPGFYYFNFQVISKWDLCLFIKSSS

hum IRRNPPMGGNVVIFDTVITNQEEPYQ**N**HSGRFVCTVPGYYYFTFQVLSQWEICLSIVSSS

pan IRRNPPMGGNVVIFDTVITNQEEPYQNHSGRFVCTVPGYYYFTFQVLSQWEICLSIVSSS

* *..*:*:.::**:*. *. ::*.* *::** *** :**:*: ::** * ..

gal ---AERVVSFCDNNSRNILQVNSGSSVLSLAMGDRVSVNTIPTKGNLIYHGSEADSVFSG

mus GGQPRDSLSFSNTNNKGLFQVLAGGTVLQLRRGDEVWIEKDPAKGR-IYQGTEADSIFSG

hum RGQVRRSLGFCDTTNKGLFQVVSGGMVLQLQQGDQVWVEKDPKKGH-IYQGSEADSVFSG

pan RGQVRRSLGFCDTTNKGLFQVVSGGMVLQLQRGDQVWVEKDPKKGH-IYQGSEADSVFSG

. :.*.:...:.::** :*. **.* **.* ::. * **. **:*:****:***

gal FMLYPQMG

mus FLIFPSA-

hum FLIFPSA-

pan FLIFPSA-

*:::*.

1. CHYMASE

gal ------MRRPHCPLL---LALLLLLSCLRADGSALRGQIIGGHEAKPHSHPYMAYLKLR-

mus MKPAAALRRPSERMHLLTLHLLLLLLG----SSTKAGEIIGGTECIPHSRPYMAYLEIVT

hum -------------MLLLPLPLLLFLLC----SRAEAGEIIGGTECKPHSRPYMAYLEIVT

pan -------------MLLLPLPLLLFLLC----SRAEAGEIIGGTECKPHSRPYMAYLEIVT

: * ***:* . : *:**** *. ***:******::

gal ----MSACGGFLVAPDWVMTAAHCLGGNITVILGAHDIYEPEQSQQVRGVLKYYPHPAYD

mus SENYLSACSGFLIRRNFVLTAAHCAGRSITVLLGAHNKTSKEDTWQKLEVEKQFLHPKYD

hum SNGPSKFCGGFLIRRNFVLTAAHCAGRSITVTLGAH**N**ITEEEDTWQKLEVIKQFRHPKY**N**

pan SNGPSKFCGGFLIRRNFVLTAAHCAGRSITVTLGAHNIAEEEDTWQKLEVIKQFRHPKYN

. *.***: ::*:***** * .*** ****: . *:: * * * : ** *:

gal PNTMANDIMLLKLTAKVKLNKYVRTIALPKTSSDLPTGTSCTIAGWGLIDEDERTSK-LF

mus ENLVVHDIMLLKLKEKAKLTLGVGTLPLSANFNFIPPGRMCRAVGWGRTNVNEPASDTLQ

hum TSTLHHDIMLLKLKEKASLTLAVGTLPFPSQFNFVPPGRMCRVAGWGRTGVLKPGSDTLQ

pan TSTLHHDIMLLKLKEKASLTLAVGTLPFSSQFNFVLPGRMCRVAGWGRTGVLKPGSDTLQ

. : :*******. *..*. * *: : . : * * .*** . : *. *

gal ETEVSIYSRRKCILFYPHLDNGMVCAGSFHEMKDSSQGDSGGPLVCNKVAQGVVSFGY--

mus EVKMRLQEPQACKHFTSFRHNSQLCVGNPKKMQNVYKGDSGGPLLCAGIAQGIASYVHRN

hum EVKLRLMDPQACSHFRDFDHNLQLCVGNPRKTKSAFKGDSGGPLLCAGVAQGIVSYGRSD

pan EVKLRLMDPQACSHFRDFDHNLQLCVGNPRKTKSAFKGDSGGPLVCAGVAQGIVSYGRSD

*.:: : . : * * . .* :*.*. :: :. :*******:* :***:.*:

gal DSPPGVYARIANYLPWIKKVMKK-

mus AKPPAVFTRISHYRPWINKILREN

hum AKPPAVFTRISHYRPWINQILQAN

pan AKPPAVFTRISHYRPWINQILQAN

.**.*::**::* ***:::::

1. Cysteine rich secretory protein 1

gal ------MILPAVLLC-LAAVLSPSAGEDLSYVPLPLLTASSS-QQIPEASLLLSTNRTDQ

hum ------MEIKHLLFLVAAACLL------------PMLSMK-KKSARDQFNKLVTD-LPNV

pan ------MEIKRLLFLVAAACLL------------PMLSMK-KKSARDQFNKLVTD-LPNV

mus METSISMAVKFILLLFVA-AFV------------PVVTIRPLKLDRALYNKLITESQTEP

* : :*: * : *::: . *:: :

gal QKLIVDKHNALRRRVSPPARNMLRMEWSPQAAVNAQNWANQCSLSHSPPNQREI-GQPCG

hum QEEIVNIHNALRRRVVPPASNMLKMSWSEEAAQNARIFSKYCDMTESNPLERRLPNTFCG

pan QEEIVNIHNALRRRVVPPASNMLKMSWSEEAAENARIFSKYCDMTESNPLERRLPNTFCG

mus QEEIVNTHNAFRRKVSPPARNMLKVSWSSAAAENARILARYCDKSDSDSLERRLPNTFCG

*: **: ***:**:* *** ***::.** ** **: :. *. :.* :*.: . **

gal ENLYMSTAPSSWSDSIQAWFDEEKDFKYGSGATTAN-AVIGHYTQLVWYNSYQVGCAVAY

hum ENMHMTSYPVSWSSVIGVWYSESTSFKHGEWTTTDDDITTDHYTQIVWATSYLIGCAIAS

pan ENMHMTSYPVSWSSVIGVWYSESTSFKHGEWTTTDDDITTDHYTQIVWATSYLIGCAIAS

mus ENMLMEHYPSSWSKVIEIWFNESKYFKYGEWPSTDDDIETDHYTQMVWASTYLVGCDVAA

**: * * ***. * *:.*.. **:*. :* : .****:** .:* :** :*

gal CPER-TFKYFYVCHYCPAGNIIGSIETPYKEGQPCGDCPSACDNGLCTNPCKYRDVYSNC

hum CRQQGSPRYLYVCHYCHEGNDPETKNEPYKTGVPCEACPSNCEDKLCTNPCIYYDEYFDC

pan CRQKGSPRYLYVCHYCHEGNDPETKNEPYKTGVPCEACPSNCEDKLCTNPCIYYDEYFDC

mus CRRQKAATYLYVCHYCHEGNHQDTLNMPYKEGSPCDDCPNNCEDGLCTNPCIYYDEYNNC

* .: : *:****** ** : : *** * ** **. *:: ****** * * * :*

gal PEMAKAYGCEHSFIKTNCLASCRCLSEII

hum DIQVHYLGC**N**HSTTILFCKATCLCDTEIK

pan DIQVHYLGCNHSTTILFCKATCLCDTEIK

mus DTQVKLYGCSHPAVQPFCKASCLCTTEIK

.: **.* * *:* * :**

1. Tumor necrosis factor ligand superfamily member 12

gal MNEAYSPAAPRPMGS-TSPSTMKMFMCFLSVFMVVQTIGTVLFCLYLHMKMDKMEEVLSL

hum MAARRS---QRRRGRRGEPGTALLV-------PLALGLGLALACLGLLLAVVSLGSRASL

pan MAARRS---QRRRGRRGEPGTALLV-------PLALGLGLALACLGLLLAVVSLGSRASL

mus MAARRS---QRRRGRRGEPGTALLA-------PLVLSLGLALACLGLLLVVVSLGSWATL

* * * * .*.* : :. :* .* ** * : : .: . :*

gal NEDYIFLRKVQKCQTGE-DQKSTLLDCEKVLKGFQDLQCKDRTASEELPKFEMH---RGH

hum SAQE----PAQEELVAEEDQDPSEL------------NPQTEESQDPAPFLNRLVRPRRS

pan SAQE----PAQEELVAEEDQDPSEL------------NPQTEESQDPAPFLNRLVRPRRS

mus SAQE----PSQEELTAEDRREPPEL------------NPQTEESQDVVPFLEQLVRPRRS

. : *: ..* :. * : : . :.: * :: *

gal EHPHLKSRNETSVAEEKRQPIATHLAGVKSNTTVRVLKWMTTSYAPTSSL-ISYHEGKLK

hum APKGRKTRARRAIAAHYEVHPRPGQDGAQAGVDGTVSGWEEARI**N**SSSPLRYNRQIGEFI

pan APKGRKTRARRAIAAHYEVHPRPGQDGAQAGVDGTVSGWEEARINSSSPLRYNRQIGEFI

mus APKGRKARPRRAIAAHYEVHPRPGQDGAQAGVDGTVSGWEETKINSSSPLRYDRQIGEFT

*:* . ::* . . *.::.. * * : :* * . : *::

gal VEKAGLYYIYSQVSFCTKAAASAPFTLYIYLYLPMEEDRLLMKGLDTHS---TSTALCEL

hum VTRAGLYYLYCQVHFDEGKAVYLKLDLLVDGV-------LALRCLEEFSATAASSLGPQL

pan VTRAGLYYLYCQVHFDEGKAVYLKLDLLVDGV-------LALRCLEEFSATAASSLGPQL

mus VIRAGLYYLYCQVHFDEGKAVYLKLDLLVNGV-------LALRCLEEFSATAASSPGPQL

* :*****:*.** * *. : * : * :: *: .* :*: :*

gal QSIREGGVFELRQGDMVFVNVTDSTAVNVNPGNTYFGMFKL-

hum RLCQVSGLLALRPGSSLRIRTLPWAHLKAAPFLTYFGLFQVH

pan RLCQVSGLLALRPGSSLRIRTLPWAHLKAAPFLTYFGLFQVH

mus RLCQVSGLLPLRPGSSLRIRTLPWAHLKAAPFLTYFGLFQVH

: : .*:: ** *. : :.. : ::. * ****:*::

1. Lymphocyte function-associated antigen3

mus ----------------------------------------------MAQRHLWIWFLCLQ

hum -----------------MVAGSDA--G------RALGVLSVVCLLH-CF--GFISCFSQQ

pan -----------------MVAGSDA--G------RALGVLGVVCLLH-CF--GFISCFPQQ

gal MTEELYEKFLAPDEPCPLLSHQHPPRGGSLSEEGCLDVSDFGCQLSSCHRTDPLHRFHSN

. : : :

mus TWSEAAGKDADPVVMNGILGESVTFLLNIQEPKKIDNIAWTSQSSVAF-IKPGVNKAEVT

hum IYGVVY-------------G**N**-VTFHVPSNV--PLKEVLWKKQKDKVAELENSEFRAFSS

pan IYGVVN-------------GS-VTFHVPSNV--PLKEVLWKKQKDKVAELENSEFRAFSS

gal RWNLTS------------CGTSVASSECSEE--LFSSVSVGDQDDCYSLLDDQEFTSFDL

:. . * *: : :..: .*.. :. :

mus ITQGTYKGRIEIIDQKYDLVIRDLRMEDAGTYKADINEENEETITKIYYLHIYRRLKTPK

hum FKNRVYLDTV-----SGSLTIY**N**LTSSDEDEYEME--S-P**N**ITDTMKFFLYVLESLPSPT

pan FKNRVYLDTV-----SGSLTIYNLTSSDEDEYEME--S-PNITDTMKFFLYVLESLPSPT

gal FPEGSVCSDV-----SSSISAYWDWSDSEFEWQLP--G-SDITSGSDVLSDVIPSIP---

: : . : . .: .. :: : * : :

mus ITQSLISSLNNTCNITLTCSVE----KEEKDVTYSWSPFGEKSNVLQI-------VHSPM

hum LTCALT-----**N**GSIEVQCMIPEHYNSHRGLIMYSWDCPMEQCKR**N**STSIYFK--MENDL

pan LTCALT-----NGSIEVQCMIPEHYNSHRGLIIYSWDCPMEQCKRNSTSIYFK--MENDL

gal ---------------SSPCLLPKKKNKHRNLDELPWSAMTNDEQVEYI-EYLSRKVSTEM

* : ... *. :. : : . :

mus DQKLTYTC-TAQNPV---SNSSDSVTVQQPCTDTPSFHP-RHAV-LPGGLAVLFLLILIP

hum PQKIQCTL---SNPL---F**N**TT-SSIILTTCIPSSGHSRHRYAL-IPIPLAVITTCIV--

pan PQKIQCTL---SNPL---FNKT-SSISLTTCIPSSGHSRHRYAL-IPVPLAVITTCIV--

gal GLREQLDIIKIIDPTAQISPTDSEFIIELNCLTDEKLKQVRNYIKEHGPRQ--RSARE--

: :* . . * * :

mus MLAFLFRLYKRRRDRIVLEADDVSKKTVYAVVSRNAQPTESRIYDEIPQ--SKMLSCKKD

hum --LYMNGILKCDRKPDRTNS-N--------------------------------------

pan --LYMNGILKCDRKPDRTNS-N--------------------------------------

gal --NWKRSSYSCASTSGVSGA-SVSSSSAS-MVSS-ASSSGSSVANSASNSSANMSRAHSD

: . : .

mus PVT------TIYSSVQ--LSEKMKETNMKDRS-----------LPKALGNEIVV------

hum ------------------------------------------------------------

pan ------------------------------------------------------------

gal SNLSTSAAERIRDSKKRSKQRKLQQKALRKRQLKEQRQARKERLSGLFLNEEVLSLKVTE

mus -----------

hum -----------

pan -----------

gal EDHEGDVDVLM

1. Azurocidin

gal ------------------MLAALLILSLGGSALGPAGAHSSWVIGGKAAVPHSRPFIASI

mus MSGSYPSPKGIHPFLLLAL-------VVGG------AVQASKIVGGHEARPHSRPYVASL

hum ----------MTRLTVLALLAGLLASSRAG------SSPLLDIVGGRKARPRQFPFLASI

pan ----------MTRLTVLALLAGLLASSRAG------SSPLVDIVGGRKARPRQFPFLASI

: .* . ::**: * *:. *::**:

gal Q---MDGQHFCGGFLVWPRWVMTAAHCPVPRREPSVRVVLGAHSLEQPEE-SQQVFGVEE

mus QLSRFPGSHFCGGTLIHPRFVLTAAHCLQDISWQLVTVVLGAHDLLSSEP-EQQKFTI-S

hum Q---NQGRHFCGGALIHARFVMTAASCFQSQNPGVSTVVLGAYDLRRRERQSRQTFSI-S

pan Q---NQGRHFCGGALIHARFVMTAASCFRSQNPGVSTVVLGAYDLRQRERQSRQTFSI-S

* * ***** *: *:*:*** * *****:.* * .:* * : .

gal SIAHPLYNPRTVDNDIRMLRLNHTATLNAFVKRIRLPRPRIDLKPGTLCSVVGWGDISNY

mus QVFQNNYNPEENLNDVLLLQLNRTASLGKEVAVASLPQQDQTLSQGTQCLAMGWGRLGTQ

hum SMSENGYDPQQNLNDLMLLQLDREA**N**LTSSVTILPLPLQ**N**ATVEAGTRCQVAGWGSQRSG

pan SMSENGYDPQQNLNDLMLLQLDREANLTSSVTILPLPLQNATVEAGTGCQVAGWGSQRSG

.: . *:*. **: :*:*:: *.* * ** :. ** * . *** .

gal GERPIQLMEANTTIVKRSLCRTLWKGRVSGNMLCGASRNATLQGVCAGDSGGPLVFKGKV

mus APTPRVLQELNVTVV-TFLCREHN--------VCTL-VPRRAAGICFGDSGGPLICNGIL

hum GRLSRFPRFV**N**VTVTPEDQCRPNN--------VCTG-VLTRRGGICNGDGGTPLVCEGLA

pan GRLSRFPRFVNVTVTPEDQCRPNN--------VCTG-VLTRRGGICNGDGGTPLVCEGLA

. *.*:. ** :* *:* **.* **: :*

gal YGVVSFSGERCGDRRYPDIYTRISNYIDWVHHVVLSHRQPPGQRKDKPGPEKAGGDQGAA

mus HGVDSFVIRECASLQFPDFFARVSMYVDWIQNVLRGAEP---------------------

hum HGVASFSLGPCGR--GPDFFTRVALFRDWIDGVLNNPGPGPA------------------

pan HGVASFSLGPCGR--GPDFFTRVALFRDWIDGVLNNPGPGPGPA----------------

:** ** *. **:::*:: : **:. *: .

gal GWGRPGFPRPPPAPRGLMFNGN

mus ----------------------

hum ----------------------

pan ----------------------

1. C4-B binding protein beta chain

hum ------------------------------------------------------------

pan ------------------------------------------------------------

mus MTAWRKFKSLLLPLVLAVLCAGLLTAAKGQNCGGLVQGPNGTIESPGFPHGYPNYANCTW

gal MTAWRKFEALL------VFCAGLLAAAQGQNCGGLVQGPNGTIESPGFPHGYPNYANCTW

hum ------------------------------------------------------------

pan ------------------------------------------------------------

mus IIITGERNRIQLSFHTFALEEDFDILSVYDGQPQQGNLKVRLSGFQLPSSIVSTGSLLTL

gal IIITGERNRIQLSFHTFALEEDFDILSIYDGQPQQGNLKVRLSGFQLPSSIVSTGSILTL

hum ------------------------------------------------------------

pan ------------------------------------------------------------

mus WFTTDFAVSAQGFKAMYEVLPSHTCGNPGEILKGVLHGTRFNIGDKIRYSCLSGYILEGH

gal CFTTDFAVSAQGFKAIYEVLPSHTCGNPGEIPKGVLHGTRFNIGDKIRYSCISGYILEGH

hum ------------------------------------------------------------

pan ------------------------------------------------------------

mus AILTCIVSPGNGASWDFPAPFCRAEGACGGTLRGTSGSISSPHFPSEYDNNADCTWTILA

gal AMLTCIVSPGNGASWDFPVPFCRAEGACGGTLRGTSGTISSPHFPSEYENNADCTWTILA

hum ------------------------------------------------------------

pan ------------------------------------------------------------

mus EPGDTIALVFTDFQLEEGYDFLEISGTEAPSIWLTGMNLPSPVISSKNWLRLHFTSDSNH

gal EPGDTIALIFTDFQLEEGYDFLEISGTEAPSIWLTGMNLPSPVISSKNWLRLHFTSDSNH

hum ------------------------------------------------------------

pan ------------------------------------------------------------

mus RRKGFNAQFQVKKAIELKSRGVKMLPSKDSSHKNSVLTQGGVSLISDMCPDPGIPDNGRR

gal RRKGFNAQFQVKKAIELKSRGVKMLPSKDSNHKNSVLTQGGDAVASDTCPDPGIPENGKR

hum ------------------------------------------------------------

pan ------------------------------------------------------------

mus AGSDFRVGANVQFSCEDNYVLQGAKGITCQRVTETLAAWNDHRPICRARTCGSNLRGPSG

gal VGSDFRVGASVQFSCEDNYVLQGSKSITCQRVTDTLAAWSDHRPICRARTCGSNLRGPSG

hum ------------------------------------------------------------

pan ------------------------------------------------------------

mus VITSPNYPVQYEDNAHCVWVITTTDPDKVIKLAFEEFELERGYDTLTVGDAGKVGDTRSV

gal IITSPNYPVQYEDNAHCVWVITTVDPEKVIKLAFEEFELERGYDTLTVGDAGKVGDTRTV

hum ------------------------------------------------------------

pan ------------------------------------------------------------

mus LYVLTGSSVPDLIVSMSNQMWLHLQSDDSIGSPGFKAVYQEIEKGGCGDPGIPAYGKRTG

gal LYVLTGSSVPDLIVSMSNQMWLHLQSDDSIGSPGFKAVYQEIEKGGCGDPGIPSYGKRTG

hum ------------------------------------------------------------

pan ------------------------------------------------------------

mus SSFLHGDTLTFECQAAFELVGERVITCQKNNQWSGNKPSCVFSCFFNFTAPSGIILSPNY

gal SSFLHGDTLTFECQAAFELVGERMITCQQNNQWSGNKPSCVFSCFFNFTTPSGIILSPNY

hum ------------------------------------------------------------

pan ------------------------------------------------------------

mus PEEYGNNMNCVWLIISEPGSRIHLIFNDFDVEPQFDFLAVKDDGISDITVLGTFSGNEVP

gal PEEYGNNMNCVWLIISEPGSRIHLIFNDFDVEPQFDYLTVKDDGISDLPPLGTFSGNEVP

hum ------------------------------------------------------------

pan ------------------------------------------------------------

mus AQLASSGHIVRLEFQSDHSTTGRGFNITYTTFGQNECHDPGIPVNGRRFGDRFLLGSSVS

gal SQLASSGHIVRLEFQSDHSTTGRGFNITYTTFGQNECHDPGIPVNGRRFGDRFLLGSSVS

hum ------------------------------------------------------------

pan ------------------------------------------------------------

mus FHCDDGFVKTQGSESITCILQDGNVVWSSTVPRCEAPCGGHLTASSGVILPPGWPGYYKD

gal FHCDDGFVKTQGSESITCIMQDGNVVWSSAVPRCEAPCGGHLTASSGVILPPGWPGYYKD

hum ------------------------------------------------------------

pan ------------------------------------------------------------

mus SLNCEWVIEAKPGHSIKITFDRFQTEVNYDTLEVRDGPTSSSPLIGEYHGTQAPQFLIST

gal SLNCEWVIEARPGHSIKITFDRFQTEVNYDTLEVRDGPANSSPLIGEYHGTQAPQFLIST

hum ------------------------------------------------------------

pan ------------------------------------------------------------

mus GNYMYLLFTTDSSRASVGFLIHYESVTLESDSCLDPGIPVNGQRHGSNFGIRSTVTFSCD

gal GNYMYLLFTTDNSRSSVGFLIHYESVTLESDSCLDPGIPVNGHRHGNNFNIRSTVTFSCD

hum ------------------------------------------------------------

pan ------------------------------------------------------------

mus PGYTLSDDEPLVCEKNHQWNHALPSCDALCGGYIHGKSGTVLSPGFPDFYPNSLNCTWTI

gal PGYTLSDEEPLVCERNHQWNHALPSCDALCGGYIHGRSGTILSPGFPDFYPNSLNCTWTI

hum ------------------------------------------------------------

pan ------------------------------------------------------------

mus EVSHGKGVQMNFHTFHLESSHDYLLITEDGSFSEPVARLTGSVLPHTIKAGLFGNFTAQL

gal EVSHGKGVQLLFHTFHLESSHDYLLITEDGSFTEPVARLTGSVLPPTVKAGLFGNFTAQL

hum ------------------------------------------------------------

pan ------------------------------------------------------------

mus RFISDFSISYEGFNITFAEYDLEPCDDPGVPAFSRRIGFQFGVGDTLAFTCFQGYRLEGA

gal RFISDFSISYEGFNITFSEYDLEPCDDPGVPAFSRRIGFQFGVGDSLIFSCFPGYRLEGA

hum ------------------------------------------------------------

pan ------------------------------------------------------------

mus TKLTCLGGGRRVWSAPLPRCVAECGASVKGNEGTLLSPNFPSHYDNNHECIYKIETEAGK

gal NKLTCLGGGRRVWSAPLPRCVAECGATVSGNEGTLLSPNFPSNYDNNHECIYKIETEAGK

hum ------------------------------------------------------------

pan ------------------------------------------------------------

mus GIHLRARTFQLFEGDTLKVYDGKDSSSRSLGVFTRSEFMGLVLNSTSNHLRLEFNTNGSD

gal GIHLRARSFQLHEGDIIKVYDGKDSSSRSLGAFTKNEMVGVILNSTSNHLWIEFNTNGSD

hum ------------------------------------------------------------

pan ------------------------------------------------------------

mus TAQGFQLTYTSFDLVKCEDPGIPNYGYRIRDDGHFTDTVVLYSCNPGYAMHGSSTLTCLS

gal TDQGFQLTYTSFDLVKCEDPGIPNYGYKIRDEGHFIDTVILYSCNPGYTMHGSGIMTCLS

hum ------------------------------------------------------------

pan ------------------------------------------------------------

mus GDRRVWDKPMPSCVAECGGLVHAATSGRILSPGYPAPYDNNLHCTWTIEADPGKTISLHF

gal GDRRVWDKPLPTCIAECGGRIHAATSGRILSPGYPAPYDNNLHCTWIIEADPGKTISLHF

hum ------------------------------------------------------------

pan ------------------------------------------------------------

mus IVFDTETAHDILKVWDGPVDSNILLKEWSGSALPEDIHSTFNSLTLQFDSDFFISKSGFS

gal IVFDTEVAHDILKVWDGPIESSILLKEWSGSALPEDIHSTFNSLTLQFDSDFFISKSGFS

hum ------------------------------------------------------------

pan ------------------------------------------------------------

mus IQFSTSIASTCNDPGMPQNGTRYGDSREPGDTITFQCDPGYQLQGPAKITCVQLNNRFFW

gal IQFSTSIASTCNDPGTPQNGTRYGDSREPGDTTTFQCDPGYQLQGQAKITCVQLNNRFFW

hum ------------------------------------------------------------

pan ------------------------------------------------------------

mus QPDPPSCIAACGGNLTGPAGVILSPNYPQPYPPGKECDWRIKVNPDFVIALIFKSFSMEP

gal QPDPPTCIAACGGNLTGPAGVILSPNYPQPYPPGKECDWRIKVNPDFVIALIFKSFNMEP

hum ------------------------------------------------------------

pan ------------------------------------------------------------

mus SYDFLHIYEGEDSNSPLIGSFQGSQAPERIESSGNSLFLAFRSDASVGLSGFAIEFKEKP

gal SYDFLHVYEGEDSNSPLIGSFQGSQAPERIESSGNSLFLAFRSDASVGMSGFAIDYKEKP

hum ------------------------------------------------------------

pan ------------------------------------------------------------

mus REACFDPGNIMNGTRIGTDFKLGSTVTYQCDSGYKIVDPSSIECVTGADGKPSWDRALPA

gal REACFDPGNIMNGTRIGTDFKLGSTITYQCDSGYKIIDPSTITCVIGTDGKPAWNRALPS

hum ------------------------------------------------------------

pan ------------------------------------------------------------

mus CQAPCGGQYTGSEGVVLSPNYPHNYTAGQMCIYSITVPKEFVVFGQFAYFQTALNDLAEL

gal CNAPCGGQYTGSEGVVLSPSYPHNYTAGQTCHYSITVPKEFVVFGQFAYFQTALNDVAEL

hum ------------------------------------------------------------

pan ------------------------------------------------------------

mus FDGTHPQARLLSSLSGSHSGETLPLATSNQILLRFSAKSGASARGFHFVYQAVPRTSDTQ

gal FDGDNSQARLLSSLSGSHSGETLPLATSNQILLRFSAKSGASARGFHFVYQAVPRTSDTQ

hum ------------------------------------------------------------

pan ------------------------------------------------------------

mus CSSVPEPRYGRRIGSEFSAGSIVRFECNPGYLLQGSTAIRCQSVPNALAQWNDTIPSCVV

gal CSSVPEPRYGRRIGSEFSAGSVVRFECSPGYLLQGSKAIRCHSVPNALAQWNDTVPSCVV

hum ------------------------------------------------------------

pan ------------------------------------------------------------

mus PCSGNFTQRRGTILSPGYPEPYGNNLNCVWKIIVSEGSGIQIQVISFATEQNWDSLEIHD

gal PCSGNFTERRGTILSPGYPEPYGNSLNCVWKIIVTEGSGIQIQVISFATEHNWDSLEIYD

hum ------------------------------------------------------------

pan ------------------------------------------------------------

mus GGDMTAPRLGSFSGTTVPALLNSTSNQLCLHFQSDISVAAAGFHLEYKTVGLAACQEPAL

gal GGDMTAPRLGSFSGTTVPALLNSTSNQLYLHFHSDISVAAAGFHLEYKTVGLAACPEPVI

hum ------------------------------------------------------------

pan ------------------------------------------------------------

mus PSNGIKIGDRYMVNDVLSFQCEPGYTLQGRSHISCMPGTVRRWNYPSPLCIATCGGTLTS

gal PSNGIKSGDRYMVNDVLSFQCEPGYTLQGRSHISCMPGTVRRWNYPSPLCIAKCGGTLTN

hum ------------------------------------------------------------

pan ------------------------------------------------------------

mus MSGVILSPGFPGSYPNNLDCTWKISLPIGYGAHIQFLNFSTEANHDYLEIQNGPYHSSPM

gal MGGVILSPGFPGNYPSNLDCTWKILLPIGYGAHIQFLNFSTEANHDFLEIQNGPYHTSSM

hum ------------------------------------------------------------

pan ------------------------------------------------------------

mus MGQFSGPDLPTSLLSTTHETLIRFYSDHSQNRQGFKLSYQAYELQNCPDPPAFQNGFMIN

gal IGQFSGMELPSPLLSTTHETLVHFYSDHSENRQGFKLTYQAYELQNCPDPPPFLNGYIVN

hum ------------------------------------------------------------

pan ------------------------------------------------------------

mus SDYSVGQSISFECYPGYILLGHPVLTCQHGTDRNWNYPFPRCDAPCGYNVTSQNGTIYSP

gal SDYSVGQSVSFECYPGYVLRGQPVLTCQHGINRNWNYRFPRCEAPCGYNVSAQNGTVYSP

hum ------------------------------------------------------------

pan ------------------------------------------------------------

mus GFPDEYPILKDCLWLVTVPPGHGVYINFTLLQTEAVNDYIAVWDGPDQNSPQLGVFSGNT

gal GFPDEYPNSKDCTWLIVVPPGHGIYINFTLLQTEPVNDYIAVWDGPDHNSPQLGVFSGNT

hum ------------------------------------------------------------

pan ------------------------------------------------------------

mus ALETAYSSTNQVLLKFHSDFSNGGFFVLNFHAFQLKRCPPPPAVPQADLLTEDEDFEIGD

gal ALETAYSSTNQVLLKFHSDFSTGGFFVLNFHAYQLKKCQPPPIVPHADLFTEDDDFGIGD

hum ------------------------------------------------------------

pan ------------------------------------------------------------

mus FVKYQCHPGYTLLGSDTLTCKLSSQLLFQGSPPTCEAQCPANEVRTESSGVILSPGYPGN

gal FVKYKCHPGFTLVGQDILTCKLNTQLQFEGSSPICEAQCPANEIRTESSGVILSPGYPGT

hum ------------------------------------------------------------

pan ------------------------------------------------------------

mus YFNSQTCAWSIKVEPNFNITLFVDTFQSEKQFDALEVFDGSSGQSPLLVVLSGNHTEQSN

gal YPNSQTCSWTIKVDPGYNISIFVEMFQSEKQFDELEVFDGSSGQSPLLVALSGNHTGQLN

hum ------------------------------------------------------------

pan ------------------------------------------------------------

mus FTSRSNHLYLRWSTDHATSKKGFKIRYAAPYCSLTSTLRNGGILNKTAGAVGSKVHYFCK

gal FTSKMNQLYLRWSTDHATSKKGFKIRYSAPYCSLNPTLRNGGIVNKTGGPAGSKVHYYCK

hum ------------------------------------------------------------

pan ------------------------------------------------------------

mus PGYRMIGHSNATCRRNPVGVYQWDSMAPLCQAVSCGIPEAPGNGSFTGNEFTLDSKVTYE

gal PGYRMIGQNNATCRRHQNGMYQWDSPVPICRAVSCGIPDSPGNGSVIGNEFTLGSRLIYE

hum ------------------------------------------------------------

pan ------------------------------------------------------------

mus CNEGFKLDASQEATTVCQEDGLWSNRGKPPTCKPVPCPSIEGQLSEHVLWRLVSGSLNEY

gal CNEGFKLESSQQATAVCQEDGLWSNKGRPPVCKSVTCPNIETLLSEHVVWRLVSGSLNEY

hum ---MFF------------WCACCLMVAWRVSASD----AEHCPELPPVDNSIFVAKEVEG

pan ---MFF------------WCACCLMVAWRVSASD----AEHCPELPPVDNSIFVAKEVEG

mus GAQVLLSCSPGYFLQGQRLLQCQANGTWSTEEDRPRCKVISCGSLSFPPNGNKIGTLTIY

gal GAQVMLSCSPGYYLLGRRLIQCRTNGTWSVGNERPICKVISCGGLPSPPNGNKIGTLTVY

::: * :* . . . * * *. :.. .

hum QILGTYVCIKGYHLVGKKTLFC**N**ASKEWD**N**TTTECRLGHCPDPVLVNGEFSSSGPV**N**VSD

pan QILGTYVCIKGYHLVGKKTLFCNASKEWDNTTTECRLGHCPDPVLVNGEFSSSGPVNVSD

mus GATAIFTCNTGYTLVGSHVRECLANGLWSGSETRCLAGHCGSPDPIVNGHISGDGFSYRD

gal GATAIFTCNTGYTLVGSHVRECLANGLWSGAETQCLAGHCGSPDPIVNGHISGDGFSYRD

. :.* .** ***.:. * *. *..: *.* *** .* : . . *.. .. *

hum KITFMCNDHYILKGS**N**RSQCLEDHTWAPPFPICKSRDCDPPGNPVHGYFEGN**N**FTLGSTI

pan KITFMCNDHYILKGSNWSQCLEDHTWAPPFPICKSRDCDPPGNPVHGYFEGNNFTLGSTI

mus TVVYQCNPGFRLVGTSVRICLQDHKWSGQTPVCVPITCGHPGNPAHGLTNGTEFNLNDLV

gal TVVYQCNPGFRLVGTSVRICLQDHRWSGQTPVCVPITCGHPGNPAHGMTNGSEFNLNDVV

.:.: ** : * *:. **:** *: *:* *. ****.** :*.:*.*.. :

hum SYYCEDRYYLVGVQEQQCV-DGEWSSALPVCKLIQEAPKPECEKALLAFQESKNLCEAME

pan SYYCEDRYYLVGVQEQQCV-DGEWSSALPVCKLIQEAPKPECEKALLAFQESKDLCEAME

mus NFTCHTGYRLQGASRAQCRSNGQWSSPLPICRVVNCSDPGSVENAVRHGQ---------Q

gal NFTCNTGYLLQGASRAQCRSNGQWSNPLPNCRVVNCSDPGFVENAIRHGQ---------Q

.: *. * * *... ** :*:**. ** *:::: : *:*: * :

hum NFMQQLKESGMTMEELK---YSLEL-----KKAELKAKLL--------------------

pan NFMQQLKESGMTMEELK---YSLEL-----KKAELKAKLL--------------------

mus NFPESFEYGTSVMYHCKTGFYLLGSSALTCMASGLWDRSLPKCLAISCGHPGVPANAVLT

gal NYPESFKYGTSVAYHCKKGFYLLGSSALTCKSNGLWDRSLPKCLSISCGHPGVPANAVLS

*: :.:: . . . * * * * : *

hum ------------------------------------------------------------

pan ------------------------------------------------------------

mus GELFTYGATVQYSCKGGQILTGNSTRVCQEDSHWSGSLPHCSGNSPGFCGDPGTPAHGSR

gal GDKFTYGSIIHYSCTAGRRLIGNSTRECQEDSHWSGTLPHCSGNNPGYCDDPGIPAHGSR

hum ------------------------------------------------------------

pan ------------------------------------------------------------

mus LGDEFKTKSLLRFSCEMGHQLRGSAERTCLVNGSWSGVQPVCEAVSCGNPGTPTNGMILS

gal LGDEFKIKSLLRFSCEMGYQLRGSSERTCLLNGSWSGIQPVCEAVSCGNPGTPANGMIIY

hum ------------------------------------------------------------

pan ------------------------------------------------------------

mus SDGILFSSSVIYACWEGYKTSGLMTRHCTANGTWTGTAPDCTIISCGDPGTLPNGIQFGT

gal SDGILFSSSVIYACWEGYKTSGLTTRHCTANGTWTGTAPDCTVISCGDPGALANGIQFGN

hum ------------------------------------------------------------

pan ------------------------------------------------------------

mus DFTFNKTVSYQCNPGYLMEPPTSPTIRCTKDGTWNQSRPLCKAVLCNQPPPVPNGKVEGS

gal DFTFNKTVSYQCNPGYVMEPASSSTMRCIKDSTWNQSKPICKAITCGPPPPVLYGKVEGS

hum ------------------------------------------------------------

pan ------------------------------------------------------------

mus DFRWGASISYSCVDGYQLSHSAILSCEGRGVWKGEVPQCLPVFCGDPGTPAEGRLSGKSF

gal DYHWGASVSYSCAEGYQLSNTAILSCEGRGIWRGDIPQCLPVFCGDPGTPAEGRLNGKSF

hum ------------------------------------------------------------

pan ------------------------------------------------------------

mus TFKSEVFIQCKPPFVLVGSSRRTCQADGIWSGIQPTCIDPAHTACPDPGTPHFGIQNSSK

gal TYRSEVSFQCRPPFILIGSSRRFCQADGTWSGIQPTCIDPAHNTCTDPGTPHFGMQNSSR

hum ------------------------------------------------------------

pan ------------------------------------------------------------

mus GYEVGSTVFFRCRKGYHIQGSTTRTCLANLTWSGIQTECIPHACRQPETPAHADVRAIDL

gal GYEVGSTVFFRCRKGYHIQGSTTRSCLANLTWSGIQSECIPHACRQPETPAHVDVKAIDL

hum ------------------------------------------------------------

pan ------------------------------------------------------------

mus PAFGYTLVYTCHPGFFLAGGSEHRTCKADMKWTGKSPVCKSKGVREVNETVTKTPVPSDV

gal PTLGYTLVYTCQPGFFLAGGSEHRTCKPDMKWTGKSPICKSKGVREVNETITKTPVPSDV

hum ------------------------------------------------------------

pan ------------------------------------------------------------

mus FFINSVWKGYYEYLGKRQPATLTVDWFNATSSKVNATFTAASRVQLELTGVYKKEEAHLL

gal FFINSLWKGFYEYLGKRQPATLTVDWFNTTSSKVNATFTEASSLQLKLSGVYKKEEAHLL

hum ------------------------------------------------------------

pan ------------------------------------------------------------

mus LKAFHIKGPADIFVSKFENDNWGLDGYVSSGLERGGFSFQGDIHGKDFGKFKLERQDPSN

gal LKIFQIKGSNDIFTNKFENDNWALDGYVSSGLERGVFTYQGDIHGKDFGKFMLQRQGPLS

hum ------------------------------------------------------------

pan ------------------------------------------------------------

mus SDADSSNHYQGTSSGSVAAAILVPFFALILSGFAFYLYKHRTRPKVQYNGYAGHENSNGQ

gal ADTDLSNHYYGTNSSSVAAAILVPFFALILSGFAFYLYKHRTRPKVQYNGYAGHENSNGQ

hum ---------------------------------

pan ---------------------------------

mus ASFENPMYDTNLKPTEAKAVRFDTTLNTVCTVV

gal ASFENPMYDRNLKPTEAKAVRFDTTLNTVCTVV

1. Kunitz-type protease inhibitor 2

gal MARGRPSRQTGLWVCLVLVAGVVFGEEQETKPFGETCLEDFTAGIPGLVLDTDASVQNGA

hum ------------------------------------------------------------

pan ------------------------------------------------------------

mus ------------------------------------------------------------

gal TFLSSPRVHRSRDCMRACCKDPDCNLALVEQAPALGEDHIQGCFLLNCLYEQTFVCRFAR

hum ------------------------------------------------------------

pan ------------------------------------------------------------

mus ------------------------------------------------------------

gal KVGFLNFLKRDVYDTYHQAMQKHGSSDDRPPIARTGMDMRVQPGESVMLRGTDSTDDHGI

hum ------------------------------------------------------------

pan ------------------------------------------------------------

mus ------------------------------------------------------------

gal VAYEWKQILGDPSVEIKKLEKDQAEISNLQVGTYVFQLTVTDTAQQQDFTNITIIVLNSE

hum ----------------------MAQLCGLRRSRAFLALLGSL------LLSGVLAADRER

pan ----------------------MAQLCGLRRSRAFLALLGSL------LLSGVLAADRER

mus ----------------------MAQLCELRRGRALLALVASL------LLSGAQVASREL

*::. *: . .: * : : . . . ..

gal QTEEHCLTPKKVGWCRGSFPRWFYDPTLQQCQEFIFGGCKPNKNNYLREEECKLACRNVK

hum SIHDFCLVSKVVGRCRASMPRWWY**N**VTDGSCQLFVYGGCDGNSNNYLTKEECLKKCATVT

pan SIHDFCLVSKVVGRCRASMPRWWYNVTDGSCQLFVYGGCDGNSNNYLTKEECLKKCATVT

mus DVHESCGVSKVVGKCRASIPRWWYNITDGSCQPFVYGGCEGNGNNYQSKEECLDKCAGVT

. .: * . * ** **.*:***:*: * .** *::***. * *** :*** * *.

gal GSVSGRQMPDARE------------FNRLQKINITRKQAHCVELPDTGQCTESIPRWYYN

hum E**N**ATGDLATSRNAADSSVPSAPRRQDSEDHSSDMFNYEEYCTANAVTGPCRASFPRWYFD

pan ENATGDLATSRNAADSSVPSAPRRQDSEDHSSDMFNYEEYCAAKAVTGPCRASFPRWYFD

mus ENTTDDMARNRNGADSSVLSVPRKQSAEDLSAEIFNYEEYCVPKAVTGPCRAAFPRWYYD

..:. . . . . :: . : :*. ** * ::****::

gal PFSEKCDRFTYGGCDGNMNNFEEEEECMKSCSGVTKRDAIGRRWETFEPHSDTLSAFEVV

hum VERNSCNNFIYGGCRGNKNSYRSEEACMLRCFRQQENPPLPL---------------GSK

pan VERNSCNNFIYGGCRGNKNSYRSEEACMLRCFRQQENPPLPL---------------GSK

mus TEKNSCISFIYGGCRGNKNSYLSQEACMQHCSGKQMHPFLTP---------------GLK

:.* * **** ** *.: .:* ** * . :

gal IAVLLGICIMVVLAIIGYFFLKNRRKSSRRRQPTTATNSTLSTTEDTEHLFYNGATKPV

hum VVVLAGLFVMVLILFLGASMVYLIRVAR--RNQERALRTVWSSGDDKEQLVKNTYVL--

pan VVVLAGLFVMVLILFLGAPMVYLIRVAR--RNQERALRTVWSSGDDKEQLVKNTYVL--

mus AVILVGLFLMVLILLLGTSMVCLIRVVR--RKQERALRTVWSTADDKEQLVKNTCV---

.:* *: :**:: ::* :: * *: * .:. *: :*.*:*. * .

1. Claudin-domain containing protein 1

gal ----------------------MMDNRFATALVIACVLSLISTIYMAASLGTDFWYEYHT

mus MGGDRLENKTSVSVASWSSLNARMDNRFATAFVIACVLSLISTIYMAASIGTDFWYEYRS

hum -----------------------MDNRFATAFVIACVLSLISTIYMAASIGTDFWYEYRS

pan MGGDRLENKTSVSVASWSSLNARMDNRFATAFVIACVLSLISTIYMAASIGTDFWYEYRS

********:*****************:********::

gal LSPAENITEAGRSIWEEFVSEEADEKTYTDALFRCNGTVGLWRRCITVPKNSHWYSPPET

mus PI-QENSSDSNKIAWEDFLGDEADEKTYNDVLFRYNGSLGLWRRCITIPKNTHWYAPPER

hum PV-QE**N**SSDLNKSIWDEFISDEADEKTYNDALFRY**N**GTVGLWRRCITIPKNMHWYSPPER

pan PV-QENSSDLNKSIWDEFISDEADEKTYNDALFRYNGTVGLWRRCITIPKNMHWYSPPER

** :: .: *::*:.:*******.*.*** **::********:*** ***:***

gal ----DMTTNCISFSLSDQFMEKYVEPGNHNSGTDLNRTYLWRLQFLLPFVSLGLMCFGAL

mus TESFDVVTKCMSFTLNEQFMEKYVDPGNHNSGIDLLRTYLWRCQFLLPFVSLGLMCFGAL

hum TESFDVVTKCVSFTLTEQFMEKFVDPGNHNSGIDLLRTYLWRCQFLLPFVSLGLMCFGAL

pan TESFDVVTKCVSFTLTEQFMEKFVDPGNHNSGIDLLRTYLWRCQFLLPFVSLGLMCFGAL

*:.*:*:**:*.:*****:*:******* ** ****** *****************

gal IGLCACACRSLYPAIATGVLHFLAGLCTLGLVGCYVAGIELLHKKLPLPGDVRGEFGWSF

mus IGLCACICRSLYPTLATGILHLLAGLCTLGSVSCYVAGIELLHQKVELPKDVSGEFGWSF

hum IGLCACICRSLYPTIATGILHLLAGLCTLGSVSCYVAGIELLHQKLELPDNVSGEFGWSF

pan IGLCACICRSLYPTIATGILHLLAGLCTLGSVSCYVAGIELLHQKLELPDSVSGEFGWSF

****** ******::***:**:******** *.**********:*: ** .* *******

gal CLACVSAPLQFMAAALFIWAARTNRKEFTLLKAYRVA

mus CLACVSAPLQFMAAALFIWAAHTNRKEYTLMKAYRVA

hum CLACVSAPLQFMASALFIWAAHTNRKEYTLMKAYRVA

pan CLACVSAPLQFMASALFIWAAHTNRKEYTLMKAYRVA

*************:*******:*****:**:******

1. Major prion protein precursor

gal MARLLTTCCLLALLLAACTDVALSKKGKGKPSGGGWGAGSHRQPSYPRQPGYPHNPGYPH

hum --MANLGCWMLVLFVATWSDLGLCKK---RPKPGGWNTGGSRYPGQGSPGGNRYPPQGGG

pan --MANLGCWMLVLFVATWSDLGLCKK---RPKPGGWNTGGSRYPGQGSPGGNRYPPQGGG

mus --MANLGYWLLALFVTMWTDVGLCKK---RPKPGGWNTGGSRYPGQGSPGGNRYPPQG-G

:*.*::: :*:.*.** :*. ***.:*. * *. * : *

gal NPGYPHNPGYPHNPGYPHNPGYPQNPG-YPHNPGYPGWGQGYNPSSGGSYHNQKPWKPPK

hum GWGQPHGGGWG----QPHGGGWGQPHGGGWGQPHGGGWGQGG-----GTHSQWNKPSKPK

pan GWGQPHGGGWG----QPHGGGWGQPHGGGWGQPHGGGWGQGG-----GTHSQWNKPSKPK

mus TWGQPHGGGWG----QPHGGSWGQPHGGSWGQPHGGGWGQGG-----GTHNQWNKPSKPK

* **. *: **. .: * * :* ***** *:: : : . **

gal TNFKHVAGAAAAGAVVGGLGGYAMGRVMSGMNYHFDSPDEYRWWSENSARYPNRVYYRDY

hum TNMKHMAGAAAAGAVVGGLGGYMLGSAMSRPIIHFGSDYEDRYYRENMHRYPNQVYYRPM

pan TNMKHMAGAAAAGAVVGGLGGYMLGSAMSRPIIHFGSDYEDRYYRENMHRYPNQVYYRPM

mus TNLKHVAGAAAAGAVVGGLGGYMLGSAMSRPMIHFGNDWEDRYYRENMYRYPNQVYYRPV

**:**:**************** :* .** **.. * *:: ** ****:****

gal SSPVPQDVFVADCFNITVTEYSIGPAAKKNTSEAVAAANQTEVEMENKVVTKVIREMCVQ

hum DEYSNQNNFVHDCV**N**ITIKQHTVTTTTKGE----------**N**FTETDVKMMERVVEQMCIT

pan DQYSSQNNFVHDCVNITIKQHTVTTTTKGE----------NFTETDVKMMERVVEQMCIT

mus DQYSNQNNFVHDCVNITIKQHTVTTTTKGE----------NFTETDVKMMERVVEQMCVT

.. *: ** **.***:.:::: ::* : . .* : *:: :*:.:**:

gal QYREYRLAS-------GIQLHPADTWLAVLLLLLTTLFAMH-

hum QYERESQAYYQ--RGSSMVLFSSPP---VILLISFLIFLIVG

pan QYERESQAYYQ--RGSSMVLFSSPP---VILLISFLIFLIVG

mus QYQKESQAYYDGRRSSSTVLFSSPP---VILLISFLIFLIVG

**.. * . *. : *:**: :* :

1. HLA class II histocompatibility antigen,Dr alpha chain

gal MAVLSGAAVPLLLLGVL---GGVGAVLKPHVLLQAEFYQRSEGPDKAWAQFGFHFDADEL

mus MATIGALVLRFFFIAVLMSSQKSWAIKEEHTIIQAEFYLLPDK----RGEFMFDFDGDEI

hum MAISGVPVLGFFIIAVLMSAQESWAIKEEHVIIQAEFYLNPDQ----SGEFMFDFDGDEI

pan MAVSGVPVLGFFIIAVLMSAQESWAIKEEHVIIQAEFYLNPDQ----SGEFMFDFDGDEI

** . .: ::::.** *: : *.::***** : .:* *.**.**:

gal FHVELDAAQTVWRLPEFGRFASFEAQGALQNMAVGKQNLEVMIGNSNRSQQDFVTPELAL

mus FHVDIEKSETIWRLEEFAKFASFEAQGALANIAVDKANLDVMKERSNNTPDANVAPEVTV

hum FHVDMAKKETVWRLEEFGRFASFEAQGALANIAVDKANLEIMTKRS**N**YTPITNVPPEVTV

pan FHVDMAKKETVWRLEEFGRFASFEAQGALANIAVDKANLEIMTKRSNYTPITNVPPEVTV

***:: :*:*** **.:********** *:**.* **::* .** : * **:::

gal FPAEAVSLEEPNVLICYADKFWPPVATMEWRRNGAVVSEGVYDSVYYGRPDLLFRKFSYL

mus LSRSPVNLGEPNILICFIDKFSPPVVNVTWLRNGRPVTEGVSETVFLPRDDHLFRKFHYL

hum LTNSPVELREPNVLICFIDKFTPPVV**N**VTWLRNGKPVTTGVSETVFLPREDHLFRKFHYL

pan LTNSPVELREPNILICFIDKFTPPVVNVTWLRNGKPVTTGVSETVFLPREDHLFRKFHYL

: . *.* ***:***: *** ***..: * *** *: ** ::*: * * ***** **

gal PFVPQRGDVYSCAVRHWGAEGPVQRMWGPEVPEPPSESSATLWCAVGLAVGIAGIAAGTA

mus TFLPSTDDFYDCEVDHWGLEEPLRKHWEFEEKTLLPETKENVVCALGLFVGLVGIVVGII

hum PFLPSTEDVYDCRVEHWGLDEPLLKHWEFDAPSPLPETTENVVCALGLTVGLVGIIIGTI

pan PFLPSTEDVYDCRVEHWGLDEPLLKHWEFDAPSPLPETTENVVCALGLTVGLVGIIIGTI

*:*. *.*.* * *** : *: : * : *:. .: **:** **:.** *

gal LILRAVRRNAA-NRQPGLL

mus LIMKGIKKRNVVERRQGAL

hum FIIKGVRKSNAAERRGPL-

pan FIIKGVRKSNAAERRGPL-

:*::.::: . :*:

1. Low affinity immunoglobulin gamma Fc region receptor III-A

gal ---------------------------------------MAVSRALLL-LAQALSLAVAE

mus ------------------------------------MWQLLLPTALVLTAFSGIQ-AGLQ

hum ------------------------------------MWQLLLPTALLLLVSAGMRTEDLP

pan MGGGTGERLFTPSCLVGLVPXGLRISLVTCPLQCGIMWQLLLPTALLLLVSAGMRTEDLP

: : **:* .:

gal MALLTMDPPWSVIFQGESVTLRCQGPPVHKQQPTAWYHNGKLLEHTETNTYRIQNARYKQ

mus KAVVNLDPKWVRVLEEDSVTLRCQGTFSPEDNSIKWFHNESLIPHQD-ANYVIQSARVKD

hum KAVVFLEPQWYRVLEKDSVTLKCQGAYSPED**N**STQWFH**N**ESLISSQA-SSYFIDAATVDD

pan KAVVFLETQWYRVLEKDDVTLKCQGAYSPEDNSTQWFHNESLISSQA-SSYFIAAATVYD

*:: :: * ::: :.***:*** ::: *:** .*: .* * * :

gal NGRYECQSPGSTSSNSVTLSVSYDLLILQVPSHAVFEGELLQMQCRGWKVGSLAAVRFYR

mus SGMYRCQTALSTISDPVQLEVHMGWLLLQTTKWLFQEGDPIHLRCHSWQNRPVRKVTYLQ

hum SGEYRCQT**N**LSTLSDPVQLEVHIGWLLLQAPRWVFKEEDPIHLRCHSWKNTALHKVTYLQ

pan SGEYRCQTSLSTLSDPVQLEVHIGWLLLQAPRLVFKEQDPIHLRCHSWKNTALHKVTYLQ

.* *.**: ** *: * *.* . *:**. . * : ::::*:.*: : * : :

gal DGADITKPYTSAMQLSIPQAKAHHSGRYHCSADMHSYLSLKRRESQGLYISIKELFTSPV

mus NGKG-KKYFHENSELLIPKATHNDSGSYFCRGLIGHN----NKSSASFRISLG----DPG

hum NGKG-RKYFHHNSDFYIPKATLKDSGSYFCRGLFGSK----**N**VSSETV**N**ITITQGLAVST

pan NGKS-RKYFRHNSDFYIPKATLKDSGSYFCRGLVGSK----NVSSETVNITITQGFAVST

:* . * : :: **:*. :.** *.* . . . .* . *::

gal LNVASSAEPLEGSPLNLSCITQLSPYRPHTVLWYLFYGNSTVLQGPVTSSEYQVPAVGLM

mus SPSM--FPP--WHQI-TFCLLIGLLF--------------------------------AI

hum ISSF--FPP--GYQV-SFCLVMVLLF--------------------------------AV

pan ISSF--SPP--GYQV-SFCLVMVLLF--------------------------------AV

* : *: : :

gal DTGFYSCAVRTESSNVQKWSPRVPITIKRVPISGVSLEVWPQEGQVMEGHRLVLHCSVAT

mus DTVLYFSVRRGLQSPVADYEEPKI--------------QWSKEPQDK-------------

hum DTGLYFSVKTNIRSSTRDWKDHKF--------------KWRKDPQDK-------------

pan DTGLYFSVKTNI------------------------------------------------

** :* ..

gal GTGSISFSWHREGSAEVLGRDSRYEIPSTQQSDNGQYYCMASNGDSPARSLKVQVTVVGV

mus ------------------------------------------------------------

hum ------------------------------------------------------------

pan ------------------------------------------------------------

gal SSPFCSRVRALLLLAALGTLMLGAVVVHVLTAGSRAPKGTKQQTLMEPFAPGGDV

mus -------------------------------------------------------

hum -------------------------------------------------------

pan -------------------------------------------------------

1. **Cathepsin G**

gal MRRPHCPLLLALLLLLSCLRADGSALRGQIIGGHEAKPHSHPYMAYLKLR----MSACGG

mus --------MQPLLLLLTFIL-LQGDEAGKIIGGREARPHSYPYMAFLLIQSPEGLSACGG

hum --------MQPLLLLLAFLL-PTGAEAGEIIGGRESRPHSRPYMAYLQIQSPAGQSRCGG

pan --------MQPLLLLLAFLL-PTGAEAGEIIGGQESRPHSRPYMAYLQIQSPAGQSRCGG

: *****: : . *:****:*::*** ****:* :: * ***

gal FLVAPDWVMTAAHCLGGNITVILGAHDIYEPEQSQQVRGVLKYYPHPAYDPNTMANDIML

mus FLVREDFVLTAAHCLGSSINVTLGAHNIQMRERTQQLITVLRAIRHPDYNPQNIRNDIML

hum FLVREDFVLTAAHCWGSNI**N**VTLGAHNIQRRENTQQHITARRAIRHPQYNQRTIQNDIML

pan FLVREDFVLTAAHCWGSNINVTLGAHNVQRRENTQQHITARRAIRHPQYNQRTIQNDIML

*** *:*:***** *..*.* ****:: *.:** . : ** *: ..: *****

gal LKLTAKVKLNKYVRTIALPKTSSDLPTGTSCTIAGWGLIDEDERTSKLFETEVSIYSRRK

mus LQLRRRARRSGSVKPVALPQASKKLQPGDLCTVAGWGRVSQSRGTNVLQEVQLRVQMDQM

hum LQLSRRVRRNRNVNPVALPRAQEGLRPGTLCTVAGWGRVSMRRGTDTLREVQLRVQRDRQ

pan LQLSRRVRRNRNVNPVALPRAQEGLRPGTRCTVAGWGRVSLRRGTDTLREVQLRVQRDSQ

*:* :.: . *. :***::.. * * **:**** :. . *. * *.:: :

gal CILFYPHLD-NGMVCAGSFHEMKDSSQGDSGGPLVCNKVAQGVVSFGYD--SPPGVYARI

mus CANRFQFYNSQTQICVGNPRERKSAFRGDSGGPLVCSNVAQGIVSYGSNNGNPPAVFTKI

hum CLRIFGSYDPRRQICVGDRRERKAAFKGDSGGPLLCNNVAHGIVSYGKSSGVPPEVFTRV

pan CLRIFDSYDPRRQICVGDRRERKSAFKGDSGGPLLCNNVAHGIVSYGKSSGVPPEVFTRV

* : : . :*.*. :* * : :*******:*.:**:*:**:* . ** *::::

gal ANYLPWIKKVMKK-----------------

mus QSFMPWIKRTMRRFAPRYQRPANSLSQAQT

hum SSFLPWIRTTMRSFKLLDQMETPL------

pan SSFLPWIRRTMRSFKLLDQMETPL------

.::***: .*:

1. **Folate receptor beta**

gal ---------------MGLAVEMRAGQVLLVLLAASVVMPAKDPLLNVCMDAKHHKTKPGP

mus -----------------MAWKQTPLLLL-VYMVTTGSGRDRTDLLNVCMDAKHHKTKPGP

hum -----------------MVWKWMPLLLLLVCVATMCSAQDRTDLLNVCMDAKHHKTKPGP

pan MLPAATEVQLRLQGQKDMAWKWMPLLLLLVWVATMCSAQDRTDLLNVCMDAKHHKTKPGP

:. : :* * :.: : *****************

gal EGMLYGQCAPWKDNACCTANTSSEAHRDQSYLYNFNWNHCGVMPPKCKRHFIQDMCLYEC

mus EDKLHDQCSPWKKNACCSVNTSQELHKADSRLY-FNWDHCGKMEPACKSHFIQDSCLYEC

hum EDKLHDQCSPWKKNACCTASTSQELHKDTSRLYNFNWDHCGKMEPACKRHFIQDTCLYEC

pan EDKLHDQCSPWKKNACCTASTSQELHKDTSRLYNFNWDHCGKMEPACKRHFIQDTCLYEC

*. *:.**:***.****:..**.* *: * ** ***:*** * * ** ***** *****

gal SPNLGPWIDQADSSWRRERILHVPLCKEDCEEWWEDCKDYVTCKENWHKGWNWATGTNRC

mus SPNLGPWIQQVDQSWRKERFLDVPLCKEDCHQWWEACRTSFTCKRDWHKGWDWSSGINKC

hum SPNLGPWIQQV**N**QSWRKERFLDVPLCKEDCQRWWEDCHTSHTCKSNWHRGWDWTSGVNKC

pan SPNLGPWIQQVNQSWRKERFLDVPLCKEDCQRWWEDCHTSHTCKSNWHRGWDWTSGVNKC

********:*.:.***:**:*.********..*** *: *** :**:**:*::* *:*

gal PWGSMCRPFTQVFPSPKDLCEKIWSNSYKYTTERRGSGRCIQMWFDPAQGNPNVVVAKYY

mus PNTAPCHTFEYYFPTPASLCEGLWSHSYKVSNYSRGSGRCIQMWFDSTQGNPNEDVVKFY

hum PAGALCRTFESYFPTPAALCEGLWSHSYKVS**N**YSRGSGRCIQMWFDSAQGNPNEEVARFY

pan PAGALCRTFESYFPTPAALCEGLWSHSYKVSNYSRGSGRCIQMWFDSAQGNPNEEVARFY

* : *: * **:* *** :**:*** :. ************ :***** *.::*

gal AWKKRSSPARMEDVTPEAGRAACAVPRLTLLLALVLLTAGWGSYGRGSL

mus ASFMT--SGTVPH------AAVLLVPSLAPVLSLWLPG-----------

hum AAAMHVNAGEMLH------GTGGLLLSLALMLQLWLLG-----------

pan AAAMHVNAGEMLH------GIGGLLLSLALMLQLWLLG-----------

* . : . : *: :* * *

1. **Microfibril-associated glycoprotein 4**

gal MGNERWKTMGGASQLEDGQQEKPQRMSCGYVLCTVLLSVAVLLAVTVTGAILFMNHYHTP

hum ------------------------------------------------------------

pan ------------------------------------------------------------

mus ------------------------------------------------------------

gal VTEPPPVISTNPEEANALVTIEKADSSRINIFIDPNCPDPAGNVGRLEGLQGSLLRALAE

hum ------------------------------------------------------------

pan ------------------------------------------------------------

mus ------------------------------------------------------------

gal HDAEAKATKGQQKALLLGVADEVAKLVTHAAQLRADCDGLKKGHSAMGQELSALQSEQGR

hum ------------------------------------------------------------

pan ------------------------------------------------------------

mus ------------------------------------------------------------

gal LIQLLSESQTNMARLVSSVSDVLDTLQKERGGVRPRLKADLQRAPARG-ARPRGCSNGSR

hum -----------MKALL-ALPLLL--LL-STPPCAPQ------VSGIRGDALER--FCLQQ

pan -----------MKALL-ALPLLL--LL-STPPCAPQ------VSGIRGDALER--FCLQQ

mus -----------MKALP-ALPLMLMLLS-MPPPCAPQ------ASGIRGDALEK--SCLQQ

* * :: :* * *: : ** * : .:

gal PRDCFDIYASGQQEDGIYSIFPTHYPSGFQVYCDMTTDGGGWTVFQRREDGSVNFFRGWE

hum PLDCDDIYAQGYQSDGVYLIYPSGPSVPVPVFCDMTTEGGKWTVFQKRF**N**GSVSFFRGWN

pan PLDCDDIYAQGYQSDGVYLIYPSGPSVPVPVFCDMTTEGGKWTVFQKRFNGSVSFFRGWN

mus PLDCDDIYAQGYQEDGVYLIYPYGPSVPVPVFCDMTTEGGKWTVFQKRFNGSVSFFRGWS

* ** ****.* *.**:* *:* . *:*****:** *****:* :***.*****.

gal AYRDGFGKLTGEHWLGLKRIHVLTVQGSYELRVDLEDFDNGTAFAHYGSFGVGLFSVDPE

hum DYKLGFGRADGEYWLGLQNMHLLTLKQKYELRVDLEDFE**N**NTAYAKYADFSISPNAVSAE

pan DYKLGFGRADGEYWLGLQNMHLLTLKQKYELRVDLEDFENNTAYAKYADFSISPNAVSAE

mus DYKLGFGRADGEYWLGLQNLHLLTLKQKYELRVDLEDFENNTAYAKYIDFSISPNAISAE

*: ***: **:****:.:*:**:: .**********:*.**:*:* .*.:. ::. *

gal EDGYPISISDYS-GTAGDSFLKHNGMKFTTKDLDNDHSENNCAAFYHGAWWYRNCHTSNL

hum EDGYTLFVAGFEDGGAGDSLSYHSGQKFSTFDRDQDLFVQNCAALSSGAFWFRSCHFANL

pan EDGYTLFVAGFEDGGAGDSLSYHSGQKFSTFDRDQDLFVQNCAALSSGAFWFRSCHFANL

mus EDGYTLYVAGFEDGGAGDSLSYHSGQKFSTFDRDQDLFVQNCAALSSGAFWFRSCHFANL

**** : ::.:. * ****: *.* **:* * *:* :****: **:*:*.** :**

gal NGQYLKGHHSSYADGIEWSSWTGWQYSLKFTEMKIRPVREEN

hum NGFYLGGSHLSYANGINWAQWKGFYYSLKRTEMKIRRA----

pan NGFYLGGSHLSYANGINWAQWKGFYYSLKRTEMKIRRA----

mus NGFYLGGSHLSYANGINWAQWKGFYYSLKRTEMKIRRA----

** ** * * ***:**:*:.*.*: **** ****** .

1. **Myeloblastin**

gal MGVFFTL----STSAAIVLLILPGDLCVDIIGGHEVAPHSRPFMAML-----KGKEFCGG

mus MAGSYPSPKGIHPFLLLALVVGGAVQASKIVGGHEARPHSRPYVASLQLSRFPGSHFCGG

hum MAHRPPSP--ALASVLLALLLSGAARAAEIVGGHEAQPHSRPYMASLQMRGNPGSHFCGG

pan MAHRPPSP--ALASVLLALLLSGAARAAEIVGGHEAQPHSRPYMASLQMRGNPGSHFCGG

*. :.*:: . . .*:****. *****::* * *..****

gal ALIKPSWVLTAAHCNLKG----GRVILGAHSRTKREEEEQVIEIAEEIRYPDYCPERKEH

mus TLIHPRFVLTAAHCLQDISWQLVTVVLGAHDLLSSEPEQQKFTIS-QVFQNNYNPEENLN

hum TLIHPSFVLTAAHCLRDIPQRLVNVVLGAHNVRTQEPTQQHFSVA-QVFLNNYDAENKLN

pan TLIHPSFVLTAAHCLQDIPQRLVNVVLGAHNVRTQEPTQQHFSVA-QVFLNNYDAENKLN

:**:* :******* . *:****. . * :* : :: :: :* *.: :

gal DIMLLKLKKRAKINSAVKVIPLPTSGDDLKQGTICSVAGWGQISKRGNKMSDTLREVNVT

mus DVLLLQLNRTASLGKEVAVASLPQQDQTLSQGTQCLAMGWGRLGTQAP-TPRVLQELNVT

hum DVLLIQLSSPA**N**LSASVATVQLPQQDQPVPHGTQCLAMGWGRVGAHDP-PAQVLQEL**N**VT

pan DVLLIQLSSPANLSDSVATVQLPQQDQPVPHGTQCLAMGWGRVGTHDP-PAQVLQELNVT

*::*::*. *.:. * . ** ..: : :** * . ***::. : .*:*:***

gal VISRRICNDKKHYRKEPRITDNMICAGSKRGDKDSCRGDSGGPLICNNVMKGITSFGKSP

mus VVTF-LCR------------EHNVCTLVPRRAAGICFGDSGGPLICNGILHGVDSFVIRE

hum VVTF-FCR------------PHNICTFVPRRKAGICFGDSGGPLICDGIIQGIDSFVIWG

pan VVTF-FCR------------PHNICTLVPRRRAGICFGDSGGPLICDGIIQGIDSFVIWG

*:: :*. : :*: * . * *********:.:::*: **

gal CGDPGGPGVYTRITDKYLKWIRKTIGGDLLTGF-

mus CASLQFPDFFARV-SMYVDWIQNVLRGAEP----

hum CATRLFPDFFTRV-ALYVDWIRSTLRRVEAKGRP

pan CATRLFPDFFARV-ALYVDWIRSTLRRVEAEGRL

*. *..::*: *:.**:..:

1. **High affinity immunoglobulin epsilon receptor subunit alpha**

gal -----------MAVSRALLLLAQALSLAVAEMALLTMDPPWSVIFQGESVTLRCQGPPVH

mus -----MVTGRSAQLCLALLFMSLDVILTATEKSVLTLDPPWIRIFTGEKVTLSCYGNNHL

hum ---MAPAMESPTLLCVALLFFAPDGVLAVPQKPKVSLNPPWNRIFKGE**N**VTLTCNGNNFF

pan MKNMAPAMESPTLLCVALLFFAPDGVLAVPQKPKVSLNPPWNRIFKGENVTLTCNGNNFF

:. ***::: *:. : ::::*** ** **.*** * *

gal KQ-QPTAWYHNGKLLEHTETNTYRIQNARYKQNGRYECQSPGSTSSNSVTLSVSYDLLIL

mus QMNSTTKWIHNGTVSEVNS-SHLVIVSATVQDSGKYICQKQGLFKSKPVYLNVTQDWLLL

hum EV-SSTKWFH**N**GSLSEET**N**-SSLNIVNAKFEDSGEYKCQHQQV**N**ESEPVYLEVFSDWLLL

pan EV-SSTKWFHNGSLSEETN-SSLNIVNAKFEDSGEYKCQHQQVNESEPVYLEVFSDWLLL

: . * * ***.: * .. . * .* ::.*.* ** .*: * *.* * *:*

gal QVPSHAVFEGELLQMQCRGWKVGSLAAVRFYRDGADITKPYTSAMQLSIPQAKAHHSGRY

mus QTSADMILVHGSFDIRCHGWKNWNVRKVIYYRNDHAFNYSYES--PVSIREATLNDSGTY

hum QASAEVVMEGQPLFLRCHGWRNWDVYKVIYYKDGEALKYWYEN-H**N**ISIT**N**ATVEDSGTY

pan QASAEVVMEGQPLFLRCHGWRNWDVYKVIYYKDGEALKYWYEN-HNISITNATVEDSGTY

*. :. :: : ::*:**: .: * :*::. :. * . :** :*. ..** *

gal HCSADMHSYLSLKRRESQGLYISIKELFTSPVLSVASSAEPLEGSPLNLSCITQLSPYRP

mus HCKGYLRQVE----YESDKFRIAV---------------------------VKA---YKC

hum YCTGKVWQLD----YESEPL**N**ITV---------------------------IKA---PR-

pan YCTGKVWQLD----YESEPLNITV---------------------------IKA---PR-

:*.. : . **: : *:: :. :

gal HTVLWYLFYGNS------TVLQGPVTSSEYQVPAVR-LMDTGFYSCEVRTESSNVQKWSP

mus -KYYWLQLIFPLLVAILFAVDTGLLLSTEEQFKSVLEIQKTGKYKK-VETE---------

hum -EKYWLQFFIPLLVVILFAVDTGLFISTQQQVTFLLKIKRTRKGFR-LLNP---------

pan -EKYWLQFFIPLLVAILFAVDTGLFISTQQQVTFLLKIKRTRKGFR-LLTP---------

* : :* * . *:: *. : : * : .

gal RVPITIKRVPISGMSLEVWPQEGQVMEGHRLVLHCSVATGTGSISFSWHREGSAEVLGRD

mus L----LT-----------------------------------------------------

hum H----PKPNPKNN-----------------------------------------------

pan H----PKPNPKNN-----------------------------------------------

.

gal SRYEIPSTQQSDNGQYYCMASNGDSPARSLKVQVTVVGVSSPFCSRVRALLLLAALGTLL

mus ------------------------------------------------------------

hum ------------------------------------------------------------

pan ------------------------------------------------------------

gal LEVMVVHVLTAGSRAPKGTKQQTLMEPFAPGGDV

mus ----------------------------------

hum ----------------------------------

pan ----------------------------------

1. Synaptophysin-like protein 1

gal ------------------MFGLQVDCGLLLEPLGFIKVLEWIFSIFVFSTCGGFHGETTL

mus MASKANMVRQRFSRLSQRMSAFQINLNPLKEPLGFIKILEWFASIFAFATCGGFKGKTEI

hum MAPNIYLVRQRISRLGQRMSGFQINLNPLKEPLGFIKVLEWIASIFAFATCGGFKGQTEI

pan MAPNIYLVRQRISRLGQRMSGFQINLNPLKEPLGFIKVLEWIASIFAFATCGGFKGQTEI

* .:*:: . * *******:***: ***.*:*****:*:* :

gal LVTCKGV---VNKTVTAVFAYPFRLNTVRFSA-PDSNHCGGTWTDIYLVGNFSSSAQFFV

mus QVNCPKVGVNKNQTVTATFGYPFRLNQASFHTPPNVNVCDVNWEKHVLIGDYSSSAQFYV

hum QVNCPPA-VTE**N**KTVTATFGYPFRLNEASFQPPPGVNICDVNWKDYVLIGDYSSSAQFYV

pan QVNCPPA-ITENKTVTATFGYPFRLNEASFQPPPGVNICDVNWKDYVLIGDYSSSAQFYV

*.* . *:****.*.****** . * *. * *. .* . *:*::******:*

gal TLAALAFLYCIAALVVYVGYKHVYQHNSKFPLTDLVVTIITTFLWLVSTSAWAKALADIK

mus TFAVFVFLYCIAALLLYVGYTNLYRDSRKLPMIDFIVTLVATFLWLVSSSAWAKALTDIK

hum TFAVFVFLYCIAALLLYVGYTSLYLDSRKLPMIDFVVTLVATFLWLVSTSAWAKALTDIK

pan TFAVFVFLYCIAALLLYVGYTSLYLDSRKLPMIDFVVTLVATFLWLVSTSAWAKALTDIK

*:*.:.********::****. :* .. *:*: *::**:::*******:*******:***

gal ISTGAGIIPLIDSCKA-PETTCHFGSVTSMRSLNVSVVFGLLNMVLWGGNIWFVYKDTNL

mus VATGHRIVEELEICNPESGVSCYFVSVTSMGSLNVSVIFGFLNMILWGGNAWFVYKETSL

hum IATGHNIIDELPPCKK-KAVLCYFGSVTSMGSLNVSVIFGFL**N**MILWGGNAWFVYKETSL

pan IATGHNIIDELPPCKK-KAVLCYFGSVTSMGSLNVSVIFGFLNMILWGGNAWFVYKETSL

::** *: : *: . *:* ***** ******:**:***:***** *****:*.*

gal HSRLIRGSQVP-G-TYSAQRI

mus HSPSNTSASHSQGGGPPTSGM

hum HSPSNTSAPHSQGGIPPPTGI

pan HSPSNTSAPHSQGGIPPPTGI

** .: * :

1. LA class II histocompatibility antigen, DP alpha 1 chain

gal ----------MAVLSGAAVPLLLLGVLGGVGAVLKPHVLLQAEFYQRSEGPDKAWAQFGF

mus ------MPRSRALILGVLALTTMLSLCGGEDDIEADHVGTYGISVYQSPG---DIGQYTF

hum MRPEDRMFHIRAVILRALSLAFLLSLR-GAGAIKADHVSTYAAFV-QTHR---PTGEFMF

pan MRPEDRMFHIRAVIFRALSLAFLLSLR-GAGAIKADHVSTYATFV-QTHR---PTGEFMF

*:: . :*.: * . : ** . :: .:: *

gal HFDADELFHVELDAAQTVWRLPEFGRFASFEAQGALQNMAVGKQNLEVMIGNSNRSQQDF

mus EFDGDELFYVDLDKKETVWMLPEFGQLASFDPQGGLQNIAVVKHNLGVLTKRSNSTPATN

hum EFDEDEMFYVDLDKKETVWHLEEFGQAFSFEAQGGLANIAILNNNLNTLIQRS**N**HTQATN

pan EFDEDEMFYVDLDKKETVWHLEEFGRAFSFEAQGGLANIAILNNNLNILIQRSNHTQAAN

.** **:*:*:** :*** * ***: **: **.* *:*: ::** : .** :

gal VTPELALFPAEAVSLEEPNVLICYADKFWPPVATMEWRRNGAVVSEGVYDSVYYGRPDLL

mus EAPQATVFPKSPVLLGQPNTLICFVDNIFPPVINITWLRNSKSVADGVYETSFFVNRDYS

hum DPPEVTVFPKEPVELGQPNTLICHIDKFFPPVL**N**VTWLCNGELVTEGVAESLFLPRTDYS

pan DPPEVTVFPKEPVELGQPNTLICHIDKFFPPVLNVTWLCNGEPVTEGVAESLFLPRTDYS

*: ::** . * * :**.***. *:::*** .: * *. *::** :: : . *

gal FRKFSYLPFVPQRGDVYSCAVRHWGAEGPVQRMWGPEVPEPPSESSATLWCAVGLAVGIA

mus FHKLSYLTFIPSDDDIYDCKVEHWGLEEPVLKHWEPEIPAPMSELTETVVCALGLSVGLV

hum FHKFHYLTFVPSAEDFYDCRVEHWGLDQPLLKHWEAQEPIQMPETTETVLCALGLVLGLV

pan FHKFHYLTFVPSAEDFYDCKVEHWGLDQPLLKHWEAQEPIQMPETRETVLCALGLVLGLV

*:*: ** *:*. *.*.* *.*** : *: : * : * * *: **:** :*:.

gal GIAAGTALILRAVRRNAANRQPGLL

mus GIVVGTIFIIQGLRSGGTSRHPGPL

hum GIIVGTVLIIKSLRSGHDPRAQGTL

pan GIIVGTILIIKSLRSGRDPRAQGPL

** .** :*::.:* . * * *

1. CD 40 ligand

gal MNEAYSPAAPRPMGSTSPSTMKMFMCFLSVFMVVQTIGTVLFCLYLHMKMDKMEEVLSLN

mus MIETYSQPSPRSVATGLPASMKIFMYLLTVFLITQMIGSVLFAVYLHRRLDKVEEEVNLH

hum MIETYNQTSPRSAATGLPISMKIFMYLLTVFLITQMIGSALFAVYLHRRLDKIEDERNLH

pan MIETYNQPSPRSAATGLPISMKIFMYLLTVFLITQMIGSALFAVYLHRRLDKIEDERNLH

* *:*. :** .: * :**:** :*:**::.* **:.**.:*** ::**:*: .*:

gal EDYIFLRKVQKCQTGEDQKSTLLDCEKVLKGFQDLQCKDR---TASEELPKFEMHRGHEH

mus EDFVFIKKLKRCNKGEGSL-SLLNCEEMRRQFEDLVKDITLNKEE-KKENSFEMQRGDED

hum EDFVFMKTIQRCNTGERSL-SLLNCEEIKSQFEGFVKDIMLNKEETKKENSFEMQKGDQN

pan EDFVFMKTIQRCNTGERSL-SLLNCEEIKSQFEGFVKDIMLNKEETKKENSFEMQKGDQN

**::*::.:::*:.** . :**:**:: *:.: . :: .***::*.:.

gal PHLKSRNETSVAEEKRQPIATHLAGVKSNTTVRVLKWMTTSYAPTSS-LISYHE-GKLKV

mus PQIA----------------AHVVSEANSNAASVLQWAKKGYYTMKSNLVMLENGKQLTV

hum PQIA----------------AHVISEASSKTTSVLQWAEKGYYTMSNNLVTLENGKQLTV

pan PQIA----------------AHVISEASSKTTSVLQWAEKGYYTMSNNLVTLENGKQLTV

*:: :*: . ...:. **:* ..* .. *: .: :*.*

gal EKAGLYYIYSQVSFCTKAAA--SAPFTLYIYLYLPMEEDRLLMKGLDTHSTSTALCELQS

mus KREGLYYVYTQVTFCSNREPSSQRPFIVGLWLKPSSGSERILLKAANTHSS-SQLCEQQS

hum KRQGLYYIYAQVTFCSNREASSQAPFIASLCLKSPGRFERILLRAANTHSS-AKPCGQQS

pan KRQGLYYIYAQVTFCSNREASSQAPFIASLCLKSPGRFERILLRAANTHSS-AKPCGQQS

:: ****:*:**:**:: . ** : * :*:*::. :***: : * **

gal IREGGVFELRQGDMVFVNVTDSTAVNVNPGNTYFGMFKL

mus VHLGGVFELQAGASVFVNVTEASQVIHRVGFSSFGLLKL

hum IHLGGVFELQPGASVFV**N**VTDPSQVSHGTGFTSFGLLKL

pan IHLGGVFELQPGASVFVNVTDPSQVSHGTGFTSFGLLKL

:: ******: * ******: : * * : **::**

1. Granzyme A

gal ---MGVFFTLSTSAAIVLLILPGDLCVDIIGGHEVAPHSRPFMAMLK--GKEFCGGALIK

hum MRNSYRFLASSLSVVVSLLLIPEDVCEKIIGGNEVTPHSRPYMVLLSLDRKTICAGALIA

pan MRNSYRFLASSLSVVVSLLLIPEDVCEKIIGGNEVTPHSRPYMVLLSLDRKNICAGALIA

mus MRNASGPRGPSLATLLFLLLIPEGGCERIIGGDTVVPHSRPYMALLKLSSNTICAGALIE

* :. : **::* . * ****. *.*****:*.:*. : :*.****

gal PSWVLTAAHCNLK-GGRVILGAHSRTKREEEEQVIEIAEEIRYPDYCPERKEHDIMLLKL

hum KDWVLTAAHCNLNKRSQVILGAHSITREEPTKQIMLVKKEFPYPCYDPATREGDLKLLQL

pan KDWVLTAAHCNLNKRSQVILGAHSITREEPTKQIMLVKKEFPYPCYDPDTREGDLKLLQL

mus KNWVLTAAHCNVGKRSKFILGAHSINK-EPEQQILTVKKAFPYPCYDEYTREGDLQLVRL

.*********: .:.****** .: * :*:: : : : ** * :* *: *::*

gal KKRAKINSAVKVIPLPTSGDDLKQGTICSVAGWGQISKRGNKMSDTLREVNVTVISRRIC

hum MEKAKINKYVTILHLPKKGDDVKPGTMCQVAGWGRTHNS-ASWSDTLREV**N**ITIIDRKVC

pan TEKAKINKYVTILHLPKKGDDVKPGTMCQVAGWGRTHNS-ASRSDTLREVNITIIDRKVC

mus KKKATVNRNVAILHLPKKGDDVKPGTRCRVAGWGRFGNK-SAPSETLREVNITVIDRKIC

::*.:* * :: **..***:* ** * *****: : *:******:*:*.*::*

gal NDKKHYRKEPRITDNMICAGSKRGDKDSCRGDSGGPLICNNVMKGITSFGK-SPCGDPGG

hum NDRNHYNFNPVIGMNMVCAGSLRGGRDSCNGDSGSPLLCEGVFRGVTSFGLENKCGDPRG

pan NDQNHYNFNPVIGMNMVCAGSLRGGRDSCNGDSGSPLLCEGVFRGVTSFGLENKCGDPRG

mus NDEKHYNFHPVIGLNMICAGDLRGGKDSCNGDSGSPLLCDGILRGITSFGG-EKCGDRRW

**.:**. .* * **:***. **.:***.****.**:*:.:::*:**** . ***

gal PGVYTRITDKYLKWIRKTIGGDLLTGF

hum PGVYILLSKKHLNWIIMTIKGAV----

pan PGVYTLLSKKHLNWIIMTIKGAV----

mus PGVYTFLSDKHLNWIKKIMKGSV----

**** ::.*:*:** : * :

1. Follistatin related protein 3

gal ---------MPLRLAL----CLLVLCSLAGGDAAHGGICWLQQGKEAKCTMILKTGVTWE

mus ---MRSGALWPLLWGALVWTVGSVGAVMGSEDSVPGGVCWLQQGREATCSLVLKTRVSRE

hum MRPGAPGPLWPLPWGALAWAVGFVS-SMGSGNPAPGGVCWLQQGQEATCSLVLQTDVTRA

pan MRPGAPGPLWPLPWGALAWAVGFVS-SMGSGNPAPGGVCWLQQGQEATCSLVLQTDVTRA

** . * :.. : . **:******:**.*:::*:* *:

gal ECCANGNVDVAWSNYTYPGNKISLLGFLGLVTCHPCKESCEGVVCGPDKVCKMKHGRPQC

mus ECCASGNINTAWSNFTHPGNKISLLGFLGLVHCLPCKDSCDGVECGPGKACRMLGGRPHC

hum ECCASGNIDTAWS**N**LTHPGNKINLLGFLGLVHCLPCKDSCDGVECGPGKACRMLGGRPRC

pan ECCASGNIDTAWSNLTHPGNKINLLGFLGLVHCLPCKDSCDGVECGPGKACRMLGGRPRC

****.**::.**** *:*****.******** * ***:**:** ***.*.*:* ***:*

gal ACAPDCSSLPRKLQVCGSDGYTYRDECDLLTAKCRDHPDLEVMYQGKCKKSCSSVVCPGT

mus ECVPNCEGLPAGFQVCGSDGATYRDECELRTARCRGHPDLRVMYRGRCQKSCAQVVCPRP

hum ECAPDCSGLPARLQVCGSDGATYRDECELRAARCRGHPDLSVMYRGRCRKSCEHVVCPRP

pan ECAPDCSGLPARLQVCGSDGATYRDECELRAARCRGHPDLSVMYRGRCRKSCEHVVCPRP

*.*:*..** :******* ******:* :*:**.**** ***:*:*:*** ****

gal HTCVVDQTGSAHCVMCRTAPCPEPTSLDHSLCGNNNVTYPSACHLRRATCHLGRSIGVRH

mus QSCLVDQTGSAHCVVCRAAPCPVPSNPGQELCGNNNVTYISSCHLRQATCFLGRSIGVRH

hum QSCVVDQTGSAHCVVCRAAPCPVPSSPGQELCGNN**N**VTYISSCHMRQATCFLGRSIGVRH

pan QSCVVDQTGSAHCVVCRAAPCPVPSSPGQELCGNNNVTYISSCHMRQATCFLGRSIGVRH

::*:**********:**:**** *:. .:.********* *:**:*:***.*********

gal YGSCSASARFSMETD--NAEENYV

mus PGICTGGPKVPA-----EEEENFV

hum AGSCAGTPEEPPGGESAEEEENFV

pan AGSCAGTPKEPPDGESAEEEENFV

* *:. . : ***:*

1. Phosphoinositide-3-kinase interacting protein 1

gal MLRGGPLRALGAVLLGSLLLAAARGAEECLRGDGASYRGNRRVASGGAPCLNWLAVRSET

hum ----MLLAWVQAFLVSNMLLAEAYGSGGCFWDNGHLYREDQTSPAPGLRCLNWLDAQSGL

pan ----MLLAWVQAFLVSNMLLAEAYGSGGCFWDNGHLYREDQTSPAPGLRCLNWLDAQSGL

mus ----MLLAWVHTFLLSNMLLAEAYGSGGCFWDNGHLYREDQPSPAPGLRCLNWLAAQGSR

* : :.*:..:*** * *: *: .:* ** :: : * ***** .:.

gal GAALPAVAEDHDSCRNPNGD-AAPWCYVRGAAGIPERRTCEIPPCPDATATTAPVPTA--

hum ASAPVSGAG**N**HSYCRNPDEDPRGPWCYVSGEAGVPEKRPCEDLRCPETTSQALPA-FTTE

pan ASTPVSGAGNHSYCRNPDEDPRGPWCYVSGEAGVPEKRPCEDLRCPEATSQALPA-FTTE

mus ESLTEPSPGNHNYCRNPDQDPRGPWCYISSETGVPEKRPCEDVSCPETTSQAPPPSSAME

: :*. ****: * .****: . :*:**:* ** **::*: : * :

gal ----EVNVSQEVNQVFEPADTLPSRSEAAAVQPVIGISQRVQMNSREKKDLGTLGYVLGL

hum IQEASEGPGADEVQVFAPANALPARSEAAAVQPVIGISQRVRMNSKEKKDLGTLGYVLGI

pan IQEASEGPGADEVQVFAPANALPARSEAAAVQPVIGISQRVRMNSKEKKDLGTLGYVLGI

mus LEEKSGAPGDKEAQVFPPANALPARSEAAEVQPVIGISQLVRMNSKEKKDLGTLGYVLGI

. . . *** **::**:***** ********* *:***:*************:

gal IMMVIIIAIGAGIVVGYIYKRGKDLKEKHEQKVYEREMQRITLPLSAFTNPACELVDENT

hum TMMVIIIAIGAGIILGYSYKRGKDLKEQHDQKVCEREMQRITLPLSAFTNPTCEIVDEKT

pan TMMVIIIAIGAGIILGYSYKRGKDLKEQHDQKVCEREMQRITLPLSAFTNPTCEIVDEKT

mus TMMVIILAIGAGIIVGYTYKRGKDLKEQHEKKACEREMQRITLPLSAFTNPTCETVDENT

*****:******::** *********:*::*. *****************:** ***:*

gal IVVHTNQTPVEDTHDGSGPLMGQAGTPGA

hum VVVHTSQTPVD-PQEGTTPLMGQAGTPGA

pan VVVHTSQTPVD-PQEGSTPLMGQAGTPGA

mus IIVHSNQTPAD-VQEGSTLLTGQAGTPGA

::**:.***.: ::*: * ********

1. C-typle lectin domain 12A

gal ----------MEEVREYP------------------------------------------

mus ----------MSEEIVYANLKIQDPDKKEETQKSDKCGGKVSADASHSQQKTVLILILLC

hum ----------MSEEVTYADLQFQNSSEMEKIPEIGKFGEKAPPAPSHVWRPAALFLTLLC

pan MWTDFFTYSSMSEEVTYADLQFQNSSEKEKIPEIGKFGEKAPPAPSRVWRPAALFLTLLC

*.* *

gal ---------------HVLGTEWSR-----REGPSRGACVTFQLT--------MAAVFTVL

mus LLLFIGMGVLGGIFYTTLATEMIKSNQLQRAKEELQENVSLQLKHNLNSSKKIKNLSAML

hum LLLLIGLGVLASMFHVTLKIEMKKMNKLQNISEELQR**N**ISLQLMSNM**N**ISNKIRNLSTTL

pan LLLLIGLGVLASMFHVTLKIEMKKMNKLQNISEELQRNVSLQLMSNMNISNKIRNLSTTL

.* * : . . :::** : : : *

gal LITAVAFAVQAF-QPHPQPCAQCPFDWIGFRGKCYYFSEDESNWTSSQNNCSALGASLAV

mus QSTATQLCRELYSKEPEHKCKPCPKGSEWYKDSCYSQLNQYGTWQESVMACSARNASLLK

hum QTIATKLCRELYSKEQEHKCKPCPRRWIWHKDSCYFLSDDVQTWQESKMACAAQ**N**ASLLK

pan QTIATKLCRELYSKEQEHECKPCPRRWIWHKDSCYFLSDDVHTWQESKMACAAQNASLLK

*. :. : : : : * ** .:..** :: .* .* *:* .***

gal FDSAEDLSFTMRHKGSSPHWVGLSREGKEHAWEWVNRSPL-S----HLFQVQGDGLCAYL

mus VKNKDVLEFIKYKKLRY-FWLALLPRKDRTQYPLSEKMF-LSEESERSTDDIDKKYCGYI

hum INNKNALEFIKSQSRSYDYWLGLSPEEDSTRGMRVDNIINSSAWVIRNAPDLNNMYCGYI

pan INNKNALEFIKSQSRSYDYWLGLSPEEDSTHGMRVDNIINSSAWVIRNAPDLNNMYCGYI

... : *.* :. .*:.* . . :. * : .. *.*:

gal GDAGLSSSHCSARRNWVCTKPALQKPRKNFCIST-----

mus DRVNVYYTYCTDENNIICEETASKVQLESVLNGLPEDSR

hum NRLYVQYYHCTYKKRMICEKMANPVQLGSTYFREA----

pan SRLYVQYYHCTYKQRMICEKMANPVQLGSIYFREA----

. : :*: ... :* : * .

1. HLA class II histocompatibility antigen,DRB1-15 beta chain

gal ---MGSGRVPAAGAVLVALLALGARPAAGTRPSAFFQWTFKAECHYLNGTERVRYLVRYV

mus --MVWLPRVPCVAAVILLLTVLSPPVALVRDSRPWFLEYCKSECHFYNGTQRVRFLKRYF

hum MVCLKLPGGSCMTALTVTLMVLSSPLALSGDTRPRFLWQPKRECHFF**N**GTERVRFLDRYF

pan MLCLKLPGVSCMTALTVTLMVLSSPLALAGDTRSRFLLQPKGECHFFNGTERVRFLDRYF

: . *: : * .*. * * * ***: ***:***:* **.

gal YNRQEYAHFDSDVGKHVADTALGEPQAEYWNSNAEILENRMNEVDTYCRHNYGVVESFTV

mus YNLEENLRFDSDVGEFRAVTELGRPDAENWNSQPEILDEKRAAVDTYCRHNYEIFDNFLV

hum YNQEESVRFDSDVGEFRAVTELGRPDAEYWNSQKDILEQARAAVDTYCRHNYGVVESFTV

pan YNQEEFMRFDSDVGEYRAVTELGRPVAEYCNSQKDFLEQARAAVDNYCRHNYGAVESFTV

** :* :******:. * * **.* ** **: ::*:: **.****** .:.* *

gal QRSVEPKVRVSALQSGSLPETDRLACYVTGFYPPEIEVKWFLNGREETERVVSTDVMQNG

mus PRRVEPTVTVYPTKTQPLEHHNLLVCSVSDFYPGNIEVRWFRNGKEEKTGIVSTGLVRNG

hum QRRVQPKVTVYPSKTQPLQHHNLLVCSVSGFYPGSIEVRWFLNGQEEKAGMVSTGLIQNG

pan QRRVQPKVTVYPAKTQPLQHHNLLVCSVSGFYPGSIEVRWFLNGQEEKAGVVSTGLIQNG

* *:*.* * :: * . : *.* *:.*** .***:** **:**. :***.:::**

gal DWTYQVLVVLETVPRRGDSYVCRVEHASLRQPISQAWEPPADAGRSKLLTGVGGFVLGLV

mus DWTFQTLVMLETVPQSGEVYTCQVEHPSLTDPVTVEWKAQSTSAQNKMLSGVGGFVLGLL

hum DWTFQTLVMLETVPRSGEVYTCQVEHPSVTSPLTVEWRARSESAQSKMLSGVGGFVLGLL

pan DWTFQTLVMLETVPRSGEVYTCQVEHPSVTSPLTVEWRARSESAQSKMLSGVGGFVLGLL

***:*.**:*****: *: *.*:*** *: .*:: *. : :.:.*:*:*********:

gal FLALGLFVFLRGQKGRPVAAAPGMLN

mus FLRAGLFIYFRNQKGQSGLQPTGLLS

hum FLGAGLFIYFRNQKGHSGLQPTGFLS

pan FLGAGLFIYFRNQKGHSGLQPTGFLS

** ***:::*.***: *:*.

1. HLA class II histocompatibility antigen,DR beta 3 chain

gal ---MGSGRVPAAGAVLVALLALGARPAAGTRPSAFFFHGAISECHYLNGTERVRYLDRYF

hum MVCLKLPGGSSLAALTVTLMVLSSRLAFAGDTRPRFLELRKSECHFF**N**GTERVRYLDRYF

pan MVCLKLPGGSSLAALTVTLMVLSSRLAFAGDTRPRFLELHKSECHFFNGTERVRYLDRYF

mus --MVWLPRVPCVAAVILLLTVLSPPVALVRDSRPWFLEYCKSECHFYNGTQRVRFLKRYF

: . .*: : * .*. * *:. ****: ***:***:*.***

gal YNRQEFTHFDSDVGKFVADTPLGEPQAEYWNSNAEILEYKRGQVDRFCRHNYGILESFTV

hum HNQEEFLRFDSDVGEYRAVTELGRPVAESWNSQKDLLEQKRGRVDNYCRHNYGVGESFTV

pan HNQEEFLRFDSDVGEYRAVTELGRPVAESWNSQKDYVEQKRGQVDNYCRHNYRVGESFTV

mus YNLEENLRFDSDVGEFRAVTELGRPDAENWNSQPEILDEKRAAVDTYCRHNYEIFDNFLV

:* :* :******:: * * **.* ** ***: : :: **. ** :***** : :.* *

gal QRSVEPKVRVSALQSGSLPETDRLACYVTGFYPPEIEVKWFQNGREETERVVSTDVMQNG

hum QRRVHPQVTVYPAKTQPLQHHNLLVCSVSGFYPGSIEVRWFRNGQEEKAGVVSTGLIQNG

pan QRRVLPKVTVYPAKTQPLQHHNLLVCSVSGFYPGSIEVRWFQNGQEEKTGVVSTGLIQNG

mus PRRVEPTVTVYPTKTQPLEHHNLLVCSVSDFYPGNIEVRWFRNGKEEKTGIVSTGLVRNG

* * * * * :: * . : *.* *:.*** .***:**:**:**. :***.:::**

gal DWTYQVLVVLETVPRRGDSYVCRVEHASLRQPISQAWEPPADAGRSKLLTGVGGFVLGLV

hum DWTFQTLVMLETVPRSGEVYTCQVEHPSVTSALTVEWRARSESAQSKMLSGVGGFVLGLL

pan DWTFQTLVMLETVPRSGEVYTCQVEHPSVTSPLTVEWRARSESAQSKMLSGVGGFVLGLL

mus DWTFQTLVMLETVPQSGEVYTCQVEHPSLTDPVTVEWKAQSTSAQNKMLSGVGGFVLGLL

***:*.**:*****: *: *.*:*** *: . :: *. : :.:.*:*:*********:

gal FLALGLFVFLRGQRGRPVAAAPGMLN

hum FLGAGLFIYFRNQKGHSGLQPTGFLS

pan FLGAGLFIYFRNQKGHSGLQPTGFLS

mus FLRAGLFIYFRNQKGQSGLQPTGLLS

** ***:::*.*:*: *:*.

1. HLA class II histocompatibility antigen,DR beta 5 chain

gal ---MGSGRVPAAGAVLVALLALGARPAAGTRPSAFFFCGAIFECHYLNGTERVRFLDREI

mus --MVWLPRVPCVAAVILLLTVLSPPVALVRDSRPWFLEYCKSECHFYNGTQRVRFLKRYF

hum MVCLKLPGGSYMAKLTVTLMVLSSPLALAGDTRPRFLQQDKYECHFF**N**GTERVRFLHRDI

pan MVCLKLPGGSYMAVLTVTLMVLSSPLALAGDTRPRFLKQDKHECHFFNGTERVRFLHRDI

: . : : * .*. * *: ***: ***:*****.* :

gal YNRQQYAHFDSDVGKYVADTPLGEPQAEYWNSNAEFMENRMNEVDTFCRHNYGVGESFTV

mus YNLEENLRFDSDVGEFRAVTELGRPDAENWNSQPEILDEKRAAVDTYCRHNYEIFDNFLV

hum YNQEEDLRFDSDVGEYRAVTELGRPDAEYWNSQKDFLEDRRAAVDTYCRHNYGVGESFTV

pan YNQEEDLRFDSDVGEYRAVTELGRPDAEYWNSQKDILERRRAAVDTYCRHNYGVLESFTV

** :: :******:: * * **.*:** ***: :::: : ***:***** : :.* *

gal QRSVEPKVRVSALQSGSLPETDRLACYVTGFYPPEIEVKWFLNGREETERVVSTDVMQNG

mus PRRVEPTVTVYPTKTQPLEHHNLLVCSVSDFYPGNIEVRWFRNGKEEKTGIVSTGLVRNG

hum QRRVEPKVTVYPARTQTLQHHNLLVCSVNGFYPGSIEVRWFRNSQEEKAGVVSTGLIQNG

pan QRRVEPKVTVYPARTQPLQHHNLLVCSVNGFYPGSIEVRWFRNGQEEKAGVVSTGLIQNG

* ***.* * :: * . : *.* *..*** .***:** *.:**. :***.:::**

gal DWTYQVLVVLETVPRRGDSYVCRVEHASLRQPISQAWEPPADAGRSKLLTGVGGFVLGLV

mus DWTFQTLVMLETVPQSGEVYTCQVEHPSLTDPVTVEWKAQSTSAQNKMLSGVGGFVLGLL

hum DWTFQTLVMLETVPRSGEVYTCQVEHPSVTSPLTVEWRAQSESAQSKMLSGVGGFVLGLL

pan DWTFQILVMLETVPRSGEVYTCQVEHPSVTSPLTVEWRARSESAQSKMLSGVGGFVLGLL

***:* **:*****: *: *.*:*** *: .*:: *. : :.:.*:*:*********:

gal FLALGLFVFLRGQKGRPVAAAPGMLN

mus FLRAGLFIYFRNQKGQSGLQPTGLLS

hum FLGAGLFIYFKNQKGHSGLHPTGLVS

pan FLGAGLFIYFRNQKGHSGLHPTGLVS

** ***::::.***: *::.

1. Integral membrane protein 2B

hum MVKVTFNSALAQKEAKKDEPKSGEEALIIPPDAVAVDCKDPDDVVPVGQRRAWCWCMCFG

pan MVKVTFNSALAQKEAKKDEPKSGEEALIIPPDAVAVDCKDPDDVVPVGQRRAWCWCMCFG

mus MVKVTFNSALAQKEAKKDEPKSSEEALIVPPDAVAVDCKDPGDVVPVGQRRAWCWCMCFG

gal MVKVSFNSALAQKEAAKKEEEN-SQVLILPPDAKE----PEDVVVPAGQRRAWCWCMCFG

****:********** *.* :. .:.**:**** . ***.*************

hum LAFMLAGVILGGAYLYKYFALQPDDVYYCGIKYIKDDVILNEPSADAPAALYQTIEENIK

pan LAFMLAGVILGGAYLYKYFALQPDDVYYCGIKYIKDDVILNEPSADAPAALYQTIEENIK

mus LAFMLAGVILGGAYLYKYFALQPDDVYYCGLKYIKDDVILNEPSADAPAARYQTIEENIK

gal LAFMLAGVILGGAYLYKYFAFQQGGVYFCGIKYIEDGLSLPESGAEAQSARYHTIEQNIQ

********************:* ..**:**:***:*.: * * .*:* :* *:***:**:

hum IFEEEEVEFISVPVPEFADSDPANIVHDFNKKLTAYLDLNLDKCYVIPL**N**TSIVMPPRNL

pan IFEEEEVEFISVPVPEFADSDPANIVHDFNKKLTAYLDLNLDKCYVIPLNTSIVMPPRNL

mus IFEEDAVEFISVPVPEFADSDPANIVHDFNKKLTAYLDLNLDKCYVIPLNTSIVMPPKNL

gal ILEEEDVEFISVPVPEFADSDPADIVHDFHRRLTAYLDLSLDKCYVIPLNTSVVMPPKNF

*:**: *****************:*****:::*******.************:****:*:

hum LELLINIKAGTYLPQSYLIHEHMVITDRIENIDHLGFFIYRLCHDKETYKLQRRETIKGI

pan LELLINIKAGTYLPQSYLIHEHMVITDRIENIDHLGFFIYRLCHDKETYKLQRRETIKGI

mus LELLINIKAGTYLPQSYLIHEHMVITDRIENVDNLGFFIYRLCHDKETYKLQRRETIRGI

gal LELLINIKAGTYLPQSYLIHEQMIVTDRIENVDQLGFFIYRLCRGKETYKLQRKEAMKGI

*********************:*::******:*:*********:.********:*:::**

hum QKREASNCFAIRHFENKFAVETLICS-

pan QKREASNCFAIRHFENKFAVETLICS-

mus QKREASNCFTIRHFENKFAVETLICS-

gal QKREAVNCRKIRHFENRFAMETLICEQ

***** ** ******:**:*****.

1. Neutrophil elastase

gal -----------MLAALLILSLGGSALGPAGAHSSWVIGGKAAVPHSRPFIASIQMDGQHF

hum MTLGRRLACLFLACVLPALLLGGTAL------ASEIVGGRRARPHAWPFMVSLQLRGGHF

pan MTLGRRLACLFLACVLPALLLGGTAL------ASEIVGGRRARPHAWPFMVSLQLRGGHF

mus MALGR-LSSRTLAAMLLALFLGGPAL------ASEIVGGRPARPHAWPFMASLQRRGGHF

: . * * *** ** :* ::**: * **: **:.*:* * **

gal CGGFLVWPRWVMTAAHCPVPRREPSVRVVLGAHSLEQPEESQQVFGVEESIAHPLYNPRT

hum CGATLIAPNFVMSAAHCVANVNVRAVRVVLGAH**N**LSRREPTRQVFAVQRI-FENGYDPVN

pan CGATLIAPNFVMSAAHCVANVNVRAVRVVLGAHNLSRREPTRQVFAVQRI-FENGYDPVN

mus CGATLIARNFVMSAAHCVNGLNFRSVQVVLGAHDLRRQERTRQTFSVQRI-FENGFDPSQ

**. *: .:**:**** . :*:******.* : * ::*.*.*:. . ::*

gal VDNDIRMLRLNHTATLNAFVKRIRLPRPRIDLKPGTLCSVVGWGDISNYGERPIQLMEAN

hum LLNDIVILQL**N**GSATINANVQVAQLPAQGRRLGNGVQCLAMGWGLLGRNRGIASVLQEL**N**

pan LLNDIVILQLNGSATINANVQVAQLPAQGRHLGNGVQCLAMGWGLLGRNRGIASVLQELN

mus LLNDIVIIQLNGSATINANVQVAQLPAQGQGVGDRTPCLAMGWGRLGTNRPSPSVLQELN

: *** :::** :**:** *: :** : . * .:*** :. * * *

gal TTIVKRSLCRTLWKGRVSGNMLCGASRNATLQGVCAGDSGGPLVFKGKVYGVVSFSGERC

hum VTVVT-SLCRR-S-------NVCTL-VRGRQAGVCFGDSGSPLVCNGLIHGIASFVRGGC

pan VTVVT-SLCRR-S-------NVCTL-VRGRRAGVCFGDSGSPLVCNGLIHGIASFVRGGC

mus VTVVT-NMCRRRV-------NVCTL-VPRRQAGICFGDSGGPLVCNNLVQGIDSFIRGGC

.*:*. .:** :* *:* ****.*** :. : *: ** *

gal GDRRYPDIYTRISNYIDWVHHVVLSHRQPPGQRKDKPGPEKAGGDQGAAGWGRPGFPRPP

hum ASGLYPDAFAPVAQFVNWIDSIIQRSEDNPCPHPRDPDPASRTH----------------

pan ASGLYPDAFAPVAQFVNWINSIIQRSEDNPCPHPRDPDPASRTH----------------

mus GSGLYPDAFAPVAEFADWINSIIRSHNDHLLTHPKDREGRTN------------------

.. *** :: :::: :*:. :: .: : . .

gal PAPRGLMFNGN

hum -----------

pan -----------

mus -----------

1. Aquaporin-1

gal MASEFKKKMFWRAVVAEFLAMILFIFISIGSALGFNFPVSVNGTSATQDNVKVSLAFGLS

mus MASEIKKKLFWRAVVAEFLAMTLFVFISIGSALGFNYPLERNQT-LVQDNVKVSLAFGLS

hum MASEFKKKLFWRAVVAEFLATTLFVFISIGSALGFKYPVGN**N**QT-AVQDNVKVSLAFGLS

pan MASEFKKKLFWRAVVAEFLATTLFVFISIGSALGFKYPVGNNQT-AVQDNVKVSLAFGLS

****:***:*********** **:**********::*: * * .*************

gal IATMAQSVGHISGAHLNPAVTLGLLLSCQISIFKALMYILAQCLGAVVATAILSGVTSSL

mus IATLAQSVGHISGAHLNPAVTLGLLLSCQISILRAVMYIIAQCVGAIVATAILSGITSSL

hum IATLAQSVGHISGAHLNPAVTLGLLLSCQISIFRALMYIIAQCVGAIVATAILSGITSSL

pan IATLAQSVGHISGAHLNPAVTLGLLLSCQISIFRALMYIIAQCVGAIVATAILSGITSSL

***:****************************::*:***:***:**:********:****

gal PYNSLGLNALAKGINAGQGLGIEIIATLQLVLCVLATTDRRRNDVSGSAPLAIGLSVALG

mus VDNSLGRNDLAHGVNSGQGLGIEIIGTLQLVLCVLATTDRRRRDLGGSAPLAIGLSVALG

hum TGNSLGRNDLADGVNSGQGLGIEIIGTLQLVLCVLATTDRRRRDLGGSAPLAIGLSVALG

pan NGNSLGRNDLADGVNSGQGLGIEIIGTLQLVLCVLATTDRRRRDLGGSAPLAIGLSVALG

**** * **.*:*:*********.****************.*:.**************

gal HLLAIDYTGCGINPARSFGSALIANNFENHWIFWVGPIIGGAGAALIYDFILAPRSSDLT

mus HLLAIDYTGCGINPARSFGSAVLTRNFSNHWIFWVGPFIGGALAVLIYDFILAPRSSDFT

hum HLLAIDYTGCGINPARSFGSAVITH**N**FSNHWIFWVGPFIGGALAVLIYDFILAPRSSDLT

pan HLLAIDYTGCGINPARSFGSAVITHNFSNHWIFWVGPFIGGALAVLIYDFILAPRNSDLT

*********************:::.**.*********:**** *.**********.**:*

gal DRVKVWTSGQVEEYDLEGDDMNSRVEMKPK

mus DRMKVWTSGQVEEYDLDADDINSRVEMKPK

hum DRVKVWTSGQVEEYDLDADDINSRVEMKPK

pan DRVKVWTSGQVEEYDLDADDINSRVEMKPK

**:*************:.**:*********

1. Myelin zero-like protein 1

CLUSTAL O(1.2.4) multiple sequence alignment

gal MGARGGGSRKMAAAGGTAGRSAAVVAARLPLSACLLAAAASLAAVKVSAVEVTTPEEMFV

hum ----------MAASAGAGAVIAAP--DSRRWLWSVLAAALGLLTAGVSALEVYTPKEIFV

pan ----------MAAPAGAGAVIAAP--DSRRWLWSVLAAALGLLTAGVSALEVYTPKEIFV

mus ----------MAEAVGAVALIAAP--ARRRWLWSVLAAMLGLLTARISALEVHTPKEIFV

** *: . ** .:*** .* :. :**:** **:*:**

gal ENETDVKLPCTFTSAEVISSAASVSWSFQPEGAATRISFFYYSNGKPYTGKDIPFKDRVT

hum A**N**GTQGKLTCKFKSTSTTGGLTSVSWSFQPEGADTTVSFFHYSQGQVYLGNYPPFKDRIS

pan ANGTQGKLTCKFKSTSTTGGLTSVSWSFQPEGADTTVSFFHYSQGQVYLGNYPPFKDRIS

mus VNGTQGKLTCTFDSPNTTGWLTTVSWSFQPDGTDSAVSFFHYSQGQVYIGDYPPFKDRVT

* *: ** *.* * .. . ::*******:*: : :***:**:*: * *. *****::

gal WAGDLNKKDASISISNMQFQDNGTYICDVKNPPDIVVKPGEIRLRVVEKDSLPAFPIAMV

hum WAGDLDKKDASINIENMQFIH**N**GTYICDVKNPPDIVVQPGHIRLYVVEKENLPVFPVWVV

pan WAGDLDKKDASINIENMQFIHNGTYICDVKNPPDIVVQPGHIRLYVVEKENLPVFPVWVV

mus WAGDLDKKDASINIENIQAVHNGTYICDVKNPPDIVVRPGHIRLHVVEIDNLLVFLVWVV

*****:******.*.*:* .****************:**.*** *** :.* .* : :*

gal AGIVIGTVTGLTLLISIVVCLVMRKNNSKKRYSGCGTSESLMSPVKQAPQKSPSDTEGLV

hum VGIVTAVVLGLTLLISMILAVLYRRKNSKRDYTGCSTSES-LSPVKQAPRKSPSDTEGLV

pan VGIVTAVVLGLTLLISMILAVLYRRKNSKRDYTGCSTSES-LSPVKQAPRKSPSDTEGLV

mus VGTVTAVVLGLTLLISLVLVVLYRRKHSKRDYTGCSTSER-LSPVKQAPRKCPSDTEGLV

.* * ..* *******::: :: *:::**: *:**.*** :*******:*.********

gal NSVPA-RSHQGPVIYAQLDHSGGQHSDKINKSESVVYADIRKN

hum KSLPS-GSHQGPVIYAQLDHSGGHHSDKINKSESVVYADIRKN

pan KSLPS-GSHQGPVIYAQLDHSGGHHSDKINKSESVVYADIRKN

mus KSPPSAGSHQGPVIYAQLDHSGGHHSGKINKSESVVYADIRKD

:* *: ****************:**.***************:

1. Chymotrypsin-like elastase family member 3 B

gal -MLALLVPLL--LLAGGCHAAVLPHGSRVVNGQDAVPYSWPWQISLQYERDGTFSHTCGG

mus -MLRLLSSLLLVALASGCGQPSHNPSSRVVNGEEAVPHSWPWQVSLQYEKDGSFHHTCGG

hum MMLRLLSSLLLVAVASGYGPPSSRPSSRVVNGEDAVPYSWPWQVSLQYEKSGSFYHTCGG

pan MMLRLLSSLLLVAVASGYGPPSSRPSSRVVNGEDAVPYSWPWQVSLQYEKSGSFYHTCGG

** ** ** :*.* .******::***:*****:*****:.*:* *****

gal TLIAPDWVMTAAHCISSTLTYEVVLGEYDMSSAEGPEQRIPVAADDIFVHPKWNRFCAAC

mus SLITPDWVLTAGHCISTSRTYQVVLGEHERGVEEGQEQVIPINAGDLFVHPKWNSMCVSC

hum SLIAPDWVVTAGHCISSSRTYQVVLGEYDRAVKEGPEQVIPINSGDLFVHPLW**N**RSCVAC

pan SLIAPDWVVTAGHCISSSLTYQVVLGEYDRAVKEGPEQVIPINSGDLFVHPLWNRSCVAC

:**:****:**.****:: **:*****:: . ** ** **: :.*:**** ** *.:*

gal GNDIALLKLRRNAVLNDYVQTGRLPPAGTVLPNGYPCYLSGWGRLTTGGPLPDKLQDALM

mus GNDIALVKLSRSAQLGDAVQLACLPPAGEILPNGAPCYISGWGRLSTNGPLPDKLQQALL

hum GNDIALIKLSRSAQLGDAVQLASLPPAGDILPNETPCYITGWGRLYTNGPLPDKLQEALL

pan GNDIALIKLSRSAQLGDAVQLASLPPAGDILPNETPCYITGWGRLYTNGPLPDKLQEALL

******:** *.* *.* ** . ***** :*** ***::***** *.********:**:

gal PVVDHEQCTQPDWWGSLAIRTTMICAGGAEQAGCNGDSGGPLNCQAEDGQWEVHGIASFV

mus PVVDYEHCSRWNWWGL-SVKTTMVCAGGDIQSGCNGDSGGPLNCPADNGTWQVHGVTSFV

hum PVVDYEHCSRWNWWGS-SVKKTMVCAGGDIRSGCNGDSGGPLNCPTEDGGWQVHGVTSFV

pan PVVDYEHCSRWNWWGS-SVKKTMVCAGGDIRSGCNGDSGGPLNCPTEDGGWQVHGVTSFV

****:*:*:: :*** :::.**:**** ::************ :::* *:***::***

gal SSLGCDTPKKPTVFTRVSAFNDWIAETMENNS

mus SSLGCNTLRKPTVFTRVSAFIDWIEETIANN-

hum SAFGCNTRRKPTVFTRVSAFIDWIEETIASH-

pan SAFGCNTRRKPTVFTRVSAFIDWIEETIASH-

*::**:* :*********** *** **: .:

1. Complement Factor-H related protein 2

gal MPFLGYAALLLCWMYCTAERACEQSPPRRVKEIPTETWDNPPYPHGTLVTYKCRPGYIKI

mus ------------------------------------------------------------

hum ------------------------------------------------------------

pan ------------------------------------------------------------

gal GRIVVKCADGVWKQQGNTECRSKPCGHPGDIEFGSFQLTAGNEFVFGARVEYRCNDGYRM

mus ------------------------------------------------------------

hum ------------------------------------------------------------

pan ------------------------------------------------------------

gal LSQKNYRECLAEGWSNDIPHCEVAKCLPVKAPENGRIVLSGAFELNREYSFGEVMEFECN

mus ------------------------------------------------------------

hum ------------------------------------------------------------

pan ------------------------------------------------------------

gal EHYRLVGSKAIHCSSNGKWDSDVPQCQDIICNVPSIPNGVVRSSQKTYRESEQLHYVCNK

mus ------------------------------------------------------------

hum ------------------------------------------------------------

pan ------------------------------------------------------------

gal GYTYGERADAQCTESGWSPTPYCTEVVCFPPTFRNGNFRPQKDRYTEGATITIDCDLGYR

mus ------------------------------------------------------------

hum ------------------------------------------------------------

pan ------------------------------------------------------------

gal YSTLTAKNVAKCTSSGWVPAPGCVEKPCDYPAVENIRLSGNWQQNYFPMRIGQTIYYRCR

mus ------------------------------------------------------------

hum ------------------------------------------------------------

pan ------------------------------------------------------------

gal EGYLTPSEEYWVHIVCSQGGWKPEPQCLKRCDVGPLENGYIQNNWKRYFKEGERTKYFCN

mus ------------------------------------------------------------

hum ------------------------------------------------------------

pan ------------------------------------------------------------

gal KNYRTENEDGEITCSKNGWSPTPRCIRKETCQWVDLTNGYFMERRATFDIGETVSYSCYN

mus ------------------------------------------------------------

hum ------------------------------------------------------------

pan ------------------------------------------------------------

gal DFVTPEKQKMGMIQCQKNGWSPPPKCIQTCKTSHIHTLVDCGPRENIYLPGDILEYSCPE

mus ------------------------------------------------------------

hum ------------------------------------------------------------

pan ------------------------------------------------------------

gal KYKTVDNMPYSTTRCDVNGEWKPTPHCVAIECKLPVLSHGQAHPSKNTYYNGDVVKFICV

mus ------------------------------------------------------------

hum ------------------------------------------------------------

pan ------------------------------------------------------------

gal KNYIRVGPASSQCYYFGWFPSPPTCKVDPRDCGPPPEITNGNVIGGFLERYQHGNRMEYE

mus ------------------------------------------------------------

hum ------------------------------------------------------------

pan ------------------------------------------------------------

gal CDTPFTLVGSKEIECLDGQWSSLPSCIENKMPCGSPSSVLNVVLLQQDRAQFSHGDEVTY

mus ------------------------------------------------------------

hum ------------------------------------------------------------

pan ------------------------------------------------------------

gal RCSQGSENATRMRTKCLNGEWKPSPLCNDPFHQCVTPEDIVIERTGSHRKAKETGLYLTY

mus ------------------------------------------------------------

hum ------------------------------------------------------------

pan ------------------------------------------------------------

gal KCKPADRQFKQATCVSGKWTPEIQCTGESTCPLPPQLPNANKIPIGRNYKNGSKIAFSCL

mus ------------------------------------------------------------

hum ------------------------------------------------------------

pan ------------------------------------------------------------

gal EGFHLIGANEIMCINGKWQSPPYCVEKPCSPPQPVEHADDVRLVNQSLKMEKEGKTVYLA

mus MGFCR---------------------------------------------------LLLL

hum --------------------------------------------------------MWLL

pan ---------------------------------------------------------YVT

:

gal GAVVKFTCHSGFELDGPSEISCSMGNWSSYPTCSEASCGSIPNVPNSAIEG----RNKEA

mus A-------------------IVLLTSWFSTAKGEV-SLCDFPKIRHGILYDEKKNEPFSS

hum V-------------------SVILISRISSVGGEA-MFCDFPKINHGILYDEEKYKPFSQ

pan Y-------------------YVIL---QFYVIFPA-TFCDFPKINHGILYDEEKYKPFSQ

: .:*:: :. : . . .

gal YEPGETIRYQCDEGFEAVG---VPEIICRKGNWSTPPFCEDVSCEAPPEIHNAYITSPQQ

mus VLSGKILYYSCEYNFASPSNSFWTRITCTESGWSPTPKCLRLCFFP--FVENGN-STSSG

hum VPTGEVFYYSCEYNFVSPSKSFWTRITCAEEGWSPTPKCLRLCFFP--FVENGH-SESSG

pan VPTGEVFYYSCEYNFVSPPKSFWTRITCTEEGWSPTPKCLRLCFFP--FVENGH-SESSG

*: : *.*: .* : .* * : .** * * :. :.*. : .

gal QRYLPGARVQYECDSHFQIT-AVNYVTCSNGQWLQTPTCKD--TRCGPPPEISGGKVQGF

mus QTHVEGDIVQVVCNQGYSLQNNQSTITCAEEGWSITPKCISTN-----------------

hum QTHLEGDTVQIICNTGYRLQNNEN**N**ISCVERGWSTPPKCRSTI-----------------

pan QTHLEGDTVQIICNTGYRLQKNENNISCVERGWSTPPKCRSTDTSCVNPPTVQNAHILSR

* :: * ** *: : : . ::* : * *.* .

gal KKSRYLPGETAKYECWKGFRMTGASTVSCQNGTWTELPTCKG---KSEKCGPPPDIENGD

mus ---------------------------------------------PTGKCGPPPPIDNGD

hum ---------------------------------------------SAEKCGPPPPIDNGD

pan QMSKYPSGERVRYQCRSPYEMFGDEEVMCLNGYWTEPPQCKECGPSITXCGPPPPIDNGD

***** *:***

gal ILSFPMPEYSQGETLKYKCPNLYILEGSQQITCINGQWTNPPVCLVACTAAEEDMNNNNI

mus ITSLSLPVYASLSSVEYQCQKYYLLKGNKTITCRNGKWSEPPTCIYPTGEM---------

hum ITSFLLSVYAPGSSVEYQCQNLYQLEGNNQITCRNGQWSEPPKCLDPCVISQEIMEKYNI

pan ITSFPLSVYAPASTVEYQCQNLYELEGNKRITCRNGQWSEPPKCLNPCVISREIMEKYNI

* *: : *: .:::*:* : * *:*.: *** **:*::** *:

gal ELKWREESKLYSRPGDFIEFDCKIGNVEDPASSPFRVQCINGTFKYPRCNPGRDCRVLES

mus ---WAPSTY---------------------------------------------------

hum KLKWTNQQKLYSRTGDIVEFVCKSGYHPT-KSHSFRAMCQNGKLVYPSCEEK--------

pan ALRWTARQKLYLRTGESVEFVCKYGYRLSPSSHTLRTTCWDGKLEYPTCVKR--------

*

gal EMDKNNIQLQWRMPHTYRSKERISFKCKWLYEPVSRPEKFKPRCLDGVIEYPQCAYRWK

mus -----------------------------------------------------------

hum -----------------------------------------------------------

pan -----------------------------------------------------------

1. IL-2 Receptor subunit alpha

gal MARPPGLLRAALLLLTAASSAPRRRCGPVALPQDTVLGRPGANVTLLCREREPPNGTVLW

mus ------------------------------------------------------------

hum ------------------------------------------------------------

pan ------------------------------------------------------------

gal SGRRRALGGGNALLLGGLRPEDAGRYSCHLGGHTLRTVRLLVEGGPRAPHVSCSRRSHDK

mus -----------------------------------MEPRLLMLGFLS-----LTIVPSCR

hum -----------------------------------MDSYLLMWGLLT-----FIMVPGCQ

pan -----------------------------------MDSYLLMWGLLT-----LIMVPGCQ

**: * :

gal DVLCEWRPRASPAPGTRAVLWMKRRFTMENATEQRCHFYSAAQKFVCRVKVPPGTDDTK-

mus AELCLYDPPEVPNATFKALSYKNG-------TILNCE-----------CKR--GFRRLKE

hum AELCDDDPPEIPHATFKAMAYKEG-------TMLNCE-----------CKR--GFRRIKS

pan AELCDDDPPEITHATFKAMAYKEG-------TMLNCE-----------CKR--GFRRIKS

** * :*: : : * .*. * * *

gal ALVVSVCVSSRAGSAAA---------------------E--DRIFTLNGILKPDPPLNVT

mus -LVYMRCLGN----SWSSNCQCTSNSHDKSRKQVTAQLEHQKEQQTTTDMQKPTQSMH--

hum GSLYMLCTG**N**SSHSSWDNQCQCTSSATR**N**TTKQVTPQPEEQKER-KTTEMQSPMQPVD--

pan GSLYMLCTGNSSHSSWDNQCQCTSSATRNTTKQVTPQPEEQKER-KTTEMQSPMQPVD--

: * .. : * .. . . : .* :.

gal VEAVERSPQRLCVRWSYPPSWDPR----FYWLRF--QVRYRPEPAPNFTQVDQVTRTWLD

mus -------QENLTGHCREPPPWKHEDSKRIYHFVEGQSVHYEC------------------

hum -------QASLPGHCREPPPWENEATERIYHFVVGQMVYYQC------------------

pan -------QASLPGHCREPPPWENEATERIYHFVVGQTVYYQC------------------

* : ** *. . :* : * *.

gal IRDAWRGMRHVVQVRAQEEFGHG-AWSEWSREAVGTPWTEPRDVTEMGLYSSQFPAEDDP

mus -IPGYKAL------------QRGPAISICKMKCGKTGWTQPQLTCVDEREHHRFLASEES

hum -VQGYRAL------------HRGPAESVCKMTHGKTRWTQPQLICTGEMETSQFPGEEKP

pan -VQGYRAL------------HRGPAESVCKMTHGKTRWTQPQLICTGEMETSQFPGEEKP

.::.: :* * * . * **:*: :* ..:.

gal YGYGATLPPELFGDD------TADDAGGAVLEATVRSPTSPYALLVAGGSLLLAIILCFA

mus QGSRNSSPESETSCPITTTDFPQPTETTAMTET--FVLTMEYKVAVASCL-FLLISILLL

hum QASPEGRPESETSCLVTTTDFQIQTEMAATMET--SIFTTEYQVAVAGCV-FLLISVLLL

pan QASPEGRPESETSCLITTTDFQIQTEMAATMET--FIFTTEYQVAVAGCV-FLLISVLLL

. * . . * *: * * : **. :* * : :

gal IGMRYKQRARAQRGAKLEGGGQHPMVPLCPPGSPLSATPLLSPAAPPGPLHVTNLDYFLS

mus SGLTWQHRWRKSRRTI--------------------------------------------

hum SGLTWQRRQRKSRRTI--------------------------------------------

pan SGLTWQRRQRKSRRTI--------------------------------------------

*: :::* * .* :

gal GK

mus --

hum --

pan --

1. Oxidized low-density lipo protein receptor 1

gal ------------------------------------------------------------

mus MTFDD-KMKPANDEPDQKSCGKKPKGLHLLSSPWWFPAAMTLVILCLVLSVTLIVQWTQL

hum MTFDDLKIQTVKDQPDEKSNGKKAKGLQFLYSPWWCLAAATLGVLCLGLVVTIMVLGMQL

pan MTFDDLKIQTVKDQPDEKSNGKKAKGLQFLYSPWWCLAAATLGVLCLGLVVTIMVLGMQL

gal --------------------------------------MEEVR---------------EY

mus RQVSDLLKQYQANLTQQDRILEGQMLAQQKAENTSQESKKELKGKIDTLTQKLNEKSKEQ

hum SQVSDLLTQEQA**N**LTHQKKKLEGQISARQQAEEASQESENELKEMIETLARKLN------

pan SQVSDLLTQEQANLTHQKKKLEGQISARQQAEEASQESQNELKEMIETLARKLN------

:*::

gal PHVL------GTEWSRR----E---GPSRGVCVTF--QLTMAAVFTVLLITAVAFAVQAF

mus EELLQKNQNLQEALQRAANSSEESQRELKGKIDTLTLKLNEKSKEQEELLQKNQNLQEAL

hum ----------------------------------------EKSKEQMELHHQNLNLQETL

pan ----------------------------------------EKSKEQMELHHQNLNLQETL

: * :::

gal QPHPQPCAQCPFDWIGFRGNCYYFSEDESNWTSSQNNCSALGASLAVFDSAEDLSFTMRH

mus QRAANFSGPCPQDWLWHKENCYLFH-GPFSWEKNRQTCQSLGGQLLQINGADDLTFILQA

hum KRVA**N**CSAPCPQDWIWHGENCYLFSSGSFNWEKSQEKCLSLDAKLLKINSTADLDFIQQA

pan KRVANCSAPCPQDWIWHGENCYLFSSGSFNWEKSQEKCLSLNAKLLKINSTADLDFIQQA

: : .. ** **: . *** * . .* ..::.* :*...* ::.: ** * :

gal --KGSSPHWVGLSREGKEHPWEWVNRSPLSHL-FQ-------VQGDGLCAYLGDAGLSSS

mus ISHTTSPFWIGLHRKKPGQPWLWENGTPLNFQFFKTRGVSLQLYSSGNCAYLQDGAVFAE

hum ISYSSFPFWMGLSRRNPSYPWLWEDGSPLMPHLFRVRGAVSQTYPSGTCAYIQRGAVYAE

pan ISYSSFPFWMGLSRRNPSYPWLWEDGSPLMPHLFRVRGAVSQTYPSGTCAYIQRGAVYAE

: *.*:** *. ** * : :** *: .* ***: ..: :.

gal HCSARRNWVCTKPALQKPRKNFCIST

mus NCILIAFSICQKKTNHLQI-------

hum NCILAAFSICQKKANLRAQ-------

pan NCILAAFSICQKKANLRAQ-------

:* :* * :

1. Kit ligand

gal MKKAQTWIITCFCLQLLLLNPLVKAQSSCGNPVTDDVNDIAKLVGNLPNDYLITLKYVPK

hum MKKTQTWILTCIYLQLLLFNPLVKTEGICRNRVTNNVKDVTKLVANLPKDYMITLKYVPG

pan MKKTQTWILTCIYLQLLLFNPLVKTEGICRNRVTNNVKDVTKLVANLPKDYMITLKYVPG

mus MKKTQTWIITCIYLQLLLFNPLVKTKEICGNPVTDNVKDITKLVANLPNDYMITLNYVAG

***:****:**: *****:*****:: * * **::*:*::***.***:**:***:**

gal MDSLPNHCWLHLMVPEFSRSLHNLLQKFSDISDMSDVLSNYSIINNLTRIINDLMACLAF

hum MDVLPSHCWISEMVVQLSDSLTDLLDKFS**N**IS---EGLSNYSIIDKLVNIVDDLVECVKE

pan MDVLPSHCWISEMVVQLSDSLTDLLDKFSNIS---EGLSNYSIIDKLVNIVDDLVECVKE

mus MDVLPSHCWLRDMVIQLSLSLTTLLDKFSNIS---EGLSNYSIIDKLGKIVDDLVLCMEE

** **.***: ** ::* ** **:***:** : *******::* .*::**: *:

gal DKNKDFIKENGHLYEEDRFIPENFFRLFNSTIEVYKEFADSLDKNDCIMPSTVETPENDS

hum **N**SSKDL-KKSFKSPEPRLFTPEEFFRIFNRSIDAFKDFVVASETSDCVVSSTL-SPEKDS

pan NSSKDL-KKSFKSPEPRLFTPEEFFRIFNRSIDAFKDFVVASETSDCVVSSTL-SPEKDS

mus NAPKNI-KESPKRPETRSFTPEEFFSIFNRSIDAFKDFMVASDTSDCVLSSTL-GPEKDS

: *:: *:. : * * **:** :** :*:.:*:* : :..**:: **: **:**

gal RVAVTKTISFPPVAASSLRNDSIGSNTSSNSNKEALGFISSSSLQGISIALTSLLSLLIG

hum RVSVTKPFMLPPVAASSLRNDSSSSNRKAK------NPPGDSSLHWAAMALPALFSLIIG

pan RVSVTKPFMLPPVAASSLRNDSSSSNRKAK------NPPGDSSLHWAAMALPALFSLIIG

mus RVSVTKPFMLPPVAASSLRNDSSSSNRKAA------KAPEDSGLQWTAMALPALISLVIG

**:*** : :************ .** .: .*.*: ::** :*:**:**

gal FILGAIYWKKTHPKSRPESNETIQCHGCQEENEISMLQQKEKEHLQV

hum FAFGALYWKKRQPSLTRAV-ENI--QINEEDNEISMLQEKEREFQEV

pan FAFGALYWKKRQPSLTRAV-ENI--QINEEDNEISMLQEKEREFQEV

mus FAFGALYWKKKQSSLTRAV-ENI--QINEEDNEISMLQQKEREFQEV

* :**:**** : . *.* : :*:*******:**:*. :*

1. Transmembrane protein 106B

gal MKKMGKSLSHLPIHTCKEDGYDGGTVSDNMRNGLVHSESHGEDGRCGDVSQFPYVEFTGR

hum ---MGKSLSHLPLHSSKEDAYDGVTS-ENMRNGLVNSEVHNEDGRNGDVSQFPYVEFTGR

pan ---MGKSLSHLPLHSSKEDAYDGVTS-ENMRNGLVNSEVHNEDGRNGDVSQFPYVEFTGR

mus ---MGKSLSHLPLHSNKEDGYDGVTSTDNMRNGLVSSEVHNEDGRNGDVSQFPYVEFTGR

*********:*: ***.*** * :******* ** *.**** **************

gal DSVTCPTCQGTGRIPRGQENQLVALIPYSDQRLRPRRTKLYVTASVIVCLLLSGLAVFFL

hum DSVTCPTCQGTGRIPRGQENQLVALIPYSDQRLRPRRTKLYVMASVFVCLLLSGLAVFFL

pan DSVTCPTCQGTGRIPRGQENQLVALIPYSDQRLRPRRTKLYVMASVFVCLLLSGLAVFFL

mus DSVTCPTCQGTGRIPRGQENQLVALIPYSDQRLRPRRTKLYVMASVFVCLLLSGLAVFFL

****************************************** ***:*************

gal FPRSVDVEYIGVKSVYVNYEQSRRIIYLNITNTLNITNNNYYSVEVANITAQVQFSKTVI

hum FPRSIDVKYIGVKSAYVSYDVQKRTIYL**N**ITNTL**N**ITNNNYYSVEVE**N**ITAQVQFSKTVI

pan FPRSIDVKYIGVKSAYVSYDVQKRTIYLNITNTLNITNNNYYSVEVENITAQVQFSKTVI

mus FPRSIEVKYIGVKSAYVSYDAEKRTIYLNITNTLNITNNNYYSVEVENITAQVQFSKTVI

****::*:******.**.*: .:* ********************* *************

gal GKARLNNITNIGPLDMKQIDYMVPTVIQDEMSYMFDFCTLASIKVHNIVVMMQVTVTTSY

hum GKARLN**N**ITIIGPLDMKQIDYTVPTVIAEEMSYMYDFCTLISIKVHNIVLMMQVTVTTTY

pan GKARLNNITIIGPLDMKQIDYTVPTVIAEEMSYMYDFCTLISIKVHNIVLMMQVTVTTTY

mus GKARLNNITNIGPLDMKQIDYTVPTVIAEEMSYMYDFCTLLSIKVHNIVLMMQVTVTTAY

********* *********** ***** :*****:***** ********:********:*

gal FGHSEQISREKYQYVDCGGNTTYQLGQSEYLNVLQPPQ

hum FGHSEQISQERYQYVDCGR**N**TTYQLGQSEYLNVLQPQQ

pan FGHSEQISQERYQYVDCGRNTTYQLGQSEYLNVLQPQQ

mus FGHSEQISQERYQYVDCGRNTTYQLAQSEYLNVLQPQQ

********:*:******* ******.********** *

1. Intercellular adhesion molecule 2

gal ---------------LLCTPLTPSPITPLAALLCASRSIRLSPPVLLVPYGGSLPFSCAT

hum MSSFGYRTLTVALFTLICCPGSDEK----------VFEVHVRPKKLAVEPKGSLEV**N**CST

pan MSSFSYRTLTVALFALICCPGSDEK----------VFEVHVRPKKLAVEPKGSLKVNCST

mus MSSFAC--WSLSLLILFYSPGSGEK----------AFEVYIWSEKQIVEATESWKINCST

*: * : . .: : * * ..*:*

gal TCADPNVSGGVETSSVYTTHLNVSGAVETSALYTLHPRGARMEVELHNVTEWNSTVQCFF

hum TCNQPEVGGL-ETSLD-------------KILLDE--QAQWKHYLVS**N**-ISHDTVLQCHF

pan TCNQPEVGGL-ETSLD-------------KILLDE--QAQWKHYLVSN-ISHDTVLQCHF

mus NCAAPDMGGL-ETPTN-------------KIMLEEHPQGKWKQFLVSN-VSKDTVFFCHF

.* *::.* ** . : :. . : * . ::.. *.*

gal RCYGARDGLKADLIAYRPLDQPELQPLPP-LRSGRAYNVSCVVPNVSPIRNLSLSLFRGD

hum TCSGKQESMNS**N**VSVYQPPRQVILTLQPTLVAVGKSFTIECRVPTVEPLDSLTLFLFRG**N**

pan TCSGKQESMNSNVSVYQPPRQVILTLQPTLVAVGKSFTIECRVPTVEPLDSLTLFLFRGN

mus TCSGKQHSESLNIRVYQPPAQVTLKLQPPRVFVGEDFTIECTVSPVQPLERLTLSLLRGR

* * :.. . :: .*:* * * * : *. :.:.* * *.*: *:* *:**

gal IAVHQRHLPGLPALPSDQRLADVRADGAAEGPRAERRLQGCARHAALRTAAHRQLHRARP

hum ETLHYETFGKAAPAPQEATATF**N**STADREDGH----R**N**FSCLAVLDLMSRGGNIFHKHSA

pan ETLHYETFGKAAPAPQEATVTFNSTADRDDGH----RNFSCLAVLDLMSRGGNIFHKHSA

mus ETLKNQTFGGAETVPQEATATFNSTALKKDG-----LNFSCQAELDLRPHGGYIIRSISE

::: . : *.: : : :* .* * . ::

gal GR-LR-------------------------------------------------------

hum PKMLEIYEPVSDSQMVIIVTVVSVLLSLFVTSVLLCFIFGQHLRQQRMGTYGVRAAWRRL

pan PKMLEIYEPVSDSQMVIIVTVVSVLLSLFVTSVLLCFIFGQHLRQQRMGTYGVRAAWRRL

mus YQILEVYEPMQDNQMVIIIVVVSILLFLFVTSVLLCFIFGQHWHRRRTGTYGVLAAWRRL

: *.

gal ---------

hum PQAFRP---

pan PQAFRP---

mus PRAFRARPV

1. Tryptase

gal MEGKTSPTTASPSSLHASAASSIFSIRPPQPRENVLGISFKPYSPDSIPAPVPCTACEST

mus ------------------------------------------------------------

hum ------------------------------------------------------------

pan ------------------------------------------------------------

gal RSSMFRAPCMSQRRLALIFCVSVLIVLIVALILLFMFWRSQTGIVYKEPAETCKDDPVRC

mus ------------------------------------------------------------

hum ------------------------------------------------------------

pan ------------------------------------------------------------

gal DGVVDCSQRSDELGCVRFSSDQSLLHVYSSTENQWLPVCSSAWDESFSRKTCRQLGFQNA

mus ------------------------------------------------------------

hum ------------------------------------------------------------

pan ------------------------------------------------------------

gal SQTEYVPLHVSGKSLTVADERDTIQQSLNS----SQCLTG-KFVSLRCTTCGQRISGRII

mus -----------------------MLKRLLLLLWALSLLASLVYSAPR--P--ANQRVGIV

hum --------------------------MLNLLLLALPVLASRAYAAPA--PGQALQRVGIV

pan --------------------------MLSLLLVALPVLASPAYAAPA--PGQALQRAGIV

* *:. : : *:

gal GGKETSVSKWPWQVSVQYGP---VHICGGTIIDAQWVLTAAHCFFMNSMKILDDWKVYGG

mus GGHEASESKWPWQVSLRFKLNYWIHFCGGSLIHPQWVLTAAHCVGPHIKS-PQLFRVQLR

hum GGQEAPRSKWPWQVSLRVHGPYWMHFCGGSLIHPQWVLTAAHCVGPDVKD-LAALRVQLR

pan GGQEAPRSKWPWQVSLRVRGKYWMHFCGGSLIHPQWVLTAAHCVGPDFKD-LAALRVQLR

**:*: ********:: :*:***::*. *********. . . :*

gal VSDLKQPMEGIPVSQVIINSNYSDDHDDYDIALMKLSRPLTLSAQIRPACLPMHGQRFQT

mus EQYLYYGDQLLSLNRIVVHPHYYTAEGGADVALLELEVPVNVSTHLHPISLPPASETFPP

hum EQHLYYQDQLLPVSRIIVHPQFYTAQIGADIALLELEEPV**N**VSSHVHTVTLPPASETFPP

pan EQHLYYQDQLLPVSRIIVHPQFYTAQIGADIALLELEEPVNISSRVHTVTLPPASETFPP

. * : : :.::::: :: . . *:**::*. *:.:*:::: ** .: *

gal GRSCFITGFGKTRENEDNTSP-KLREAEVKLIDYKICNSDKVYEGY-------LTPRMMC

mus GTSCWVTGWGDIDNDEPLPPPYPLKQVKVPIVENSLCDRKYHTGLYTGDDFPIVHDGMLC

hum GMPCWVTGWGDVDNDERLPPPFPLKQVKVPIMENHICDAKYHLGAYTGDDVRIVRDDMLC

pan GMPCWVTGWGDVDNDESLPPPFPLKQVKVPIMENHICDAKYHLGAYTGDNVRIVRDDMLC

* *::**:*. ::* * *::.:* ::: :*: . * : *:*

gal AGYLQGGKDACQGDSGGPLVCEDNGRWYVAGVTSWGTGCGQKNKPGVYTRVTKLLGWIYS

mus AGNT--RRDSCQGDSGGPLVCKVKGTWLQAGVVSWGEGCAQPNKPGIYTRVTYYLDWIHR

hum AGNT--RRDSCQGDSGGPLVCKV**N**GTWLQAGVVSWGEGCAQPNRPGIYTRVTYYLDWIHH

pan AGNT--RRDSCQGDSGGPLVCKVNGTWLQAGVVSWGEGCAQPNRPGIYTRVTYYLDWIHH

** :*:***********: :* * ***.*** **.* *:**:***** *.**:

gal KMESENN

mus YVPEHS-

hum YVPKKP-

pan YVPKKP-

: ..

1. Cation-dependent mannose-6-phosphate receptor

gal MGRARVVRQPWLLLWPLGSAPARRLRPVPEQPSFLRRAAVRAERTVHSSRMSSHCHTSAV

hum ------------------------------------------------MFPFYSCWRTGL

pan ------------------------------------------------MFPFYSCWRTGL

mus ------------------------------------------------MFPFSGCWRTEL

* : :

gal LVVFMALAAGVGAEPLSEKSCDVVGDESTESQMEKALLKKLEPLSQIRFNTTVEIGTTEN

hum LL-LLLAVAVRESWQTEEKTCDLVGEKGKESEKELALVKRLKPLFNKSFESTVGQGS-DT

pan LL-LLLAVAVRESWQTEEKTCDLVGEKGKESEKELALVKRLKPLF**N**KSFESTVGQGS-DT

mus LLLLLLAVAVRESWQIEEKSCDLVGEKDKESKNEVALLERLRPLFNKSFESTVGQGS-DT

*: :: .* : .**:**:**::..**: * **:::*.** : *::** *: :.

gal YAYHFRVCREVNSSLHDFAGLVQMDRQSGKTTVIGRINETQVFNGSDWIMLIYKGGDSYG

hum YIYIFRVCREAG**N**H-TSGAGLVQI**N**KSNGKETVVGRL**N**ETHIF**N**GSNWIMLIYKGGDEYD

pan YIYIFRVCREAGNH-TSGAGLVQINKSNGKETVVGRLNETHIFNGSNWIMLIYKGGDEYD

mus YSYIFRVCREASNH-SSGAGLVQINKSNDKETVVGRINETHIFNGSNWIMLIYKGGDEYD

* * ******... . *****:::...* **:**:***::****:**********.*.

gal RHCSGEKRRAVIMISCKRGITASSFSIISEEREKEQDCFYLFEMDSSVACPAEDSHLSTG

hum NHCGKEQRRAVVMISCNRHTLADNFNPVSEERGKVQDCFYLFEMDSSLACSPEISHLSVG

pan NHCGKEQRRAVVMISCNRHTLADNFNPVSEERGKVQDCFYLFEMDSSLACSPEISHLSVG

mus NHCGKEQRRAVVMISCNRHTLAANFNPVSEERGKVQDCFYLFEMDSSLACSPEVSHLSVG

.**. *:****:****:* * .*. :**** * ************:** * ****.*

gal SILLITFSALVTVYIVGGFLYQRLIVGAKGMEQFPHFAFWQDLGNLVADGCDFVCRSKPR

hum SILLVTFASLVAVYVVGGFLYQRLVVGAKGMEQFPHLAFWQDLGNLVADGCDFVCRSKPR

pan SILLVTFASLVAVYVVGGFLYQRLVVGAKGMEQFPHLAFWQDLGNLVADGCDFVCRSKPR

mus SILLVIFASLVAVYIIGGFLYQRLVVGAKGMEQFPHLAFWQDLGNLVADGCDFVCRSKPR

****: *::**:**::********:***********:***********************

gal NVPAAYRGVGDDQLGDESEERDDHLLPM

hum NVPAAYRGVGDDQLGEESEERDDHLLPM

pan NVPAAYRGVGDDQLGEESEERDDHLLPM

mus NVPAAYRGVGDDQLGEESEERDDHLLPM

***************:************

1. Protein canopy homolog-3

gal ---MAAEGPAALLLL------LLL-------VAVAGGDDADWVRLPSKCEVCKYVALELK

mus MESMSELAPRCLLFPLLLLLPLLLLPAPKLGPSPAGAEETDWVRLPSKCEVCKYVAVELK

hum MDSMPEPASRCLLLLPLLLLLLLLLPAPELGPSQAGAEENDWVRLPSKCEVCKYVAVELK

pan MDSLPEPASRCLLLLPLLLLLLLLLPAPELGPSQAGAEENDWVRLPSKCEVCKYVAVELK

: . .**: *** : **.:: ****************:***

gal SAFEETGKTKEVIDTKYGFLDGKGAAVKYTQSDIRLIEVTENICKRLLDYNLHKERSGSN

mus SAFEETGKTKEVIDTGYGILDGKGSGVKYTKSDLRLIEVTETICKRLLDYSLHKERTGSN

hum SAFEETGKTKEVIGTGYGILDQKASGVKYTKSDLRLIEVTETICKRLLDYSLHKERTGSN

pan SAFEETGKTKEVIGTGYGILDQKASGVKYTKSDLRLIEVTETICKRLLDYSLHKERTGSN

*************.* **:** *.:.****:**:*******.********.*****:***

gal RFAKGMSETFETLHNLVHKGVKVVMDIPYELWNETSAEVADLKKQCDVLVEEYEDVIEDW

mus RFAKGMSETFETLHNLVHKGVKVVMDIPYELWNETSAEVADLKKQCDVLVEEFEEVIEDW

hum RFAKGMSETFETLHNLVHKGVKVVMDIPYELW**N**ETSAEVADLKKQCDVLVEEFEEVIEDW

pan RFAKGMSETFETLHNLVHKGVKVVMDIPYELWNETSAEVADLKKQCDVLVEEFEEVIEDW

****************************************************:*:*****

gal YRHHQTEDLSQFLCADRVLKGKDASCLAEKWTGKKGDLASAGEKPSKKKSGKKKKKEGKE

mus YRNHQEEDLTEFLCANHVLKGKDTSCLAERWSGKKGDIASLGGKKSKKKRSGV--KGSSS

hum YRNHQEEDLTEFLCANHVLKGKDTSCLAEQWSGKKGDTAALGGKKSKKKSSRAKAAGGRS

pan YRNHQEEDLTEFLCANHVLKGKDTSCLAEQWSGKKGDTAALGGKKSKKKSSRAKAAGGRS

**:** ***::****::******:*****:*:***** *: * * **** . . .

gal Q---------SEGARSLPEEESGVQEAAPLPHSPTDEL

mus GSSKQRKELGGLGEDANAEEEEGVQKASPLPHSPPDEL

hum SSSKQRKELGGLEGDPSPEEDEGIQKASPLTHSPPDEL

pan SSSKQRKELGGLEGDPSPEEDEGIQKASPLTHSPPDEL

. **:.*:*:*:** *** ***

1. Na/K transporting ATPase 3

gal MSKETKKPFRQSVAEWRQFVYNPNSGEFLGRTAKSWGLILLFYLVFYGFLAALFTFTMWV

hum MTKNEKKSLNQSLAEWKLFIYNPTTGEFLGRTAKSWGLILLFYLVFYGFLAALFSFTMWV

pan MTKNEKKSLNQSLAEWKLFIYNPTTGEFLGRTAKSWGLILLFYLVFYGFLAALFSFTMWV

mus MTKTEKKSFHQSLAEWKLFIYNPSSGEFLGRTSKSWGLILLFYLVFYGFLAALFTFTMWA

*:* ** :.**:***: *:***.:*******:*********************:****.

gal MLQTLSNDIPKYRDRISSPGLMISPKPDTALEFYFNKSDAQSYAEYVSTLRKFLETYDDS

hum MLQTLNDEVPKYRDQIPSPGLMVFPKPVTALEYTFSRSDPTSYAGYIEDLKKFLKPYTLE

pan MLQTLNDEVPKYRDQIPSPGLMVFPKPVTALEYTFSRSDPTSYAGYIEDLKKFLKPYTLE

mus MLQTLNDEVPKYRDQIPSPGLMVFPKPQTALEYTFSMSEPQTYKKLVEDLESFLKPYSVE

*****.:::*****:* *****: *** ****: *. *: :* :. *..**: * .

gal KQSQNINCTPGKVFDQNDVAVKKACRFNLSELGQCSGKEDKTFGYSKGTPCVLVKMNRII

hum EQK**N**LTVCPDGALFEQKG-PVYVACQFPISLLQACSGMNDPDFGYSQGNPCILVKMNRII

pan EQKNLTVCPDGALFEQKG-PVYVACQFPISLLQACSGMNDPDFGYSQGNPCILVKMNRII

mus EQKNLTSCPDGAPFIQHG-PDYRACQFPVSLLEECSGVTDANFGYSKGQPCILVKMNRII

:*.: * * * *:. **:* :* * *** * ****:* **:********

gal GLKPEGEPYIQCTSKEPGAVEINYFPSGGLIDLMYFPYYGKTLHAHYLQPLVAVQLAINS

hum GLKPEGVPRIDCVSKNEDIPNVAVYPHNGMIDLKYFPYYGKKLHVGYLQPLVAVQVSFAP

pan GLKPEGVPRIDCVSKNEDIPNVAVYPHNGMIDLKYFPYYGKKLHVGYLQPLVAVQVSFAP

mus DLIPDGYPQISCLPKEEN-ATIATYPEFGVLDLKYFPYYGKKRHVGYRQPLVAVQVKFDS

.* *:* * *.* *: . : :* *::** *******. *. * *******: :

gal NSTNEEIAIECKILGSPNLKNEDDRDKFLGRIAFKVEMTE

hum **N**NTGKEVTVECKIDGSANLKSQDDRDKFLGRVMFKITARA

pan NNTGKEVTVECKIDGSANLKSQDDRDKFLGRVMFKITARA

mus GLNKKEVTVECHIAGTRNLKNKNERDKFLGRVSFKVTARA

. . :*:::**:* *: ***.:::*******: **:

1. Pre T cell antigen receptor- alpha

gal MEVPWLLLASA---LLLPPGGAADPLPTLAPPLTMMVSGQWRRLVVCVVSEL-PASSGHA

mus MARTWLLLLLGVRCQALPSGIAGTPFPSLAPPITLLVDGRQHMLVVCLVLDAAPPGLDNP

hum MAGTWLLLLLALGCPALPTGVGGTPFPSLAPPIMLLVDGKQQMVVVCLVLDVAPPGLDSP

pan MAGTWLLLLLALGCPALPTGVGGIPFPSLAPPIMLLVDGKQQMVVVCLVLDVAPPGLDSP

* **** . ** * .. *:*:****: ::*.*: : :***:* : * . .

gal VWISSGNGSALQSFAYGASQEEGGTVCAVSIL---PDSPMEQELLACHVGPNSSAPAHSS

mus VWFSAGNGSALDAFTYGPSLAPDGTWTSLAQLSLPSEELEAWEPLVCHTRPGAGGQNRST

hum IWFSAG**N**GSALDAFTYGPSPATDGTWTNLAHLSLPSEELASWEPLVCHTGPGAEGHSRST

pan IWFSAGNGSALDAFTYGPSPATDGTWTNLAHLSLPSEELASWEPLVCHTGPGAEGHSRST

:*:*:******::*:** * .** :: * :. * *.**. *.: . :*:

gal SPIAVAGSDEEAELCPSTPAEPRACAAIALLAAARVVLLKAALLDALLTALLLARR----

mus HPLQLSGESSTARSCFPEPLGGTQRQV-LWLSLLRLLLFKLLLLDVLLTCSHLRLH----

hum QPMHLSGEASTARTCPQEPLRGTPGGA-LWLGVLRLLLFKLLLFDLLLTCSCLCDPAGPL

pan QPVQLSGEASTARTCPQEPLRGTPGGA-LWLGVLRLLLFKLLLFDLLLTCSCLCDPAGLL

*: ::*. . *. * * . *. *::*:* *:* ***. *

gal ------------------------------------------------------------

mus ----------VLAGQHLQPPPS--------RKSLPPTHRIWT------------------

hum PSPATTTRLRALGSHRLHPATETGGREATSSPRPQPRDRRWGDTPPGRKPGSPVWGEGSY

pan PSPATTTRLRALGSHRLHLATETGGREATSSPRPQPRDCR--------------------

gal ------------------------------------------

mus ------------------------------------------

hum LSSYPTCPAQAWCSRSALRAPSSSLGAFFAGDLPPPLQAGAA

pan ------------------------------------------

1. TNF-14 receptor

gal ---------------MARGLFHLLLVIVLVMETHCINDTEVPTHTAHNKSITRKRNIAKR

mus MEPLPGWGSAPWSQAPTDNTFRLVPCVFLLN----------------------LLQRISA

hum MEPPGDWGPPPWRSTPKTDVLRLVLYLTFLG----------------------APCYAPA

pan MEPPGDWGPPPWRSTRKTDVLRLVLYLTFLG----------------------APCYAPA

. ::*: : ::

gal EITCGEGNYSFSGQCCTKCKRGHVKSIDC-PKTQAHCVPCKSGEEYMDHINDLDECMRCR

mus QPSCRQEEFLVGDECCPMCNPGYHVKQVCSEHTGTVCAPCPPQ-TYTAHANGLSKCLPCG

hum LPSCKEDEYPVGSECCPKCSPGYRVKEACGELTGTVCEPCPPG-TYIAHLNGLSKCLQCQ

pan LPSCKEDEYPVGSECCPKCSPGYRVKEACGELTGTVCEPCPPG-TYIAHLNGLSKCLQCQ

:* : :: ...:** *. *: . * * : * ** * * *.*.:*: *

gal SCDKALGLEVVKNCTSTENAECSCAKNHYCNSSRCEHCESCTV---CENGQ-IEKECTST

mus VCDPDMGLLTWQECSSWKDTVCRCIPGYFCENQDGSHCSTCLQHTTCPPGQRVEKRGTHD

hum MCDPAMGLRASR**N**CSRTENAVCGCSPGHFCIVQDGDHCAACRAYATSSPGQRVQKGGTES

pan MCDPAMGLRASRNCSRTENAVCGCSPGHFCIVQDGDHCAACRAYATSSPGQSVQKGGTES

** :** . ::*: ::: * * .::* . .** :* . ** ::* *

gal SDTVCRMQVKRKVNNYTTQGNTAAADTGKVHSPETLRLIHIDVDLTHHVPDIVREMTLRQ

mus QDTVCADCLTGTFSLGGTQEECLP---------WT------------------------N

hum QDTLCQNCPPGTFSP**N**GTLEECQH---------QT------------------------K

pan QDTLCQNCPPGTFSPNGTLEECQH---------QT------------------------K

.**:* ... * : * :

gal VITFVRHHRLSEPTIEETLLDNSNNTSEQKIKLFQKWYQKHGMGGAYETLICSLRDLKMC

mus CSAFQQEVR------------RGTNSTDTTCSSQVVYYVVSILL-PLVIVGAGIAGFLIC

hum CSWLVTKAG------------AGTSS------SHWVWWFLSGSL-VIVIV-CSTVGLIIC

pan CSWLVTKAG------------PGTSS------SHWVWWFLSGSL-VIVIV-CSTLGLIIC

: . ...: :: : .. .: :*

gal TAADKIERKLKAAVSSH-QERRESYNDKTEQSNTCSQEGEKCY---DDNAEISKTYPESL

mus TRRHLHTSSVAKELEPFQQEQQENTIRFPVTEVGFAE-------TEEETASN--------

hum VKRRKPRGDVVKVIVSVQRKRQEAEGEATVIEALQAPPDVTTVAVEETIPSFTGRSPNH-

pan VKRRKPRGDVVKVIVSVQRKRQEAEGEATVIEALQAPPDVTTVAVEETIPAFTGRSPNH-

. .: : ::::* . : :

gal EET

mus ---

hum ---

pan ---

1. CD 82 antigen

gal MGSGCLKVTKYFLFLFNLLFLILGAVILGFGIWILADKTSFISVLQNSSPSVRTGAYILV

hum MGSACIKVTKYFLFLFNLIFFILGAVILGFGVWILADKSSFISVLQTSSSSLRMGAYVFI

pan MGSACIKVTKYFLFLFNLIFFILGAVILGFGVWILADKSSFISVLQTSSSSLRMGAYVFI

mus MGAGCVKVTKYFLFLFNLLFFILGAVILGFGVWILADKNSFISVLQTSSSSLQVGAYVFI

**:.*:************:*:**********:******.*******.** *:: ***:::

gal GVGSLTMLMGFLGCLGAVNEIRCLLGLYFTCLMMILITQVAAGLVIYFQQEELKGELSRI

hum GVGAVTMLMGFLGCIGAVNEVRCLLGLYFAFLLLILIAQVTAGALFYFNMGKLKQEMGGI

pan GVGAVTMLMGFLGCIGAVNEVRCLLGLYFAFLLLILIAQVTAGALFYFNMGKLKQEMGGI

mus GVGAITIVMGFLGCIGAVNEVRCLLGLYFVFLLLILIAQVTVGVLFYFNADKLKKEMGNT

***::*::******:*****:********. *::***:**:.* ::**: :** *:.

gal VEKLIGDYDPVNGEDKNLQDAWDYVQKQLTCCGWNGAEEWEKNPILINKSMTAYPCSCSN

hum VTELIRDY**N**SS--REDSLQDAWDYVQAQVKCCGWVSFY**N**WTDNAELMNRPEVTYPCSCEV

pan VTELIRDYNSS--REDSLQDAWDYVQAQVKCCGWVSFYNWTHNAELMNRPEVTYPCSCEV

mus VMDIIRNYTAN--ATSSREEAWDYVQAQVKCCGWVSHYNWTENEELMGFTKTTYPCSCEK

* .:* :* .. ::****** *:.**** . :* .* *:. .:*****.

gal SSKDA---EENTGFCTLDVVVN-ETATHANWPVHRQGCVDGVQDWLKDNLGIILGVCTGV

hum KGEEDNSLSVRKGFCEAPG**N**RTQSGNHPEDWPVYQEGCMEKVQAWLQENLGIILGVGVGV

pan KGEEDNSLSVRKGFCEAPGNRTQSGNHPEDWPVYQEGCMEKVQAWLQENLGIILGVGVGV

mus IKEEDNQLIVKKGFCEADN-STVSENNPEDWPVNTEGCMEKAQAWLQENFGILLGVCAGV

:: ..*** . . :*** :**:: .* **::*:**:*** .**

gal AVVELLGMILSISLCKNIHSEDYTKVPKS

hum AIIELLGMVLSICLCRHVHSEDYSKVPKY

pan AIIELLGMVLSICLCRHVHSEDYSKVPKY

mus AVIELLGLFLSICLCRYIHSEDYSKVPKY

*::****:.***.**: :*****:****

1. TNF 13B

gal ----------------------MKSVDCVHVIQQKDTASSPSGPPGAASGTTGLFSVTFL

hum MDDSTER-EQSRLTSCLKKREEMKLKECVSILPRKESPSVRSSKDG------KLLA--AT

pan MDDSTER-EQSRLTSCLKKREEMKLKECVSILPRKESPSVRSSKDG------KLLA--AT

mus MDESAKTLPPPCLCFCSEKGEDMKVGYDPITPQKEEGAWFGICRDG------RLLA--AT

** ::: * *::

gal WLAMLLSSCLAAVSLYHAITLKTELEALRSELIYRVRARSPLEQPPVSPGDKKAGASVSS

hum LLLALLSCCLTVVSFYQVAALQGDLASLRAELQGHHAEKLP-----AGAGAPKAGLEEAP

pan LLLALLSCCLTVVSFYQVAALQGDLASLRAELQGHHAEKLP-----AGAGAPKAGLEEAP

mus LLLALLSSSFTAMSLYQLAALQADLMNLRMELQSYRGSATP-----AAAGA--------P

* ***..::.:*:*: :*: :* ** ** * .. *

gal FLQVSAAGARQENRLPGPSPAESFQTEIWDRNRNRGRRSIVNAEETVLQACLQLIADSKS

hum AVT---AGLK-IFEPPAPGEG**N**-------SSQNSRNKRAVQGPEETVTQDCLQLIADSET

pan AVT---AGLK-IFEPPAPGEGN-------SSQSSRNKRAVQGPEETVTQDCLQLIADSET

mus ELT---AGVK-LLTPAAPRPHN-------SSRGHRNRRAFQGPEETVIQDCLQLIADSDT

: ** : .* : . . *.:*:. . **** * ********.:

gal DIQQKDDSSIVPWLLSFKRGTALEEQGNKIVIKETGYFFIYGQVLYTDTTFAMGHLIQRK

hum PTIQKGSYTFVPWLLSFKRGSALEEKENKILVKETGYFFIYGQVLYTDKTYAMGHLIQRK

pan PTIQKGSYTFVPWLLSFKRGSALEEKENKILVKETGYFFIYGQVLYTDKTYAMGHLIQRK

mus PTIRKGTYTFVPWLLSFKRGNALEEKENKIVVRQTGYFFIYSQVLYTDPIFAMGHVIQRK

:*. ::**********.****: ***::::*******.****** :****:****

gal KAHVFGDDLSLVTLFRCIQNMPQSYPNNSCYTAGIAKLEEGDELQLTIPRRRAKISLDGD

hum KVHVFGDELSLVTLFRCIQNMPETLP**N**NSCYSAGIAKLEEGDELQLAIPRENAQISLDGD

pan KVHVFGDELSLVTLFRCIQNMPETLPNNSCYSAGIAKLEEGDELQLAIPRENAQISLDGD

mus KVHVFGDELSLVTLFRCIQNMPKTLPNNSCYSAGIARLEEGDEIQLAIPRENAQISRNGD

*.*****:**************:: ******:****:******:**:***..*:** :**

gal GTFFGAVRLL

hum VTFFGALKLL

pan VTFFGALKLL

mus DTFFGALKLL

*****::**

1. Translocon-associated protein

gal MRSVPRLLLLALLVFPAALLLRGSGTGSGLLVAAQDATEDEETVEDTVVEDEDDEAEVEE

mus MRLLPRLLLLFLLAFPAAVLLRGGPGG--SLALAQDPTEDEEIVEDSIIEDEDDEAEVEE

hum MRLLPRLLLLLLLVFPATVLFRGGPRG--LLAVAQDLTEDEETVEDSIIEDEDDEAEVEE

pan MRLLPRLLLLLLLVFPATVLFRGGPRG--SLAVAQDLTEDEETVEDSIIEDEDDEAEVEE

** :****** **.***::*:**. * *. *** ***** ***:::***********

gal DEPTDLTEEKEEEDLSGEPKASPSADTTILFVKGEDFPANNIVKFLVGFTNKGTEDFIVE

mus DEPTDLAEDKEEEDVSSEPEASPSADTTILFVKGEDFPANNIVKFLVGFTNKGTEDFIVE

hum DEPTDLVEDKEEEDVSGEPEASPSADTTILFVKGEDFPANNIVKFLVGFTNKGTEDFIVE

pan DEPTDLVEDKEEEDVSGEPEASPSADTTILFVKGEDFPANNIVKFLVGFTNKGTEDFIVE

******.*:*****:*.**:****************************************

gal SLDASFRYPQDYQFYIQNFTALPLNTVVPPQRQATFEYSFIPAEPMGGRPFGLVINLNYR

mus SLDASFRYPQDYQFYIQNFTALPLNTVVPPQRQATFEYSFIPAEPMGGRPFGLVINLNYK

hum SLDASFRYPQDYQFYIQ**N**FTALPLNTVVPPQRQATFEYSFIPAEPMGGRPFGLVINLNYK

pan SLDASFRYPQDYQFYIQNFTALPLNTVVPPQRQATFEYSFIPAEPMGGRPFGLVINLNYK

***********************************************************:

gal DANGNMFQDAVFNQTVTIIEKEDGLDGETIFMYMFLAGLGLLVIVGLHQLLESRKRKRPV

mus DLNGNVFQDAVFNQTVTVIEREDGLDGETIFMYMFLAGLGLLVVVGLHQLLESRKRKRPI

hum DLNGNVFQDAVF**N**QTVTVIEREDGLDGETIFMYMFLAGLGLLVIVGLHQLLESRKRKRPI

pan DLNGNVFQDAVFNQTVTVIEREDGLDGETIFMYMFLAGLGLLVIVGLHQLLESRKRKRPI

* ***:***********:**:**********************:***************:

gal QKVEMGTSNQNDVDMSWIPQETLNQINKASPRRLPRKRAQKRSVGSDEKTHPTRS

mus QKVEMGTSSQNDVDMSWIPQETLNQINKASPRRQPRKRAQKRSVGSDE-------

hum QKVEMGTSSQNDVDMSWIPQETLNQINKASPRRLPRKRAQKRSVGSDE-------

pan QKVEMGTSSQNDVDMSWIPQETLNQINKASPRRLPRKRAQKRSVGSDE-------

********.************************ **************

1. FCAR

gal -----------------------------SLSLHPSQGVSLGDNVTLRCHLPRQAAWVWL

mus MSPASPTFFCIGLCVLQVIQTQSGPLPKPSLQAQPSSLVPLGQSVILRCQGPPDVDLYRL

hum MDPKQTTLLCLVLCLGQRIQAQEGDFPMPFISAKSSPVIPLDGSVKIQCQAIREAYLTQL

pan MDPKQTTLLCLVLCLGQRIQAQEGDSPMPFISAKSSPVIPLDGSVKIQCQAIREAYLTQL

:. : * : *. .* ::*: :. *

gal YQEGGWSYKKRK----EKEQDTTEFLFDSTKGEHAGRYRCQYRVSESAELSVESDPVELV

mus EKLKPEKYED-----------QDFLFIPTMERSNAGRYRCSYQNGSHW--SLPSDQLELI

hum MIIK**N**STYREIGRRLKFW**N**ETDPEFVIDHMDANKAGRYQCQYRIGHYR--FRYSDTLELV

pan MIIKNSTYREIGRKLKFWNETDPEFVIDHMDANKAGRYQCQYRIGHYR--FRYSDTLELV

.*.. :.: . .:****:*.*: . ** :**:

gal LTDLRYPPSRISLHPEQHVGTGTNVTIRCWNKDYGA-TFLLHKDGSSDPIQRQESSGGGA

mus ATGVY-AKPSLSAHPSSAVPQGRDVTLKCQSP-YSFDEFVLYKEGDTGSYKRP--EKWYR

hum VTGLY-GKPFLSADRGLVLMPGE**N**ISLTCSSAHIPFDRFSLAKEGELSLPQHQ--SGEHP

pan VTGLY-GKPFLSADRGLVLMPGENISLTCSSAHIPFDRFSLAKEGELSLPQHQ--SGEHP

*.: :* . : * :::: * . * * *:*. . :: .

gal ANFTLLAVTPADSGTYRCSYRPRGYPFVSSPLGDSMTLEVTPTPAP-----SGGSEP---

mus ANFPIITVTAAHSGTYRCYSFSSSSPYLWSAPSDPLVLVVTGLSATPSQVPTEESFPVTE

hum A**N**FSLGPVDL**N**VSGIYRCYGWY**N**RSPYLWSFPSNALELVVTD------------------

pan ANFSLGPVDLNVSGIYRCYGWYNRSPYLWSFPSNALELVVTD------------------

*** : * ** *** *:: * .: : * **

gal ----PICMPPLS---------------WMGGCVSASSLH-----GGCGWQ----------

mus SSRRPSILPTNKISTTGFAHQHYAKGNLVRICLGATIIIILLGLLAEDWHSRKKCLQHRM

hum -----------------SIHQDYTTQNLIRMAVAGLVLVALLAILVENWHSHTALNKEAS

pan -----------------SIHQDYTIQNLIRMAVAGLVLVALLAILVENWHSHTALNKEAS

: .:.. : .*:

gal ---------------------------

mus RALQRPL-------PPLPLA-------

hum ADVAEPSWSQQMCQPGLTFARTPSVCK

pan ADVAEPRWSQQMCQPGLTFA-------

1. CD80

gal ------MKMGCLK----RWPLKRWLGLG---LGLIVLHCITLGCAQEKKVAKSKVGEKVG

mus MFSKASEAMACNCQLMQDTPLLKFPCPRLILLFVL-LIRLSQVSSDVDEQLSKSVKDKVL

hum --------MG----HTRRQGTSPSKCPYLNFFQLLVLAGLSHFCSG-VIHVTKEVKEVAT

pan --------MG----HTRRQGTSPSKCPYLNFFQLLVLAGLSHFCSG-VIHVTKEVKEVAT

*. : :: * :: .: ...* : .

gal LPCCYKIPSSESLQNYRVYWQMNVTDVVLAYAGEKKINEHPRYVNRTKLDFEN-LTLWIS

mus LPCRYNSPHE-DESEDRIYWQKHDKVVLSVIAGK--LKVWPEYKNRTLYDNTT-YSLIIL

hum LSCGH**N**VSVE-ELAQTRIYWQKEKKMVLTMMSGD--MNIWPEYK**N**RTIFDITN**N**LSIVIL

pan LSCGHNVSVE-ELAQTRIYWQKEKKMVLTMMSGD--MNIWPEYRNRTIFDITNNLSIVIL

* * :: . . : *:*** . . *: :*. :: *.* *** * . :: *

gal SVEILDSGPYQCIVQSLQSSPDKPGSHLLCGEPVTLFVTADFSKPNIEREVTASSCASTE

mus GLVLSDRGTYSCVVQKKERGTYEVK----HLALVKLSIKADFSTPNITESGNPS---ADT

hum ALRPSDEGTYECVVLKYEKDAFKRE----HLAEVTLSVKADFPTPSISDFEIPT---SNI

pan ALRPSDEGTYECVVLKYEKDAFKRE----HLAEVTLSVKADFPTPSISDFEIPT---SNI

.: * * *.*:* . : . : *.* :.*** .*.* : :

gal MVVRCSSHGGFPKPEIRGFLNDERV-VLNTTWESESSLSPYNVTGTLWLNMTKDSNFTCF

mus KRITCFASGGFPKPRFSWLENGRELPGINTTISQDPESELYTISSQLDFNTTRNHTIKCL

hum RRIICSTSGGFPEPHLSWLENGEELNAI**N**TTVSQDPETELYAVSSKLDF**N**MTT**N**HSFMCL

pan RRIICSTSGGFPEPHLSWLENGEELNAINTTVSQDPETELYAVSSKLDFNMTTNHSFMCL

: * : ****:*.: : *...: :*** ..: . . * ::. * :* * : .: *:

gal VEYDGLLRSTSLLLAKANDCIV--STALPSYNVITASSIIIITFVLAVTLAVKYLPRHAC

mus IKYGDAHVSEDFTWEKPPEDPPDSKNTLVLFGAGFGA---VITVVVIVVIIKCFCKHRSC

hum IKYGHLRV**N**QTFNW**N**TTKQEHFPD-NLLPSWAITLIS---VNGIFVICCLTYCFA--PRC

pan IKYGHLRVNQTFNWNTTKQEHFPD-NLLPSWAIILIS---VNGIFVICCLTYCFA--PRC

::*. . : . : . * : : : ..: : : *

gal SHCSKNQVSAEDDVRESM--NPPHSCKLTCEMSSL

mus FRRNEAS----RETNNSLTFGPEEALAE--QTVFL

hum RERRRNE----RLRRESV--RPV------------

pan RERRRNE----RLRRESV--RPV------------

. . . .:*: *

1. Na/K transporting ATPase subunit beta

gal -MSKETKKPFRQSVAEWRQFVYNPNSGEFLGRTAKSWGLILLFYLVFYGFLAALFTFTMW

hum MVIQKEKKSCGQVVEEWKEFVWNPRTHQFMGRTGTSWAFILLFYLVFYGFLTAMFTLTMW

pan MVIQKEKKSCGQVVEEWKEFVWNPRTHQFMGRTGTSWAFILLFYLVFYGFLTAMFTLTMW

mus MVIQKEKKSCGQVVEEWKEFVWNPRTHQFMGRTGTSWAFILLFYLVFYGFLTAMFSLTMW

: :: ** * * **::**:**.: :*:***..**.:************:*:*::***

gal VMLQTLSNDIPKYRDRISSPGLMISPKPDTALEFYFNKSDAQSYAEYVSTLRKFLETYDD

hum VMLQTVSDHTPKYQDRLATPGLMIRPKTE-NLDVIV**N**VSDTESWDQHVQKLNKFLEPY**N**D

pan VMLQTVSDHTPKYQDRLATPGLMIRPKTE-NLDVIVNVSDTESWDQHVQKLNKFLEPYND

mus VMLQTVSDHTPKYQDRLATPGLMIRPKTE-NLDVIVNISDTESWGQHVQKLNKFLEPYND

*****:*:. ***:**:::***** ** : *:. .* **::*: ::*..*.**** *:*

gal SKQSQ-NINCTPGKVFDQNDVAV----KKACRFNLSELGQCSGKED-KTFGYSKGTPCVL

hum SIQAQKNDVCRPGRYYEQPDNGVLNYPKRACQF**N**RTQLG**N**CSGIGDSTHYGYSTGQPCVF

pan SIQAQKNDVCRPGRYYEQPDNGVLNYPKRACQFNRTQLGNCSGIGDSTHYGYSTGQPCVF

mus SIQAQKNDVCRPGRYYEQPDNGVLNYPKRACQFNRTQLGDCSGIGDPTHYGYSTGQPCVF

* *:* * * **: ::* * .* *:**:** ::**:*** * . :***.* ***:

gal VKMNRIIGLKPEGEPYI--QCTSKEP----GAVEINYFPSGGLIDLMYFPYYGKTLHAHY

hum IKM**N**RVI**N**FYAGANQSMNVTCAGKRDEDAENLGNFVMFPANGNIDLMYFPYYGKKFHV**N**Y

pan IKMNRVINFYAGANQSMNVTCAGKRDEDAENLGNFVMFPANGNIDLMYFPYYGKKFHVNY

mus IKMNRVINFYAGANQSMNVTCVGKRDEDAENLGHFVMFPANGSIDLMYFPYYGKKFHVNY

:****:*.: .: : *..*. . .: **:.* ***********.:*.:*

gal LQPLVAVQLAINSNSTNEEIAIECKILGSPNLKNEDDRDKFLGRIAFKVEMTE-

hum TQPLVAVKFL**N**--VTPNVEVNVECRIN-AANIATDDERDKFAGRVAFKLRINKT

pan TQPLVAVKFLN--VTPNVEVNVECRIN-AANIATDDERDKFAGRVAFKLRINKT

mus TQPLVAVKFLN--VTPNVEVNVECRIN-AANIATDDERDKFAGRVAFKLRINKT

******:: : * *: :**:* : *: .:*:**** **:***:.:.:

1. ER-golgi compartment protein

hum MPFDFRRFDIYRKVPKDLTQPTYTGAIISICCCLFILFLFLSELTGFITTEVVNELYVDD

pan MPFDFRRFDIYRKVPKDLTQPTYTGAIISICCCLFILFLFLSELTGFITTEVVNELYVDD

mus MPFDFRRFDIYRKVPKDLTQPTYTGAIISICCCLFILFLFLSELTGFITTEVVNELYVDD

gal MPFDFRRFDIYRKVPKDLTQPTYTGAIISVCCCLFILFLFLSELTGFIATEIVNELYVDD

*****************************:******************:**:********

hum PDKDSGGKIDVSL**N**ISLPNLHCELVGLDIQDEMGRHEVGHIDNSMKIPLNNGAGCRFEGQ

pan PDKDSGGKIDVSLNISLPNLHCELVGLDIQDEMGRHEVGHIDNSMKIPLNNGAGCRFEGQ

mus PDKDSGGKIDVSLNISLPNLHCELVGLDIQDEMGRHEVGHIDNSMKIPLNNGAGCRFEGQ

gal PDKDSGGKIEVNLNISLPNLHCELVGLDIQDEMGRHEVGHIDNSMKIPLNNGDGCRFEGH

*********:*.**************************************** ******:

hum FSINKVPGNFHVSTHSATAQPQNPDMTHVIHKLSFGDTLQVQNIHGAFNALGGADRLTSN

pan FSINKVPGNFHVSTHSATAQPQNPDMTHVIHKLSFGDTLQVQNIHGAFNALGGADRLTSN

mus FSINKVPGNFHVSTHSATAQPQNPDMTHTIHKLSFGDTLQVQNVHGAFNALGGADRLTSN

gal FSINKVPGNFHVSTHSATAQPQNPDMTHIIHKLSFGDKLQVQNVHGAFNALEGADKLSSN

**************************** ********.*****:******* ***:*:**

hum PLASHDYILKIVPTVYEDKSGKQRYSYQYTVANKEYVAYSHTGRIIPAIWFRYDLSPITV

pan PLASHDYILKIVPTVYEDKSGKQRYSYQYTVANKEYVAYSHTGRIIPAIWFRYDLSPITV

mus PLASHDYILKIVPTVYEDKSGKQRYSYQYTVANKEYVAYSHTGRIIPAIWFRYDLSPITV

gal PLASHDYILKIVPTVYEDMSGKQRYSYQYTVANKEYVAYSHTGRIIPAIWFRYDLSPITV

****************** *****************************************

hum KYTERRQPLYRFITTICAIIGGTFTVAGILDSCIFTASEAWKKIQLGKMH

pan KYTERRQPLYRFITTICAIIGGTFTVAGILDSCIFTASEAWKKIQLGKMH

mus KYTERRQPLYRFITTICAIIGGTFTVAGILDSCIFTASEAWKKIQLGKIH

gal KYTERRQPLYRFITSICAIIGGTFTVAGILDSCIFTASEAWKKIQLGKMQ

**************:*********************************::

1. Programmed cell death ligand

gal MSRVRFLFSGCFRLGNWGHYSSTEGTYQVITSDLVIGGLLSAGSAHCRKRLNFQPSDASP

hum ------------------------------------------------------------

pan ------------------------------------------------------------

mus ------------------------------------------------------------

gal RPLVFGNLGSGDGNGSTGTLRAPLLDPPASQTLSFFFDFIYRKPLAFGNSKLPGTARTTP

hum ------------------------------------------------------------

pan ------------------------------------------------------------

mus ------------------------------------------------------------

gal FRACRDAAPARASPSPALFGHISAADDRSSPAGSTYMMEKLLLLHIFLFCWRSLNALFTV

hum --------------------------------------MRIFAVFIFMTYWHLLN-AFTV

pan --------------------------------------MRIFAVFIFMTYWHLLN-AFTV

mus --------------------------------------MRIFAGIIFTACCHLLR-AFTI

::: ** : *. **:

gal EAPKSLYTAELGSNVTMECVFPVNGKLKFRDLSVIWEKKDEVRKDVYILLKGKEDSGSQH

hum TVPKDLYVVEYGS**N**MTIECKFPVEKQLDLAALIVYWEMEDK---NIIQFVHGEEDLKVQH

pan TVPKDLYVVEYGSNMTIECKFPVEKQLDLAALIVYWEMEDK---NIIQFVHGEEDLKVQH

mus TAPKDLYVVEYGSNVTMECRFPVERELDLLALVVYWEKEDE---QVIQFVAGEEDLKPQH

.**.**..* ***:*:** ***: :*.: * * ** :*: :: :: *:** **

gal SDFQGRIKLLKENLDFGQSLLQISNVKLRDAGLYHCLIEYGGADYKTINLKVQAPYRTIT

hum SSYRQRARLLKDQLSLGNAALQITDVKLQDAGVYRCMISYGGADYKRITVKVNAPYNKIN

pan SSYRQRARLLKDQLSLGNAALQITDVKLQDAGVYRCMISYGGADYKRITVKVNAPYNKIN

mus SNFRGRASLPKDQLLKGNAALQITDVKLQDAGVYCCIISYGGADYKRITLKVNAPYRKIN

*.:: * * *::* *:: ***::***:***:* *:*.******* *.:**:***..*.

gal QEV--VSTGDKEWKLTCQSEGYPKAEVMWQNGECQDLTDKANTSYETGSDQLYRVTSTLT

hum QRILVVDPVTSEHELTCQAEGYPKAEVIWTSSDHQVLSGKTTTTNSKREEKLF**N**VTSTLR

pan QRILVVDPVTSEHELTCQAEGYPKAEVIWTSSDHQVLSGKTTTTNSKREEKLFNVTSTLR

mus QRIS-VDPATSEHELICQAEGYPEAEVIWTNSDHQPVSGKRSVTTSRTEGMLLNVTSSLR

*.: *. .* :* **:****:***:* ..: * ::.* ..: . . * .***:*

gal VKNRTCENFRCIFWNKEIQENTSANLYILARASKDC---RTRMA-KSSIH----------

hum I**N**TTTNEIFYCTFRRLDPEE**N**HTAELVIPELPLAHPPNERTHLVILGAILLCLGVALTF-

pan INTTTNEIFYCTFRRLDPEENHTAELVIPELPLAHPPNERTHLVILGAILLCLGVALTF-

mus VNATANDVFYCTFWRSQPGQNHTAELIIPELPATHPPQNRTHWVLLGSILLFLIVVSTVL

:: : : * * * . : :* :*:* * . **: . .:*

gal I--TKLSKDKGAHDCRGPSFEDAELKYIQIETT

hum IFRLRKGRMMDVKKCGIQDTNSKKQSDTHLEET

pan IFCLRKGRMMDVKKCGIQDTNSKKQSDTHLEET

mus LFLRKQVRMLDVEKCGVEDTSSKNRNDTQFEET

: : : ....* . .. : . ::* *

1. Glycolipid transfer protein 2

gal ------------------------------------------------------------

mus MVMGVS-LSPALGRWFRHAIPFAILTLLLLYISIWFFYEWPFPLPAQRTQQSGLRGLKLP

hum --MGVAARPPALRHWFSHSIPLAIFALLLLYLSVRSLG----------------------

pan --MGVAVRPPALRHWFSHSIPLAIFALLLLYLSVRSLG----------------------

gal -------------------------------------------MAAAPGAFSLREVLAGF

mus SPSPVLGSLLSFPAGVQSCNPERPLPSQTGPAARPLVVPEKEELPCLGPHGALGRMVSPF

hum -------ARSGCGPRAQPCVPGETAPFQVRQESGTLEAPERKQPPCLGPRGMLGRMMRRF

pan -------ARSGCGPRAQPCVPGGTAPFQVRQESGTLEAPERKQPPCLGPRGMLGRMMRRF

. * .:: *

gal QECVTEQREVLLGPYLCGWRGLVRFLNGLGAIFSFISKDAVAKIQIMENYCGGERREEYR

mus LACMSPEGDVALSQYLAGWRELLRFLTPLGTVFAFATSEAFNKVTDLEARVHGPNASHYT

hum HASLKPEGDVGLSPYLAGWRALVEFLTPLGSVFAFATREAFTKVTDLEARVHGPDAEHYW

pan HASLKPEGDVGLSPYLAGWRALVQFLTPLGSVFAFATREAFTKVTDLEARVHGPDAEHYW

.:. : :* *. **.*** *:.**. **::*:* : :*. *: :* * ..*

gal TLQAMVRYELSGGLVDLQR---RSAHPDSGCRTVLRLHRALRWLQLFLEGLRTARQD-AS

mus SLMTMITWERGAGLLQRPGTEPGHSAGSSGSRTLLLLHRALRWSQLCLHRVATGTLGGPD

hum SLVAMAAWERRAGLLEQPGAAPRDPTRSSGSRTLLLLHRALRWSQLCLHRVATGALGGPD

pan SLAAMAAWERRAGLLEQPGAAPRDLTRSSGSRTLLLLHRALRWSQLCLHRVATGALGGPD

:* :* :* .**:: .**.**:* ******* ** *. : *. . .

gal TSAICTDSYNASLAAYHPWVVRKAAVVAFCTLPSRDAFLEVMNVGGPEE-AVEMLGDALP

mus AGTQCGEAYSTALAPHHPWLIRQAARLAILALPSRGRLLQLACPGTGEADARVALARAAG

hum AGVQCSDAYRAALGPHHPWLVRQTARLAFLAFPGRRRLLELACPGATEAEARAALVRAAG

pan AGVQCSDAYRAALGSHHPWLVRQTARLAFLAFPGRRRLLELACPGATEAEARAALVRAAG

:.. * ::* ::*. :***::*::* :*: ::*.* :*:: * * * * *

gal HIRNVYGITQELYEQHHLLDLP

mus VLEDVYNRTQGLLAGHGLLQLA

hum TLEDVY**N**RTQSLLAERGLLQLA

pan TLEDVYNRTQSLLAERGLLQLA

:.:**. ** * : **:*

1. Insulin-like GF

gal MVSRPGVLWAVAAAALALVGR---------RALPAPVVRCEPCDARALQQCKP--LQPDC

hum MQRARPTLWAAALTLLVLLRGPPVARAGASSAGLGPVVRCEPCDARALAQCAPPPA--VC

pan MQRARPTLWAAALTLLVLLRGPPVARAGASSAGLGPVVRCEPCDARALAQCAPPPA--VC

mus MHPARPALWAAALTALTLLRGPPVAELAAGAV-GGPVVRCEPCDARAVSQCAPPPTAPAC

* .***.* : *.*: . .************: ** * *

gal AERVREPGCGCCLTCALRLGQPCGIYTERCGAGLNCQPRQEEARPLQALLEGRGFCTNAT

hum AELVREPGCGCCLTCALSEGQPCGIYTERCGSGLRCQPSPDEARPLQALLDGRGLCV**N**AS

pan AELVREPGCGCCLTCALSEGQPCGIYTERCGSGLRCQPSPDEARPLQALLDGRGLCVNAS

mus TELVREPGCGCCLTCALREGDACGVYTERCGTGLRCQPRPAEQYPLRALLNGRGFCANAS

:* ************** *: **:******:**.*** * **:***:***:*.**:

gal AGDKLRAFLLPGPHAAGNSSDSEEDRSTSSLENQAIPSLHRVPDSNLPPQHIRIDIIRKE

hum AVSRLRAYLLPAPPAPG**N**ASESEEDRSAGSVESPSVSSTHRVSDPKFHPLHSKIIIIKKG

pan AVSRLRAYLLPAPPAPGNASESEEDRSAGSVESPSVSSTHRVSDPKFHPLHSKIIIIKKG

mus AAGSLSTY-LPSQPAPGNISESEEEHNAGSVESQVVPSTHRVTDSKFHPLHAKMDVIKKG

* . * :: **. * ** *:***::.:.*:*. : * *** * :: * * :: :*:*

gal QAKNTQRYKVEYDSQSTDTLNFSSESKQETEYGPCRREMEDTLNHLKILNVLSPRGFHIP

hum HAKDSQRYKVDYESQSTDTQ**N**FSSESKRETEYGPCRREMEDTLNHLKFLNVLSPRGVHIP

pan HAKDSQRYKVDYESQSTDTQNFSSESKRETEYGPCRREMEDTLNHLKFLNVLSPRGVHIP

mus HARDSQRYKVDYESQSTDTQNFSSESKRETEYGPCRREMEDTLNHLKFLNVLSPRGVHIP

:*:::*****:*:****** *******:*******************:********.***

gal NCDKKGFYKKKQCRPSKGRKRGYCWCVDKYGQPLPGYDGKGKGDVHCYNLGSK

hum NCDKKGFYKKKQCRPSKGRKRGFCWCVDKYGQPLPGYTTKGKEDVHCYSMQSK

pan NCDKKGFYKKKQCRPSKGRKRGFCWCVDKYGQPLPGYTTKGKEDVHCYSMQSK

mus NCDKKGFYKKKRCRPSKGRKQSFCWCVDKYGQRLPGYDTKGKDDVHCLSVQSQ

***********:********:.:********* **** *** **** .: *:

1. Corticosteroid beta dehydrogenase

gal MGLLQKILIPVLGLVLAFCFYSSRENFRPEMLKGKRVIVTGASTGIGEQMAYHLARMGAH

mus MAVMKNYLLPILVLFLAYYYYSTNEEFRPEMLQGKKVIVTGASKGIGREMAYHLSKMGAH

hum MAFMKKYLLPILGLFMAYYYYSANEEFRPEMLQGKKVIVTGASKGIGREMAYHLAKMGAH

pan MAFMKKYLLPILGLFMAYYYYSANEEFRPEMLQGKKVIVTGASKGIGREMAYHLAKMGAH

*..::: *:*:* *.:*: :**:.*:******:**:*******.***.:*****::****

gal VLVTARTEAKLQQVVERCRALGAGSARLVSGSMEDMATTRQLVEVAEAELGGLDMLILNH

mus VVLTARSEEGLQKVVSRCLELGAASAHYIAGTMEDMTFAEQFIVKAGKLMGGLDMLILNH

hum VVVTARSKETLQKVVSHCLELGAASAHYIAGTMEDMTFAEQFVAQAGKLMGGLDMLILNH

pan VVVTARSKETLQKVVSHCLELGAASAHYIAGTMEDMTFAEQFVAQAGKLMGGLDMLILNH

*::***:: **:**.:* ***.**: ::*:****: :.*:: * :**********

gal VGKSYFNYFDGDVGHVQKLLNINFLSYVAMTVSALPMLKRSGGSIVVVSSMAGKVGFPFT

mus ITQTSLSLFHDDIHSVRRVMEVNFLSYVVMSTAALPMLKQSNGSIAVISSLAGKMTQPMI

hum IT**N**TSLNLFHDDIHHVRKSMEVNFLSYVVLTVAALPMLKQS**N**GSIVVVSSLAGKVAYPMV

pan ITNTSLNLFHDDIHHVRKSMEVNFLSYVVLTVAALPMLKQSNGSIVVVSSLAGKVAYPMV

: :: :. *..*: *:: :::******.::.:******:*.***.*:**:***: *:

gal VPYSATKFALDGFFSSLRQEFSIQSVNVSITLCILGFIDTENAMRAAADVLLMSPAPKEE

mus APYSASKFALDGFFSTIRTELYITKVNVSITLCVLGLIDTETAMKEISGIINAQASPKEE

hum AAYSASKFALDGFFSSIRKEYSVSRV**N**VSITLCVLGLIDTETAMKAVSGIVHMQAAPKEE

pan AAYSASKFALDGFFSSIRKEYSVSKVNVSITLCVLGLIDTETAMKAVSGIVHMQAAPKEE

. ***:*********::* * : ********:**:****.**: :.:: . :****

gal CALEIIKGGTLRQREVYYRYASTKLPLLLRDWAAELLDLLVRQRYRPERLRAA

mus CALEIIKGTALRKSEVYYDKSP-LTPILLGNPGRKIMEFFSLRYYNKDMFVSN

hum CALEIIKGGALRQEEVYYDSSL-WTTLLIRNPCRKILEFLYSTSYNMDRFINK

pan CALEIIKGGALRQEEVYYDSSL-WTTLLIRNPCRKILEFLYSTSYNMDRFINK

******** :**: **** : :*: : :::::: *. : :

1. Kallikrein

gal ------------------------------------------------------------

mus MARAGHPWKWAMATLITTLVLG--------------------------------------

hum MATARPPWMWVLCALITALLLG--------------------------------------

pan MATARPPWMWVLCALITALLLGVTEVVITVERCDCVLLAGDYVIVAECDVMDACICDRAA

gal ------------------------------------------------------------

mus ------------------------------------------------------------

hum ------------------------------------------------------------

pan QERVYLETELYVSCPCGGNMYVSAELRACLAVTAVVRVVLGAPAPTALALPLSLSRNPAW

gal ----------------------------------------------------------MN

mus ----------------------------------------------------------VS

hum ----------------------------------------------------------VT

pan ALSLSLSLGLRGATLILQALPLPPQRVRHPPSSWSGLLSLKPTPSPSPASEPRPTHFPFP

.

gal SL---FLILSCLGAAVAFP------------------GGADDDKIVGGYTCPEHSVPYQV

mus EPVLAGDVSSCDNPSGTEPSGSNRDLSTDSKSGEDT-RSDSSSRIVNGSDCQKDAQPWQG

hum EHVLANNDVSCDHPSNTVPSGSNQDLGAGA--GEDARSDDSSSRII**N**GSDCDMHTQPWQA

pan EHVLANNDVSCDHPSNTVPSGSNQDLGAGA--GEDARSDDSSSRIINGSDCDKHTQPWQA

. ** : : * . ...:*:.* * .: *:*

gal SL--NSGYHFCGGSLINSQWVLSAAHCYKSRIQVRLGEYNIDVQED-SEVVRSSSVIIRH

mus ALLLGPNKLYCGAVLISPQWLLTAAHCRKPVFRIRLGHHSMSPVYESGQQMFQGIKSIPH

hum ALLLRPNQLYCGAVLVHPQWLLTAAHCRKKVFRVRLGHYSLSPVYESGQQMFQGVKSIPH

pan ALLLRPNQLYCGAVLVHPQWLLTAAHCRKKVFRVRLGHYSLSPVYESGQQMFQGVKSIPH

:* . :**. *: **:*:**** * :::***.:.:. : .: : .. * *

gal PKYSSITLNNDIMLIKLASAVEYSADIQPIALPSSCAKAGTECLISGWGNTLSNGYNYPE

mus PGYSHPGHSNDLMLIKMNRKIRDSHSVKPVEIACDCATEGTRCMVSGWGTTSSSHNNFPK

hum PGYSHPGHSNNLMLIKLNRRIRPTKDVRPI**N**VSSHCPSAGTKCLVSGWGTTKSPQVHFPK

pan PGYSHPGHSNDLMLIKLNRRIRPTKDVRPINVSSHCPSAGTKCLVSGWGTTKSPQVRFPK

* ** .*::****: :. : .::*: : . * . **.*::****.* * .:*:

gal LLQCLNAPILSDQECQEAYPGDITSNMICVGFLEGGKDSCQGDSGGPVVCNGELQGIVSW

mus VLQCLNITVLSEERCKNSYPGQIDKTMFCAGDE-EGRDSCQGDSGGPVVCNGKLQGLVSW

hum VLQCL**N**ISVLSQKRCEDAYPRQIDDTMFCAGDK-AGRDSCQGDSGGPVVC**N**GSLQGLVSW

pan VLQCLNISVLSQKRCEDAYPRQIDDTMFCAGDK-AGRDSCQGDSGGPVVCNGSLQGLVSW

:***** :**::.*:::** :* ..*:*.* *:***************.***:***

gal G-IGCALKGYPGVYTKVCNYVDWIQETIAAY-

mus GDFPCAQRNRPGVYTNLCEFVKWIKDTMNSN-

hum GDYPCARPNRPGVYTNLCKFTKWIQETIQANS

pan GDYPCARPNRPGVYTNLCKFTKWIQETIQANS

* ** . *****::*::..**::*: :

1. Dehydrodolichoyl diphosphate synthase

gal MSAVGGLAWRALHALLRALLCLQRALLA--APRAWLRR--RAAASCALLGPAARALAFPG

mus MTGLYELVWRVLHALLCLHLTLTSWLRVRFGTWNWIWRRCCRAASAAVLAPLGFTLRKPR

hum MTGLYELVWRVLHALLCLHRTLTSWLRVRFGTWNWIWRRCCRAASAAVLAPLGFTLRKPP

pan MTGLYELVWRVLHALLCLHRTLTSWLRVRFGTWNWIWRRCCRAASAAVLAPLGFTLRKSP

*:.: *.**.***** * * . . *: * ***.*:*.* . :*

gal AARGVRR------------PGRRRPGVRARGRADGRALQKLPLHVGLVVTEE--EPSYAD

mus AVGRNRRHHRHPHGGPGPGPGPAATHPRLRWRADVRSLQKLPVHMGLLVTEEVQEPSFSD

hum AVGRNRRHHRHPRGGSC----LAAAHHRMRWRADGRSLEKLPVHMGLVITEVEQEPSFSD

pan AVGRNRRHHRHPRGGSC----LAAAHHRMRWRADGRSLEKLPVHMGLVITEVEQEPSFSD

*. ** * * *** *:*:***:*:**::** ***::*

gal MASLVVWCMAVGISYVSVYDHNGIFKRNNSRLMDEILKQEQELLGLDCSKYTVEFANQ-D

mus IASLVVWCMAVGISYISVYDHQGIFKRNNSRLMDEILKQQQELLGQDCSKYSAEFANSND

hum IASLVVWCMAVGISYISVYDHQGIFKR**N**NSRLMDEILKQQQELLGLDCSKYSPEFANSND

pan IASLVVWCMAVGISYISVYDHQGIFKRNNSRLMDEILKQQQELLGLDCSKYSPEFANSND

:**************:*****:*****************:***** *****: ****. *

gal KADQVLNCQSTLKVLSPEDGKADIVKAAQNFCQLVAQQQRTYTDLDVNVLDNLLSSTSGF

mus KDDQDLNCPSAVKVLSPEDGKADIVRAAQDFCQLVAQQQRKPTDLDVDLLGSLLS-SHGF

hum KDDQVLNCHLAVKVLSPEDGKADIVRAAQDFCQLVAQKQKRPTDLDVDTLASLLS-SNGC

pan KDDQVSNCHLAVKVLSPEDGKADIVRAAQDFCQLVAQKQKRPTDLDVDTLGSLLS-SNGC

* ** ** ::*************:***:*******:*: *****: * .*** : *

gal PDPDLVLKFGPVDSTLGFLPWHIRLTEIISLPSHQNISYEDFFSALHRYAACEQRWGK

mus PDPDLVLKFGPVDSTLGFLPWQIRLTEIVSLPSHLNISYEDFFSALRQYAACEQRLGK

hum PDPDLVLKFGPVDSTLGFLPWHIRLTEIVSLPSHL**N**ISYEDFFSALRQYAACEQRLGK

pan PDPDLVLKFGPVDSTLGFLPWHIRLTEIVSLPSHLNISYEDFFSALRQYAACEQRLGK

*********************:******:***** ***********::******* **

1. CD74

gal ---------------MAEEQRDLISSDGSSGVLPIGNSE-RSSLGRRTALSALSILVALL

mus ----------------MDDQRDLISNHEQLPILGNRPREPE-RCSRGALYTGVSVLVALL

hum MHRRRSRSCREDQKPVMDDQRDLISNNEQLPMLGRRPGAPESKCSRGALYTGFSILVTLL

pan MHRRRSRSCREDQKPVMDDQRDLISNNEQLPMLGRRPGAPESKCSRGALYTGFSILVTLL

::******.. . :* . .* : :..*:**:**

gal IAGQAVTIYYVYQQSGQISKLTKTSQTLKLESLQRKMPIGTQPANKMSMSTMNMPMAMKV

mus LAGQATTAYFLYQQQGRLDKLTITSQNLQLESLRMKLPKSAKPVSQMRMATP---LLMRP

hum LAGQATTAYFLYQQQGRLDKLTVTSQNLQLENLRMKLPKPPKPVSKMRMATP---LLMQA

pan LAGQATTAYFLYQQQGRLDKLTVTSQNLQLENLRMKLPKPPKPVSKMRMATP---LLMQA

:****.* *::***.*::.*** ***.*:**.*: *:* :*..:* *:* : *:

gal LPLAPSVGDMPMEAMKPRSNKTEDQIRHLLLKSDPRKTFPDLKDDMLGNLKRLKKTMSAM

mus MSMDN-MLLGPVKNVTKYGNMTQDHVMHLLTRSGPL-EYPQLKGTFPENLKHLKNSMDGV

hum LPMGA-LPQGPMQ**N**ATKYG**N**MTEDHVMHLLQNADPLKVYPPLKGSFPENLRHLKNTMETI

pan LPMGA-LPQGPMQNATKYGNMTEDHVMHLLQNADPLKVYPPLKGSFPENLRHLKNTMETI

: : : *:: . .* *:*:: *** .:.* :* **. : **::**::*. :

gal DWQDFETWMHKWLLFEMAKGPKMEEQNTIPAEKVQTKCQAEAS-FGGVHPGRFRPECDEN

mus NWKIFESWMKQWLLFEMSKNSLEEKKPTEAPPKVLTKCQEEVSHIPAVYPGAFRPKCDEN

hum DWKVFESWMHHWLLFEMSRHSLE-QKPTDAPPKVLTKCQEEVSHIPAVHPGSFRPKCDEN

pan DWKVFESWMHHWLLFEMSRHSLE-QKPTEAPPKVLTKCQEEVSHIPAVHPGSFRPKCDEN

:*: **:**::******:: :: * ** **** *.* : .*:** ***:****

gal GDYLPKQCYASTGYCWCCYKNGTRIEGTATRGQLDCSAPAPTQPPSAEPEEVIFSGVDMV

mus GNYLPLQCHGSTGYCWCVFPNGTEVPHTKSRGRHNCSEPLDMEDLSSG--------LGVT

hum GNYLPLQCYGSIGYCWCVFP**N**GTEVPNTRSRGHHNCSESLELEDPSSG--------LGVT

pan GNYLPLQCYGSIGYCWCVFPNGTEVPNTRSRGHHNCSESLELEDPSSG--------LGVT

*:*** **:.* ***** : ***.: * :**: :** : *: :.:.

gal KAK------

mus RQELGQVTL

hum KQDLGPVPM

pan KQDLGPVPM

: .

1. Lymphocyte antigen 6 complex

gal ----FFSAVAV--SRAQVQQEPSAETSEGTGINITCSHP-NILSSDYIHWYRHLPGRAPT

hum MAVLFLLLFLCGTPQA-ADNMQAIYVALGEAVELPCPSPPTLHGDEHLSWFCSPAAGSFT

pan MAVLFLLLFLCGTPQT-ADNMQAIYVALGEAVELPCPSPPTLHGDEHLSWFRSPAAGSFT

mus MAVVVFLLFLCGHSQAVADSIQTIYVASGESVEMPCPSPPSLLGGQLLTWFRSPVAGSST

.: . :: .:. : .: * .::: * * .: ..: : *: . : *

gal FLVSAVKGTKNVPDPAGQLSVSADR--RSSVLCLTRPGLADAAVYYCAVGRSRSSASNWK

hum TLVAQVQVGRPAPDPGKPGRESRLRLLG**N**YSLWLEGSKEEDAGRYWCAVLGQHHNYQNWR

pan TLVAQVQVGRPAPDPGKPGRESRLRLLGNYSLWLEGSKEEDAGRYWCAVLGQHHNYQNWR

mus ILVAQVQVDKPVSDLRKPEPDSRYKLFGNYSLWLEGSRDEDAGRYWCTVMDQNHKYQNWR

**: *: : . * * : . * * **. *:*:* .. . .**:

gal VTF---GSGTQLMVKPDITPSPSVYRLTSEDDKDLEMCLLTDYSPEKLDLSSVDSKTETV

hum VYDVLVLKGSQLSARAADGSPCNV-----------LLCSVV--PSRRMDSVTWQE-----

pan VYDVLVLKGSQLSARAADGSPCNV-----------LLCSVV--PSRRMDSVTWQE-----

mus VYDVSVLKGSQFSVKSPDGPSCAA-----------LLCSVV--PARRLDSVTWLE-----

* .*:*: .: . :* :. .::* : .

gal VEVATSENKHEASYLSTYWAKKDEMQCGAKHEGFGILKGDDPEA----------------

hum ------GKGPVRGRVQSFWGSEAALLLVCPGEGLSEPRSRRPRIIRCLMTHNKGVSFSLA

pan ------GKGPVRGRVQSFWGSEAALLLVCPGEGLSEPRSRRPRIIRCLMTHNKGVSFSLA

mus ------GRNTVRGHAQYFWGEGAALLLVCPTEGLPETRARRPRNIRCLLPQNKRFSFSLA

. . . :*.. : . **: :. *.

gal ----GASTVCITGMSLLFKTDENLNMLTFSQLGLKIIFMKAVIFNVLITMLMWKKNQ---

hum ASIDASPALCAPSTGW--------DM---PWILMLLLTMGQGVVILALSIVLWRQR-VRG

pan ASIDASPALCAPSTGW--------DM---PWILMLLLTMGQGVVILALSIVLWRQR-VRG

mus ASAEPSPTVCATLPSW--------DV---PWILVLLFTAGQGVTIIALSIVLWRRRRAQG

: ::* . :: : : :: : : :::::*::.

gal ----------------------------------

hum APGRDASIPQFKPEIQVYENIHLARLGPPAHKPR

pan APGRDASIPQFKPEIQVYENIHLARLGPPAHKPR

mus SRDREPSVPHFKPEVQVYENIHLARLSPPNHKTR

1. Mimecan

gal MKTLQAAFFLVAFVPLVKPAPPIQQDSPKFYEYVDADFATGSLIQQDYEML--PKDTIKD

mus METVHSTFLLLLFVPLTQQAPQSQLDSHVNYEYATGNSEETK-FSQDYEDKYLDGKSIKE

hum MKTLQSTLLLLLLVPLIKPAPPTQQDSRIIYDYGTDNFEESI-FSQDYEDKYLDGKNIKE

pan MKTLQSTLLLLLFVPLIKPAPPTQQDSRIIYDYGTDNFEESI-FSQDYEDKYLDGKNIKE

*:*::::::*: :*** : ** * ** *:* : :.**** ..**:

gal GTNVSL--DTALRLQADDSELSAR-PTKDTNLPTCLLCVCLSGSVYCEEIDIEAVPPLPK

mus KETMIIPDEKSLQLQKDEVIPSLPTKKENDEMPTCLLCVCLSGSVYCEEVDIDAVPPLPK

hum KETVIIPNEKSLQLQKDEAITPLPPKKENDEMPTCLLCVCLSGSVYCEEVDIDAVPPLPK

pan KETVIIPNEKSLQLQKDEAITPLPPKKENDEMPTCLLCVCLSGSVYCEEVDIDAVPPLPK

.: : :.:*:** *: .:: ::*****************:**:*******

gal ETAYLYARFNKIKRIAVSDFADITTLRRIDFSGNMIEEIEDGAFSKLLLLEELSLAENRL

mus ESAYLYARFNKIKKLTAKDFADMPNLRRLDFTGNLIEDIEDGTFSKLSLLEELTLAENQL

hum ESAYLYARFNKIKKLTAKDFADIPNLRRLDFTGNLIEDIEDGTFSKLSLLEELSLAENQL

pan ESAYLYARFNKIKKLTAKDFADIPNLRRLDFTGNLIEDIEDGTFSKLSLLEELSLAENQL

*:***********:::..****: .***:**:**:**:****:**** *****:****:*

gal VKLPVLPPKLTTFNANQNRIKSRGIKNNAFKKLTNLAYLYLGHNALESVPLNLPESLRIL

mus LRLPVLPPKLTLLNAKHNKIKSKGIKANTFKKLNKLSFLYLDHNDLESVPPNLPESLRVI

hum LKLPVLPPKLTLFNAKYNKIKSRGIKANAFKKLN**N**LTFLYLDHNALESVPLNLPESLRVI

pan LKLPVLPPKLTLFNAKYNKIKSRGIKANAFKKLNNLTFLYLDHNALESVPLNLPESLRVI

::********* :**: *:***:*** *:****.:*::***.** ***** *******::

gal HLQHNNITTINDDTFCKSNNTRYIRTRMDEIRMEGNPILLAKHVNAFSCLRTLPVGTYY

mus HLQFNSISSLTDDTFCKANDTRYIRERIEEIRLEGNPIALGKHPNSFICLKRLPIGSYF

hum HLQFNNIASITDDTFCKA**N**DTSYIRDRIEEIRLEGNPIVLGKHPNSFICLKRLPIGSYF

pan HLQFNNIASITDDTFCKANDTSYIRDRIEEIRLEGNPIVLGKHPNSFICLKRLPIGSYF

***.*.*:::.******:*:* *** *::***:***** *.** *:* **: **:*:*:

1. Zn alpha-2 glycoprotein

gal ---MGPSEAVVLGLLLG-----AAECGSHSLRYFMTGMTDPGPGMPQFVIVGYVDGELFG

hum MVRMVPVL-LSLLLLLGPAVPQENQDGRYSLTYIYTGLSKHVEDVPAFQALGSLNDLQFF

pan MVRMVPVL-LSLLLLLGPAVPQENQDGRYSLTYIYTGLSKHVEDVPAFQALGSLNDLQFF

mus ---MVPVL-LSLPLLLGPAVFQE--TGSYSLTFLYTGLSRPSKGFPRFQATAFLNDQAFF

* * : * **** * :** :: **:: ..* * . ::. *

gal KYDSKSRWVHPIVEKLPQEDQEHWDTQTLKAREGELEFSEGLHRLQVRYNRSGGSHTLQK

hum RYNSKDRKSQPMGLWRQVEGMEDWKQDSQLQKAREDIFMETLKDIVEYYNDSNGSHVLQG

pan RYNSKDRKSQPMGLWRQVEGMEDWKQDSQLQKAREDIFMETLKDIVEYY**N**DS**N**GSHVLQG

mus HYNSNSGKAEPVGPWSQVEGMEDWEKESQLQRAREEIFLVTLKDIMDYYKDTTGSHTFQG

:*:*:. .*: *. *.*. :: : * * *: : *: : ***.:*

gal MFGCDILEDGSIRGYDQYAFDGRDYIAFDMDTMTFTAADPVAEITKRRWETEGTYAERWK

hum RFGCEIENNRSSGAFWKYYYDGKDYIEFNKEIPAWVPFDPAAQITKQKWEAEPVYVQRAK

pan RFGCEIEN**N**RSSGAFWKYYYDGKDYIEFNKEIPAWVPFDPAAQITKQKWEAEPVYVQRAK

mus MFGCEITNNRSSGAVWRYAYDGEDFIEFNKEIPAWIPLDPAAANTKLKWEAEKVYVQRAK

***:* :: * . :* :**.*:* *: : :: **.* ** :**:* .*.:* *

gal HELGTVCVQNLRRYLEHGKAALKRRVLPEVRVWGKEANG-ILTLFCRAYGFYPWPISLSW

hum AYLEEECPATLRKYLKYSKNILDRQDPPSVVVTSHQAPGEKKKLKCLAYDFYPGKIDVHW

pan AYLEEECPATLRKYLKYSKNILDRQDPPSVVVTSHQAPGEKKKLKCLAYDFYPGKIDVHW

mus AYLEEECPEMLKRYLNYSRSHLDRIDPPTVTITSRVIPGGNRIFKCLAYGFYPQRISLHW

* * *::**::.: *.* * * : .: * : * **.*** *.: *

gal MKDGMVRDQETHWGGVVPNSDGTYHASAAIDVPPEDGDKYRCHVEHASLPQPGLFLWEPQ

hum TRAGEVQEPEL-RGDVLHNG**N**GTYQSWVVVAVPPQDTAPYSCHVQHSSLAQPLVVPWEAS

pan TRAGEVQEPEL-RGDVLHNGNGTYQSWVVVAVPPQDTAPYSCHVQHSSLAQPLVVPWEAR

mus NKANKKLAFEP-ERGVFPNGNGTYLSWAEVEVSPQDIDPFFCLIDHRGFSQSLSVQWDRT

: . * .*. *.:*** : . : * *:* : * ::* .: * . *:

gal PNLIPSGAGAVIAIVAVIAAVVGLVVWKSKSDKGRELGGRKKGLRSSTRPRWGIQRLGHR

hum ------------------------------------------------------------

pan ------------------------------------------------------------

mus RKVKDENNVVAQPQ----------------------------------------------

gal K

hum -

pan -

mus -

1. Ficolin

mus ------------------------------------------------------MQ----

gal MGSCRSRRKLCRHRQGTQAVGKRRGGAYMGFFGQCKSLRQLSANHNAWSTGKGSREWSTV

hum ------------------------------------------------------------

pan ------------------------------------------------------------

mus ------WPTLWAFSGLLCLCPSQALGQERGACPDVKVVGLGAQDKVVVIQSCPGFPGPPG

gal LAMGRAAPILLALLS--ASATTCST---QDTCPEVKIVGLGEADRLAVLQGCPGIPGAAG

hum MDLLWILPSLWLLL---LGGPACLKTQEHPSCPGPR---ELEASKVVLLPSCPGAPGSPG

pan ------------------------------------------------------------

mus PKGEPGSPAGRGERGFQGSPGKMGPAGSKGEPGTMGPPGVKGEKGDTGAAPSLGEKELGD

gal PKGEPGLSGKKGEMGTKGPPGKAGPPGIKGAAGDPGLPGPKGAKGEPGFPE---------

hum EKGAPGPQG------------PPGPPG------------KMGPKGEPGDPVN-------L

pan ------------------------------------------------------------

mus TLCQRGPRSCKDLLTRGIFLTGWYTIHLPDCRPLTVLCDMDVDGGGWTVFQRRVDGSIDF

gal ----PLARNCQELLDKGKILSGWYTIHPQGCNATTVFCDMDTDGGGWIVFQRRLDGSVNF

hum LRCQEGPRNCRELLSQGATLSGWYHLCLPEGRALPVFCDMDTEGGGWLVFQRRQDGSVDF

pan ---------------------------------------MDTEGGGWLVFQRRQDGSVDF

**.:**** ***** ***::*

mus FRDWDSYKRGFGNLGTEFWLGNDYLHLLTANGNQELRVDLQDFQGKGSYAKYSSFQVSEE

gal LRDWNSYKRGFGNQLTEFWLGNDNLHFLTSLGTCELRVDLRDFDNNYYFAKYASFRVLGE

hum FRSWSSYRAGFGNQESEFWLGNENLHQLTLQGNWELRVELEDFNG**N**RTFAHYATFRLLGE

pan FRSWSSYRAGFGNQESEFWLGNENLHQLTLQGNWELRVELEDFNGNCTFAHYATFRLLGE

:*.*.**: **** :******: ** ** *. ****:*.**:.: :*:*::*:: *

mus QEKYKLTLGQFLEGTAGDSLTKHNNMSFTTHDQDNDANSMNCAALFHGAWWYHNCHQSNL

gal SEKYKLVLGDFLGGNAGDSLSYHKDMSFSTADQDNDMSSFNCATAYKGAWWYNDCHYSNL

hum VDHYQLALGKFSEGTAGDSLSLHSGRPFTTYDADHDSSNSNCAVIVHGAWWYASCYRSNL

pan VDHYQLALGKFSEGTAGDSLSHHSGRPFTTYDADHDSSNSNCAVIVHGAWWYASCYRSNL

::*:*.**.* *.*****: *.. *:* * *:* .. ***. :***** .*: ***

mus NGRYLSGSHESYADGINWGTGQGHHYSYKVAEMKIRAS

gal NGMYWLGAHGSYADGINWKTGKEYHYSHKRTEMKFRPI

hum NGRYAVSEAAAHKYGIDWASGRGVGHPYRRVRMMLR--

pan NGRYAVSEAAAHKYGIDWASGRGVGHPYRRVQMMLR--

** * . :: **:* :*: : :: ..* :*

1. Junctional adhesion molecule 1

gal MAGGVRRGGGGARPL-------VLLCAAAVSLGGAQVTSETKEVPENQPVDIPCSAYRSS

hum ----MGTKAQVERKLLCLFILAILLCSLALGSVTVHSSEPEVRIPENNPVKLSCAY--SG

pan ----MGTKAQVERKLLCLFILAILLCSLALGSVTVHSSEPEVRIPENNPVKLSCAY--SG

mus ----MGTEGKAGRKLLFLFTS-MILGSLVQGKGSVYTAQSDVQVPENESIKLTCTY--SG

: . * * ::* : . . . :. .:***: :.: *: *.

gal WTDIRIEWKFQKGSSLGLFYYGGELTDPYKNRVRFSVTSIHLTAVTREDTGKYICKVVG-

hum FSSPRVEWKFDQGDTTRLVCYNNKITASYEDRVTFLPTGITFKSVTREDTGTYTCMVSEE

pan FSSPRVEWKFDQGDTTRLVCYNNKITASYEDRVTFLPTGITFKSVTREDTGTYTCMVSEE

mus FSSPRVEWKFVQGSTTALVCYNSQITAPYADRVTFSSSGITFSSVTRKDNGEYTCMVSEE

::. *:**** :*.: *. *..::* * :** * :.* :.:***:*.* * * *

gal GGSQIAKSEVNLIVQVPPSKPTAHVPSSATIGRTAVLRCSESEGSPPPTFRWYRDSMLIP

hum GGNSYGEVKVKLIVLVPPSKPTVNIPSSATIGNRAVLTCSEQDGSPPSEYTWFKDGIVMP

pan GGNSYGEVKVKLIVLVPPSKPTVNIPSSATIGNRAVLTCSEQDGSPPSEYTWFKDGIVMP

mus GGQNYGEVSIHLTVLVPPSKPTISVPSSVTIGNRAVLTCSEHDGSPPSEYSWFKDGISML

**.. .: .::* * ******* :***.***. *** *** :**** : *::*.: :

gal A-DPRSSLSFRNSSYTLDSTTGELTFNPVSAFDTGDYYCEASNNVGTAQRSDTVRMEASE

hum T-NPKSTRAFS**N**SSYVL**N**PTTGELVFDPLSASDTGEYSCEARNGYGTPMTSNAVRMEAVE

pan T-NPKSTRAFSNSSYVLNPTTGELVFDPLSASDTGEYSCEARNGYGTPMTSNAVRMEAVE

mus TADAKKTRAFMNSSFTIDPKSGDLIFDPVTAFDSGEYYCQAQNGYGTAMRSEAAHMDAVE

: : :.: :* ***:.:: .:*:* *:*::* *:*:* *:* *. ** *::.:*:* *

gal LNVGGVVAAVVVLLTLLGLIAFGLWFAYSRGHFQRKDT---ASKKVIYSQPSQRSDGDFK

hum RNVGVIVAAVLVTLILLGILVFGIWFAYSRGHFDRTKKGTS-SKKVIYSQPSARSEGEFK

pan RNVGVIVAAVLVTLILLGILVFGIWFAYSRGHFDRTKKGTS-SKKVIYSQPSARSEGEFK

mus LNVGGIVAAVLVTLILLGLLIFGVWFAYSRGYFERTKKGTAPGKKVIYSQPSTRSEGEFK

*** :****:* * ***:: **:*******:*:*... .********* **:*:**

gal QTSSFLV

hum QTSSFLV

pan QTSSFLV

mus QTSSFLV

*******

1. ADP ribosyl cyclase

gal --------MPSQRGSARRRQRCVLLAGIAILLIALVLAAVLASVLTGSKEEANPEPLRWQ

mus MANYEFSQVSGDRPGCRLSRKAQIGLGVGLLVLIALVVGIVVILL------RPRSLLVWT

hum MANCEFSPVSGDKPCCRLSRRAQLCLGVSILVLILVVV---LAVV------VPRWRQQWS

pan MANCEFSPVSGDKPCCRLSRRAQLCLGVSILVLILVVV---LAVV------VPRWRQQWS

: .:: .* ::. : *:.:*:: ::. :: *

gal GRGTTRNLREIVLGRCYSYLAEGHAELRDKDCLKIWESLKAAFVHKNPCNATTEDYRPLM

mus GEPTTKHFSDIFLGRCLIYTQILRPEMRDQNCQEILSTFKGAFVSKNPCNITREDYAPLV

hum GPGTTKRFPETVLARCVKYTEI-HPEMRHVDCQSVWDAFKGAFISKHPC**N**ITEEDYQPLM

pan GPGTTKRFPETVLARCVKYTEI-HPEMRHVDCQSVWDAFKGAFISKHPCNITEEDYQPLM

* **:.: : .*.** * : *:*. :* .: .::*.**: *:*** * *** **:

gal ELTSHPVPCNESLFWSRTYDLVHLYSKCNHHFLTLEDTFLGYMLDGLSWCGDPSAPGINY

mus KLVTQTIPCNKTLFWSKSKHLAHQYTWIQGKMFTLEDTLLGYIADDLRWCGDPSTSDMNY

hum KLGTQTVPCNKILLWSRIKDLAHQFTQVQRDMFTLEDTLLGYLADDLTWCGEF**N**TSKINY

pan KLGTQTVPCNKILLWSRIKDLAHQFTQVQQDMFTLEDTLLGYLADDLTWCGEFSTSEINY

:* :: :***: *:**: .*.* :: : .::*****:***: *.* ***: .: :**

gal ESCPKWSE-CERSSGSVFWKMASKMFAEAACGVVHVMLNGSIEAGAFRTSSVFGSVEIFN

mus VSCPHWSENCPNNPITVFWKVISQKFAEDACGVVQVMLNGSLRE-PFYKNSTFGSVEVFS

hum QSCPDWRKDCSNNPVSVFWKTVSRRFAEAACDVVHVML**N**GSRSK-IFDK**N**STFGSVEVHN

pan QSCPDWRKDCSNNPVSVFWKTVSRRFAEAACDVVHVMLNGSRSK-IFDKNSTFGSVEVHN

***.* : * .. :**** *: *** **.**:****** * ..*.*****:..

gal LNPDKVSEIRIWLMNDIGGPQSDSCSGYSIKKLRNILEERNFKITCEDNYRPVQLLQCVH

mus LDPNKVHKLQAWVMHDIEGASSNACSSSSLNELKMIVQKRNMIFACVDNYRPARFLQCVK

hum LQPEKVQTLEAWVIHGGREDSRDLCQDPTIKELESIISKRNIQFSCKNIYRPDKFLQCVK

pan LQPEKVQTLEAWVIHGGREDSRDLCQDPTIKELESIISKRNIQFSCKNIYRPDKFLQCVK

*:*:** :. *:::. . : *.. ::::*. *:.:**: ::* : *** ::****:

gal NPDHPDCNLCISSTEAP

mus NPEHPSCRLNT------

hum NPEDSSCTSEI------

pan NPEDSSCTSEI------

**:. .*

1. Sulfatase modifying factor 2

gal ------------------------MRRAAVLCCCVLGCSCALLAFGSNEHMVRLPGGRFQ

mus ------------MRS-EFWFPSMGSLLPPVLLLWLLSCPRLQLGHAQDPAMVHLPGGRFL

hum -------------------MAR--HGLPLLPLLSLLVGAWLKLGNGQATSMVQLQGGRFL

pan MRAHAQRGRGCTRRSAAALMAR--HGLPLLPLLSLLVGAWLKLGNGQATSMVQLQGGRFL

: :* *. .. **:* ****

gal MGSSSTQSRDEEGPIREVTVKPFAIDKFPVTNRDFREFVREKKYKTEAEAFGWSFVFEDF

mus MGTDAPDGRDGEGPAREVTVKPFAIDIFPVTNKDFREFVREKKYQTEAEAFGWSFVFEDF

hum MGTNSPDSRDGDGPVREATVKPFAIDIFPVTNKDFRDFVREKKYRTEAEMFGWSFVFEDF

pan MGTNSPDSRDGEGPVREATVKPFAIDIFPVTNKDFRDFVREKKYRTEAEIFGWSFVFEDF

**:.: :.** :** **.******** *****:***:*******:**** **********

gal VSEELKKKITQKLESAPWWLPVEKAFWRQPAGPGSGIADRLDHPVLHVSWNDAQAFCRWK

mus VSPELRKQ-ENLMPAVHWWQPVPKAFWRQPAGPGSGIREKLELPVVHVSWNDAGAYCAWR

hum VSDELRNKATQPMKSVLWWLPVEKAFWRQPAGPGSGIRERLEHPVLHVSWNDARAYCAWR

pan VSDELRNKATQPMKSVLWWLPVERAFWRQPAGPGSGILERLEHPVLHVSWNDARAYCAWR

** **::: : : :. ** ** :************* ::*: **:******* *:* *:

gal GKRLPTEEEWEFAARGGLEQRLYPWGNKFQPNRTNLWQGDFPRGDTAEDGYHGVSPVAAF

mus GRRLPTEEEWEFAARGGLKGQVYPWGNRFQPNRTNLWQGKFPKGDKAEDGFHGLSPVNAF

hum GKRLPTEEEWEFAARGGLKGQVYPWGNWFQPNRTNLWQGKFPKGDKAEDGFHGVSPVNAF

pan GKRLPTEEEWEFAARGGLKGQVYPWGNWFQP**N**RTNLWQGKFPKGDKAEDGFHGVSPVNAF

*:****************: ::***** ***********.**:**.****:**:*** **

gal SPQNSYGLYDLLGNTWEWTASQYTPPGPPRPRAEAMHVLRGASWIDTVDGSANHRASITT

mus PPQNNYGLYDLMGNVWEWTASTYQPA------GQDMRVLRGASWIDTADGSANHRARVTT

hum PAQNNYGLYDLLGNVWEWTASPYQAA------EQDMRVLRGASWIDTADGSANHRARVTT

pan PAQNNYGLYDLLGNVWEWTASPYQAA------EQDMRVLRGASWIDTADGSANHRARVTT

**.******:**.****** * : *:**********.******** :**

gal RMGNTPDSASDNLSFRCAADIPNRTTKSSRTKPEL

mus RMGNTPDSASDNLGFRCASSAGRPKEDL-------

hum RMGNTPDSASDNLGFRCAADAGRPPGEL-------

pan RMGNTPDSASDNLGFRCAADAGRPPGEL-------

*************.****:. . .

1. DNAse 1 like

gal --------------------MLLFALFSLLCFNPSLSLRICSFNVRSFGEAKIGRAEVLD

mus MPFGQPGFLWRVPDAHIAMRGLVMAPLLILLVGGTEAFRICAFNAHRLTLAKLTKESVMD

hum -------------------MHYPTALLFLILANGAQAFRICAFNAQRLTLAKVAREQVMD

pan -------------------MHYPTALLFLILANGAQAFRICAFNAQRLTLAKVAREQVMD

* : :: . : ::***:**.: : **: : .*:*

gal AVVKIISRCDIMLLMEIKENKNRMCPLLVQQLAGQTKGNREEYSCVVSGRLGRKSYKEQY

mus TLVQILARCDIMVLQEVVDSSQNTVPFLLQKL---K--SSRSYSFLNSSLLGRSTYKEKY

hum TLVRILARCDIMVLQEVVDSSGSAIPLLLRELNRFD--GSGPYSTLSSPQLGRSTYMETY

pan TLVRILARCDIMVLQEVVDSSGSAIPLLLRELNRFD--GSGPYSTLSSPQLGRSTYMETY

::*:*::*****:* *: :.. *:*:::* . ** : * ***.:* * *

gal AFLYRRNLVSVKQTYQYPDTQPGDEDAFSREPFVVWFRSPKTAVKEFAIVPLHTAPEMAV

mus VYIYRSDKTQVLNFYQYNDT----DDIFAREPFVAHFTLPSKTLPSVVLVPLHTTPKDVE

hum VYFYRSHKTQVLSSYVYNDE----DDVFAREPFVAQFSLPSNVLPSLVLVPLHTTPKAVE

pan VYFYRSHKTQVLSSYVYNDE----DDVFAREPFVAQFSLPSNVLPSLVLVPLHTTPKAVE

.::** . ..* . * * * :* *:*****. * *...: ...:*****:*: .

gal REIDELYDVYLDIKQHWDTENFVFMGDFNAGCSYVPRKHWKNIRLRTSSEFAWLIGDTND

mus KELNALYDVFLDVYQRWQNENVILLGDFNADCASLTKKRLKSLLLRTKAGFHWVIPDGED

hum KELNALYDVFLEVSQHWQSKDVILLGDFNADCASLTKKRLDKLELRTEPGFHWVIADGED

pan KELNALYDVFLEVSQHWQSKDVILLGDFNADCASLTKKRLDKLELRTEPGFHWVIADGED

:*:: ****:*:: *:*:.::.:::*****.*: : :*: ..: ***. * *:* * :*

gal TTVRRSTSCPYDRIVVSGEQLSQAVVPHSATIFDFQTAFQMSEEQALGVSDHFPVEFELK

mus TTVRASTNCTYDRIVVHGQGCQ--MLLKAAATFDFPKRFQLTEEEALRISDHYPVEVELS

hum TTVRASTHCTYDRVVLHGERCR--SLLHTAAAFDFPTSFQLTEEEALNISDHYPVEVELK

pan TTVRASTHCTYDRIVLHGERCR--SLLHTAAAFDFPTSFQLTEEEAL**N**ISDHYPVEVELK

**** ** * ***:*: *: : ::*: *** . **::**:** :***:***.**.

gal ARGGFFD------------WIKSKFSKKGRARKSRRSGS

mus QATPLSIPPHYLAALL-LSLLPSQLD-------------

hum LSQAHSVQPLSLTVLLLLSLLSPQLCPAA----------

pan LSQAHSVQPLSLTVLLLLSLLSPQLCPAA----------

: ::

1. ICOS ligand

gal -------------------------MKRLGYGFLLLFLHILRAVTALEKIISKPGDNATL

hum -------------------------MRLGSPGLLFLLFSSLRADTQEKEVRAMVGSDVEL

pan -------------------------MRLGSPGLLFLLFSSLRADTQEKEVRAMVGSDVEL

mus MQLKCPCFVSLGTRQPVWKKLHVSSGFFSGLGLFLLLLSSLCAASAETEVGAMVGSNVVL

. *:::*:: * * : :: : *.:. *

gal SCIYAN-RGFDLDSLRVYWQIDGVEGSKSCSVVHALISGQDNESQQCSQFKNRTQLLWDK

hum SCACPEGSRFDLNDVYVYWQTSESKTVVTY-----HIPQ**N**SSLENVDSRYRNRALMSPAG

pan SCTCPEGSRFDLNDVYVYWQTSESKTVVTY-----HIPQNSSLENVDSRYRNRALMSPAG

mus SCIDPHRRHFNLSGLYVYWQIENPEVSVTY-----YLPYKSPGINVDSSYKNRGHLSLDS

** . *:*..: **** . : : : :. : * ::** :

gal LGDGDFSLLLYNVRQSDEHTYKCVVMQTI-EYTRVIHQEQVVLSLAASYSQPILSGPIRN

hum MLRGDFSLRLFNVTPQDEQKFHCLVLSQS-LGFQEVLSVEVTLHVAANFSVPVVSAPH--

pan MRRGDFSLRLF**N**VTPQDEQKFHCLVLSQS-LGFQEVLSIEVTLHVAA**N**FSVPVVSTPH--

mus MKQGNFSLYLKNVTPQDTQEFTCRVFMNTATELVKILEEVVRLRVAANFSTPVISTSD--

: *:*** * ** .* : : * *: : . * * :**.:* *::*

gal SYSTGEEVTFSCRSDNGYPEPNVYWINRTDNTRLSQSDFN--ITQHPDGTYSVLSTLKVN

hum SP-SQDELTFTCTSINGYPRPNVYWI**N**KTDNSLLDQALQ**N**DTVFLNMRGLYDVVSVLRIA

pan SP-SQDELTFTCTSINGYPRPNVYWINKTDNSLLDQALQNDTIFLNTRGLYDVVSVLRIA

mus SSNPGQERTYTCMSKNGYPEPNLYWINTTDNSLIDTALQNNTVYLNKLGLYDVISTLRLP

* :* *::* * ****.**:**** ***: :. : * : : * *.*:*.*::

gal ATSDMQLECFIENKVLQENTSANYTEEMQNNGSSTGSHKDAAKGGQGAQAAAVVSVVILM

hum RTPSVNIGCCIENVLLQQ**N**LTVGSQTGNDIGERDKITENPVSTGEKNAATWSILAVLCLL

pan RTPSVNIGCCIENVLLQQNLTVGSQTGNNIGERDKITENPVSTGEKNAATWSILAVLCLL

mus WTSRGDVLCCVENVALHQNITSISQAESFTGNNTKN---PQETHNNE--LKVLVPVLAVL

* :: * :** *::* : . . . : :: *: ::

gal AFLTVLICWLWRRRSFQLVSYTAPV------------

hum VVVAVAIGWV-CRDRCLQHSYAGAWAVSPETELTGHV

pan VVVAVAIGWV-CRDRCLQHSYAGAWAVSPETELTGHV

mus AAAA-FVSFIIYRRTRPHRSYTGPKTVQL--ELTDHA

. : : :: * **:.

1. Lysosomal thioesterase

gal MAALGLAVPALLW-------LGLGLGL-GSGRAMAAVPLVIWHGMGDSCCNPLSMGYVRD

mus MPGLWRQRLPSAWALLLLPFLPLLMPAAPAAHRGSYKPVIVVHGLFDSSYSF---RHLLD

hum MLGLCGQRLPAAWVLLLLPFLPLLLLAAPAPHRASYKPVIVVHGLFDSSYSF---RHLLE

pan MLGLWGQRLPAAWVLLLLPFLPLLLLAAPGPHRASYKPVIVVHGLFDSSYSF---RHLLE

* .* * * * : . : : *::: **: **. . :: :

gal LVQRRIPGIYVL--SLRIGSSLVQDVENSFFMNVNDQVREVCSQLAKDPHLKGGYNAMGF

mus YINETHTGTVVTVLDLFDGRESL----RPLWEQVQGFREAVVPIM---EKAPEGVHLICY

hum YI**N**ETHPGTVVTVLDLFDGRESL----RPLWEQVQGFREAVVPIM---AKAPQGVHLICY

pan YINETHPGTVVTVLDLFDGRESL----RPLWEQVQGFREAVVPIM---AKAPQGLHLICY

::. * * .* * . : . :: :*:. . * : : * : : :

gal SQGGQFLRAVAQRCPSPPMFTLISIGGQHQGVYGFPRCPGESSQICDWIRKML-DLGAY-

mus SQGGLVCRALLSVMDNHNVDSFISLSSPQMGQYGDTD-------YLKWLFPTSMRSNLYR

hum SQGGLVCRALLSVMDDHNVDSFISLSSPQMGQYGDTD-------YLKWLFPTSMRSNLYR

pan SQGGLVCRALLSVMDDHNVDSFISLSSPQMGQYGDTD-------YLKWLFPTSMRSNLYR

**** . **: . . : ::**:.. : * ** .*: . *

gal --TQAVQEHLVQAQYWHDPLKEEDYRKKSIFLADINQERGI--NETYKKNLMALKKFVMV

mus VCYSPWGQEFSICNYWHDPHHDDLYLNASSFLALINGERDHPNATAWRKNFLRVGRLVLI

hum ICYSPWGQEFSICNYWHDPHHDDLYL**N**ASSFLALINGERDHP**N**ATVWRKNFLRVGHLVLI

pan ICYSPWGQEFSICNYWHDPHHDDLYLNASSFLALINGERDHPNATVWRKNFLRVGHLVLI

. :.: .:***** ::: * : * *** ** **. .::**:: : ::*::

gal KFLNDTMVDPPISEWFGFYKSGQAKDTIPLKETLLYKEDRLGLQQMDKAGKLVFLGVEG-

mus GGPDDGVITPWQSSFFGFYDANET--VLEMEEQPVYLRDSFGLKTLLARGAIVRCPMAGI

hum GGPDDGVITPWQSSFFGFYDA**N**ET--VLEMEEQLVYLRDSFGLKTLLARGAIVRCPMAGI

pan GGPDDGVITPWQSSFFGFYDANET--VLEMEEQLVYLRDSFGLKTLLARGAIVRCPMAGI

:* :: * *.:****.:.:: .: ::* :* .* :**: : * :* : *

gal --DHLHFTEEWFYTHILPFLQ

mus SHTTWHSNRTLYDTCIEPWLS

hum SHTAWHS**N**RTLYETCIEPWLS

pan SHTAWHSNRTLYETCIEPWLS

* .. : * * *:*.

1. Sodium/potassium-transporting ATPase subunit beta-1

gal MARGKAKDGDGNWKKFIWNSEKKELLGRTGGSWFKILLFYVIFYGCLAGIFIGTIQVMLL

mus MARGKAK-EEGSWKKFIWNSEKKEFLGRTGGSWFKILLFYVIFYGCLAGIFIGTIQVMLL

hum MARGKAK-EEGSWKKFIWNSEKKEFLGRTGGSWFKILLFYVIFYGCLAGIFIGTIQVMLL

pan MARGKAK-EEGSWKKFIWNSEKKEFLGRTGGSWFKILLFYVIFYGCLAGIFIGTIQVMLL

******* :*.************:***********************************

gal TVSEFEPKYQDRVAPPGLTQVPQVQKTEISFTVNDPKSYDPYVKNLEGFLNKYSAGEQTD

mus TISELKPTYQDRVAPPGLTQIPQIQKTEISFRPNDPKSYEAYVLNIIRFLEKYKDSAQKD

hum TISEFKPTYQDRVAPPGLTQIPQIQKTEISFRPNDPKSYEAYVLNIVRFLEKYKDSAQRD

pan TISEFKPTYQDRVAPPGLTQIPQIQKTEISFRPNDPKSYEAYVLNIVRFLEKYKDSAQRD

*:**::*.************:**:******* ******: ** *: **:**. . * *

gal NIVFQDCGDIPTDYKERGPYNDAQGQKKVCKFKREWLENCSGLQDNTFGYKDGKPCILVK

mus DMIFEDCGNVPSEPKERGDINHERGERKVCRFKLDWLGNCSGLNDDSYGYREGKPCIIIK

hum DMIFEDCGDVPSEPKERGDFNHERGERKVCRFKLEWLG**N**CSGLNDETYGYKEGKPCIIIK

pan DMIFEDCGDVPSEPKERGDFNHERGERKVCRFKLEWLGNCSGLNDETYGYKEGKPCIIIK

:::*:***::*:: **** *. :*::***:** :** *****:*:::**::*****::*

gal LNRIIGFKPKAPENESLPS-DLAGKYNPYLIPVHCVAKRDEDADKIGMVEYYGMGGYPGF

mus LNRVLGFKPKPPKNESLETYPLMMKYNPNVLPVQCTGKRDEDKDKVGNIEYFGMGGYYGF

hum LNRVLGFKPKPPK**N**ESLETYPV-MKYNPNVLPVQCTGKRDEDKDKVGNVEYFGLGNSPGF

pan LNRVLGFKPKPPKNESLETYPV-MKYNPNVLPVQCTGKRDEDKDKIGNVEYFGLGNSPGF

***::***** *:**** : : **** ::**:*..***** **:* :**:*:*. **

gal ALQYYPYYGRLLQPQYLQPLVAVQFTNLTYDVEVRVECKAYGQNIQYSDKDRFQGRFDIK

mus PLQYYPYYGKLLQPKYLQPLLAVQFTNLTVDTEIRVECKAYGENIGYSEKDRFQGRFDVK

hum PLQYYPYYGKLLQPKYLQPLLAVQFT**N**LTMDTEIRIECKAYGENIGYSEKDRFQGRFDVK

pan PLQYYPYYGKLLQPKYLQPLLAVQFTNLTMDTEIRIECKAYGENIGYSEKDRFQGRFDVK

********:****:*****:******** *.*:*:******:** **:*********:*

gal FDIKSS

mus IEIKS-

hum IEVKS-

pan IEVKS-

:::**

1. Cathepsin Z

gal MLSMAGPVRRLAALLLVLCGCLYPCRAGLYVREGQHCYKPAPRR---APGLRTYPRPHEY

hum -MARRGP--GWRPLLLLV-LLAGAAQGGLYFRRGQTCYRPLRGDGLAPLGRSTYPRPHEY

pan -MARRGP--GWRPLLLLV-LLAGAAQGGLYFRRGQTCYRPLRGDGLAPLGRSTYPRPHEY

mus -MASSGSVQQLPLVLLML-LLASAARARLYFRSGQTCYHPIRGDQLALLGRRTYPRPHEY

:: * :**:: .:. **.* ** **:* * ********

gal LDMAELPQSWDWRNVNGVNYASTTRNQHIPQYCGSCWAHGSTSALADRINIKRKGAWPSA

hum LSPADLPKSWDWRNVDGVNYASITRNQHIPQYCGSCWAHASTSAMADRINIKRKGAWPST

pan LSPADLPKSWDWRNVDGVNYASITRNQHIPQYCGSCWAHASTSAMADRINIKRKGAWPST

mus LSPADLPKNWDWRNVNGVNYASVTRNQHIPQYCGSCWAHGSTSAMADRINIKRKGAWPSI

*. *:**:.******:****** ****************.****:**************

gal YLSVQNVIDCANAGSCEGGDHTGVWMYAHDHGIPDETCNNYQAKNQKCKKFNQCGTCVTF

hum LLSVQNVIDCGNAGSCEGGNDLSVWDYAHQHGIPDETCNNYQAKDQECDKFNQCGTCNEF

pan LLSVQNVIDCGNAGSCEGGNDLSVWDYAHQHGIPDETCNNYQAKDQECDKFNQCGTCNEF

mus LLSVQNVIDCGNAGSCEGGNDLPVWEYAHKHGIPDETCNNYQAKDQDCDKFNQCGTCTEF

*********.********:. ** ***.**************:*.*.******** *

gal GECHVIKNYTLWKVADYGAVSGREKMMAEIYANGPISCGIMATEKLDAYTGGLYTEYNPS

hum KECHAIR**N**YTLWRVGDYGSLSGREKMMAEIYANGPISCGIMATERLA**N**YTGGIYAEYQDT

pan KECHAIRNYTLWRVGDYGSLSGREKMMAEIYANGPISCGIMATERLANYTGGIYAEYQDT

mus KECHTIQNYTLWRVGDYGSLSGREKMMAEIYANGPISCGIMATEMMSNYTGGIYAEHQDQ

***.*:*****:*.***::************************ : ****:*:*::

gal PTVNHIVSVAGWGVE-NGTEYWIVRNSWGEPWGERGWLRIVTSAYKGGRGAEYNLAVEED

hum TYINHVVSVAGWGIS-DGTEYWIVRNSWGEPWGERGWLRIVTSTYKDGKGARYNLAIEEH

pan TYINHVVSVAGWGIS-DGTEYWIVRNSWGEPWGERGWLRIVTSTYKDGKGARYNLAIEEH

mus AVINHIISVAGWGVSNDGIEYWIVRNSWGEPWGEKGWMRIVTSTYKGGTGDSYNLAIESA

:**::******:. :* ***************:**:*****:**.* * ****:*.

gal CAYGDVILP

hum CTFGDPIV-

pan CTFGDPIV-

mus CTFGDPIV-

*::** *:

1. Paired Ig-like receptor 2

gal ------------------------------------------------------------

hum ------------------------------------------------------------

pan MKGGSESRHLCLIPDLKGKARTGEASSGSRTCGRRTSLCTSAKSSWTYRSGRLSWQSIKG

mus ------------------------------------------------------------

gal ------------------------------------------------------------

hum -----------------------------------MGRPLLLPLLPLLLPPAFLQPSGST

pan THLTITQALRQPLHRAPLLPGQLCWSPRPLEKNKAMGRPLLLPLLPLLLPPAFLQPGGST

mus ---------------------------MALLISLPGGTPA-MAQILLLLSSACLHAGNSE

gal ---SLVQAALTQPASVSANPGETVKITCSGGARSYYYGWYQQKSPGSAPVTV------IY

hum GSGPSYLYGVTQPKHLSASMGGSVEIP-----FSFYYPWELATAPDVRISWRRGHFHRQS

pan GSGPSYPYGVTQPKHLSASMGGSVEIP-----FSFYYPWELATAPDVRISWRRGHFHGQS

mus RSNRKNGFGVNQPESCSGVQGGSIDIP-----FSFYFPWKLAKDPQMSIAWRWKDFHGEF

.:.** *. * ::.* *:*: * . *

gal DNTNRPSNIPSRFSGSL------SGSTNTLTITGVQVEDEAIYFCGSWKDSKYSGIFG--

hum FYSTRPPSIHKDYVNRLFL**N**WTEGQKSGFLRISNLQKQDQSVYFCRVELDTRSSGRQQWQ

pan FYSTRPPSIHKDYVNRLFLNWTEGQKSGFLRISNLRKQDQSVYFCRVELDTRSSGRQQWQ

mus IYNSSLPFIHEHFKGRLILNWTQGQTSGVLRILNLKESDQTRYFGRVFLQT-TEGIQFWQ

.. * . : . * . .:. * * .:: .*:: ** :: .*

gal --AGTTLTVL--------------------------------------------------

hum SIEGTKLSITQAVTTTTQRPSSMTTTWRLSSTTTTTGLRVTQGKRRSDSWHISLETAVGV

pan SIEGTKLSITQAVTTTTQRPSSMTTTWRLSSTTTTTGLRVTQGKRRSDSWHISLETAVGV

mus SIPGTQLNVTNATCTPTTLPSTTAATSAHTQ----NDI---TEVKSANIGGLDLQTTVGL

** *.:

gal ------------------------------------------------------------

hum AVAVTVLGIMILGLICLLRWRRRKGQQRTKATTPAREPFQNTEEPYENIRNEGQNTDPKL

pan AVAATVLGIMILGLICLLRWRR-KGQQRTKATTPAREPFQNTEEPYENIRNEGQNTDPKL

mus ATAAAVFLVGVLGLIVFLWWKRRRQGQKTKAEIPAREPLETSE-KHESVGHEGQCMDPKE

gal --------------------------------------------

hum NPKDDGIVYASLALSSSTSPRAPPSHRPLKSPQNETLYSVLKA-

pan NPKDDGIVYASLALSSSTSPRAPPSHRPLKSPQNETLYSVLKA-

mus NPKDNNIVYASISLSSPTSPGTAPNLPVHGNPQEETVYSIVKAK

1. Junctional adhesion molecule 2

gal ---MASRRLRLLLLGYLG-VFCYHKVTGIAIETDN-KNVKAEEFKEAILSCKHKFSKGMS

hum MARRSRHRLLLLLLRYLVVALGYHKAYGFSAPKDQ-QVVTAVEYQEAILACKTP-KKTVS

pan MARRSRHRLLLLLLRYLVVALGYHKAYGFSAPKDQ-QVVTAVEYQEAILACKTP-KKTVS

mus MARSPQGLLMLLLLHYLIVALDYHKANGFSASKDHRQEVTVIEFQEAILACKTP-KKTTS

* **** ** .: ***. *:: .*: : *.. *::****:** .* *

gal LRIEWKKIQSQEVSFVYYNGEFTGDLKDRAEMLNTGIRIRNVTRKDSGTYRCEISAKSEE

hum SRLEWKKLG-RSVSFVYYQQTLQGDFKNRAEMIDFNIRIK**N**VTRSDAGKYRCEVSAPSEQ

pan SRLEWKKLG-RSVSFVYYQQTLQGDFKNRAEMIDFNIRIKNVTRSDAGKYRCEVSAPSEQ

mus SRLEWKKVG-QGVSLVYYQQALQGDFKDRAEMIDFNIRIKNVTRSDAGEYRCEVSAPTEQ

*:****: : **:***: : **:*:****:: .***:****.*:* ****:** :*:

gal GQRLGEATITLTVLVPPTTPICVVPNSAMTGTVVELSCKEAEGSPPSEYQWYKNGVALLE

hum GQNLEEDTVTLEVLVAPAVPSCEVPSSALSGTVVELRCQDKEGNPAPEYTWFKDGIRLLE

pan GQNLEEDTVTLEVLVAPAVPSCEVPSSALSGTVVELRCQDKEGNPAPEYTWFKDGIRLLE

mus GQNLQEDKVMLEVLVAPAVPACEVPTSVMTGSVVELRCQDKEGNPAPEYIWFKDGTSLLG

**.* * .: * *** *:.* * **.*.::*:**** *:: **.* ** *:*:* **

gal KTGTGSARTANITYTMNKKSGTLIFNTVSKNDTGEYFCVASNGIGLPQKCSMKRMQVDDL

hum NPRLGS-QST**N**SSYTMNTKTGTLQFNTVSKLDTGEYSCEARNSVGY-RRCPGKRMQVDDL

pan NPRLGS-QSTNSSYTMNTKTGTLQFNTVSKLDTGEYSCEARNSVGY-RRCPGKRMQVDDL

mus NPKGGT--HNNSSYTMNTKSGILQFNMISKMDSGEYYCEARNSVGH-RRCPGKRMQVDVL

: *: * :****.*:* * ** :** *:*** * * *.:* ::* ****** *

gal NVSGIIAAVVIVALVMALCGLGVLYAQKKGYFTKESSSQKSNSQ----STSEKDFKHTKS

hum **N**ISGIIAAVVVVALVISVCGLGVCYAQRKGYFSKETSFQKSNSSSKATTMSENDFKHTKS

pan NISGIIAAVVVVALVISVCGLGVCYAQRKGYFSKETSFQKSNSSSKATTMSENDFKHTKS

mus NISGIIATVVVVAFVISVCGLGTCYAQRKGYFSKETSFQKGSPASKVTTMSENDFKHTKS

*:*****:**:**:*:::****. ***:****:**:* **.. : **:*******

gal FVI

hum FII

pan FII

mus FII

*:*

1. Thyroxin-binding globulin

gal MKPTFSLCFLLAGLYSVAQCHQRPRYHNKQDNSKGAYYWGSSSHREGVFPNKNKTFVKVV

mus MSVFFYLFVLVFGLQATIHCAPHNSSEGK--------------VTTCHLPQQNATLYKMP

hum MSPFLYLVLLVLGLHATIHCAS---PEGK--------------VTACHSSQP**N**ATLYKMS

pan MSPFLYLVLLVLGLHATIHCAS---PEGK--------------VTACHSSQPNATLYKMS

*. : * .*: ** :. :* ..* : * *: *:

gal HSNADFALSFYKLVASEATDQNIFFSPISISTSLAMLALGAKSVTLTQILEGLAFNLKKT

mus SINADFAFSLYRRLSVENPDLNIFFSPVSISVALAMLSFGSGSSTQTQILEVLGFNLTDT

hum SINADFAFNLYRRFTVETPDKNIFFSPVSISAALVMLSFGACCSTQTEIVETLGF**N**LTDT

pan SINADFAFNLYRRFTVETPDKNIFFSPVSISAALVMLSFGACCSTQTEIVETLGFNLTDT

*****:.:*: .: * * ******:***.:*.**::*: . * *:*:* *.***..*

gal QDQEIHEGFCQLLHMLNRSDSDLHLSLGNTLFIEETLKPLQKFLDDAKSFYQSEVLSADF

mus PVTELQQGFQHLICSLNFPKNELELQMGNAVFIGQQLKPLAKFLDDVKTLYETEVFSTDF

hum PMVEIQHGFQHLICSLNFPKKELELQIGNALFIGKHLKPLAKFLNDVKTLYETEVFSTDF

pan PMVEIQHGFQHLICSLNFPKKELELQIGNALFIGKHLKPLAKFLNDVKTLYETEVFSTDF

*::.** :*: ** ..:*.*.:**::** : **** ***:*.*::*::**:*:**

gal NNSSGAENQINSYIEEKTNGKIVKLVENLDPLTAMVLVNYVFFKAHWEKPFSDSYTKK-E

mus SNVSAAQHKINSYVEKQTKGKIVGLIQGLKLNIIMILVNYIHFRAQWANPFRVSKTEESS

hum S**N**ISAAKQEINSHVEMQTKGKVVGLIQDLKPNTIMVLVNYIHFKAQWANPFDPSKTEDSS

pan SNISAAKQEINSHVEMQTKGKVVGLIQDLKPNTIMVLVNYIHFKAQWANPFDPSKTEDSS

.* *.*:::***::* :*:**:* *::.*. *:****:.*:*:* :** * *:. .

gal DFFVDKKTSVKVDMMYRKGYYRNYFDEELSCWLVQIPYNGNAAALFVLPDEGKMKQVEDA

mus NFSVDKSTTVQVPMMHQLEQYYHYVDMELNCTVLQMDYSENALALFVLPKEGHMEWVEAA

hum SFLIDKTTTVQVPMMHQMEQYYHLVDMEL**N**CTVLQMDYSKNALALFVLPKEGQMESVEAA

pan SFLIDKTTTVQVPMMHQMEQYYHLVDMELNCTVLQMDYSKNALALFVLPKEGQMESVEAA

.* :**.*:*:* **:: * : .* **.* ::*: *. ** ******.**:*: ** *

gal LLKRTVSKWEKLLQHRKIHLHIPKLSISGTYDVKKIVREVGIIDLFTAQADLSGITEDPG

mus MSSKTLKKWNYLLQKGWVELFVPKFSISATYDLGSTLQKMGMRDAFAESADFPGITEDSG

hum MSSKTLKKWNRLLQKGWVDLFVPKFSISATYDLGATLLKMGIQHAYSENADFSGLTEDNG

pan MSSKTLKKWNRLLQKGWVDLFVPKFSISATYDLGATLLKMGIQHAYSENADFSGLTEDNG

: .:*:.**: ***: :.*.:**:***.***: : ::*: . :: .**: *:*** *

gal LMVSKVIHRAVLNVHENGTEAAGVTVTEITWRSGDFPRPPRVRFNRPFLLMILDKYAHTI

mus LKLSYAFHKAVLHIGEEGTKEGASPEVGSLDQQEVPPLHPVIRLDRAFLLMILEKRTRSV

hum LKLSNAAHKAVLHIGEKGTEAAAVPEVELSDQPENTFLHPIIQIDRSFMLLILERSTRSI

pan LKLSNAAHKAVLHIGEKGTEAAAVPEVELSDQPENTFLHPIIQIDRSFMLLILERSTRSI

* :* . *:***:: *:**: .. . : * ::::* *:*:**:: ::::

gal LFIGKIVNPLKNN

mus LFLGKLVNPTKQ-

hum LFLGKVVNPTEA-

pan LFLGKVVDPTEA-

**:**:*:* :

1. Adipocyte plasma membrane associated protein

gal MNEAEGLRQRRPLRPQVITED-SPAQEAKEGSAYSSKVFRVTFLTLAASLAVPLLGATVL

hum MSEADGLRQRRPLRPQVVTDDDGQAPEAKDGSSFSGRVFRVTFLMLAVSLTVPLLGAMML

pan MSEADGLRQRRPLRPQVVTDDDGQAPEAKDGSSFSGRVFRVTFLMLAVSLTVPLLGAMML

mus MSEADGLRQRRPLRPQVVTD-DGQVPEVKEGSSFSGRVFRMTFLMLAVSLAIPLLGAMML

*.**:************:*: . . *.*:**::*.:***:*** **.**::***** :*

gal LDCPIDPQPISLKEPPLLTGVLEPNNKLQKAERLWENQLVGPESIVNIGDVLFTGTADGK

hum LESPIDPQPLSFKEPPLLLGVLHPNTKLRQAERLFENQLVGPESIAHIGDVMFTGTADGR

pan LESPIDPQPLSFKEPPLLLGVLHPNTKLRQAERLFENQLIGPESIAHIGDVMFTGTADGR

mus LESPIDPQSFSFKEPPFMFGVLHPNTKLRQAERLFENQLSGPESIVNIGDVLFTGTADGR

*:.***** :*:****:: ***.**.**::****:**** *****.:****:*******:

gal ILKIEDGEVQTVARIGHGPCGTPEDEPTCGRPLGIRVGPNNTLFVADAYYGLYEVNPGTG

hum VVKLENGEIETIARFGSGPCKTRDDEPVCGRPLGIRAGP**N**GTLFVADAYKGLFEVNPWKR

pan VVKLENGEIETIARFGSGPCKTRDDEPVCGRPLGIRAGPNRTLFVADAYKGLFEVNPWKR

mus VVKLENGEIETIARFGSGPCKTRDDEPTCGRPLGIRAGPNGTLFVVDAYKGLFEVNPQKR

::*:*:**::*:**:* *** * :***.********.*** ****.*** **:**** .

gal ETKMLVSTKTLIEGQKLSFLNDLTVTQDGRKIYFTDSSSKWQRRDFLFLVMEGTDDGRLL

hum EVKLLLSSETPIEGK**N**MSFVNDLTVTQDGRKIYFTDSSSKWQRRDYLLLVMEGTDDGRLL

pan EVKLLLSSETPIEGKKMSFVNDLTVTQDGRKIYFTDSSSKWQRRDYLLLVMEGTDDGRLL

mus SVKLLLSSETPIEGKKMSFVNDLTVTRDGRKIYFTDSSSKWQRRDYLLLVMEATDDGRLL

..*:*:*::* ***:::**:******:******************:*:****.*******

gal EYDTVTKEVKVLMVGLRFPNGVQLSPAEDFVLVLETAMARIRRYYVSGLMKGGADMFVEN

hum EYDTVTREVKVLLDQLRFPNGVQLSPAEDFVLVAETTMARIRRVYVSGLMKGGADLFVEN

pan EYDTVTREVKVLLDQLRFPNGVQLSPAEDFVLVAETTMARIRRVYVSGLMKGGADLFVEN

mus EYDTVTKEVKVLLDQLQFPNGVQLSPEEDFVLVAETTMARIRRVYVSGLMKGGADMFVEN

******:*****: *:********* ****** **:****** ***********:****

gal MPGLPDNIRLSSSGGYWVAMPVVRPNPGFSMLDFLSEKPWIKRMIFKLLSQETVTKLLPK

hum MPGFPDNIRPSSSGGYWVGMSTIRPNPGFSMLDFLSERPWIKRMIFKLFSQETVMKFVPR

pan MPGFPDNIRPSSSGGYWVGMSTIRPNPGFSMLDFLSERPWIKRMIFKLFSQETVMKFVPR

mus MPGFPDNIRPSSSGGYWVAAATIRANPGFSMLDFLSDKPFIKRMIFKMFSQETVMKFVPR

***:***** ********. .:* ***********::*:*******::***** *::*:

gal RSLVVELSETGSYRRSFHDPTGLTVPYVSEAHEHNGYLYLGSFRSPFICRLNLQHV

hum YSLVLELSDSGAFRRSLHDPDGLVATYISEVHEHDGHLYLGSFRSPFLCRLSLQAV

pan YSLVLELSDSGAFRRSLHDPDGLVATYISEVHEHDGHLYLGSFRSPFLCRLSLQAV

mus YSLVLEVSDSGAFRRSLHDPDGQVVTYVSEAHEHDGYLYLGSFRSPFICRLSLQSI

***:*:*::*::***:*** * .. *:**.***:*:**********:***.** :

1. Lysosome-associated membrane glycoprotein 3

gal MGRSKSHLVLLAFVCAFSSCCAEVALGVR----LSPQTTSFHHTITSALPLSVYHSPPHQ

mus MPGQISAVA-----VLFLSLTVILH-GYQIREKEFPKARGYLQYTATS-AEQITTKPLLQ

hum MPRQLSAAA-----ALFASLAVILHDGSQMRAKAFPETRDYSQPTAAA-TVQDIKKPVQQ

pan MPRQLSAAA-----ALFASLAVILHDGSQMTAKAFPETRDYSQPTAAA-TVQDIKKPVQQ

* . * . * * . : * : *:: .: : ::: . .* *

gal STTVQPNTTGSISHTTTLQ-TTDQHWVTTAPASHMTTQAG-------ANTSKAHGQTSS-

mus LINQR-------SHITLASRFKDDYIQMAAETSAIENTAHITMKTVTPVTTKSLPPISSA

hum PAKQA-------PHQTLAARFMDGHITFQTAA-------TVKIPTTTPATTKNTATTSPI

pan PAKQA-------PHQTLAARFMDGHITFQTAA-------TVKTPTTTPATTKNTATTSLI

. * * * : : : *:* *

gal --TAVTTTAADTAAAGQATTQAMETVTQAVKNVTVPPYNQITTHV-----DTVTNTTIEN

mus SYTFVRS-----NNAHMTASSTDDTIGSGSI-------AHLPVPTTRASLAIVNYITGRA

hum TYTLVTTQATP-**N**NSHTAPPVTEVTVGPSLAPYSLPPTITPPAHTTGTSSSTVSHTTG**N**T

pan TYTLVTTQATS-NNSHTAPPVTEVTVGPSLAPYSLPPTITPPAHTTGTSSSTISHTTGNT

* * : : : : *: . . . :. * .

gal TTSKTQTTTTATNTTATTSST--VKPTTSSTNHTTSGSSTATTMTNATTTHQGTHTTIPS

mus TQLGGQTTLPKTFFTASHKSTTNQRPTLST-----------NVLGTSTPTHKDR-STTSP

hum TQPS**N**QTTLPATLSIALHKSTTGQKPVQPT-----------HAPGTTAAAH**N**TT-RTAAP

pan TQPSNQTTLPATLSIALHKSTTGQKPVQPT-----------HAPGTTAAAHNTT-RTAAP

* *** * * .** :*. : . .:: :*: *

gal TTMMVRPTLAPQPSPIPTGTYIISSSNKTCIKAVMGLQLMALSTQ--KKQMKYLTVSPNA

mus VPLVPRPTLVTWSSPAKIGTYEVLNGSRLCIKAEMGLALIVQEKDLDSATQRYFNIDPSL

hum ASTVPGPTLAPQPSSVKTGIYQVL**N**GSRLCIKAEMGIQLIVQDKESVFSPRRYFNIDP**N**A

pan ASTVPGPTLAPQPSSVKTGIYQVLNGSRLCIKAEMGIQLIVQDKESVFSPRRYFNIDPNA

. : ***. * * * : ...: **** **: *:. ..: :*:.:.*.

gal TQISGSCGMVQSVLNITFPGGFISFVFVKKDPTYYVSTIEAELQLPSEGILYYV-AIRQQ

mus THASGKCDSQKSNLFLNFQGGSVNITFTKEENLYYISEVGAYLTISNTEKTYQGKKNTLM

hum TQASGNCGTRKSNLLLNFQGGFV**N**LTFTKDEESYYISEVGAYLTVSDPETIYQGIKHAVV

pan TQASGNCGTRKSNLLLNFQGGFVNLTFTKDEESYYISEVGAYLTVSDPETIYQGIKHAVV

*: **.*. :* * :.* ** :.:.*.*.: **:* : * * : . *

gal HFTAKLGNSYKCASKQTFGLERTYQLVIVNMQLQAFDIVDNQFGREEECFLDKSTKAVPV

mus MFETVVGHSFKCVSEQSIQLSAQLQMKTMNIHLQAFDFEGDSFGNVNECLSDYTVVL--P

hum MFQTAVGHSFKCVSEQSLQLSAHLQVKTTDVQLQAFDFEDDHFGNVDECSSDYTIVL--P

pan MFQTAVGHSFKCVSEQSLQLSAHLQLKTTDVQLQAFDFEDDHFGNVDECSSDYTIVL--P

* : :*:*:**.*:*:: *. *: :::*****: .: **. :** * :

gal AVGLSILGLLVIVFVTFLISRRKPYRGYERI

mus MVAIIVVVICVVGLSVYKIRQRHQSSAYQRI

hum VIGAIVVGLCLMGMGVYKIRLRCQSSGYQRI

pan VIGAIVVGLCLMGMGVYKIRLRCQSSGYQRI

:. :: : :: : .: * * .*:**

1. Calreticulin

gal MSRLCLPLLLGAVLAVTAAGPAQFFREEFLDGDSWTQRWVESKHKSDYGRFVLTAGKFYG

mus -MLLSVPLLLG-LLGLAAADPAIYFKEQFLDGDAWTNRWVESKHKSDFGKFVLSSGKFYG

hum -MLLSVPLLLG-LLGLAVAEPAVYFKEQFLDGDGWTSRWIESKHKSDFGKFVLSSGKFYG

pan -MLLSVPLLLG-LLGLAVAEPAVYFKEQFLDGDGWTSRWIESKHKSDFGKFVLSSGKFYG

*.:***** :*.::.* ** :*:*:*****.**.**:*******:*:***::*****

gal DAEKDKGIQTSQDARFYALSSRFEPFSNRDKTLVVQFTVKHEQNIDCGGGYVKLFPASLN

mus DLEKDKGLQTSQDARFYALSAKFEPFSNKGQTLVVQFTVKHEQNIDCGGGYVKLFPSGLD

hum DEEKDKGLQTSQDARFYALSASFEPFSNKGQTLVVQFTVKHEQNIDCGGGYVKLFPNSLD

pan DEEKDKGLQTSQDARFYALSASFEPFSNKGQALVVQFTVKHEQNIDCGGGYVKLFPNSLD

* *****:************: ******:.::************************ .*:

gal QEDMHGDSEYNIMFGPDICGPGTKKVHVIFNYKGKNVLINKDIRCKDDEFTHLYTLIVRP

mus QKDMHGDSEYNIMFGPDICGPGTKKVHVIFNYKGKNVLINKDIRCKDDEFTHLYTLIVRP

hum QTDMHGDSEYNIMFGPDICGPGTKKVHVIFNYKGKNVLINKDIRCKDDEFTHLYTLIVRP

pan QTDMHGDSEYNIMFGPDICGPGTKKVHVIFNYKGKNVLINKDIRCKDDEFTHLYTLIVRP

* **********************************************************

gal DNTYEVKIDNSKVESGSLEDDWDFLPPKKIKDPEAKKPDDWDERAKIDDPEDSKPEDWDK

mus DNTYEVKIDNSQVESGSLEDDWDFLPPKKIKDPDAAKPEDWDERAKIDDPTDSKPEDWDK

hum DNTYEVKIDNSQVESGSLEDDWDFLPPKKIKDPDASKPEDWDERAKIDDPTDSKPEDWDK

pan DNTYEVKIDNSQVESGSLEDDWDFLPPKKIKDPDASKPEDWDERAKIDDPTDSKPEDWDK

***********:*********************:* **:*********** *********

gal PEHIPDPDAKKPEDWDEEMDGEWEPPVIQNPEYKGEWRPRQIDNPDYKGKWVHPEIDNPE

mus PEHIPDPDAKKPEDWDEEMDGEWEPPVIQNPEYKGEWKPRQIDNPDYKGTWIHPEIDNPE

hum PEHIPDPDAKKPEDWDEEMDGEWEPPVIQNPEYKGEWKPRQIDNPDYKGTWIHPEIDNPE

pan PEHIPDPDAKKPEDWDEEMDGEWEPPVIQNPEYKGEWKPRQIDNPDYKGTWIHPEIDNPE

*************************************:***********.*:********

gal YTPDPNLYAYDSFGVIGLDLWQVKSGTIFDNFLITDDEKFAEEFGNETWGATKEAERKMK

mus YSPDANIYAYDSFAVLGLDLWQVKSGTIFDNFLITNDEAYAEEFGNETWGVTKAAEKQMK

hum YSPDPSIYAYDNFGVLGLDLWQVKSGTIFDNFLITNDEAYAEEFG**N**ETWGVTKAAEKQMK

pan YSPDPSIYAYDNFGVLGLDLWQVKSGTIFDNFLITNDEAYAEEFGNETWGVTKAAEKQMK

*:** .:****.*.*:*******************:** :**********.** **::**

gal EQQDEEQRQKQEEEDKQRKEEEGDEDGDGDDEEDEEDEEDEA--EK-------------

mus DKQDEEQRLKEEEEDKKRKEEEEAEDKEDDDDRDEDEDEEDEK-EEDEEESPGQAKDEL

hum DKQDEEQRLKEEEEDKKRKEEEEAEDKEDDEDKDEDEEDEEDKEEDEEEDVPGQAKDEL

pan DKQDEEQRLKEEEEDKKRKEEEEAEDKEDDEDKDEDE---EDKEEDEEEDVPGQAKDEL

::****** *:*****:***** ** :.*::.**:: : *.

1. Serine protease Hepsin

gal MEGKTSPTTASPSSLHASAASSIFSIRPPQPRENVLGISFKPYSPDSIPAPVPCTACEST

hum ---------------------------------------------------------MAQ

pan ---------------------------------------------------------MAQ

mus ----------------------------------------------------------MA

gal RSSMFRAPCMSQRRLALIFCVSVLIVLIVALILLFMFWRSQTGIVYKEPAETCKDDPVRC

hum KEGGRTVPCCSRPKVAALTAGTLLLLTAI----GAASWAIVAVL----------------

pan KEGGRTVPCCSRPKVAALTAGTLLLLTAI----GAASWAIVAVL----------------

mus KEGGRTAACCSRPKVAALIVGTLLFLTGI----GAASWAIVTIL----------------

:.. . * *: ::* : ::*:: : * : :

gal DGVVDCSQRSDELGCVRFSSDQSLLHVYSSTENQWLPVCSSAWDESFSRKTCRQLGFQNA

hum -----LRSDQEPLYPVQVSSADARLMVFDKTEGTWRLLCSSRSNARVAGLSCEEMGFLRA

pan -----LRSDQEPLYPVQVSSADARLMVFDKTEGTWRLLCSSRSNARVAGLSCEEMGFLRA

mus -----LQSDQEPLYQVQLSPGDSRLAVFDKTEGTWRLLCSSRSNARVAGLGCEEMGFLRA

. .: * *:.* :: * *:..**. * :*** : .: *.::** .*

gal SQTEYVPLHVSGKSLT-----VAD----ERDTIQQSLNSSQCLTGKFVSLRCTTCGQRI-

hum LTHSELDVRTAGA**N**GTSGFFCVDEGRLPHTQRLLEVISVCDCPRGRFLAAICQDCGRRKL

pan LTHSELDVRTAGANGTSGFFCVDEGRLPHTQRLLEVISVCDCPRGRFLAAICQDCGRRKL

mus LAHSELDVRTAGANGTSGFFCVDEGGLPLAQRLLDVISVCDCPRGRFLTATCQDCGRRKL

. : ::.:* . * * : : : : :. .:* *:*:: * **:*

gal -SGRIIGGKETSVSKWPWQVSVQYGPVHICGGTIIDAQWVLTAAHCFFMNSMKILDDWKV

hum PVDRIVGGRDTSLGRWPWQVSLRYDGAHLCGGSLLSGDWVLTAAHCFPE-RNRVLSRWRV

pan PVDRIVGGRDTSLGRWPWQVSLRYDGAHLCGGSLLSGDWVLTAAHCFPE-RNRVLSRWRV

mus PVDRIVGGQDSSLGRWPWQVSLRYDGTHLCGGSLLSGDWVLTAAHCFPE-RNRVLSRWRV

.**:**:::*:.:******::*. .*:***:::..:********* ::*. *:*

gal YGGVSDLKQPMEG-IPVSQVIINSNY------SDDHDDYDIALMKLSRPLTLSAQIRPAC

hum FAGAVAQASPHGLQLGVQAVVYHGGYLPFRDPNSEENSNDIALVHLSSPLPLTEYIQPVC

pan FAGAVAQASPHGLQLGVQAVVYHGGYLPFRDPNSEENSNDIALVHLSSPLPLTEYIQPVC

mus FAGAVARTSPHAVQLGVQAVIYHGGYLPFRDPTIDENSNDIALVHLSSSLPLTEYIQPVC

:.*. .* : *. *: :..* . :.:. ****::** * *: *:*.*

gal LPMHGQRFQTGRSCFITGFGKTRENEDNTSPKLREAEVKLIDYKICNSDKVYEGYLTPRM

hum LPAAGQALVDGKICTVTGWGNTQYYGQ-QAGVLQEARVPIISNDVCNGADFYGNQIKPKM

pan LPAAGQALVDGKICTVTGWGNTQYYGQ-QAGVLQEARVPIISNDVCNGADFYGNQIKPKM

mus LPAAGQALVDGKVCTVTGWGNTQFYGQ-QAMVLQEARVPIISNEVCNSPDFYGNQIKPKM

** ** : *: * :**:*:*: : : *:**.* :*. .:**. ..* . :.*:*

gal MCAGYLQGGKDACQGDSGGPLVCEDN----GRWYVAGVTSWGTGCGQKNKPGVYTRVTKL

hum FCAGYPEGGIDACQGDSGGPFVCEDSISRTPRWRLCGIVSWGTGCALAQKPGVYTKVSDF

pan FCAGYPEGGIDACQGDSGGPFVCEDSISRTPRWRLCGIVSWGTGCALAQKPGVYTKVSDF

mus FCAGYPEGGIDACQGDSGGPFVCEDSISGTSRWRLCGIVSWGTGCALARKPGVYTKVTDF

:**** :** **********:****. ** :.*:.******. .******:*:.:

gal LGWIYSKMESENN--------

hum REWIFQAIKTHSEASGMVTQL

pan REWIFQAIKTHSEASGMVTQL

mus REWIFKAIKTHSEASGMVTQP

**:. :::..:

1. Gastric instrinsic factor

gal MQRQRGTAGTEVLRMLGVALSTAVLLALAGCGVEGTDPQNCTVSAEERARMLG---ILQL

mus ------------MAWLTLYL-LSVLW--AVAGTSTRAQSSCSVPPDQQPWVDGLQALMEN

hum ------------MAWFALYL-LSLLW--ATAGTSTQTQSSCSVPSAQEPLVNGIQVLMEN

pan ------------MAWFALYL-LSLLW--ATAGTSTQTRSSCSVPSAQEPLVNGIQVLMEN

: : : * ::* * .*.. ..*:* :. : * :::

gal SAMDSGTPNPSVLLALNLAGDSSKARQELLERIKETAAKQAKDMSSGQVALYTLALRSSC

mus SVTDSDFPNPSILIAMNLAGAYNVEAQKLLTYQ--LMASDSADLTSGQLALTVMALTSSC

hum SVTSSAYPNPSILIAMNLAGAYNLKAQKLLTYQ--LMSSDNNDLTIGQLGLTIMALTSSC

pan SVTSSAYPNPSILIAMNLAGAYNLKAQKLLTYQ--LMSSDTNDLTIGQLGLTIMALTSSC

*. .* ****:*:*:**** . *:** :.: *:: **:.* :** ***

gal CDPGDVAAHGQSVDLLSILQEKTGQELTHLEQNGTPKSSLFSVGLDAQALCVTGAGDYES

mus RDPGSKVSTL---------LKKME--NWSPSSPGAESSAFYGPGLAILALCQKSSEATLP

hum RDPGDKVSIL---------QRQME--NWAPSSPNAEASAFYGPSLAILALCQKNSEATLP

pan RDPGDKVSIL---------QRQME--NWAPSSPNAEASAFYGPSLAILALCQKNSEATLP

***. .: .: .. .: *:::. .* *** ..:

gal AATILAKQLQRSQDKLSVDEQAMMALALVCAYNRTEHKD---VQDLLNRTLTMVSNGFLD

mus IAVRFAKTLMMEPSPFNVDTGAVATLALTCMYNKIPVGSQENYRDLFGQALKAIVE-KIS

hum IAVRFAKTLLANSSPFNVDTGAMATLALTCMYNKIPVGSEEGYRSLFGQVLKDIVE-KIS

pan IAVRFAKTLLANSSPFNVDTGAMATLALTCMYNKIPVGSEEGYRSLFGQVLKDIVE-KIS

*. :** * . . :.** *: :***.* **: . :.*:.:.*. : : :.

gal KQAEGNGIIGNIYSTGLAMQLLLAAGKFYAPRPWDCTQPVAA----ITAQHLQQPMAVAQ

mus LRIKADGIIGDIYSTGLAMQALSVTP-EQPTKKWDCEKTMHTILNEIKQGKFQNPMSIAQ

hum MKIKDNGIIGDIYSTGLAMQALSVTP-EPSKKEWNCKKTTDMILNEIKQGKFHNPMSIAQ

pan MKIKDNGIIGDIYSTGLAMQALSVTP-EPPKKEWNCKKTMDMILNEIKQGKFHNPMSIAQ

: : :****:********* * .: : *:* : *. ::::**::**

gal ALPALVGRTYLDSASLDCSPEAPTTAGMQLDTVLTKGTTPEQAGSNITVNYTITNEVRGK

mus ILPSLKGKTYLDVPQVTCGPDHEVPPTLTD-----YPTPVPTSVSNITVIYTINNQLRGV

hum ILPSLKGKTYLDVPQVTCSPDHEVQPTLPS-----NPGPGPTSAS**N**ITVIYTINNQLRGV

pan ILPSLKGKTYLDVPQVTCSPDHEVQPTLPS-----NPGPGPTSASNITVIYTINNQLRGV

**:* *:**** .: *.*: . : : ***** ***.*::**

gal HFSYST--EVEVPAGSVLLVVLEEAQKSNKTIFSFKTESTFWGPMVVSIHGLAASENDRT

mus DLLFNVTIEVSVKSGSVLLAVLEEAQRKN-SMFKFETTMTSWGLIVSSINNIAENVNHKT

hum ELLF**N**ETI**N**VSVKSGSVLLVVLEEAQRKN-PMFKFETTMTSWGLVVSSINNIAENVNHKT

pan ELLFNETINVSVKSGSVLLVVLEEAQRKN-PMFKFETTMTSWGLVVSSINNIAENVNHKT

.: :. :*.* :*****.******:.* :*.*:* * ** :* **:.:* . *.:*

gal FWQFFNGSVPLQEGVGTYKPQDGEHIRAVFSIY

mus YWEFLSGKTPLDEGVAYYIPFNHEHITANFTQY

hum YWQFLSGVTPLNEGVADYIPFNHEHITA**N**FTQY

pan YWQFLSGVTPLNEGVADYIPFNHEHITANFTQY

:*:*:.* .**:***. * * : *** * *: *

1. LAMP1

gal ---MGRAARAV-------LLGFLQA--SSSFDVRDSTGKVCIIANLTVAFSVEYKSSGQK

mus MAAPGA-RRPLLL---LLLAGLAHGASA-LF-EVKNNGTTCIMASFSASFLTTYETANGS

hum MAAPGSARRPLLLLLLLLLLGLMHCASAAMFMVKNG**N**GTACIMA**N**FSAAFSVNYDTKSGP

pan MAAPGSARRPLLLLLLLLLLGLVHCASAAMFMVKNGNGTACIMANFSASFSVNYDTKSGP

* * : * *: : : * ...*..**:*.::.:* . *.: .

gal QFAHFFLPQNATS-QSHSSCGEGNTSHPILALSFGAGHLISLNFSKTLDKYQVEELTFHY

mus QIVNISLPASAEVLKNGSSCGKENVSDPSLTITFGRGYLLTLNFTKNTTRYSVQHMYFTY

hum K**N**MTFDLPSDATVVL**N**RSSCGKE**N**TSDPSLVIAFGRGHTLTL**N**FTR**N**ATRYSVQLMSFVY

pan KNMTFDLPSDATVVLNRSSCGKENTSDPSLVIAFGRGHTLTLNFTRNATRYSVQLMSFVY

: : ** .* . ****: *.*.* *.::** *: ::***::. :*.*: : * *

gal NLSDETLFPNATEGKVMVATQKSVIQARIGTEYRCINSKYIRMKHVNITFSNVTLEAYPT

mus NLSDTEHFPNAISKEIYTMDSTTDIKADINKAYRCVSDIRVYMKNVTVVLRDATIQAYLS

hum **N**LSDTHLFP**N**ASSKEIKTVESITDIRADIDKKYRCVSGTQVHMN**N**VTVTLHDATIQAYLS

pan NLSDTHLFPNASSKEIKTVESITDIRADIDKKYRCVSGTQVHMNNVTVTLHDATIQAYLS

**** **** . :: . . : *:* *.. ***:.. : *::*.:.: :.*::** :

gal NDTFSANKTECREDMVSTTTVAPTTPKHATSQVPTTSPAPTAAPSSPAVGKYNVTGANGT

mus SGNFSKEETHCTQDGPSPTTGPP-------------SPSPPLVPTNPTVSKYNVTGNNGT

hum **N**SSFSRGETRCEQDRPSPTTAPPAP----------PSPSPSPVPKSPSVDKY**N**VSGT**N**GT

pan NSSFSRGETRCEQDRPSPTTAPPAP----------PSPSPSPVPESPSVDKYNVSGTNGT

...** :*.* :* * ** * **:* .* .*:*.****:* ***

gal CVLASMGLQLNITYVKKDEKMGLDLLNFIPHNTSASGMCESTSAFLNLAFEK-TKITFHF

mus CLLASMALQLNITYLKKDNKTVTRAFNISPNDT-SSGSCGINLVTLKVENKN-RALELQF

hum CLLASMGLQL**N**LTYERKD**N**TTVTRLLNINP**N**KTSASGSCGAHLVTLELHSEGTTVLLFQF

pan CLLASMGLQLNLTYERKDNTTVTRLLNINPNKTSASGSCGAHLVTLELHSEGSTVLLFLF

*:****.****:** :**:. :*: *:.* :** * . *:: : : : *

gal VLNASSEKFFLQGVNVSTTLPSEAKAPTFEASNDSMSELRATVGNSYKCSAEENFQVTDK

mus GMNASSSLFFLQGVRLNMTLPD-ALVPTFSISNHSLKALQATVGNSYKCNTEEHIFVSKM

hum GM**N**ASSSRFFLQGIQLNTILPD-ARDPAFKAA**N**GSLRALQATVGNSYKCNAEEHVRVTKA

pan GMNASSSRFFLQGIQLNTTLPD-ARDPAFKAANGSLRALQATVGNSYKCNAEEHVRVTKA

:****. *****:.:. **. * *:*. :* *: *:*********.:**:. *:.

gal ALVNVFNVQVQAFKVDGDKFGAVEECQLDENNMLIPIIVGAALAGLVLIVLIAYLIGRKR

mus LSLNVFSVQVQAFKVDSDRFGSVEECVQDGNNMLIPIAVGGALAGLVLIVLIAYLIGRKR

hum FSVNIFKVWVQAFKVEGGQFGSVEECLLDENSMLIPIAVGGALAGLVLIVLIAYLVGRKR

pan FSVNIFKVWVQAFKVEGGQFGSVEECVLDENNMLIPIAVGGALAGLVLIVLIAYLVGRKR

:*:*.* ******:..:**:**** * *.***** **.**************:****

gal SHAGYQTI

mus SHAGYQTI

hum SHAGYQTI

pan SHAGYQTI

********

1. Poliovirus receptor

gal MAAIGAVSRVSPAPIGRRELFPAAGASPRWWWWQQRRRRAGSALPPGLGLLGLVFSRFCC

mus --------------MARAAVLPPSRLSPTLP------------------LLPLLLLLLQE

hum --------------MARA-------MAAAWPLL----------------LVALLVLSWPP

pan --------------MARA-------MAAAWLLL----------------LVALLVLSWPP

:.* : *: *:.

gal SA--LAGPVVDPHVTAVWGKKVALKCIIDVNE----TITQVSWEKIHGKTSETIAVHHPE

mus TGAQDVRVRVLPEVRGRLGGTVELPCHLLPPTTE--RVSQVTWQRLDGT---VVAAFHPS

hum PGTGDVVVQAPTQVPGFLGDSVTLPCYLQVPNMEVTHVSQLTWARHGES--GSMAVFHQT

pan PGTGDVVVQAPTQVPGFLGDSVTLPCYLQVPNMEVTHVSQLTWARHGES--GSMAVFHQT

. . . .* . * .* * * : ::*::* : . :*..*

gal YGISIQEKYQG--KVS------FKNYSLTDATIILKNVSFSDAGEYICKAVTFPLGNSQS

mus FGVDFPNSQFSKDRLSFVRARPETNADLRDATLAFRGLRVEDEGNYTCEFATFPNGTRRG

hum QGPSYSE----SKRLEFVAAR--LGAELR**N**ASLRMFGLRVEDEG**N**YTCLFVTFPQGSRSV

pan QGPSYSE----SKRLEFVAAR--LGAELRNASLRMFGLRVEDEGNYTCLFVTFPQGSRSV

* . : ::. . .* :*:: : .: ..* *:* * .*** *.

gal SITVTVLVEPVVSLTKGPNPLIDGANQTIAAICTAATGKPAAEIDWEGGLGEMESSS--T

mus VTWLRVIAQPENHAEA--QEVTIGPQSVAVARCVSTGGRPPARITWISSLGGEAKDTQEP

hum DIWLRVLAKPQNTAEV--QKVQLTGEPVPMARCVSTGGRPPAQITWHSDLGGMP**N**TSQVP

pan DIWLRVLAKPQNTAEV--QKVQLTGEPVPVARCVSTGGRPPAQITWRSDLGGMPNTSQVP

: *:.:* : : : . * *.:: *:* *.* * ..** . :

gal LFPNETVTVISQYTIIPTRFARGRRITCIVRHPALEKEIRFSHVLDIQYAPEVSVTGYDG

mus GIQAGTVTIISRYSLVPVGRADGVKVTCRVEHESFEEPILLPVTLSVRYPPEVSISGYDD

hum GFLSGTVTVTSLWILVPSSQVDGK**N**VTCKVEHESFEKPQLLTV**N**LTVYYPPEVSISGYDN

pan GFLSGTVTVTSLWILVPSSQVDGKSVICKVEHESFEKPQLLTVNLTVYYPPEVSISGYDN

: ***: * : ::* . * : * *.* ::*: : * : * ****::***.

gal NWFIGRENVQLRCNADANPLPMEFMWTRLDGQWPEGLLSVNNTLQFSSPLTYNYTGTYIC

mus NWYLGRSEAILTCDVRSNPEPTDYDWSTTSGVFPASAVAQGSQL-LVHSVDRMVNTTFIC

hum NWYLGQNEATLTCDARSNPEPTGY**N**WSTTMGPLPPFAVAQGAQL-LIRPVDKPI**N**TTLIC

pan NWYLGQNEATLTCDARSNPEPTGYNWSTTMGPLPPFAVAQGAQL-LIRPVDKPINTTLIC

**::*:.:. * *:. :** * : *: * * :: . * : : . * **

gal KVTNSLGQRSDQKTIYILDMPFKQTSSVAVAGAVIGAVLALFIITIFVT--VLLTPRKKR

mus TATNAVGTGRAEQVILVRESPSTAGAGA-TGGIIGGI-IAAIIATAVAGTGILICRQQRK

hum **N**VTNALGARQAELTVQVKEGPPSEHSGI-SRNAIIFLVLGILVFLILLGIGIYFYWSKCS

pan NVTNALGARQAELTVQVKEGPPSEHSGM-SGNAIIFLVLGILTFLILLGIGIYFYWSKCS

..**::* : .: : : * . :. . : :. : . : : :

gal PSYLDK-------------VIDLPPTHKPCSHEERAFSLPQKETI----IQKEHLALQSQ

mus EQRLQAA--------DEEEELEGPPSYKPPTPKA-KLEEPEMPSQLFTLGASEHSPVKTP

hum REVLWHCHLCPSSTEHASASANGHVSYSAVSRENSSSQDPQTE------GTR--------

pan REFLWHRHLCPSSTEHASASANGHVSYSAVSRENSSSQDPQTE------GTR--------

. * : ::. : : . *:

gal YGEKDTGNLQHML-----------------------------------------------

mus YFDAGVSCADQEMPRYHELPTLEEWSGPLLLGATGLGPSLLVPPGPNVVEGVSLSLEDEE

hum ------------------------------------------------------------

pan ------------------------------------------------------------

gal -----------------------------------------

mus EDDEEEDFLDKINPIYDALSYPSPSDSYQSKDFFVSRAMYV

hum -----------------------------------------

pan -----------------------------------------

1. Alpha-1-antitrypsin

gal MKS-ALYLCLFLTGLQVQALPKPNHSNKHKEERPHSLGDHPHVEHKNLAHMKIAPSNAEF

mus -----------LAGLCCMV-P--SFLAEDVQETDTSQ------KDQSPASHEIATNLGDF

hum MPSSVSWGILLLAGLCCLV-P--VSLAEDPQGDAAQKTDTSHHDQDHPTFNKITPNLAEF

pan MLSSVSWGILLLAGLCCLV-P--VSLAEDPQGDAAQKTDTSHHDQDHPTFNKITPNLAEF

*:** . * :. : . ... : :*: . .:*

gal AFRFYKQVTEAGGNKNIFFSPLSLSTAFAMLSLGARSNTLSQLHKCLTFNLTEMEEQEIH

mus AISLYRELVHQSNTSNIFFSPVSIATAFAMLSLGSKGDTHTQILEGLQFNLTQTSEADIH

hum AFSLYRQLAHQS**N**STNIFFSPVSIATAFAMLSLGTKADTHDEILEGLNF**N**LTEIPEAQIH

pan AFSLYRQLAHQSNSTNIFFSPVSIATAFAMLSLGTKADTHDEILEGLNFNLTEIPEAQIH

*: :*:::.. ....******:*::*********::.:* :: : * ****: * :**

gal EGFQRLLQLLNDSQRDIQLNMGNTLFIDERLKLQQKFLDDVTNFYYSEAVSMDFQNSEHA

mus KSFQHLLQTLNRPDSELQLSTGNGLFVNNDLKLVEKFLEEAKNHYQAEVFSVNFAESEEA

hum EGFQELLRTLNQPDSQLQLTTGNGLFLSEGLKLVDKFLEDVKKLYHSEAFTVNFGDTEEA

pan EGFQELLRTLNQPDSQLQLTTGNGLFLSEGLKLVDKFLEDVKKLYHSEAFTVNFGDTEEA

:.**.**: ** : ::**. ** **:.: *** :***::..: * :*..:::* ::*.*

gal KEEINNYIKAKTHGKFLDLLDSIGKDVVMILTNYVYFKGYWEEPFESYNTRDDDFFVDAK

mus KKVINDFVEKGTQGKIVEAVKELDQDTVFALANYILFKGKWKKPFDPENTEEAEFHVDKS

hum KKQINDYVEKGTQGKIVDLVKELDRDTVFALVNYIFFKGKWERPFEVKDTEEEDFHVDQV

pan KKQINDYVEKGTQGKIVDLVKELDRDTVFALVNYIFFKGKWERPFEVKDTEEEDFHVDQA

*: **:::: *:**::: :..:.:*.*: *.**: *** *:.**: :*.: :*.**

gal HSVKVKMMYKNTYYNIHRDEQLSCWVVEIPYRGNAAAFFVLPDEGSMNQVEDALLQDTVS

mus TTVKVPMMMLSGMLDVHHCSILSSWVLLMDYAGNASAVFLLPEDGKMQHLEQTLNKELIS

hum TTVKVPMMKRLGMFNIQHCKKLSSWVLLMKYLG**N**ATAIFFLPDEGKLQHLENELTHDIIT

pan TTVKVPMMKRLGMFNIQHCKKLSSWVLLMKYLGNATAIFFLPDEGKLQHLENELTHDIIT

:*** ** :::: . **.**: : * ***:*.*.**::*.::::*: * :: ::

gal NWSQSLEGRSIDLYLPKFSISGSYDVKKLFLKMGVTDMFSNNADFSGVAK-NTLLKVSRA

mus KILLNRRRRLVQIHIPRLSISGDYNLKTLMSPLGITRIFNNGADLSGITEENAPLKLSKA

hum KFLENEDRRSASLHLPKLSITGTYDLKSVLGQLGITKVFSNGADLSGVTE-EAPLKLSKA

pan KFLENEDRRSASLHLPKLSITGTYDLKSVLGQLGITKVFSNGADLSGVTE-EAPLKLSKA

: . * .:::*::**:* *::*.:: :*:* :*.*.**:**::: :: **:*:*

gal IHKAKLNVNENGTEAAAVTMVEMKVFSAMIDPLEIKFNRPFVMMIFDKITNSILFMGKVV

mus VHKAVLTIDETGTEAAAATVFEAV---PMSMPPILRFDHPFLFIIFEEHTQSPIFVGKVV

hum VHKAVLTIDEKGTEAAGAMFLEAI---PMSIPPEVKFNKPFVFLMIEQNTKSPLFMGKVV

pan VHKAVLTIDEKGTEAAGAMFLEAI---PMSIPPEVKFNKPFVFLMIEQNTKSPLFVGKVV

:*** *.::*.*****.. ..* * * ::*::**::::::: *:* :*:****

gal NPVAKED

mus DPTHK--

hum NPTQK--

pan NPTQK--

:*. *

1. Pigment epithelium factor

gal MQIPAVLLLLGLLTIPSKSQ--NSPAGQNSPTTDGTVGEVEEEDPFYKTPINKLAAAVSN

hum MQALVLLLCIGALLGHSSCQNPASPPEEGSPDPDSTGALVEEEDPFFKVPVNKLAAAVSN

pan MQALVLLLCIGALLGHSSCQNPASPPEEGSPDPDSTGALVEEEDPFFKVPVNKLAAAVSN

mus MQALVLLLWTGALLGHGSSQNVPS-SSEGSPVPDSTGEPVEEEDPFFKVPVNKLAAAVSN

** .:** * * ...* * :.** *.* *******:*.*:*********

gal FGYDLYRQQSSRTATANVLLSPFSLATALSGLSLGAGERTEDVISRALFYDLLNKAEVHN

hum FGYDLYRVRSSTSPTTNVLLSPLSVATALSALSLGAEQRTESIIHRALYYDLISSPDIHG

pan FGYDLYRVRSSMSPTTNVLLSPLSVATALSALSLGAEQRTESIIHRALYYDLISSPDIHG

mus FGYDLYRLRSSASPTGNVLLSPLSVATALSALSLGAEHRTESVIHRALYYDLITNPDIHS

******* :** : * ******:*:*****.***** .***.:* ***:***:.. ::*.

gal TYKDLLASVTGPEKSLKSASRIIVEKRLRVKSTFHSQLEKSYRMRLRALSGNTQLDLQEI

hum TYKELLDTVTAPQKNLKSASRIVFEKKLRIKSSFVAPLEKSYGTRPRVLTGNPRLDLQEI

pan TYKELLDTVTAPQKNLKSASRIVFEKKLRIKSSFVAPLEKSYGTRPRVLTGNPRLDLQEI

mus TYKELLASVTAPEKNLKSASRIVFERKLRVKSSFVAPLEKSYGTRPRILTGNPRVDLQEI

***:** :**.*:*.*******:.*::**:**:* : ***** * * *:** ::*****

gal NNWVRQQTRGRILRFMKDMPTDVSILLAGAAYFKGTWKTKFDTKRTVLKDFHLDEDRTVQ

hum NNWVQAQMKGKLARSTKEIPDEISILLLGVAHFKGQWVTKFDSRKTSLEDFYLDEERTVR

pan NNWVQAQMKGKLARSTKEIPDEISILLLGVAHFKGQWVTKFDSRKTSLEDFHLDEERTVR

mus NNWVQAQMKGKIARSTREMPSALSILLLGVAYFKGQWVTKFDSRKTTLQDFHLDEDRTVR

****: * :*:: * :::* :**** *.*:*** * ****:::* *:**:***:***:

gal VSMMSDPKAILRYGFDSELNCKIAQLPLTEGVSAMFFLPTKVTQNMTLIEESLTSEFVHD

hum VPMMSDPKAVLRYGLDSDLSCKIAQLPLTGSMSIIFFLPLKVTQ**N**LTLIEESLTSEFIHD

pan VPMMSDPKAVLRYGLDSDLSCKIAQLPLTGSTSIIFFLPLKVTQNLTLIEESLTSEFIHD

mus VPMMSDPKAILRYGLDSDLNCKIAQLPLTGSMSIIFFLPLTVTQNLTMIEESLTSEFIHD

* *******:****:**:*.********* . * :**** .****:*:*********:**

gal VDRELKTVHAVLSLPKLKLNYEEALGNTVKETRLQSLFTSPDFTKISAKPIKLSHVQHKA

hum IDRELKTVQAVLTVPKLKLSYEGEVTKSLQEMKLQSLFDSPDFSKITGKPIKLTQVEHRA

pan IDRELKTVQAVLTVPKLKLSYEGEVTKSLQETKLQSLFDSPDFSKITGKPIKLTQVEHRA

mus IDRELKTIQAVLTVPKLKLSFEGELTKSLQDMKLQSLFESPDFSKITGKPVKLTQVEHRA

:******::***::*****.:* : ::::: :***** ****:**:.**:**::*:*:*

gal VLELNEDGEKSTPNPGVNAARLTFPIEYHVDRPFLLVLRDDTTGTLLFIGKILDPRSV

hum GFEWNEDGAGTTPSPGLQPAHLTFPLDYHLNQPFIFVLRDTDTGALLFIGKILDPRGP

pan GFEWNEDGAGTTPSPGLQPAHLTFPLDYHLNQPFIFVLRDTDTGALLFIGKILDPRGT

mus AFEWNEEGAGSSPSPGLQPVRLTFPLDYHLNQPFLFVLRDTDTGALLFIGRILDPSST

:* **:* ::*.**:: .:****::**:::**::**** **:*****:**** .

1. Trophoblast glycoprotein

gal MPGREAE----RRGALCLG-----------------------LLLHALLGCGSA--QPPA

hum MPGGCSRGPAAGDGRLRLARLALVLLGWVSSSSPTSSASSFSSSAPFLASAVSAQPPLPD

pan MPGGCSRGPAAGDGRLRLARLALVLLGWVSSSSPTSSASSFSSSAPFLASAVSAQPPLPD

mus MPGAGSRGPSAGDGRLRLARLALVLLGWVSASAPSSSVPSSSTSPAAFLASGSAQPPPAE

*** :. * * *. : .. **

gal ACPAPCECSEAAKTVKCVNKNLTEVPPDLPPYVRNLFITGNRLGRLPAGALS-APRLAEL

hum QCPALCECSEAARTVKCVNR**N**LTEVPTDLPAYVRNLFLTGNQLAVLPAGAFARRPPLAEL

pan QCPALCECSEAARTVKCVNRNLTEVPTDLPAYVRNLFLTGNQLAVLPAGAFARRPPLAEL

mus RCPAACECSEAARTVKCVNRNLLEVPADLPPYVRNLFLTGNQMTVLPAGAFARQPPLADL

*** *******:******:** *** *** ******:***:: *****:: * **:*

gal GSLNLSGNHLRAVEAGALAALPALRQLDLGGNPLAELSPLAFGRA-------SPLEELAL

hum AAL**N**LSGSRLDEVRAGAFEHLPSLRQLDLSHNPLADLSPFAFSGSNASVSAPSPLVELIL

pan AALNLSGSRLDEVRAGAFEHLPSLRQLDLSHNPLADLSPFAFSGSNASVSAPSPLVELIL

mus EALNLSGNHLKEVCAGAFEHLPGLRRLDLSHNPLTNLSAFAFAGSNASVSAPSPLEELIL

:*****.:* * ***: **.**:***. ***::** :**. : *** ** *

gal RGAL-----REQGALLG--------LADLLQAGALRNLSRLELADNGLLLLPTGMLGALP

hum NHIVPPEDERQNRSFEGM------VVAALLAGRALQGLRRLELASNHFLYLPRDVLAQLP

pan NHIVPPEDEQQNRSFEGM------VVAALLAGRALQGLRRLELASNHFLYLPRDVLAQLP

mus NHIVPPEDQRQNGSFEGMVAFEGMVAAALRSGLALRGLTRLELASNHFLFLPRDLLAQLP

. : ::: :: * * * . **:.* *****.* :* ** .:*. **

gal ALRHLDLSNNSLVGLRNVSFQGLVRLQSLNLSDNSLGVLRNGTLAQWRGLPALRRISLSH

hum SLRHLDLSNNSLVSLTYVSFRNLTHLESLHLEDNALKVLH**N**GTLAELQGLPHIR-VFLDN

pan SLRYLDLSNNSLVSLTYVSFRNLTHLESLHLEDNALKVLHNGTLAELQGLPHIR-VFLDN

mus SLRYLDLRNNSLVSLTYASFRNLTHLESLHLEDNALKVLHNSTLAEWQGLAHVK-VFLDN

:**:*** *****.* .**:.*.:*:**:*.**:* **:*.***: :** :: : *.:

gal NTWVCDCAIEDMVAWLKESDQVEGKEALSCAFPEKMAGRALLKLNTSELNCSAPVDVPSQ

hum NPWVCDCHMADMVTWLKETEVVQGKDRLTCAYPEKMRNRVLLELNSADLDCDPI--LPPS

pan NPWVCDCHMADMVTWLKETEVVQGKDRLTCAYPEKMRNRVLLELNSADLDCDPI--LPPS

mus NPWVCDCYMADMVAWLKETEVVPDKARLTCAFPEKMRNRGLLDLNSSDLDCDAV--LPQS

* ***** : ***:****:: * .* *:**:**** .* **.**:::*:*. :* .

gal LQTSYVFLGIVLALIGAIFLLVLYLNRKGIKKWMHNIRDACRDHMEGYHYRYEINADPRL

hum LQTSYVFLGIVLALIGAIFLLVLYLNRKGIKKWMHNIRDACRDHMEGYHYRYEINADPRL

pan LQTSYVFLGIVLALIGAIFLLVLYLNRKGIKKWMHNIRDACRDHMEGYHYRYEINADPRL

mus LQTSYVFLGIVLALIGAIFLLVLYLNRKGIKKWMHNIRDACRDHMEGYHYRYEINADPRL

************************************************************

gal TNLSSNSDV

hum TNLSSNSDV

pan TNLSSNSDV

mus TNLSSNSDV

*********

1. Carboxypeptidase A4

gal MKLILIFSALFGAALCL-ETFVGHQVLRIKTRNEEQLKKLRFLETLDHLELDFWLNPSTP

hum MRWILFIGALIGSSICGQEKFFGDQVLRINVRNGDEISKLSQLVNSNNLKLNFWKSPSSF

pan MRWILFIGALIGSSICGQEKFFGDQVFRINVRNGDEISKLSQLVNSNNLKLNFWKSPSSF

mus MKWLLFFGALIGAGICGRDKFFGDQVFRINVRNGDEIRKLTELVNSDHLKLSVWKSPSTF

*: :*::.**:*:.:* :.*.*.**:**:.** ::: ** * . ::*:*..* .**:

gal ALPVDVRIPAHSLQAVKIFLESHGIEYSILIEDLQVILDREKQDIVASQQMERSSSTFNY

hum NRPVDVLVPSVSLQAFKSFLRSQGLEYAVTIEDLQALLDNEDDEMQHNEGQERSSNNFNY

pan NRPVDVLVPSVSLQAFKSFLRSQGLEYAVTIKDLQALLDNEDDEMQHNEGQERSSNNFNY

mus DRPVDILVPSVSLLPVKSFLKSQGLDYSVTIEDLQALLDNEDEEMQHNEGIERS-GDFNY

***: :*: ** .* **.*:*::*:: *:***.:**.*.::: .: *** . ***

gal GTYHSLASIYQELDNLASEYGNIVSKIQIGESYEKRPLYVLKFSTGRGN-RPAIWLDAGI

hum GAYHSLEAIYHEMDNIAADFPDLARRVKIGHSFENRPMYVLKFSTGKGVRRPAVWLNAGI

pan GAYHSLEAIYHEMDNIAADFPDLARRVKIGHSFENRPMYVLKFSTGKGARRPAVWLNAGI

mus GAYHPLEAIYHEMDSIATDFPELVSRVKIGETFEKRPMYVLKFSTGGGKKRPAIWLNAGI

*:** * :**:*:*.:*::: ::. :::**.::*:**:******** * ***:**:***

gal HSREWVTQASAMWIARKIASDYGNDPSITSLLNNLDIFLLPVANPDGYEFTHTTNRMWRK

hum HSREWISQATAIWTARKIVSDYQRDPAITSILEKMDIFLLPVANPDGYVYTQTQNRLWRK

pan HSREWISQATAIWTARKIVSDYQRDPAITSILEKMDIFLLPVANPDGYVYTQTQNRLWRK

mus HAREWISQATAIWTARKIVTDYKKDPAITSILKKVDIFLLPVANPDGYVYTQSQNRLWRK

*:***::**:*:* ****.:** .**:***:*:::************* :*:: **:***

gal TRSKNSGSLCIGVDPNRNWDAGFGGPGASSNPCSDSYRGPRANSEVEVKSVVDFIKNHGN

hum TRSRNPGSSCIGADPNRNW**N**ASFAGKGASDNPCSEVYHGPHANSEVEVKSVVDFIQKHGN

pan TRSRNPGSSCIGADPNRNWNASFAGKGASDNPCSEVYHGPHANSEVEVKSVVDFIQKHGN

mus TRSRNPGSRCVGADPNRNWNASFAGEGTSDNPCSEVYHGSHPNSEVEVKSVVDFIQKHGN

***:* ** *:*.******:*.*.* *:*.****: *:* : *************::***

gal IQAFLTLHSYSQLLMYPYGYKCTPPADNTELNTIGKAAANSIRSLYGTTYTVGSICSTIY

hum FKGFIDLHSYSQLLMYPYGYSVKKAPDAEELDKVARLAAKALASVSGTEYQVGPTCTTVY

pan FKCFIDLHSYSQLLMYPYGYSVKKAPDAEELDKVARLAAKALASVSGTEYQVGPTCTTVY

mus FKCFIDLHSYSQLLMYPYGYTVKKAPDAEELDDVARNAAQALASLSGTKYRVGPTCTTVY

:: *: **************. . * **: :.: **::: *: ** * ** *:*:*

gal QASGGSIDWSYDYGIKYSFAFELRDTGRYGFLLPAAQIIPTAEETWLGLKTIMEYVRDNP

hum PASGSSIDWAYDNGIKFAFTFELRDTGTYGFLLPANQIIPTAEETWLGLKTIMEHVRDNL

pan PASGSSIDWAYDNGIKFAFTFELRDTGTYGFLLPANQIIPTAEETWLGLKTIMEHVRDNF

mus PASGSSVDWAYDNGIKYAFTFELRDTGYYGFLLPASQIIPTAEETWLGLKTIMEHVRDHL

***.*:**:** ***::*:******* ******* ******************:***:

gal Y

hum Y

pan Y

mus Y

*

1. Carboxypeptidase B

gal --MMYLLFFTLFVWIQEKHAFILPRDEVLWAFPKTDKQAEDLQSFLNTTEVILWQPVVVE

mus MKLHGLGILVAIIL-YEQHGFAFQSGQVLSALPRTSRQVQLLQNLTTTYEVVLWQPVTAE

hum MKLCSLAVLVPIVLFCEQHVFAFQSGQVLAALPRTSRQVQVLQ**N**LTTTYEIVLWQPVTAD

pan MKLCSLAVLVPIVLFCEQHVFAFQSGQVLAALPRTSRQVQVLQNLTTTYEIVLWQPVTAD

: * .:. :: *:* * : .:** *:*:*.:*.: **.: .* *::*****..:

gal NIQKDQEVHFYVSASNTNRIKAGLRQLTISHKVLMRDVQGLIEKQTLNDTANPRSSSSYY

mus FIEKKKEVHFFVNASDVDSVKAHLNVSRIPFNVLMNNVEDLIEQQTFNDTVSPRASASYY

hum LIVKKKQVHFFV**N**ASDVDNVKAHL**N**VSGIPCSVLLADVEDLIQQQIS**N**DTVSPRASASYY

pan LIVKKKQVHFFVNASDVDNVKAHLNVSGIPCSVLLADVEDLIQQQISNDTVSPRASASYY

* *.::***:*.**:.: :** *. * .**: :*:.**::* ***..**:*:***

gal ENYHSMTEIYHWMEEIVRVHSDLLEKIYIGSSYEKRPLYVLKLSKRQGNPKSAIWIDCGI

mus EQYHSLNEIYSWIEVITEQHPDMLQKIYIGSSFEKYPLYVLKVSGKEQRIKNAIWIDCGI

hum EQYHSLNEIYSWIEFITERHPDMLTKIHIGSSFEKYPLYVLKVSGKEQAAKNAIWIDCGI

pan EQYHSLNEIYSWIEFITERHPDMLTKIHIGSSFEKNPLYVLKVSGKEQTAKNAIWIDCGI

*:***:.*** *:* *.. * *:* **:****:** ******:* :: *.********

gal HAREWISPAFCLWFIGHAIHLRERDQIMTTLLEHFDFYVMPVMNVDGYEYTWSKPSNRLW

mus HAREWISPAFCLWFIGYVTQFHGKENLYTRLLRHVDFYIMPVMNVDGYDYTWKK--NRMW

hum HAREWISPAFCLWFIGHITQFYGIIGQYTNLLRLVDFYVMPVVNVDGYDYSWKK--NRMW

pan HAREWISPAFCLWFIGHITQFYGIIGQYTNLLRLVDFYVMPVVNVDGYDYSWKK--NRMW

****************: :: * **. .***:***:*****:*:*.* **:*

gal RKSRSSHSNSGCIGTDMNRNFD-AHWCGRGASPNECYETYCGPYPESEPEVKAVARFIRD

mus RKNRSAHKNNRCVGTDLNRNFASKHWCEKGASSSSCSETYCGLYPESEPEVKAVADFLRR

hum RK**N**RSFYANNHCIGTDLNRNFASKHWCEEGASSSSCSETYCGLYPESEPEVKAVASFLRR

pan RKNRSFYANNHCIGTDLNRNFASKHWCEEGASSSSCSETYCGLYPESEPEVKAVASFLRR

**.** : *. *:***:**** *** .*** ..* ***** ************ *:*

gal HKDIIKAYITMHSYSQLVLFPYSYTTDKSKDHDELESLAKKAANAIRRTT-KKIYTPGPG

mus NIDHIKAYISMHSYSQQILFPYSYNRSKSKDHEELSLVASEAVRAIESINKNTRYTHGSG

hum NINQIKAYISMHSYSQHIVFPYSYTRSKSKDHEELSLVASEAVRAIEKISKNTRYTHGHG

pan NINHIKAYISMHSYSQHIVFPYSYTRSKSKDHEELSLVASEAVRAIEKTSKNTRYTHGHG

: : *****:****** ::*****. .*****:**. :*.:*..**. . :. ** * *

gal ARTIYLAPGGSDDWAYDLGIKYSFTIELRDTGTYGFLLPSAQIRPTCIEALSAVREIARH

mus SESLYLAPGGSDDWIYDLGIKYSFTIELRDTGRYGFLLPERYIKPTCAEALAAISKIVWH

hum SETLYLAPGGGDDWIYDLGIKYSFTIELRDTGTYGFLLPERYIKPTCREAFAAVSKIAWH

pan SETLYLAPGGGDDWIYDLGIKYSFTIELRDTGTYGFLLPERYIKPTCREAFAAVSKIAWH

:.::******.*** ***************** ******. *:*** **::*: :*. *

gal VLQNL

mus VIRNT

hum VIRNV

pan VIRNV

*::*

1. Alpha-1-antichymotrypsin

gal ----------------------------MKPTFSLCFLLAGLYSVAQCHQRPRYHNKQDN

mus ----------------------------MAFIVALGLVITGICPGVLCFPDGTLERDTLF

hum -------------------------MERMLPLLALGLLAAGFCPAVLCHPNSPLDEE**N**LT

pan MKIHYSRQTALESTSYIQLPEAELRMERMLPLLALGLLVAGLCPAVLCHPNSPLDEENPT

* .:* :: :*: . *. ...

gal SKGAYYWGSSSHREGVFPNKNKTFVKVVHSNADFALSFYKLVASEATDQNIFFSPISIST

mus HKD------------KENGTQLDSLTLASINTDFAFSLYKKLALKNPDTNIVFSPLSISA

hum QEN------------QDRGTHVD-LGLASANVDFAFSLYKQLVLKAPDKNVIFSPLSIST

pan QEN------------QDRGTHVD-LGLASANVDFAFSLYKQLVLKAPDKNVIFSPLSIST

:. ..: : :. *.***:*:** :. : * *:.***:***:

gal SLAMLALGAKSVTLTQILEGLAFNLKKTQDQEIHEGFCQLLHMLNRSDSDLHLSLGNTLF

mus ALAIVSLGAKGNTLEEILEGLNFNLTETPEADIHQGFGHLLQRLSHPGEQVQISTGSALF

hum ALAFLSLGAH**N**TTLTEILKGLKF**N**LTETSEAEIHQSFQHLLRTL**N**QSSDELQLSMGNAMF

pan ALAFLSLGAHNTTLTEILKGLKFNLTETSEAEIHQSFQHLLRTLNQSSDELQLNMGNAMF

:**:::***:. ** :**:** ***.:* : :**:.* :**: *.: ..::::. *.::*

gal IEETLKPLQKFLDDAKSFYQSEVLSADFNNSSGAENQINSYIEEKTNGKIVKLVENLDPL

mus VEKHLQILAEFQEKARALYQAEAFTADFQQPLEATKLINDYVSNQTQGKIKGLISDLDTD

hum VKEQLSLLDRFTEDAKRLYGSEAFATDFQDSAAAKKLINDYVK**N**GTRGKITDLIKDLDSQ

pan VEEQLSLLDRFTEDAKRLYGSEAFATDFQDSAAAKKLINDYVKNRTRGKITDLIKDLDSQ

::: *. * .* :.*: :* :*.:::**:: * : **.*:.: *.*** *:.:**

gal TAMVLVNYVFFKAHWEKPFSDSYTKKEDFFVDKKTSVKVDMMYRKGYYRNYF-DEELSCW

mus TLMVLVNYIYFKGKWKMPFNPRDTFESEFYLDVKRSVKVPMMKIKTLTTPYFRDEELSCT

hum TMMVLVNYIFFKAKWEMPFDPQDTHQSRFYLSKKKWVMVPMMSLHHLTIPYFRDEELSCT

pan TMMVLVNYIFFKAKWEMPFDPQDTHQSRFYLSEKKWVMVPMMSLHHLTIPYFRDEELSCT

* ******::**.:*: **. * :. *::. * * * ** : ** ******

gal LVQIPYNGNAAALFVLPDEGKMKQVEDALLKRTVSKWEKLLQHRKI-HLHIPKLSISGTY

mus VVELKYKGNASALFILPDQGRMQQVEASLQPETLRKWKNSLRPRKMGELYLPKFSISTDY

hum VVELKYTG**N**ASALFILPDQDKMEEVEAMLLPETLKRWRDSLEFREIGELYLPKFSISRDY

pan VVELKYTGNASALFILPDQDKMEEVEAMLLPETLKRWRDSLEFREIGELYLPKFSISRDY

:*:: *.***:***:***:.:*::** * .*: :*.. *. *:: .*::**:*** *

gal DVKKIVREVGIIDLFTAQADLSGITEDPGLMVSKVIHRAVLNVHENGTEAAGVTVTEITW

mus SLKNILPELGIKEIFSNQADLSGITGTKDLIVSQMVHKVVLDVAETGTEGVAATGVNFRI

hum NLNDILLQLGIEEAFTSKADLSGITGARNLAVSQVVHKAVLDVFEEGTEASAATAVKITL

pan NLKDILLQLGIEEAFTSKADLSGITGARNLVVSQVVHKAVLDVFEEGTEASAATAVRITL

.::.*: ::** : *: :******* .* **:::*:.**:* * ***. ..* ..:

gal RSGDFPRPPRVRFNRPFLLMILDKYAHTILFIGKIVNPLKNN

mus L----SRRTSLWFNRPFLMVISHTDVQTTLFIAKITHPKRA-

hum LSALVETRTIVRFNRPFLMIIVPTDTQNIFFMSKVTNPKQA-

pan LSALVETRTIVRFNRHFLMIIVPTDTQNIFFMSKVTNPKQA-

: *** **::* . .:. :*:.*:.:* :

1. Lysosomal acid phosphatase

gal MAAGRRAGA------VLLLLAAWLQLPPVRPRSLRFATLVYRHGDRSPIKAYPRDPFQES

hum -MAGKRSGWSRAALLQLLLGVNLVVMPPTRARSLRFVTLLYRHGDRSPVKTYPKDPYQEE

pan -MAGKRSGWSRAALLQLLLGVNLVVMPPTQARSLRFVTLLYRHGDRSPVKTYPKDPYQEE

mus -MAGRQTGWSQAALLQFLLGMCLTVMPPIQARSLRFVTLLYRHGDRSPVKTYPKDPYQEE

**:::* :** :** : *****.**:********:*:**:**:**.

gal AWPQGFGQLMQVGMRQQWELGQALRRRYHGFLSASYRRQEIFIRSTDYDRTLMSAEANLA

hum EWPQGFGQLTKEGMLQHWELGQALRQRYHGFL**N**TSYHRQEVYVRSTDFDRTLMSAEANLA

pan EWPQGFGQLTKEGMLQHWELGQALRQRYHGFLNTSYHRQEVYVRSTDFDRTLMSAEANLA

mus KWPQGFGQLTKEGMLQHWELGQALRQRYHGFLNTSYHRQEVYVRSTDFDRTLMSAEANLA

******** : ** *:********:******.:**:***:::****:************

gal GLYPPEEQQMFNPNISWQPIPVHTVPESGEMLLKFPLTPCPRYEQLQNETRNSAEYINKT

hum GLFPPNGMQRFNP**N**ISWQPIPVHTVPITEDRLLKFPLGPCPRYEQLQ**N**ETRQTPEYQ**N**ES

pan GLFPPNGMQRFNPNISWQPIPVHTVPITEDRLLKFPLGPCPRYEQLQNETRQTPEYQNES

mus GLFPPNEVQHFNPNISWQPIPVHTVPITEDRLLKFPLGPCPRYEQLQNETRQTPEYQNRS

**:**: * **************** : : ****** *************:: ** *.:

gal RDNLQFLQMVANETGIRDLSLESVWSVYDTLFCEQAHKMDLPSWVTPDVMTQMKQLKDFG

hum SRNAQFLDMVA**N**ETGLTDLTLETVWNVYDTLFCEQTHGLRLPPWASPQTMQRLSRLKDFS

pan SRNAQFLDMVANETGLTDLTLETVWNVYDTLFCEQTHGLRLPPWASPQTMQRLSRLKDFS

mus IQNAQFLNMVANETGLTNVTLETIWNVYDTLFCEQTHGLLLPPWASPQTVQRLSQLKDFS

* ***:*******: :::**::*.*********:* : ** *.:*:.: ::.:****.

gal FEFLFGIHHRVEKARLQGGVLLDHIRKNLTKAANASAHQQLKLLVYSAHDTTLVALQMAL

hum FRFLFGIYQQAEKARLQGGVLLAQIRK**N**LTLMATT--SQLPKLLVYSAHDTTLVALQMAL

pan FRFLFGIYQQAEKARLQGGVLLAQIRKNLTLMATT--SQLPKLLVYSAHDTTLVALQMAL

mus FLFLFGIHEQVQKARLQGGVLLAQILKNLTLMATT--SQFPKLLVYSAHDTTLVALQMAL

* *****:.:.:********** :* **** *.: * *******************

gal DVYNKIQAPYASCHLFELYQEDDGNFSVEMFFRNESGKEPFPLTIPGCQQICPLQRFLEL

hum DVYNGEQAPYASCHIFELYQEDSG**N**FSVEMYFR**N**ESDKAPWPLSLPGCPHRCPLQDFLRL

pan DVYNGEQAPYASCHIFELYQEDSGNFSVEMYFRNESDKAPWPLSLPGCPHCCPLQDFLRL

mus NVYNGKQAPYASCHIFELYQEDNGNFSVEMYFRNDSKKAPWPLILPGCPHRCPLQDFLRL

:*** ********:*******.*******:***:* * *:** :*** : **** **.*

gal TDPVVPQDWEQECQIASSMHDTGLFVGLAVCGSILLLLIILLLTVLFRIQSQPPGYRHVS

hum TEPVVPKDWQQECQLASGPADTEVIVALAVCGSILFLLIVLLLTVLFRMQAQPPGYRHVA

pan TEPVVPKDWQQECQLASGPADTEVIVALAVCGSILFLLIVLLLTVLFRMQAQPPGYRHVA

mus TEPVIPKDWQKECQLANDTADTEVIVALAVCGSILFLLIVLLLTILFRMQAQPPGYHHVA

*:**:*:**::***:*.. ** ::*.********:***:****:***:*:*****:**:

gal NEGEEQA

hum DGEDHA-

pan DGEDHA-

mus DREDHA-

: :.

1. Zona pellucida sperm-binding protein

gal MGSGGSLGVVLLCWMLAEAASYSPWDFSPRDSGVLRVRGDSSWRPPQVPSFSQPSPWAWV

hum MELSYRL---FICLLLW--------------------------GST---ELCYPQPLWLL

pan MELSYRL---FICLLLW--------------------------GST---ELCYPQPLWLL

mus MASSYFL---FLCLLLC--------------------------GGP---ELCNSQTLWLL

* . * ::* :* .:. . :

gal DVSQLQAASPLHPVSVWCQEAQVVVTVHRDLFGTGRLVRAADLTLGTAACPATAQNAAEN

hum QGGASHPETSVQPVLVECQEATLMVMVSKDLFGTGKLIRAADLTLGPEACEPLVSMDTED

pan QGGASRPETSVQPVLVECQEATLMVMVSKDLFGTGKLIRAADLTLGPEACEPLVSMDTED

mus PGGTPTPVGSSSPVKVECLEAELVVTVSRDLFGTGKLVQPGDLTLGSEGCQPRVSVDT-D

. ** * * ** ::* * :******:*:: .***** .* .. : :

gal VVTFTAGLHECGSTLQMTPDSLIYKTSLFYKPTPVGNMVIVRTSPAVVPIECHYPRRSNV

hum VVRFEVGLHECGNSMQVTDDALVYSTFLLHDPRPVG**N**LSIVRTNRAEIPIECRYPRQG**N**V

pan VVRFEVGLHECGNSMQVTDDALVYSTFLLHDPRPVGNLSIVRTNRAEIPIECRYPRQGNV

mus VVRFNAQLHECSSRVQMTKDALVYSTFLLHDPRPVSGLSILRTNRVEVPIECRYPRQGNV

** * . ****.. :*:* *:*:*.* *::.* **..: *:**. . :****:***:.**

gal SSNAVRPTWVPFHSTLSMEEKLVFSLRLMSDDWRTERLSNGVQLGESLHLQADVMAGNHI

hum SSQAILPTWLPFRTTVFSEEKLTFSLRLMEENWNAEKRSPTFHLGDAAHLQAEIHTGSHV

pan SSQAILPTWLPFRTTVFSEEKLTFSLRLMEENWNAEKTSPTFHLGDAAHLQAEIHTGSHV

mus SSHPIQPTWVPFRATVSSEEKLAFSLRLMEENWNTEKSAPTFHLGEVAHLQAEVQTGSHL

**: : ***:**::*: ****.******.::*.:*: : .:**: ****:: :*.*:

gal PLRLFVDDCVATLS--PDRNSSPRYALIDLSGCLVDGRTDDATSAFISPRPRQETLQFMV

hum PLRLFVDHCVATPT--PDQ**N**ASPYHTIVDFHGCLVDGLTD-ASSAFKVPRPGPDTLQFTV

pan PLRLFVDHCVATPT--PDQNASPYHTIVDFHGCLVDGLTD-ASSAFKVPRPGPDTLQFTV

mus PLQLFVDHCVATPSPLPDPNSSPYHFIVDFHGCLVDGLSE-SFSAFQVPRPRPETLQFTV

**:****.**** : ** *:** : ::*: ****** :: : *** *** :**** *

gal DAFKFAGDDRNLIYITCHLKVSPADQAPNPLNKACSFNKASSLWAPVEGTGDICSCCETG

hum DVFHFA**N**DSRNMIYITCHLKVTLAEQDPDELNKACSFSKPSNSWFPVEGSADICQCCNKG

pan DVFHFANDSRNMIYITCHLKVTLAEQDPDELNKACSFSKPSNSWFPVEGPADICQCCNKG

mus DVFHFANSSRNTLYITCHLKVAPANQIPDKLNKACSFNKTSQSWLPVEGDADICDCCSHG

*.*:**...** :********: *:* *: *******.* *. * **** .***.**. *

gal NCQLYGGYSRRSNPLSRWSGRRLKRDTSSRQDASLRRAEAEVSVGPLLILDPAQGL----

hum DCGTPSHSRRQPHVMSQWSRSASRN-------RRHVTEEADVTVGPLIFLDRRGDHEVEQ

pan DCGTPSHSRRQPHVVSQWSRSASRN-------RRHVTEEADVTVGPLIFLDRSGDHEVEQ

mus NCSNSSSSQFQIHGPRQWSKLVSRN-------RRHVTDEADVTVGPLIFLGKANDQTVEG

:* . : : :** :. **:*:****::*. .

gal WSSSGRRVPAGKAPQGAAGGLPMPVQVAMIIAAVVLSLTALGIFLVCKRYSRPPSVSL

hum WALPSD-----TS--VVLLGVGLAVVVSLTLTAVILVL-----TRRCRTASHPVSASE

pan WALPSD-----TS--VVLLGVGLAVVVSLTLTAVILVL-----TRRCRTASHPVSASE

mus WTASAQ-----TS---VALGLGLATVAFLTLAAIVLAV-----TRKCHSSSYLVSLPQ

*: . .: . *: : . . : ::*::* : *: * *

1. Pannexin-1

gal MAIAHIATEYVFSDFLLKEPPETRYKGLRLELALDKIVTCIAVGLPLLLISLAFAQEVSI

mus MAIAHLATEYVFSDFLLKEPTEPKFKGLRLELAVDKMVTCIAVGLPLLLISLAFAQEISI

hum MAIAQLATEYVFSDFLLKEPTEPKFKGLRLELAVDKMVTCIAVGLPLLLISLAFAQEISI

pan MAIAHLATEYVFSDFLLKEPTEPKFKGLRLELAVDKMVTCIAVGLPLLLISLAFAQEISI

****::************** * ::********:**:********************:**

gal GAQISCFAPSSFSWRQAAYVDSYCWAAVQQKQPAYNNPENIPLWLHKFFPYILLLVAILL

mus GTQISCFSPSSFSWRQAAFVDSYCWAAVQQKSSLQSESGNLPLWLHKFFPYILLLFAILL

hum GTQISCFSPSSFSWRQAAFVDSYCWAAVQQKNSLQSESGNLPLWLHKFFPYILLLFAILL

pan GTQISCFSPSSFSWRQAAFVDSYCWAAVQQKNSLQSESGNLPLWLHKFFPYILLLFAILL

*:*****:**********:************. .: *:**************.****

gal YLPCLFWRFTAAPHLSSDLKFIMEELDKAYNRAIKAANSVRSGDPRDPADSIPAANENLT

mus YLPALFWRFSAAPHLCSDLKFIMEELDKVYNRAIKAAKSARDLDLRDGP-GPPGVTENVG

hum YLPPLFWRFAAAPHICSDLKFIMEELDKVYNRAIKAAKSARDLDMRDGACSVPGVTENLG

pan YLPPLFWRFAAAPHICSDLKFIMEELDKVYNRAIKAAKSVRDLDMRDGACSVPGVTENLG

*** *****:****:.************.********:*.*. * ** . *...**:

gal QSLWEISDSHFKYPIVEQYLKTKKNSKCLIIKYIFCRLLTLVIIFIACLYLGYYISLSSL

mus QSLWEISESHFKYPIVEQYLKTKKNSSHLIMKYISCRLVTFVVILLACIYLSYYFSLSSL

hum QSLWEVSESHFKYPIVEQYLKTKKNSNNLIIKYISCRLLTLIIILLACIYLGYYFSLSSL

pan QSLWEVSDSHFKYPIVEQYLKTKKNSNNLIIKYISCRLLTLIIILLACIYLGYYFSLSSL

*****:*:******************. **:*** ***:*:::*::**:**.**:*****

gal SDEFLCTIKTGILRNDTTVPEVVQCKLITVGVFKVLSYINLIVYLLVMPLVVYAMFVPFR

mus SDEFLCSIKSGVLKNDSTIPDRFQCKLIAVGIFQLLSLINLIVYALLIPVVVYTFFIPFR

hum SDEFVCSIKSGILR**N**DSTVPDQFQCKLIAVGIFQLLSVINLVVYVLLAPVVVYTLFVPFR

pan SDEFVCSIKSGILRNDSTVPDQFQCKLIAVGIFQLLSVINLVVYVLLAPVVVYTLFVPFR

****:*:**:*:*:**:*:*: .*****:**:*::** ***:** *: *:***::*:***

gal WNSGILKVYEILPTFDVLKLKSKSLDDLSIYLLFLEENVSELKSYKCLKVLENIAVS-EK

mus QKTDILKVYEILPTFDVLHFKSEGYNDLSLYNLFLEENISELKSYKCLKVLENIKSNGQG

hum QKTDVLKVYEILPTFDVLHFKSEGYNDLSLYNLFLEENISEVKSYKCLKVLENIKSSGQG

pan QKTDVLKVYEILPTFDVMHFKSEGYNDLSLYNLFLEENISEVKSYKCLKVLENIKSSGQG

::.:************:::**:. :***:* ******:**:************ . :

gal FDVMQLLINLGTIKTDTVDGKPGTAVPGKPEETAVEELEKDATELQVLADRDASGHVSPK

mus IDPMLLLTNLGMIKMDIIDGKIPTSLQTKGEDQGSQRVEFKDLDL-------SSEAAANN

hum IDPMLLLTNLGMIKMDVVDGKTPMSAEM-REEQGNQTAELQGMNI-------DSETKANN

pan IDPMLLLTNLGMIKMDVVDGKTPMSAEM-REEQGNQTAELQAMNI-------DSETKANN

:* * ** *** ** * :*** : *: . : * . :: * : :

gal EDKKLRQRLIDSSC

mus GEKNSRQRLLNPSC

hum GEKNARQRLLDSSC

pan GEKNARQRLLDSSC

:*: ****:: **

1. Kallistatin

gal MKTVFYICLLL----AGLHAFAYGQLTASHHNGHNPNEPKDHMHHNAEAAACLKLVPNNA

mus MAFIVALGLVITGICPGVLCFPDGTL--------ERDTLFHKDKENGTQLDSLTLASINT

hum MHLIDYLLLLL----VGLLALSHGQLHVEHDGESCS**N**SSHQQILETGEGSPSLKIAPANA

pan MHLIDYLLLLL----VGLLALSHGQLHVEHDGESCSNSSHQQILETGEGSPSLKIAPANA

* : : *:: *: .: * * : .: ... .*.:. *:

gal DFAFKFLNEVAQEAPNKNIFFSPVSISAAFAMLALGARSITKTQILEGLAFNLTEIQEKE

mus DFAFSLYKKLALKNPDTNIVFSPLSISAALAIVSLGAKGNTLEEILEGLNFNLTETPEAD

hum DFAFRFYYLIASETPGKNIFFSPLSISAAYAMLSLGACSHSRSQILEGLGF**N**LTELSESD

pan DFAFRFYYLIASETPGKNIFFSPLSISAAYAMLSLGACSHSRSQILEGLGFNLTELSESD

**** : :* : *..**.***:***** *:::*** . : :***** ***** * :

gal IHEGFHNLMHMLSHPESGVQLNMGNAIFLTKKLKPLKKFLDDAKPLYQLEVLATDFNNPT

mus IHQGFGHLLQRLSHPGEQVQISTGSALFVEKHLQILAEFQEKARALYQAEAFTADFQQPL

hum VHRGFQHLLHTLNLPGHGLETRVGSALFLSHNLKFLAKFL**N**DTMAVYEAKLFHTNFYDTV

pan VHRGFQHLLHTLNLPGHGLETRVGSALFLSHNLKFLAKFLNDTTAVYEAKLFHTNFYDTV

:*.** :*:: *. * :: *.*:*: ::*: * :* :.: :*: : : ::* :

gal EAEKEINDYTEKKTQGKITNLVKEIDPQTVMLLASFVFFRGNWEKPFKPENTEEREFFVD

mus EATKLINDYVSNQTQGKIKGLISDLDTDTLMVLVNYIYFKGKWKMPFNPRDTFESEFYLD

hum GTIQLINDHVKKETRGKIVDLVSELKKDVLMVLVNYIYFKALWEKPFISSRTTPKDFYVD

pan GTIQLINDHVKKETRGKIVDLVSELKKDVLMVLVNYIYFKALWEKPFISSRTTPKDFYVD

: : ***:..::*:*** .*:.::. :.:*:*..:::*:. *: ** * :*::*

gal AETTVKVPMMCRIGTFDLYF-DKDLPCTVVRLHYNGSATAFLILPAKGKMKQLEQTLDKE

mus VKRSVKVPMMKIKTLTTPYFRDEELSCTVVELKYKGNASALFILPDQGRMQQVEASLQPE

hum E**N**TTVRVPMMLQDQEHHWYLHDRYLPCSVLRMDYKGDATVFFILPNQGKMREIEEVLTPE

pan ENTTVRVPMMLQDQEHHWYLHDRYLPCSVLRMDYKGDATVFFILPNQGKMREIEEVLTPE

: :*:**** *: *. * *:*:.:.*:*.*:.::*** :*:*:::* * *

gal RVKKWSDHLFKS----KIQLYFPKFSISGTYEITNILSKMGIVDVFTNQADLSGISGVPE

mus TLRKWKNSLRPRKM---GELYLPKFSISTDYSLKNILPELGIKEIFSNQADLSGITGTKD

hum MLMRWNNLLRKRNFYKKLELHLPKFSISGSYVLDQILPRLGFTDLFSKWADLSGITKQQK

pan MLMRWNNLLQKRNFYKKLELHFPKFSISGSYVLDQILPRLGFTDLFSKWADLSGITKQQK

: :*.: * :*::****** * : :** .:*: ::*:: ******: .

gal LKVSKVIHKAALDVDERGTEASATAATP-K-IMALSLAPIIEFNRPFLMLIFDRDTNSTL

mus LIVSQMVHKVVLDVAETGTEGVAATGVNFRILS---RRTSLWFNRPFLMVISHTDVQTTL

hum LEASKSFHKATLDVDEAGTEAAAATSFAIKFFSAQTNRHILRFNRPFLVVIFSTSTQSVL

pan LEASKSFHKATLDVDEAGTEAAAATSFAIKFFSAQTNRHILRFNRPFLVVIFSTSTQSVL

* .*: .**..*** * ***. *::. : : : ******::* ..::.*

gal FIGKIANPTTTSRTEI

mus FIAKITHPKRA-----

hum FLGKVVDPTKP-----

pan FLGKVVDPTKP-----

*:.*:..*.

1. Ectonucleoside triphosphate diphosphohydrolase

gal MTSSRLPVLLALVFSSLSPVLSHSNREMWFQDLFPPNTCPINAKTKTFYGIMFDAGSTGT

hum MATSWGTVFFMLVVSCVCSAVSHRNQQTWFEGIFLSSMCPINVSASTLYGIMFDAGSTGT

pan MATSWGTVFFMLVVSCVCSTVSHRNQQTWFEGIFLSSMCPINVSASTLYGIMFDAGSTGT

mus MATSWGAVF-MLIIACVGSTVFYREQQTWFEGVFLSSMCPINVSAGTFYGIMFDAGSTGT

*::* *: *:.:.: .: : ::: **:.:* . ****..: *:************

gal RIHIYTFVQKSPEILPELEGEIFESVKPGLSAYADQPEKGAESVKRLLDMAIDAVPPHLW

hum RIHVYTFVQKMPGQLPILEGEVFDSVKPGLSAFVDQPKQGAETVQGLLEVAKDSIPRSHW

pan RIHIYTFVQKMPGQLPILEGEVFDSVKPGLSAFVDQPKQGAETVQGLLEVAKDSIPRSHW

mus RIHVYTFVQKTAGQLPFLEGEIFDSVKPGLSAFVDQPKQGAETVQELLEVAKDSIPRSHW

***:****** ** ****:*:********:.***::***:*: **::* *::* *

gal KKTPVVLKATAGLRLLSEEKAQALLSEVKEVFEESPFLVPEDSVSIMDGSYEGILAWITV

hum KKTPVVLKATAGLRLLPEHKAKALLFEVKEIFRKSPFLVPKGSVSIMDGSDEGILAWVTV

pan KKTPVVLKATAGLRLLPEHKAKALLFEVKEIFRKSPFLVPKGSVSIMDGSDEGILAWVTV

mus ERTPVVLKATAGLRLLPEQKAQALLLEVEEIFKNSPFLVPDGSVSIMDGSYEGILAWVTV

::************** *.**:*** **:*:*.:******..******** ******:**

gal NFLTGQLSGQNQHTVGTLDLGGASTQITFLPRFEETLKESPTDFLTSFEMFNSTYKLYTH

hum NFLTGQLHGHRQETVGTLDLGGASTQITFLPQFEKTLEQTPRGYLTSFEMF**N**STYKLYTH

pan NFLTGQLHGHRQETVGTLDLGGASTQITFLPQFEKTLEQTPRGYLTSFEMFNSTYKLYTH

mus NFLTGQLHGRGQETVGTLDLGGASTQITFLPQFEKTLEQTPRGYLTSFEMFNSTFKLYTH

******* *: *.******************:**:**:::* .:**********:*****

gal SYLGFGLKAARLATLGALNTEVADRQMFRSSCLPKQLEAEWHFGGVKYRYGGNKEGETGF

hum SYLGFGLKAARLATLGALETEGTDGHTFRSACLPRWLEAEWIFGGVKYQYGGNQEGEVGF

pan SYLGFGLKAARLATLGALETEGTDGHTFRSACLPRWLEAEWIFGGVKYQYGGNQEGEVGF

mus SYLGFGLKAARLATLGALEAKGTDGHTFRSACLPRWLEAEWIFGGVKYQYGGNQEGEMGF

******************::: :* : ***:***: ***** ******:****:*** **

gal KPCYLEVLKVVKGKLHQPDEIRGSSFYAFSYYYDRAADTNLIDYEQGGVLEVRDFERKAK

hum EPCYAEVLRVVRGKLHQPEEVQRGSFYAFSYYYDRAVDTDMIDYEKGGILKVEDFERKAR

pan EPCYAEVLRVVRGKLHQPEEVQRGSFYAFSYYYDRAVDTDMIDYEKGGILKVEDFERKAR

mus EPCYAEVLRVVQGKLHQPEEVRGSAFYAFSYYYDRAADTHLIDYEKGGVLKVEDFERKAR

:*** ***:**:******:*:: .:***********.**.:****:**:*:*.******:

gal EVCDNMERFSSASPFLCMDLTYITALLKEGFGFRDNTLLQLTKKVNNIETSWTLGATFHL

hum EVCDNLENFTSGSPFLCMDLSYITALLKDGFGFADSTVLQLTKKVNNIETGWALGATFHL

pan EVCDNLE**N**FTSGSPFLCMDLSYITALLKDGFGFADSTVLQLTKKVNNIETGWALGATFHL

mus EVCDNLGSFSSGSPFLCMDLTYITALLKDGFGFADGTLLQLTKKVNNIETGWALGATFHL

*****: *:*.********:*******:**** *.*:************.*:*******

gal LQSLGITY

hum LQSLGISH

pan LQSLGISH

mus LQSLGITS

******:

1. IgE chain C

gal EFVAAISSTGSGTNYGSAVKGRATISRDNGQSTLRLQLNNLRAEDTGTYYCARDLGYGDL

mus ------------------------------------------------------------

hum ------------------------------------------------------------

pan ------------------------------------------------------------

gal YAGQIDAWGHGTEVIVSSASPTSPPRLYPLSACCSDSA---VPPAVGCLLSPSSAG--GI

mus --------------------SIRNPQLYPLKPCKG-----TASMTLGCLVKDYFPNPVTV

hum -------------------ASTQSPSVFPLTRCCKNIPS**N**ATSVTLGCLATGYFPEPVMV

pan --------------------PTRSPSLFPLTRCCKNIPSNATSVTLGCLAMGYFPEPVMV

* ::**. * . ::*** :

gal SWEGSGGT--AV-----AGRVSGTPVKLSF--VRLSPGEKRKSFVCSAAPGGAL----LK

mus TWYSDSLNMSTVNFPALGSELKVTTS------QVTSWGKSAKNFTCHVTHPPSF---NES

hum TWDTGSL**N**GTTMTLPATTLTLSGHYATISLLTVSGAWA--KQMFTCRVAHTPSSTDWVD**N**

pan TWDAGSLNGTTMTLPATTLTPSGHYATISLLTVSGAWA--KQMFTCRVAHTPSSTDWVDN

:* .. . :: . : . : *.* .: : .

gal KEVQVCRVDPVPPVAPEVQVLHASSCTPS--QSESVELLCLVTGFSPASAEVEWLVDGVG

mus RTILVRPVNITE---PTLELLH-SSCDPN-AFHSTIQLYCFIYGHILNDVSVSWLMDDRE

hum KTFSVCSRDFTP---PTVKILQ-SSCDGGGHFPPTIQLLCLVSGYTPGTI**N**ITWLEDGQV

pan KTFSVCSRDFTP----TVKVLQ-SSCDGGGHFPPTIQLLCLVSGYTPGTINITWLEDGQV

: . * : . :::*: *** . :::* *:: *. .: ** *.

gal GLLVA-SQSPAVRSGSTYSLSSRVNVSGTDWREGKSYSCRVRHPATNTVVEDHVKGCPDG

mus ITDTLAQTVLIKEEGKLASTCSKLNITEQQWMSESTFTCKVTSQG--VDYLAHTRRCPDH

hum MDVDL-STASTTQEGELASTQSELTLSQKHWLSDRTYTCQVTYQG--HTFEDSTKKCADS

pan MDVDL-STASATQEGELASTQSELTLSQKHWLSDRTYTCQVTYQG--GTFEDSTKKCADS

. ..*. * *.:.:: .* . :::*:* . .: * *

gal AQSCSPIQLYAIPPSPGELYISLDAKLRCLVVNLPSD-SSLSVTWTREKSGNLRPDPMVL

mus EP--RGVITYLIPPSPLDLYQNGAPKLTCLVVDLESE-KNVNVTWNQEKKTSVSASQWYT

hum NP--RGVSAYLSRPSPFDLFIRKSPTITCLVVDLAPSKGTV**N**LTWSRASGKPVNHSTRKE

pan NP--RGVSAYLSRPSPFDLFIRKSPTITCLVVDLAPSKGTVNLTWSRASGKPVNHSTRKQ

: * *** :*: .: ****:* . .:.:**.: . : .

gal QEHFNGTYSASSAVPVSTQDWLSGERFTCTVQHEELPLPLSKSVYRNTGPTTPPLIYPFA

mus KHHNNATTSITSILPVVAKDWIEGYGYQCIVDHPDFPKPIVRSITKTPGQRSAPEVYVFP

hum EKQRNGTLTVTSTLPVGTRDWIEGETYQCRVTHPHLPRALMRSTTKTSGPRAAPEVYAFA

pan EKQRNGTLTVTSTLPVGTRDWIEGETYQCRVTHPHLPRALVRSTTKTSGPRAAPEVYAFA

:.: *.* : :* :** ::**:.* : * * * .:* : :* :. * : * :* *

gal PHPEELSLSRVTLSCLVRGFRPRDIEIRWLRDHRAVPATEFVTTAVLPEERTANGAGGDG

mus PPEE-ESEDKRTLTCLIQNFFPEDISVQWLGDGKLISNSQHSTTTPL-------KSNGSN

hum TPEWPGSRDKRTLACLIQNFMPEDISVQWLHNEVQLPDARHSTTQPR-------KTKG--

pan TPEGPGSRDKRTLACLIQNFMPEDISVQWLHNEVQLPDARHSTTQPH-------KTKG--

* .: **:**::.* *.**.::** : : :.. ** : *

gal DTFFVYSKMSVETAKWNGGTVFACMAVHEALPM-RFSQRTLQKQAGK------

mus QGFFIFSRLEVAKTLWTQRKQFTCQVIHEALQKPRKLEKTISTSLGNTSLRPS

hum SGFFVFSRLEVTRAEWEQKDEFICRAVHEAASPSQTVQRAVSVNPGK------

pan SGFFVFSRLEVTRAEWEQKDEFICRAVHEAASPSQTVQRTVSVNPGK------

. **::*::.* : * * * .:*** : ::::. . *:

1. Ubiquitin protein ligase RNF128

gal MADELTRLIQHSINLFQAGLAPPVAERSANINSAARSGPDAASCGLRHAGGRPGALLSAR

mus -SRERASLALACRSCVCSSLAADAA-----CRLAGQPATSLAPRPLLAAGSPPGSHLVPA

hum ------------------------------------------------------------

pan ------------------------------------------------------------

gal GVGCAAAGAAPLLPRRRWRGRGDLGGGGDSGGAVRCGMASGAAGLLAAVAALLCVVPEPG

mus RVACRVRAM-----------------GPPPGIGVYCRGGCGAARLLAWCFLLALSPHAPG

hum --------M-----------------GPPPGAGVSCRGGCGFSRLLAWCFLLALSPQAPG

pan --------M-----------------GPPPGAGVSCRGGCGFSRLLAWCFLLALSPQAPG

* * .* * ..* : *** * **

gal VGAAAAVWTAWLNVSWEDG-AGGNRSGWEAGESGLYGQDSPLQAAAGLLVLPDGRDAFNA

mus SRGAEAVWTAYLNVSWRVPHTGVNRTVWELSEEGVYGQDSPLEPVSGVLVPPDGPGALNA

hum SRGAEAVWTAYL**N**VSWRVPHTGV**N**RTVWELSEEGVYGQDSPLEPVAGVLVPPDGPGALNA

pan SRGAEAVWTAYLNVSWRVPHTGVNRTVWELSEEGVYGQDSPLEPVAGVLVPPDGPGALNA

.* *****:*****. :* **: ** .*.*:*******: .:*:** *** .*:**

gal CSALTNFSAAPPGG---GPGWVALIQRGGGCSFADKIRLAAERGAAAAVIYNYRGTGNDV

mus CNPHTNFTVPTVWGSTVQVSWLALIQRGGGCTFADKIHLASERGASGAVIFNFPGTRNEV

hum CNPHT**N**FTVPTVWGSTVQVSWLALIQRGGGCTFADKIHLAYERGASGAVIFNFPGTRNEV

pan CNPHTNFTVPTVWGSTVQVSWLALIQRGGGCTFADKIHLAYERGASGAVIFNFPGTRNEV

*. ***:. * .*:*********:*****:** ****:.***:*: ** *:*

gal LPMSHAGAGSIVAIMIGNLKGMEILRRIESGLKVTMVIEVGKKHGPWMNHYSIFFVSVSF

mus IPMSHPGAGDIVAIMIGNLKGTKILQSIQRGIQVTMVIEVGKKHGPWVNHYSIFFVSVSF

hum IPMSHPGAVDIVAIMIGNLKGTKILQSIQRGIQVTMVIEVGKKHGPWVNHYSIFFVSVSF

pan IPMSHPGAVDIVAIMIGNLKGTKILQSIQRGIQVTMVIEVGKKHGPWVNHYSIFFVSVSF

:**** ** .*********** :**: *: *::**************:************

gal FIVTAATVGYFIFYSARRFRITRAQSRKQRQLKAEAKKAIGQLQLRTLKQGDKETGPDGD

mus FIITAATVGYFIFYSARRLRNARAQSRKQRQLKADAKKAIGKLQLRTLKQGDKEIGPDGD

hum FIITAATVGYFIFYSARRLRNARAQSRKQRQLKADAKKAIGRLQLRTLKQGDKEIGPDGD

pan FIITAATVGYFIFYSARRLRNARAQSRKQRQLKADAKKAIGRLQLRTLKQGDKEIGPDGD

**:***************:* :************:******:************ *****

gal SCAVCIELYKPNEVVRILTCNHLFHKNCIDPWLLEHRTCPMCKCDILKVLGVEVDAEDGA

mus SCAVCIELYKPNDLVRILTCNHIFHKTCVDPWLLEHRTCPMCKCDILKALGIEVDVEDGS

hum SCAVCIELYKPNDLVRILTCNHIFHKTCVDPWLLEHRTCPMCKCDILKALGIEVDVEDGS

pan SCAVCIELYKPNDLVRILTCNHIFHKTCVDPWLLEHRTCPMCKCDILKALGIEVDVEDGS

************::********:***.*:*******************.**:***.***:

gal ESVQATVSSGTSNITSINEMDSHSETASSGYASVQGADESVLEEHAPSENDNTHLVNNES

mus VSLQVPVSNEASNTASPHEEDSRSETASSGYASVQGADEPPLEEHAQSANENLQLVNHEA

hum VSLQVPVSNEISNSASSHEEDNRSETASSGYASVQGTDEPPLEEHVQSTNESLQLVNHEA

pan VSLQVPVSNEISNSASSHEEDNRSETASSGYASVQGTDEPPLEEHVQSTNENLQLVNHEA

*:*. **. ** :* :* *.:*************:** ****. * *:. :***:*:

gal QTSAVTVLPPLDNPTFEADETQVSEVRS-----

mus NSVAVDVVPHVDNPTFEEDETPDQEAAVREIKS

hum NSVAVDVIPHVDNPTFEEDETPNQETAVREIKS

pan NSVAVDVIPHVDNPTFEEDETPNQETAVREIKS

:: ** *:* :****** *** .*.

1. Alpha-galactosidase A

gal -------------MAAAARWALVAAVVVALAVALDNGVARTPPMGWLHWERFLCGTDCAA

mus MAMKLLSRDTRLVCELALCPLALVFWSILGVRALDNGLARTPTMGWLHWERFMCNLDCQE

hum --MQLRNPELHLGCALALRFLALVSWDIPGARALDNGLARTPTMGWLHWERFMCNLDCQE

pan --MQLRNPELHLGCALALRFLALFSWDIPWARALDNGLARTPTMGWLHWERFMCNLDCQE

* : : . *****:**** *********:*. **

gal EPDRCVSERLFTEMADVMVAEGWKEAGYEFVCIDDCWMAPTRDERGRLRADPRRFPSGIR

mus EPDACISEQLFMQMAELMVSDGWRDAGYDYLCIDDCWMAPERDSKGRLQADPQRFPSGIK

hum EPDSCISEKLFMEMAELMVSEGWKDAGYEYLCIDDCWMAPQRDSEGRLQADPQRFPHGIR

pan EPDSCISEKLFMEMAELMVSEGWKDAGYEYLCIDDCWMAPQRDSEGRLQADPQRFPHGIR

*** *:**:** :**::**::**::***:::********* **..***:***:*** **:

gal ALADYVHSKGLKLGIYSDVGNTTCAGFPGSYGHYELDAQTFASWGVDLLKFDGCNADSLE

mus HLANYVHSKGLKLGIYADVGNKTCAGFPGSFGSYDIDAQTFADWGVDLLKFDGCHCDSVV

hum QLANYVHSKGLKLGIYADVG**N**KTCAGFPGSFGYYDIDAQTFADWGVDLLKFDGCYCDSLE

pan QLANYVHSKGLKLGIYADVGNKTCAGFPGSFGYYDIDAQTFADWGVDLLKFDGCYCDSLE

**:************:****.********:* *::******.*********** .**:

gal LLAEGYRNMSLALNKTGRPIVYSCEWPFYLRPMQQPNYTEIKQYCNHWRNFYDVYDSWNS

mus SLENGYKYMALALNRTGRSIVYSCEWPLYLRPFHKPNYTDIQYYCNHWRNFDDVYDSWES

hum NLADGYKHMSLAL**N**RTGRSIVYSCEWPLYMWPFQKP**N**YTEIRQYCNHWRNFADIDDSWKS

pan NLADGYKHMSLALNRTGRSIVYSCEWPLYMWPFQKPNYTEIRQYCNHWRNFADVDDSWKS

* :**: *:****:*** ********:*: *:::****:*: ******** *: ***:*

gal IKSIMEWTALHQDSIVKIAGPGGWNDPDMLVIGNFGLSWEQAVTQMAMWAIMAAPLFMSN

mus IKNILSWTVVYQKEIVEVAGPGSWNDPDMLVIGNFGLSWDQQVTQMALWAIMAAPLLMSN

hum IKSILDWTSFNQERIVDVAGPGGWNDPDMLVIGNFGLSWNQQVTQMALWAIMAAPLFMSN

pan IKSILDWTSFNQERIVDVAGPGGWNDPDMLVIGNFGLSWNQQVTQMALWAIMAAPLFMSN

**.*:.** . *. **.:****.****************:* *****:********:***

gal DLRHMKPEAKWLLQNKEVIAINQDPLGKQGYRITKDKNFELWERPLSDRAYAVAVLNQQE

mus DLRQISSQAKALLQNKDVIAINQDPLGKQGYCFRKENHIEVWERPLSNLAWAVAVRNLQE

hum DLRHISPQAKALLQDKDVIAINQDPLGKQGYQLRQGDNFEVWERPLSGLAWAVAMINRQE

pan DLRHISPQAKALLQDKDVIAINQDPLGKQGYQLRQADNFEVWERPLSGLAWAVAMINRQE

***::. :** ***:*:************** : : .::*:******. *:***: * **

gal IGGPQNFTFSLTFLGNGLACNPACCIQQILPTSRDWGVHNWVSSLSVEVNPTGTVLLKVV

mus IGGPCPYTIQISSLGRGLACNPGCIITQLLPEKVHLGFYEWTLTLKTRVNPSGTVLFRLE

hum IGGPRSYTIAVASLGKGVACNPACFITQLLPVKRKLGFYEWTSRLRSHI**N**PTGTVLLQLE

pan IGGPRSYTIALASLGKGVACNPACFITQLLPVKRKLGFYEWTSRLRSHINPTGTVLLQLE

**** :*: :: **.*:****.* * *:** . . *.::*. * .:**:****:::

gal PL---------

mus R----------

hum NTMQMSLKDLL

pan NTMQMSLKDLL

1. GDP-fucosyl transferase 2

gal MAAHHPGLLMLLLLVLATLAFLPPAAAQSLPTALRAGHAATSLSAAAAAPHTRYLLYDVN

mus -----MAALSVVCLLLAAASWRPVSAS---GEEFWPGQSAADI-LSGAASRRRYLLYDVN

hum -----MATLSFVFLLLGAVSWPPASAS---GQEFWPGQSAADI-LSGAASRRRYLLYDVN

pan -----MATLSFVFLLLGAVSWPPASAS---GQEFWPGQSAADI-LSGAASRRRYLLYDVN

. * .: *:*.: :: * :*: : *::*:.: :.** : ********

gal PPEGFNLRRDVYIRIASLMKTLLKSENWVLVLPPWGRLYHWQSPDILQVRIPWSEFFDLP

mus PPEGFNLRRDVYIRVASLLKTLLKTEEWVLVLPPWGRLYHWQSPDIHQVRIPWSEFFDLP

hum PPEGFNLRRDVYIRIASLLKTLLKTEEWVLVLPPWGRLYHWQSPDIHQVRIPWSEFFDLP

pan PPEGFNLRRDVYIRIASLLKTLLKTEEWVLVLPPWGRLYHWQSPDIHQVRIPWSEFFDLP

**************:***:*****:*:******************* *************

gal SLNKNIPVIEYEQFLAESGGPFIEQIYVLQGYEEGWKEGTWEEKIDERPCIDQLMYSKDK

mus SLNKNIPVIEYEQFIAESGGPFIDQVYVLQGYAEGWKEGTWEEKVDARPCIDPLLYSQDK

hum SLNKNIPVIEYEQFIAESGGPFIDQVYVLQSYAEGWKEGTWEEKVDERPCIDQLLYSQDK

pan SLNKNIPVIEYEQFIAESGGPFIDQVYVLQSYAEGWKEGTWEEKVDERPCIDQLLYSQDK

**************:********:*:****.* ***********:* ***** *:**:**

gal HQYYRGWFWGYEETRGLNVSCLSVQGSASVVAPILLKNTSAQSVMLDRAENLLHDHYGGK

mus HEYYRGWFWGYEETRGLNVSCLSVQGSASIVAPVLLKNTSARSVMLDRAENLLHDHYGGR

hum HEYYRGWFWGYEETRGL**N**VSCLSVQGSASIVAPLLLR**N**TSARSVMLDRAENLLHDHYGGK

pan HEYYRGWFWGYEETRGLNVSCLSVQGSASIVAPLLLRNTSARSVMLDRAENLLHDHYGGK

*:***************************:***:**:****:*****************:

gal DYWNTRRSMVFAKHLRVAGDEFRNKYLQSTDEADRTHYNEDWTQMKVKTGTALGGPYLGV

mus EYWDTRRSMVFAKHLRAVGDEFRSQHLNSTDAADKMAPEEDWTKMKVKLGSALGGPYLGV

hum EYWDTRRSMVFARHLREVGDEFRSRHL**N**STDDADRIPFQEDWMKMKVKLGSALGGPYLGV

pan EYWDTRRSMVFARHLREVGDEFRSRHLNSTDDADGIPFQEDWTKMKVKLGSALGGPYLGV

:**:********:*** .*****.::*:*** ** :*** :**** *:*********

gal HLRRKDFIWGHREDVPSLHGAAKKIHSLLKTHKLEKVFIATDAVEDEIELLKKLVPEMVR

mus HLRRKDFIWGHREDVPSLEGAVKKIRSLMKTHQLDKVFVATDAIRKEQEELRKLLPEMVR

hum HLRRKDFIWGHRQDVPSLEGAVRKIRSLMKTHRLDKVFVATDAVRKEYEELKKLLPEMVR

pan HLRRKDFIWGHRQDVPSLEGAVRKIRSLMKTHRLDKVFVATDAVRKEYEELKKLLPEMVR

************:*****.**.:**:**:***:*:***:****:..* * *:**:*****

gal FEPTWEELELYKDGGMAVIDQWICAHARYFIGTSVSTFSFRIHEEREILGFDPKTTYNRF

mus FEPTWEELELYKDGGVAIIDQWICAHARFFIGTSVSTFSFRIHEEREILGLDPKTTYNRF

hum FEPTWEELELYKDGGVAIIDQWICAHARFFIGTSVSTFSFRIHEEREILGLDPKTTYNRF

pan FEPTWEELELYKDGGVAIIDQWICAHARFFIGTSVSTFSFRIHEEREILGLDPKTTYNRF

***************:*:**********:*********************:*********

gal CGEKEKNCEQPTHWKIVY

mus CGDQEKACEQPTHWKIAY

hum CGDQEKACEQPTHWKITY

pan CGDQEKACEQPTHWKITY

**::** *********.*

1. Urokinase type plasminogen activator

gal MKLIIFLTVTLCTLVTGLDSVYIRQYYKLSHKHRPQHRECQCLNGGTCITYRFFSQIKRC

mus MKV-WLASLFLCALVVK-------NSEGGSVLGAPDESNCGCQNGGVCVSYKYFSRIRRC

hum MRA-LLARLLLCVLVVS-------DSKGSNELH-QVPSNCDCLNGGTCVSNKYFSNIHWC

pan MRA-LLARLLLCVLVVS-------DSKGSNELH-QVPSNCDCLNGGTCVSNKYFSNIHWC

*: : : **.**. : . :* * ***.*:: ::**.*: *

gal LCPEGYGGLHCEIDTNSVCYSGNGEDYRGMAED----PGCLYWDHPSVIRWGDYHADLKN

mus SCPRKFQGEHCEIDASKTCYHGNGDSYRGKANTDTKGRPCLAWNAPAVLQ-KPYNAHRPD

hum NCPKKFGGQHCEIDKSKTCYEGNGHFYRGKASTDTMGRPCLPWNSATVLQ-QTYHAHRSD

pan NCPKKFGGQHCEIDKSKTCYEGNGHFYRGKASTDTMGRPCLAWNSATVLQ-QTYHAHRSD

**. : * ***** ...** ***. *** *. ** *: :*:: *:*. :

gal ALQLGLGKHNYCRNPNGRSRPWCYTKRRYS------------IQETPCSTIEKCERTCGQ

mus AISLGLGKHNYCRNPDNQKRPWCYVQIGLRQFVQECMVHDCSLSKKPSSSVDQQGFQCGQ

hum ALQLGLGKHNYCRNPDNRRRPWCYVQVGLKPLVQECMVHDCADGKKPSSPPEELKFQCGQ

pan ALQLGLGKHNYCRNPDNRRRPWCYVQVGLKPLVQECMVHDCADGKKPSSPPEELKFQCGQ

*:.************:.: *****.: :.*.* :: ***

gal RSFSKYFKIVGGSQAEVETQPWIAGIFQNIMGT--DQFLCGGSLIDPCWVLTAAHCFYNP

mus KALRPRFKIVGGEFTEVENQPWFAAIYQKNKGGSPPSFKCGGSLISPCWVASAAHCFIQL

hum KTLRPRFKIIGGEFTTIENQPWFAAIYRRHRGGS-VTYVCGGSLISPCWVISATHCFIDY

pan KTLRPRFKIIGGEFTTIENQPWFAAIYRRHRGGS-VTYLCGGSLISPCWVISATHCFIDY

::: ***:**. : :*.***:*.*::. * : ******.**** :*:*** :

gal TKKQPNKSVYKVFLGKSILNTNDEHEQVFMVDEIISHPDFTDHTGGNDNDIALIRIRTAS

mus ----PKKENYVVYLGQSKESSYNPGEMKFEVEQLILHEYYREDSLAYHNDIALLKIRTST

hum ----PKKEDYIVYLGRSRLNSNTQGEMKFEVENLILHKDYSADTLAHHNDIALLKIRSKE

pan ----PKKEDYIVYLGRSRLNSNTQGEMKFEVENLILHKDYSADTLAHHNDIALLKIRSKE

*:*. * *:**:* .: * * *:::* * : .: . .*****::**:

gal GQCAVESNYVRTVCLPEKNLNLYDNTWCEIAGYGKQNSYDIYYAQRLMSATVNLISQDDC

mus GQCAQPSRSIQTICLPPRFTDAPFGSDCEITGFGKESESDYLYPKNLKMSVVKLVSHEQC

hum GRCAQPSRTIQTICLPSMYNDPQFGTSCEITGFGKE**N**STDYLYPEQLKMTVVKLISHREC

pan GRCAQPSRTIQTICLPSMYNDPQFGTSCEITGFGKENSTDYLYPEQLKMTVVKLISHREC

*:** *. ::*:*** : .: ***:*:**:.. * * :.* :.*:*:*: :*

gal KNKYYDSTRVTDNMVCAGDPLWETDACKGDSGGPMVCEHNGRMTLYGIVSWGDGCAKKNK

mus MQPHYYGSEINYKMLCAADPEWKTDSCKGDSGGPLICNIEGRPTLSGIVSWGRGCAEKNK

hum QQPHYYGSEVTTKMLCAADPQWKTDSCQGDSGGPLVCSLQGRMTLTGIVSWGRGCALKDK

pan QQPHYYGSEVTTKMLCAADPQWKTDSCQGDSGGPLVCSLQGRMTLTGIVSWGRGCALKDK

: :* .:.:. :*:**.** *:**:*:******::*. :** ** ****** *** *:*

gal PGVYTRVTRYLNWIDSNMNAVFTKSRSFREPK

mus PGVYTRVSHFLDWIQSHIGEEKGLAF------

hum PGVYTRVSHFLPWIRSHTKEENGLAL------

pan PGVYTRVSHFLPWIRSHTKEENGLAL------

*******:::* ** *: :

1. Transcobalamin

hum ------MRQSHQLPLVGLLL-----FSF----IPSQLCEICEVSEENYIRLKPLLNTMIQ

pan ------MRQSHQLPLVGLLL-----FSF----IPSQLCEICEVSEENYIRLKPLLNTMIQ

gal MQRQRGTAGTEVLRMLGVALSTAVLLALAGCGVEGTDPQNCTVSAEERARML---GILQL

mus ------------MAWLTLY-LLSVLWAVA--GTSTRAQSSCSVPPDQQPWVDGLQALMEN

: : : :. . * * :: : :

hum SNYNRGTSAVNVVLSLKLVGIQIQTLMQKMIQQIKYNVKSRLSDVSSGELALIILALGV-

pan SNYNRGTRAVSVVLSLKLVGIQIQTLMQKMIQQIKYNVKSRLSDVSSGELALIILALGV-

gal SAMDSGTPNPSVLLALNLAGDSS-KARQELLERIKETAAKQAKDMSSGQVALYTLALRSS

mus SVTDSDFPNPSILIAMNLAGAYN-VEAQKLLTYQ--LMASDSADLTSGQLALTVMALTSS

* : . .::::::*.* *::: . *::**::** :**

hum CRNAEENLI--YDYHLIDKLENKFQAEIENMEAHNGTPLTNYYQLSLDVLALCLFNG**N**YS

pan CRNAEENLI--YDYHLIDKLENKFQAEIENMEAHNGTPLTNYYQLSLDVLALCLFNGNYS

gal CCDPGDVAAHGQSVDLLSILQEKTGQELT-HLEQNGTPKSSLFSVGLDAQALCVTGAGDY

mus CRDPGSKVSTL-----LKKM------ENW-SPSSPGAESSAFYGPGLAILALCQKSSEAT

* : . :. : * *: : : .* *** ..

hum TAEVVNHFTPENKNYYFGSQFSVDTGAMAVLALTCVKKSLINGQIKADEGSLK**N**ISIYTK

pan TAEVVNHFTPENKNYYFGSQFSVDTGAMAVLALTCVKKSLINGQVKADEGSLKNISIYTK

gal ESAAT-IL--AKQLQRSQDKLSVDEQAMMALALVCAYNRTEHKD---VQDLL---NRTLT

mus LPIAV-RF--AKTLMMEPSPFNVDTGAVATLALTCMYNKIPVGSQENYRDLF---GQALK

.. : : . :.** *: .***.* : . .. : . .

hum SLVE-KILSEKKENGLIGNTFSTGEAMQALFVSSDYYNENDWNCQQTLNTVLTEISQGAF

pan SLVE-KILSEKKENGLIGNTFSTGEAMQALFVSSDYYNENDWNCQQTLNTVLTEISQGAF

gal MVSNGFLDKQAEGNGIIGNIYSTGLAMQLLLAAGKFYAPRPWDCTQPVAAI----TAQHL

mus AIVE-KISLRIKADGIIGDIYSTGLAMQALSVTP-EQPTKKWDCEKTMHTILNEIKQGKF

: : : . : :*:**: :*** *** * .: . *:* : : :: . :

hum SNPNAAAQVLPALMGKTFLDINKDSSCVSASGNF**N**ISAD--EPITVTPPDSQSYISV**N**YS

pan SNPNAAAQVLPALMGKTFLDINKDSSCVSASGNFNISAD--EPITVTPPDSQSYISVNYS

gal QQPMAVAQALPALVGRTYLDSASLDCSPEAPTTAGMQLDTVLTKGTTPEQAGSNITVNYT

mus QNPMSIAQILPSLKGKTYLDVPQVTCGPDHEVPPTLT-----DYPTPVPTSVSNITVIYT

.:* : ** **:* *:*:** . . . : . : * *:* *:

hum VRI**N**-------ETYFT**N**VTVL**N**GSVFLSVMEKAQKM**N**DTIFGFTMEERSWGPYITCIQGL

pan VRIN-------ETYFTNVTVLNGSVFLSVMEKAQKMNDTIFGFTMEERSWGPYITCIQGL

gal ITNEVRGKHFSYS--TEVEVPAGSVLLVVLEEAQKSNKTIFSFKTESTFWGPMVVSIHGL

mus INNQLRGVDLLFNVTIEVSVKSGSVLLAVLEEAQRK-NSMFKFETTMTSWGLIVSSINNI

: : . :* * ***:* *:*:**: .::* * ** : .*:.:

hum CANNNDRTYWELLSGGEPLSQGAGSYVVRNGENLEVRWSKY

pan CANDNDRTYWELLSGGKPLSQGAGSYVVRNGENLEVRWSKY

gal AASENDRTFWQFFNGSVPLQEGVGTYKPQDGEHIRAVFSIY

mus AENVNHKTYWEFLSGKTPLDEGVAYYIPFNHEHITANFTQY

. . *.:*:*:::.* **.:*.. * : *:: . :: *

1. Hyaluronidase

gal ----------------------------MAPGWLCWALLLLLPAPVRAAGPGPVLVNRPF

mus MLGLTQHAQKVWRMKSFSPEVSPGSSPATAGHLLRISTLFLTLLELAQVCRGSVVSNRPF

hum ----------------------------MAAHLLPICALFLTLLDMAQGFRGPLLPNRPF

pan ----------------------------MAAHLLPICALFLTLLDMAQGFRGPLLPNRPF

* * . *:* : * :: ****

gal VTVWNIPSEPCAQQYNVTLPLGVFDVVANTEEAFIGQDIALFYSSHLGLFPYYTAQGQPV

mus ITVWNGDTHWCLTEYGVDVDVSVFDVVANKEQSFQGSNMTIFYREELGTYPYYTPTGEPV

hum TTVWNANTQWCLERHGVDVDVSVFDVVANPGQTFRGPDMTIFYSSQLGTYPYYTPTGEPV

pan TTVWNANTQWCLERHGVDVDVSVFDVVANPGQTFRGPDMTIFYSSQLGTYPYYTPTGEPV

**** :. * .:.* : :.******* ::* * ::::** ..** :**** *:**

gal DGGLPQNASLVAHLQRARRDIGAALPSTRYDGLAVVDWEQWRPLWARDWSSMDIYREKSE

mus FGGLPQNASLVTHLAHTFQDIKAAMPEPDFSGLAVIDWEAWRPRWAFNWDSKDIYRQRSM

hum FGGLPQ**N**ASLIAHLARTFQDILAAIPAPDFSGLAVIDWEAWRPRWAFNWDTKDIYRQRSR

pan FGGLPQNASLIAHLARTFQDILAAIPAPDFSGLAVIDWEAWRPRWAFNWDTKDIYRQRSR

*********::** :: :** **:* :.****:*** *** ** :*.: ****::*

gal ELVRRQHPLWPHSHVEEVAQQQFQKAARAFMEQTLRLGEAMRPAGYWGFYGFPDCYNNNF

mus ELVQAEHPDWPETLVEAAAKNQFQEAAEAWMAGTLQLGQVLRPRGLWGYYGFPDCYNNDF

hum ALVQAQHPDWPAPQVEAVAQDQFQGAARAWMAGTLQLGRALRPRGLWGFYGFPDCYNYDF

pan ALVQAQHPDWPAPQVEAVAQDQFQGAARAWMAGTLQLGRALRPRGLWGFYGFPDCYNYDF

**: :** ** ** .*::*** **.*:* **:**..:** * **:******** :*

gal SDPLYNGSCPVVEQQRNQELGWLWNCSRALYPSIYLPQQLQGTDKVLRYVRYRVAEAFAV

mus LSLNYTGQCPVFVRDQNDQLGWLWNQSYALYPSIYLPAALMGTGKSQMYVRHRVQEALRV

hum LSP**N**YTGQCPSGIRAQNDQLGWLWGQSRALYPSIYMPAVLEGTGKSQMYVQHRVAEAFRV

pan LSPNYTGQCPSGIRAQNDQLGWLWGQSRALYPSIYMPAVLEGTGKSQMYVQHRVAEAFRV

. *.*.** : :*::*****. * *******:* * **.* **::** **: *

gal QKGILSNAVPVLPYTEIIYANTSDFLSEEDLVNTIGESAAQGAAGIIIWGRSADTASKEK

mus AIVSRDPHVPVMPYVQIFYEMTDYLLPLEELEHSLGESAAQGVAGAVLWLSSDKTSTKES

hum AVAAGDPNLPVLPYVQIFYDTTNHFLPLDELEHSLGESAAQGAAGVVLWVSWENTRTKES

pan AVAAGDPNLPVLPYVQIFYDTTNHFLPLDELEHSLGESAAQGAAGVVLWVSWENTRTKES

. :**:**.:*:* *. :* ::* :::*******.** ::* .* :**.

gal CLKLRDYLDGALGHYIVNVTTSAQLCSQSLCSGNGRCVRKEGK-VAFLQLDPNRFAINLK

mus CQAIKAYMDSTLGPFIVNVTSAALLCSEALCSGHGRCVRHPSYPEALLTLNPASFSIELT

hum CQAIKEYMDTTLGPFIL**N**VTSGALLCSQALCSGHGRCVRRTSHPKALLLLNPASFSIQLT

pan CQAIKEYMDTTLGPFILNVTSGALLCSQALCSGHGRCVRRTSHPKALLLLNPASFSIQLT

* :: *:* :** :*:***:.* ***::****:*****: . *:* *:* *:*:*.

gal AKKPQPMVWILNADSDMSQLAEGFTCQCYSGWRGEHCDSHGSSNK

mus HDGRPPSLKGTLSLKDRAQMAMKFRCRCYRGWRGKWCDKRGM---

hum PGGGPLSLRGALSLEDQAQMAVEFKCRCYPGWQAPWCERKSMW--

pan PGGGPLSLQGALSLEDQAQMAVEFKCRCYPGWQGPWCEQKSMW--

: : .* :*:* * *:** **:. *: :.

1. Epsilon sarcoglycan

gal MRCCAGPGPRGGGALTTLLLTAFLLSSADGSNGTVNWKTKQANSY--IISRIKAVEKNVY

hum -----------------MQLPRW-----WELGDPCAWTGQGRGTRRMSPATTGTFLLTVY

pan -----------------MQLPRW-----WELGDPCAWTGQGRGTRRMSPATTGTFLLTVY

mus -----------------MLLFWW-----WELGDPCAWTGKGRGTLKMSPATTGTFLLTVY

: * : .. *. : .: : :. .**

gal TILSKVHSDRNVYPSAGVLFVHVLEREYFKGEFPPYPKPGEISNDPITFNTNLMGYPDRP

hum SIFSKVHSDRNVYPSAGVLFVHVLEREYFKGEFPPYPKPGEISNDPITFNTNLMGYPDRP

pan SIFSKVHSDRNVYPSAGVLFVHVLEREYFKGEFPPYPKPGEISNDPITFNTNLMGYPDRP

mus TLFSKVHSDRNVYPSAGVLFVHVLEREYFKGEFPPYPKPGEVSNDPITFNTNLMGYPDRP

:::**************************************:******************

gal GWLRYIQRTPYSDGVLYGSPTVENVGKPTVIEITAYNRRTFETARHNLIINIMSAEDFPL

hum GWLRYIQRTPYSDGVLYGSPTAENVGKPTIIEITAYNRRTFETARHNLIINIMSAEDFPL

pan GWLRYIQRTPYSDGVLYGSPTAENVGKPTIIEITAYNRRTFETARHNLIINIMSAEDFPL

mus GWLRYIQRTPYSDGVLYGSPTAENVGKPTIIEITAYNRRTFETARHNLIINIMSAEEFPL

*********************.*******:**************************:***

gal PYQAEFFIRNMNVEEMLASEVLGDFLGAVKNVWQPERLNAINITSALDRGGRVPLPINDM

hum PYQAEFFIKNMNVEEMLASEVLGDFLGAVKNVWQPERLNAINITSALDRGGRVPLPINDL

pan PYQAEFFIKNMNVEEMLASEVLGDFLGAVKNVWQPERLNAI**N**ITSALDRGGRVPLPINDL

mus PYQAEFFIKNMNVEEMLASEVLGDFLGAVKNVWQPERLNAINITSALDRGGRVPLPINDM

********:**************************************************:

gal KEGVYVMVGADVPFSSCLREVENPQNQLRCSQEMEPSITCDKKFRTQFHIDWCKISLVDN

hum KEGVYVMVGADVPFSSCLREVENPQNQLRCSQEMEPVITCDKKFRTQFYIDWCKISLVDK

pan KEGVYVMVGADVPFSSCLREVENPQNQLRCSQEMEPVITCDKKFRTQFYIDWCKISLVDK

mus KEGVYVMVGADVAFSSCLREVENPQNQLRCSQEMEPVITCDKKFRTHFHIDWCKISLVDK

************ *********************** *********:*:**********:

gal TKQVSTFQEVIRGEGILPDGGEYKPPSDTLKSRDYYSDFLITLAVPSAVALVLFLILAYI

hum TKQVSTYQEVIRGEGILPDGGEYKPPSDSLKSRDYYTDFLITLAVPSAVALVLFLILAYI

pan TKQVSTYQEVIRGEGILPDGGEYKPPSDSLKSRDYYTDFLITLAVPSAVALVLFLILAYI

mus TKQVSTYQEVVRGEGILPDGGEYKPPSDSLKSRDYYTDFLVTLAVPSAVALVLFLILAYI

******:***:*****************:*******:***:*******************

gal MCCRREGVEKRNMQTPDIQLVHHSAIQKSTKELRDMSKNREIAWPLSTLPVFHPVTGEIV

hum MCCRREGVEKRNMQTPDIQLVHHSAIQKSTKELRDMSKNREIAWPLSTLPVFHPVTGEII

pan MCCRREGVEKRNMQTPDIQLVHHSAIQKSTKELRDMSKNREIAWPLSTLPVFHPVTGEII

mus MCCRREGVEKRDMQTPDIQLVHHSSIQKSTKELRDMSKNREIAWPLSTLPVFHPVTGEVI

***********:************:*********************************::

gal PPLHPDNYDNTSMPLMQTQQNLPHQTQIPQQQSAGKWYS

hum PPLHTDNYDSTNMPLMQTQQNLPHQTQIPQQQTTGKWYP

pan PPLHTDNYDSTNMPLMQTQQNLPHQTQIPQQQTTGKWYP

mus PPTHTDNYDSTNMPLMQAQQNLPHQTQIPQPQTTGKWYP

** * ****.*.*****:************ *::****

1. Battenin

gal ------------------------------------------------------MLSAAH

mus MGSSAGSWRRLEDSEREETDSEPQAPRLDSRSVLWKNAVGFWILGLCNNFSYVVMLSAAH

hum MGGCAGSRRRFSDSEGEETVPEPRLPLLDHQGAHWKNAVGFWLLGLCNNFSYVVMLSAAH

pan ------------------------------------------------------MLSAAH

******

gal DILRPNQ------------LPPAPHNTSGHDCGPISTGAVLLADILPSLLIKMAAPFGAH

mus DILKQEQASGNQSHVEPGRTPTPHNSSSRFDCNSISTAAVLLADILPTLVIKLLAPLGLH

hum DILSHKRTSG**N**QSHVDPGPTPIPH**N**SSSRFDCNSVSTAAVLLADILPTLVIKLLAPLGLH

pan DILSHKRTSGNQSHVDPGPTLMPHNSSSRFDCNSVSTAAVLLADILPTLVIKLLAPLGLH

*** :: :.:* .**. :**.*********:*:**: **:* *

gal LVPYSVRVVAVAIAAWGGCSVVAMGAGVGVSLGGEWLWGSAMGQ-------------RYG

mus LLPYSPRVLVSGVCSAGSFVLVAFSQSVGLSLCGVVLASISSGLGEVTFLSLTAFYPSAV

hum LLPYSPRVLVSGICAAGSFVLVAFSHSVGTSLCGVVFASISSGLGEVTFLSLTAFYPRAV

pan LLPYSPRVLVSGICAAGSFVLVAFSHSVGTSLCGVVFASISSGLGEVTFLSLTAFYPRAV

*:*** **:. .:.: *. :**:. .** ** * : . : *

gal AGMWGSAWG---------------------------------------------------

mus ISWWSSGTGGAGLLGSLSYLGLTQAGLSPQHTLLSMLGIPVLLLASYFLLLTSPEPWDPG

hum ISWWSSGTGGAGLLGALSYLGLTQAGLSPQQTLLSMLGIPALLLASYFLLLTSPEAQDPG

pan ISWWSSGTGGAGLLGALSYLGLTQAGLSPQQTLLSMLGIPALLLASYFLLLTSPEAQDPG

. *.*. *

gal ------------------------------------------------------------

mus GENEAETAARQPLIGTETPESKPGASWDLSLQERWTVFKGLLWYIIPLVLVYFAEYFINQ

hum GEEEAESAARQPLIRTEAPESKPGSSSSLSLRERWTVFKGLLWYIVPLVVVYFAEYFINQ

pan GEEEAESAARQPLIRTEAPESKPV------------------------------------

gal ------------------------------------------------------------

mus GLFELLFFRNTSLSHAHEYRWYQMLYQAGVFASRSSLQCCRIRFTWVLALLQCLNLALLL

hum GLFELLFFW**N**TSLSHAQQYRWYQMLYQAGVFASRSSLRCCRIRFTWALALLQCLNLVFLL

pan ------------------------------------------------------------

gal ------------------------------------------------------------

mus ADVCLNFLPSIYLIFIIILHEGLLGGAAYVNTFHNIALETSDKHREFAMEAACISDTLGI

hum ADVWFGFLPSIYLVFLIILYEGLLGGAAYVNTFHNIALETSDEHREFAMAATCISDTLGI

pan ------------------------------------------------------------

gal ------------------

mus SLSGVLALPLHDFLCHLP

hum SLSGLLALPLHDFLCQLS

pan ------------------

1. Fibroleukin

gal MKQLVYLVLLKTALLALTNVFAVILEEEKDAKEEKTTGACPIKLKTNGNCDEGEDCPYQI

hum MKLAN-WYWLSSAVLATYGFLVVAN**N**ETEEIKDERAKDVCPVRLESRGKCEEAGECPYQV

pan MKLAN-WYWLSSAVLATYGFLVVANNETEEIKDERAKDVCPVRLESRGKCEEAGECPYQV

mus MRLPG-WLWLSSAVLAACRAV-EEHNLTEGLEDASAQAACPARLEGSGRCEG-SQCPFQL

*: *.:*:** . : : :: : .** :*: *.*: :**:*:

gal NLPPMTIQLPKQFRLIEKTLKEVQTLKEAVNKLKKCCQDCKLQADDNQERDSSNEFLPPN

hum SLPPLTIQLPKQFSRIEEVFKEVQNLKEIVNSLKKSCQDCKLQADDNGDPGR-NGLLLPS

pan SLPPLTIQLPKQFSRIEEVFKEVQNLKEIVNSLKKSCQDCKLQADDNGDPGR-NGLLLPS

mus TLPTLTIQLPRQLGSMEEVLKEVRTLKEAVDSLKKSCQDCKLQADDHRDPGG-NGGNG--

.** :*****:*: :*:.:***:.*** *:.***.**********: : . *

gal TETPAEIQDNRVKELQSKVNRMATSLKNARNQIQTLQGRIEKMSLLNMNNVEHYVDSKVA

hum TGAPGEVGDNRVRELESEVNKLSSELKNAKEEINVLHGRLEKL**N**LVNMNNIENYVDSKVA

pan TGAPGEVGDNRVRELESEVNKLSSELKNAKEEINVLHGRLEKLNLVNMNNIENYVDSKVA

mus ---AETAEDSRVQELESQVNKLSSELKNAKDQIQGLQGRLETLHLVNMNNIENYVDNKVA

*.**:**:*:**::::.****:::*: *:**:*.: *:****:*:***.***

gal NLTFVVNSLDNKCSSQCP---AMQPSPVIQVMQRDCADHYAAGKRSSGIYPVTPDPRNST

hum NLTFVVNSLDGKCSK-CPSQEQIQSRPVQHLIYKDCSDYYAIGKRSSETYRVTPDPK**N**SS

pan NLTFVVNSLDGKCSK-CPSQEQIQSRPVQHLIYKDCSDYYAIGKRSSETYRVTPDPKNSS

mus NLTVVVNSLDGKCSK-CPSQEHMQSQPVQHLIYKDCSDHYVLGRRSSGAYRVTPDHRNSS

***.******.***. ** :* ** ::: :**:*:*. *:*** * **** :**:

gal FQVYCDMETQGGGWTVLQRRQDGSTNFNRTWNEYKHGFGNLSREFWLGNDKIHLLTKSQE

hum FEVYCDMETMGGGWTVLQARLDGST**N**FTRTWQDYKAGFGNLRREFWLGNDKIHLLTKSKE

pan FEVYCDMETMGGGWTVLQARLDGSTNFTRTWQDYKAGFGNLRREFWLGNDKIHLLTKSKE

mus FEVYCDMETMGGGWTVLQARLDGSTNFTREWKDYKAGFGNLEREFWLGNDKIHLLTKSKE

*:******* ******** * ******.* *::** ***** ****************:*

gal MQLRIDLEDFNGIKEYAKYQYFYVANEYLKYRLSVHGYSGTAGDALLYSKHYNHDQKFFT

hum MILRIDLEDFNGVELYALYDQFYVANEFLKYRLHVGNY**N**GTAGDALRFNKHYNHDLKFFT

pan MILRIDLEDFNGVKLYALYDQFYVANEFLKYRLHVGNYNGTAGDALRFNKHYNHDLKFFT

mus MILRIDLEDFNGLTLYALYDQFYVANEFLKYRLHIGNYNGTAGDALRFSRHYNHDLRFFT

* **********: ** *: ******:***** : .*.******* :.:***** :***

gal TPDKDNDRYASGNCGAFYSSGWWFDACLSANLNGKYYHKKYKGVRNGIFWGTWHGISDDT

hum TPDKDNDRYPSGNCGLYYSSGWWFDACLSANLNGKYYHQKYRGVRNGIFWGTWPGVSEAH

pan TPDKDNDRYPSGNCGLYYSSGWWFDACLSANLNGKYYHQKYRGVRNGIFWGTWPGVSEAH

mus TPDRDNDRYPSGNCGLYYSSGWWFDSCLSANLNGKYYHQKYKGVRNGIFWGTWPGINQAQ

***:***** ***** :********:************:**:*********** *:.:

gal PSGYKQSLKSVKIMIRPKSFAP

hum PGGYKSSFKEAKMMIRPKHFKP

pan PGGYKSSFKEAKMMIRPKHFKP

mus PGGYKSSFKQAKMMIRPKNFKP

*.***.*:*..*:***** * *

1. Leukocyte Ig-like receptor

gal ------------------------------------------------------------

mus MSCTFTALLRLGLTLSLWIPVLTGSLPKPILRVQPDSVVSRRTKVTFLCEETIGANEYRL

hum ------------------------------------------------------------

pan ------------------------------------------------------------

gal ------------------------------------------------------------

mus YKDGKLYKTVTKNKQKPENKAEFSFSNVDLSNAGQYRCSYSTQYKSSGYSDLLELVVTGH

hum ------------------------------------------------------------

pan ----------------------MSVEELGPPPPQPYRK----D-----------------

gal ------------------------------------------------------------

mus YWTPSLLAQASPVVTSGGYVTLQCESWHNDHKFILTVEGPQKLSWTQDSQYNYSTRK-YH

hum ------------------------------------------------------------

pan --------------SQGPGRAVPLPVWLQMTKPHEKKDPASKCPH-PVCPLSCQHRGLIH

gal ------------------------------------------------------------

mus ---ALFSVGPVTP-NQRWICRCYSYDRNRPYVWSPPSESVELLVSGNLQKPTIKAEPGSV

hum ----------MTPILTVLICLGLSL------------DPRTHVQAGPLPKPTLWAEPGSV

pan PQSSAVGGDAMTPILTVLICLGLSL------------DPRTHVQAGPLPKPTLWAEPGSV

gal ------------------------------------------------------------

mus ITSKRAMTIWCQGNLDAEVYFLHNEKSQKTQSTQTLQEPGNKGKFFIPSVTLQHAGQYRC

hum ITQGSPVTLRCQGSLETQEYHLYREKKTALWITRIPQELVKKGQFPILSITWEHAGRYCC

pan ITQGSPVTLRCQGSLETQEYHLYREKKTALWITRIPQELVKKGQFPIPSITWEHAGRYCC

gal ------------------------------------------------------------

mus YC-YGSAGWSQPSDTLELVVTGIYEYYEPRLSVLPSPVVTAGGNMTLHCASDFPYDKFIL

hum IYGSHTAGLSESSDPLELVVTGAY--SKPTLSALPSPVVTSGG**N**VTIQCDSQVAFDGFIL

pan IYGSHTAGRSESSDPLELVVTGAY--SKPTLSALPSPVVTSGGNVTLQCDSQVAFDGFIL

gal ------------------------------------------------------------

mus TKEDKKFGNSLDTEHISSSGQYRALFIIGPTTPTHTGAFRCYGYYKNAPQLWSVPSALQQ

hum CKEGEDEHPQCLNSHSHARGSSRAIFSVGPVSPSRRWSYRCYGYDSRAPYVWSLPSDLLG

pan CKEGEYEHPQCLNSHSHARGSSRAIFSVGPVSPSRRWSYRCYGYDSHAPYVWSLPSDLLE

gal ------------SLHPSQGVSPGDPVTLRCHLPRLAAWVWLYREGGWSYKKGKEKEQD--

mus ILISGLSKKPSLLTHQGHILDPGMTLTLQCFSDINYDRFALHKVGGADIMQHSSQQTDTG

hum LLVPGVSKKPSLSVQPGPVVAPGEKLTFQCGSDAGYDRFVLYKEWGRDFLQRPGRQPQAG

pan LLVPGVSKKPSLSVQPGPVVAPGEKLTLQCGSDVGYDRFVLFKEWGRDFLQRPGRQPQAG

: . : ** :*::* . *.: * . : :: :

gal --TTEFFFASTLWEHAGRYRCQYRVSESAEVSVESDPVELVLTDLRYPPSSISLHPEQHV

mus FSVANFTLGYVSSSTGGQYRCYGAHNLSSEWSASSEPLDILITGQLPLTPSLSVQPNHTV

hum LSQA**N**FTLGPVSRSYGGQYTCSGAY**N**LSSEWSAPSDPLDILITGQIRARPFLSVRPGPTV

pan LSQANFTLGPVSRSYGGQYRCSGAHNLSSEWSAPSNPLDILIAGQIRARPFLSVRPGPTV

::* :. . . .*:* * . *:* *. *:*::::::. :*::* *

gal GTGTNVTIRCWNKDYGATFLLHKDGSSDPL---QCQDSSGGGTATFTLFGVTPADSGTYR

mus HSGETVSLLCWSMDSVDTFILSKEGSAQQPLRLKSKSHDQQSQAEFSMSAVTSHLSGTYR

hum ASGE**N**VTLLCQSQGGMHTFLLTKEGAADSPLRLKSKRQSHKYQAEFPMSPVTSAHAGTYR

pan ASGENVTLLCQSQGGMNTFLLTKEGAADSPLRLKSKRQSHKYQAEFPMSPVTSAHAGTYR

:* .*:: * . . **:* *:*::: :.: . * * : ** :****

gal CSYRPWRYAFMSSPLGDSVMLE--------------------------------------

mus CYGAQDSSFYLLSSASAPVELTVSGPIETSTPPPTMSMPLGGLHMYLKALIGVSVAFILF

hum CYGSLSSNPYLLTHPSDPLELVVSGAAETLSPPQ**N**KSDSKAGE-----------------

pan CYGSLSSNPYLLTHPSDPLELVVSGAAETLSPP---------------------------

* :: : . : *

gal ---------------------------------------

mus LFIFIFILLRRRHRGKFRKDVQKEKDLQLSSGAEEPITR

hum ---------------------------------------

pan ---------------------------------------

1. Glucoside xylosyl transferase

gal MRRFARVALLFLGCGVCSLLYGVSQLALSLEQEAGGARQRQ--ARESAAPGGGRQAGSAD

mus MRRYLRVVGLCLACGFCSLLYAFSQLAVSLEEGAAG-GRRPQAAVVSWLADGGRGTGRGA

hum MRRYLRVVVLCVACGFCSLLYAFSQLAVSLEEGTGGGGGKPQAAVASWLAGGGRGAVRGA

pan MRRYLRVVVLCVACGFCSLLYAFSQLAVSLEEGAGGGGGKPQAAVASWLAGGGRGAVRGA

***: **. * :.**.*****..****:***: :.* : * * .*** : .

gal G------GEEGAGRCKNLSVSFWNSYWMLPSDVCGVNCFWEAAFRYTYMETQPSETMHLA

mus GSAGP----GRTGRCKEFSLSYWNPYWMLPSDVCGMNCFWEAAFRYD-MKTRPDEKMHLA

hum GVAGPAAHPGVSDRCKDFSLCYWNPYWMLPSDVCGMNCFWEAAFRYS-LKIQPVEKMHLA

pan GVAGPAAHPGVSDRCKDFSLCYWNPYWMLPSDVCGMNCFWEAAFRYS-LKIQPVEKMHLA

* :.***::*:.:** **********:********** :: :* *.****

gal VVACGERLEETITMLRSAIIFSIKPLHFHIFAEDQLHESFKDILDDFPYEGKVNYTLYPI

mus VVACGERLEETVTMLKSALIFSIKPLHVHIFAEDQLHDSFKDRLASWSFLRRFDYSLYPI

hum VVACGERLEETMTMLKSAIIFSIKPLQFHIFAEDQLHHSFKGRLDNWSFLQTF**N**YTLYPI

pan VVACGERLEETMTMLKSAIIFSIKPLQFHIFAEDQLHHSFKGRLDNWSFLQTFNYTLYPI

***********:***:**:*******:.*********.***. * .: : .:*:****

gal TFPSEGAKEWKKLFKPCASQRLFLPLILKDVDSLLYVDTDILFLRPVDDIWSFLRKFDST

mus TFPGDSAADWKKLFKPCASQRLFLPLILKEVDSLLYVDTDILFLRPVDDIWSLLKKFNST

hum TFPSENAAEWKKLFKPCASQRLFLPLILKEVDSLLYVDTDILFLRPVDDIWSLLKKF**N**ST

pan TFPSENAAEWKKLFKPCASQRLFLPLILKEVDSLLYVDTDILFLRPVDDIWSLLKKFNST

***.:.* :********************:**********************:*:**:**

gal QIAAMAPEHEEPRIGWYNRFARHPYYGVTGINSGVMLMNMTRIRRKYFKNDMTSVRLRWA

mus QIAAMAPEHEEPRIGWYNRFARHPYYGRTGVNSGVMLMNMTRMRRKYFKNDMTTARLQWG

hum QIAAMAPEHEEPRIGWYNRFARHPYYGKTGVNSGVMLM**N**MTRMRRKYFKNDMTTVRLQWG

pan QIAAMAPEHEEPRIGWYNRFARHPYYGKTGVNSGVMLMNMTRMRRKYFKNDMTTVRLQWG

*************************** **:***********:**********:.**:*.

gal EILMPLLKKYKLNITWGDQDLLNIMFFHNPESLYVFPCQWNYRPDHCIYGSNCKEAEEEG

mus DILMPLLKKYKLNITWGDQDLLNIVFSHNPESLFVFPCQWNYRPDHCIYGSNCREAEEEG

hum DILMPLLKKYKLNITWGDQDLLNIVFFHNPESLFVFPCQWNYRPDHCIYGSNCQEAEEGG

pan DILMPLLKKYKLNITWGDQDLLNIVFFHNPESLFVFPCQWNYRPDHCIYGSNCQEAEEEG

:***********************:* ******:*******************:**** *

gal IFILHGNRGVYHDDKQPTFRAVYEAIKNYSFGDDLVRSLLQPLELELQKTVHTYCGRVYE

mus VFILHGNRGVYHDDKQPAFRAVYEALRNCSLEDDSVRSLLKPLELELQKTVHTYCGKTYK

hum IFILHGNRGVYHDDKQPAFRAVYEALRNCSFEDDNIRSLLKPLELELQKTVHTYCGKIYK

pan IFILHGNRGVYHDDKQPAFRAVYEALRNCSFEDDNIRSLLKPLELELQKTVHTYCGKIYK

:****************:*******::* *: ** :****:***************: *:

gal VFIKQLKKSIKDLSVRRSKGS

mus IFIKQLTKSIRNRYDTPPKER

hum IFIKQLAKSVRDRYARSPKEK

pan IFIKQLAKSVRDRYARSPKEK

:***** **::: *

1. Phosphatidylcholine sterolacyltransferase

gal MGRTGAGFTLLTLL-LLLPQPTSQFWLFNVLFPPTTTPEAPPTNSTPPVVLVPGCLGNQL

hum MGPPGSPWQWVTLLLGLLLPPAAPFWLLNVLFPPHTTPKAELS**N**HTRPVILVPGCLGNQL

pan MGPPGSPWQWVTLLLGLLLPPAAPFWLLNVLFPPHTTPKAELSNHTRPVILVPGCLGNQL

mus MGLPGSPWQRVLLLLGLLLPPATPFWLLNVLFPPHTTPKAELSNHTRPVILVPGCLGNRL

** *: : : ** ** *:: ***:****** ***:* :* * **:********:*

gal EAKLDKPDVVNWMCYRKTEDYFTIWLNLNTFLPVGVDCWIDNTRVVYNRTARKMTNAPGV

hum EAKLDKPDVVNWMCYRKTEDFFTIWLDLNMFLPLGVDCWIDNTRVVY**N**RSSGLVSNAPGV

pan EAKLDKPDVVNWMCYRKTEDFFTIWLDLNMFLPLGVDCWIDNTRVVYNRSSGLVSNAPGV

mus EAKLDKPDVVNWMCYRKTEDFFTIWLDFNLFLPLGVDCWIDNTRIVYNHSSGRVSNAPGV

********************:*****::* ***:**********:***::: ::*****

gal HIRVPGFGKTYSVEYLDQSKLAGYLHTLVQNLVNNGYVRDQTVRAAPYDWRVGPQEQPEY

hum QIRVPGFGKTYSVEYLDSSKLAGYLHTLVQNLVNNGYVRDETVRAAPYDWRLEPGQQEEY

pan QIRVPGFGKTYSVEYLDSSKLAGYLHTLVQNLVNNGYVRDETVRAAPYDWRLEPGQQEEY

mus QIRVPGFGKTESVEYVDDNKLAGYLHTLVQNLVNNGYVRDETVRAAPYDWRLAPHQQDEY

:********* ****:*..*********************:**********: * :* **

gal FQNLKALIEEMHDEYQQRVFLIGHSMGNLNVLYFLLQQKQAWKDQYIGGFISLGAPWGGS

hum YRKLAGLVEEMHAAYGKPVFLIGHSLGCLHLLYFLLRQPQAWKDRFIDGFISLGAPWGGS

pan YRKLAGLVEEMHAAYGKPVFLIGHSLGCLHLLYFLLRQPQAWKDRFIDGFISLGAPWGGS

mus YKKLAGLVEEMYAAYGKPVFLIGHSLGCLHVLHFLLRQPQSWKDHFIDGFISLGAPWGGS

:::* .*:***: * : *******:* *::*:***:* *:***::*.************

gal VKPLRVLASGDNQGIPLMSNIKLREEQRMTTTSPWMFPTSLAWPEDHVFISTPSYNYTYR

hum IKPMLVLASGDNQGIPIMSSIKLKEEQRITTTSPWMFPSRMAWPEDHVFISTPSF**N**YTGR

pan IKPMLVLASGDNQGIPIMSSIKLKEEQRITTTSPWMFPSRMAWPEDHVFISTPSFNYTGR

mus IKAMRILASGDNQGIPILSNIKLKEEQRITTTSPWMLPAPHVWPEDHVFISTPNFNYTVQ

:* : :**********::*.***:****:*******:*: .***********.:*** :

gal DYQRFFTDVNLEDGWYMWEDMKDLLKGLPPPGVDTYCLYGTGYPTVETYIYDEHFPYEDP

hum DFQRFFADLHFEEGWYMWLQSRDLLAGLPAPGVEVYCLYGVGLPTPRTYIYDHGFPYTDP

pan DFQRFFADLHFEEGWYMWLQSRDLLAGLPAPGVEVYCLYGVGLPTPRTYIYDHGFPYTDP

mus DFERFFTDLHFEEGWHMFLQSRDLLERLPAPGVEVYCLYGVGRPTPHTYIYDHNFPYKDP

*::***:*:::*:**:*: : :*** ** ***:.*****.* ** .*****. *** **

gal VDMIYGDGDDTVNKRSSELCKRWRNQQKQKVHVQELRGIDHLNMVFSNLTLTSINEILLG

hum VGVLYEDGDDTVATRSTELCGLWQGRQPQPVHLLPLHGIQHLNMVFS**N**LTLEHINAILLG

pan VGVLYEDGDDTVATRSTELCGLWQGRQPQPVHLLPLHGIQHLNMVFSNLTLEHINAILLG

mus VAALYEDGDDTVATRSTELCGQWQGRQSQPVHLLPMNETDHLNMVFSNKTLEHINAILLG

* :* ****** .**:*** *:.:* * **: :. :******** ** ** ****

gal STKAGAGTKEQGEPKQMGALRSSPEVGRRGHKVLKEPRKN

hum AYRQGPPASPTASPEPPP-----PE---------------

pan AYRQGPPASPTASPEPPP-----PE---------------

mus AYRT--PKSPAASPSPPP-----PE---------------

: : . ..*. **

1. 5-hydroxytryptamine receptor 3B

gal MVLVVAVVLLLLALLPPVSMLQDAVGALQGNPEPDPSTPALLRLSDTLLAHYRRGVRPVR

mus -------------MILLWSCLLVAVVGILGTATPQPGNSSLHRLTRQLLQQYHKEVRPVY

hum --------MLSSVMAPLWACILVA-AGILATDTHHPQDSALYHLSKQLLQKYHKEVRPVY

pan --------MLASVMAPLWACILVA-AGILATDTHHPRDSALYHLSKQLLQKYHKEVRPVY

: : : * .: .. .* :* :*: ** :*:: ****

gal DWRTTTTVAIDVMVYAILSVDEKNQVLTTYIWYRQHWTDEFLQWDPAHFDNITQISLPAE

mus NWAEATTVYLDLCVHAVLDVDVQNQKLKTSVWYREVWNDEFLSWNSSLFDEIQEISLPLS

hum **N**WTKATTVYLDLFVHAILDVDAENQILKTSVWYQEVWNDEFLSW**N**SSMFDEIREISLPLS

pan NWTKATTVYLDLFVRAILDVDAENQILKTSVWYQEVWNDEFLSWNSSMFDEIREISLPLS

:* :*** :*: * *:*.** :** *.* :**:: *.****.*: : **:* :**** .

gal SIWVPDILINEFVDVGKSPDVPYVYVRHHGEVQNLKPIQVVTACSLDIYNFPFDVQNCSL

mus ALWAPDIIINEFVDVERSPDLPYVYVNSSGTIRNHKPIQVVSACSLQTYAFPFDIQNCSL

hum AIWAPDIIINEFVDIERYPDLPYVYV**N**SSGTIENYKPIQVVSACSLETYAFPFDVQ**N**CSL

pan AIWAPDIIINEFVEIERSPDLPYVYVNSSGTIENYKPIQVVSACSLETYAFPFDVQNCSL

::*.***:*****:: : **:*****. * :.* ******:****: * ****:*****

gal TFTSWLHNIHDINLSLWRQPELVKFDRSVFMNQGEWELLYVLSHFQEFSVKSSDSYAEMK

mus TFNSILHTVEDIDLGFLRNREDIENDKRAFMNDSEWQLLSVSSTYHIR-QSSAGDFAQIR

hum TFKSILHTVEDVDLAFLRSPEDIQHDKKAFL**N**DSEWELLSVSSTYSIL-QSSAGGFAQIQ

pan TFKSILHTVEDVDLAFLRSPEDIQHDKKAFLNDSEWELLSVSSTYSIL-QSSAGGFAQIQ

**.* **.:.*::*.: *. * :: *: .*:*:.**:** * * : .*:..:*:::

gal FYVVIRRRPLFYTINLLLPSIFLMVMDIVGFYLPPNSGERVSFKITLLLGYSVFLIIVSD

mus FNVVIRRCPLAYVVSLLIPSIFLMLVDLGSFYLPPNCRARIVFKTNVLVGYTVFRVNMSD

hum FNVVMRRHPLVYVVSLLIPSIFLMLVDLGSFYLPPNCRARIVFKTSVLVGYTVFRV**N**MSN

pan FNVVMRRHPLVYVVSLLIPSIFLMLVDLGSFYLPPNCRARIVFKTSVLVGYTIFRVNMSN

* **:** ** *.:.**:******::*: .******. *: ** .:*:**::* : :*:

gal TLPATAVGTPLIGIYFVVCMALLVISLTETIFIVRLVHKQDLQPHVPSWVKHLLLERATA

mus EVPRSAGCTPLIGVFFTVCMALLVLSLSKSILLIKFLYEERHSGQ------------ERP

hum QVPRSVGSTPLIGHFFTICMAFLVLSLAKSIVLVKFLHDEQRGGQ------------EQP

pan QVPRSVGSTPLIGHFFTICMAFLVLSLAKSIVLVKFLHDEQRGGQ------------EQP

:* :. ***** :*.:***:**:**:::*.::::::.: :

gal LLCIWDRKKFSQSRTQSSDISRHVENNDSTAKLNHYGSEDPREREVAGSTRPTPASATPA

mus LMCLQGDSDAEESRLYLGAPR-----------------ADVTESPVHQEHRV--------

hum FLCLRGDTDADRPRVEPRAQR-----------------AVVTESSLYGEHLA--------

pan FLCLRGDTDADRPRVEPRAQR-----------------AVVTESSLYGEHLA--------

::*: . .. .. * * : .

gal EGSLLIHSILHEITTIRQFLEKRDEFRDVAREWLQVGYVLDVLLFRAYLVAVLAYTITLG

mus -PSDTLKDFWFQFRSINNSLRTRDQIHQKEVEWLAILYRFDQLLFRIYLAVLGLYTVTLC

hum -QPGTLKEVWSQLQSISNYLQTQDQTDQQEAEWLVLLSRFDRLLFQSYLFMLGIYTITLC

pan -QPGTLKEVWSQLQSISNYLQTQDQTDQQEAEWLVLLSRFDRLLFQSYLFMLGIYTITLC

::.. :: :* : *..:*: : *** : :* ***: ** : **:**

gal TLWSVWQYA

mus SLWALWSRM

hum SLWALWGGV

pan SLWALWGGV

:**::*

1. CAM 1

mus MASAVLPSGSQCAAAAAVAAAAAPPGLRLRLLLLLLSAAALIPTGDGQNLFTKDVTVIEG

hum MASVVLPSGSQCAAAA---AAAAPPGLRLRLLLLLFSAAALIPTGDGQNLFTKDVTVIEG

pan MASVVLPSGSQCAAAA---AAAAPPGLRLRLLLLLFSAAALIPTGDGQNLFTKDVTVIEG

gal MAARAQHSGP--------AANGGGAPALLPLLLLLLSAAAIIPRGDGQNLITEDVTVVEG

**: . ** * .. * *****:****:** ******:*:****:**

mus EVATISCQVNKSDDSVIQLLNPNRQTIYFRDFRPLKDSRFQLLNFSSSELKVSLTNVSIS

hum EVATISCQV**N**KSDDSVIQLLNPNRQTIYFRDFRPLKDSRFQLL**N**FSSSELKVSLT**N**VSIS

pan EVATISCQVNKSDDSVIQLLNPNRQTIYFRDFRPLKDSRFQLLNFSSSELKVSLTNVSIS

gal DVATISCRVKNSDDSVIQLLNPNRQTIYFRDFRPLKDSRFQLVNFSNSELRVSLTNVSIS

:******:*::*******************************:***.***:*********

mus DEGRYFCQLYTDPPQESYTTITVLVPPRNLMIDIQKDTAVEGEEIEVNCTAMASKPATTI

hum DEGRYFCQLYTDPPQESYTTITVLVPPRNLMIDIQKDTAVEGEEIEV**N**CTAMASKPATTI

pan DEGRYFCQLYTDPPQESYTTITVLVPPRNLMIDIQKDTAVEGEEIEVNCTAMASKPATTI

gal DEGRYFCQLYTDPPQEIYTTITVLVPPRNLVIDIQKEIAVEGEEIELNCTAMASRPATTI

**************** *************:*****: ********:*******:*****

mus RWFKGNKELKGKSEVEEWSDMYTVTSQLMLKVHKEDDGVPVICQVEHPAVTGNLQTQRYL

hum RWFKGNTELKGKSEVEEWSDMYTVTSQLMLKVHKEDDGVPVICQVEHPAVTGNLQTQRYL

pan RWFKGNTELKGKSEVEEWSDMYTVTSQLMLKVHKEDDGVPVICQVEHPAVTGNLQTQRYL

gal RWFKGNKELTGKSEVEQWSDMYTVTSQLLLKVGREDDGVPVICLVDHPAVK-DLQTQRYL

******.**.******:***********:*** :********* *:****. :*******

mus EVQYKPQVHIQMTYPLQGLTREGDAFELTCEAIGKPQPVMVTWVRVDDEMPQHAVLSGPN

hum EVQYKPQVHIQMTYPLQGLTREGDALELTCEAIGKPQPVMVTWVRVDDEMPQHAVLSGPN

pan EVQYKPQVHIQMTYPLQGLTREGDALELTCEAIGKPQPVMVTWVRVDDEMPQHAVLSGPN

gal EVMYKPQVRVSQNYPVQGLTREGEPLELTCAAFGKPQPTDMRWLRVDDEMPQHVVVSGSN

** *****::. .**:*******: :**** *:*****. : *:*********.*:** *

mus LFINNLNKTDNGTYRCEASNIVGKAHSDYMLYVYDPPTTIPPPTTTTTTTT-TTTTTILT

hum LFI**NN**L**N**KTD**N**GTYRCEASNIVGKAHSDYMLYVYDPPTTIPPPTTTTTTTT-TTTTTILT

pan LFINNLNKTDNGTYRCEASNIVGKAHSDYMLYVYDPPTTIPPPTTTTTT-T-TTTTTILT

gal LLISNLNKTDNGTYRCEASNAVGKSHADYMLFVYDTTATTEPAVHGFTQLPNSAEELDYG

*:*.**************** ***:*:****:*** :* * . * ::

mus IITDSRAGEEGTIGAVDHAVIGGVVAVVVFAMLCLLIILGRYFARHKGTYFTHEAKGADD

hum IITDSRAGEEGSIRAVDHAVIGGVVAVVVFAMLCLLIILGRYFARHKGTYFTHEAKGADD

pan IITDSRAGEEGSIRAVDHAVIGGVVAVVVFAMLCLLIILGRYFARHKGTYFTHEAKGADD

gal DLSDSRAGEEGAIRSVDHAVVGGVVAVVVFAMLCLLIILGRYFARHKGTYFTHEAKGADD

::********:* :*****:***************************************

mus AADADTAIINAEGGQNNSEEKKEYFI

hum AADADTAIINAEGGQNNSEEKKEYFI

pan AADADTAIINAEGGQNNSEEKKEYFI

gal AADADTAIINAEGGQNNSEEKKEYFI

**************************

1. Carboxypeptidase M

gal --MRTGAGEAVGVLCLVSLVAALDYKYHHSEELEAYLKEVHAAYPALTHLHSIGRSVEGR

hum -----MDFPCLWLGLLLPLVAALDFNYHRQEGMEAFLKTVAQ**N**YSSVTHLHSIGKSVKGR

pan -----MDFPCLWLGLLLPLVAALDFNYHHQEGMEAFLKTVAQNYSSITHLHSIGKSVKGR

mus RLVLDMDRARLWLGLLLPVVAALDFRYHHQEGMEAFLKSVAQNYSSITHLHSIGKSVRGR

: : *: :*****:.**:.* :**:** * * ::*******:**.**

gal DLWVLVLGRFPTQHKIGIPEFKYVANMHGDETVGREILLHLIDHLVTNYGRDPVITRLLN

hum NLWVLVVGRFPKEHRIGIPEFKYVANMHGDETVGRELLLHLIDYLVTSDGKDPEITNLI**N**

pan NLWVLVVGRFPKEHRIGIPEFKYVANMHGDETVGRELLLHLIDYLVTSDGKDPEITNLIN

mus NLWVLVVGQTPKEHRVGIPEFKYVANMHGDETVGRELLLHLIDYLVSSYRKDPEITHLID

:*****:*: *.:*::********************:******:**:. :** **.*::

gal NTRIHIMPTMNPDGFEATVVPDCYYSRGRYNKNGEDLNRNFPDAFENNNNLIQPETQAVI

hum STRIHIMPSMNPDGFEAVKKPDCYYSIGRENYNQYDLNRNFPDAFEYN**N**VSRQPETVAVM

pan STRIHIMPSMNPDGFEAVKKPDCYYSIGRENYNQYDLNRNFPDAFEYNNVSRQPETVAVM

mus STRIHIMPSMNPDGFEAVQKPDCYYSNGRENYNNYDLNRNFPDAFENNNVTKQPETLAIM

.*******:********. ****** ** * * *********** ** **** *::

gal NWIKNETFVLSANLHGGALVASYTFDNGNSVTGTSNGYSRSPDDDVFIHLAKTYSFNHAS

hum KWLKTETFVLSANLHGGALVASYPFDNGVQATGALYSRSLTPDDDVFQYLAHTYASRNPN

pan KWLKTETFVLSANLHGGALVASYPFDNGVQATGALYSRSLTPDDDVFQYLAHTYASRNPN

mus EWLKTETFVLSANLHGGALVASYPFDNGVQATGTLLSRSLTPDDDVFQHLAYTYASRNPN

:*:*.****************** **** ..**: . * :****** :** **: .: .

gal MYKGTGCDSKQTFPDGITNGYSWYQLEGGMQDYNYVWGQCFEITLELSCCKYPPAEQLEK

hum MKKGDECKNKMNFPNGVTNGYSWYPLQGGMQDYNYIWAQCFEITLELSCCKYPREEKLPS

pan MKKGDECKNKMNFPNGVTNGYSWYPLQGGMQDYNYIWAQCFEITLELSCCKYPREEKLPS

mus MTKGDQCKNKRNFPNGIINGYSWYPLQGGMQDYNYIWAQCFEITLELSCCKYPREEKLPL

* ** *..* .**:*: ****** *:********:*.*************** *:*

gal FWRDNKVALVEYIKQVHLGVKGQVTDKNGNPIPNAIVEAKGRPHICPYRTNEHGEYFLLL

hum FWNNNKASLIEYIKQVHLGVKGQVFDQNGNPLPNVIVEVQDRKHICPYRTNKYGEYYLLL

pan FWNNNKASLIEYIKQVHLGVKGQVFDQNGNPLPNVIVEVQDRKHICPYRTNKYGEYYLLL

mus FWNDNKASLIEYIKQVHLGVKGQVFDQSGAPLPNVIVEVQDRKHICPFRTNKLGEYYLLL

**.:**.:*:************** *:.* *:**.***.:.* ****:***: ***:***

gal LPGKYVINATVPGFKSMLKTVDIPDNTANFSALKQDFSFPEV----SVKPRPASCPRTPL

hum LPGSYII**N**VTVPGHDPHITKVIIPEKSQ**N**FSALKKDILLPFQGQLDSIPVSNPSCPMIPL

pan LPGSYIINVTVPGHDPHITKVIIPEKSQNFSALKKDILLPFQGQLDSIPVPNPSCPMIPL

mus LPGSYVINVTVPGHDSYLTKLTIPGKSQPFSALKKDFHLPLRWQPDSISVSNPSCPMIPL

***.*:**.****.. :..: ** :: *****:*: :* *: *** **

gal YQELERSSAAVKPTLHILVLVIVTISILK

hum YRNLPDHSAATKPSLFLFLVSLLHIFFK-

pan YRNLPDHSAATKPSLFLFLVSLLHILFK-

mus YKFMPSHSAATKPSLGVFFMTLLYVFFK-

*: : ***.**:* ::.: :: : :

1. Killer cell Ig-like receptor

gal ----MAVALILGWWLVAAS--RAQQLPRPSLSLHPSQGVSLGDNVTLRCHLPQLAAWVWL

mus MLLWFLSLVCSGFFLVQRMSAHVGSHDKPFLSAWPSYVVPLGQNVTLTCDSHRGSNIFKL

hum MSLMVVSMACVGLFLVQRAGPHMGGQDKPFLSAWPSAVVPRGGHVTLRCHYRHRFNNFML

pan MSLMVVSMACVGFFLLQRACPHMGGHDKPFLSAWSSAVVPRGGHVTLRCHYRGGFNNFML

. * :*: : :* ** * * * :*** *. . *

gal YREGRWSYAKYSDKKQDTTEFSFLSTSREHAGTYSCQYQLSES-EDISVMSDPVELVLTD

mus YKEEGSPNHQLHETT-FQKSQVFGPVTTEHAGTYRCF--HPQYANVLSAHSEPLKIIISG

hum YKEDRIHIPIFHGRI-FQESFNMSPVTTAHAG**N**YTCRGSHPHSPTGWSAPSNPVVIMVTG

pan YKEDRIHVPI--GRI-FQESFNMSPVTTAHAGNYTCRGSHPHSPTGWSAPSNPVVIMVTG

*:* . : .: ***.* * . *. *:*: ::::.

gal RSFPPPHISLHPEERVGTGTNVTICCWNKDYGAAFLL--HKDGRSAPIQRLVP---DDVG

mus -IYMKPFLLILQSPLVDTGGNVTLECHSENMFDTYILISHRMGIIKNSVQVSAEHHESGS

hum -NHRKPSLLAHPGPLVKSGERVILQCWSDIMFEHFFL--HKEGISKDPSRLVGQIHDGVS

pan -NHRKPSLLAHPGPLVRSGETVILQCWSDVMFEHFFL--HREGISQDPSRLVGQIHDGVS

. * : * :* * : * .. ::* *: * :: :. .

gal AVSFTLFGVTPADSGTYRCSYRPRNYSFLSSPLGDIVTLEVTP------TAAPPGAELVS

mus HVTYSIGPMTPDLVGTYTCYGANSYYPYEWSDSSDPIDIKITGVYKKPSLSALMGPVLMM

hum KA**N**FSIGPMMLALAGTYRCYGSVTHTPYQLSAPSDPLDIVVTGPYEKPSLSAQPGPKV-Q

pan KANFSIGPMMLALAGTYRCYGSVTHTPYQLSAPSDPLDIMVTGLYEKPSLSAQPGPTV-Q

..::: : *** * : * .* : : :* :* * :

gal CGNLVVAVVRGCAAVLIFALGVFFVIDARSLWIRRDESLGGEGI----------------

mus SGETMAL---SCISDHQFDT----------FHMSREGVPRGQGMPAVQSHSGKFEAKFLL

hum AGESVTL---SCSSRSSYDM----------YHLSREGGAHERRLPAVRKV**N**RTFQADFPL

pan AGENVTL---SCSSRRSYDM----------YHLFREGEAHECRLRAVRKVNRTFQADFPL

.*: :. .* : : : *: :

gal ------------------------------------------------------------

mus SPMIQKGNYRCYGSFRNASHVWSSPSDPLYLPAKGNC----PAYTEADTKTNNYKNLHIL

hum GPATHGGTYRCFGSFRHSPYEWSDPSDPLLVSVTGNPSSSWPSPTEPSSKSGNPRHLHIL

pan GPATHRGTYRCFGSFRHSPYEWSDPSDPLLVSVTGNPSSSWPSPTEPSSKSGILRHLHIL

gal ------------------------------------------------------------

mus TGLLVTMVLVVIIIFYSCYFSKQNKSQKQAAASMEQEYEVKNTINTQNFEGQERQEVTYT

hum IGTSVVIILFILLLFFLLHL---WCSNKKNAAVMDQEPAGNRTANSEDSDEQDPEEVTYA

pan IGTSVVIILFIIILFFLLHR---WCSNKKNAAVMDQEPAGDRTVNRQDSDEQDPQEVTYA

gal --------------------------------------------------------

mus ELEQRVFNQNLMP-PISRISEFSADTIVYMEIMK----------------------

hum QLDHCVFTQRKITRPSQRPKTPPTDTILYTELPNAKPRSKVVSCP-----------

pan QLDHCVFIQRKIGRPSQRPKTPLTDTSVYTELPNAEPRSKVVSCPRAPQSGLEGVF

1. Protein Z protease inhibitor

gal ----------------------------------------MKIRI--YLLLLCELCFEIS

mus ----------------------------------------MRVASSLFLPVLLTEVWLVT

hum ----------------------------------------MKVVPSLLLSVLLAQVWLVP

pan MSRSAQELLGYHCRLQDKLQEQEGSLAAEGRHSLASAADHMKVVPSLLLSVLLAQVWLVP

*:: * :* : :

gal KADIKPKSPKKDKRLNFLGRNKNVSISEEWHQHK---------------NDHKPLEEQSF

mus SFNLSSHSPEASVHLESQD-----YENQTWEEYTRTDPREEEEEEE---EKEEGKDEEYW

hum GLAPSPQSPETPAPQ**N**QTS-------------RVVQAPKEEEEDEQEASEEKASEEEKAW

pan GLAPSPQSPETPAPQNQTS-------------RVVQAPREEEEDEQEASEEKASEEEKAW

. :**: : . :.. :*: :

gal EELTLHNFTEKTANFGFNLYRKIAMKLDNNIIISPLSVTTLMATYLLAAEGETHRQIAKA

mus L-RASQQLSNETSSFGFNLLRKISMRHDGNVIFSPFGLSVAMVNLMLGTKGETKVQIENG

hum LMASRQQLAKETSNFGFSLLRKISMRHDGNMVFSPFGMSLAMTGLMLGATGPTETQIKRG

pan LMASRQQLAKETSNFGFSLLRKISMRHDGNMVFSPFGVSLAMTGLMLGASGPTETQIKRG

: ::::::*:.***.* ***:*: *.*:::**:.:: *. :*.: * *. ** ..

gal LNLHSLKDRDRHYLPALFKQLKDNITTNEELLFVQGILSFIQKDFTVREAFLNLSKQYFD

mus LNLQALSQAGPLILPALFKKVKETFSSNRDLGLSQGSFAFIHKDFDIKETYFNLSKKYFD

hum LHLQALKPTKPGLLPSLFKGLRETLSRNLELGLTQGSFAFIHKDFDVKETFF**N**LSKRYFD

pan LHLQALKPTKPGLLPSLFKGLRETLSHNLELGLTQGSFAFIHKDFDVIETFFNLSKRYFD

*:*::*. **:*** :::.:: * :* : ** ::**:*** : *:::****:***

gal MEFLCVDFQNSTQAKFVINQNIKQRTKGKISELFEEVDRHSKLLLLDYIFFKGKWLYPFN

mus IEYVSINFQNSSQARGLINHCIVKETEGKIPKLFDEINPETKLILVDYVLFKGKWLTPFD

hum TECVPMNFR**N**ASQAKRLMNHYINKETRGKIPKLFDEINPETKLILVDYILFKGKWLTPFD

pan TECVPMNFRNASQAKRLMNHYINKETGGKIPKLFDEIDPETKLILVDYILFKGKWLTPFD

* : ::*:*::**: ::*: * :.* *** :**:*:: .:**:*:**::****** **:

gal SEFTEIETFHINKYRSVQVPMMFKSDKVNSTYDENLRCNVIKLPYKGKAYMLIVIPEKGE

mus PSFTEADTFHLDKYRAIKVPMMYREGNFTSTFDKKFRCHILKLPYQGNATMLVVLMEKTG

hum PVFTEVDTFHLDKYKTIKVPMMYGAGKFASTFDKNFRCHVLKLPYQG**N**ATMLVVLMEKMG

pan PVFTEVDTFHLDKYKTIKVPMMYGAGKFASTFDKNFRCHVLKLPYQGNATMLVVLMEKMG

*** :***::**::::****: .:. **:*:::**:::****:*:* **:*: **

gal DYVSLEDHLTMELVESWLANMKSRNMDISFPKFKLEQKYKMKKLLYALGIKNLFARTADL

mus DYLALEDYLTVDLVETWLQNMKTRKMEVFFPKFKLNQRYEMHELLKQMGIRRLFSTSADL

hum DHLALEDYLTTDLVETWLRNMKTRNMEVFFPKFKLDQKYEMHELLRQMGIRRIFSPFADL

pan DHLALEDYLTTDLVETWLRNMKTRNMEVFFPKFKLDQKYEMHELLRQMGIRRIFSPFADL

*:::***:** :***:** ***:*:*:: ******:*:*:*::** :**:.:*: ***

gal SHLTD-QKHLTVSQVVQKAVIEVDEKGTEAAAATGSEIIAFSVPPVLKVDRPFLFMIFEE

mus SELSAMARNLQVSRVLQQSVLEVDERGTEAVSGTLSEIIAYSMPPAIKVNRPFHFIIYEE

hum SELSATGRNLQVSRVLQRTVIEVDERGTEAVAGILSEITAYSMPPVIKVDRPFHFMIYEE

pan SELSATGRNLQVSRVLQRTVIEVDERGTEAVAGILSEITAYSMPPVIKVDRPFHFMIYEE

*.*: ::* **:*:*::*:****:****.:. *** *:*:**.:**:*** *:*:**

gal TFKTLLFIGRVVDPTET

mus MSRMLLFLGRVVNPTVL

hum TSGMLLFLGRVVNPTLL

pan TSGMLLFLGRVVNPTLL

***:****:**

1. Tapasin

gal MAAGLRLLLAGLCWSQFRVEDAASPPPPPAPVRCALLEGVGRGGGLPGGGNARPALLRFG

hum -MKSLSLLLA-VALGLAT-----AVSAGPAVIECWFVEDASGKGLA----KRPGALLLRQ

pan -MKSLSLLLA-VALGLAT-----AVSAGPAVIECWFVEDASGKGLA----KRPGALLLRQ

mus -------LVA-VALGLATV--VSVVSAGPEAIECWFVEDAGGGGLS----KKPATLLLRH

*:* :. . * :.* ::*... * : :**

gal GDAETPPEPGPEPEVTFNVSDPWGTLTPLGV---PPRTPPSCELNPTNPQTGSDPWSRPL

hum GPGEPPPRPDLDPELYLSVHDPAGALQAAFRRYPRGAPAPHCEMSRFVPLPASAKWASGL

pan GPGEPPPRPDLDPELYLSVHDPAGALQAAFRRYPRGAPAPHCEMSRFVPLPASAKWASGL

mus GPRGPPPRPDLDPKLYFKVDDPAGMLLAAFRRYPAGASAPHCEMSRFIPFPASAKWARSL

* **.*. :*:: :.* ** * * * **:. * .* *: *

gal HPDARSPPTAGGHWWVAAVGTPQYSVTALLQGGMGTEG----TITAAVALAVLTHTPTLR

hum TPAQNCPRALDGAWLMVSISSPVLSLSSLLRPQPEPQQEPVLITMATVVLTVLTHTPAPR

pan TPAQNCPRALDGAWLMVSISSPVLSLSSLLRPQPEPQQEPVLITMATVVLTVLTHTPAPR

mus SPEQNCPRALDGDWLLVSVSSTLFSLSSLLRPQPEPLREPVVITMATVVLTVLTHNPAPR

* ..* : .* * :.::.: *:::**: *:*.*:****.*: *

gal ARVGSPIHLHCAFA-------------APPSPFVLEWRHQNRGAGRVLLAYDSSTA-RAP

hum VRLGQDALLDLSFAYMPPTSEAASSLAPGPPPFGLEWRRQHLGKGHLLLAATPGLNGQMP

pan VRLGQDALLDLSFAYMPPTSEAASSLAPGPPPFGLEWRRQHLGKGHLLLAATPGLNGQMP

mus VQLGKDAVLDLRFAYAPSALEGSPSLDVGPPPFGLEWRRQHRGKGHLLLAATPGLAGRMP

.::*. *. ** * ** ****:*: * *::*** . : *

gal RATPGAELLLGTRDG----DGVTAVTLRLARPSPGDEGTYICSVFLPHGHTQTVLQLHVF

hum AAQEGAVAFAAWDDDEPWGPWTG**N**GTFWLPRVQPFQEGTYLATIHLPYLQGQVTLELAVY

pan AAQEGAVAFAAWDDDEPWGPWTGNGTFWLPRVQPFQEGTYLATIHLPYLQGQVTLELAVY

mus PAQEKATAFAAWDDDEPWGPWTGNGTFWLPAVKPSQEGVYLATVHLPYLQGQVSLELTVH

* * : . *. . *: * .* :**.*:.::.**: : *. *:* *.

gal EPPKVTLSPKNLVVAP--GTSAELRCHVSGFYPLD-VTVTWQRRAGGSGTSQSPRDTVMD

hum KPPKVSLMPATLARAAPGEAPPELLCLVSHFYPSGGLEVEWELRGGPGGRSQK---AEGQ

pan KPPKVSLMPATLARAAPGEAPPELLCLVSHFYPSGGLEVEWELRGGPGGRSQK---AEGQ

mus KAPRVSLTPAPVVWAAPGEAPPELLCLASHFFPSEGLEVKWELRGGPGGSSRK---VEGK

: *:*:* * :. * : ** * .* *:* : * *: *.* .* *:. . .

gal SWTSGHRQAADGTYSRTAAARLIPARPQHHGDVYSCVVTHIALAKPMR-VSVRLLLAGTE

hum RWLSALRHHSDGSVSLSGHLQPPPVTTEQHGARYACRIHHPSLPASGRSAEVTLEVAGLS

pan RWLSALRHHSDGSVSLSGHLQPPPVTTEQHGARYACRIHHPSLPASGRSAEVTLEVAGLS

mus TWLSTIRHHSDGSVSQSGHLQLPPVTAKQHGVHYVCRVYHSSLPASGRSADVTLEVAGFS

* * *: :**: * :. : *. ::** * * : * :* * ..* * :** .

gal GPHLEDITGLFLVAFVLCGLIR---WLYPKAARPKEETKKSQ

hum GPSLEDSVGLFLSAFLLLGLFKALGWAAVYLSTCKDSKKKAE

pan GPSLEDSVGLFLSAFLLLGLFKALGWAAVYLSTCKDSKKKAE

mus GPSIEDGIGLFLSAFLLLGLLKVLGWL---------------

** :** **** **:* **:: *

1. Retinoid inducible serine carboxypeptidase

gal MG--AL-----RAAVGLALFAALAAGAA----LEPPRELWGYVQVRSKAHIFWWLYYADS

mus MELSRRICLVRLWLLLLSFLLGFSAGSAIDWREPEGKEVWDYVTVRKDAHMFWWLYYATN

hum MELALRRSPVPRWLLLLPLLLGLNAGAVIDWPTEEGKEVWDYVTVRKDAYMFWWLYYATN

pan MELALRRSPVPRWLLLLPLLLGLNAGAVIDWPTEEGKEVWDYVMVRKDAYMFWWLYYATN

* : * :: .: **:. :*:*.** **..*::******* .

gal RAGGFTELPLILWLQGGPGSSGCGFGNFEEIGPLDKELKPRNTTWLQAASILFVDNPVGT

mus PCKNFSELPLVMWLQGGPGGSSTGFGNFEEIGPLDTQLKPRNTTWLQWASLLFVDNPVGT

hum SCK**N**FSELPLVMWLQGGPGGSSTGFGNFEEIGPLDSDLKPRKTTWLQAASLLFVDNPVGT

pan SCKNFSELPLVMWLQGGPGGSSTGFGNFEEIGPLDSDLKPRKTTWLQAASLLFVDNPVGT

. .*:****::*******.*. ************.:****:***** **:*********

gal GFSYVDDCSLFAKNLTTVVSDMMVFLKEFFTHRTEFQSIPFYIFSESYGGKMAAGIALEL

mus GFSYVNTTDAYAKDLDTVASDMMVLLKSFFDCHKEFQTVPFYIFSESYGGKMAAGISVEL

hum GFSYV**N**GSGAYAKDLAMVASDMMVLLKTFFSCHKEFQTVPFYIFSESYGGKMAAGIGLEL

pan GFSYVNGSGAYAKDLAMVASDMMVLLKTFFNCHKEFQTVPFYIFSESYGGKMAAGIGLEL

*****: . :**:* *.*****:** ** :.***::*****************.:**

gal HNAVQKGSIKCNFAGVALGDSWISPLDSVLSWGPYLYSTSLLDDHGLAEVTAVAKEIMDA

mus YKAVQQGTIKCNFSGVALGDSWISPVDSVLSWGPYLYSMSLLDNQGLAEVSDIAEQVLDA

hum YKAIQRGTIKCNFAGVALGDSWISPVDSVLSWGPYLYSMSLLEDKGLAEVSKVAEQVLNA

pan YKAIQRGTIKCNFAGVALGDSWISPVDSVLSWGPYLYSMSLLEDKGLAEVSKVAEQVLNA

::*:*:*:*****:***********:************ ***:::*****: :*:::::*

gal INKNEYGLATELWSKAEGIIEENTDNVNFYNIMTKEVPEMKADEQE---NFHLVRLYQRH

mus VNKGFYKEATQLWGKAEMIIEKNTDGVNFYNILTKSSPEKAMESSLEFLRSPLVRLCQRH

hum VNKGLYREATELWGKAEMIIEQNTDGVNFYNILTKSTPTSTMESSLEFTQSHLVCLCQRH

pan VNKGLYREATELWGKAEMIIEQNTDGVNFYNILTKSTPTSTMESSLEFTQSHLVCLCQRH

:**. * **:**.*** ***:***.******:**. * :.. . ** * ***

gal VKIMHKNNLNELMNGPIRKKLKVIPDCVKWGGQSTKVFENMAEDFMRPVIDIVDQLLAAN

mus VRHLQGDALSQLMNGPIKKKLKIIPEDISWGAQASYVFLSMEGDFMKPAIDVVDKLLAAG

hum VRHLQRDALSQLMNGPIRKKLKIIPEDQSWGGQATNVFVNMEEDFMKPVISIVDELLEAG

pan VRHLQRDALSQLMNGPIRKKLKIIPEDQSWGGQAANVFVNMAEDFMKPVISIVDELLEAG

*: :: : *.:******:****:**: .**.*:: ** .* ***:*.*.:**:** *.

gal VNVTVYNGQLDLIVDTMGQEAWIRKLKWPGLKEFSQQRWKALYVSPESTDTAAFHKAYEN

mus VNVTVYNGQLDLIVDTIGQESWVQKLKWPQLSKFNQLKWKALYTDPKSSETAAFVKSYEN

hum I**N**VTVYNGQLDLIVDTMGQEAWVRKLKWPELPKFSQLKWKALYSDPKSLETSAFVKSYKN

pan INVTVYNGQLDLIVDTMGQEAWVRKLKWPELPKFSQLKWKALYSDPKSLETSAFVKSYKN

:***************:***:*::***** * :*.* :***** .*:* :*:** *:*:*

gal FAFFWILKAGHMVPSDQGEMALKMVRMVTQQKH

mus LAFYWILKAGHMVPSDQGEMALKMMKLVTKQE-

hum LAFYWILKAGHMVPSDQGDMALKMMRLVTQQE-

pan LAFYWILKAGHMVPSDQGNMALKMMRLVTQQD-

:**:**************:*****:::**:*.

1. Bis(5'-adenosyl)-triphosphatase

gal -----------------MNLQIVLFVSAILS-C-CARSGSSAPRLLLVSFDGFRADYLET

hum -----------------MKLLVILLFSGLITGFRSDSSSSLPPKLLLVSFDGFRADYLKN

pan -----------------MKLLVILLFSGLITGFRSDSSSSLPPKLLLVSFDGFRADYLKN

mus MSTVALGALAAVCNMFNMKILVIPLFWGLVTGYKGNSSDSSAPRLLLVSFDGFRADYLKS

*:: :: :. .::: *.* *:**************:.

gal YSLPHLQELIEDGVLVKQVTNAFITKTFPNHYSIVTGLYEESHGIVANDMYDPDAKKKFS

hum YEFPHLQNFIKEGVLVEHVKNVFITKTFPNHYSIVTGLYEESHGIVANSMYDAVTKKHFS

pan YEFPHLQNFIKEGVLVEHVKNVFITKTFPNHYSIVTGLYEESHGIVANSMYDAVTKKHFS

mus YDLPHLQNFIKEGVLVEHVKNVFITKTFPNHYSIVTGLYEESHGIVANSMYDSVTKKHFS

*.:****::*::****::*.*.**************************.*** :**:**

gal QFNDSDPFWWNEAVPIWVTNQQQRNGTSAAAMWPGTDVRINNTTPQFFMKYNFSVTFEER

hum DSNDKDPFWWNEAVPIWVTNQLQENRSSAAAMWPGTDVPIHDTISSYFMNY**N**SSVSFEER

pan DSNDKDPFWWNEAVPIWVTNQLQENRSSAAAMWPGTDVPIHDTISSYFMNYNSSVSFDER

mus ESNDKDPFWWNGAEPIWVTNQLQENRSSAAAMWPGTDVPIHNITASYFMNYSSSVSFKER

: **.****** * ******* *.* :*********** *:: .:**:*. **:*.**

gal VEKIIRWLNSSDPVVNFATLYWEEPDASGHKYGPDDTENMRRVLEQVDQHVGFLTGKLKA

hum LN**N**ITMWLNNSNPPVTFATLYWEEPDASGHKYGPEDKENMSRVLKKIDDLIGDLVQRLKM

pan LNNITMWLNNSNPPVTFATLYWEEPDASGHKYGPEDKENMSRVLKKIDDLIGDLVQRLKM

mus LGNVTTWLSSSNPPVTFAALYWEEPDVSGHKYGPEDKENMRRVLKEVDDLIGDIVLKLKV

: :: **..*:* *.**:*******.*******:*.*** ***:::*: :* :. :**

gal SGLWDTINVIITSDHGMAQCSPKKLIVLDNCIGRGNYTLIDRSPVAAVLPKNNKEYVYNL

hum LGLWENLNVIITSDHGMTQCSQDRLINLDSCIDHSYYTLIDLSPVAAILPKI**N**RTEVYNK

pan LGLWENLNVIITSDHGMTQCSQDRLINLDSCIDHSYYTLIDLSPVAAILPKINRTEVYNK

mus LGLWDSLNVIITSDHGMAQCSKNRLIDLDSCIDRSNYSVIDLTPVAAILPKINVTEVYDK

***:.:**********:*** .:** **.**.:. *::** :****:*** * **:

gal LKQCDDRHMKVYLKEEIPDRFHYRHNKRIQPIILIADEGWTIVQNEALSKLGDHGYDNTL

hum LKNCS-PHMNVYLKEDIPNRFYYQHNDRIQPIILVADEGWTIVLNESSQKLGDHGYDNSL

pan LKNCS-PHMNVYLKEDIPNRFYYQHNDRIQPIILVADEGWTIVLNESSQKLGDHGYDNSL

mus LKRCN-PHMNVYLKEAIPNRFYYQHSSRIQPIILVAEEGWTITLNKSSFKLGDHGYDNSL

**.*. **:***** **:**:*:*..*******:*:*****. *:: *********:*

gal PSMHPFLAAHGPAFRRGYQQSMINNVDIYPMMCHILGLTPQPHNGTLSHTKCLLADQWCI

hum PSMHPFLAAHGPAFHKGYKHSTINIVDIYPMMCHILGLKPHPN**N**GTFGHTKCLLVDQWCI

pan PSMHPFLAAHGPAFHKGYKHSTINIVDIYPMMCHILGLKPHPNNGTFGHTKCLLVDQWCI

mus PSMHPFLAAHGPAFRKGYRQSTINTVDIYPMMCHILGLKPHPNNGTLSHTKCLLVDQWCI

**************::**::* ** *************.*:*:***:.******.*****

gal HLPEAIGIVIGGLLVLTTFTCIVIIITKSRVASPRPFSRLQLQSDDDDPLIE

hum NLPEAIAIVIGSLLVLTMLTCLIII-MQNRLSVPRPFSRLQLQEDDDDPLIG

pan NLPEAIAIVIGSLLVLTMLTCLIII-MQNRLSVPRPFSRLQLQEDDDDPLIG

mus NLPEAIGIVVSALLVLTMLTGLMIF-MRSRASTSRPFSRLQLQEDDDDPLID

:*****.**:..***** :* ::*: :.* : *********.*******

1. Fibrinogen gamma chain

gal MAPGEMGPKPGRWVPLGHLLSLLFSTSMAYIATRENCCILDERFGSYCPTTCGIADFFNK

mus ---MSWSLQPPSFLLCCLL-LLFSPTGLAYVATRDNCCILDERFGSFCPTTCGIADFLSS

hum ---MSWSLHPRNLILYFYALLFLSSTCVAYVATRDNCCILDERFGSYCPTTCGIADFLST

pan ---MSWSLHPRNLILYFYALLFLSSTCVAYVATRDNCCILDERFGSYCPTTCGIADFLST

. . :* : :: * :**:***:***********:**********:..

gal YRLTTDGELLEIEGLLQQATNSTGSIEYLIQHIKTIYPSEKQTLPQSIEQLTQKSKKIIE

mus YQTDVDNDLRTLEDILFRAENRTTEAKELIKAIQVYYNPDQPPKPGMIDSATQKSKKMVE

hum YQTKVDKDLQSLEDILHQVE**N**KTSEVKQLIKAIQLTYNPDESSKPNMIDAATLKSRKMLE

pan YQTKVDKDLQSLEDILHQVENKTSEVKQLIKAIQLTYNPDESSKPNMIDAATMKSRKMLE

*: .* :* :*.:* :. * * . : **: *: * :: * *: * **:*::*

gal EIIRYENTILAHENTIQQLTDMHIMNSNKITQLKQKIAQLESHCQEPCKDTAEIQETTGR

mus EIVKYEALLLTHETSIRYLQEIYNSNNQKITNLKQKVAQLEAQCQEPCKDSVQIHDTTGK

hum EIMKYEASILTHDSSIRYLQEIYNSNNQKIVNLKEKVAQLEAQCQEPCKDTVQIHDITGK

pan EIMKYEASILTHDTSIRYLQEIYNSNNQKIVNLKEKVAQLEAQCQEPCKDTVQIHDITGK

**::** :*:*:.:*: * ::: *.:**.:**:*:****::*******:.:*:: **:

gal DCQDIANKGARKSGLYFIKPQKAKQSFLVYCEIDTYGNGWTVLQRRLDGSEDFRRNWVQY

mus DCQEIANKGAKESGLYFIRPLKAKQQFLVYCEIDGSGNGWTVLQKRIDGSLDFKKNWIQY

hum DCQDIANKGAKQSGLYFIKPLKANQQFLVYCEIDGSGNGWTVFQKRLDGSVDFKKNWIQY

pan DCQDIANKGAKQSGLYFIKPLKANQQFLVYCEIDGSGNGWTVFQKRLDGSVDFKKNWIQY

***:******::******:* **:*.******** ******:*:*:*** **::**:**

gal KEGFGHLSPDDTTEFWLGNEKIHLITTQSTLPYALRIELEDWSGKKGTADYAVFKVGTEE

mus KEGFGHLSPTGTTEFWLGNEKIHLISMQSTIPYALRIQLKDWNGRTSTADYAMFRVGPES

hum KEGFGHLSPTGTTEFWLGNEKIHLISTQSAIPYALRVELEDWNGRTSTADYAMFKVGPEA

pan KEGFGHLSPTGTTEFWLGNEKIHLISTQSAIPYALRVELEDWNGRISTADYAMFKVGPEA

********* .**************: **::*****::*:**.*: .*****:*:** *

gal DKYRLTYAYFIGGEAGDAFDGFNFGDDPSDKSYTYHNGMRFSTFDNDNDNFEGNCAEQDG

mus DKYRLTYAYFIGGDAGDAFDGYDFGDDPSDKFFTSHNGMQFSTWDNDNDKFEGNCAEQDG

hum DKYRLTYAYFAGGDAGDAFDGFDFGDDPSDKFFTSH**N**GMQFSTWDNDNDKFEGNCAEQDG

pan DKYRLTYAYFAGGDAGDAFDGFDFGDDPSDKFFTSHNGMQFSTWDNDNDKFEGNCAEQDG

********** **:*******::******** :* ****:***:*****:**********

gal SGWWMNRCHAGHLNGQYYIGGVYSRDTGTNSYDNGIIWATWRDRWYSMKKTTMKIIPFNR

mus SGWWMNKCHAGHLNGVYHQGGTYSKSSTTNGFDDGIIWATWKSRWYSMKETTMKIIPFNR

hum SGWWMNKCHAGHLNGVYYQGGTYSKASTPNGYDNGIIWATWKTRWYSMKKTTMKIIPFNR

pan SGWWMNKCHAGHLNGVYYQGGTYSKASTPNGYDNGIIWATWKTRWYSMKKTTMKIIPFNR

******:******** *: **.**: : *.:*:*******: ******:**********

gal LSIDG--QQHSGGLKQVGDS----------------

mus LSIGEGQQHHMGGSKQVSVDHEVEIEY---------

hum LTIGEGQQHHLGGAKQVRPEHPAETEYDSLYPEDDL

pan LTIGEGQQHHLGGAKQVRPEHPVETEYDSLYPEDDL

*:*. *:* ** *** .

1. Serine incorporator

gal MGGVLGLCSMASWIPCLCGSAPCLLCRCCPSGNNSTITRLIYAFFLLLGVSVACVMLIPG

hum MGSVLGLCSMASWIPCLCGSAPCLLCRCCPSGNNSTVTRLIYALFLLVGVCVACVMLIPG

pan MGSVLGLCSMASWIPCLCGSAPCLLCRCCPSGNNSTVTRLIYALFLLVGVCVACVMLIPG

mus MGSVLGLCSVASWIPCLCGSAPCLLCRCCPSGNNSTVTRLIYALFLLVGVCVACVMLIPG

**.******:**************************:******:***:**.*********

gal MEEQLKKIPGFCDGGMGTTIPGVHGHVNCDVLVGYKAVYRVCFGMAMFFLLFSLLMIKVK

hum MEEQLNKIPGFCENEK--------GVVPCNILVGYKAVYRLCFGLAMFYLLLSLLMIKVK

pan MEEQLNKIPGFCENEK--------GVVPCNILVGYKAVYRLCFGLAMFYLLLSLLMIKVK

mus MEEQLNKIPGFCENEK--------GVVPCNILVGYKAVYRLCFGLAMFYLLLSLLMIKVK

*****:******:. * * *::*********:***:***:**:********

gal SSNDPRAAVHNGFWFFKFATALAISVGAFFIPEGPFTTVWFYVGMSGAFCFILIQLVLLI

hum SSSDPRAAVHNGFWFFKFAAAIAIIIGAFFIPEGTFTTVWFYVGMAGAFCFILIQLVLLI

pan SSSDPRAAVHNGFWFFKFAAAIAIIIGAFFIPEGTFTTVWFYVGMAGAFCFILIQLVLLI

mus SSSDPRAAVHNGFWFFKFATAVAIIIGAFFIPEGTFTTVWFYVGMAGAFCFILIQLVLLI

**.****************:*:** :******** **********:**************

gal DFAHSWNESWVEKMEEGNSRCWYAALLSATAVNYLLSLVAIVLFYVYYTHPEGCSENKTF

hum DFAHSWNESWVEKMEEGNSRCWYAALLSATALNYLLSLVAIVLFFVYYTHPASCSENKAF

pan DFAHSWNESWVEKMEEGNSRCWYAALLSATALNYLLSLVAIVLFFVYYTHPASCSENKAF

mus DFAHSWNESWVEKMEEGNSRCWYAALLSATALNYLLSLVAVVLFFVYYTHPASCAENKAF

*******************************:********:***:****** .*:***:*

gal ISVNMLLCIGASVMSILPRIQESQPRSGLLQSSVITIYTMYLTWSAMTNEPDRRCNPSLL

hum ISVNMLLCVGASVMSILPKIQESQPRSGLLQSSVITVYTMYLTWSAMTNEPETNCNPSLL

pan ISVNMLLCVGASVMSILPKIQESQPRSGLLQSSVITVYTMYLTWSAMTNEPETNCNPSLL

mus ISVNMLLCIGASVMSILPKIQESQPRSGLLQSSVITVYTMYLTWSAMTNEPETNCNPSLL

********:*********:*****************:**************: .******

gal SIIGYNTT-TIPTQGQVVQWWDAQGIVGLILFLLCVLYSSIRTSNNSQVNKLMLTSDEST

hum SIIGY**N**TTSTVPKEGQSVQWWHAQGIIGLILFLLCVFYSSIRTSNNSQVNKLTLTSDEST

pan SIIGYNTTSTVPKEGQSVQWWHAQGIIGLILFLLCVFYSSIRTSNNSQVNKLTLTSDEST

mus SIIGFNTTRPIPKDGQSVQWWHPQGIIGLVLFLLCVFYSSIRTSNNSQVNKLTLTSDEST

****:*** :*.:** ****. ***:**:******:*************** *******

gal LIEDGMPRNDGSLDDGDDVHRAIDNERDGVTYSYSFFHFMLFLASLYIMMTLTNWYSPDS

hum LIEDGGARSDGSLEDGDDVHRAVDNERDGVTYSYSFFHFMLFLASLYIMMTLTNWYRYEP

pan LIEDGGARSDGSLEDGDDVHRAVDNERDGVTYSYSFFHFMLFLASLYIMMTLTNWYRYEP

mus LIEDGNGRSDGSLDDGDGIHRAVDNERDGVTYSYSFFHFMLFLASLYIMMTLTNWYRYEP

***** *.****:***.:***:********************************* :

gal TYETMTSKWPSVWVKISSSWIGIVLYVWTLVAPLVLTNRDFD

hum S-REMKSQWTAVWVKISSSWIGIVLYVWTLVAPLVLTNRDFD

pan S-REMKSQWTAVWVKISSSWIGIVLYVWTLVAPLVLTNRDFD

mus S-REMKSQWTAVWVKISSSWIGLVLYVWTLVAPLVLTNRDFD

: . *.*:* :***********:*******************

1. Natural cytotoxixity triggering ligand receptor

hum ------------------------------------------------------------

pan ------------------------------------------------------------

gal MVLPAVWSRAWFQLVFPSIMVCRRPFLRGSMILGLILCCCMLAVLAGTVEGLTPV---PE

mus MG----LEPSWYLL-----------------------LCLAVSGAAGTDPPTAPTTAERQ

hum ------------------------------------------------------------

pan --------MC------------KSAGW--KSRLCLRQLF---------------------

gal LRRVDVVLGCSYVWEGGLSRAFG--GSEHPATLVLRGLSVTDDGTLGDVTDYEIPQA---

mus RQPTDIILDCFLVTEDRHRGAFASSGDRERALLVLKQVPVLDDGSLEGITDFQGSTETKQ

hum --------------------------------------------------MTWRAAA---

pan ----------TQQRSPPALGFYPAACSHSAKKKNT----------TAAAAMAWRAAA---

gal ------------------------------------------------------------

mus DSPVIFEASVDLVQIPQAEALLHADCSGKAVTCEISKYFLQARQEATFEKAHWFISNMQV

hum ------------------STCAALLILLWALTTEGDLK--VEMM--A-GGTQITPLND**N**V

pan ------------------STCAALLILLWALTTAGDLK--VEMM--A-GGTQITPLNDNV

gal ---------------------------DHSSSPPIIFEASVEFQSSSNNTSLRTRLGSSI

mus SRGGPSVSMVMKTLRDAEVGAVRHPTLNLPLSAQGTVKTQVEFQVTSETQTLNHLLGSSV

: .: **: : : *...:

hum TIFCNIFYSQPL**N**ITSMGITWFWKSLTFDKEVKVFEF-FGDHQEAFRPGAIVSPW-RLKS

pan TIFCNIFYSQPLNITSMGITWFWKSLTFDKEVKVFEF-FGDHQEAFRPGAIVSPW-RLKS

gal TLDCHFALAPSFLLS--SLEWRRQHR--GSGRSLFRYHVGNAGLTAQPKVHVDVEQLLGN

mus SLHCSFSMAPGLDLT--GVEWRLQHK--GSGQLVYSWKTGQGQA-KRKGATLEPEELLRA

:: * : : : :: .: * : .. :: : *: : . :. *

hum GDASLRLPGIQLEEAGEYRCEVVVTPLKAQGTVQLEVVASPASRLLLDQVGMKEN-EDKY

pan GDASLRLPGIQLEEAGEYRCEVVVTPLKAQGTVQLEVVASPASRLFLDQVGVKEN-EGKY

gal GDASLTLQEATVNDEGTYICLVSTAQHQVQHNIQLLVSEPPRVRVFPTEASLKRDETITL

mus GNASLTLPNLTLKDEGNYICQISTSLYQAQQIMPLNILAPPKVQLHLA----NKDPLPSL

*:*** * ::: * * * : .: :.* : * : * :: :.: .

hum MCESSGFYPEAI**N**ITWEKQTQKFPHPIEISEDVITGPTIKNMDGTF**N**VTSCLKL**N**SSQED

pan MCESSGFYPEAINITWEKQTQKFPHPIEISEDVITGPTIKNMDGTFNVTSCLKLNSSQED

gal TCNIAGYYPLDISVSWTQKTPEDEVEISPSNTRFS-SHRQSQDGTYSINSYLSVNLATAQ

mus VCSIAGYYPLDVGVTWIREELG-GIPAQVSGASFS-SLRQSTMGTYSISSTVMAD--PGP

*. :*:** :.::* :: . * :: :. **:.:.* : :

hum PGTVYQCVVRHASLHTPLRS**N**FTLTAARHSLSETEKTD**N**FSIHWWPISFIGVGLVLLIVL

pan PGTVYQCVVRHASLHTPLRSNFTLTAARHSLSETEKTDNFSIHWWPISFIGVGLVLLIVL

gal APATYTCHVSHVALEAPISISTHLKAPEHTEL----EGLVGGAIATAIFVSVLFI---V-

mus TGATYTCQVAHVSLEEPLTTSMRV--LPNPEQ----RGTLGVIFASIIFLSALLL---F-

:.* * * *.:*. *: . : : . .. *:.. :: .

hum IPWKKICNKSSSAYTPLKCILKHWNSFDTQTLKKEHLIFFCTRAWPSYQLQDGEAWPPEG

pan IPWKKICNKSS-------------------------------------------------

gal LRRKR--AAEPKPEQLLTAS----E-----------------------------------

mus LGLHRQQASSSRSTRPMRHS----G-----------------------------------

: :: .

hum SVNINTIQQLDVFCRQEGKWSEVPYVQAFFALRDNPDLCQCCRIDPALLTVTSGKSIDDN

pan ------------------------------------------------------------

gal ------------------------------------------------------------

mus ------------------------------------------------------------

hum STKSEKQTPREHSDAVPDAPILPVSPIWEPPPATTSTTPVLSSQPPTLLLPLQ

pan -----------------------------------------------------

gal -----------------------------------------------------

mus -----------------------------------------------------

1. Protein disulphide isomerase

gal MRSEGRSARRRAVSPSGRARSPVNMAAMGGRQ----QCLWAAVVALALASEAAFVEDLDE

mus ------------------------MANAVGRRSWAALRLCAAVILLDLAVCKGFVEDLNE

hum ---------------------------MAAWKSWTALRLCATVVVLDMVVCKGFVEDLDE

pan ---------------------------MAAWKSWTALRLCATVVVLDMVVCKGFVEDLDE

. : * *:*: * :. .*****:*

gal SFKENRKDDIWLVDFYAPWCGHCKKLEPVWNEVGMEMKNMGSPVKVGKMDATSFSSIASE

mus SFKDNRKDDIWLVDFYAPWCGHCKKLEPIWNEVGLEMKSIGSPVKVGKMDATSYSSIASE

hum SFKENRNDDIWLVDFYAPWCGHCKKLEPIWNEVGLEMKSIGSPVKVGKMDATSYSSIASE

pan SFKENRNDDIWLVDFYAPWCGHCKKLEPIWNEVGLEMKSIGSPVKVGKMDATSYSSIASE

***:**:*********************:*****:***.:*************:******

gal FGVRGYPTIKLLKGDLAYNYRGPRTKDDIIEFANRVAGPLIRPLPSQHMFEHVRKRHRVL

mus FGVRGYPTIKLLKGDLAYNYRGPRTKDDIIEFAHRVSGALIRPLPSQQMFDHVRKRHRVF

hum FGVRGYPTIKLLKGDLAYNYRGPRTKDDIIEFAHRVSGALIRPLPSQQMFEHMQKRHRVF

pan FGVRGYPTIKLLKGDLAYNYRGPRTKDDIIEFAHRVSGALIRPLPSQQMFEHMQKRHRVF

*********************************:**:* ********:**:*::*****:

gal FVYVGGESPLKEKYIEVASELIVYTYFFSASEDVLPEYLTLPELPAVVVFKDGTYFVYDE

mus FVYIGGESPLKEKYIDAASELIVYTYFFSASEDVVPEYVTLKEMPAVLVFKDDTYFVYDE

hum FVYVGGESPLKEKYIDAASELIVYTYFFSASEEVVPEYVTLKEMPAVLVFKDETYFVYDE

pan FVYVGGESPLKEKYIDAASELIVYTYFFSASEEVVPEYVTLKEMPAVLVFKDETYFVYDE

***:***********:.***************:*:***:** *:***:**** *******

gal YEDGDLSSWINRERFQGYLTVDGFTLYELGDTGKLVAIAVIDDKNSSVEHTRLKSIIQEV

mus YEDGDLSSWISRERFQNYLTMDGFLLYELGDTGKLVAIAVIDEKNTSLEHTRLKSIIQEV

hum YEDGDLSSWINRERFQNYLAMDGFLLYELGDTGKLVALAVIDEK**N**TSVEHTRLKSIIQEV

pan YEDGDLSSWINRERFQNYLAMDGFLLYELGDTGKLVALAVIDEKNTSVEHTRLKSIIQEV

**********.*****.**::*** ************:****:**:*:************

gal ARDYRDHFHRDFQFGHMDGNDYINSLLMDDLTIPTIVVLNTSNQQYFLPDRHIENTEDMV

mus ARDFRDHFHRDFQFGHMDGNDYINTLLMDELTVPTIVVLNTSNQQYFLLDRHIKDASDMV

hum ARDYRDLFHRDFQFGHMDGNDYINTLLMDELTVPTVVVL**N**TSNQQYFLLDRQIKNVEDMV

pan ARDYRDLFHRDFQFGHMDGNDYINTLLMDELTVPTVVVLNTSNQQYFLLDRQIKNVEDMV

***:** *****************:****:**:**:************ **:*::..***

gal QFINNILDGTAEAQGGDGVLQRIKRIVYDAKSTVVSVFKSSPLLGCFLFGLPLGVISIMC

mus QFINSILDGTVPAQGGDSIFQRLKRIVFDAKSTIVSIFKSSPLMGCFLFGLPLGVISIMC

hum QFINNILDGTVEAQGGDSILQRLKRIVFDAKSTIVSIFKSSPLMGCFLFGLPLGVISIMC

pan QFINNILDGTVEAQGGDSILQRLKRIVFDAKSTIVSIFKSSPLMGCFLFGLPLGVISIMC

****.*****. *****.::**:****:*****:**:******:****************

gal YGICTADTDGGVDEHEAVK--KENSDRELTDDGSEEEQEEENGKYTELSDGELKQKDLLE

mus YGIYTADTDGGYIEERYEVSKSEMENQEQIEES-KEQ-ESSSG--GSLAPTVQEPKDVLE

hum YGIYTADTDGGYIEERYEVSKSENENQEQIEES-KEQQEPSSG--GSVVPTVQEPKDVLE

pan YGIYTADTDGGYIEERYEVSKSENENQEQVEES-KEQQEPSSG--GSVVPTAQEPKDVLE

*** ******* *.. .* .::* ::. :*: * ..* .: : **:**

gal KKKD

mus KKKD

hum KKKD

pan KKKD

****

1. Equilibrative nucleoside transporter 1

gal --------------------------MTTRDGPQDRYKAVWLIFFILGLGTLLPWNFFMT

mus --------------------------MTTSHQPQDRYKAVWLIFFVLGLGTLLPWNFFMT

hum --------------------------MTTSHQPQDRYKAVWLIFFMLGLGTLLPWNFFMT

pan MLTPKSQQQAPEGGSCQPGKTENTITMTTSHQPQDRYKAVWLIFFMLGLGTLLPWNFFMT

*** . *************:**************

gal ARQYFINRLADPQNISHLSNQTSVGT---------ASDLSYLQSMFDNFMTLCSMVPLLI

mus ATKYFTNRLDVSQNVSSDTDQSCESTKALADPTVALPARSSLSAIFNNVMTLCAMLPLLV

hum ATQYFTNRLDMSQ**N**VSLVTAELSKDAQASAAPAAPLPERNSLSAIFNNVMTLCAMLPLLL

pan ATQYFTNRLDMSQNVSLVTAELSKDAQASAAPAAPLPERNSLSAIFNNVMTLCAMLPLLL

* :** *** **:* : : . .: . *.::*:*.****:*:***:

gal FTCLNSFIHQRIPQQIRISGSLVAIGLVFLITAIMVKVTMDPLPFFVFTMVSIVFINSFG

mus FTCLNSFLHQRISQSVRILGSLLAILLVFLVTAALVKVEMDALTFFVITMIKIVLINSFG

hum FTYLNSFLHQRIPQSVRILGSLVAILLVFLITAILVKVQLDALPFFVITMIKIVLINSFG

pan FTYLNSFLHQRIPQSVRILGSLVAILLVFLITAILVKVQLDALPFFVITMIKIVLINSFG

** ****:**** *.:** ***:** ****:** :*** :* * ***:**:.**:*****

gal AMLQGSLFGLAGLLPASYTAPIMSGQGLAGIFAALAMIISISIGAQQPESYIGYFTTACV

mus AILQASLFGLAGVLPANYTAPIMSGQGLAGFFTSVAMICAIASGSELSESAFGYFITACA

hum AILQGSLFGLAGLLPASYTAPIMSGQGLAGFFASVAMICAIASGSELSESAFGYFITACA

pan AILQGSLFGLAGLLPASYTAPIMSGQGLAGFFASVAMICAIASGSELSESAFGYFITACA

*:**.*******:***.*************:*:::*** :*: *:: ** :*** ***.

gal AILLAIFSYVLLPRMDFFRYYSMKDKTEYHVCNAELETKRDLIKKDE-PNGMEQNNSKII

mus VVILAILCYLALPRTEFYRHYLQLNLA----GPAEQETKLDLISKGEEPKGRREESGVPG

hum VIILTIICYLGLPRLEFYRYYQQLKLE----GPGEQETKLDLISKGEEPRAGKEESGVSV

pan VIILTIICYLGLPRLEFYRYYQQLKLE----GPGEQETKLDLISKGEEPRAGKEESGVSV

.::*:*:.*: *** :*:*:* . .* *** ***.*.* *.. .::..

gal PVHN-PDEKPSVISIFKKLWVMAVSVCLVFTVTIGVFPSITAKVSTTLGKESKWDLYFVS

mus PNSPPTNRNQSIKAILKSICVPALSVCFIFTVTIGLFPAVTAEVESSIAGTSPWKSYFIP

hum SNSQPTNESHSIKAILKNISVLAFSVCFIFTITIGMFPAVTVEVKSSIAGSSTWERYFIP

pan SNSQPTSESHSIKAILKNISVLAFSVCFIFTITIGMFPAVTVEVKSSIAGSSTWERYFIP

... *: :*:*.: * *.***::**:***:**::*.:*.:::. * *. **:

gal VSCFLIFNVFDWMGRSLTALFTWPGKDSCLLPVMVVLRVIFIPLFMLCNVQPRNH----L

mus VACFLNFNVFDWLGRSLTAVCMWPGQDSRWLPVLVASRIVFVPLLMLCNVKARHCGAQRH

hum VSCFLTFNIFDWLGRSLTAVFMWPGKDSRWLPSLVLARLVFVPLLLLCNIKPRRY----L

pan VSCFLTFNIFDWLGRSLTAVFMWPGKDSRWLPSLVLARLVFVPLLLLCNIKPRRY----L

*:*** **:***:******: ***:** ** :* *::*:**::***:: *.

gal PVIFSHDAWYIIFMIFFSISNGYLASLCMCFGPKKVLAHEAETAGAVMAFFLTLGLALGA

mus HFVFKHDAWFIAFMAAFAFSNGYLASLCMCFGPKKVKPAEAETAGNIMSFFLCLGLALGA

hum TVVFEHDAWFIFFMAAFAFSNGYLASLCMCFGPKKVKPAEAETAGAIMAFFLCLGLALGA

pan TVVFEHDAWFIFFMAAFAFSNGYLASLCMCFGPKKVKPAEAETAGAIMAFFLCLGLALGA

.:*.****:* ** *::***************** ****** :*:*** *******

gal AISFLFQILI

mus VLSFLLRALV

hum VFSFLFRAIV

pan VFSFLFRAIV

.:***:: ::

1. Tcell surface glycoprotein CD4

gal MLPGPLWLLFFAGDEGAALKMEMICVLFLSLVPAYSRGQGVYAPAQAQIIHAGQACVVKE

mus ------------------------------------------------------------

hum ------------------------------------------------------------

pan ------------------------------------------------------------

gal DNISERVYTIREGDTLVLQCLVTGHPRPQVRWTKTAGSASDKFQETSVLNETLRIEKIQR

mus ------------------------------------------------------------

hum ------------------------------------------------------------

pan ------------------------------------------------------------

gal LQGGRYYCKAENGVGVPAIKSIRVDVQYLDEPVLTVHQTISDVRGSFYQEKTVFLRCTVN

mus ------MCR-----AISLRRLLLLLLQLSQLLA--VTQGKTLVLG--KEGESAELPCESS

hum ------MNR-----GVPFRH-LLLVLQLALLPA--ATQGKKVVLG--KKGDTVELTCTAS

pan ------MNR-----GVPFRH-LLLVLQLALLPA--ATQGKKVVLG--KKGDTVELTCTAS

: .: : : : :* . . * . * * : .:. * * .

gal SNPPARFIWKRGAETLSHS--------------QDNGVDIYEPLYTQGETKVLKLKNLRP

mus QKKITVFTWKFSDQRKILGQHGKGVLIRGGSPSQFDRFDSKKGAWEKGSF-PLIINKLKM

hum QKKSIQFHWKNSNQIKILGNQGS--FLTKGPSKLNDRADSRRSLWDQGNF-PLIIKNLKI

pan QKKSIQFHWKNSNQTKILGNQGS--FLTKGPSKLNDRVDSRRSLWDQGNF-PLIIKNLKI

.: * ** . : . : * . : :*. * :::*:

gal QDYASYTCQVSVRNVCSIPDKSITFQLTNTTAPPALKLSVNETLVVNPGDNVTMQCSLTG

mus EDSQTYICELENRKEEV---ELWVFKVT---FSPGTSL--------LQGQSLTLTLDSNS

hum EDSDTYICEVEDQKEEV---QLLVFGLT---ANSDTHL--------LQGQSLTLTLESPP

pan EDSDTYICEVGDQKEEV---QLLVFGLT---ANSDTHL--------LQGQSLTLTLESPP

:* :* *:: :: : .* :* * *:.:*: .

gal GDPQPEVLWSHSPGPLPPNSLVQGGNLTIWRIRVEDSGYYNCTAINNVGNPAKKTVNLLV

mus KVSNPLTECKHKKG----KVVSGSKVLSMSNLRVQDSDFWNCTVTLDQKKNWFGM-TLSV

hum G-SSPSVQCRSPRG----KNIQGGKTLSVSQLELQDSGTWTCTVLQNQKKVEFKI-DIVV

pan G-SSPSVQCRSPRG----KNIQGGKTLSVSQLELQDSGTWTCTVLQNQKKVEFKI-DIVV

.* . * : : . *:: .:.::**. :.**. : : : *

gal RSMKNATFQITPDVIKESETIQLGQDLKLSCHVDAVPQEKVVYSWYKNGKPARFSDRLLI

mus -----LGFQSTAITAYKSEGESAEFSFPLNFAEE----NGWGELMWKAEKDSFFQPWISF

hum -----LAFQKASSIVYKKEGEQVEFSFPLAFTVEKL--TGSGELWWQAERASSSKSWITF

pan -----LAFQKASSIVYKKEGEQVEFSFPLAFTVEKL--TGSGELWWQAERASSSKSWITF

** : :.* . .: * : :: : : . : :

gal TRNDPEL----------------PPVTCSLEIIDLRFSDYGTYLCVATFQGAPIPDLSVE

mus SIKNKEVSVQKSTKDLKLQLKETLPLTLKIPQVSLQFAGSGNLTLTLD-----KGTLHQE

hum DLKNKEVSVKRVTQDPKLQMGKKLPLHLTLPQALPQYAGSG**N**LTLALE---AKTGKLHQE

pan DLKNKEVSVKRVTQDPKLQMGKKLPLHLTLPQALPQYAGSGNLTLALE---AKTGKLHQE

:: *: *: .: :::. *. . * *

gal VNISSETVPPTISVPKGQSTITVREGSRAELQCEVRGKPKPPIIWSRVDKE--TPMPSGT

mus VNLV----------------VMKVAQLNNTLTCEVMGPTSPKMRLTLKQENQEARVSEE-

hum VNLV----------------VMRATQLQK**N**LTCEVWGPTSPKLMLSLKLENKEAKVSKR-

pan VNLV----------------VMRATQLQKNLTCEVWGPTSPKLMLSLKLENKEAKVSKR-

**: : . * *** * .* : : :: : : .

gal MTVETYDGKLRLESVSRDMSGTYKCQTARYNGFNIRPREALVQLNV-QFPPVVEPAFQ--

mus ---------QKVVQVVAPETGLWQCLLSEGDKVKMDSRIQVLSRGVNQ-TVFLACVLGGS

hum ---------EKAVWVLNPEAGMWQCLLSDSGQVLLESNIKVLPTWSTPVQPMALIVLGGV

pan ---------EKAVWVLNPEAGMWQCLLSDSGQVLLESNIKVLPTWSTPVQPMALIVLGGV

: * :* ::* : . . : . :: . .:

gal ---DVRQGMGRSVTLRCTMLKGSPMKVATSVWRFNGTLLAQPPAEQQDYSELKVDSVSRE

mus FGFLGFLGLCILCCVRCRHQQRQAAR---------------------------MSQIKRL

hum AGLLLFIGLGIFFCVRCRHRRRQAER---------------------------MSQIKRL

pan AGLLLFIGLGIFFCVRCRHRRRQAQR---------------------------MSQIKRL

*: :** : . : :..:.*

gal TSGSYECSISNDVGVSACLFQVSAKAYSPEFYYDTPNPTLSQKQSKNYSYILQWTQKEPD

mus LSEKKTCQCPHRMQKSHNLI----------------------------------------

hum LSEKKTCQCPHRFQKTCSPI----------------------------------------

pan LSEKKTCQCPHRFQKTCSPI----------------------------------------

* . *. : . : :

gal AVDPILKYRLEVRQLAQRNTIQTFIPVQKMEKGLLLEHILPNLKVPQSYEVRLTPITSFG

mus ------------------------------------------------------------

hum ------------------------------------------------------------

pan ------------------------------------------------------------

gal AGDMAARIIRYMEPINYPSPTDNTCRFEDEKICGFVQDKMDNFDWTRQNALTQNPKRTVN

mus ------------------------------------------------------------

hum ------------------------------------------------------------

pan ------------------------------------------------------------

gal TGPPTDISGTPEGYYMFIEASRPRVTGDKARLISPLYNITAKYYCVSFYYHMYGKHIGSL

mus ------------------------------------------------------------

hum ------------------------------------------------------------

pan ------------------------------------------------------------

gal NLLVRVRNKRAIDTQVWSLSGNRGNMWQQAHVPINPPGPFQIIFEGVRGTSYEGDIAIDD

mus ------------------------------------------------------------

hum ------------------------------------------------------------

pan ------------------------------------------------------------

gal VTLKKGDCPRKPIGPNKAVALPGSGVSAQHGPCLCGPLTFFLYVLLR

mus -----------------------------------------------

hum -----------------------------------------------

pan -----------------------------------------------

1. Ectonucleotide pyrophosphatase

gal MSALPLLKGEMMFPSLVLLFAALPVAKGSPVPHTSGRDKVLLVSFDGFRWDYDQDVDTPN

mus ------MGHSAVL-LCVALAILPACVTGAPVQR---QHKLLLVSFDGFRWNYDQDVDTPN

hum ------MRGLAVL-LTVALATLLAPGAGAPVQSQGSQNKLLLVSFDGFRWNYDQDVDTPN

pan ------MRGPAVL-LTVALATLLAPGAGAPVQSQGSQNKLLLVSFDGFRWNYDQDVDTPN

: :: * * *:** :.*:**********:*********

gal LDAMAAEGVKARYMTPAFITLTSPCHFTLLTGKYMENHGVIHNMCFNTSTGVKLPYYSTQ

mus LDSMAQEGVKAQYMTPAFVTMTSPCHFTLVTGKYIENHGVVHNMFYNTTSTVRLPYHATL

hum LDAMARDGVKARYMTPAFVTMTSPCHFTLVTGKYIENHGVVHNMYY**N**TTSKVKLPYHATL

pan LDAMARDGVKARYMTPAFVTMTSPCHFTLVTGKYIENHGVVHNMYYNTTSKVKLPYHATL

**:** :****:******:*:********:****:*****:*** :**:: *:***::*

gal GIDSWWDNGSLPIWITAQRQGLKTGSIHFPGTKAKYQGEEVSMKLVEPPLFNYSNETIWR

mus GIQRWWDNGSIPIWITAQRQGLKTGSFFYPGGNVTYQGEAVTMSRKEGVLHNYKNETEWR

hum GIQRWWD**N**GSVPIWITAQRQGLRAGSFFYPGG**N**VTYQGVAVTRSRKEGIAHNYK**N**ETEWR

pan GIQKWWDNGSVPIWITAQRQGLRAGSFFYPGGNVTYQGVAVTRSRKEGIAHNYKNETEWR

**: ******:***********::**:.:** :..*** *: . * .**.*** **

gal QNIDTAMEWFTVNNLDFITLYFGEPDSTGHKYGPESTQRRNMVGQVDKTIGYLRQRIRES

mus GNVDTVMKWFLEEDVSLVTLYFGEPDSTGHKYGPESQERKDMVKQVDRTVGYLRDSIKRH

hum ANIDTVMAWFTEEDLDLVTLYFGEPDSTGHRYGPESPERREMVRQVDRTVGYLRESIARN

pan ANIDTVMAWFTEEDLDLVTLYFGEPDSTGHKYGPESLERREMVRQVDRTVGYLRESIARN

*:**.* ** :::.::************:***** :*::** ***:*:****: * .

gal GLESNLNLIITSDHGMETVIKSN--EIYLRNVENFTFSDIQFELLDYGPNGLLIPKEGKL

mus HLSDSLNLIITSDHGMTTVNKKASDLVEFHKFSNFTFQDIQFELLDYGPIGMLIPKEGML

hum HLTDRLNLIITSDHGMTTVDKRAGDLVEFHKFP**N**FTFRDIEFELLDYGPNGMLLPKEGRL

pan HLTDRLNLIITSDHGMTTVDKRAGDLVEFHKFPNFTFRDIEFELLDYGPNGMLLPKEGRL

* . *********** ** * : :::. **** **:******** *:*:**** *

gal EHVYSVLKNAHPKLHVYKKEEFPKRFHYANHPRITPLLMYGDPGYVIHGRYKVQFNTGEH

mus EKVYSVLKDAHPRLHVYKKEDFPKNFHYANNPRITPLLMYSDLGYVIHGRVNVQFNNGEH

hum EKVYDALKDAHPKLHVYKKEAFPEAFHYANNPRVTPLLMYSDLGYVIHGRINVQFNNGEH

pan EKVYDALKDAHPKLHVYKKEAFPEAFHYANNPRVTPLLMYSDLGYVIHGRINVQFNNGEH

*:**..**:***:******* **: *****:**:******.* ******* :****.***

gal GFDNEAMNMKTIFRAVGPAFKQGLLVEPFESVNVYALLCELLGIAPEPHDGSLEVTRPML

mus GFNNQDMDMKTIFRAVGPSFKAGLEVEPFESVHVYELMCQLLGIVPEPNDGNPGILRPML

hum GFDNKDMDMKTIFRAVGPSFRAGLEVEPFESVHVYELMCRLLGIVPEANDGHLATLLPML

pan GFDNKDMDMKTIFRAVGPSFRAGLEVEPFESVHVYELMCRLLGIVPEANDGHPATLLPML

**:*: *:**********:*: ** *******:** *:*.****.** :** ***

gal RSSAPLLAAIKLP-VTLGIALILSCGRGVY---------------

mus RSGSASLLSSQHHLVALLCRSQNTAASGFYQAGDSN---------

hum HTESALPPDGRPTLLPKGRSALPPSSRPLLVMGLLGTVILLSEVA

pan HTESALPPDGRPTVLPKGRSALPPSSRPLLMMGLLGTVILLSEVT

:: : : : .. .

1. Carbonic anhydrase 9

gal ------------------------------------------------------------

mus ------------------------------------------------------------

hum ------------------------------------------------------------

pan MAPITFCLCTHLPLTPPPSWLWYGGEGTGPDKPVRLWLHLCKRALCESACSPPGLLLPHP

gal ------------------------------------------------------------

mus ----------------------MASLGPSPWAPLSTPAPTA----QLLLFLLLQVSAQPQ

hum ----------------------MAPLCPSPWLPLLIPAPAPGLTVQLLLSLLLLVPVHPQ

pan ALVSNARTARTHRVLGHPTVSHMAPLCPSPWLPLLIPAPAPGLTVQLLLSLLLLVPVHPQ

gal -------------------------------MYVRQSRPAGSRGPS------RTPHLLEM

mus GLSGMQGEPSLGDSSSGE-DELGVDVLPSEEDAPEEADPPDGEDPP------------EV

hum RLPRMQEDSPLGGGSSGEDDPLGEEDLPSEEDSPREEDPPGEEDLPGEEDLPGEEDLPEV

pan RLPRMQEDSPLGGGSSGEDDPLGEEDLPSEEDSPREEDPPREEDP------PGEEDLPEV

.: * .. *:

gal NRTALRVCVLLLAGLGRAAGGQEHDHNDENAPHSAEKGPSDSHWSYEDPGQWAKHFPACS

mus NSEDRMEESLGLEDLST-PEAPEHS----QGSHGDEKGGGHSHWSYGGTLLWPQVSPACA

hum KPKSEEEGSLKLEDLPT-VEAPGDPQEPQNNAHRDKEGDDQSHWRYGGDPPWPRVSPACA

pan KPKSEEEGSLKLEDLPT-VEAPGDPQEPQNNAHRDKEGDDQSHWRYGGDPPWPQVSPACA

: * * .* . . : * ::* ..*** * . * : ***:

gal GTMQSPININTETTIFSPQLRPIQLSGYSLPASQMLALKNNGHTVVLKLPESLAITGGYA

mus GRFQSPVDIRLERTAFCRTLQPLELLGYELQPLPELSLSNNGHTVQLTLPPGLKMALGPG

hum GRFQSPVDIRPQLAAFCPALRPLELLGFQLPPLPELRLRNNGHSVQLTLPPGLEMALGPG

pan GRFQSPVDIRPQLAAFCPALRPLELLGFQLPPLPELRLRNNGHSVQLTLPPGLEMALGPG

* :***::*. : : *. *:*::* *:.* * * ****:* *.** .* :: * .

gal QQYRAVQLHLHWGSPSNPGSEHTVDHKRFAGELHVVHYNTKYENFEAAVTQPDGLAVLGV

mus QEYRALQLHLHWGTSDHPGSEHTVNGHRFPAEIHVVHLSTAFSELHEALGRPGGLAVLAA

hum REYRALQLHLHWGAAGRPGSEHTVEGHRFPAEIHVVHLSTAFARVDEALGRPGGLAVLAA

pan REYRALQLHLHWGAAGRPGSEHTVEGHRFPAEIHVVHLSTAFARVDEALGRPGGLAVLAA

::***:*******: ..*******: :** .*:**** .* : ... *: :*.*****..

gal FLEVGPRENPYYQQILEHLRSIQEEDGEVFVPGFNIAGLLPDNLHLYFHYNGSLTTPPCL

mus FLQESPEENSAYEQLLSHLEEISEEGSKIEIPGLDVSALLPSDLSRYYRYEGSLTTPPCS

hum FLEEGPEENSAYEQLLSRLEEIAEEGSETQVPGLDISALLPSDFSRYFQYEGSLTTPPCA

pan FLEEGPEENSAYEQLLSRLEEIAEEGSETQVPGLDISALLPSDFSRYFQYEGSLTTPPCA

**: .*.** *:*:*.:*..* **..: :**::::.***.:: *::*:********

gal ESVKWTVFNQTVMLSKEQMSVLVSSLQTDDNHLLMNNFRQDQSLHRRWVLASFEPSSSRD

mus QGVIWTVFNETVKLSAKQLHTLSVSLWGPRDSRLQLNFRATQPLNGRTIEASFPAAEDSS

hum QGVIWTVF**N**QTVMLSAKQLHTLSDTLWGPGDSRLQLNFRATQPLNGRVIEASFPAGVDSS

pan QGVIWTVFNQTVMLSAKQLHTLSDTLWGPGDSRLQLNFRATQPLNGRVIEASFPAGVDSS

:.* *****:** ** :*: .* :* : * *** * *: * : *** . . .

gal RQVPAGGGSSVTAAGHTSSFHAGDVLAVLFGVLFAITMLAFLLYVYKNRSQNARLDSPTK

mus P----------EPVHVNSCFTAGDILALVFGLLFAVTSIAFLLQLRRQHR----HRSGTK

hum PR-------AAEPVQLNSCLAAGDILALVFGLLFAVTSVAFLVQMRRQH------RRGTK

pan PR-------AAEPVQLNSCLAAGDILALVFGLLFAVTSVAFLVQMRRQH------RRGTK

. .*.: ***:**::**:***:* :***: : ::: **

gal SKVIYTAATAENTA-

mus DRVSYSPAEMTETGA

hum GGVSYRPAEVAETGA

pan GGVSYRPAEVAETGA

. * * * :*.

1. IL-7 receptor

gal MLRMTRMSTVLSIFILFLHTTFGESGCTSADGDGTFGDDEPDNFDIDCFSQLEFKDSYSS

mus ---MMALGRAFAIVFCLIQAVSGESG---NAQDGDLEDADADDHSFWCHSQLEVDGSQHL

hum ---MTILGTTFGMVFSLLQVVSGESG---YAQNGDLEDAELDDYSFSCYSQLEV**N**GSQHS

pan ---MTILGTTFGMVFSLLQVVSGESG---YAQNGDLEDAELDDYSFSCYSQLEVNGSQHS

* :. .:.:.: :::.. **** :* : * : *:..: *.****...*

gal LTCNFTELPPHNTNYTLAVCTKEDSSYLCFNMEKQEDVYFLQFTDI--LSNKDICVEYEI

mus LTCAFNDSDINTANLEFQICGAL-LRVKCLTLNKLQDIYFIKTSEFLLIGSSNICVKLGQ

hum LTCAFEDPDV**N**TTNLEFEICGAL-VEVKCLNFRKLQEIYFIETKKFLLIGKSNICVKVGE

pan LTCAFEDPDVNTTNLEFEICGAL-VEVKCLNFRKLQEIYFIETKKFLLIGKSNICVKVGE

*** * : :.:* : :* *:.:.* :::**:: ..: :...:***:

gal KRRACRSLIVTDIVKPEVPFDINITYQKEANEYLIHYSTPHSRKKYLKDKLIHQIAYRQE

mus KNLTCKNMAINTIVKAEAPSDLKVVYRKEANDFLVTFNAPHLKKKYLK-KVKHDVAYRPA

hum KSLTCKKIDLTTIVKPEAPFDLSVIYREGANDFVVTF**N**TSHLQKKYVK-VLMHDVAYRQE

pan KSLTCKKIDLTTIVKPEAPFDLSVIYREGANDFVVTFNTSHLQKKYVK-VLMHDVAYRQE

* :*:.: :. *** *.* *:.: *:: **:::: :.: * :***:* : *::***

gal --ESTWKTIKSPYLQVKLLGKNLEADALYEVKVRSQPNGDYFKGIWSEWSTSKSFRTTGE

mus RGESNWTHVSLFHTRTTIPQRKLRPKAMYEIKVRSIPHNDYFKGFWSEWSPSSTFETPEP

hum KDENKWTHV**N**LSSTKLTLLQRKLQPAAMYEIKVRSIPD-HYFKGFWSEWSPSYYFRTPEI

pan KDENKWMHVNLSSTKLTLLQRKLQPAAMYEIKVRSIPD-HYFKGFWSEWSPSYYFRTPEI

*..* :. : .: ::*. *:**:**** *. .****:***** * *.*

gal HSMESYSSMFVIILSIPGFILSVVMIVLILTFWESRIKPVVWPNLPDHKITLERLCKRPK

mus KNQ-GGWDPVLPSVTILSLFSVFLLVILAHVLWKKRIKPVVWPSLPDHKKTLEQLCKKPK

hum **NN**SSGEMDPILLTISILSFFSVALLVILACVLWKKRIKPIVWPSLPDHKKTLEHLCKKPR

pan NNSSGEMDPILLTISILSFFSVALLVILACVLWKKRIKPIVWPSLPDHKKTLEHLCKKPR

:. . . .: ::* .:: ::::* .:*:.****:***.***** ***:***:*:

gal NNFDISFNPESFGYVFIHEVDGIQAKAEQENFLQPPPTPETDIPPKFRSGSDLKRSPARI

mus TSLNVSFNPESFLDCQIHEVKGVEARDEVESFLPNDLPAQPEELETQGHRAAVHS-ANRS

hum KNLNVSFNPESFLDCQIHRVDDIQARDEVEGFLQDTFPQQLEESEKQRLGGDVQS-PNCP

pan KNLNVSFNPESFLDCQIHRVDDIQARDEVEGFLQDTFPQQLEESEKQRLGGDVQS-PSCP

..:::******* **.*..::*: * *.** : : . . ::

gal DKNSLNLSVSYGGIWPAEALHGLFGCSQSTAADVCSSGTYEVCH-----SSRVPLCDNGF

mus PETSVSPPETVRRESPL----------------RCLARNLSTCNAPPLLSSRSPDYRDGD

hum SEDVVITPESFGRDSSL----------------TCLAGNVSACDAPILSSSRSLDCRESG

pan SEDVVITPESFGRDSSL----------------TCLAGNVSACDAPILSSSRSLDCRESD

: : : * : . ..*. *** :.

gal HL-PS-----APPLDPPGQ----PGPQPQ-NGNVV-----PPNSELKSPSDEEAYVTMSS

mus RNRPPVYQDLLPNSGNTNVPVPVPQPLPFQSGILIPVSQRQPISTSSVLNQEEAYVTMSS

hum KNGPHVYQDLLLSLGTTNSTLPPPFSLQSGILTLNPVAQGQPILTSLGSNQEEAYVTMSS

pan KNGPHVYQDLLLSLGTTNSTLPPPFSLQSGILTLNPVAQGQPILTSLGSNQEEAYVTMSS

: * . . * : * .:*********

gal FYKNQ

mus FYQNK

hum FYQNQ

pan FYQNQ

**:*:

1. Metalloreductase STEAP4

gal MNKNSSNIMALVPKTSNKRETVCIFGTGDFGRALGHKLIQSGYPVVFGSRSPWPSSLIPK

mus MEKAHADEFPLTTDSSEKQGVVCIFGTGDFGKSLGLKMLQCGYSIVFGSRNPQVSSLLPR

hum MEKTCIDALPLTMNSSEKQETVCIFGTGDFGRSLGLKMLQCGYSVVFGSRNPQKTTLLPS

pan MEKTCIDALPLTMNSSEKQETVCIFGTGDFGRSLGLKMLQCGYSVVFGSRNPQKTTLLPS

*:* : : *. .:*:*: .**********::** *::*.** :*****.* ::*:*

gal DAEVLNHAEAAQKAAIIIIAIQRQHYDFLASLEETLHGKVLVDISNNLKINQYPESNAKY

mus GAEVLSYSEAASKSDIIILAMHREHYDSLTELVDYLKGKVLVDVSNNRKINQYPESNAEY

hum GAEVLSYSEAAKKSGIIIIAIHREHYDFLTELTEVLNGKILVDISNNLKINQYPESNAEY

pan GAEVLSYSEAAKKSDIIIIAIHREHYDFLTELTEVLNGKILVDISNNLKINQYPESNAEY

.****.::***.*: ***:*::*:*** *:.* : *:**:***:*** **********:*

gal LAQLVPGARVVKAFNTVSAWALQSGTLDASRQVFVCGDDTEAKQMVMDIIRALGLTPLDQ

mus LAQLEPGAHVVKAFNTISAWALQSGTLDASRQVFVCGNDSKAKQRVMDIARTLGLTPLDQ

hum LAHLVPGAHVVKAFNTISAWALQSGALDASRQVFVCGNDSKAKQRVMDIVRNLGLTPMDQ

pan LAHLVPGAHVVKAFNTISAWALQSGALDASRQVFVCGNDSKAKQRVMDIVRNLGLTPMDQ

**:* ***:*******:********:***********:*::*** **** * *****:**

gal GSLLAAREIENYPLQLFPMWKIPIFLSLGLTAFFFFYCLVRDVIYPYVYENKDYSFFLAI

mus GSLMAASEIENYPLQLFPMWRFPFYLSSVLCVFFFVYCAIREVIYPYVNGKTDATYRLAI

hum GSLMAAKEIEKYPLQLFPMWRFPFYLSAVLCVFLFFYCVIRDVIYPYVYEKKDNTFRMAI

pan GSLMAAKEIEKYPLQLFPMWRFPFYLSAVLCVFLFFYCVIRDVIYPYVYEKKDNTFRMAI

***:** ***:*********::*::** * .*:*.** :*:****** :.* :: :**

gal SIPNRICPIIALVLLALVYLPGILAAIIQLYRGTKYSRFPDWLDKWMLCRKQLGLVALAF

mus SIPNRVFPITALILLALVYLPGILAAILQLYRGTKYRRFPNWLDHWMLCRKQLGLVALGF

hum SIPNRIFPITALTLLALVYLPGVIAAILQLYRGTKYRRFPDWLDHWMLCRKQLGLVALGF

pan SIPNRIFPITALTLLALVYLPGVIAAILQLYRGTKYRRFPDWLDHWMLCRKQLGLVALGF

*****: ** ** *********::***:******** ***:***:*************.*

gal ASLHVIYTLVIPIRYYVRWRIEDRTISQALNNKTTPFDNTNGWLSDSYLALGILGFLLFV

mus AFLHVIYTLVIPIRYYVRWRLRNATITQALTNKDSPFITSYAWINDSYLALGILGFFLFL

hum AFLHVLYTLVIPIRYYVRWRLG**N**LTVTQAILKKENPFSTSSAWLSDSYVALGILGFFLFV

pan AFLHVLYTLVIPIRYYVRWRLGNLTATQAILKKENPFSTSSAWLSDSYVALGILGFFLFV

* ***:**************: : * :**: :* .** .: .*:.***:*******:**:

gal LLGITSLPSVSNNVNWREFRFVQSKLGYLTLILCTAHTLVYGGKRFLSPSAYRWYLPNAY

mus LLGITSLPSVSNMVNWREFRFVQSKLGYLTLVLCTAHTLVYGGKRFLSPSILRWSLPSAY

hum LLGITSLPSVSNAVNWREFRFVQSKLGYLTLILCTAHTLVYGGKRFLSPSNLRWYLPAAY

pan LLGITSLPSVSNAVNWREFRFVQSKLGYLTLILCTAHTLVYGGKRFLSPSNLRWYLPAAY

************ ******************:****************** ** ** **

gal MLSLIIPCIVLVVKFVLILPCLDKQLTRIRQGWERNPQYSEQSNYVINKSAV

mus ILALVIPCAVLVLKCILIMPCIDKTLTRIRQGWERNSKYTQSALNG--KSDI

hum VLGLIIPCTVLVIKFVLIMPCVDNTLTRIRQGWERNSKH-------------

pan VLGLIIPCTVLVIKFVLIMPCVDNTLTRIRQGWERNSKH-------------

:*.*:*** ***:* :**:**:*: *********** ::

1. Angiopoietin-related protein

gal MKIILLLLFVAPLALSVRAEKDFAFLDSAATPETKSRFAMLDDVRILANGLLQLGHGLKD

hum MFTIKLLLFIVPLVISSRIDQDNSSFDSL-SPEPKSRFAMLDDVKILANGLLQLGHGLKD

pan MFTIKLLLFIVPLVISSRIDQDNSSFDSL-SPEPKSRFAMLDDVKILANGLLQLGHGLKD

mus MHTIKLFLFVVPLVIASRVDPDLSSFDSA-PSEPKSRFAMLDDVKILANGLLQLGHGLKD

* * *:**:.**.:: * : * : :** * **********:***************

gal FVHKTKGQMNDIFQKLYIFDRSFYELSLQTSEIKEEEEQLRQTTARLQINNEEIKNLSQE

hum FVHKTKGQINDIFQKLNIFDQSFYDLSLQTSEIKEEEKELRRTTYKLQVKNEEVK**N**MSLE

pan FVHKTKGQINDIFQKLNIFDQSFYDLSLQTSEIKEEEKELRRTTYKLQVKNEEVKNMSLE

mus FVHKTKGQINDIFQKLNIFDQSFYDLSLRTNEIKEEEKELRRTTSTLQVKNEEVKNMSVE

********:******* ***:***:***:*.******::**:** **::***:**:* *

gal MNLKIEDLIQNKIQLQEKVWGLEDKVTKLAIIQPTVQETNEISSLKAFVEQQDNHIKQLH

hum LNSKLESLLEEKILLQQKVKYLEEQLTNLIQNQPETPEHPEVTSLKTFVEKQDNSIKDLL

pan LNSKLESLLEEKILLQQKVKYLEEQLTNLIQNQPETPEHPEVTSLKTFVEKQDNSIKDLL

mus LNSKLESLLEEKTALQHKVRALEEQLTNLILSPAGAQEHPEVTSLKSFVEQQDNSIRELL

:* *:*.*:::* **.** **:::*:* . * *::***:***:*** *::*

gal KVVEDQHVQLDKQHNQIMELEDKLNHIELQELAENSFLEEQAESNEGSPFLVHNSTAVMH

hum QTVEDQYKQLNQQHSQIKEIENQLRRTSIQEPTEISLSSKPRAP-RTTPFLQLNEIR-NV

pan QTVEDQYKQLNQQHSQIKEIENQLRRTSIQEPTEISLSSKPRAP-RTTPFLQLNEIR-NV

mus QSVEEQYKQLSQQHMQIKEIEKQLRKTGIQEPSENSLSSKSRAP-RTTPPLQLNETE-NT

: **:*: **.:** ** *:*.:*.: :** :* *: .: . :* * *.

gal KLEGATPDCTALYNSGIRSSGIYTIKPNGSEAFDVYCEMKFGTSWTVIQNRVDGSLDFNQ

hum KHDGIPAECTTIYNRGEHTSGMYAIRPSNSQVFHVYCDVISGSPWTLIQHRIDGSQNF**N**E

pan KRDGIPAECTTIYNRGEHTSGMYAIRPSNSQVFHVYCDVVSGSPWTLIQHRIDGSQNFNE

mus EQDDLPADCSAVYNRGEHTSGVYTIKPRNSQGFNVYCDTQSGSPWTLIQHRKDGSQDFNE

: :. :*:::** * ::**:*:*:* .*: *.***: *: **:**:* *** :**:

gal TWDAYTNGFGDLNEEFWLGLNKTFSITKQGDYILRIELQDWKDNKRYVEYAFTLGGPETD

hum TWENYKYGFGRLDGEFWLGLEKIYSIVKQSNYVLRIELEDWKDNKHYIEYSFYLGNHET**N**

pan TWENYKYGFGRLDGEFWLGLEKIYSIVKQSNYVLRIELEDWKDNKHYIEYSFYLGNHETN

mus TWENYEKGFGRLDGEFWLGLEKIYAIVQQSNYILRLELQDWKDSKHYVEYSFHLGSHETN

**: * *** *: ******:* ::*.:*.:*:**:**:****.*:*:**:* **. **:

gal YVLQLSRISGSIPNALPEQTELRFSTADRDMAIINDLDCPQNYLGGW-WHSECEETNLNG

hum YTLHLVAITGNVPNAIPENKDLVFSTWDHK--AKGHFNCPEGYSGGWWWHDECGENNLNG

pan YTLHLVAITGNVPNAIPENKDLVFSTWDHK--AKGHFNCPEGYSGGWWWHDECGENNLNG

mus YTLHVAEIAGNIPGALPEHTDLMFSTWNHR--AKGQLYCPESYSGGWWWNDICGENNLNG

*.*:: *:*.:*.*:**:.:* *** :: ..: **:.* *** *:. * *.****

gal KYVTPRSKGRLDRTKGLYWKPKNGRYYLLKSTKIMIHPTDLKIFD

hum KYNKPRAKSKPERRRGLSWKSQNGRLYSIKSTKMLIHPTDSESFE

pan KYNKPRAKSKPERRRGLSWKSQNGRLYSIKSTKMLIHPTDSESFE

mus KYNKPRTKSRPERRRGIYWRPQSRKLYAIKSSKMMLQPTT-----

** .**:*.: :* :*: *: :. : * :**:*::::**

1. cAMP-responsive element-binding protein 3-like protein

gal MCCWSLNFATRVRASQGRRWPGWASRTPKELRAKDGSAGSPLELGSHTAMASTVGGLDSI

hum -------------------------------------MNTDLAAGKMASAACSMDPIDSF

pan -------------------------------------MNMDLAAGKMASAACSMDPIDSF

mus -------------------------------------MDGDIAAGKMASPVCAMAPLDSM

. : *. :: ..:: :**:

gal DLLDLLFDRQDGILRGVELGTTSPGTWHE--DGRAQDGEDFLSSILGSGDSISDSPSWSP

hum ELLDLLFDRQDGILRHVELGEGWGHVKD-QQVLPNPDSDDFLSSILGSGDSLPSSPLWSP

pan ELLDLLFDRQDGILRHVELGEGWGHVKD-QQVLPNPDSDDFLSSILGSGDSLPSSPLWSP

mus EVLDLLFDRQDGILRNVELAEGWILAREEQKVLLNSDSDEFLNCILGPGDSDPSSPLWSP

::************* ***. . . *.::**..*** *** .** ***

gal GVSDSGVSEDPPSDQLDSPPGCCDG-----------GL---SEAPYAFTNSCQVLTHPGG

hum EGSDSGISEDLPSDPQDTPPRSGP--ATSPAGCHPAQPGKGPCLSYHPGNSCSTTTPGPV

pan EGSDSGISEDLPSDPQDTPPRSGP--ATSPAGCHPAQPGKGPCLSYHPGNSCPTTPPGPV

mus ADSDSGISEDLPSDPQDTPPRSGTEPANTVARCHTREQGKGPCPSYLPSTPCPEP---PR

****:*** *** *:** . * . *

gal TGAPHPEVSIDLDMWNPGFFLEEGRGLSTVPVPASCTLTVKDLLLSGSSDAPQM----PS

hum IQVPEASVTIDLEMWSPGGRICAEKPADPVDLSPRCNLTVKDLLLSGSSGDLQQHHLGAS

pan IQVPEASVTIDLEMWSPGGRICAEKPADPVDLSPRCNLTVKDLLLSGSSGDLQQHHLGAS

mus TQVQESSVAIDLDMWSTDTLY----PEEPAGSPSRFNLTVKELLLSGGSGDLQQHSLAAS

. . .*:***:**. . . . .****:*****.*. * *

gal SLLRQGQGQFQELVLTEDEKKLLAKEGVSLPTQLPLTKYEERVLKKIRRKIRNKQSAQES

hum YLLRPGAGHCQELVLTEDEKKLLAKEGITLPTQLPLTKYEERVLKKIRRKIRNKQSAQES

pan YLLRPGAGHCQELVLTEDEKKLLAKEGITLPTQLPLTKYEERVLKKIRRKIRNKQSAQES

mus QLLGPGSGHCQELVLTEDEKKLLAKEGVTLPTQLPLTKYEERVLKKIRRKIRNKQSAQES

** * *: *****************::*******************************

gal RKKKKEYIDGLESRMSACTAQNQELQRKVLHLEKQNSSLLEQLKKLQALVVQSSNKAAQT

hum RKKKKEYIDGLETRMSACTAQNQELQRKVLHLEKQNLSLLEQLKKLQAIVVQSTSKSAQT

pan RKKKKEYIDGLETRMSACTAQNQELQRKVLHLEKQNLSLLEQLKKLQAIVVQSTSKSAQT

mus RKKKKEYIDGLENRMSACTAQNQELQRKVLHLEKQNLSLLEQLKHLQALVVQSTSKPAHA

************.*********************** *******:***:****:.* *::

gal GTCIAVLLLSFALIVFPSISPFAASKAETGGDFGPVRVFSRSLHNTAASRVAYAQPRAGD

hum GTCVAVLLLSFALIILPSISPFGPNKTESPGDFAPVRVFSRTLHNDAASRVAADAVPGSE

pan GTCVAVLLLSFALIILPSISPFGPNKTESPGDFAPVRVFSRTLHNDAASRVAADAVPGSE

mus GTCIAVLLLSFALIILPSISPFNSNKVDSPGDFVPVRVFSRTLHNHAASRVAPDVTPGSE

***:**********::****** .*.:: *** *******:*** ****** ..:

gal EKPPEPLWSKHQ--DETETLHKAFGSSSFTPHLDKAPTRNSTPPLASEGLSQSDGDQSGT

hum APGPRPEADTTRE-ESPGSPGADWG-FQDTA**N**LT**N**-----STEELD**N**A----------TL

pan APGPRPEADTTQE-ESPGSPGADWG-FQDTANLTN-----SMEELDNA----------TL

mus VPGPWPDVGTPHKGPSSGGLSADWGNFLEIPMLDN-----LTEELDNS----------TL

* * .. : . :* * : * .

gal ALGDSTALHQSLTSLAWTEADHSR----PTVLEPAEEL--------------------

hum VLR**N**ATEGLGQVALLDWVAPGPSTGSGRAGLEAAGDEL--------------------

pan VLRNATEGLGQVPLLDWVAPGPSTGSGRAGLEAAGDEL--------------------

mus VLANSTEDLGRATLLDWVASEPLLSPGRVGLEIPGEMWLSWVPRWLRVRLVQDALGVL

.* ::* * *. : .:

1. Vit K dependent protein C

gal ---------------------MWKLITIGVLLA---ACSSPVCHASIFYSYKDANQVLKI

hum ---------------------MWQLTSLLLFVATWGISGTPAPLDSVFSSSERAHQVLRI

pan MAAGRRTCSISTTRPCASASRMWQLTSLLLFVATWGISGTPAPLDSVFSSSERAHQVLRI

mus ---------------------MWQFRVFLLLMSTWGISSIPAHPDPVFSSSEHAHQVLRV

**:: : :::: .. *. :* * : *:***::

gal RKRANSFLEELKPGSVERECNEEKCNFEEASEIFETKEATLEFWSKYVDGDQCAQKP---

hum RKRANSFLEELRHSSLERECIEEICDFEEAKEIFQNVDDTLAFWSKHVDGDQCLVLPLEH

pan RKRANSFLEELRHSSLERECIEEICDFEEAKEIFQNVDDTLAFWSKHVDGDQCLVLPLEH

mus R-RANSFLEEMRPGSLERECMEEICDFEEAQEIFQNVEDTLAFWIKYFDGDQCSAPPLDH

* ********:: .*:**** ** *:****.***:. : ** ** *:.***** *

gal ------CSNGACKDNIGSYSCICDKGWEGAQCNYEVKYNNCSVDNGGCQHFCKEDPAKQC

hum PCASLCCGHGTCIDGIGSFSCDCRSGWEGRFCQREVSFL**N**CSLDNGGCTHYCLEEV--GW

pan PCASLCCGHGTCIDGIGSFSCDCRSGWEGRFCQREVSFLNCSLDNGGCTHYCLEEV--GW

mus QCDSPCCGHGTCIDGIGSFSCSCDKGWEGKFCQQELRFQDCRVNNGGCLHYCLEES--NG

*.:*:* *.***:** * .**** *: *: : :* ::**** *:* *:

gal RYCSCASGYQLTNDHNMCTPVVEFPCGRVKMDYT------------EGKAEFNIRLIGGN

hum RRCSCAPGYKLGDDLLQCHPAVKFPCGRPWKRMEKKRSHLKRDTEDQ-EDQVDPRLIDGK

pan RRCSCAPGYKLGDDLLQCQPAVKFPCGRPWKRMEKKRSHLKRDTEDQ-EDQVDPRLIDGK

mus RRCACAPGYELADDHMRCKSTVNFPCGKLGRWIEKKRKILKRDTDLEDELEPDPRIVNGT

* *:** **:* :* * .*:****: : : : : *::.*.

gal SGGRGFSPWQVMLQNLKGKFLCGGVLIHPSWVLTAAHCVETGETLKVRLGKYHRLRIENS

hum MTRRGDSPWQVVLLDSKKKLACGAVLIHPSWVLTAAHCMDESKKLLVRLGEYDLRRWEKW

pan MTRRGDSPWQVVLLDSKKKLACGAVLIHPSWVLTAAHCMDESKKLLVRLGEYDLRRWEKW

mus LTKQGDSPWQAILLDSKKKLACGGVLIHTSWVLTAAHCVEGTKKLTVRLGEYDLRRRDHW

:* ****.:* : * *: **.**** *********:: :.* ****:*. * ::

gal EQTIRVDKYVRHENYTKLTSDNDIAMLHLAEPVMYNKYALPICLPTRDLAEHELTTKGRQ

hum ELDLDIKEVFVHP**N**YSKSTTDNDIALLHLAQPATLSQTIVPICLPDSGLAERELNQAGQE

pan ELDLDIKEVFVHPNYSKSTTDNDIALLHLAQPATLSQTIVPICLPDSGLAERELNQAGQE

mus ELDLDIKEILVHPNYTRSSSDNDIALLRLAQPATLSKTIVPICLPNNGLAQQELTQAGQE

* : :.: . * **:: ::*****:*:**:*. .: :***** .**::**. *::

gal MLVTGWGSTSDE----MRNYSALLSYIEIPIVPKNECAQVMTNTISDNMLCAGSLGDRKD

hum TLVTGWGYHSSREKEAKR**N**RTFVLNFIKIPVVPH**N**ECSEVMSNMVSENMLCAGILGDRQD

pan TLVTGWGYHSSREKEAKRNRTFVLNFIKIPVVPHNECSEVMSNMVSENMLCAGILGDRQD

mus TVVTGWGYQSDRIKDGRRNRTFILTFIRIPLVARNECVEVMKNVVSENMLCAGIIGNTRD

:***** *.. ** : :*.:*.**:* :*** :**.* :*:****** :*: :*

gal SCSGDSGGPMATKYKDTWFLVGLVSWGEGCGKKEKFGVYTKVSQYLEWIQHHINKKSGSW

hum ACEGDSGGPMVASFHGTWFLVGLVSWGEGCGLLHNYGVYTKVSRYLDWIHGHIRDKEAPQ

pan ACEGDSGGPMVASFHGTWFLVGLVSWGEGCGLLHNYGVYTKVSRYLDWIHGHIRDKEAPQ

mus ACDGDSGGPMVVFFRGTWFLVGLVSWGEGCGHTNNYGIYTKVGSYLKWIHSYIGEKGVSL

:*.*******.. ::.*************** .::*:****. **.**: :* .*

gal RG---

hum KSWAP

pan KSWAP

mus KSQKL

:.

1. Nucleotide exchange factor SIL1

gal MSHWAGHLCTVAKVSLL-LLLVSAVFFQSSLSERVPEFALTKIEESDIKDGSGKEPIAGD

mus MAPQHLPSTRMASPGMLLGLLLTSCLTLCLSCQNSNNFALTNPEKSIHQESDTKETREEE

hum MAPQSLPSSRMAPLGMLLGLLMAACFTFCLSHQNLKEFALTNPEKSSTKETERKETKAEE

pan ----------MAPLGMLLGLLMAACFTFRLSHQNLKEFALTNPEKSSTKETERKETKAEE

:* .:* **::: : :. :****: *:* :: . ** :

gal DADPEDLEVFYPTHQWQAVRPGQAVPAGSHVRLNLQTGEREARLPDSESGKSDTKEEKRR

mus ELDTEILEVFHPTQEWQTLQPGQAVPAGSHVRMNLQTGVNEVKLQQEDKFQNNLKGF---

hum ELDAEVLEVFHPTHEWQALQPGQAVPAGSHVRLNLQTGEREAKLQYEDKFRNNLK-----

pan ELDAEVLEVFHPTHEWQALRPGQAVPAGSHVRLNLQTGEREAKLQYEDKFRNNLK-----

: * * ****:**::**:::************:***** .*.:* .:. :.: *

gal KRLNKMDIDTNSFTSQELKKALAKMKESEKAE----RMAHEEEVRKKFRPIEQLKEEFEK

mus KRGRRLDINANTYTSQDLKSALAKFKEGTEMENSKDELARQATVKQLFRPIEELKKEFDE

hum --GKRLDINTNTYTSQDLKSALAKFKEGAEMESSKEDKARQAEVKRLFRPIEELKKDFDE

pan --GKRLDINTNTYTSQDLKSALAKFKEGAEMESSKEDKARQAEVKRLFRPIEELKKDFDE

.::**::*::***:**.****:**. : * *:: *:: *****:**::*::

gal LNVKMETDYEIMDKLISKFNSSASTLDEKVAALYDLEYYVHQVDNAKDFLSMGGLRLVIE

mus LNVVLETDMQIMVRLINKFNSSSSSLEEKVAALFDLEYYVHQMDNAQDLLSFGGLQVVIN

hum LNVVIETDMQIMVRLINKF**N**SSSSSLEEKIAALFDLEYYVHQMDNAQDLLSFGGLQVVIN

pan LNVVIETDMQIMVRLINKFNSSSSSLEEKIAALFDLEYYVHQMDNAQDLLSFGGLQVEIN

*** :*** :** :**.*****:*:*:**:***:********:***:*:**:***:: *:

gal GLNSTEAVLKEHAAFVLGAALSSNPKVQIEAIEGGALQKLLVIVATEQPQAVKKKALFAL

mus GLNSTEPLVKEYAAFVLGAAFSSNPKVQVEAIEGGALQKLLVILATNQPLPAKKKVLFAL

hum GL**N**STEPLVKEYAAFVLGAAFSSNPKVQVEAIEGGALQKLLVILATEQPLTAKKKVLFAL

pan GLNSTEPLVREYAAFVLGAAFSSNPKVQVEAIEGGALQKLLVILATEQPLTAKKKVLFAL

****** :::*:********:*******:**************:**:** .***.****

gal SSLLRHFPYAQQQFLKLGGLQVLRGLFRQPGTSALCVRAVTLLYDLFVEKMLLEDSQ--H

mus CSLLRHFPYAQQQFLKLGGLQVLRSLVQEKSAKVLAVRVVTLLYDLVTEKMFAEEEAELT

hum CSLLRHFPYAQRQFLKLGGLQVLRTLVQEKGTEVLAVRVVTLLYDLVTEKMFAEEEAELT

pan CSLLRHFPYAQRQFLKLGGLQVLRSLVQEKGTEVLAVRVVTLLYDLVTEKMFAEEEAELT

.**********:************ *.:: .:..*.**.*******..***: *:.

gal GDHAEEKVQQYRRVQLVPAVLEQDWCVAVPGLLALPEHDAREKVLKAVAVLMEFCRERFR

mus QDSSPEKLQQYRQVQLLPGLQEQGWCEITAQLLALPEHDAREKVLQTLGALLTTCRDRYR

hum QEMSPEKLQQYRQVHLLPGLWEQGWCEITAHLLALPEHDAREKVLQTLGVLLTTCRDRYR

pan QEMSPEKLQQYRQVHLLPGLWEQGWCEITAHLLALPEHDAREKVLQTLGVLLTTCRDRYR

: : **:****:*:*:*.: **.** . **************:::..*: **:*:*

gal GDAALGATLGLLRSEYEELAAAERRDGDGDGYFQELLGSVNSILRELG

mus QDLQLSRTLGRLQAEYQALASLELQEGEDDGYFRELLASINSLMKELR

hum QDPQLGRTLASLQAEYQVLASLELQDGEDEGYFQELLGSVNSLLKELR

pan QDPQLGKTLASLQAEYQALASLELQDGEDEGYFQELLGSVNSLLKELR

* *. **. *::**: **: * ::*:.:***:***.*:**:::**

1. Hemopexin

gal MGGSTAALCLAALLVLVGGRPLTQHKPHTPGDEHPH-GAEPPGNDTALAQICGDEGGFDA

hum MARVLGAPVALGLWSLCWS--LAIATPLPPTSAHGNVAEGETKPDPDVTERCSDGWSFDA

pan MARVLGAPVALGLWSLCWS--LAIANPLPPTSAHGNVAEGETKPDPDVTERCSDGWSFDA

mus MARTAVALNILVLLGLCWS--LAVASPLPTAHGRVAEVENGTKPDSDVPEHCLDTWSFDA

*. * * * . *: .* : * : : * * .***

gal ATLSENGTMLFFRGGDVWEISGEGPQPHSRPLAESWPELEGPVDAALRIHRQDHPEEHQS

hum TTLDD**N**GTMLFFKGEFVWKSHKW----DRELISERWKNFPSPVDAAFRQ-------GH**N**S

pan TTLDDNGTMLFFKGEFVWKSHKW----DRELISERWKNFPSPVDAAFRQ-------GHNS

mus ATMDHNGTMLFFKGEFVWRGHSG----TRELISARWKNPITSVDAAFR--------GPDS

:*:..*******:* **. . :: * : ****:* :*

gal LYLFQDEKVWSYAGGQLRPGFPRLIGDEFPGVPGGLDAAVECHPEECGGETVLFFKGDKV

hum VFLIKGDKVWVYPPEKKEKGYPKLLQDEFPGIPSPLDAAVECHRGECQAEGVLFFQGDRE

pan VFLIKGDKVWVYPPEKKEKGYPKLLQDEFPGIPSPLDAAVECHRGECQAEGVLFFQGDRE

mus VFLIKEDKVWVYPPEKKENGYPKLFQEEFPGIPYPPDAAVECHRGECQSEGVLFFQGNRK

::*:: :*** * : . *:*:*: :****:* ******* ** .* ****:*::

gal FSFDLELRVTKERPWLDAGPCDAALRWLERYYCLQGTQFYRFRPHSWEVLPGYPRDLRDY

hum WFWDLATGTMKERSWPAVGNCSSALRWLGRYYCFQGNQFLRFDPVRGEVPPRYPRDVRDY

pan WFWDLATGTMKERSWPAVGNCSSALRWLGRYYCFQGNQFLRFDPVRGEVPPRYPRDVRDY

mus WFWDFATRTQKERSWSTVGNCTAALRWLERYYCFQGNKFLRFNPVTGEVPPRYPLDARDY

: :*: . *** * .* * :***** ****:**.:* ** * ** * ** * ***

gal FIPCPGRGHRHGNTSW--------GNAGDRCSGEP-FQAITSDDSGRIYAFRGGLSFRLD

hum FMPCPGRGHGH-R**N**GTGHG**N**STHHGPEYMRCSPHLVLSALTSDNHGATYAFSGTHYWRLD

pan FMPCPGRGHGH-RNGTGHGNSTHHGPEYMRCSPHLVLSALTSDNHGATYAFSGTHYWRLD

mus FVSCPGRGHGRPRNGTAHGNSTH--PMHSRCSPDPGLTALLSDHRGATYAFTGSHYWRLD

*: ****** : ... *** . : *: **. * *** * :***

gal SWRDGWHAWPQAHSWPGLQGDVDAAFSWDKRMYLIQGSQVSIYVSGRGGHQLVEGYPRAL

hum TSRDGWHSWPIAHQWPQGPSAVDAAFSWEEKLYLVQGTQVYVFLT-KGGYTLVSGYPKRL

pan TSRDGWHSWPIAHQWPQGPSAVDAAFSWEEKLYLVQGTQVYVFLT-KGGYTLVSGYPKRL

mus SSRDGWHSWPIAHHWPQGPSTVDAAFSWDDKVYLIQGTQVYVFLT-KGGNNLVSGYPKRL

: *****:** ** ** . *******:.::**:**:** :::: :** **.***: *

gal QEELGVPK------ADAAFTCPGSAELYVITGDRMQRVDLTKSPRHADEPQPLPYDGVDG

hum EKEVGTPHGIILDSVDAAFICPGSSRLHIMAGRRLWWLDLKSGAQATWTELPWPHEKVDG

pan EKEVGTPHGIILDSVDAAFICPGSSRLHIMAGRRLWWLDLKSGAQATWTELPWPHEKVDG

mus EKELGSPPGISLETIDAAFSCPGSSRLYVSSGRRLWWLDLKSGAQATWTEVSWPHEKVDG

::*:* * **** ****:.*:: :* *: :**... : : *:: ***

gal AMCTAD------------GIYLLRGDRYHRHRDVAELLAAHPPADPPSIAVDLFHCAQ

hum ALCMEKSLGPNSCSANGPGLYLIHGPNLYCYSDVEKLNAAKALPQP-Q**N**VTSLLGCTH

pan ALCMEKSLGPNSCSANGPGLYLIHGPNLYCYSDVEKLNAAKALPQP-QNVTSLLGCTH

mus ALCLDKSLGPNTCSSNGSSLYFIHGPNLYCYSSIDKLNAAKSLPQP-QKVNSILGCSQ

*:* . .:*:::* . : : .: :* **: :* . . .:: *::

1. Dipeptidyl peptidase 1

gal --MAGCALWAVVAVLAMLPAGLRADTPANCSYADLLGSWELRVWRAGG-RHGNCSQAAPV

mus MGPWTHSLRAVLLLVLLGVCTVRSDTPANCTYPDLLGTWVFQVGPRSSRSDINCSVMEAT

hum MGAGPSLLLAALLLLLSGDGAVRCDTPA**N**CTYLDLLGTWVFQVGSSGSQRDV**N**CSVMGPQ

pan MGAGPALLLAALLLLLSGDRAVRCDTPANCTYLDLLGTWVFQVGSSGSQRDVNCSVMGPQ

* *.: :: :*.******:* ****:* ::* .. . ***

gal EKTVLVNLQKLDVAQDSLGNFGFFTLIYNQGFEIVLNNYKWFAFFKYKKEGLNVTSYCNE

mus EEKVVVHLKKLDTAYDELGNSGHFTLIYNQGFEIVLNDYKWFAFFKYEVRGHTAISYCHE

hum EKKVVVYLQKLDTAYDDLGNSGHFTIIYNQGFEIVLNDYKWFAFFKYKEEGSKVTTYC**N**E

pan EKKVVVYLQKLDTAYDDLGNSGHFTIIYNQGFEIVLNDYKWFAFFKYKEEGSKVTTYCNE

*:.*:* *:***.* *.*** *.**:***********:*********: .* .. :**:*

gal TLPGWVHDVLGRNWACFTGQKISSSSSDVHVRQLPLQKPRVGLSSRRFVHNFDFVNAINA

mus TMTGWVHDVLGRNWACFVGKKVESHIEKVNMNAAHLGGLQERYSERLYTHNHNFVKAINT

hum TMTGWVHDVLGRNWACFTGKKVGTASENVYVNIAHLKNSQEKYSNRLYKYDHNFVKAINA

pan TMTGWVHDVLGRNWACFTGKKVGTASENVYVNTAHLKNSQEKYSNRLYKYDHNFVKAINA

*: **************.*:*: : ..* :. * : *.* : ::.:**:***:

gal HQKSWRATRYEEYENFSLEELTRRAGGLYSRTSRPKPAPLTPELLKKVSGLPESWDWRNV

mus VQKSWTATAYKEYEKMSLRDLIRRS-GHSQRIPRPKPAPMTDEIQQQILNLPESWDWRNV

hum IQKSWTATTYMEYETLTLGDMIRRSGGHSRKIPRPKPAPLTAEIQQKILHLPTSWDWRNV

pan IQKSWTATTYMEYETLTLGDMIRRSGGHSRKIPRPKPAPLTAEIQQKLLHLPTSWDWRNV

**** ** * ***.::* :: **: * : ******:* *: ::: ** *******

gal NGVNYVSPVRNQASCGSCYAFASMGMLEARIRILTNNTQKPVFSPQQVVSCSQYSQGCDG

mus QGVNYVSPVRNQESCGSCYSFASMGMLEARIRILTNNSQTPILSPQEVVSCSPYAQGCDG

hum HGINFVSPVRNQASCGSCYSFASMGMLEARIRILT**N**NSQTPILSPQEVVSCSQYAQGCEG

pan HGINFVSPVRNQASCGSCYSFASMGMLEARIRILTNNSQTPILSPQEVVSCSQYAQGCEG

:*:*:******* ******:*****************:*.*::***:***** *:***:*

gal GFPYLIAGKYVQDFGVVEEDCFPYTAKDTPCLFKRSCYHYYTSEYHYVGGFYGACNEALM

mus GFPYLIAGKYAQDFGVVEESCFPYTAKDSPCKPRENCLRYYSSDYYYVGGFYGGCNEALM

hum GFPYLIAGKYAQDFGLVEEACFPYTGTDSPCKMKEDCFRYYSSEYHYVGGFYGGCNEALM

pan GFPYLIAGKYAQDFGLVEEACFPYTGTDSPCKMKEDCFRYYSSEYHYVGGFYGGCNEALM

**********.****:*** *****..*:** :..* :**:*:*:*******.******

gal KLELVLSGPMAVAFEVYNDFMFYKEGIYHHTGLKDEFNPFELTNHAVLLVGYGKDPESGE

mus KLELVKHGPMAVAFEVHDDFLHYHSGIYHHTGLSDPFNPFELTNHAVLLVGYGRDPVTGI

hum KLELVHHGPMAVAFEVYDDFLHYKKGIYHHTGLRDPFNPFELTNHAVLLVGYGTDSASGM

pan KLELVHHGPMAVAFEVYDDFLHYKKGIYHHTGLRDPFNPFELTNHAVLLVGYGTDSASGM

***** *********::**:.*:.******** * ***************** * :*

gal KFWIVKNSWGTSWGEDGYFRIRRGTDECAIESIAVAATPIPKL

mus EYWIIKNSWGSNWGESGYFRIRRGTDECAIESIAVAAIPIPKL

hum DYWIVKNSWGTGWGENGYFRIRRGTDECAIESIAVAATPIPKL

pan DYWIVKNSWGTGWGEDGYFRIRRGTDECAIESIAVAATPIPKL

.:**:*****:.***.********************* *****

1. Glucagon-like peptide receptor

gal MPVAPAV--PLVLLLAAAGRAAPARRSTDGSLSGVVQKWKEYQLQCLKYLYEAPPIAAEG

mus MASTPSLLRLALLLLGAVGRAGPRPQGTTVSLSETVQKWREYRRQCQRFLTEAP-LLATG

hum MAGAPGPLRLALLLLGMVGRAGPRPQGATVSLWETVQKWREYRRQCQRSLTEDP-PPATD

pan MAGAPGPLRLALLLLGMVGRAGPRPQGATVSLWETVQKWREYRRQCQRSLTEDP-PPATD

* :*. :***. .***.* :.: ** .****:**: ** : * * * * .

gal KFCNRTFDNYACWPDGLPGTYVNVSCPWYLPWANTVLHGQVYRFCTSEGTWLLKENSTLP

mus LFCNRTFDDYACWPDGPPGSFVNVSCPWYLPWASSVLQGHVYRFCTAEGLWLHKDNSSLP

hum LFC**N**RTFDEYACWPDGEPGSFV**N**VSCPWYLPWASSVPQGHVYRFCTAEGLWLQKD**N**SSLP

pan LFCNRTFDEYACWPDGEPGSFVNVSCPWYLPWASSVPQGHVYRFCTAEGLWLQKDNSSLP

*******:******* **::************.:* :*:******:** ** *:**:**

gal WRNLTECEASD---QDAPEEQLLNLSIIYTIGYALSFSALVIATAILLGFRHLHCTRNYI

mus WRDLSECEESKRGERNFPEEQLLSLYIIYTVGYALSFSALVIASAILVGFRHLHCTRNYI

hum WRDLSECEESKRGERSSPEEQLLFLYIIYTVGYALSFSALVIASAILLGFRHLHCTRNYI

pan WRDLSECEESKRGERSSPEEQLLFLYIIYTVGYALSFSALVIASAILLGFRHLHCTRNYI

**:*:*** *. :. ****** * ****:************:***:************

gal HLNLFTSFILRAISVFIKDSVVKWMYSTATQEHQWEGLISFQESLSCRLVFVMMQYCVAA

mus HLNLFASFILRALSVFIKDAALKWMYSTAAQQHQWDGLLSYQDSLGCRLVFLLMQYCVAA

hum HLNLFASFILRALSVFIKDAALKWMYSTAAQQHQWDGLLSYQDSLSCRLVFLLMQYCVAA

pan HLNLFASFILRALSVFIKDAALKWMYSTAAQQHQWDGLLSYQDSLSCRLVFLLMQYCVAA

*****:******:******:.:*******:*:***:**:*:*:**.*****::*******

gal NYYWLLVEGMYLYTLLVLSVFSEQRIFRLYLCIGWGVPMLFVILWGTVKYLYEDEGCWSR

mus NYYWLLVEGVYLYTLLAFSVFSEQRIFKLYLSIGWGVPLLFVIPWGIVKYLYEDEGCWTR

hum NYYWLLVEGVYLYTLLAFSVLSEQWIFRLYVSIGWGVPLLFVVPWGIVKYLYEDEGCWTR

pan NYYWLLVEGVYLYTLLAFSVFSEQRIFRLYVSIGWGVPLLFVVPWGIVKYLYEDEGCWTR

*********:******.:**:*** **:**:.******:***: ** ***********:*

gal NYNMNYWLIIRLPILIAIGVNFLIFIRVICIIISKLQANLMCKTDIKCRLAKSTLTLIPL

mus NSNMNYWLIIRLPILFAIGVNFLIFIRVICIVVSKLKANLMCKTDIKCRLAKSTLTLIPL

hum NSNMNYWLIIRLPILFAIGVNFLIFVRVICIVVSKLKANLMCKTDIKCRLAKSTLTLIPL

pan NSNMNYWLIIRLPILFAIGVNFLIFVRVICIVVSKLKANLMCKTDIKCRLAKSTLTLIPL

* *************:*********:*****::***:***********************

gal LGTHEVIFAFITDEHARGMLRFVKLFTELSFASFQGLMVAILYCFINNEVQMEFRKSWER

mus LGTHEVIFAFVMDEHARGTLRFIKLFTELSFTSFQGLMVAILYCFVNNEVQMEFRKYWER

hum LGTHEVIFAFVMDEHARGTLRFIKLFTELSFTSFQGLMVAILYCFVNNEVQLEFRKSWER

pan LGTHEVIFAFVMDEHARGTLRFIKLFTELSFTSFQGLMVAILYCFVNNEVQLEFRKSWER

**********: ****** ***:********:*************:*****:**** ***

gal WRLEHLYVQRDSSMKPLKCPANSISSGGTVGSSVYAATCQATFS

mus WRLEHLNIQRDCSMKPLKCPTSSVSSGATVGSSVYAATCQSSYS

hum WRLEHLHIQRDSSMKPLKCPTSSLSSGATAGSSMYTATCQASCS

pan WRLEHLHIQRDSSMKPLKCPTSSLSSGATAGSSMYTATCQASCS

****** :***.********:.*:***.*.***:*:****:: *

1. Antithrombin III

gal ---------------MHLFMVCLFGLWGMASPAPYAVEDICTAKPRDIPVNPICIYRNPE

mus MYSPGAGSGAAGERKLCLLSLLLIGALGCAICHGNPVDDICIAKPRDIPVNPLCIYRSPG

hum MYSNVIGTVTSGKRKVYLLSLLLIGFWDCVTCHGSPV-DICTAKPRDIPMNPMCIYRSPE

pan MYSNVIGTVTSGKRKVYLLSLLLIGFWDCVTCHGSPV-DICTAKPRDIPMNPMCIYRSPE

: *: : *:* . . * *** *******:**:****.*

gal KKPQESEVLEPGKGRIPEFTNPRVWELSRANSRFAVVFYKHLADSKDNEENIFLSPLSIS

mus KKATEED---GSEQKVPEATNRRVWELSKANSRFATNFYQHLADSKNDNDNIFLSPLSIS

hum KKATEDE---GSEQKIPEATNRRVWELSKANSRFATTFYQHLADSKNDNDNIFLSPLSIS

pan KKATEDE---GSEQKIPEATNRRVWELSKANSRFATTFYQHLADSKNDNDNIFLSPLSIS

** *.: .: ::** ** ******:******. **:******::::**********

gal TAFAMTKLGACGDTLQQLMEVFQFDTISEKTSDQVHFFFAKLNCRLYKKANKSSELISAN

mus TAFAMTKLGACNDTLKQLMEVFKFDTISEKTSDQIHFFFAKLNCRLYRKANKSSDLVSAN

hum TAFAMTKLGAC**N**DTLQQLMEVFKFDTISEKTSDQIHFFFAKLNCRLYRKA**N**KSSKLVSAN

pan TAFAMTKLGACNDTLQQLMEVFKFDTISEKTSDQIHFFFAKLNCRLYRKANKSSKLVSAN

***********.***:******:***********:************:******.*:***

gal RLFGEKSLVFNETYQNISEIVYGAKLWPLNFKEKPELSRKIINEWVANKTERRITEVIPE

mus RLFGDKSLTFNESYQDVSEVVYGAKLQPLDFKENPEQSRVTINNWVANKTEGRIKDVIPQ

hum RLFGDKSLTF**N**ETYQDISELVYGAKLQPLDFKENAEQSRAAINKWVS**N**KTEGRITDVIPS

pan RLFGDKSLTFNETYQDISELVYGAKLQPLDFKENAEQSRAAINKWVSNKTEGRITDVIPS

****:***.***:**::**:****** **:***: * ** **:**:**** **.:***.

gal KGIDDLTVLVLVNTIYFKGHWKSQFPAPNTRLDLFHKANGETCNVPIMYQESRFRYAFIQ

mus GAINELTALVLVNTIYFKGLWKSKFSPENTRKEPFYKVDGQSCPVPMMYQEGKFKYRRVA

hum EAINELTVLVLVNTIYFKGLWKSKFSPENTRKELFYKADGESCSASMMYQEGKFRYRRVA

pan EAINELTVLVLVNTIYFKGLWKSKFSPENTRKELFYKADGESCSASMMYQEGKFRYRRVA

.*::**.*********** ***:* *** : *:*.:*::* . :****.:*:* :

gal EDKVQVLELPYKGDDITMVLVLPKAGTPLVEVERDLTSDKLQDWIDSMMEVSLTVSFPRF

mus E-GTQVLELPFKGDDITMVLILPKPEKSLAKVEQELTPELLQEWLDELSETMLVVHMPRF

hum E-GTQVLELPFKGDDITMVLILPKPEKSLAKVEKELTPEVLQEWLDELEEMMLVVHMPRF

pan E-GTQVLELPFKGDDITMVLILPKPEKSLVKVEKELTPEVLQEWLDELEEMMLVVHMPRF

* .******:*********:*** . *.:**::** : **:*:*.: * *.* :***

gal RVEDSFSVKEKLRKMGLEDLFSPENAKLPGIVAGDRTDLYVSEAFHKAFLEVNEEGSEAS

mus RTEDGFSLKEQLQDMGLIDLFSPEKSQLPGIVAGGRDDLYVSDAFHKAFLEVNEEGSEAA

hum RIEDGFSLKEQLQDMGLVDLFSPEKSKLPGIVAEGRDDLYVSDAFHKAFLEVNEEGSEAA

pan RIEDSFSLKEQLQDMGLVDLFSPEKSKLPGIVAEGRDDLYVSDAFHKAFLEVNEEGSEAA

* **.**:**:*:.*** ******:::****** .* *****:****************:

gal AATAVVISGRSFPMNRIIFEANRPFLLFIREATLNTIIFMGRISDPCS-

mus ASTSVVITGRSLNPNRVTFKANRPFLVLIREVALNTIIFMGRVANPCVN

hum ASTAVVIAGRSLNPNRVTFKANRPFLVFIREVPLNTIIFMGRVANPCVK

pan ASTAVVIAGRSLNPNRVTFKANRPFLVFIREVPLNTIIFMGRVANPCVK

*:*:***:***: **: *:******::***. *********:::**

1. Glycine receptor alpha-3

gal MPRTRALQGSSAASSSMARLRYLRAAVSGFYLWEAAVLLSLVATKETDSARSRSMPMSPS

hum ----------------MAHVRHFRTLVSGFYFWEAALLLSLVATKETDSARSRSAPMSPS

pan ----------------MAHVRHFRTLVSGFYFWEAALLLSLVATKETDSARSRSAPMSPS

mus ----------------MAHVRHFRTLVSGFYFWEAALLLSLVATKETNSARSRSAPMSPS

**::*::*: *****:****:**********:****** *****

gal DFLDKLMGRMSGYDARIRPNFKGPPVNVTCNIFINSFGSIAETTMDYRVNIFLRQNWNDP

hum DFLDKLMGRTSGYDARIRPNFKGPPV**N**VTCNIFINSFGSIAETTMDYRVNIFLRQKWNDP

pan DFLDKLMGRTSGYDARIRPNFKGPPVNVTCNIFINSFGSIAETTMDYRVNIFLRQKWNDP

mus DFLDKLMGRTSGYDARIRPNFKGPPVNVTCNIFINSFGSIAETTMDYRVNIFLRQKWNDP

********* *********************************************:****

gal RLAYSEYPDDSLDLDPSMLDSIWKPDLFFANEKGANFHEVTTDNKLLRIFKNGNVLYSIR

hum RLAYSEYPDDSLDLDPSMLDSIWKPDLFFANEKGANFHEVTTDNKLLRIFKNGNVLYSIR

pan RLAYSEYPDDSLDLDPSMLDSIWKPDLFFANEKGANFHEVTTDNKLLRIFKNGNVLYSIR

mus RLAYSEYPDDSLDLDPSMLDSIWKPDLFFANEKGANFHEVTTDNKLLRIFKNGNVLYSIR

************************************************************

gal LTLILSCPMDLKNFPMDVQTCIMQLESFGYTMNDLIFEWQEKGAVQVAEGLTLPQFLLKE

hum LTLTLSCPMDLKNFPMDVQTCIMQLESFGYTMNDLIFEWQDEAPVQVAEGLTLPQFLLKE

pan LTLTLSCPMDLKNFPMDVQTCIMQLESFGYTMNDLIFEWQDEAPVQVAEGLTLPQFLLKE

mus LTLTLSCPMDLKNFPMDVQTCIMQLESFGYTMNDLIFEWQDEAPVQVAEGLTLPQFLLKE

*** ************************************::. ****************

gal EKDLCYCTKHYNTGKFTCIEVRFHLERQMGYYLIQMYIPSLLIVILSWVSFWINMDAAPA

hum EKDLRYCTKHYNTGKFTCIEVRFHLERQMGYYLIQMYIPSLLIVILSWVSFWINMDAAPA

pan EKDLRYCTKHYNTGKFTCIEVRFHLERQMGYYLIQMYIPSLLIVILSWVSFWINMDAAPA

mus EKDLRYCTKHYNTGKFTCIEVRFHLERQMGYYLIQMYIPSLLIVILSWVSFWINMDAAPA

**** *******************************************************

gal RVALGITTVLTMTTQSSGSRASLPKVSYVKAIDIWMAVCLLFVFSALLEYAAVNFVSRQH

hum RVALGITTVLTMTTQSSGSRASLPKVSYVKAIDIWMAVCLLFVFSALLEYAAVNFVSRQH

pan RVALGITTVLTMTTQSSGSRASLPKVSYVKAIDIWMAVCLLFVFSALLEYAAVNFVSRQH

mus RVALGITTVLTMTTQSSGSRASLPKVSYVKAIDIWMAVCLLFVFSALLEYAAVNFVSRQH

************************************************************

gal KELLRFRRKRKKNKYFEGVLDGNEINESLQHSNGLRCAGPIDGTLPQIYSTNLRDDDARE

hum KELLRFRRKRKNKTEA--------------------------FALEKFYRFSDMDDEVRE

pan KELLRFRRKRKNKTEA--------------------------FALEKFYRFSDMDDEVRE

mus KELLRFRRKRKNKTEA--------------------------FALEKFYRFSDTDDEVRE

***********::. :* ::* . **:.**

gal SQFSFTAYGVGPCLQAKDGVAQKGPNNPAQAAPKSPDEMRKVFIDRAKKIDTISRACFPL

hum SRFSFTAYGMGPCLQAKDGMTPKGPNHPVQVMPKSPDEMRKVFIDRAKKIDTISRACFPL

pan SRFSFTAYGMGPCLQAKDGMTPKGPNHPVQVMPKSPDEMRKVFIDRAKKIDTISRACFPL

mus SRFSFTAYGMGPCLQAKDGVVPKGPNHAVQVMPKSPDEMRKVFIDRAKKIDTISRACFPL

*:*******:*********:. ****: .*. ****************************

gal AFLIFNIFYWVIYKIIRHEDVHQ---

hum AFLIFNIFYWVIYKILRHEDIHQQQD

pan AFLIFNIFYWVIYKILRHEDIHQQQD

mus AFLIFNIFYWVIYKILRHEDIHQQQD

***************:****:**

1. Chitotriosidase-1

gal ----------MAKLILITGLALLLNAQIGSTYVLSCYFTNWAQYRPGLGKYMPDNIDPCL

mus ----------MVQSLAWAGVMTLLMVQWGSAAKLVCYLTNWSQYRTEAVRFFPRDVDPNL

hum ----------MVRSVAWAGFMVLLMIPWGSAAKLVCYFTNWAQYRQGEARFLPKDLDPSL

pan MGCSLPLSCIMVRSVAWAGFMVLLMIPWGSAAKLVCYFTNWAQYRQGEARFLPKDVDPSL

*.: : :*. ** **: * **:***:*** :::* ::** *

gal CDHLIYAFAGMSNNEITTYEWNDETLYKSFNGLKNQNGNLKTLLAIGGWNFGTAKFSTMV

mus CTHVIFAFAGMDNHQLSTVEHNDELLYQELNSLKTKNPKLKTLLAVGGWTFGTQKFTDMV

hum CTHLIYAFAGMTNHQLSTTEWNDETLYQEFNGLKKMNPKLKTLLAIGGW**N**FGTQKFTDMV

pan CTHLIYAFAGMTNHQLSTIEWNDETLYQEFNGLKKMNPKLKTLLAIGGWNFGTQKFTDMV

* *:*:***** *::::* * *** **:.:*.**. * :******:***.*** **: **

gal STPENRQTFINSVIKFLRQYQFDGLDIDWEYPGSKGSPSQDKGLFTVLVQEMLAAFEQEA

mus ATASNRQTFVKSALSFLRTQGFDGLDLDWEFPGGRGSPTVDKERFTALIQDLAKAFQEEA

hum ATANNRQTFVNSAIRFLRKYSFDGLDLDWEYPGSQGSPAVDKERFTTLVQDLANAFQQEA

pan ATANNRQTFVNSAIRFLRKYSFDGLDLDWEYPGSRGSPAVDKERFTALVQDLANAFQQEA

:* .*****::*.: *** *****:***:**.:***: ** **.*:*:: **::**

gal KQVNKPRLMITAAVAAGLSNIQAGYQIAELGKYLDYFHVMTYDFHGSWDGQTGENSPLYK

mus QSSGKERLLLTAAVPSDRGLVDAGYEVDKIAQSLDFINLMAYDFHSSLEKTTGHNSPLYK

hum QTSGKERLLLSAAVPAGQTYVDAGYEVDKIAQNLDFVNLMAYDFHGSWEKVTGHNSPLYK

pan QTSGKERLLLSAAVPAGQTYVDAGYEVDKIAQNLDFVNLMAYDFHGSWEKVTGHNSPLYK

: .* **:::*** :. ::***:: ::.: **:.::*:****.* : **.******

gal GPADTGDLIYFNVDYAMNYWKSNGAPAEKLLVGFPTYGHSYILKNPSDTAVGAPTSGPGP

mus RQGESGAAAEQNVDAAVTLWLQKGTPASKLILGMPTYGRSFTLASSSDNGVGAPATGPGA

hum RQEESGAAASLNVDAAVQQWLQKGTPASKLILGMPTYGRSFTLASSSDTRVGAPATGSGT

pan RQEESGAAASLNVDAAVQQWLQKGTPASKLILGMPTYGRSFTLASSSDTRVGAPATGSGT

::* *** *: * .:*:**.**::*:****:*: * . **. ****::* *

gal AGPYTRQSGFLAYYEICTFLDSGATQAWDAPQDVPYAYKSSEWVGYGNIKSFNIKIDWLK

mus PGPYTKDKGVLAYYEACSWKE----RHRIEDQKVPYAFQDNQWVSFDDVESFKAKAAYLK

hum PGPFTKEGGMLAYYEVCSWKGAT--KQRIQDQKVPYIFRDNQWVGFDDVESFKTKVSYLK

pan PGPFTKEGGMLAYYEVCSWKGAT--KQRIQDQKVPYIFRDNQWVGFDDVESFKTKVSYLK

**:*:: *.***** *:: : *.*** ::..:**.:.:::**: * :**

gal KNNYGGAMVWSLDMDDFTGTFCKQGKYPLITTLKNALGQQSSSCVPPAQPNPPITAAPST

mus QKGLGGAMVWVLDLDDFKGSFCNQGPYPLIRTLRQELNLPSETPRSPEQ------IIPEP

hum QKGLGGAMVWALDLDDFAGFSCNQGRYPLIQTLRQELSLPYLPSGTPEL------EVPKP

pan QKGLGGAMVWALDLDDFAGFSCNQGRYPLIQTLRQELSLPYLPSGTPEL------EVPKP

::. ****** **:*** * *:** **** **:: *. * *.

gal GSGSGSGSGSGSSGSNTGSSGGSGFCAGKANGIYADPTNKSKFYNCNNGETFVQSCQAGL

mus RPS--------SMPEQGPSPGLDNFCQGKADGVYPNPGDESTYYNCGGGRLFQQSCPPGL

hum GQP--------SEPEHGPSPGQDTFCQGKADGLYPNPRERSSFYSCAAGRLFQQSCPTGL

pan GQP--------SEPEHGPSPGQDTFCQGKADGLYPNPRERSSFYSCAGGRLFQQSCPTGL

* .: * * . ** ***:*:* :* :.*.:*.* *. * *** **

gal VFDSSCSCCNWA

mus VFRASCKCCTWS

hum VFSNSCKCCTWN

pan VFSSSCKCCTWN

** **.**.*

1. Coagulation factor VII

gal ----------------------------------------------------MVSRQCVA

mus ----------------------------------------------------MVPQAHGL

hum ------------------------------------------------------MVSQAL

pan MATGRPGAALSWGVQRTPLSSPPPSLCHPWRQRTLPVSPMGNVNRQGQHCRDFIMVSQAL

gal LLLCFPLLV----------------------PPSLEAVFLKQEEANSIFQRHRRANSFFE

mus LLLCFLLQLQGPL---------------------GTAVFITQEEAHGVLHRQRRANSLLE

hum RLLCLLLGLQGCLAAGGVAKASGGETRDMPWKPGPHRVFVTQEEAHGVLHRRRRANAFLE

pan RLLCLLLGLQGCLAAGGVAEASGGETRDMPWKPGPHRVFITQEEAHGVLHRRRRANAFLE

***: * : **:.****:.:::*:****:::*

gal EIKLGPLERECIEEKCSFEEAREIYRDDERTKEFWHIYSDPNQCDSSPCQNGGSCDDQFQ

mus ELWPGSLERECNEEQCSFEEAREIFKSPERTKQFWIVYSDGDQCASNPCQNGGTCQDHLK

hum ELRPGSLERECKEEQCSFEEAREIFKDAERTKLFWISYSDGDQCASSPCQNGGSCKDQLQ

pan ELRPGSLERECKEEQCSFEEAREIFKDLERTKLFWISYSDGDQCASSPCQNGGSCKDQLQ

*: * ***** **:*********::. **** ** *** :** *.******:*.*:::

gal DYVCRCPPEYEGKSCETAVAENLKCIYDNGGCEQYCADEQSEKRVCFCAEGYALASDGVS

mus SYVCFCLLDFEGRNCEKSKNEQLICANENGDCDQYCRDHVGTKRTCSCHEDYTLQPDEVS

hum SYICFCLPAFEGRNCETHKDDQLICVNENGGCEQYCSDHTGTKRSCRCHEGYSLLADGVS

pan SYICFCLPAFEGRNCETYKDDQLICVNENGGCEQYCSDHTGTKRSCRCHEGYSLLADGVS

.*:* * :**:.**. ::* * :**.*:*** *. . ** * * *.*:* * **

gal CIPQVKYPCGTIPVLARKNT-TAQGRIVGGVTCPPGECPWQALIIQDQKGKCGGSLLSPE

mus CKPKVEYPCGRIPVVEKRNSSSRQGRIVGGNVCPKGECPWQAVLKINGLLLCGAVLLDAR

hum CTPTVEYPCGKIPILEKR**N**ASKPQGRIVGGKVCPKGECPWQVLLLVNGAQLCGGTLINTI

pan CTPTVEYPCGKIPILEKRNASKPQGRIVGGKVCPKGECPWQVLLLVNGAQLCGGTLINTI

* * *:**** **:: ::*: . ******* .** ******.:: : **. *:.

gal WVVTAAHCLDYAHS-KQLRVRLGEYSVKVAEKTEQESGVSKIIRHEEYTIGQVNHDIALL

mus WIVTAAHCFDNIRYWGNITVVMGEHDFSEKDGDEQVRRVTQVIMPDKYIRGKINHDIALL

hum WVVSAAHCFDKIKNWRNLIAVLGEHDLSEHDGDEQSRRVAQVIIPSTYVPGTTNHDIALL

pan WVVSAAHCFDKIKNWRNLIAVLGEHDLSEHDGDEQSRRVAQVIIPSTYIPGTTNHDIALL

*:*:****:* : :: . :**:... : ** *:::* . * * *******

gal KLETPVNLTDFVVPICLPEKRFAVYELSSIKFSMVSGWGRLLDGGATSTFLMRVHLPRVK

mus RLHRPVTFTDYVVPLCLPEKSFSENTLARIRFSRVSGWGQLLDRGATALELMSIEVPRLM

hum RLHQPVVLTDHVVPLCLPERTFSERTLAFVRFSLVSGWGQLLDRGATALELMVLNVPRLM

pan RLHQPVVLTDHVVPLCLPERAFSERTLAFVRFSLVSGWGQLLDRGATALELMVLNVPRLM

:*. ** :**.***:****: *: *: ::** *****:*** ***: ** :.:**:

gal TQECEKQA-----NLNITENMFCAGDLTGKKDSCKGDSGGPHATKYKNTWFLTGIVSWGK

mus TQDCLEHAKHSSNTPKITENMFCAGYMDGTKDACKGDSGGPHATHYHGTWYLTGVVSWGE

hum TQDCLQQSRKVGDSP**N**ITEYMFCAGYSDGSKDSCKGDSGGPHATHYRGTWYLTGIVSWGQ

pan TQDCLQQSRKVGDSPNITEYMFCAGYSDGSKDSCKGDSGGPHATHYRGTWYLTGIVSWGQ

**:* ::: . :*** ***** *.**:***********:*:.**:***:****:

gal GCAVEGSYGVYTRVSRYINWLKRHME--------------

mus GCAAIGHIGVYTRVSQYIDWLVRHMDSKLQVGVFRLPLL-

hum GCATVGHFGVYTRVSQYIEWLQKLMRSEPRPGVLLRAPFP

pan GCASVGHFGVYTRVSQYIEWLQKLMRSEPRPGVLLRAPFP

*** * *******:**:** : *

1. Tissue alpha-L-fucosidase

gal --------MAAG-------GVLWLAAALGPGLAAPRYSPDWASLDARPLPAWFDQAKVGV

mus --------------MLLLLLLLLVAAAQAVALAPRRFTPDWQSLDSRPLPSWFDEAKFGV

hum MRAPGMRSRPAGPALLLLLLFLGAAESVRRAQPPRRYTPDWPSLDSRPLPAWFDEAKFGV

pan MRAPGMRSRPAGPALLLLLLFLEAAESVRRAQPRRRYTPDWPSLDSRPLPAWFDEAKFGV

.* * : . *::*** ***:****:***:**.**

gal FVHWGVFSVPAWGSEWFWWHWQGEHRADYERFMQQRFPPAASYADFAPHFTAYDFQPHEW

mus FVHWGVFSVPAWGSEWFWWHWQGDRMPAYQRFMTENYPPGFSYADFAPQFTARFFHPDQW

hum FIHWGVFSVPAWGSEWFWWHWQGEGRPQYQRFMRDNYPPGFSYADFGPQFTARFFHPEEW

pan FIHWGVFSVPAWGSEWFWWHWQGEGRPQYQRFMRDNYPPGFSYADFGPQFTARFFHPEEW

*:*********************: *:*** :.:**. *****.*:*** *:*.:*

gal ARLFQRAGARYVVLTTKHHEGFTNWGSPVSWNWNSLDTGPHRDLVGELGQALRENNIRYG

mus AELFQAAGAKYVVLTTKHHEGFTNWPSPVSWNWNSKDVGPHRDLVGELGAAVRKRNIRYG

hum ADLFQAAGAKYVVLTTKHHEGFTNWPSPVSWNWNSKDVGPHRDLVGELGTALRKRNIRYG

pan ADLFQAAGAKYVVLTTKHHEGFTNWPSPVSWNWNSKDVGPHRDLVGELGTALRKRNIRYG

* *** ***:*************** ********* *.*********** *:*:.*****

gal LYHSLLEWFNPLYLSDKESGFKTQNFVLKKTMPELYDLVLKYKPDLIWSDGDWEAPDSYW

mus LYHSLLEWFHPLYLLDKKNGFKTQHFVRAKTMPELYDLVNSYKPDLIWSDGEWECPDTYW

hum LYHSLLEWFHPLYLLDKKNGFKTQHFVSAKTMPELYDLVNSYKPDLIWSDGEWECPDTYW

pan LYHSLLEWFHPLYLLDKKNGFKTQHFVSAKTMPELYDLVNSYKPDLIWSDGEWECPDTYW

*********:**** **:.*****:** ********** .**********:**.**:**

gal NSTSFLAWLYNNSPVKDTVVVNDRWCNNCSCHHGGFYNCADKYKPGTLLAHKWEMCSSID

mus NSTSFLAWLYNDSPVKDEVIVNDRWGQNCSCHHGGYYNCQDKYKPQSLPDHKWEMCTSMD

hum **N**STNFLSWLYNDSPVKDEVVVNDRWGQ**N**CSCHHGGYYNCEDKFKPQSLPDHKWEMCTSID

pan NSTNFLSWLYNDSPVKDEVVVNDRWGQNCSCHHGGYYNCEDKFKPQSLPDHKWEMCTSID

***.**:****:***** *:***** :********:*** **:** :* ******:*:*

gal KISWGYRSNMHIDELMDVASIIEELVQTVSFGGNYLLNVGPTKEGVIVPIFQERLLALGR

mus RASWGYRKDMTMSTIAKENEIIEELVQTVSLGGNYLLNIGPTKDGLIVPIFQERLLAVGK

hum KFSWGYRRDMALSDVTEESEIISELVQTVSLGGNYLLNIGPTKDGLIVPIFQERLLAVGK

pan KFSWGYRRDMAMSDVTEESEIISELVQTVSLGGNYLLNIGPTKDGLIVPIFQERLLAVGK

: ***** :* :. : . .**.*******:*******:****:*:***********:*:

gal WLDTNGEAIYESKPWRVQMENSTDTVWYTSKGAVVYAIFLTWPQNSVLQLSVPTPSPATQ

mus WLQINGEAIYASKPWRVQSEKNKTVVWYTSKNATVYATFLYWPENGIVNLKSPKTTSATK

hum WLSINGEAIYASKPWRVQWEK**N**TTSVWYTSKGSAVYAIFLHWPENGVLNLESPITTSTTK

pan WLSINGEAIYASKPWRVQWEKNTTSVWYTSKGSAVYAIFLHWPENGVLNLESPITTSTTK

**. ****** ******* *:.. ******.:.*** ** **:*.:::*. * : :*:

gal VTMLGFAGTLQWQQPPGEGLLVTLPDAPPSPVRSQPGWAVRLEGVK

mus ITMLGLEGDLSWTQDPLEGVLISLPQLPPTVLPVEFAWTLKLTKVN

hum ITMLGIQGDLKWSTDPDKGLFISLPQLPPSAVPAEFAWTIKLTGVK

pan ITMLGIQGDLKWSTDPDKGLLISLPQLPPSAVPAEFAWTIKLTGVK

:****: * *.* * :*::::**: **: : : .*:::* *:

1. Gastric inhibitory polypeptide receptor

gal MRDAPGPGYVVHLAWMCL----FSAVPHGGAKVLERTFEEWMRYRDECLRRMASEPYPAG

mus ---MPLRL-LLLLLWLWGL--QWAETDSEGQTTTGELYQRWEHYGQECQKMLETTEPPSG

hum MTTSPILQ-LLLRLSLCGLLLQRAETGSKG-QTAGELYQRWERYRRECQETLAAAEPPSG

pan MTTSPILQ-RLLRLSLCGLLLQRAETGSKG-QTAGELYQRWERYRRECQETLAAAEPPSG

* : : : . * . . ::.* :* ** . : : *:*

gal LFCNRTFDMYACWPDGSPGTAVNVSCPFYLPWFEKVKHGLVSRRCGADGQWVTVNGSQPW

mus LACNGSFDMYACWNYTAANTTARVSCPWYLPWFRQVSAGFVFRQCGSDGQWG------SW

hum LAC**N**GSFDMYVCWDYAAP**N**ATARASCPWYLPWHHHVAAGFVLRQCGSDGQWG------LW

pan LACNGSFDMYVCWDYAAPNATARASCPWYLPWHHHVAAGFVLRQCGSDGQWG------LW

* ** :****.** : .::...***:****..:* *:* *:**:**** *

gal RDYSQCEEELEDGAEEEGARRLMVSFKVLYTVGYALSLLTLVSALLVLTVFRKLHCTRNY

mus RDHTQCENPEKNGAFQD-QTLILERLQIMYTVGYSLSLTTLLLALLILSLFRRLHCTRNY

hum RDHTQCENPEKNEAFLD-QRLILERLQVMYTVGYSLSLATLLLALLILSLFRRLHCTRNY

pan RDHTQCENPEKNEAFLD-QRLILERLQVMYTVGYSLSLATLLLALLILSLFRRLHCTRNY

**::***: :: * : :: ::::*****:*** **: ***:*::**:*******

gal IHANLFASFGLRATSVMVKDALLERRWGAEVLQVADWQALLSHEAALGCRAAQVLMQYCI

mus IHMNLFTSFMLRAAAILTRDQLLPPLGPY----TGDQAPTPWNQALAACRTAQIMTQYCV

hum IHINLFTSFMLRAAAILSRDRLLPRPGPY----LGDQALALWNQALAACRTAQIVTQYCV

pan IHINLFTSFMLRAAAILSRDRLLPQPGPY----LGDQAPALWNQALAACRTAQIVTQYCV

** ***:** ***:::: :* ** .* ::* .**:**:: ***:

gal LANHYWFLVEAVYLYKLLIGAVFSEKNYYRLYLYLGWGTPVVFVVPWMAAKYLKENAECW

mus GANYTWLLVEGVYLHHLLVIVGRSEKGHFRCYLLLGWGAPALFVIPWVIVRYLRENTQCW

hum GANYTWLLVEGVYLHSLLVLVGGSEEGHFRYYLLLGWGAPALFVIPWVIVRYLYENTQCW

pan GANYTWLLVEGVYLHSLLVLVGGSEEGHFRYYLLLGWGAPALFVIPWVIVRYLYENTQCW

**: *:***.***: **: . **:.::* ** ****:*.:**:**: .:** **::**

gal ALNENMAYWWIIRIPILLASMINLLIFMRILKVILAKLRANQKGYADYKLRLAKATLTLI

mus ERNEVKAIWWIIRTPILITILINFLIFIRILGILVSKLRTRQMRCPDYRLRLARSTLTLV

hum ERNEVKAIWWIIRTPILMTILINFLIFIRILGILLSKLRTRQMRCRDYRLRLARSTLTLV

pan ERNEVKAIWWIIRTPILMTILINFLIFIRILGILLSKLRTRQMRCRDYRLRLARSTLTLV

** * ***** ***:: :**:***:*** ::::***:.* **:****::****:

gal PLFGIHEVVFIFATDEQTTGILRYIKVFFTLFLNSFQGFLVAVLYCFANKEVKSEMKKKW

mus PLLGVHEVVFAPVTEEQVEGSLRFAKLAFEIFLSSFQGFLVSVLYCFINKEVQSEIRQGW

hum PLLGVHEVVFAPVTEEQARGALRFAKLGFEIFLSSFQGFLVSVLYCFINKEVQSEIRRGW

pan PLLGVHEVVFAPVTEEQARGALRFAKLGFEIFLSSFQGFLVSVLYCFINKEVQSEIRRGW

**:*:***** .*:**. * **: *: * :**.*******:***** ****:**::: *

gal QLWKLDHPALCCTQ----------------------------------------------

mus RHRRLRLSLQEQRPRPHQELAPRAVPLSSACREAAVGNALPSGMLHVPGDE---VLESYC

hum HHCRLRRSLGEEQRQ-LPERAFRALPSGSGPGEVPTSRGLSSGTLPGPGNEASRELESYC

pan HHCRLRRSLGEEQRQ-LPERAFRALPSGSGPGEVPTSRGLSSGTLPGPGNEASRELESYC

: :*

1. ERO1-like protein beta

gal MSGGGSGSRSTAAWARWATRLLAALGS-VLAAEAQLTGVLDDCLCDIESIDDFNTFKIFP

mus MSPGFRRA-VTGQGAAAAVQLLVTLSFLSSLVKTQVTGVLDDCLCDIDSIDKFNTYKIFP

hum MSQGVRRA-GAGQGVAAAVQLLVTLSFLRSVVEAQVTGVLDDCLCDIDSIDNFNTYKIFP

pan MSQGVRRA-GAGQGVAAAVQLLVTLSFLRSVVEAQVTGVLDDCLCDIDSIDNFNTYKIFP

** * : :. . *.:**.:*. .::*:***********:***.***:****

gal KIQKLQERDYFRYYKVNLKRPCPFWADDGHCSIKDCHVEPCPESKIPVGIKAGSSNKYSK

mus KIKKLQERDYFRYYKVNLKRPCPFWAEDGHCSIKDCHVEPCPESKIPVGIKAGRSNKYSQ

hum KIKKLQERDYFRYYKVNLKRPCPFWAEDGHCSIKDCHVEPCPESKIPVGIKAGHSNKYLK

pan KIKKLQERDYFRYYKVNLKRPCPFWAEDGHCSIKDCHVEPCPESKIPVGIKAGHSNKYLK

**:***********************:************************** **** :

gal AANNSKELEDCEQANKLGAVNSTLSNQSKEAFIDWARYDDSQDHFCELDDERSPDAQYVD

mus AANSTKELDDCEQANKLGAINSTLSNESKEAFIDWARYDDSQDHFCELDDERSPAAQYVD

hum MA**N**NTKELEDCEQANKLGAI**N**STLS**N**QSKEAFIDWARYDDSRDHFCELDDERSPAAQYVD

pan MANNTKELEDCEQANKLGAINSTLSNQSKEAFIDWARYDDSRDHFCELDDERSPAAQYVD

**.:***:**********:******:**************:************ *****

gal LLLNPERYTGYKGPSAWRVWNSIYEENCFKPRSVYRPLNPLAPSRGGDDGESFYTWLEGL

mus LLLNPERYTGYKGSSAWRVWNSIYEENCFKPRSVYRPLNPLAPSRGEDDGESFYTWLEGL

hum LLLNPERYTGYKGTSAWRVWNSIYEENCFKPRSVYRPLNPLAPSRGEDDGESFYTWLEGL

pan LLLNPERYTGYKGTSAWRVWNSIYEENCFKPRSVYRPLNPLAPSRGEDDGESFYTWLEGL

************* ******************************** *************

gal CLEKRVFYKLISGLHASINLHLCANYLLEETWGKPRWGPNVKEFTRRFDPIETKGEGPRR

mus CLEKRVFYKLISGLHASINLHLCANYLLEETWGKPSWGPNIKEFRRRFDPVETKGEGPRR

hum CLEKRVFYKLISGLHASINLHLCANYLLEETWGKPSWGPNIKEFKHRFDPVETKGEGPRR

pan CLEKRVFYKLISGLHASINLHLCANYLLEETWGKPSWGPNIKEFKRRFDPVETKGEGPRR

*********************************** ****:*** :****:*********

gal LKNLYFLYLIELRALSKVAPYFERSVVDLYTGNGHEDAESKALLLEIFRDTKSFHMHFDE

mus LKNLYFLYLIELRALSKVAPYFERSIVDLYTGNVEDDADTKTLLLSIFQDTKSFPMHFDE

hum LKNLYFLYLIELRALSKVAPYFERSIVDLYTGNAEEDADTKTLLLNIFQDTKSFPMHFDE

pan LKNLYFLYLIELRALSKVAPYFERSIVDLYTGNAEEDADTKTLLLNIFQDTKSFPMHFDE

*************************:******* .:**::*:***.**:***** *****

gal KSMFAGDKKGAKSLKEEFRLHFKNISRIMDCVGCDKCRLWGKLQTQGLGTALKILFSEKE

mus KSMFAGDKKGAKSLKEEFRLHFKNISRIMDCVGCDKCRLWGKLQTQGLGTALKILFSEKE

hum KSMFAGDKKGAKSLKEEFRLHFK**N**ISRIMDCVGCDKCRLWGKLQTQGLGTALKILFSEKE

pan KSMFAGDKKGAKSLKEEFRLHFKNISRIMDCVGCDKCRLWGKLQTQGLGTALKILFSEKE

************************************************************

gal IQNLPENSPSKGFQLTRQEIVALVNAFGRLSTSIKELQNFKVLLQQTR

mus IQNLPENSPSKGFQLTRQEIVALLNAFGRLSTSIRELQNFKALLQHRR

hum IQKLPENSPSKGFQLTRQEIVALLNAFGRLSTSIRDLQNFKVLLQHSR

pan IQKLPENSPSKGFQLTRQEIVALLNAFGRLSTSIRDLQNFKVLLQHSR

**:********************:**********::*****.***: *

1. Plasma alpha-L-fucosidase

gal ----------MSGLLPSVLL-LALPGLLGGRPRYEPTWASLDARPLPTWFDEAKFGVFIH

mus ------MRLGFLMLLPLLLLPLLRPWGVTRALSYDPTWESLDRRPLPAWFDQAKFGIFIH

hum MRPQELPRLAFPLLLLLLLLLPPPPCPAHSATRFDPTWESLDARQLPAWFDQAKFGIFIH

pan MRPQELPRLAFPLLL--LLLLPPPPCPAHSATRFDPTWESLDARQLPAWFDQAKFGIFIH

: ** :** * ::*** *** * **:***:****:***

gal WGVFAVPSFGSEWFWWYWQKEKREPYVKFMKANYPPGFSYEDFGPLFTAEFFDPNQWADI

mus WGVFSVPSFGSEWFWWYWQKEKKPQFVDFMNNNYAPGFKYEDFVVLFTAKYFNANQWADI

hum WGVFSVPSFGSEWFWWYWQKEKIPKYVEFMKDNYPPSFKYEDFGPLFTAKFFNANQWADI

pan WGVFSVPSFGSEWFWWYWQKEKIPKYVEFMKDNYPPSFKYEDFGPLFTAKFFNANQWADI

****:***************** :*.**: ** *.*.**** ****::*: ******

gal LKASGAKYVVLTSKHHEGFTLWGSKYSWNWNAVDVGPKRDLVAELETSVRNRTDLRFGLY

mus LQASGAKYVVFTSKHHEGFTMWGSDHSWNWNAVDEGPKRDIVKELEVAVRNRTGLHFGLY

hum FQASGAKYIVLTSKHHEGFTLWGSEYSWNWNAIDEGPKRDIVKELEVAIR**N**RTDLRFGLY

pan FQASGAKYIVLTSKHHEGFTLWGSEYSWNWNAIDEGPKRDIVKELEVAIRNRTDLRFGLY

::******:*:*********:***.:******:* *****:* ***.::****.*:****

gal HSLFEWFNPLFLEDASNVFKTRKFPTSKSLPELYEIVTKYQPEIVWSDGDGNAPDTYWNS

mus YSLFEWFHPLFLEDQSSSFQKQRFPVSKTLPELYELVNRYQPEVLWSDGDGGAPDHYWNS

hum YSLFEWFHPLFLEDESSSFHKRQFPVSKTLPELYELVNNYQPEVLWSDGDGGAPDQYW**N**S

pan YSLFEWFHPLFLEDESSSFHKRQFPVSKTLPELYELVNNYQPEVLWSDGDGGAPDQYWNS

:******:****** *. *:.::**.**:******:*..****::******.*** ****

gal TGFLAWLYNDSPVRDTVVTNDRWGAGGICKHGGFYTCSDRYNPGHLLPHKWENCMTIDKR

mus TGFLAWLYNESPVRKTVVTNDRWGVGSICKHGGYYTCSDRYNPGYLLPHKWENCMTIDKF

hum TGFLAWLYNESPVRGTVVTNDRWGAGSICKHGGFYTCSDRYNPGHLLPHKWENCMTIDKL

pan TGFLAWLYNESPVRGTVVTNDRWGAGSICKHGGFYTCSDRYNPGHLLPHKWENCMTIDKL

*********:**** *********.*.******:**********:**************

gal SWGYRRNANLEDYLTVEDLVKQLVETVSCGGNLLMNIGPTHDGRIAAIFEERLRQMGAWL

mus SWGYRREAEISDYLTIEELVKKLVETVACGGNLLMNIGPTGDGTIPVIFEERLRQMGTWL

hum SWGYRREAGISDYLTIEELVKQLVETVSCGGNLLMNIGPTLDGTISVVFEERLRQMGSWL

pan SWGYRREAGISDYLTIEELVKQLVETVSCGGNLLMNIGPTLDGTISVVFEERLRQMGSWL

******:* :.****:*:***:*****:************ ** * .:*********:**

gal KVNGEAIYETKPWRAQNDTVTPDVWYTFRPKEGKVNAIFLNWPNSGILELGEPQARLAET

mus KVNGEAIYETHTWRSQNDTVTPDVWYTSKPEKKLVYAIFLKWPISGKLFLGQPIGSLGET

hum KVNGEAIYETHTWRSQ**N**DTVTPDVWYTSKPKEKLVYAIFLKWPTSGQLFLGHPKAILGAT

pan KVNGEAIYETHTWRSQNDTVTPDVWYTSKPKEKLVYAIFLKWPTSGQLFLGHPKAILGAT

**********: **:************ :*:: * ****:** ** * **.* . *. *

gal QVKLVGYKEPLKWVALGEKGIAVALPQLTLKQLPCQWGWTLQLTDVN

mus EVELLGHWQPLTWTSSQPSGITVELPLLSVHQMPCKWGWTLVLSNVI

hum EVKLLGHGQPLNWISLEQNGIMVELPQLTIHQMPCKWGWALALTNVI

pan EVKLLGHGQPLNWISLEQNGIMVELPQLTIHQMPCKWGWALALTNVI

:*:*:*: :**.* : .** * ** *:::*:**:***:* *::*

1. Neutrophil collagenase

gal ---MKVLSLLLLLYAAVSSAFPVAPEKEDEGKNINLVETYLQNFYNLQKDHRPHLRQGGK

hum MFSLKTLPFLLLLHVQISKAFPVSS----KEKNTKTVQDYLEKFYQLPSNQYQSTRK**N**GT

pan MFSLKMLPFLLLLHVQISKAFPVSS----KEKNTKIVQDYLEKFYQLPSNQYQSTRKNGT

mus MFRLKTLPLLIFLHTQLANAFPVPEHL--EEKNIKTAENYLRKFYNLPSNQFRSSRN--A

:* * :*::*:. ::.**** : ** : .: **.:**:* .:: *:

gal NHLAEKLKEMQEFFGLQVTGKPDRDTLEMMNKPRCGVPDVEQYVFTPGNPKWKKNNLTYR

hum NVIVEKLKEMQRFFGL**N**VTGKPNEETLDMMKKPRCGVPDSGGFMLTPGNPKWERT**N**LTYR

pan NVIVEKLKEMQRFFGLNVTGKPNEETLDMMKKPRCGVPDSGGFMLTPGNPKWEHTNLTYR

mus TMVAEKLKEMQRFFSLAETGKLDAATMGIMEMPRCGVPDSGDFLLTPGSPKWTHTNLTYR

. :.*******.**.* *** : *: :*: ******* :::***.*** :.*****

gal IVNYTTKMRQTDVDEAIQKALKVWSSVTPLTFQKTEDKIADIMISFAYRDHNDNSPFDGP

hum IRNYTPQLSEAEVERAIKDAFELWSVASPLIFTRISQGEADINIAFYQRDHGDNSPFDGP

pan IRNYTPQLSEAEVERAIKDAFELWSVASPLIFTRISQGEADINIAFYQRDHGDNSPFDGP

mus IINHTPQLSRAEVKTAIEKAFHVWSVASPLTFTEILQGEADINIAFVSRDHGDNSPFDGP

* *:* :: .::*. **:.*:.:** .:** * . : *** *:* ***.********

gal NGLLAHAFQPGEGLGGDVHLDEEETWTKDGRGYNLFIVVAHELGHSLGLSHSNDPGALMY

hum NGILAHAFQPGQGIGGDAHFDAEETWT**N**TSANYNLFLVAAHEFGHSLGLAHSSDPGALMY

pan NGILAHAFQPGQGIGGDAHFDAEETWTDTSANYNLFLVAAHEFGHSLGLAHSSDPGALMY

mus NGILAHAFQPGQGIGGDAHFDSEETWTQDSKNYNLFLVAAHEFGHSLGLSHSTDPGALMY

**:********:*:***.*:* *****. . .****:*.***:******:**.*******

gal PNYAYTDPKEFLLPQDDIDGIQAIYGQSDDAVQPTGPTTPQVCDPNLTFDAITTLRGEMI

hum PNYAFRETS**N**YSLPQDDIDGIQAIYGLSSNPIQPTGPSTPKPCDPSLTFDAITTLRGEIL

pan PNYAFRETSNYSLPQDDIDGIQAIYGLSSNPIQPTGPSTPKPCDPSLTFDAITTLRGEIL

mus PNYAYREPSTYSLPQDDINGIQTIYGPSDNPIQPTGPSTPKACDPHLRFDATTTLRGEIY

****: : . : ******:***:*** *.: :*****:**: *** * *** ******:

gal FFKGRYMLRKHPERSETELNFISLFWPNLPSGIQAAYENIERDEVLLFKEDKYWVIRGYD

hum FFKDRYFWRRHPQLQRVEMNFISLFWPSLPTGIQAAYEDFDRDLIFLFKGNQYWALSGYD

pan FFKDRYFWRRHPQLQRVEMNFISLFWPSLPTGIQAAYEDFDRDLIFLFKGNQYWALSGYD

mus FFKDKYFWRRHPQLRTVDLNFISLFWPFLPNGLQAAYEDFDRDLVFLFKGRQYWALSGYD

***.:*: *:**: .::******** **.*:*****:::** ::*** :**.: ***

gal IVRGYPKPIYRLGFPKTVKRVNAAYNDETTGKTYFFVADRYWRYDENKKSMDHGYPRKII

hum ILQGYPKDISNYGFPSSVQAIDAAVF--YRSKTYFFVNDQFWRYDNQRQFMEPGYPKSIS

pan ILQGYPKDIANYGFPSSVQAIDAAVF--YRSKTYFFVNDQFWRYDNQRQFMEPGYPKSIS

mus LQQGYPRDISNYGFPRSVQAIDAAVS--YNGKTYFFINNQCWRYDNQRRSMDPGYPKSIP

: :***: * . *** :*: ::** .*****: :: ****:::: *: ***:.*

gal HDFGKI-GRVDAAFQKDGYVYFFHKTTQFQFDPRAKRIVSHMKSISWFNC---

hum GAFPGIESKVDAVFQQEHFFHVFSGPRYYAFDLIAQRVTRVARGNKWLNCRYG

pan GAFPGIESKVDAVFQQEHFFLFFSGPRYYAFDLIAQRVTRVARGNKWLNCRYG

mus SMFPGVNCRVDAVFLQDSFFLFFSGPQYFAFNFVSHRVTRVARSNLWLNCS--

* : :***.* :: :. .* : *: ::*:. :. *:**

1. Tubulointerstitial nephritis antigen

gal MANTALVPWV-LLWLLAAV----GSAARARTRRELSPGLYEHGVFDAGGSYCQRGDVCCR

hum ----MWRCPLGLLLLLPLAGHLALGAQQGRGRRELAPGLHLRGIRDAGGRYCQEQDLCCR

pan ----MWRCPLGLLLLLPLAGHLALGAQQGRGRRELAPGLHLRGIRDAGGRYCQEQDLCCR

mus ----MWGCWLGLLLLL-LAGQAALEARRSRWRRELAPGLHLRGIRDAGGRYCQEQDMCCR

: ** ** . * :.* ****:***: :*: **** ***. *:***

gal GRDDGCTVPYLDTICYCDLFCNRTVSDCCPDFWEYCLGIPAPFPKAPGCARSGRTYPSGA

hum GRADDCALPYLGAICYCDLFC**N**RTVSDCCPDFWDFCLGVPPPFPPIQGCMHGGRIYPVLG

pan GRADDCALPYLGAICYCDLFCNRTVSDCCPDFWDFCLGVPPPFPPIQGCMHGGRIYPVLG

mus GRADECALPYLGATCYCDLFCNRTVSDCCPDFWDFCLGIPPPFPPVQGCMHGGRIYPVFG

** * *::***.: *******************::***:* *** ** :.** ** .

gal TYRDNCNLCTCSPGGQWQCEDHACLMDGDLIDAVNRGNYGWRAANYSQFWGMTLEDGMRY

hum TYWDNCNRCTCQENRQWQCDQEPCLVDPDMIKAINQGNYGWQAG**N**HSAFWGMTLDEGIRY

pan TYWDNCNRCTCQENRQWQCDQEPCLVDPDMIKAINQGNYGWQAGNHSAFWGMTLDEGIRY

mus TYWDNCNRCTCHEGGHWECDQEPCLVDPDMIKAINRGNYGWQAGNHSAFWGMTLDEGIRY

** **** *** . :*:*::. **:* *:*.*:*:*****:*.*:* ******::*:**

gal RLGTFRPPPTVMNMNEMHMAMDSNEVLPRHFDAATKWPGMIHEPLDQGNCAGSWAFSTAA

hum RLGTIRPSSSVMNMHEIYTVLNPGEVLPTAFEASEKWPNLIHEPLDQGNCAGSWAFSTAA

pan RLGTIRPSSSVMNMHEIYTVLNPGEVLPTAFEASEKWPNLIHEPLDQGNCAGSWAFSTAA

mus RLGTIRPSSTVMNMNEIYTVLGQGEVLPTAFEASEKWPNLIHEPLDQGNCAGSWAFSTAA

****:** :****:*:: .:. .**** *:*: ***.:********************

gal VASDRISIHSMGHMTPSLSPQNLLSCDTRNQRGCSGGRLDGAWWYLRRRGVVTDECYPFT

hum VASDRVSIHSLGHMTPVLSPQNLLSCDTHQQQGCRGGRLDGAWWFLRRRGVVSDHCYPFS

pan VASDRVSIHSLGHMTPVLSPQNLLSCDTHQQQGCRGGRLDGAWWFLRRRGVVSDHCYPFS

mus VASDRVSIHSLGHMTPILSPQNLLSCDTHHQQGCRGGRLDGAWWFLRRRGVVSDNCYPFS

*****:****:***** ***********::*:** *********:*******:*.****:

gal SQDS--QPAAQPCMMHSRSTGRGKRQATARCPNPQTHANDIYQSTPAYRLAPSEKEIMKE

hum GRERDEAGPAPPCMMHSRAMGRGKRQATAHCPNSYVNNNDIYQVTPVYRLGSNDKEIMKE

pan GRERDEAGPAPPCMMHSRAMGRGKRQATAHCPNSYVNNNDIYQVTPVYRLGSNDKEIMKE

mus GREQNEASPTPRCMMHSRAMGRGKRQATSRCPNGQVDSNDIYQVTPAYRLGSDEKEIMKE

.:: : ******: ********::*** .. ***** **.***. .:******

gal LMENGPVQAILEVHEDFFLYKSGIYRHTAVAEGKGPKHQQHGTHSVKITGWGEEQLPDGQ

hum LMENGPVQALMEVHEDFFLYKGGIYSHTPVSLGRPERYRRHGTHSVKITGWGEETLPDGR

pan LMENGPVQALMEVHEDFFLYKGGIYSHTPVSLGRPERYRRHGTHSVKITGWGEETLPDGR

mus LMENGPVQALMEVHEDFFLYQRGIYSHTPVSQGRPEQYRRHGTHSVKITGWGEETLPDGR

*********::*********: *** ** *: *: ::::************** ****:

gal VQKYWTAANSWGRAWGEDGHFRIARGVNECEVESFVVGVWGRVSVEDMPHK

hum TLKYWTAANSWGPAWGERGHFRIVRGVNECDIESFVLGVWGRVGMEDMGHH

pan TLKYWTAANSWGPAWGERGHFRIVRGVNECDIESFVLGVWGRVGMEDMGHH

mus TIKYWTAANSWGPWWGERGHFRIVRGTNECDIETFVLGVWGRVGMEDMGHH

. ********** *** *****.**.***::*:**:******.:*** *:

1. ERO 1 like protein alpha

gal MAAG-GALLRALGFAALLL-PALPRGSPGSAERRCFCQVTGHLDDCTCDVETIDAFNNYK

mus MGRAWGLLVGLLGVVWLLRLGHGEERRPETAAQRCFCQVSGYLDDCTCDVETIDKFNNYR

hum MGRGWGFLFGLLGAVWLLSSGHGEEQPPETAAQRCFCQVSGYLDDCTCDVETIDRFNNYR

pan MGRGWGFLFGLLGAVWLLSLGHGEEQPPETAAQRCFCQVSGYLDDCTCDVETIDRFNNYR

*. . * *. ** . ** . * :* :******:*:************ ****:

gal LFPRLNQLLESDYFRYYKVNLKKPCPFWNDNSHCGIRDCAVKPCPSDEVPDGIRSAGYKY

mus LFPRLQKLLESDYFRYYKVNLKKPCPFWNDINQCGRRDCAVKPCHSDEVPDGIKSASYKY

hum LFPRLQKLLESDYFRYYKVNLKRPCPFWNDISQCGRRDCAVKPCQSDEVPDGIKSASYKY

pan LFPRLQKLLESDYFRYYKVNLKRPCPFWNDISQCGRRDCAVKPCQSDEVPDGIKSASYKY

*****::***************:******* .:** ******** ********:**.***

gal SEEANSRAEECEEANRLGAVDESLSKETQQAVLQWTWHDDSSDSFCEADDIHSPDAEYVD

mus SEEAN-RIEECEQAERLGAVDESLSEETQKAVLQWTKHDDSSDSFCEIDDIQSPDAEYVD

hum SEEANNLIEECEQAERLGAVDESLSEETQKAVLQWTKHDDSSDNFCEADDIQSPEAEYVD

pan SEEANNLIEECEQAERLGAVDESLSEETQKAVLQWTKHDDSSDNFCEADDIQSPEAEYVD

***** ****:*:**********:***:****** ******.*** ***:**:*****

gal LLLNPERYTGYKGPDAWKIWNSIYEENCFKPQNVKRP---LTSGRGDDGGQMFYKWLKGV

mus LLLNPERYTGYKGPDAWRIWSVIYEENCFKPQTIQRP---LASGRGKSKENTFYNWLEGL

hum LLLNPERYTGYKGPDAWKIWNVIYEENCFKPQTIKRPLNPLASGQGTSEENTFYSWLEGL

pan LLLNPERYTGYKGPDAWKIWNVIYEENCFKPQTIKRPLNPLASGQGKSEENTFYSWLEGL

*****************:**. **********.::** *:**:* . : **.**:*:

gal CVEKRAFYRLISGLHASINIHLSARYLLQDTWSEKKWGPNVTEFQQRFDEVVTRGEGPRR

mus CVEKRAFYRLISGLHASINVHLSARYLLQDTWLEKKWGHNVTEFQQRFDGILTEGEGPRR

hum CVEKRAFYRLISGLHASINVHLSARYLLQETWLEKKWGH**N**ITEFQQRFDGILTEGEGPRR

pan CVEKRAFYRLISGLHASINVHLSARYLLQETWLEKKWGHNITEFQQRFDGILTEGEGPRR

*******************:*********:** ***** *:******** ::*.******

gal LKNLYFLYLIELRALSKVLPFFERPGFQLYTGNQSHDAEIKNLLLEVLHLAKSFPLHFDE

mus LRNLYFLYLIELRALSKVLPFFERPDFQLFTGNKVQDAENKALLLEILHEIKSFPLHFDE

hum LKNLYFLYLIELRALSKVLPFFERPDFQLFTGNKIQDEENKMLLLEILHEIKSFPLHFDE

pan LKNLYFLYLIELRALSKVLPFFERPDFQLFTGNKIQDEENKMLLLEILHEIKSFPLHFDE

*:***********************.***:***: :* * * ****:** *********

gal NSFFAGNKKEAAKLKEEVRLHFKNISKIMDCVGCFKCRLWGKLQTQGLGTALKILFSESL

mus NSFFAGDKNEAHKLKEDFRLHFRNISRIMDCVGCFKCRLWGKLQTQGLGTALKILFSEKL

hum NSFFAGDKKEAHKLKEDFRLHFR**N**ISRIMDCVGCFKCRLWGKLQTQGLGTALKILFSEKL

pan NSFFAGDKKEAHKLKEDFRLHFRNISRIMDCVGCFKCRLWGKLQTQGLGTALKILFSEKL

******:*:** ****:.****:***:*******************************.*

gal IEKIPESGPSYGFQLTRQEIVALFNAFGRISTSVRELENFRAILRTMR

mus IANMPESGPSYEFQLTRQEIVSLFNAFGRISTSVRELENFRHLLQNVH

hum IANMPESGPSYEFHLTRQEIVSLFNAFGRISTSVKELENFRNLLQNIH

pan IANMPESGPSYEFHLTRQEIVSLFNAFGRISTSVKELENFRNLLQNIH

* ::******* *:*******:************:****** :*:.::

1. Il-6 receptor

gal MARPPGLLRAALLLLTAASSAPRRRCGPVALPQDTVLGRPGANVTLLCREREPPN-GTVL

mus MLTVGCTLLVA-LLAAPAVALVLGSCRALEVANGTVTSLPGATVTLICPGKEAAGNVTIH

hum MLAVGCALLAA-LLAAPGAALAPRRCPAQEVARGVLTSLPGDSVTLTCPGVEPED**N**ATVH

pan MLAVGCALLAA-LLAAPGAALAPRRCPAQEVARGVLTSLPGDSVTLTCPGVEPEDNATVH

* * .* ** : . : * : ...: . ** .*** * * . *:

gal WSGR--------RRALGGGNALLLGGLRPEDAGRYSCHLGGHTLRTVRLLVEEPPEPPHV

mus WVYS----GSQNREWTTTGNTLVLRDVQLSDTGDYLCSLNDHLVGTVPLLVDVPPEEPKL

hum WVLRKPAAGSHPSRWAGMGRRLLLRSVQLHDSG**N**YSCYRAGRPAGTVHLLVDVPPEEPQL

pan WVLRKPAAGSHPSRWAGMGRRLLLRSVQLHDSGNYSCYRAGRPAGTVHLLVDVPPEEPQL

* . *. *:* .:: *:* * * .: ** ***: *** *::

gal SCSRRSHDKDVLCEWRPRASPAPGTRAVLWMKRRFTME--NATEQRCHFYSAAQKFVCRV

mus SCFRKNPLVNAICEWRPSSTPSPTTKAVLFAKKINTTNGKSDFQVPCQYSQQLKSFSCQV

hum SCFRKSPLSNVVCEWGPRSTPSLTTKAVLLVRKFQNS-PAEDFQEPCQYSQESQKFSCQL

pan SCFRKSPLSNVVCEWGPRSTPSQTTKAVLLVRKFQNS-PAEDFQEPCQYSQESQKFSCQL

** *:. :.:*** * ::*: *:*** :: . . : *:: . :.* *::

gal KVPPGTDDTKALVVSVCVSSRAGSAAAEDRIFTLNGILKPDPPLNVTVEAVERSPQRLCV

mus EILEGD--KVYHIVSLCVANSVGSKSSHNEAFHSLKMVQPDPPANLVVSAIPGRPRWLKV

hum AVPEGD--SSFYIVSMCVASSVGSKFSKTQTFQGCGILQPDPPA**N**ITVTAVARNPRWLSV

pan AVPEGD--SSFYIVSMCVASSVGSKFSKTQTFQGCGILQPDPPANITVTAVARNPRWLSV

: * . :**:**:. .** :. . * :::**** *:.* *: *: * *

gal RWSYPPSWDPRFYWLRFQVRYRPEPAPNFTQVD-QVTRTWLDIRDAWRGMRHVVQVRAQE

mus SWQHPETWDPSYYLLQFQLRYRPVWSKEFTVLLLPVAQYQCVIHDALRGVKHVVQVRGKE

hum TWQDPHSWNSSFYRLRFELRYRAERSKTFTTWMVKDLQHHCVIHDAWSGLRHVVQLRAQE

pan TWQDPHSWNSSFYRLRFELRYRAERSKTFTTWMVKDLQHHCVIHDAWSGLRHVVQLRAQE

*. * :*: :* *:*::*** : ** : *:** *::****:*.:*

gal EFGHGAWSEWSREAVGTPWTEPRDVTEMGLYSSQFPAEDDPYGYGATLPPELFGDDTADD

mus ELDLGQWSEWSPEVTGTPWIEPRTTPAGILWNPTQVSVEDSANHED----QYE---SSTE

hum EFGQGEWSEWSPEAMGTPWTESRSPPAENEVSTPMQALTT-NKDDD----NIL---FRDS

pan EFGQGEWSEWSPEAMGTPWTESRSPPAENEVSTPTQALTT-NKDDD----NIL---FRDS

*:. * ***** *. **** * * . : : .

gal AGGAVLEAAVRSPTSPYALLVAGGSLLLAIILCFAIGMRYKQRARAQRGAKLEGGGQHP-

mus ATSVLAPVQESSSMSLPTFLVAGGSLAFGLLLCVFIILRLKQKWKSEAEK--ESKTTSPP

hum ANATSLPVQDSSSVPLPTFLVAGGSLAFGTLLCIAIVLRFKKTWKLRALK--EGKTSM--

pan ANATSLPVQDSSSVPLPTFLVAGGSLAFGTLLCIAIVLRFKKTWKLRALK--EGKTSM--

* .. . * ::******* :. :**. * :* *: : . *.

gal --------MVPLCPPGSPLSATPLLS--------------------PAAPPGPLHVTNLD

mus PPPYSLGPLKP-----T-FLLVP----LLTPHSSGSDNTVNHSCLGVRDAQSPYDNSNRD

hum HPPYSLGQLVPERPRPT-PVLVPLISPPVSPSSLGSDNTSSHNRPDARDPRSPYDISNTD

pan HPPYSLGQLVPERPRPT-PVLVPLISPPVSPSSLGSDNTSSHNRPDARDPRSPYDISNTD

: * : .* .* . :* *

gal YFLSGK

mus YLFPR-

hum YFFPR-

pan YFFPR-

*::

1. Pancreatic lipase related

gal --------------MLRIGIFALFLLCTARGSEVCYDRLGCFTDDIPWSGTAERPIYRLP

mus MPMDVRGCLFPSVQMLLCWLVS-LLLATVGGKEVCYGHLGCFSNDKPWAGMIQRPSKIFP

hum --------------MLPPWTLGLLLLATVRGKEVCYGQLGCFSDEKPWAGTLQRPVKLLP

pan -------------MMLPPWTLGLLLLATVRGKEVCYGQLGCFSDEKPWAGTLQRPVKLLP

** .. :**.*. *.****.:****::: **:* :** :*

gal WSPEKIGTQFLLHTRENGNSNQEISAVNPSTIGSSNFKTSRKTRFVVHGFIDEGEEGWTS

mus WSPEDIDTRFLLYTNENPNNYQIISATDPATINASNFQLDRKTRFIIHGFIDKGEEGWLL

hum WSPEDIDTRFLLYTNENPNNFQLITGTEPDTIEASNFQLDRKTRFIIHGFLDKAEDSWPS

pan WSPEDIDTRFLLYTNENPNNFQLITGTEPDTIEASNFQLDRKTRFIIHGFLDKAEDSWPS

****.*.*:***:*.** *. * *:..:* ** :***: .*****::***:*:.*:.*

gal DLCKRMLTVEDVNCIAVDWKKGARCQYSQASNNVRVVGAEIAYFISVLADQYSYSSANVH

mus DMCKKMFQVEKVNCICVDWKRGSRTEYTQASYNTRVVGAEIAFLVQVLSTEMGYSPENVH

hum DMCKKMFEVEKVNCICVDWRHGSRAMYTQAVQNIRVVGAETAFLIQALSTQLGYSLEDVH

pan DMCKKMFEVEKVNCICVDWSHGSRAMYTQAVQNIRVVGAETAFLIQALSTQLGYSLEDVH

*:**:*: **.****.*** :*:* *:** * ****** *:::..*: : .** :**

gal IIGHSLGAHVAGEAGKRRPG-VGRITGLDPAQPYFQDTPIEVRLDKSDAEFVDVIHTDTA

mus LIGHSLGSHVAGEAGRRLEGHVGRITGLDPAEPCFQGLPEEVRLDPSDAMFVDVIHTDSA

hum VIGHSLGAHTAAEAGRRLGGRVGRITGLDPAGPCFQDEPEEVRLDPSDAVFVDVIHTDSS

pan VIGHSLGAHTAAEAGRRLGGRVGRITGLDPAGPCFQDEPEEVRLDPSDAVFVDVIHTDSS

:******:*.*.***:* * ********** * **. * ***** *** ********::

gal PIIPNLGFGMAQAIGHLDFYPNGGVEMPGCDKNPLSQIIDLDGIWEGTRDFVACNHLRSY

mus PIIPYLGFGMSQKVGHLDFFPNGGKEMPGCQKNILSTIVDINGIWEGTRNFAACNHLRSY

hum PIVPSLGFGMSQKVGHLDFFPNGGKEMPGCKKNVLSTITDIDGIWEGIGGFVSCNHLRSF

pan PIVPSLGFGMSQKVGHLDFFPNGGKEMPGCKKNVLSTIIDIDGIWEGISGFVACNHLRSF

**:* *****:* :*****:**** *****.** ** * *::***** .*.:******:

gal KYYSDSIVYPDGFLGYACGSYDAFKE-GCFPCPSGGCPSMGHYADKFKGKTSGSFVKLYL

mus KYYASSILNPDGFLGYPCSSYEKFQHNDCFPCPEQGCPKMGHYADQFEGKTATVEQTFFL

hum EYYSSSVLNPDGFLGYPCASYDEFQESKCFPCPAEGCPKMGHYADQFKGKTSAVEQTFFL

pan EYYSSSIVNPDGFLGYPCASYDEFQESKCFPCPAEGCPKMGHYADQFKGKTSAVEQTFFL

:**:.*:: ******* *.**: *:. ***** ***.******:*:***: .::*

gal NTAEAKDFPLWRYKVSVKLSGSSKVKGYVNIALYGNDGNTRQHQIFEGSLQPDNTYTAFV

mus NTGDSGNFTRWRYKVSVTLSGAKKLSGYILVALYGCNGNSKQYEVFKGSLQPEARYIRDI

hum NTGESG**N**FTSWRYKVSVTLSGKEKVNGYIRIALYGSNENSKQYEIFKGSLKPDASHTCAI

pan NTGESGNFTSWRYKVSVTLSGKEKVNGYIKIALYGSNENSKQYEIFKGSLKPDASHTCAI

**.:: :* *******.*** .*:.**: :**** : *::*:::*:***:*: : :

gal DAEHKVGKVTKVKFLWNNNAVNPTLPKLGAATATVQVGETGEVFNFCGSETVRENVLQTL

mus DVDVNVGEIQKVKFLWNNKVINLFRPTMGASQITVQRGKDGKEFNFCSSNTVHEDVLQSL

hum DVDFNVGKIQKVKFLWNKRGI**N**LSEPKLGASQITVQSGEDGTEYNFCSSDTVEENVLQSL

pan DVDFNVGKIQKVKFLWNKRGINLSEPKLGASQITVQSGEDGTEYNFCSSDTVEENVLQSL

*.: :**:: *******:. :* *.:**: *** *: * :***.*:**.*:***:*

gal TAC

mus YPC

hum YPC

pan YPC

*

1. Interstitial collagenase

gal MKVLS--LLLLLYAAVSSAFPVAPEKEDEGKNINLVETYLQNFYNLQKDHRPHLRQGGKN

mus MPSLP--LLLLLWAASSYSFPVFHNG--DRQNVETVWKYLENYYNLGKNMQA-KNVNGKE

hum MHSFPPLLLLLFWGVVSHSFPATLET--QEQDVDLVQKYLEKYYNLKNDGRQVEKRRNSG

pan MHSFPPLLLLLFWGVVSHSFPATLET--QEQDVDLVQKYLEKYYNLKNDGRQVEKRRNSG

* : ****::.. * :**. : : :::: * .**:::*** :: : . ..

gal HLAEKLKEMQEFFGLQVTGKPDRDTLEMMNKPRCGVPDVEQYVFTPGNPKWKKNNLTYRI

mus MMAEKLRQMQQLFGLKVTGNSDPETLRAMKKPRCGVPDVAPYAITHNNPRWTKTHLTYSI

hum PVVEKLKQMQEFFGLKVTGKPDAETLKVMKQPRCGVPDVAQFVLTEGNPRWEQTHLTYRI

pan PVVEKLKQMQEFFGLKVTGKPDAETLKVMKQPRCGVPDVAQFVLTEGNPRWEQTHLTYRI

:.***::**::***:***: * :**. *::******** :.:* .**:* :.:*** *

gal VNYTTKMRQTDVDEAIQKALKVWSSVTPLTFQKTEDKIADIMISFAYRDHNDNSPFDGPN

mus LNYTPYLPKAVVEDAIARAFRVWSDVTPLTFQRVFEEEGDIVLSFHRGDHGDNNPFDGPN

hum E**N**YTPDLPRADVDHAIEKAFQLWSNVTPLTFTKVSEGQADIMISFVRGDHRDNSPFDGPG

pan ENYTPDLPRADVDHAIAKAFQLWSNVTPLTFTKVSEGQADIMISFVRGDHRDNSPFDGPG

*** : :: *:.** :*:::**.****** :. : .**::** ** **.*****.

gal GLLAHAFQPGEGLGGDVHLDEEETWTKDGRGYNLFIVVAHELGHSLGLSHSNDPGALMYP

mus YKLAHTFQPGPGLGGDVHYDLDETWTNSSENFNLFYVTAHELGHSLGLTHSSDIGALMFP

hum GNLAHAFQPGPGIGGDAHFDEDERWTNNFREYNLHRVAAHELGHSLGLSHSTDIGALMYP

pan GNLAHAFQPGPGIGGDAHFDEDERWTNNFREYNLYRVAAHELGHSLGLSHSTDIGALMYP

***:**** *:***.* * :* **:. . :**. *.**********:**.* ****:*

gal NYAYTDPKEFLLPQDDIDGIQAIYGQSDDAVQPTGPTTPQVCDPNLTFDAITTLRGEMIF

mus SYTWYT-EDFVLNQDDINRIQDLYGPSPNPIQPTGATTPHPCNGDLTFDAITTFRGEVFF

hum SYTFS--GDVQLAQDDIDGIQAIYGRSQNPVQPIGPQTPKACDSKLTFDAITTIRGEVMF

pan SYTFS--GDVQLAQDDIDGIQAIYGRSQNPVQPIGPQTPKVCDSKLTFDAITTIRGEVMF

.*:: :. * ****: ** :** * : :** * **: *: .********:***::*

gal FKGRYMLRKHPERSETELNFISLFWPNLPSGIQAAYENIERDEVLLFKEDKYWVIRGYDI

mus FKGRFYIRVNRFMPEPELNLIGILWPNLPVKLDAAYEASMIDQVRYFKGSKVWAVQEQSV

hum FKDRFYMRTNPFYPEVELNFISVFWPQLPNGLEAAYEFADRDEVRFFKGNKYWAVQGQNV

pan FKDRFYMRTNPFYPEVELNFISVFWPQLPNGLEAAYEFADRDEVRFFKGNKYWAVQGQNV

**.*: :* : * ***:*.::**:** ::**** *:* ** .* *.:: .:

gal VRGYPKPIYR-LGFPKTVKRVNAAYNDETTGKTYFFVADRYWRYDENKKSMDHGYPRKII

mus LRGFPRDIHSFFGFPSNVTHIDAAVCEEETGKTYFFVDHMYWRYDENTQSMDPGYPRLTA

hum LHGYPKDIYSSFGFPRTVKHIDAALSEENTGKTYFFVANKYWRYDEYKRSMDPGYPKMIA

pan LHGYPKDIYSSFGFPRTVKHIDAALSEENTGKTYFFVANKYWRYDEYKRSMDPGYPKMIA

::*:*: *: :*** .*.:::** :* ******** . ****** .:*** ***:

gal HDFGKIG-RVDAAFQKDGYVYFFHKTTQFQFDPRAKRIVSHMKSISWFNC---

mus EDFPGIDDKVDDVFQKGENFYFFHQSVQHRFNLQIRRVDDSRDSSTWFNC---

hum HDFPGIGHKVDAVFMKDGFFYFFHGTRQYKFDPKTKRILTLQKANSWFNCRKN

pan HDFPGIGHKVDAVFMKDGFFYFFHGTRQYKFDPKTKRILTLQKANSWFNCRKN

.** *. :** .* *. .**** : *.:*: : :*: .: :****

1. Properdin

gal MDNKGIPTFVLLNTTGILLPARVDHLELLSISGEPLKTLPVKYYPDRKPYGLWNISDFIP

mus ------------------------------------------------------------

hum ------------------------------------------------------------

pan ------------------------------------------------------------

gal PDEAFFLKIMGYDKDGYIFQRVSSVSFSSITPDAPKVTMPNKTPGYYSQPGSVPCHVESL

mus ------------------------------------------------------------

hum ------------------------------------------------------------

pan ------------------------------------------------------------

gal IPFTQHFTKNGVKLGVDQLFKESSSMSWDINRVSLSDEGFYECIANSTAGTGHAQTFLDV

mus ------------------------------------------------------------

hum ------------------------------------------------------------

pan ------------------------------------------------------------

gal SEPPPVIQVPNNVTAVLREGAILTCLVVSTMRYNLTWQRNGRDVRLQEPLRMRVMSNLSL

mus ------------------------------------------------------------

hum ------------------------------------------------------------

pan ------------------------------------------------------------

gal EVKAVQFTDAGKYNCVASNKDGSTTASVFLTVQEPPRIVISPKDQTFVEGSEVSIRCSAT

mus ------------------------------------------------------------

hum ------------------------------------------------------------

pan ------------------------------------------------------------

gal GYPKPTVVWTHNEMFIIGSNRYRLTPEGTLIIRQAIPKDAGVYGCLASNSAGTEKQTSIL

mus ------------------------------------------------------------

hum ------------------------------------------------------------

pan ------------------------------------------------------------

gal TYIEGPTVTIFQSEILVALGDTTVMECKTTGIPHPQVKWFKGDLELRASAFLIIDTHRGL

mus ------------------------------------------------------------

hum ------------------------------------------------------------

pan ------------------------------------------------------------

gal LKIQETQELDAGDYTCVATNDAGRASGKITLDVGSPPVFIQEPSDESVDIGSNITLPCYV

mus ------------------------------------------------------------

hum ------------------------------------------------------------

pan ------------------------------------------------------------

gal QGYPEPKVKWRRLNGASLFSRPLAVSFISQLRTGALSINNLWVNDEGSYVCEAENQFGRI

mus ------------------------------------------------------------

hum ------------------------------------------------------------

pan ------------------------------------------------------------

gal QSQPATITVTGLVAPLIGISPATANVIEGQQLTLPCVLLAGNPIPDRKWIKNSMVLVSNP

mus ------------------------------------------------------------

hum ------------------------------------------------------------

pan ------------------------------------------------------------

gal YINVRSDGSLHLERVRLQDGGDYTCMASNVAGTNNKTTTVNVYVLPIVQHGQQIFSTIEG

mus ------------------------------------------------------------

hum ------------------------------------------------------------

pan ------------------------------------------------------------

gal IPVTLPCKASGVPKPSITWSKKGEVIFPSNEKFSAGSDGSLYVVSPEGEETGEYVCTATN

mus ------------------------------------------------------------

hum ------------------------------------------------------------

pan ------------------------------------------------------------

gal AAGYATRKVQLTVYVKPRVSRPGDQQGHGPVEISVITGDDVTLPCEVKSLPPPIITWAKE

mus ------------------------------------------------------------

hum ------------------------------------------------------------

pan ------------------------------------------------------------

gal TQLISPFSLRHTFLLSGSMKISETQVSDSGMYFCVATNIAGNVTQSVKLSVHVPPKIQRG

mus ------------------------------------------------------------

hum ------------------------------------------------------------

pan ------------------------------------------------------------

gal PQIMKVQAGHRVDIPCSAQGNPPPAITWFRGSSAVPIGSGQPTHSLGGALSISNVQLSNA

mus ------------------------------------------------------------

hum ------------------------------------------------------------

pan ------------------------------------------------------------

gal GIYRCIASNAAGSDTSEITIQVQEPPTMDDLDPQYNNPFQERVVNQRIAFPCPVKGTPKP

mus ------------------------------------------------------------

hum ------------------------------------------------------------

pan ------------------------------------------------------------

gal VIKWLHNGRELTGREPGISILENDMLLIIASITPSDNGEYICVATNEAGRTERKYNLEVH

mus ------------------------------------------------------------

hum ------------------------------------------------------------

pan ------------------------------------------------------------

gal VPPEIRDQERVTNTSVVVNHPISLFCEVFGNPFPVISWYKEDIQVVESNTLHQILHNGKI

mus ------------------------------------------------------------

hum ------------------------------------------------------------

pan ------------------------------------------------------------

gal LKLLKATVDDAGQYSCKAINVAGSSEKLFNLYILVPPSIIGADTPSEIAVILNQETSLEC

mus ------------------------------------------------------------

hum ------------------------------------------------------------

pan ------------------------------------------------------------

gal RAKGFPFPGIHWFKDSMPLFLGDPNVELLEKGQVLHIKSARMVDKGHYQCSATNAAGKQI

mus ------------------------------------------------------------

hum ------------------------------------------------------------

pan ------------------------------------------------------------

gal KEVKLIIHVPPSIKGGNITTEVSALLNNLINLDCETKGIPVPTITWYKEGRRIISSPQAL

mus ------------------------------------------------------------

hum ------------------------------------------------------------

pan ------------------------------------------------------------

gal YVDRGQFLQIPHAQVSDSAKYTCHVTNAAGAAEKTYEVDVYVPPVIEGDAETAQSRQVVA

mus ------------------------------------------------------------

hum ------------------------------------------------------------

pan ------------------------------------------------------------

gal GNSLTLECKAAGNPSPLLTWLKDGVPVKASDNLRVVSGGKKLEILNAVEADRGQYLCVAT

mus ------------------------------------------------------------

hum ------------------------------------------------------------

pan ------------------------------------------------------------

gal SIAGEQEIKYGVEILVPPFVEGGDEFLDYIVVLHSPLELDCSATGTPLPTITWLKGGQPI

mus ------------------------------------------------------------

hum ------------------------------------------------------------

pan ------------------------------------------------------------

gal EEGTGHKILLSGQKFLISRAQVSDTGHYKCVAVNVAGEHEREFVVTVHVPPTIKSAGTSE

mus ------------------------------------------------------------

hum ------------------------------------------------------------

pan ------------------------------------------------------------

gal RAVVVHKAVTLQCIANGIPSPSITWLKDGQPVNTARGNTRLESSGRVLQVAEALLEDAGR

mus ------------------------------------------------------------

hum ------------------------------------------------------------

pan ------------------------------------------------------------

gal YTCVAINAAGEAQQHIRLRVHEPPSLEDAGKMLNETVVVNNPIHLECRALGNPLPAITWY

mus ------------------------------------------------------------

hum ------------------------------------------------------------

pan ------------------------------------------------------------

gal KDSHSLTSAASVTFLNKGQVLEIEGAQISDTGIYKCVAVNIAGTAELSYSLQVHVPPSIS

mus ------------------------------------------------------------

hum ------------------------------------------------------------

pan ------------------------------------------------------------

gal DSSDTVTAVVNNLVRMECEARGIPAPILTWLKDGSPVSSFSDGLQILSGGRVLVLTSAQI

mus ------------------------------------------------------------

hum ------------------------------------------------------------

pan ------------------------------------------------------------

gal SDTGKYTCVAVNAAGESQRDIDLRVYVPPNIMGEEQNVSVLISQAVELLCQSNAVPLPML

mus ------------------------------------------------------------

hum ------------------------------------------------------------

pan ------------------------------------------------------------

gal MWLKDGRPLLNKPGLSISEDGSVLKIEGAQVQDTGRYTCEATNVAGKTKKNYNVNIWVPP

mus ------------------------------------------------------------

hum ------------------------------------------------------------

pan ------------------------------------------------------------

gal SIYGSDDTSQLTVIEGSLISLICESTGIPPPSLTWKKSGSPLVADQSGRVRILSGGRQLQ

mus ------------------------------------------------------------

hum ------------------------------------------------------------

pan ------------------------------------------------------------

gal ISVAEMSDAASYICIASNVAGSAKKEYSLQVYTRPVILDSGSYPSEVVAAQGSEISLECK

mus ------------------------------------------------------------

hum ------------------------------------------------------------

pan ------------------------------------------------------------

gal AQGIPEPAVTWMKDGRPLVSGRDVAVLHDGHFLLLRNIQVSDTGHYVCVAANVAGLYDRK

mus ------------------------------------------------------------

hum ------------------------------------------------------------

pan ------------------------------------------------------------

gal YDLNVHVPPDIAGDLQLPENISTVEKNPISLVCEASGIPLPSVMWLKNGWPVTSNTSVQI

mus ------------------------------------------------------------

hum ------------------------------------------------------------

pan ------------------------------------------------------------

gal LSGGRTLRLTHTTVSDEGHYTCVVTNAAGEARKDFYLSVLVPPGIVDENKQEDMKVKEKN

mus ------------------------------------------------------------

hum ------------------------------------------------------------

pan ------------------------------------------------------------

gal SVTLTCEVIGNPVPQITWIKDGQPLMEDKDHRFLSSGRFLQITNAQVSDTGRYTCVASNT

mus ------------------------------------------------------------

hum ------------------------------------------------------------

pan ------------------------------------------------------------

gal AGDKSKSYFLNVLVSPTIVGADSHGSAEEVTVILNSPTSLVCEAYSYPPATITWLKDGSL

mus ------------------------------------------------------------

hum ------------------------------------------------------------

pan ------------------------------------------------------------

gal LESNRNIHILPGGRILQILSAQKHSAARYTCIATNEAGETMKHYEVQVYIPPTINRGDVP

mus ------------------------------------------------------------

hum ------------------------------------------------------------

pan ------------------------------------------------------------

gal GMDLSPKEMKIKINHSLTLECEAHAVPAAAISWYKDGQPLKPDDRVIIQASGHTLQITEA

mus ------------------------------------------------------------

hum ------------------------------------------------------------

pan ------------------------------------------------------------

gal QVSDTGRYTCLASNIAGEDEVEFDINIQVPPSFQKPYRGWESGNMVDTGRGGENKDVIIN

mus ------------------------------------------------------------

hum ------------------------------------------------------------

pan ------------------------------------------------------------

gal NPLSLYCETDAVPPPVLTWYKDGYPLNSSDKVLILPGGRVFQIPRARAEDAGRYMCVAVN

mus ------------------------------------------------------------

hum ------------------------------------------------------------

pan ------------------------------------------------------------

gal EAGEDSIHYNVHVLLPPSIRGADGDLPEEVNVLGNKVAVMDCVTSGSPSPSITWEKDGHL

mus ------------------------------------------------------------

hum ------------------------------------------------------------

pan ------------------------------------------------------------

gal LAEDNKHSFLSNGRRLQILNSQVTDTGRYVCVVENIAGRAKKYFNLNVHVPPSVVGANPE

mus ------------------------------------------------------------

hum ------------------------------------------------------------

pan ------------------------------------------------------------

gal NLTVVVNNLISLTCEVTGFPPPDLSWLKNGKPVSLNTNTFIVPGARTLQIPQAKLSDDGE

mus ------------------------------------------------------------

hum ------------------------------------------------------------

pan ------------------------------------------------------------

gal YTCIARNHAGESQKKSFLTVLVPPGIKDHSGTSVTVFNVRVGTPVMLECKASAIPPPVIT

mus ------------------------------------------------------------

hum ------------------------------------------------------------

pan ------------------------------------------------------------

gal WYKNRRMISESANVEILADGQTLQIKGAEVSDTGQYVCKAINIAGRDDKNFHLNVYVPPN

mus ------------------------------------------------------------

hum ------------------------------------------------------------

pan ------------------------------------------------------------

gal IEGPEEELVIETISNPVAFICDATGIPPPTLVWLKNGKPLENSNSLDVHIFSGGSRLQIA

mus ------------------------------------------------------------

hum ------------------------------------------------------------

pan ------------------------------------------------------------

gal HSQLLDSGTYTCIASNVEGKAQKRFVLSVQVPPSIVGSEMPREVGVLLGEGIQLVCNATG

mus ------------------------------------------------------------

hum ------------------------------------------------------------

pan ------------------------------------------------------------

gal VPMPVVQWLKDGKTVASDNLERIRVAPDGSTLNIFRALTSDTGKYTCVATNPAGEEDQIF

mus ------------------------------------------------------------

hum ------------------------------------------------------------

pan ------------------------------------------------------------

gal NLSVYVPPTITNNKSEPEDLAALLESSINIGCAATGMPSPQINWLKNGLPLSVSSRIRLL

mus ------------------------------------------------------------

hum ------------------------------------------------------------

pan ------------------------------------------------------------

gal SAGQILRLVRVQISDAGVYTCVASSRAGVDNKHYNLQIFVPPSLDNARGTEEVTVAKGSS

mus ------------------------------------------------------------

hum ------------------------------------------------------------

pan ------------------------------------------------------------

gal ASLKCFTDGAPPPAMSWFKNGHPLSLGAHQNLNNQGMVLHFVEAEIGDVGKYTCVAANKA

mus ------------------------------------------------------------

hum ------------------------------------------------------------

pan ------------------------------------------------------------

gal GDVSKHFSLKVLEPPQINGSGQPEELSVIVNNPLELLCISTGIPIPKISWMKDGRPLLQN

mus ------------------------------------------------------------

hum ------------------------------------------------------------
[truncated: 58,435 more chars]
