# Supplementary material for: Mutually exclusive locales for N-linked glycans and disorder in human glycoproteins
Source: Sci Rep. 2020 Apr 8;10:6040. doi: 10.1038/s41598-020-61427-y (PMC7142085; doi:10.1038/s41598-020-61427-y)
Supplement: Supplementary file 5 — Supplementary File 8. [file 41598_2020_61427_MOESM5_ESM.docx]

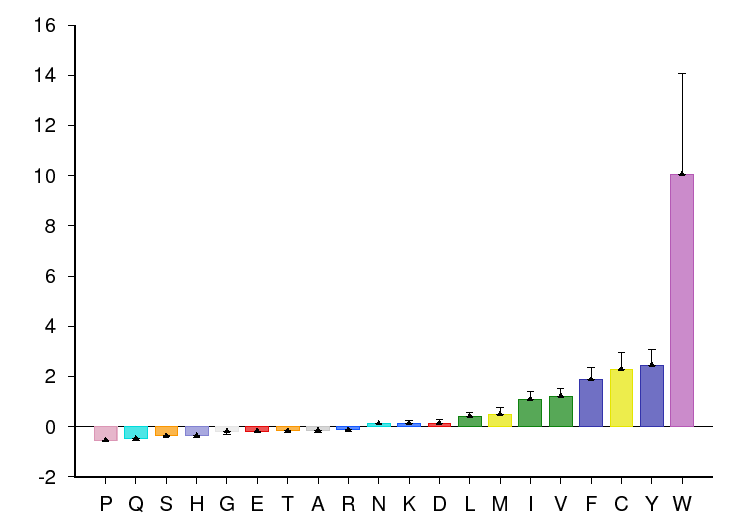


| P | 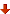Depleted. | P-value=0.000000 (≤0.002500) |
| --- | --- | --- |
| Q | 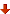Depleted. | P-value=0.000000 (≤0.002500) |
| S | 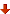Depleted. | P-value=0.000000 (≤0.002500) |
| H | Not significant. | P-value=0.006760 (>0.002500) |
| G | Not significant. | P-value=0.058744 (>0.002500) |
| E | Not significant. | P-value=0.115087 (>0.002500) |
| T | Not significant. | P-value=0.040166 (>0.002500) |
| A | Not significant. | P-value=0.191243 (>0.002500) |
| R | Not significant. | P-value=0.354007 (>0.002500) |
| N | Not significant. | P-value=0.226926 (>0.002500) |
| K | Not significant. | P-value=0.470264 (>0.002500) |
| D | Not significant. | P-value=0.452499 (>0.002500) |
| L | Not significant. | P-value=0.004018 (>0.002500) |
| M | Not significant. | P-value=0.195869 (>0.002500) |
| I | 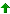Enriched. | P-value=0.000169 (≤0.002500) |
| V | 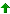Enriched. | P-value=0.000002 (≤0.002500) |
| F | 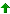Enriched. | P-value=0.000004 (≤0.002500) |
| C | 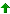Enriched. | P-value=0.000162 (≤0.002500) |
| Y | 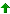Enriched. | P-value=0.000002 (≤0.002500) |
| W | 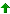Enriched. | P-value=0.000017 (≤0.002500) |

| Aromatic content | 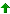Enriched. | P-value=0.000000 (≤0.001250) |
| --- | --- | --- |
| Charged residues | Not significant. | P-value=0.478704 (>0.001250) |
| Positively charged | Not significant. | P-value=0.849074 (>0.001250) |
| Negatively charged | Not significant. | P-value=0.454544 (>0.001250) |
| Polar (Zimmerman) | Not significant. | P-value=0.082493 (>0.001250) |
| Hydrophobic (Eisenberg) | 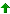Enriched. | P-value=0.000000 (≤0.001250) |
| Exposed (Janin) | 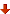Depleted. | P-value=0.000000 (≤0.001250) |
| Flexible (Vihinen) | 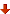Depleted. | P-value=0.000000 (≤0.001250) |
| Frequent in alpha hel. (N) | Not significant. | P-value=0.040496 (>0.001250) |
| Frequent in beta struc. (N) | 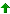Enriched. | P-value=0.000000 (≤0.001250) |
| Frequent in coils (N) | 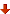Depleted. | P-value=0.000002 (≤0.001250) |
| High linker propensity (G-H) | Not significant. | P-value=0.016992 (>0.001250) |
| Disorder promoting (Dunker) | 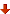Depleted. | P-value=0.000000 (≤0.001250) |
| Order promoting (Dunker) | 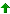Enriched. | P-value=0.000000 (≤0.001250) |
| Bulky (Zimmerman) | 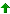Enriched. | P-value=0.000000 (≤0.001250) |

N-linked glycosites within ordered regions, when compared with N-glycosites within disordered regions showed an enrichment of aromatic, hydrophobic bulky and order-promoting amino acids and were depleted of flexible surface-exposed disorder-promoting residues that show frequency of presence in coil structures.
